# Rhodium-Catalyzed Desymmetric Addition of Boronic Acids to Malononitriles

**DOI:** 10.1021/jacs.5c23045

**Published:** 2026-04-12

**Authors:** Minghao Zhang, Qihao Zhang, Junjie Cao, Jun Joelle Wang, Jianchun Wang, Zhongxing Huang

**Affiliations:** † State Key Laboratory of Synthetic Chemistry, Shanghai Hong Kong Joint Laboratory in Chemical Synthesis, Department of Chemistry, 25809 The University of Hong Kong, Hong Kong, China; ‡ Shenzhen Grubbs Institute and Department of Chemistry, Guangming Advanced Research Institute, and Guangdong Provincial Key Laboratory of Catalysis, 255310 Southern University of Science and Technology, Shenzhen 518055, China; § Department of Chemistry, 26679 Hong Kong Baptist University, Kowloon, Hong Kong, China

## Abstract

Malononitrile is a privileged chemical feedstock for building complex nitrogen-containing molecules of interest. However, achieving high enantiocontrol in transformations of its derivatives is often challenging, as the spatial arrangement of the two linear nitriles impedes the chelative assistance commonly exploited in related multifunctionalized reactants. Here, we demonstrate a customized phosphoramidite ligand for a rhodium-catalyzed desymmetric addition of carbon nucleophiles to malononitriles. The chiral pocket, enclosed by a vaulted, chlorinated binaphthol skeleton and an iminostilbene motif, is proposed to restrict the C­(*ipso*)–C­(α) bond rotation of complexed nitrile and thus enforces precise substrate orientation. Consequently, β-ketonitriles bearing a quaternary stereocenter are obtained in good enantiopurity with versatile reactivity arising from the remaining nitrile, the newly generated ketone, and diverse substituents retained from the malononitrile reactant.

## Graphical abstract

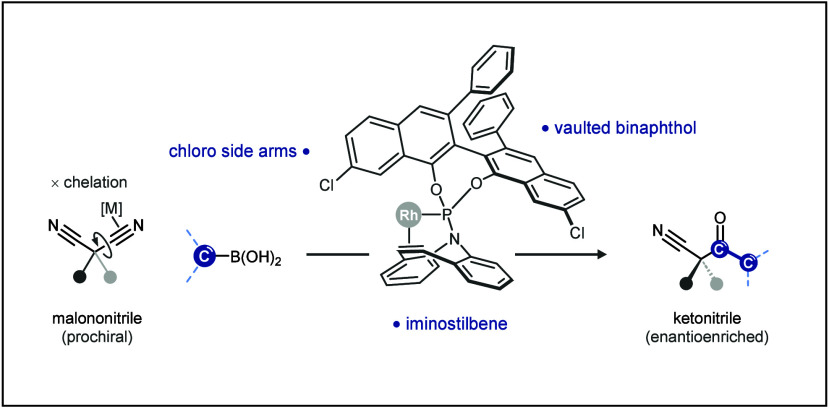

Preparation of quaternary stereocenters from active methylene compounds (AMCs) benefits significantly from their large production volume, facile methylene functionalization, and the versatile reactivity imparted by the two electron-withdrawing groups (Figure 1A).^1^ Traditionally, racemic monosubstituted AMCs have served as prominent nucleophiles for asymmetric allylation, conjugate addition, and other C–C bond-forming transformations.^2^ More recently, an alternative ‘quaternary-to-quaternary’ strategy based on desymmetric functional group interconversion (FGI) of prochiral disubstituted AMCs has gained traction,^3^ largely fueled by the ease of installing two substituents without stereochemical consideration and by the broad range of transformations available for imposing enantiocontrol.

**Figure 1:**
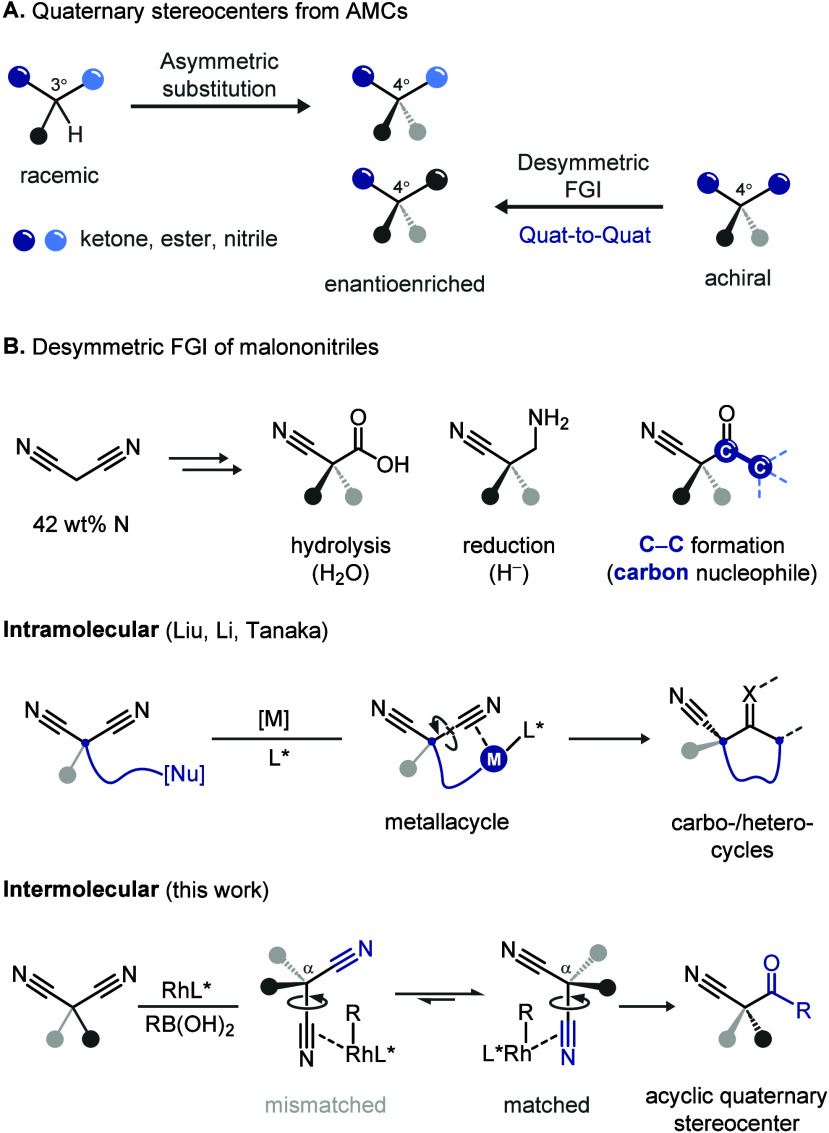
(A) Synthesis of quaternary stereocenters from active methylene compounds. (B) Desymmetric functional group interconversion (FGI) of malononitriles.

Malononitrile is a distinctive starting material for desymmetrization substrates, offering both notable advantages and inherent challenges (Figure 1B).^4^ With the highest nitrogen content among AMCs, it serves as a valuable precursor to nitrogen-rich bioactive molecules. However, the linear geometry of its triple bonds limits effective chelation to metal centers, making enantiocontrol more difficult compared with that of malonic esters or diketones, where σ-bond rotation is constrained through chelative assistance in metal-catalyzed desymmetrization. Thus, devising catalyst frameworks that can effectively differentiate between the enantiotopic nitriles is nontrivial.

Previous studies have shown that the enantiocontrol of asymmetric malononitrile hydrolysis can be tuned by interactions within enzyme active sites.^5,6^ A tailored cobalt-bisoxazoline complex has been reported to catalyze the desymmetric borohydride reduction of malononitriles.^7^ Beyond water and hydride delivery, desymmetric C–C bond-forming reactions of nitriles with carbon nucleophiles can substantially increase molecular complexity, rendering the carbon center chiral and enantioenriched with a pair of nitrogen- and oxygen-containing substituents with diverse yet distinct reactivities (i.e., nitrile and ketone). The power of this approach has been demonstrated by Liu and co-workers in multiple elegant syntheses of enantioenriched carbo- and heterocycles via the addition of a tethered and sometimes *in situ* generated nucleophile.^8^ Cycloaddition reactions of nitrile with a pair of internal and external unsaturated bonds were also developed toward chiral pyridines from malononitriles.^9,10^ Here, we report a complementary intermolecular boronic acid addition to malononitriles that affords β-ketonitriles with an acyclic quaternary stereocenter. While the metallacycle intermediates between the nitrile and a pendant nucleophile can assist the conformational restraint in an intramolecular addition, the complexation of the metal center with a single nitrile alone in an intermolecular desymmetrization is inadequate to restrict the rotation of its C­(*ipso*)–C­(α) bond. Hence, an intricate chiral pocket orchestrated by the ligand is particularly important to differentiate between the two enantiomeric orientations of α-substituents when a different nitrile is complexing.

We selected rhodium-catalyzed boronic acid addition as the platform for desymmetric C–C bond formation given its mild conditions and prior success in accessing ketones from nitriles.^11,12^ Particularly, studies by Reeves and co-workers demonstrated a high flexibility of the rhodium-catalyzed conditions toward malononitriles in tuning the reaction pathway between decyanation and addition.^12e,12f^ During our investigation of a desymmetric addition to malononitrile **1**, phosphoramidite ligand^13^ **L1** composed of a vaulted binaphthol (VANOL)^14^ and an iminostilbene motif was identified as the optimal ligand to deliver the β-ketonitrile **2** in excellent enantioselectivity and yield, with only a trace amount of decyanation product observed (Figure 2A). The indispensable role of the ancillary olefin in iminostilbene for metal chelation was demonstrated by Carreira and co-workers^15^ in their seminal development and corroborated here by the loss of enantiocontrol observed when a saturated analog (**L2**) was employed. The pair of substituents on the VANOL flanks is equally crucial. These side arms, in conjunction with the arene of the iminostilbene unit, are likely to reach out and modulate stereocontrol over the three α-substituents of the reacting nitrile by creating an optimally sized enclosure. Halogen atoms at these positions are particularly effective. Removing, relocating, or replacing them with bulkier groups (e.g., methyl, phenyl, or *tert*-butyl) diminished the enantioselectivity (Supplementary Figure S1). In comparison with the vaulted binaphthol scaffold, its biphenanthrol (VAPOL) counterpart (**L3**), with a more extended architecture, was detrimental to enantiocontrol. Meanwhile, the widely used 1,1′-binaphthol scaffolds (BINOL), with or without side arms (e.g., **L4** and **L5**), are ineffective for the desymmetrization.

**Figure 2:**
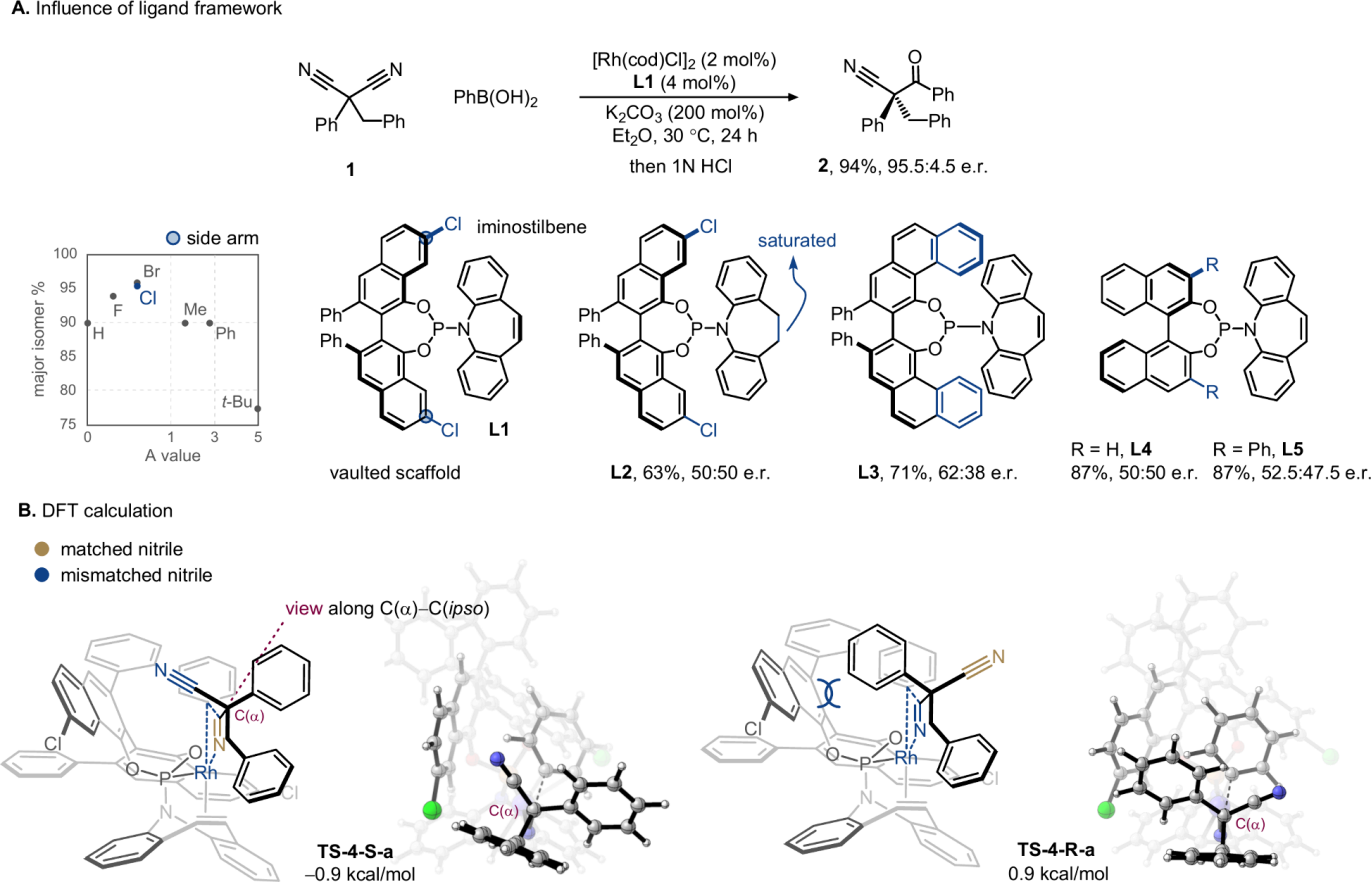
(A) Identification of a vaulted chiral ligand. The inset chart shows the relationship between the enantioselectivity of ligands with different side arms and their sterics in terms of cyclohexyl A-values. (B) Computational studies. The Gibbs free energies are given relative to Int-1 (structure shown in Supplementary Figure S8).

Subsequent density functional theory studies were built upon a common square-planar Rh­(I) complex chelated by both phosphine and olefin in the phosphoramidite ligand (Figure 2B). The anionic phenyl and reacting nitrile occupy the *trans* positions of the alkene and phosphine, respectively, to maximize ground-state stabilization. After the transmetalation and malononitrile association (Supplementary Figures S8 and S9), we were able to locate six four-membered transition states (**TS-4-S-a/b/c** and **TS-4-R-a/b/c**, Supplementary Figure S10),^16^ arising from three rotamers for each enantiotopic nitrile group upon binding to the rhodium center. In the lowest-energy, matched transition state (**TS-4-S-a**), the spectator nitrile is directed into a confined pocket enclosed by the chlorinated naphthalene, whereas the larger substituents are accommodated in a more open space. In contrast, the transition state of the lowest energy among the **TS-4-R** series (i.e., **TS-4-R-a**) points the phenyl ring toward the naphthalene ‘wall’, creating significant steric repulsion (Supplementary Figure S11). The noncovalent interaction (NCI) analysis of the pair of transition states also revealed only a weak π–π interaction during the addition, in line with the crossed-pair matrix probing experiments showing negligible impact of donor–acceptor π–π complementarity on stereoselectivity (Supplementary Figure S7). The calculated difference in energy between **TS-4-S-a** and **TS-4-R-a** is consistent with the observed enantiomeric ratio.

The intermolecular desymmetric addition can be readily scaled up to a gram scale with lowered catalyst loading and is compatible with assorted boronic acids (Table 1). Aryl nucleophiles with functional groups of distinct electronic properties at the *para* position can all proceed to give ketonitrile products in good enantiopurity (**3**–**7**), including a Lewis basic thioether (**6**) and an acid-labile acetal group (**7**). Other substitution patterns, including mono/di-*meta*-substitution (**8** and **9**) and fused arenes (**10**), are all tolerated, although an *ortho*-methyl group (**11**) was found to be disruptive to the enantiocontrol. Meanwhile, heterocyclic boronic acids (**12** and **13**) can serve as nucleophiles for addition. Notably, cyclic and acyclic olefinic boronic acids can also participate in desymmetric C–C bond formation and generate an unsaturated ketone suitable for further conjugate addition (**14**–**17**). Nevertheless, boron-based aryl and alkenyl nucleophiles other than boronic acids, such as pinacol/MIDA boronates and trifluoroborate salts, were found incompatible with the current conditions. Alkyl boronic acids failed to give the desymmetrization products as well.

**Table 1:**
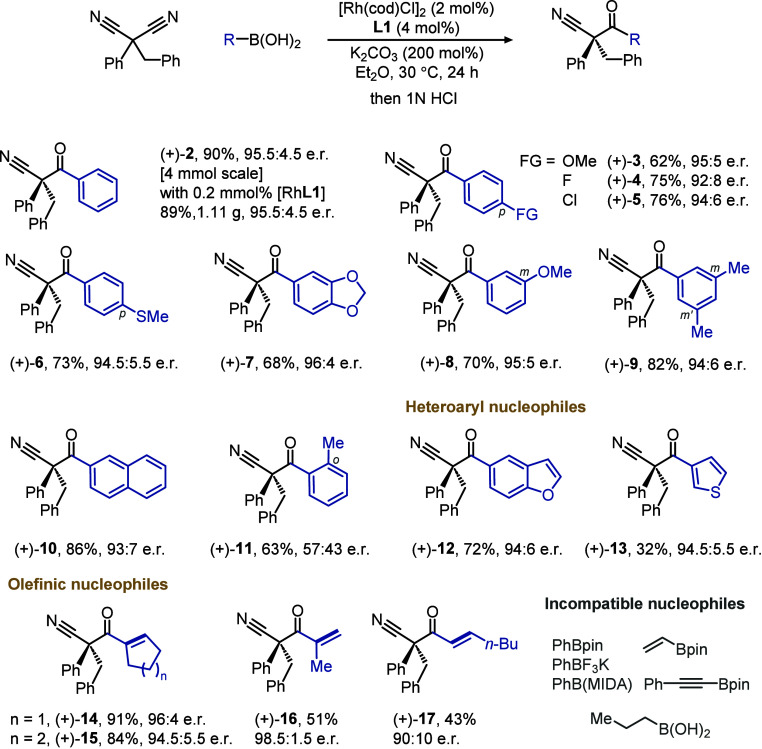
Scope of Boronic Acids

a Unless noted otherwise, the desymmetrization was run using malononitrile (0.2 mmol), boronic acid (0.4 mmol), [Rh­(cod)­Cl]_2_ (0.004 mmol), **L1** (0.008 mmol), and K_2_CO_3_ (0.4 mmol) in diethyl ether at 30 °C for 24 h. The reaction was quenched with 1 N HCl. See Supporting Information for detailed conditions.

With good structural and functional group compatibility, the desymmetrization unlocks the full potential of readily accessible, diversely substituted malononitriles (Table 2). For example, substitution patterns and functional groups of the arene directly connected to the α-position have only negligible influence on the catalyst’s performance (**18**–**22**). Its chiral pocket can also house arenes of distinct shapes, including biphenyl (**23**), naphthalene (**24** and **25**), and fluorene (**26**). Meanwhile, quaternary stereocenters substituted with a heterocycle (**27** and **28**) or containing a functionalized drug fragment (**29**) are also accessible.

**Table 2:**
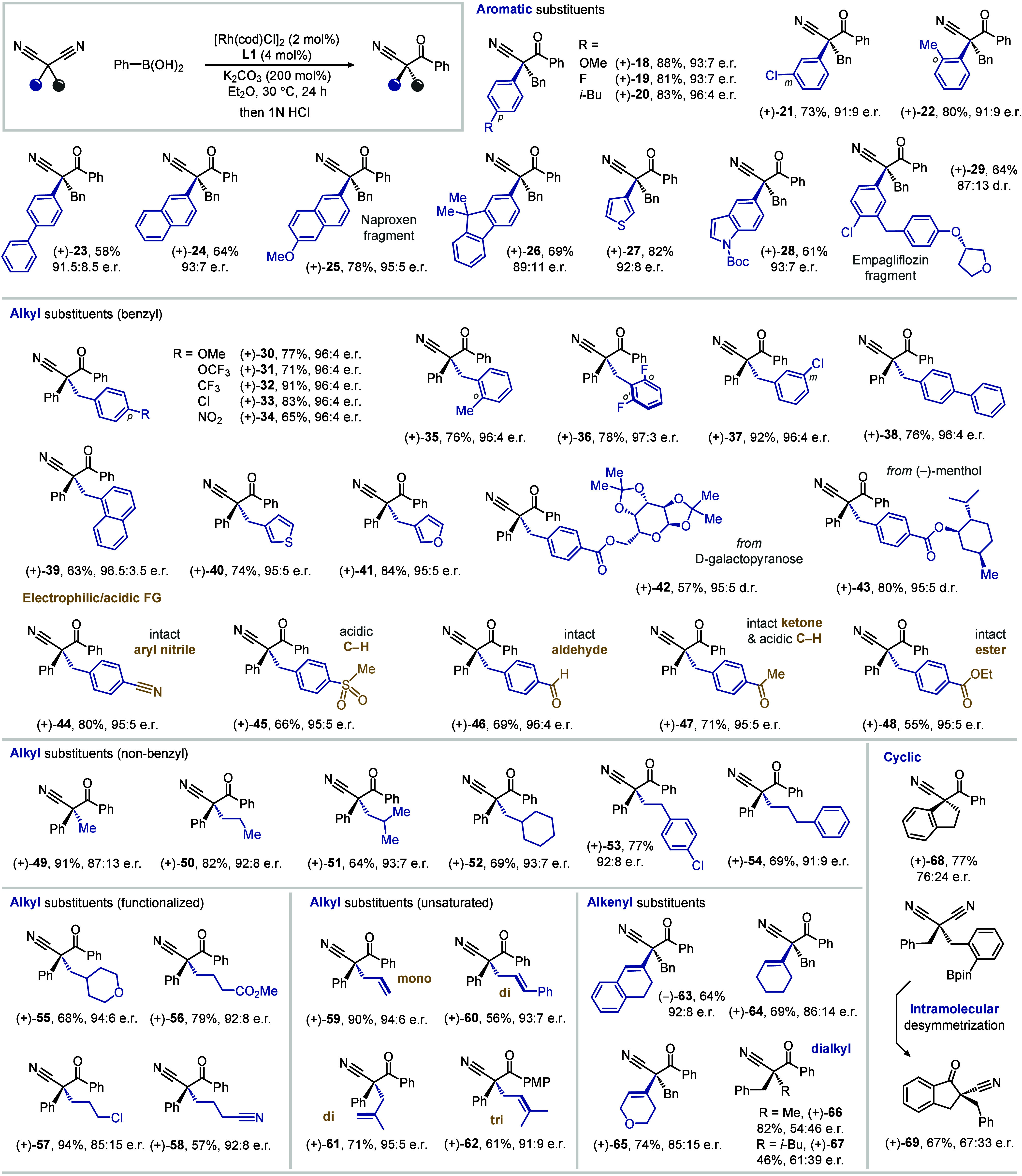
Scope of Malononitriles

a The desymmetrization conditions are the same as those in Table 1. See Supporting Information for detailed conditions.

An equally good scope of alkyl substituents was also observed. Particularly, diversely shaped and functionalized benzyl groups (**30**–**39**) are all compatible, together with heterocycles (**40** and **41**) and pendent natural metabolites (**42** and **43**). More importantly, the addition conditions demonstrated an excellent chemoselectivity toward malononitriles by tolerating a panel of electrophilic moieties as well as those containing acidic C–H bonds, including nitrile (**44**), sulfone (**45**), aldehyde (**46**), ketone (**47**), and ester (**48**). These functional groups, in turn, bestow the resulting quaternary stereocenters with rich reactivities for derivatization. The pronounced chemoselectivity observed for malononitrile is attributed to the mutually electron-withdrawing effect of its two nitrile groups. In support of this, a range of mononitriles, including aromatic, α-secondary, and α-tertiary variants, remain inert under the addition conditions (Supplementary Figure S6). In contrast, racemic α-fluoronitrile (**S85**) undergoes kinetic resolution with a moderate enantioselectivity.

The rhodium catalyst imposed effective enantiocontrol on malononitriles bearing alkyl groups of varying chain lengths and branching patterns (**49**–**54**) other than benzyls. Alkyl chains attached with varied functional groups, including hydropyran (**55**), ester (**56**), chloride (**57**), and nitrile (**58**), all proceeded through desymmetrization smoothly and offered a handle for structural modification later. Additionally, structurally diverse olefins (**59**–**62**) remained intact during the nucleophilic addition, thus bringing along their rich reactivities to the ketonitrile products.

In addition to aryl and alkyl motifs, disubstituted malononitriles bearing an alkenyl group prepared via a sequence of ketone condensation and migratory alkylation are also suitable desymmetrization substrates, affording diverse allylic quaternary stereocenters (**63**–**65**). However, the current rhodium-phosphoramidite catalyst imposes inferior stereocontrol on dialkyl-substituted malononitriles. While the enantioselectivity for the benzyl/methyl-substituted malononitrile (**66**) was negligible, a slight improvement was observed when the small methyl group was replaced by an isobutyl group (**67**), highlighting the importance of marked steric disparity between the pair of alkyl substituents. Nevertheless, attempts to employ disubstituted malononitriles bearing a hindered secondary or tertiary alkyl group (e.g., isopropyl or *tert*-butyl) were unsuccessful, as reactivity was lost, presumably due to the limited capacity of the chiral pocket to accommodate such bulky substituents.

The catalyst also shows potential for constructing cyclic quaternary stereocenters despite only moderate enantioselectivity at this stage. Enantioenriched carbocycles can be accessed either through an intermolecular addition to a malononitrile bearing two tethered substituents (**68**) or via an intramolecular addition of pinacol boronate embedded on a benzyl substituent (**69**).

The desymmetric addition adds greatly to the complexity of the quaternary carbon by introducing an oxygen-containing ketone together with its diverse reactivities (Figure 3A). Common addition (**70**), condensation (**71**), and reduction (**72**) conditions can be readily applied to the polyfunctionalized and congested ketonitrile products without a loss of enantiopurity. A stereospecific Baeyer–Villiger oxidation proceeded via a selective migration of the tertiary carbon, despite the electron-withdrawing nitrile substituent, to give enantioenriched benzoyl-protected alcohol **73**. Conversion of the remaining nitrile from desymmetrization to other nitrogen-containing motifs is also feasible, as exemplified by a mild hydrolysis to β-ketoamide **74**.^17^ In addition, when the product of ketone reduction (**72**) was further reduced under harsher conditions, polysubstituted 1,3-amino alcohol **75** was obtained in both good diastereo- and enantioselectivity.

**Figure 3:**
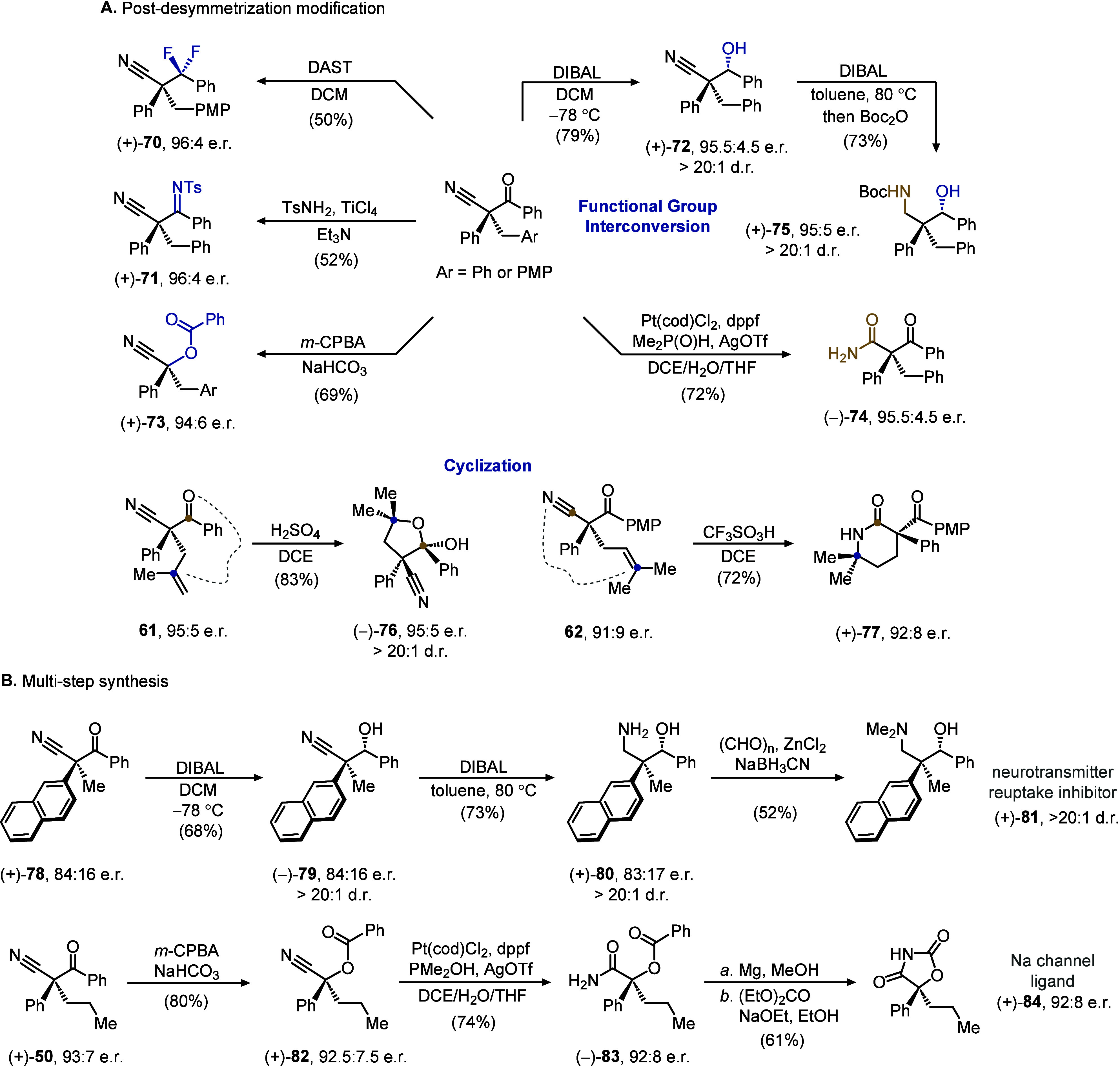
(A) Derivatization of desymmetrization products. (B) Multistep synthesis of bioactive molecules using desymmetrization.

Beyond functional group interconversion, coupling the nitrile or ketone with the two substituents from malononitrile can substantially increase the molecular complexity. When the methallyl-substituted product **61** was treated with simple acidic conditions, the protonation of olefin initiated a hemiacetal formation (**76**) with the ketone. Intriguingly, the chemoselectivity of the cyclization can be switched if an alternative acid is used on a slightly different ketonitrile product (**62**). In this case, an enantioenriched lactam (**77**) was generated from the nitrile and olefin.

These postdesymmetrization modifications are seamlessly applicable to the multistep synthesis of chiral bioactive molecules (Figure 3B). As an illustration, the DIBAL reduction sequence was carried out on a naphthyl-substituted ketonitrile (**78**) to afford amino alcohol **80**. Subsequent selective methylation of the amine furnished **81** as a neurotransmitter reuptake inhibitor.^18^ In another case, the peracid oxidation of the phenylpropyl-substituted product, followed by nitrile hydrolysis, generated α-hydroxyamide **83**. After the benzoyl deprotection, the revealed alcohol and the amide cyclized with diethyl carbonate to give an oxazolidinedione-based sodium channel ligand (**84**) in its enantioenriched form.^19^

In summary, a phosphoramidite ligand composed of a vaulted binaphthol scaffold and an iminostilbene substituent is key to enabling a rhodium-catalyzed and intermolecular desymmetric addition to malononitriles. The intricate chiral pocket of the catalyst is proposed to restrict σ-bond rotation in the coordinated malononitrile, thus allowing effective discrimination between its enantiotopic nitrile motifs. The combination of the remaining nitrile and the newly formed ketone, together with the broad substituent compatibility of the malononitrile reactants, renders the resulting acyclic quaternary stereocenters versatile building blocks for the synthesis of polyfunctionalized molecules.

## Supplementary Material



## Notes

Supplementary figures, experimental procedures, NMR spectra for all new compounds, HPLC traces for chiral compounds, crystallographic data (PDF)
