# Supplementary material for: Rhodium-Catalyzed Desymmetric Addition of Boronic Acids to Malononitriles
Source: J Am Chem Soc. 2026 Apr 12;148(15):15341–8. doi: 10.1021/jacs.5c23045 (PMC13107447; doi:10.1021/jacs.5c23045)
Supplement: Supplementary file 1 [file ja5c23045_si_001.pdf]

Supporting Information for

Rhodium-Catalyzed Desymmetric Addition of Boronic Acids to Malononitriles

Minghao Zhang,[1] Qihao Zhang,[1] Junjie Cao,[2] Jun (Joelle) Wang,[3] Jianchun Wang,[2,*] Zhongxing Huang[1,*]

[1]State Key Laboratory of Synthetic Chemistry, Shanghai Hong Kong Joint Laboratory in Chemical Synthesis, Department of Chemistry, The University of Hong Kong, Hong Kong 000000, China.

[2]Shenzhen Grubbs Institute and Department of Chemistry, Guangming Advanced Research Institute, and Guangdong Provincial Key Laboratory of Catalysis, Southern University of Science and Technology, Shenzhen 518055, China;

[3]Department of Chemistry, Hong Kong Baptist University, Kowloon, Hong Kong 000000, China

Table of Contents

Section		Page
1	Materials and methods	S3 [link]
2	Optimization of the desymmetric addition	S4 [link]
3	Preparation and characterization of ligands	S8 [link]
4	Preparation and characterization of substrates	S14 [link]
5	General procedures for desymmetric addition	S25 [link]
6	Characterization data of products	S25 [link]
7	Synthetic applications	S101 [link]
8	Mechanistic studies	S122 [link]
9	Crystal structures and crystallographic data	S177 [link]
10	Copies of spectra	S181 [link]

Section 1. Materials and method

Unless otherwise noted, all reactions were run under nitrogen atmosphere. Rhodium catalysts and boronic acids were purchased from Energy Chemical Inc., Leyan Pharmaceutical, or Bidepharm and used as received. K_2CO_3 was purchased from Bidepharm. and used as received. Et_2O is dried using a solvent drying system and other dry solvents were purchased from Energy Chemicals Inc. and used as received. Thin layer chromatography (TLC) was run on silica gel plates purchased from Yantai Huanghai Silica gel Development Co., Ltd.

^{1}H NMR, ^{13}C NMR, and ^{19}F NMR spectra were obtained on a Bruker 400, 500 or 600 spectrometer (400/500/600 MHz for ^{1}H, 100/126/151 MHz for ^{13}C, 376/471/565 MHz for ^{19}F, 202/243 MHz for ^{31}P). All ^{1}H NMR experiments were measured with tetramethylsilane (0 ppm) in $CDCl_3$, the signal of residual DMSO (2.50 ppm) in DMSO-d_6 as the internal reference; ^{13}C NMR experiments were measured in relative to the signal of $CDCl_3$ (77.0 ppm), the signal of DMSO-d_6 (39.52 ppm). Data for ^{1}H NMR, ^{13}C NMR, ^{19}F NMR and ^{31}P NMR were presented as following: chemical shifts (δ, ppm), multiplicity (br = broad, s = singlet, d = doublet, t = triplet, q = quartet, hept = heptet, dd = doublet of doublets, tt = triplet of triplets, td = triplet of doublets, m = multiplet), coupling constant (Hz), and integration. Chiral HPLC traces were measured on an Agilent 1260 Infinity II. The $[\alpha]_D$ were recorded using INESA SGW-531 Automatic Polarimeter. High-resolution EI mass spectra were recorded on a Thermo Scientific DFS high resolution magnetic sector MS. High-resolution ESI-MS measurements were performed on a Bruker impact II high-resolution LC-QTOF mass spectrometer.

Section 2. Optimization of the desymmetric addition

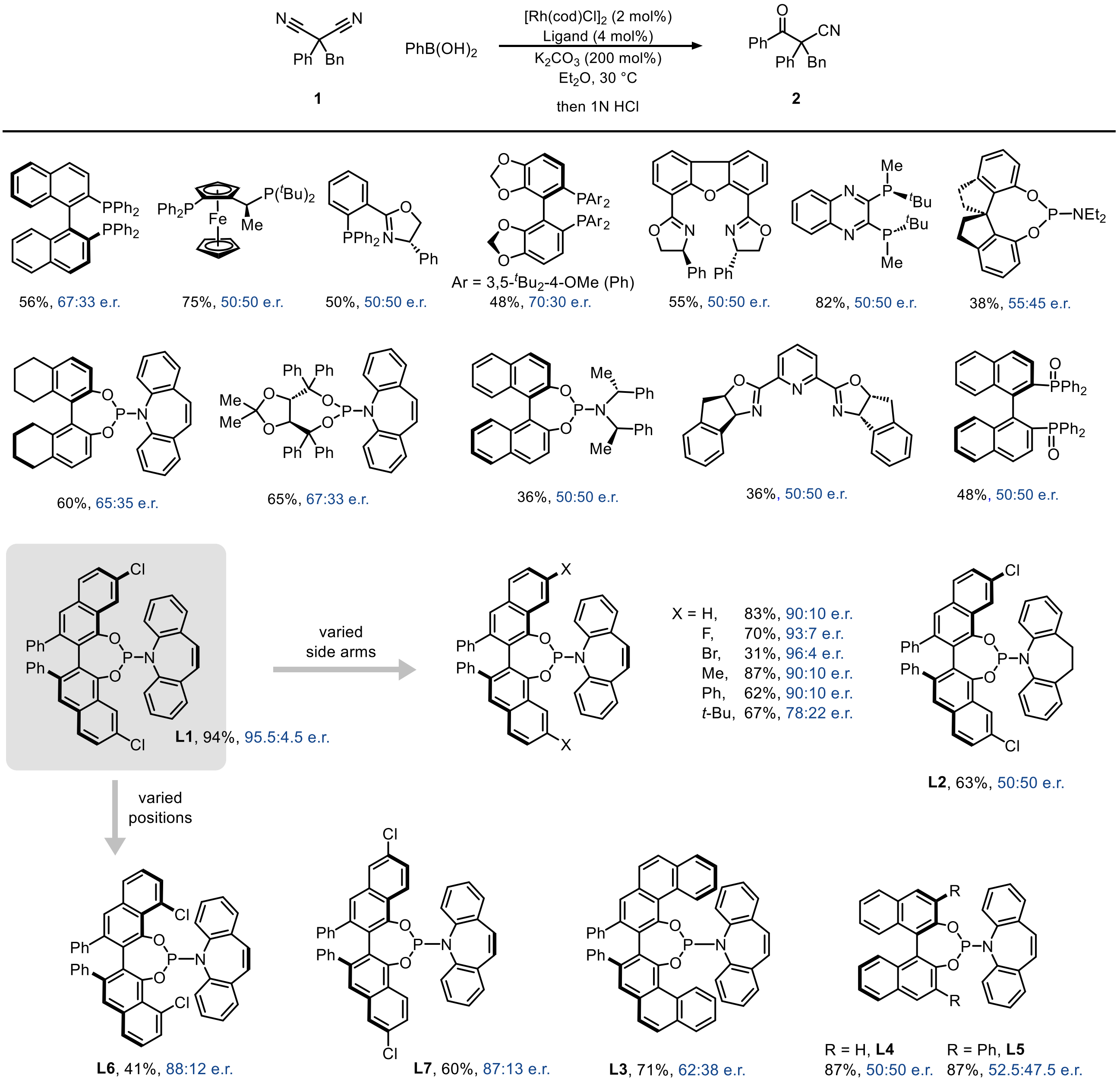

Reaction conditions unless noted otherwise: **1** (0.1 mmol), phenylboronic acid (0.2 mmol), ligand (0.004 mmol), $[Rh(cod)Cl]_2$ (0.002 mmol), K_2CO_3 (0.2 mmol) with diethyl ether (2.0 mL) at 30 °C for 24 hours. The yield was determined by crude ^1H NMR using 1,3,5-trimethoxybenzene as the internal standard. The e.r. was determined by chiral HPLC.

Supplementary Figure S1. Screening of chiral ligands.

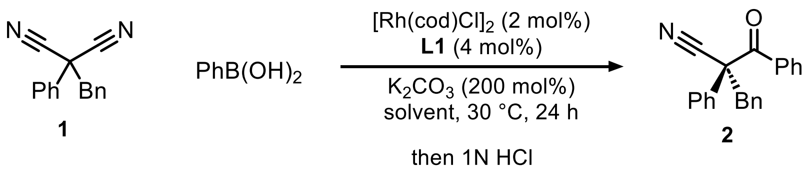

Entry	Solvent	Yield (%)	e.r.
1	dioxane	66	95:5
2	DME	59	95:5
3	2-Me-THF	46	94.5:5.5
4	CyH	79	92.5:7.5
5	toluene	50	95.5:4.5
6	CPME	34	92.5:7.5
7	MTBE	52	95:5
8	EA	72	95.5:4.5
9	Et_2O	94	95.5:4.5
10	$^iPr_2O$	40	94.5:5.5

Reaction conditions unless noted otherwise: **1** (0.1 mmol), phenylboronic acid (0.2 mmol), **L1** (0.004 mmol), [Rh(cod)Cl]$_2$ (0.002 mmol), K_2CO_3 (0.2 mmol) with examined solvent (2.0 mL) at 30 °C for 24 hours. The yield was determined by crude ^{1}H NMR using 1,3,5-trimethoxybenzene as the internal standard. The e.r. was determined by chiral HPLC.

Supplementary Figure S2. Screening of solvents.

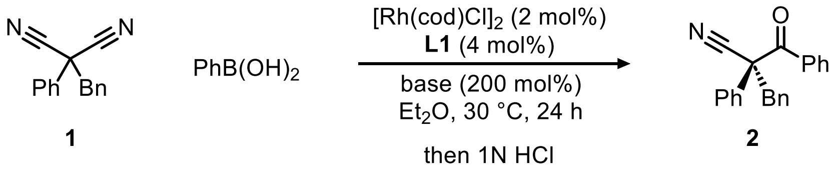

Entry	Base	Yield (%)	e.r.
1	KF	62	95:5
2	Na_2CO_3	trace	/
3	Cs_2CO_3	43	95.5:4.5
4	Ag_2CO_3	80	95.5:4.5
5	Li_2CO_3	trace	/
6	Li_3PO_4	trace	/
7	K_3PO_4	84	95:5
8	KOAc	51	95:5
9	NEt_3	53	95.5:4.5
10	Proton Sponge	42	94.5:5.5
11	w/o base	trace	/

Reaction conditions unless noted otherwise: **1** (0.1 mmol), phenylboronic acid (0.2 mmol), **L1** (0.004 mmol), $[Rh(cod)Cl]_2$ (0.002 mmol), examined base (0.2 mmol) in Et_2O (2.0 mL) at 30 °C for 24 hours. The yield was determined by crude ^1H NMR using 1,3,5-trimethoxybenzene as the internal standard. The e.r. was determined by chiral HPLC.

Supplementary Figure S3. Screening of bases.

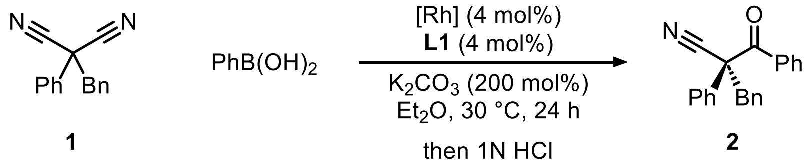

Entry	[Rh]	Yield (%)	e.r.
1	$[Rh(cod)OH]_2$	88	95:5
2	$[Rh(cod)OMe]_2$	63	95.5:4.5
3	$[Rh(cod)_2]OTf$	trace	/
4	$Rh_2(OAc)_4$	n.d.	/
5	$[Cp^*RhCl_2]_2$	trace	/
6	$Rh(cod)_2BF_4$	69	95:5
7	$Rh(NBE)_2BF_4$	n.d.	/
8	$Rh(C_2H_4)_2(acac)$	36	95.5:4.5
9	$[Rh(C_2H_4)_2Cl]_2$	25	95.5:4.5

Reaction conditions unless noted otherwise: **1** (0.1 mmol), phenylboronic acid (0.2 mmol), **L1** (0.004 mmol), [Rh] (0.004 mmol, in Rh), K_2CO_3 (0.2 mmol) in Et_2O (2.0 mL) at 30 °C for 24 hours. The yield was determined by crude ^1H NMR using 1,3,5-trimethoxybenzene as the internal standard. The e.r. was determined by chiral HPLC.

Supplementary Figure S4. Screening of rhodium catalysts.

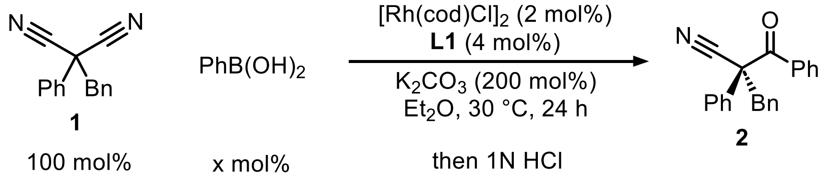

Entry	x (mol%)	Yield (%)	e.r.
1	100	54	95.5:4.5
2	150	87	95.5:4.5
3	200	94	95.5:4.5
4	250	95	95.5:4.5

Reaction conditions unless noted otherwise: **1** (0.1 mmol), phenylboronic acid (x mol%), **L1** (0.004 mmol), $[Rh(cod)Cl]_2$ (0.002 mmol), K_2CO_3 (0.2 mmol) in Et_2O (2.0 mL) at 30 °C for 24 hours. The yield was determined by crude ^1H NMR using 1,3,5-trimethoxybenzene as the internal standard. The e.r. was determined by chiral HPLC.

Supplementary Figure S5. Screening of equivalent of phenylboronic acid.

Section 3. Preparation and characterization of ligands

Representative procedures for ligand synthesis

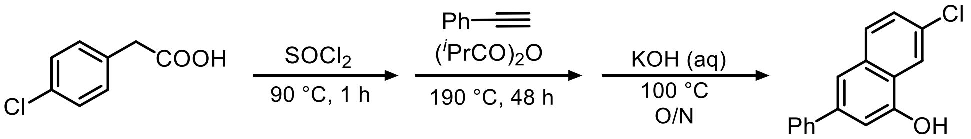

Following the literature procedure[1], a single-neck 500 mL round bottom flask equipped with a condenser were charged with 4-chlorophenylacetic acid **I** (3.41 g, 20 mmol) and $SOCl_2$ (5.22 mL, 72 mmol). The top of the condenser was vented to a bubbler and then into a beaker filled with NaOH (sat. aq.) to trap acidic gases (HCl and SO_2). The mixture was heated to reflux for 1 h, then cooled to room temperature. All of the volatiles were carefully removed under vacuo. To the flask containing the acyl chloride was added phenylacetylene (2.85 mL, 26 mmol) and $(^iPrCO)_2O$ (6.62 mL, 40 mmol) under N_2. The mixture was stirred at 190 °C for 48 h with two reflux condensers connected in series. The brown reaction mixture was cooled to below 100 °C and a solution of KOH (6.72 g, 120 mmol) in H_2O (35 mL) was then added slowly. This two-phase mixture was stirred at 100 °C overnight. The mixture was cooled to room temperature and ethyl acetate (200 mL) was added and the mixture stirred for 10 min before the organic layer was separated. The aqueous layer was extracted twice with ethyl acetate (100 mL × 3) and the combined organic layer was washed with brine (100 mL), dried, filtered, and concentrated. The residue was submitted to flash column chromatography (hexane/ethyl acetate) to afford the product **II** (2.89 g, 57%).

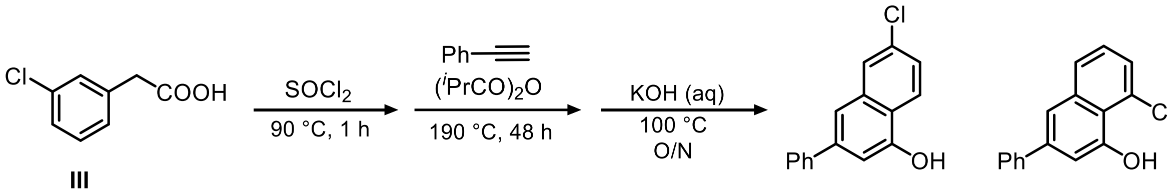

A single-neck 500 mL round bottom flask equipped with a condenser were charged with 3-chlorophenylacetic acid **III** (6.80 g, 40 mmol) and $SOCl_2$ (10.4 mL, 144 mmol). The top of the condenser was vented to a bubbler and then into a beaker filled with NaOH (sat. aq.) to trap acidic gases (HCl and SO_2). The mixture was heated to reflux for 1 h, then cooled to room temperature. All of the volatiles were carefully removed under vacuo. To the flask containing the acyl chloride was added phenylacetylene (5.7 mL, 52 mmol) and $(^iPrCO)_2O$ (13.2 mL, 80 mmol) under N_2. The mixture was stirred at 190 °C for 48 h with two reflux condensers connected in series. The brown reaction mixture was cooled to below 100 °C and a solution of KOH (13.44 g, 240 mmol) in H_2O (70 mL) was then added slowly. This two-phase mixture was stirred at 100 °C overnight. The mixture was cooled to room temperature and ethyl acetate (200 mL) was added and the mixture stirred for 10 min before the organic layer was separated. The aqueous layer was extracted twice with ethyl acetate (200 mL × 3) and the combined organic layer was washed with brine (200 mL), dried, filtered, and concentrated. The residue was submitted to flash column chromatography (hexane/ethyl acetate) to afford the product **IV** (3.20 g, 32%) and **V** (900 mg, 9%).

[1] Guan, Y., Ding, Z., Wulff, W. D. Vaulted biaryls in catalysis: A structure-activity relationship guided tour of the immanent domain of the VANOL ligand. *Chem. Eur. J.* **2013**, *19*, 15565–15571.

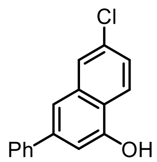

R_f = 0.5 (Hexane/EtOAc = 5:1).
^{1}H NMR (400 MHz, $CDCl_3$) δ 8.16 (s, 1H), 7.77 (d, *J* = 8.1 Hz, 1H), 7.69 (d, *J* = 7.7 Hz, 2H), 7.61 (s, 1H), 7.47 (t, *J* = 7.4 Hz, 2H), 7.42 – 7.35 (m, 2H), 7.35 – 7.26 (m, 2H).

^{13}C NMR (101 MHz, $CDCl_3$) δ 153.1, 140.3, 139.9, 137.3, 128.9, 128.9, 127.8, 127.4, 127.2, 125.9, 118.9, 118.7, 112.5.

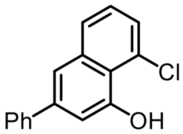

R_f = 0.8 (Hexane/EtOAc = 5:1).

^{1}H NMR (400 MHz, $CDCl_3$) δ 8.12 (d, *J* = 8.9 Hz, 1H), 7.81 (s, 1H), 7.63 (d, *J* = 7.7 Hz, 2H), 7.52 (s, 1H), 7.46 (t, *J* = 7.3 Hz, 2H), 7.43 – 7.35 (m, 2H), 5.48 (s, 1H).

^{13}C NMR (101 MHz, $CDCl_3$) δ 151.8, 140.5, 140.3, 135.7, 132.8, 128.9, 127.7, 127.3, 126.5, 126.0, 123.5, 121.8, 117.8, 108.6.

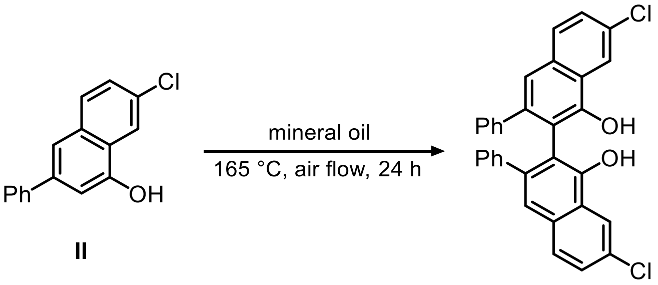

II **VI**

To a 500 mL flame-dried three neck round bottom flask equipped with a cooling condenser were added compound **II** (2.79 g, 11 mmol) and mineral oil (55 mL). Airflow was introduced from one side neck via a needle located one inch above the mixture. The airflow rate is about one bubble per second. The mixture was stirred at 165 °C for 24 h. After cooling down to room temperature, DCM (50 mL) and hexanes (100 mL) were added to the flask and the mixture was stirred until all large chunks had been broken up. The suspension was cooled in a freezer (–20 °C) and then filtered through filter paper. The powder was washed with cooled DCM/hexanes and dried under vacuum to afford a yellow solid (0.83 g). Purification of the product remaining in the mother liquor by column chromatography afforded the product **VI** (1.10 g). The total yield is 69% (1.93 g).

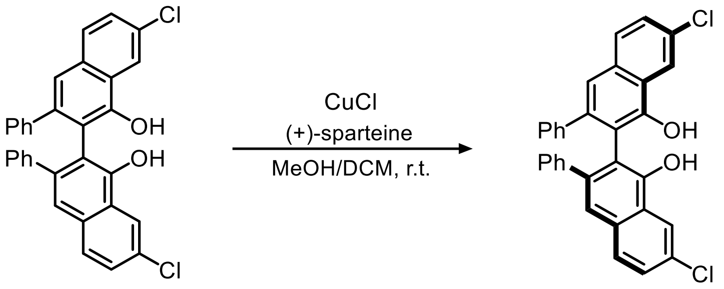

VI (*R*)-**VII**

To a 250 mL round bottom flask were added (+)-sparteine (3.1172 g, 13.3 mmol), CuCl (643.5 mg, 6.5 mmol), and MeOH (110 mL) under air. The reaction mixture was sonicated in a water bath for 60 min with exposure to air. The flask was then sealed with a septum and purged with N_2, which was introduced by a needle under the surface for 60 minutes. At the same time, to a 1 L flame-dried round bottom flask were added racemic compound **VI** (1.9231 g, 3.8 mmol) and DCM (380 mL). The resulting solution was purged with argon for 60 minutes under the surface. The green Cu-sparteine solution was then transferred via cannula to the solution of racemic compound **VI** under N_2 and then the combined mixture was sonicated for 15 minutes. The reaction mixture was stirred at room temperature overnight under nitrogen atmosphere with the flask wrapped in aluminum foil to protect it from light. The reaction was quenched by slow addition of saturated $NaHCO_3$ aqueous solution, H_2O (100 mL) and most of the organic solvent was removed under reduced pressure. The residue was then extracted with DCM (100 mL × 3). The combined organic layer was dried over Na_2SO_4, filtered through Celite and concentrated to dryness. Purification of the crude product by column chromatography on silica gel gave the product (*R*)-**VII** as an off-white foamy solid (901.1 mg, 47%). The optical purity was determined to be >99% e.e. by HPLC analysis (Chiralpak ID-3, hexane/^{i}PrOH = 90/10, 1.0 mL/min, 254 nm; tr (minor) = 13.9 min, tr (major) = 16.2 min), $[\alpha]_D^{17}$ = +218.0 (*c* = 1.0, $CHCl_3$).

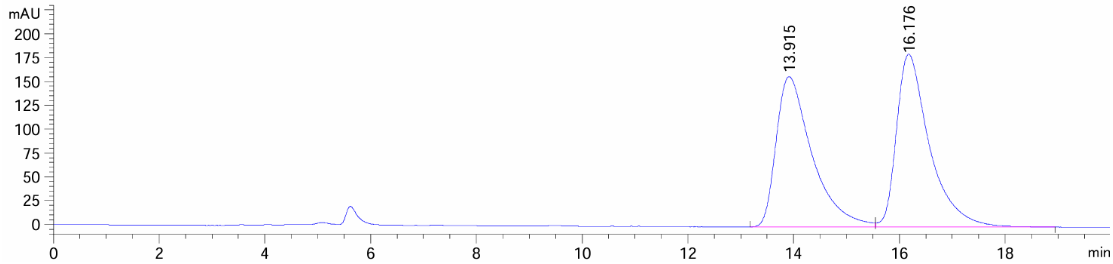

Peak #	RetTime [min]	Type	Width [min]	Area [mAU*s]	Height [mAU]	Area %
1	13.915	BV	0.6980	7459.19189	157.64703	48.6139
2	16.176	VB	0.6454	7884.53711	181.27621	51.3861

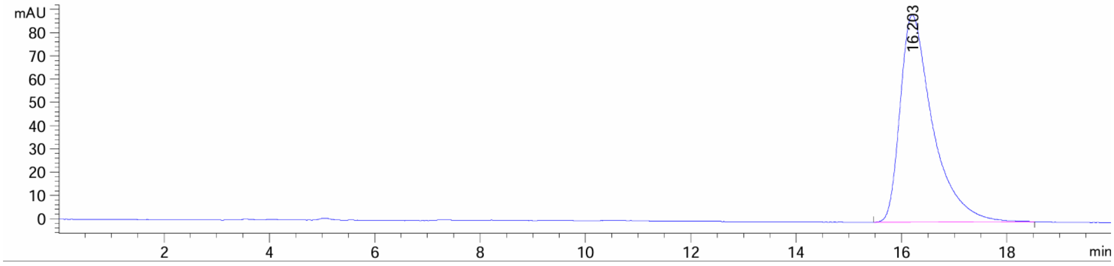

Peak #	RetTime [min]	Type	Width [min]	Area [mAU*s]	Height [mAU]	Area %
1	16.203	BB	0.6254	3812.74561	89.06396	100.0000

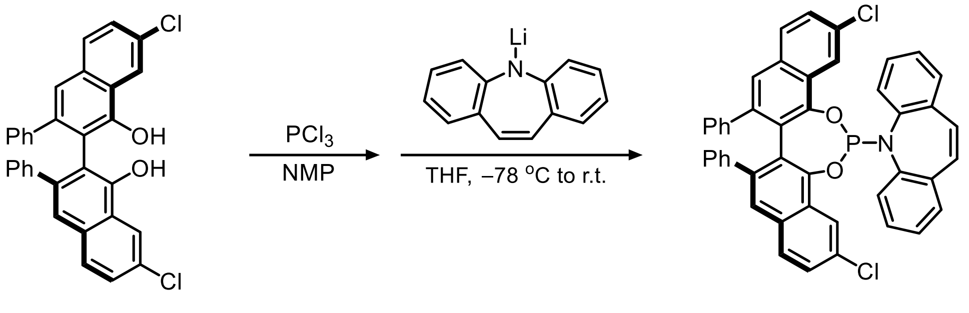

(*R*)-**VII** **L1**

Following the literature procedure[2], a Schlenk flask under argon was charged with (*R*)-**VII** (860.3 mg, 1.7 mmol). PCl_3 (2.2 mL, 25.5 mmol) and a catalytic amount of *N*-methylpyrrolidone (49 µL, 0.51 mmol) were added and the reaction mixture was heated at 50 °C for 30 min. The initially heterogeneous mixture turned into a brownish homogenous solution. After cooling to room temperature, the excess PCl_3 was evaporated in vacuo, and 1 mL toluene was added to azeotropically remove remaining PCl_3. The resulting phosphorchloridite was redissolved in 2 mL THF. In a separate flask under N_2, the iminostilbene (361.1 mg, 1.87 mmol) was dissolved in 3 mL THF, followed by the slow addition of *n*-BuLi (0.75 mL, 2.5 M solution in hexanes) at –78 °C. The resulting deep blue solution was continued to stir at –78 °C for 1 h before the phosphorchloridite solution was slowly transferred via cannula. The resulting mixture was stirred at –78 °C, then warmed to room temperature and continued to stir overnight. The solvents were then evaporated in vacuo and the residue was purified by flash column chromatography to afford ligand **L1** (717.4 mg, 58% Yield). R_f = 0.7 (Hexane/EtOAc = 10:1). $[\alpha]_D^{20}$ = –240.30 (*c* = 1.0, $CHCl_3$).

^{1}H NMR (500 MHz, CDCl3) δ 8.50 (s, 1H), 8.13 (s, 1H), 7.69 (d, *J* = 8.6 Hz, 1H), 7.51 – 7.42 (m, 2H), 7.41 – 7.29 (m, 5H), 7.24 (d, *J* = 8.7 Hz, 2H), 7.10 – 6.99 (m, 3H), 6.98 – 6.77 (m, 6H), 6.59 (d, *J* = 7.5 Hz, 1H), 6.52 – 6.37 (m, 4H), 6.32 (d, *J* = 7.6 Hz, 2H).

^{13}C NMR (126 MHz, CDCl3) δ 147.5, 147.4, 146.0, 142.5, 142.3, 141.3, 140.8, 140.5, 140.1, 139.7, 136.51, 136.48, 134.7, 132.5, 132.34, 132.29, 131.8, 131.1, 130.4, 129.6, 129.3, 129.2, 129.1, 129.0, 128.5, 128.2, 128.1, 127.92, 127.86, 127.6, 127.4, 127.3, 127.3, 127.0, 126.8, 126.48, 126.45, 126.00, 125.96, 125.7, 125.6, 124.1, 123.4, 123.0, 122.9, 121.5.

^{31}P NMR (202 MHz, CDCl3) δ 142.65.

HRMS (ESI) calcd $C_{46}H_{29}Cl_2NO_2P^+$ $[M+H]^+$: 728.1307. Found: 728.1304.

---

[2] Lafrance, M., Roggen, M., Carreira, E. M. Direct, enantioselective iridium-catalyzed allylic amination of racemic allylic alcohols. *Angew. Chem. Int. Ed.* **2012**, *51*, 3470–3473.

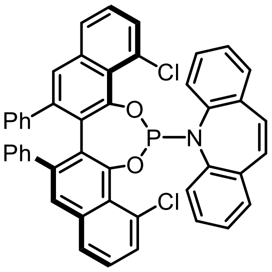

L6

Ligand **L6** was synthesized following the procedure described above for ligand **L1** as a white solid (0.2 mmol scale, 39.5 mg, 27% yield). R_f = 0.7 (hexane/ethyl acetate = 10:1). $[\alpha]_D^{20}$ = –83.85 (*c* = 1.0, $CHCl_3$).

^{1}H NMR (600 MHz, $CDCl_3$) δ 7.67 (d, *J* = 7.8 Hz, 2H), 7.58 (d, *J* = 7.4 Hz, 1H), 7.55 (d, *J* = 8.2 Hz, 2H), 7.39 (td, *J* = 7.8, 3.4 Hz, 2H), 7.33 (s, 1H), 7.22 – 7.17 (m, 2H), 7.17 – 7.11 (m, 2H), 7.07 (t, *J* = 7.4 Hz, 1H), 7.04 – 6.99 (m, 2H), 6.92 (t, *J* = 7.6 Hz, 2H), 6.83 (q, *J* = 6.8, 6.2 Hz, 3H), 6.75 (d, *J* = 11.5 Hz, 1H), 6.59 (t, *J* = 7.4 Hz, 1H), 6.48 (d, *J* = 7.5 Hz, 1H), 6.39 (d, *J* = 7.6 Hz, 2H), 6.30 (d, *J* = 7.6 Hz, 2H), 6.08 (d, *J* = 11.5 Hz, 1H).

^{13}C NMR (151 MHz, $CDCl_3$) δ 147.2, 147.2, 146.6, 142.6, 142.5, 141.1, 141.0, 140.8, 139.6, 139.4, 136.6, 136.5, 136.4, 136.3, 134.7, 131.2, 130.9, 130.8, 129.6, 129.4, 129.3, 129.3, 129.2, 129.0, 128.9, 128.5, 128.5, 128.0, 127.8, 127.8, 127.4, 127.3, 127.0, 126.5, 126.4, 126.4, 126.1, 125.7, 125.5, 125.5, 124.9, 123.6, 123.3, 123.3.

^{31}P NMR (202 MHz, $CDCl_3$) δ 140.14.

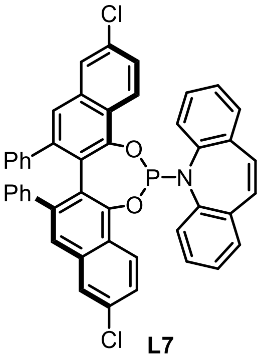

L7

Ligand **L7** was synthesized following the procedure described above for ligand **L1** as a white solid (0.2 mmol scale, 20.6 mg, 14% yield). R_f = 0.7 (hexane/ethyl acetate = 10:1). $[\alpha]_D^{20}$ = –157.27 (*c* = 1.0, $CHCl_3$).

^{1}H NMR (600 MHz, $CDCl_3$) δ 8.44 (d, *J* = 8.8 Hz, 1H), 8.22 (d, *J* = 8.8 Hz, 1H), 7.79 (s, 1H), 7.63 (dd, *J* = 8.8, 1.8 Hz, 1H), 7.58 – 7.54 (m, 1H), 7.48 – 7.41 (m, 2H), 7.32 – 7.26 (m, 3H), 7.22 (t, *J* = 7.3 Hz, 1H), 7.12 – 7.01 (m, 4H), 6.96 – 6.91 (m, 3H), 6.86 (t, *J* = 7.4 Hz, 2H), 6.72 (d, *J* = 11.5 Hz, 1H), 6.64 – 6.59 (m, 2H), 6.58 – 6.54 (m, 1H), 6.44 (d, *J* = 7.4 Hz, 2H), 6.32 (d, *J* = 7.4 Hz, 2H).

^{13}C NMR (151 MHz, CDCl$_3$) δ 148.1, 148.1, 146.9, 142.5, 142.3, 141.8, 141.6, 140.0, 139.7, 136.3, 136.3, 134.8, 134.7, 134.3, 133.0, 132.6, 132.6, 130.6, 129.2, 129.2, 129.1, 129.0, 128.6, 128.4, 127.8, 127.8, 127.6, 127.5, 127.5, 126.8, 126.6, 126.6, 126.1, 126.1, 125.9, 125.5, 125.1, 125.1, 124.9, 124.5, 124.1, 123.4, 122.5, 122.4, 77.2.

^{31}P NMR (243 MHz, CDCl$_3$) δ 142.86.

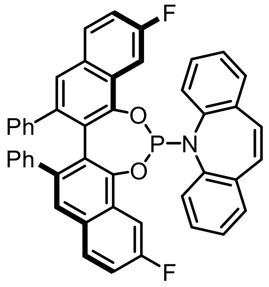

L8

Ligand **L8** was synthesized following the procedure described above for ligand **L1** as a white solid (0.3 mmol scale, 94.0 mg, 45% yield). R_f = 0.7 (hexane/ethyl acetate = 10:1). $[\alpha]_D^{20}$ = –144.03 (*c* = 1.0, CHCl$_3$).

^{1}H NMR (600 MHz, CDCl$_3$) δ 8.14 – 8.08 (m, 1H), 7.85 – 7.77 (m, 2H), 7.57 – 7.50 (m, 1H), 7.42 (d, *J* = 7.9 Hz, 1H), 7.38 – 7.32 (m, 3H), 7.28 (d, *J* = 6.5 Hz, 1H), 7.26 – 7.19 (m, 3H), 7.12 – 7.06 (m, 2H), 7.04 (t, *J* = 7.3 Hz, 1H), 7.00 – 6.90 (m, 3H), 6.86 (t, *J* = 7.5 Hz, 2H), 6.80 (d, *J* = 11.5 Hz, 1H), 6.60 (d, *J* = 7.5 Hz, 1H), 6.54 (t, *J* = 7.1 Hz, 1H), 6.47 (t, *J* = 6.1 Hz, 3H), 6.33 (d, *J* = 7.3 Hz, 2H).

^{13}C NMR (151 MHz, CDCl$_3$) δ 161.1 (d, *J* = 246.1 Hz), 160.3 (d, *J* = 246.1 Hz), 147.6 (d, *J* = 6.0 Hz), 147.5 (d, *J* = 9.0 Hz), 146.40 (d, *J* = 6.0 Hz), 146.38 (d, *J* = 6.0 Hz), 146.36, 142.6, 142.4, 141.6, 140.3, 139.9, 139.84, 139.82, 139.5, 136.4 (d, *J* = 4.5 Hz), 134.8, 132.4, 131.2, 130.59 (d, *J* = 22.7 Hz), 130.58, 129.4 (d, *J* = 9.1 Hz), 129.3, 129.2, 129.1 (d, *J* = 6.0 Hz), 128.2, 127.9 (d, *J* = 10.6 Hz), 127.54, 127.50, 127.4, 127.1 (d, *J* = 9.1 Hz), 126.9, 126.7, 126.3 (d, *J* = 4.5 Hz), 126.0 (d, *J* = 4.5 Hz), 125.7, 124.2, 123.1 (d, *J* = 3.0 Hz), 117.4 (d, *J* = 25.7 Hz), 116.7 (d, *J* = 25.7 Hz), 108.4 (d, *J* = 22.7 Hz), 106.1 (d, *J* = 22.7 Hz).

^{31}P NMR (243 MHz, CDCl$_3$) δ 142.57.

^{19}F NMR (565 MHz, CDCl$_3$) δ -112.63 (m), -114.09 – -114.53 (m).

HRMS (ESI) calcd $C_{46}H_{29}F_2NO_2P^+$ $[M+H]^+$: 696.1898. Found: 696.1896.

Section 4. Preparation and characterization of substrates

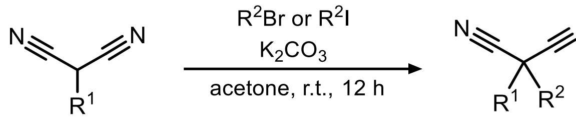

To an oven-dried 100 mL round bottom flask equipped with a stir bar were added mono-substituted malononitrile[3] (100 mol%, 0.2 M in acetone), K_2CO_3 (200 mol%), and corresponding alkyl bromide or alkyl iodide (150 mol%). Then the reaction mixture was stirred overnight at room temperature until complete consumption of mono-substituted malononitrile as monitored by TLC. The white precipitates were filtered and washed with ethyl acetate, then the filtrate was concentrated under reduced pressure and purified by flash column chromatography (hexane/ethyl acetate) to yield the desired disubstituted malononitrile.

[3](a) Huang, J., Gao, C., Tao, X., Qian, Y. A convenient and efficient palladium-catalyzed system for cross-coupling of aryl bromides with active methylene compounds. *Synlett*, **2003**, 1716–1718. (b) Weweler, J., Younas, S. L., Streuff, J. Titanium(III)-catalyzed reductive decyanation of geminal dinitriles by a non-free-radical mechanism. *Angew.Chem. Int.Ed.* **2019***, 58*, 17700–17703.

Previously reported malononitrile substrates

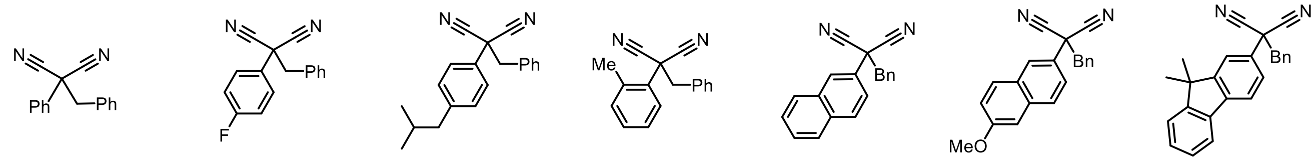

S2 **S19** **S20** **S22** **S24** **S25** **S26**

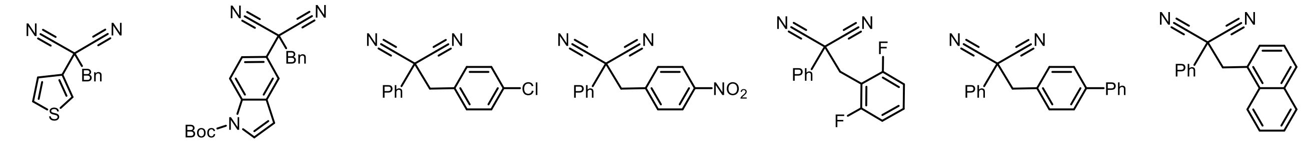

S27 **S28** **S33** **S34** **S36** **S38** **S39**

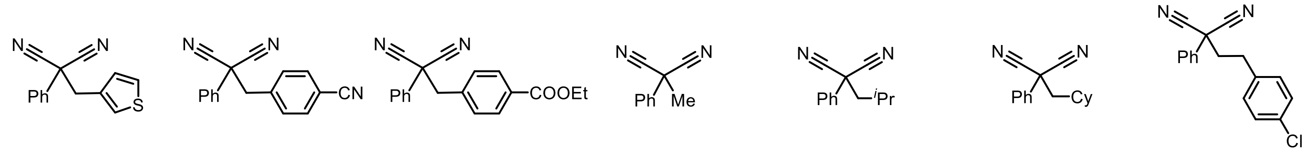

S40 **S44** **S48** **S49** **S51** **S52** **S53**

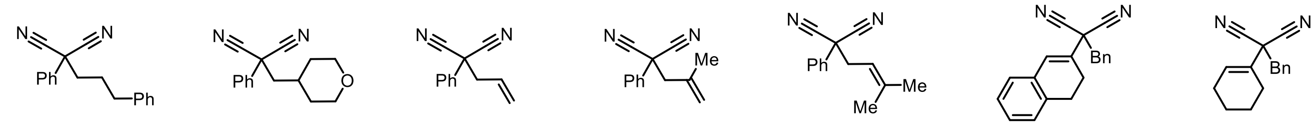

S54 **S55** **S59** **S61** **S62** **S63** **S64**

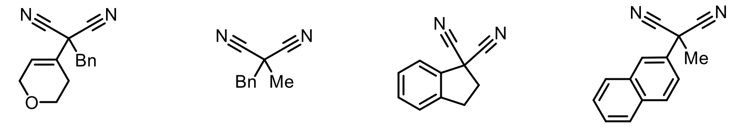

S65 **S66** **S68** **S78**

S2, **S19**, **S20**, **S22**, **S24**, **S25**, **S26**, **S27**, **S28**, **S33**, **S34**, **S36**, **S38**, **S39**, **S40**, **S44**, **S48**, **S49**, **S51**, **S52**, **S53**, **S54**, **S55**, **S59**, **S61**, **S62**, **S63**, **S64**, **S65**, **S66**, **S68**, and **S78** were known compounds and prepared using the reported methods.[4]

Previously unknown malononitrile substrates

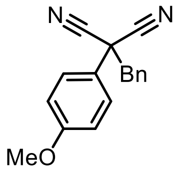

S18

S18 was synthesized using general procedures with corresponding benzyl bromide as a white solid

[4] (a) Mills, L. R., Edjoc, R. K., Rousseaux, S. A. L. Design of an electron-withdrawing benzonitrile ligand for Ni-catalyzed cross-coupling involving tertiary nucleophiles. *J. Am. Chem. Soc.* **2021**, *143*, 10422–10428. (b) Zeng, G., Liu, J., Shao, Y., Zhang, F., Chen, Z., Lv, N., Chen, J., Li, R. Selective synthesis of beta-ketonitriles via catalytic carbopalladation of dinitriles. *J. Org. Chem.* **2021**, *86*, 861–867. (c) Cao, B., Liu, G., Huang, Z. Alkene Cyclopropanation with gem-Dichloroalkanes Catalyzed by (PNN)Co Complexes: Scope and Mechanism. *ACS Catal.* **2024**, *14*, 12846-12856. (d) Zheng, Y., Yang, T., Chan, K. F., Lin, Z., Huang, Z. Cobalt-catalysed desymmetrization of malononitriles via enantioselective borohydride reduction. *Nat. Chem.* **2024**, *16*, 1845–1854.

(3.0 mmol scale, 628.3 mg, 80% yield). R_f = 0.6 (hexane/ethyl acetate = 5:1).

^{1}H NMR (400 MHz, $CDCl_3$) δ 7.39 – 7.27 (m, 5H), 7.16 – 7.10 (m, 2H), 6.97 – 6.89 (m, 2H), 3.82 (s, 3H), 3.42 (s, 2H).

^{13}C NMR (101 MHz, $CDCl_3$) δ 160.6, 131.5, 130.4, 128.7, 128.6, 127.4, 123.1, 114.8, 114.7, 55.4, 48.3, 43.3.

HRMS (ESI) calcd $C_{17}H_{15}N_2O^+$ $[M+H]^+$: 263.1179. Found: 263.1180.

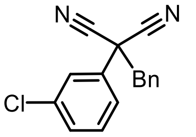

S21

S21 was synthesized using general procedures with corresponding benzyl bromide as a white solid (3.0 mmol scale, 622.0 mg, 78% yield). R_f = 0.8 (hexane/ethyl acetate = 5:1).

^{1}H NMR (400 MHz, $CDCl_3$) δ 7.54 – 7.26 (m, 7H), 7.14 (d, *J* = 7.1 Hz, 2H), 3.44 (s, 2H).

^{13}C NMR (101 MHz, $CDCl_3$) δ 135.6, 133.3, 131.0, 130.7, 130.34, 130.25, 129.0, 128.7, 126.4, 124.3, 114.1, 48.2, 43.6.

HRMS (ESI) calcd $C_{16}H_{12}ClN_2^+$ $[M+H]^+$: 267.0684. Found: 267.0689.

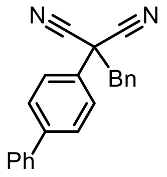

S23

S23 was synthesized using general procedures with corresponding benzyl bromide as a white solid (3.0 mmol scale, 388.2 mg, 42% yield). R_f = 0.4 (hexane/ethyl acetate = 10:1).

^{1}H NMR (400 MHz, $CDCl_3$) δ 7.67 (d, *J* = 7.7 Hz, 2H), 7.60 (d, *J* = 7.4 Hz, 2H), 7.56 – 7.44 (m, 4H), 7.44 – 7.29 (m, 4H), 7.19 (d, *J* = 7.1 Hz, 2H), 3.49 (s, 2H).

^{13}C NMR (101 MHz, $CDCl_3$) δ 143.0, 139.4, 131.5, 130.5, 130.3, 129.0, 128.9, 128.7, 128.2, 128.1, 127.1, 126.6, 114.7, 48.5, 43.8.

HRMS (ESI) calcd $C_{22}H_{17}N_2^+$ $[M+H]^+$: 309.1386. Found: 309.1387.

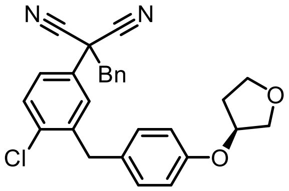

S29

S29 was synthesized using general procedures with corresponding benzyl bromide as a white solid (3.0 mmol scale, 785.2 mg, 59% yield). R_f = 0.5 (hexane/ethyl acetate = 3:1).

^{1}H NMR (400 MHz, CDCl$_3$) δ 7.44 (d, *J* = 8.4 Hz, 1H), 7.37 – 7.30 (m, 1H), 7.30 – 7.23 (m, 3H), 7.17 (d, *J* = 2.5 Hz, 1H), 7.10 – 7.03 (m, 2H), 7.00 (d, *J* = 8.6 Hz, 2H), 6.82 – 6.75 (m, 2H), 4.93 – 4.83 (m, 1H), 4.03 – 4.00 (m, 2H), 4.00 – 3.93 (m, 3H), 3.92 – 3.84 (m, 1H), 3.41 (s, 2H), 2.24 – 2.05 (m, 2H).

^{13}C NMR (101 MHz, CDCl$_3$) δ 156.0, 140.6, 136.1, 131.0, 130.6, 130.4, 130.3, 130.0, 129.8, 128.8, 128.6, 125.2, 115.5, 114.3, 77.3, 73.0, 67.1, 48.1, 43.3, 38.3, 32.9.

HRMS (ESI) calcd $C_{27}H_{24}ClN_2O_2^+$ [M+H]$^+$: 443.1521 Found: 443.1521.

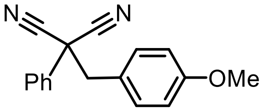

S30

S30 was synthesized using general procedures with corresponding alkyl bromide as a white solid (3.0 mmol scale, 581.0 mg, 74% yield). R_f = 0.5 (hexane/ethyl acetate = 5:1).

^{1}H NMR (400 MHz, CDCl$_3$) δ 7.47 – 7.43 (m, 5H), 7.08 – 7.00 (m, 2H), 6.86 – 6.78 (m, 2H), 3.78 (s, 3H), 3.40 (s, 2H).

^{13}C NMR (101 MHz, CDCl$_3$) δ 159.9, 131.6, 131.5, 129.8, 129.4, 126.1, 123.4, 114.8, 114.0, 55.2, 47.8, 44.2.

HRMS (ESI) calcd $C_{17}H_{15}N_2O^+$ [M+H]$^+$: 263.1179. Found: 263.1179.

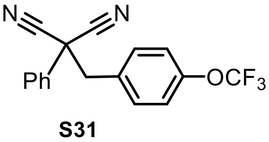

S31

S31 was synthesized using general procedures with corresponding alkyl bromide as a white solid (3.0 mmol scale, 842.2 mg, 89% yield). R_f = 0.5 (hexane/ethyl acetate = 5:1).

^{1}H NMR (400 MHz, CDCl$_3$) δ 7.49 – 7.45 (m, 5H), 7.18 – 7.14 (m, 4H), 3.46 (s, 2H).

^{13}C NMR (101 MHz, CDCl$_3$) δ 149.6 (q, *J* = 2.0 Hz), 131.9, 131.1, 130.2, 130.0, 129.6, 126.0, 120.9,

120.3 (q, J = 257.6 Hz), 114.4, 47.6, 43.8.

^{19}F NMR (376 MHz, CDCl$_3$) δ -57.81.

HRMS (ESI) calcd $C_{17}H_{11}F_3N_2NaO^+$ [M+Na]$^+$: 339.0716. Found: 339.0726.

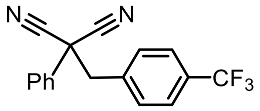

S32

S32 was synthesized using general procedures with corresponding alkyl bromide as a white solid (3.0 mmol scale, 840.0 mg, 93% yield). R$_f$ = 0.5 (hexane/ethyl acetate = 10:1).

^{1}H NMR (500 MHz, CDCl$_3$) δ 7.58 (d, J = 8.0 Hz, 2H), 7.50 – 7.47 (m, 5H), 7.27 (d, J = 7.9 Hz, 2H), 3.52 (s, 2H).

^{13}C NMR (126 MHz, CDCl$_3$) δ 135.3, 131.12 (q, J = 34.0 Hz), 131.1, 130.9, 130.3, 129.7, 126.1, 125.6 (q, J = 3.8 Hz), 123.8 (q, J = 272.2 Hz), 114.3, 48.0, 43.6.

^{19}F NMR (471 MHz, CDCl$_3$) δ -62.78.

HRMS (EI) calcd $C_{17}H_{11}F_3N_2^+$ [M]$^+$: 300.0869. Found: 300.0870.

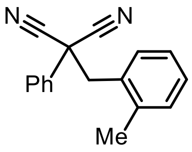

S35

S35 was synthesized using general procedures with corresponding alkyl bromide as a white solid (3.0 mmol scale, 593.0 mg, 80% yield). R$_f$ = 0.5 (hexane/ethyl acetate = 10:1).

^{1}H NMR (500 MHz, CDCl$_3$) δ 7.48 – 7.41 (m, 5H), 7.27 – 7.12 (m, 4H), 3.53 (s, 2H), 2.08 (s, 3H).

^{13}C NMR (126 MHz, CDCl$_3$) δ 137.9, 131.7, 131.2, 130.9, 130.0, 129.9, 129.5, 128.8, 126.22, 126.20, 114.9, 44.7, 43.5, 19.4.

HRMS (ESI) calcd $C_{17}H_{15}N_2^+$ [M+H]$^+$: 247.1230. Found: 247.1230.

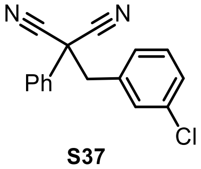

S37

S37 was synthesized using general procedures with corresponding alkyl bromide as a white solid (3.0

mmol scale, 655.5 mg, 82% yield). R_f = 0.6 (hexane/ethyl acetate = 5:1).

^{1}H NMR (400 MHz, CDCl$_3$) δ 7.51 – 7.42 (m, 5H), 7.34 – 7.31 (m, 1H), 7.25 (t, *J* = 8.1 Hz, 1H), 7.08 – 7.01 (m, 2H), 3.42 (s, 2H).

^{13}C NMR (101 MHz, CDCl$_3$) δ 134.4, 133.2, 131.1, 130.5, 130.1, 129.9, 129.6, 129.0, 128.6, 126.0, 114.4, 47.8, 43.6.

HRMS (ESI) calcd $C_{16}H_{12}ClN_2^+$ $[M+H]^+$: 267.0684. Found: 267.0679.

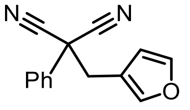

S41

S41 was synthesized using general procedures with corresponding alkyl bromide as colorless oil (2.0 mmol scale, 409.1 mg, 92% yield). R_f = 0.5 (hexane/ethyl acetate = 10:1).

^{1}H NMR (600 MHz, CDCl$_3$) δ 7.55 – 7.44 (m, 5H), 7.37 (s, 1H), 7.32 (d, *J* = 1.4 Hz, 1H), 6.23 (s, 1H), 3.35 (s, 2H).

^{13}C NMR (151 MHz, CDCl$_3$) δ 143.5, 142.2, 131.4, 130.0, 129.6, 126.0, 115.8, 114.8, 111.2, 43.5, 38.9.

HRMS (ESI) calcd $C_{14}H_{11}N_2O^+$ $[M+H]^+$: 223.0866. Found: 223.0866.

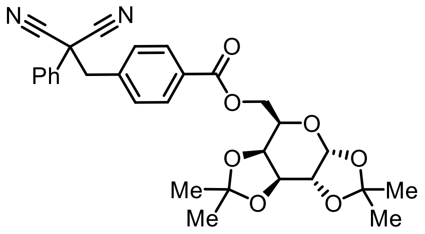

S42

S42 was synthesized using general procedures with corresponding alkyl bromide as a white solid (2.0 mmol scale, 694.0 mg, 67% yield). R_f = 0.5 (hexane/ethyl acetate = 2:1).

^{1}H NMR (500 MHz, CDCl$_3$) δ 7.98 (d, *J* = 8.1 Hz, 2H), 7.53 – 7.36 (m, 5H), 7.19 (d, *J* = 8.1 Hz, 2H), 5.57 (d, *J* = 4.9 Hz, 1H), 4.70 – 4.63 (m, 1H), 4.55 – 4.47 (m, 1H), 4.46 – 4.41 (m, 1H), 4.38 – 4.30 (m, 2H), 4.18 (t, *J* = 5.5 Hz, 1H), 3.52 (s, 2H), 1.53 (s, 3H), 1.48 (s, 3H), 1.36 (s, 3H), 1.34 (s, 3H).

^{13}C NMR (126 MHz, CDCl$_3$) δ 165.8, 136.3, 131.0, 130.50, 130.48, 130.1, 129.9, 129.6, 126.0, 114.4, 109.7, 108.8, 96.3, 71.1, 70.7, 70.5, 66.0, 64.1, 48.1, 43.5, 26.0, 25.9, 24.9, 24.5.

HRMS (ESI) calcd $C_{29}H_{31}N_2O_7^+$ $[M+H]^+$: 519.2126. Found: 519.2126.

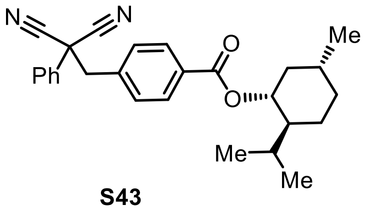

S43

S43 was synthesized using general procedures with corresponding alkyl bromide as a white solid (2.0 mmol scale, 531.0 mg, 64% yield). R_f = 0.6 (hexane/ethyl acetate = 5:1).

^{1}H NMR (500 MHz, $CDCl_3$) δ 7.98 (d, *J* = 8.2 Hz, 2H), 7.53 – 7.45 (m, 5H), 7.21 (d, *J* = 8.2 Hz, 2H), 4.96 – 4.90 (m, 1H), 3.51 (s, 2H), 2.17 – 2.08 (m, 1H), 1.99 – 1.92 (m, 1H), 1.75 – 1.71 (m, 2H), 1.61 – 1.51 (m, 2H), 1.20 – 1.05 (m, 2H), 0.96 – 0.88 (m, 7H), 0.80 (d, *J* = 6.9 Hz, 3H).

^{13}C NMR (126 MHz, $CDCl_3$) δ 165.5, 136.0, 131.3, 131.2, 130.5, 130.2, 129.8, 129.7, 126.1, 114.44, 114.37, 75.1, 48.1, 47.2, 43.6, 40.9, 34.3, 31.4, 26.4, 23.5, 22.0, 20.8, 16.4.

HRMS (ESI) calcd $C_{27}H_{30}N_2O_2Na^+$ $[M+Na]^+$: 437.2199. Found: 437.2199.

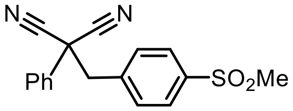

S45

S45 was synthesized using general procedures with corresponding alkyl bromide as a white solid (3.0 mmol scale, 520.5 mg, 56% yield). R_f = 0.2 (hexane/ethyl acetate = 5:1).

^{1}H NMR (400 MHz, $CDCl_3$) δ 7.92 (d, *J* = 8.4 Hz, 2H), 7.52 – 7.48 (m, 5H), 7.38 (d, *J* = 8.3 Hz, 2H), 3.55 (s, 2H), 3.08 (s, 3H).

^{13}C NMR (101 MHz, $CDCl_3$) δ 141.1, 137.5, 131.5, 130.9, 130.4, 129.9, 127.8, 126.0, 114.1, 47.9, 44.4, 43.5.

HRMS (ESI) calcd $C_{17}H_{15}N_2O_2S^+$ $[M+H]^+$: 311.0849. Found: 311.0849.

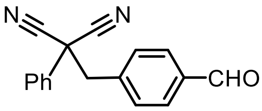

S46

S46 was synthesized using general procedures with corresponding alkyl bromide as a white solid (3.0 mmol scale, 350.0 mg, 45% yield). R_f = 0.3 (hexane/ethyl acetate = 5:1).

^{1}H NMR (400 MHz, $CDCl_3$) δ 10.02 (s, 1H), 7.88 – 7.75 (m, 2H), 7.50 – 7.46 (m, 5H), 7.32 – 7.30 (m, 2H), 3.55 (s, 2H).

^{13}C NMR (101 MHz, $CDCl_3$) δ 191.6, 137.8, 136.5, 131.2, 131.0, 130.3, 129.9, 129.7, 126.1, 114.3,

48.2, 43.5.

HRMS (ESI) calcd $C_{17}H_{13}N_2O^+$ $[M+H]^+$: 261.1022. Found: 261.1022.

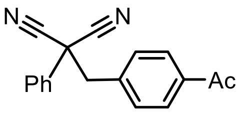

S47

S47 was synthesized using general procedures with corresponding alkyl bromide as a white solid (3.0 mmol scale, 632.3 mg, 77% yield). R_f = 0.5 (hexane/ethyl acetate = 3:1).

^{1}H NMR (500 MHz, CDCl$_3$) δ 7.90 (d, *J* = 8.1 Hz, 2H), 7.51 – 7.44 (m, 5H), 7.24 (d, *J* = 8.1 Hz, 2H), 3.52 (s, 2H), 2.60 (s, 3H).

^{13}C NMR (126 MHz, CDCl$_3$) δ 197.4, 137.3, 136.4, 131.1, 130.7, 130.2, 129.7, 128.6, 126.1, 114.4, 48.1, 43.6, 26.6.

HRMS (ESI) calcd $C_{18}H_{15}N_2O^+$ $[M+H]^+$: 275.1179. Found: 275.1179.

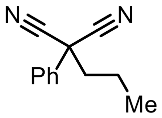

S50

S50 was synthesized using general procedures with corresponding alkyl iodide as colorless oil (3.0 mmol scale, 255.1 mg, 46% yield). R_f = 0.5 (hexane/ethyl acetate = 10:1).

^{1}H NMR (400 MHz, CDCl$_3$) δ 7.57 (dd, *J* = 8.1, 1.7 Hz, 2H), 7.53 – 7.41 (m, 3H), 2.24 – 2.13 (m, 2H), 1.73 – 1.60 (m, 2H), 1.01 (t, *J* = 7.4 Hz, 3H).

^{13}C NMR (101 MHz, CDCl$_3$) δ 132.3, 129.8, 129.7, 125.7, 115.1, 44.5, 42.3, 19.1, 13.1.

HRMS (EI) calcd $C_{12}H_{12}N_2^+$ $[M]^+$: 184.0995. Found: 184.0995.

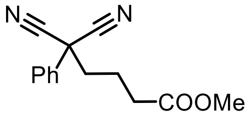

S56

S56 was synthesized using general procedures with corresponding alkyl iodide as colorless oil (3.0 mmol scale, 199.1 mg, 27% yield). R_f = 0.3 (hexane/ethyl acetate = 5:1).

^{1}H NMR (600 MHz, CDCl$_3$) δ 7.57 (d, *J* = 6.9 Hz, 2H), 7.51 – 7.46 (m, 3H), 3.67 (s, 3H), 2.41 (t, *J* = 7.1 Hz, 2H), 2.35 – 2.26 (m, 2H), 2.00 – 1.86 (m, 2H).

^{13}C NMR (151 MHz, CDCl$_3$) δ 172.4, 131.7, 130.0, 129.8, 125.7, 114.8, 51.8, 42.0, 41.6, 32.5, 20.8.

HRMS (ESI) calcd $C_{14}H_{15}N_2O_2^+$ [M+H]$^+$: 243.1128. Found: 243.1128.

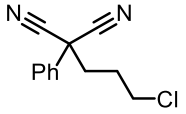

S57

S57 was synthesized using general procedures with corresponding alkyl iodide as colorless oil (2.0 mmol scale, 372.0 mg, 85% yield). R_f = 0.5 (hexane/ethyl acetate = 5:1).

^{1}H NMR (500 MHz, CDCl$_3$) δ 7.59 – 7.57 (m, 2H), 7.54 – 7.45 (m, 3H), 3.59 (t, *J* = 6.1 Hz, 2H), 2.49 – 2.40 (m, 2H), 2.10 (dq, *J* = 12.4, 6.1 Hz, 2H).

^{13}C NMR (126 MHz, CDCl$_3$) δ 131.6, 130.1, 129.9, 125.7, 114.7, 42.9, 41.7, 40.0, 28.3.

HRMS (EI) calcd $C_{12}H_{12}ClN_2^+$ [M]$^+$: 218.0605. Found: 218.0601.

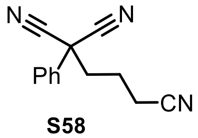

S58

S58 was synthesized using general procedures with corresponding alkyl iodide as colorless oil (3.0 mmol scale, 459.0 mg, 73% yield). R_f = 0.6 (hexane/ethyl acetate = 5:1).

^{1}H NMR (600 MHz, CDCl$_3$) δ 7.60 – 7.56 (m, 2H), 7.56 – 7.48 (m, 3H), 2.48 (t, *J* = 6.9 Hz, 2H), 2.44 – 2.30 (m, 2H), 2.05 – 1.95 (m, 2H).

^{13}C NMR (151 MHz, CDCl$_3$) δ 131.1, 130.4, 130.0, 125.7, 117.8, 114.3, 41.6, 41.1, 21.6, 16.5.

HRMS (ESI) calcd $C_{13}H_{12}N_3^+$ [M+H]$^+$: 210.1026. Found: 210.1025.

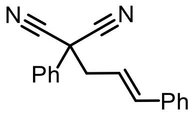

S60

S60 was synthesized using general procedures with corresponding alkyl bromide as a white solid (3.0 mmol scale, 560.0 mg, 72% yield). R_f = 0.5 (hexane/ethyl acetate = 10:1).

^{1}H NMR (400 MHz, CDCl$_3$) δ 7.59 – 7.57 (m, 2H), 7.52 – 7.43 (m, 3H), 7.39 – 7.25 (m, 5H), 6.61 (d, *J* = 15.8 Hz, 1H), 6.11 – 6.07 (m, 1H), 3.10 – 3.07 (m, 2H).

^{13}C NMR (101 MHz, CDCl$_3$) δ 138.2, 135.7, 131.4, 129.9, 129.6, 128.6, 128.4, 126.7, 125.8, 118.8, 114.6, 46.0, 42.8.

HRMS (ESI) calcd $C_{18}H_{15}N_2^+$ [M+H]$^+$: 259.1230. Found: 259.1231.

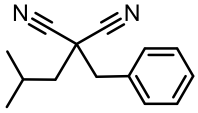

S67

S67 was synthesized using general procedures with corresponding 1-bromo-2-methylpropane as colorless oil (3.0 mmol scale, 439.0 mg, 69% yield). R$_f$ = 0.6 (hexane/ethyl acetate = 10:1).

^{1}H NMR (400 MHz, CDCl$_3$) δ 7.48 – 7.30 (m, 5H), 3.18 (s, 2H), 2.20 – 1.99 (m, 1H), 1.85 (d, *J* = 6.8 Hz, 2H), 1.11 (d, *J* = 6.7 Hz, 6H).

^{13}C NMR (101 MHz, CDCl$_3$) δ 131.9, 130.3, 128.8, 128.7, 115.5, 45.3, 44.6, 37.7, 26.5, 22.8.

HRMS (ESI) calcd $C_{14}H_{17}N_2^+$ [M+H]$^+$: 213.1386. Found: 213.1385.

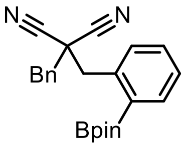

S69

S69 was synthesized using general procedures with corresponding alkyl bromide as a white solid (3.0 mmol scale, 826.0 mg, 74% yield). R$_f$ = 0.4 (hexane/ethyl acetate = 10:1).

^{1}H NMR (500 MHz, CDCl$_3$) δ 7.98 (d, *J* = 7.2 Hz, 1H), 7.52 – 7.31 (m, 8H), 3.71 (s, 2H), 3.27 (s, 2H), 1.34 (s, 12H).

^{13}C NMR (126 MHz, CDCl$_3$) δ 138.9, 137.1, 132.4, 131.4, 130.6, 130.3, 128.8, 128.6, 127.8, 115.3, 84.0, 43.7, 41.5, 41.4, 24.9.

^{11}B NMR (160 MHz, CDCl$_3$) δ 31.63.

HRMS (ESI) calcd $C_{23}H_{26}BN_2O_2^+$ [M+H]$^+$: 373.2082. Found: 373.2082.

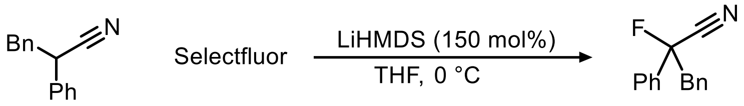

S85

To a 10 mL round bottom flask was added mono-substituted malononitrile (621 mg, 3 mmol, 100 mol%). The flask was sealed with a rubber septum and evacuated/refilled with nitrogen for three times. Dry THF (10 mL) was added and the resulting solution was cooled to 0 °C, followed by the addition of LiHMDS (4.5 mL, 1 M in hexane, 150 mol%). After stirring at 0 °C for 1 h, Selectfluor (2.124 g, 6 mmol, 200 mol%) was added to the mixture. The reaction was allowed to proceed at room temperature overnight, then quenched with saturated aqueous NH_4Cl and extracted with DCM (10 mL × 3). The

organic phase was combined, washed with brine, dried with Na_2SO_4, filtered, and concentrated. The residue was purified by flash column chromatography to afford **S-85** (214.1 mg, 32% Yield). R_f = 0.8 (Hexane/EtOAc = 10:1).

^{1}H NMR (400 MHz, $CDCl_3$) δ 7.44 – 7.40 (m, 5H), 7.30 – 7.27 (m, 3H), 7.14 – 7.12 (m, 2H), 3.56 – 3.32 (m, 2H).

^{13}C NMR (101 MHz, $CDCl_3$) δ 135.6 (d, *J* = 22.7 Hz), 131.9 (d, *J* = 3.6 Hz), 130.6, 129.9 (d, *J* = 1.7 Hz), 128.7, 128.4, 128.0, 124.8 (d, *J* = 6.1 Hz), 116.9 (d, *J* = 33.4 Hz), 91.9 (d, *J* = 186.6 Hz), 48.1 (d, *J* = 25.8 Hz).

^{19}F NMR (376 MHz, $CDCl_3$) δ -146.43 (dd, *J* = 22.8, 16.2 Hz).

HRMS (ESI) calcd $C_{15}H_{12}FNNa^+$ $[M+Na]^+$: 248.0846 Found: 248.0848.

Section 5. General procedures for desymmetric addition

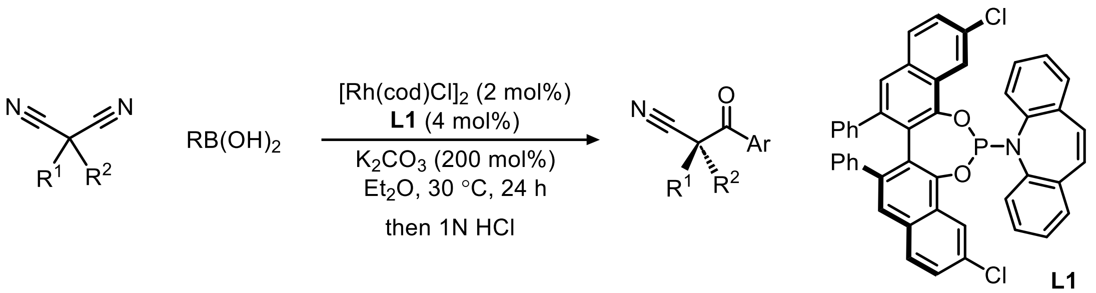

To an oven-dried 4 mL vial equipped with a stir bar were added **L1** (5.9 mg, 0.008 mmol, 4 mol%) and $[Rh(cod)Cl]_2$ (2.0 mg, 0.004 mmol, 2 mol%). The vial was sealed with a rubber septum and evacuated/refilled with nitrogen for three times. Dry Et_2O (2 mL) was added to the vial via a syringe, then the resulting catalyst solution was stirred at room temperature for 15 min.

To a separate oven-dried 15 mL Schlenk tube equipped with a stir bar were added disubstituted malononitrile (0.2 mmol, 100 mol%), K_2CO_3 (55.2 mg, 0.4 mmol, 200 mol%) and boronic acid (0.4 mmol, 200 mol%). The tube was sealed with a rubber septum and evacuated/refilled with nitrogen for three times. Dry Et_2O (2 mL) was added to the reaction tube, followed by the addition of above prepared catalyst solution. The reaction mixture was stirred at 30 °C for 24 h under N_2, and then followed by the addition of aqueous HCl (1 M, 1 mL) and THF (1 mL). The resulting mixture was stirred at room temperature for 15 min, then extracted with EtOAc (10 mL × 3). The organic phase was combined, washed with brine, dried with Na_2SO_4, filtered, and concentrated. The residue was purified by flash column chromatography to afford the product.

Gram-scale desymmetrization: In an argon-filled glovebox, to an oven-dried 4 mL vial equipped with a stir bar were added **L1** (5.9 mg, 0.008 mmol, 0.2 mol%) and $[Rh(cod)Cl]_2$ (2.0 mg, 0.004 mmol, 0.1 mol%). Dry Et_2O (2 mL) was added to the vial via a syringe, then the resulting catalyst solution was stirred at room temperature for 15 min. To a separate oven-dried 150 mL tube equipped with a stir bar were added disubstituted malononitrile **1** (928 mg, 4.0 mmol, 100 mol%), K_2CO_3 (1.104 g, 8.0 mmol, 200 mol%), and phenylboronic acid (976 mg, 8.0 mmol, 200 mol%). Dry Et_2O (80 mL) was added to the reaction tube, followed by the addition of above prepared catalyst solution. The reaction mixture was sealed, removed from the glovebox, and stirred at 30 °C for 48 h, then followed by the addition of aqueous HCl (1 M, 5 mL) and THF (5 mL). The resulting mixture was stirred at room temperature for 15 min, then extracted with EtOAc (20 mL × 3). The organic phase was combined, washed with brine, dried with Na_2SO_4, filtered, and concentrated. The residue was purified by flash column chromatography to afford the product **2** in 89% yield (1.11 g, 95.5:4.5 e.r.).

Section 6. Characterization data of products

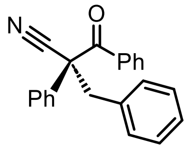

(+)-**2**

(+)-**2** was obtained as a white solid from the desymmetric addition of **S2** using the general procedure (55.9 mg, 90% Yield). R_f = 0.6 (Hexane/EtOAc = 10:1).

HPLC analysis (Chiralpak IC-3, hexane/^{i}PrOH = 99:1, 1.0 mL/min, 254 nm; t_r (minor) = 8.55 min, t_r (major) = 11.32 min) gave the isomeric composition of the product: 95.5:4.5 e.r., $[\alpha]_D^{20}$ = +121.3 (*c* = 1.0, $CHCl_3$).

^{1}H NMR (500 MHz, $CDCl_3$) δ 7.89 – 7.80 (m, 2H), 7.49 – 7.42 (m, 1H), 7.39 – 7.25 (m, 7H), 7.20 – 7.14 (m, 3H), 6.93 – 6.86 (m, 2H), 3.65 (d, *J* = 13.7 Hz, 1H), 3.43 (d, *J* = 13.7 Hz, 1H).

^{13}C NMR (126 MHz, $CDCl_3$) δ 191.0, 134.8, 134.0, 133.8, 133.6, 130.7, 130.1, 129.4, 128.7, 128.4, 127.9, 127.3, 126.4, 119.1, 58.2, 44.7.

HRMS (ESI) calcd $C_{22}H_{18}NO^+$ $[M+H]^+$: 312.1383. Found: 312.1382.

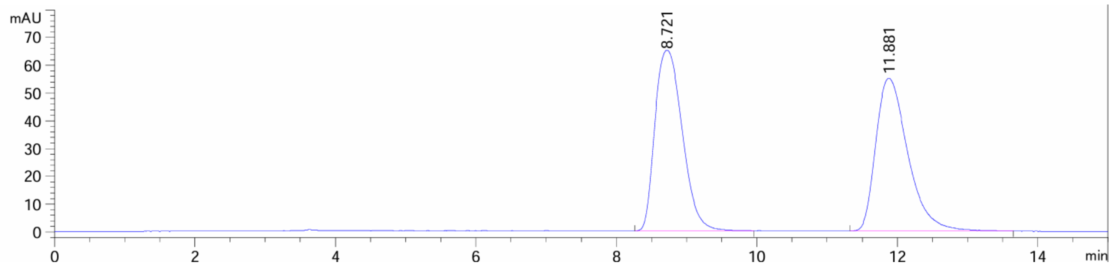

Peak #	RetTime [min]	Type	Width [min]	Area [mAU*s]	Height [mAU]	Area %
1	8.721	BB	0.4354	1759.60229	65.09332	50.1676
2	11.881	BB	0.4924	1747.84351	55.05182	49.8324

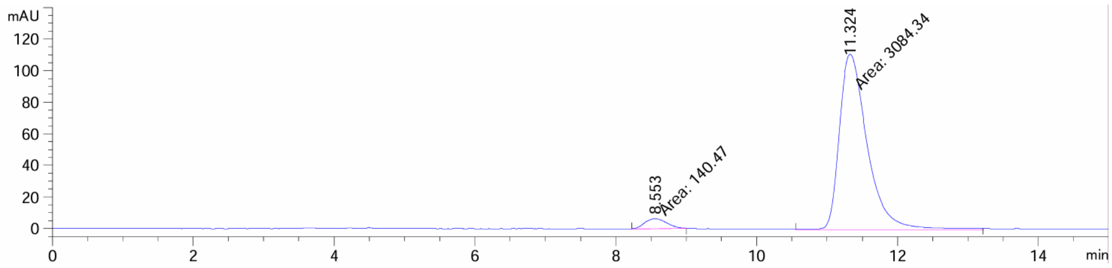

Peak #	RetTime [min]	Type	Width [min]	Area [mAU*s]	Height [mAU]	Area %
1	8.553	MM	0.3683	140.46982	6.35732	4.3559
2	11.324	MM	0.4623	3084.33545	111.18934	95.6441

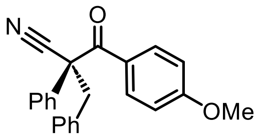

(+)-**3**

(+)-**3** was obtained as a white solid from the desymmetric addition of **S2** using the general procedure (42.1 mg, 62% Yield). R_f = 0.6 (Hexane/EtOAc = 5:1).

HPLC analysis (Chiralpak IC-3, hexane/^{i}PrOH = 98/2, 1.0 mL/min, 254 nm; tr (minor) = 10.59 min, tr (major) = 12.05 min) gave the isomeric composition of the product: 95:5 e.r., $[\alpha]_D^{20}$ = +97.5 (*c* = 1.5, $CHCl_3$).

^{1}H NMR (400 MHz, $CDCl_3$) δ 7.86 (d, *J* = 8.9 Hz, 2H), 7.35 – 7.32 (m, 3H), 7.26 – 7.21 (m, 2H), 7.20 – 7.14 (m, 3H), 6.92 – 6.85 (m, 2H), 6.80 (d, *J* = 9.0 Hz, 2H), 3.79 (s, 3H), 3.63 (d, *J* = 13.7 Hz, 1H), 3.41 (d, *J* = 13.7 Hz, 1H).

^{13}C NMR (101 MHz, $CDCl_3$) δ 189.3, 163.8, 135.3, 134.2, 132.7, 130.7, 129.4, 128.6, 127.9, 127.2, 126.44, 126.38, 119.4, 113.7, 57.8, 55.5, 44.8.

HRMS (ESI) calcd $C_{23}H_{20}NO_2^+$ $[M+H]^+$: 342.1489. Found: 342.1489.

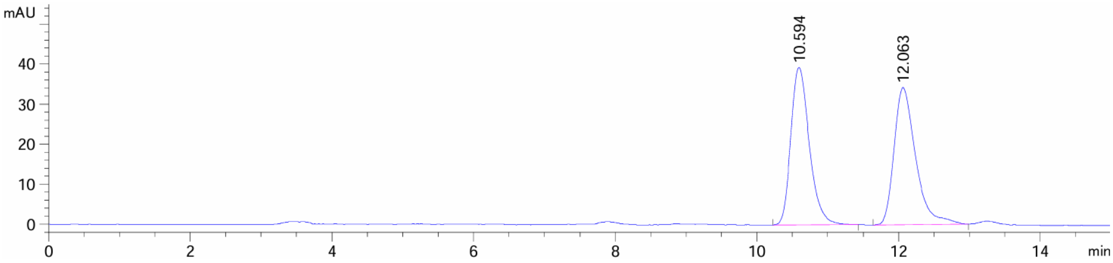

Peak #	RetTime [min]	Type	Width [min]	Area [mAU*s]	Height [mAU]	Area %
1	10.594	BB	0.2771	711.83112	39.34023	49.5367
2	12.063	BB	0.3203	725.14722	34.34422	50.4633

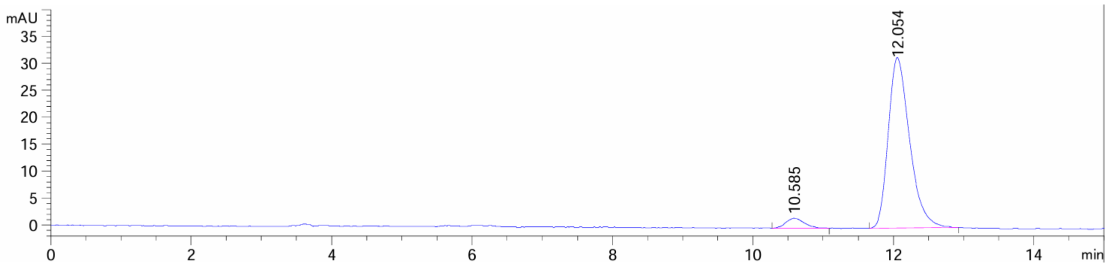

Peak #	RetTime [min]	Type	Width [min]	Area [mAU*s]	Height [mAU]	Area %
1	10.585	BB	0.2446	34.42202	1.83719	4.8808
2	12.054	BB	0.3249	670.83301	31.70432	95.1192

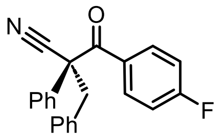

(+)-**4**

(+)-**4** was obtained as colorless oil from the desymmetric addition of **S2** using the general procedure (49.1 mg, 75% Yield). R_f = 0.7 (Hexane/EtOAc = 10:1).

HPLC analysis (Chiralpak IC-3, hexane/^{i}PrOH = 99/1, 1.0 mL/min, 254 nm; tr (minor) = 7.16 min, tr (major) = 8.94 min) gave the isomeric composition of the product: 92:8 e.r., $[\alpha]_D^{20}$ = +106.4 (*c* = 1.0, $CHCl_3$).

^{1}H NMR (500 MHz, $CDCl_3$) δ 7.92 – 7.84 (m, 2H), 7.36 – 7.33 (m, 3H), 7.26 – 7.24 (m, 2H), 7.21 – 7.14 (m, 3H), 7.03 – 6.95 (m, 2H), 6.92 – 6.84 (m, 2H), 3.64 (d, *J* = 13.7 Hz, 1H), 3.41 (d, *J* = 13.7 Hz, 1H).

^{13}C NMR (126 MHz, $CDCl_3$) δ 189.5, 165.7 (d, *J* = 257.3 Hz), 134.7, 133.9, 132.9 (d, *J* = 9.5 Hz), 130.7, 130.1 (d, *J* = 3.1 Hz), 129.5, 128.8, 127.9, 127.4, 126.3, 119.0, 115.7 (d, *J* = 21.4 Hz), 58.1, 44.7.

^{19}F NMR (377 MHz, $CDCl_3$) δ -103.01.

HRMS (ESI) calcd $C_{22}H_{17}FNO^+$ $[M+H]^+$: 330.1289. Found: 330.1289.

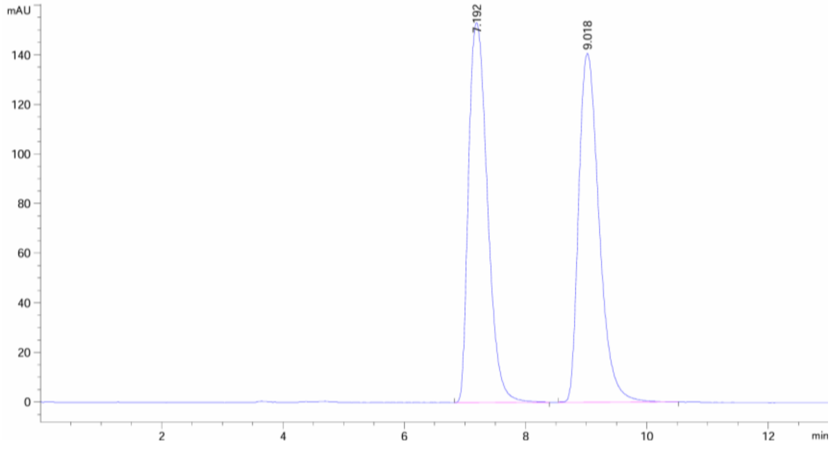

Peak #	RetTime [min]	Type	Width [min]	Area [mAU*s]	Height [mAU]	Area %
1	7.192	BB	0.3444	3310.28076	153.00963	50.1842
2	9.018	BB	0.3665	3285.97534	140.66191	49.8158

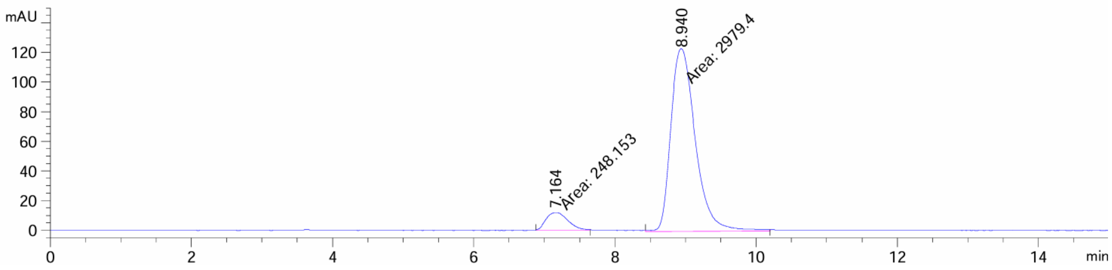

Peak #	RetTime [min]	Type	Width [min]	Area [mAU*s]	Height [mAU]	Area %
1	7.164	MM	0.3517	248.15341	11.75973	7.6886
2	8.940	MM	0.4025	2979.39575	123.38216	92.3114

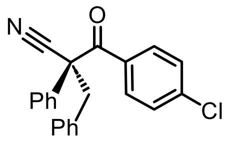

(+)-**5**

(+)-**5** was obtained as colorless oil from the desymmetric addition of **S2** using the general procedure (52.2 mg, 76% Yield). R_f = 0.7 (Hexane/EtOAc = 10:1).

HPLC analysis (Chiralpak IC-3, hexane/^{i}PrOH = 99/1, 1.0 mL/min, 254 nm; tr (minor) = 9.15 min, tr (major) = 10.91 min) gave the isomeric composition of the product: 94:6 e.r., $[\alpha]_D^{20}$ = +76.7 (c = 1.0, $CHCl_3$).

^{1}H NMR (400 MHz, $CDCl_3$) δ 7.78 (d, J = 8.7 Hz, 2H), 7.37 – 7.33 (m, 3H), 7.33 – 7.28 (m, 2H), 7.26 – 7.22 (m, 2H), 7.22 – 7.12 (m, 3H), 6.89 (d, J = 6.9 Hz, 2H), 3.63 (d, J = 13.7 Hz, 1H), 3.41 (d, J = 13.7 Hz, 1H).

^{13}C NMR (101 MHz, $CDCl_3$) δ 189.9, 140.3, 134.5, 133.8, 132.1, 131.5, 130.7, 129.6, 128.9, 128.8, 128.0, 127.4, 126.4, 118.9, 58.2, 44.7.

HRMS (ESI) calcd $C_{22}H_{17}ClNO^+$ $[M+H]^+$: 346.0993. Found: 346.0992.

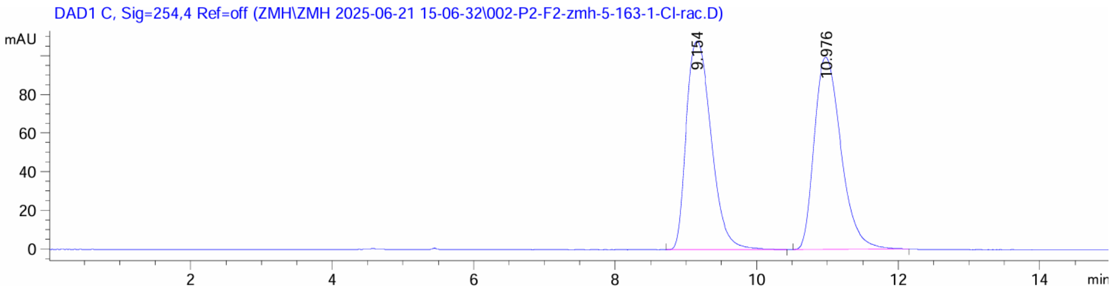

Peak #	RetTime [min]	Type	Width [min]	Area [mAU*s]	Height [mAU]	Area %
1	9.154	BB	0.3893	2632.15381	107.63993	50.3123
2	10.976	BB	0.4138	2599.47607	99.18465	49.6877

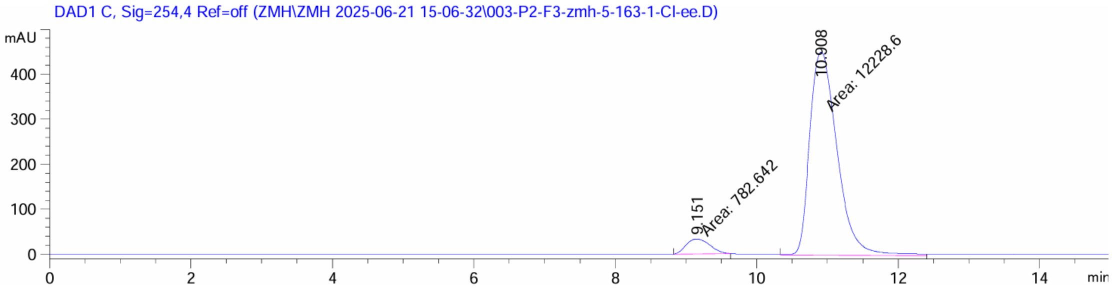

Peak #	RetTime [min]	Type	Width [min]	Area [mAU*s]	Height [mAU]	Area %
1	9.151	MM	0.3918	782.64233	33.29001	6.0151
2	10.908	MM	0.4512	1.22286e4	451.74432	93.9849

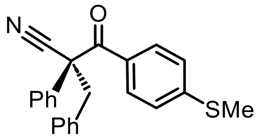

(+)-**6**

(+)-**6** was obtained as colorless oil from the desymmetric addition of **S2** using the general procedure (52.0 mg, 73% Yield). R_f = 0.5 (Hexane/EtOAc = 10:1).

HPLC analysis (Chiralpak IC-3, hexane/^{i}PrOH = 98:2, 1.0 mL/min, 230 nm; t_r (minor) = 9.33 min, t_r (major) = 10.39 min) gave the isomeric composition of the product: 94.5:5.5 e.r., $[\alpha]_D^{20}$ = +112.2 (*c* = 1.0, $CHCl_3$).

^{1}H NMR (400 MHz, $CDCl_3$) δ 7.77 (d, *J* = 8.7 Hz, 2H), 7.34 – 7.31 (m, 3H), 7.28 – 7.22 (m, 2H), 7.21 – 7.07 (m, 5H), 6.88 (d, *J* = 6.2 Hz, 2H), 3.63 (d, *J* = 13.7 Hz, 1H), 3.41 (d, *J* = 13.7 Hz, 1H), 2.43 (s, 3H).

^{13}C NMR (101 MHz, $CDCl_3$) δ 189.8, 147.3, 135.1, 134.1, 130.7, 130.5, 129.7, 129.4, 128.6, 127.9, 127.3, 126.4, 124.5, 119.2, 57.9, 44.7, 14.5.

HRMS (ESI) calcd $C_{23}H_{20}NOS^+$ $[M+H]^+$: 358.1260. Found: 358.1260.

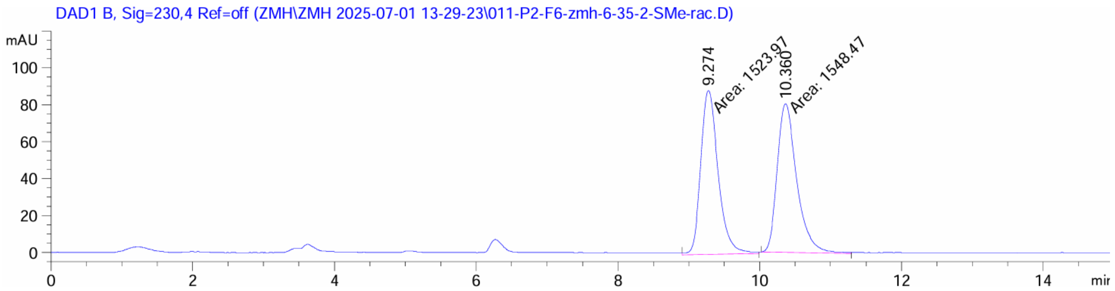

Peak #	RetTime [min]	Type	Width [min]	Area [mAU*s]	Height [mAU]	Area %
1	9.274	MM	0.2850	1523.96973	89.12803	49.6013
2	10.360	MM	0.3194	1548.47241	80.81089	50.3987

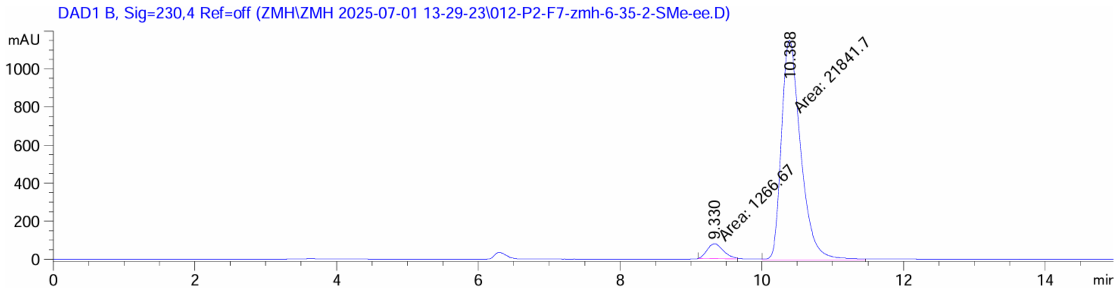

Peak #	RetTime [min]	Type	Width [min]	Area [mAU*s]	Height [mAU]	Area %
1	9.330	MM	0.2678	1266.66870	78.83557	5.4814
2	10.388	MM	0.3155	2.18417e4	1153.95972	94.5186

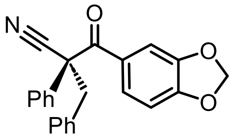

(+)-**7**

(+)-**7** was obtained as a white solid from the desymmetric addition of **S2** using the general procedure (48.3 mg, 68% Yield). R_f = 0.6 (Hexane/EtOAc = 10:1).

HPLC analysis (Chiralpak IC-3, hexane/^{i}PrOH = 98:2, 1.0 mL/min, 230 nm; t_r (minor) = 14.57 min, t_r (major) = 16.08 min) gave the isomeric composition of the product: 96:4 e.r., $[\alpha]_D^{20}$ = +77.8 (*c* = 1.0, $CHCl_3$).

H NMR (400 MHz, $CDCl_3$) δ 7.48 (d, *J* = 8.3 Hz, 1H), 7.36 – 7.29 (m, 4H), 7.25 – 7.13 (m, 5H), 6.87 (d, *J* = 7.2 Hz, 2H), 6.69 (d, *J* = 8.5 Hz, 1H), 5.97 (s, 2H), 3.62 (d, *J* = 13.6 Hz, 1H), 3.39 (d, *J* = 13.6 Hz, 1H).

^{13}C NMR (101 MHz, $CDCl_3$) δ 189.0, 152.2, 147.8, 135.2, 134.1, 130.7, 129.4, 128.6, 128.1, 127.9, 127.3, 127.2, 126.3, 119.2, 109.8, 107.9, 102.0, 57.9, 44.9.

HRMS (ESI) calcd $C_{23}H_{18}NO_3^+$ $[M+H]^+$: 356.1281. Found: 356.1281.

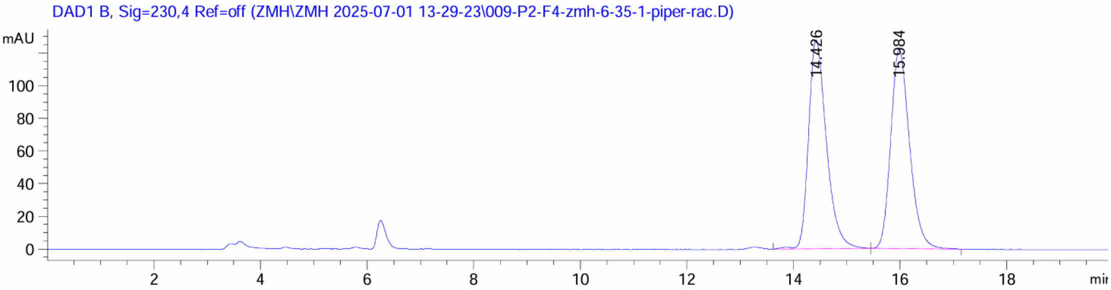

Peak #	RetTime [min]	Type	Width [min]	Area [mAU*s]	Height [mAU]	Area %
1	14.426	BB	0.3537	2975.10571	127.74868	49.9144
2	15.984	BB	0.3775	2985.31470	121.98604	50.0856

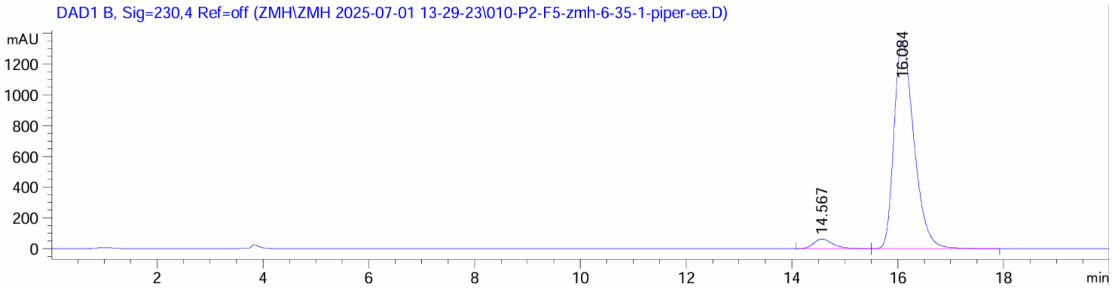

Peak #	RetTime [min]	Type	Width [min]	Area [mAU*s]	Height [mAU]	Area %
1	14.567	BB	0.3872	1554.09155	62.26714	4.2226
2	16.084	BB	0.4005	3.52499e4	1350.77722	95.7774

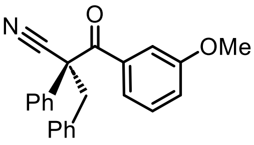

(+)-**8**

(+)-**8** was obtained as colorless oil from the desymmetric addition of **S2** using the general procedure (47.5 mg, 70% Yield). R_f = 0.6 (Hexane/EtOAc = 10:1).

HPLC analysis (Chiralpak IC-3, hexane/^{i}PrOH = 98/2, 1.0 mL/min, 254 nm; tr (minor) = 8.31 min, tr (major) = 10.29 min) gave the isomeric composition of the product: 95:5 e.r., $[\alpha]_D^{20}$ = +53.8 (*c* = 1.0, $CHCl_3$).

^{1}H NMR (400 MHz, $CDCl_3$) δ 7.49 – 7.07 (m, 11H), 7.01 (d, *J* = 8.2 Hz, 1H), 6.90 (d, *J* = 7.2 Hz, 2H), 3.72 (s, 3H), 3.65 (d, *J* = 13.7 Hz, 1H), 3.42 (d, *J* = 13.9 Hz, 1H).

^{13}C NMR (101 MHz, $CDCl_3$) δ 190.9, 159.4, 135.0, 134.9, 134.0, 130.7, 129.44, 129.36, 128.7, 127.9, 127.3, 126.4, 122.7, 120.4, 119.0, 114.2, 58.4, 55.3, 44.7.

HRMS (ESI) calcd $C_{23}H_{20}NO_2^+$ [M+H]$^+$: 342.1489. Found: 342.1487.

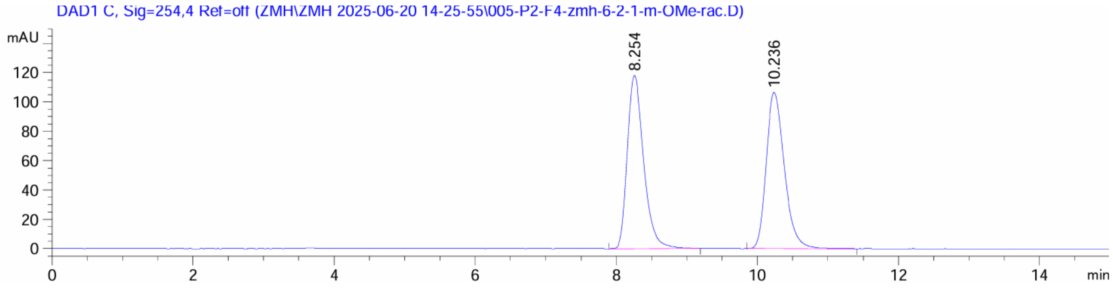

Peak #	RetTime [min]	Type	Width [min]	Area [mAU*s]	Height [mAU]	Area %
1	8.254	BB	0.2479	1902.90466	118.12368	49.9577
2	10.236	BB	0.2764	1906.12964	106.71526	50.0423

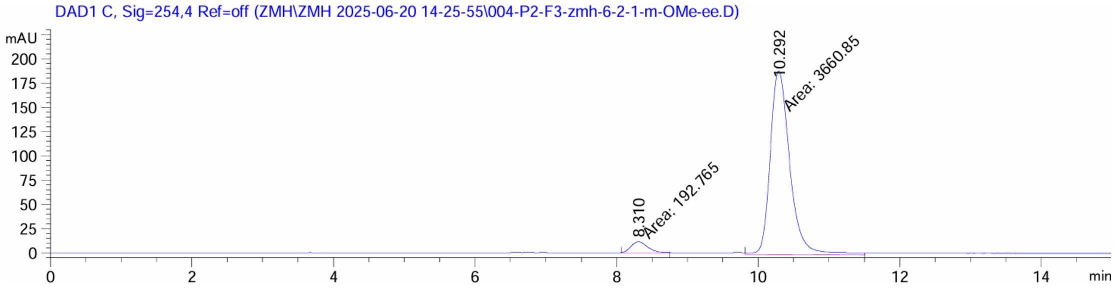

Peak #	RetTime [min]	Type	Width [min]	Area [mAU*s]	Height [mAU]	Area %
1	8.310	MM	0.2792	192.76465	11.50649	5.0022
2	10.292	MM	0.3226	3660.85425	189.12828	94.9978

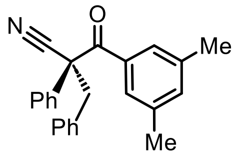

(+)-**9**

(+)-**9** was obtained as colorless oil from the desymmetric addition of **S2** using the general procedure (55.8 mg, 82% Yield). R_f = 0.7 (Hexane/EtOAc = 10:1).

HPLC analysis (Chiralpak IC-3, hexane/^{i}PrOH = 99/1, 1.0 mL/min, 254 nm; tr (minor) = 7.31 min, tr (major) = 9.54 min) gave the isomeric composition of the product: 94:6 e.r., $[\alpha]_D^{20}$ = +37.5 (c = 1.0, $CHCl_3$).

^{1}H NMR (400 MHz, $CDCl_3$) δ 7.42 (s, 2H), 7.39 – 7.26 (m, 5H), 7.23 – 7.12 (m, 3H), 7.10 (s, 1H), 6.96 – 6.86 (m, 2H), 3.64 (d, J = 13.7 Hz, 1H), 3.41 (d, J = 13.7 Hz, 1H), 2.23 (s, 6H).

^{13}C NMR (101 MHz, $CDCl_3$) δ 191.5, 138.0, 135.4, 135.1, 134.2, 134.1, 130.7, 129.4, 128.6, 127.9,

127.8, 127.3, 126.4, 119.2, 58.3, 44.9, 21.2.

HRMS (ESI) calcd $C_{24}H_{22}NO^+$ $[M+H]^+$: 340.1696. Found: 340.1696.

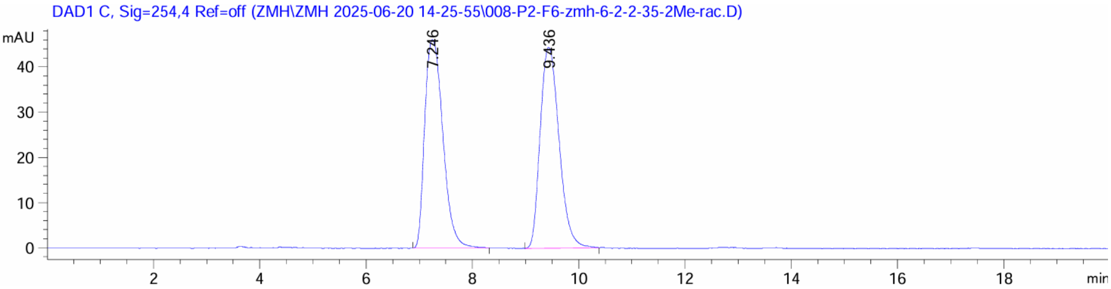

Peak #	RetTime [min]	Type	Width [min]	Area [mAU*s]	Height [mAU]	Area %
1	7.246	BB	0.3808	1093.89453	46.10827	50.0363
2	9.436	BB	0.3908	1092.30859	44.43489	49.9637

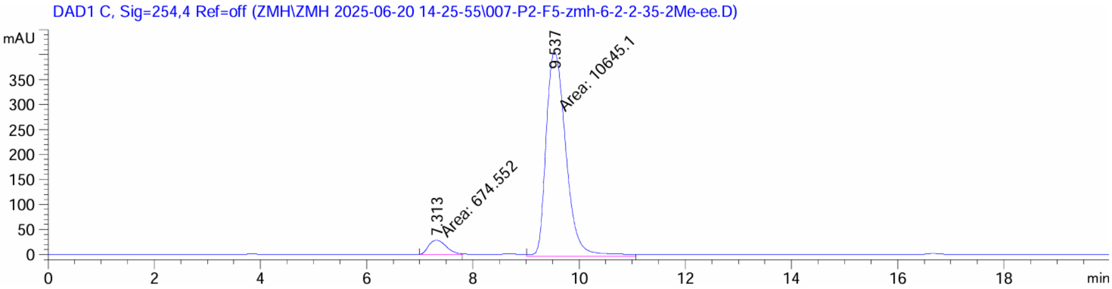

Peak #	RetTime [min]	Type	Width [min]	Area [mAU*s]	Height [mAU]	Area %
1	7.313	MM	0.3918	674.55231	28.69628	5.9591
2	9.537	MM	0.4344	1.06451e4	408.43219	94.0409

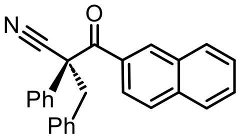

(+)-**10**

(+)-**10** was obtained as colorless oil from the desymmetric addition of **S2** using the general procedure (62.4 mg, 86% Yield). R_f = 0.6 (Hexane/EtOAc = 10:1).

HPLC analysis (Chiralpak IC-3, hexane/^{i}PrOH = 98/2, 1.0 mL/min, 254 nm; tr (minor) = 7.84 min, tr (major) = 9.23 min) gave the isomeric composition of the product: 93:7 e.r., $[\alpha]_D^{20}$ = +32.0 (c = 1.0, $CHCl_3$).

^{1}H NMR (400 MHz, $CDCl_3$) δ 8.40 (s, 1H), 7.90 – 7.87 (m, 1H), 7.83 – 7.72 (m, 3H), 7.56 (t, J = 7.5 Hz, 1H), 7.47 (t, J = 7.5 Hz, 1H), 7.38 – 7.33 (m, 5H), 7.24 – 7.11 (m, 3H), 6.98 – 6.88 (m, 2H), 3.71 (d, J = 13.6 Hz, 1H), 3.48 (d, J = 13.6 Hz, 1H).

^{13}C NMR (101 MHz, CDCl$_3$) δ 191.0, 135.5, 135.1, 134.1, 132.6, 132.0, 131.1, 130.8, 130.0, 129.5, 129.1, 128.7, 128.3, 128.0, 127.6, 127.3, 126.8, 126.5, 125.1, 119.2, 58.3, 44.9.

HRMS (ESI) calcd $C_{26}H_{20}NO^+$ $[M+H]^+$: 362.1539. Found: 362.1539.

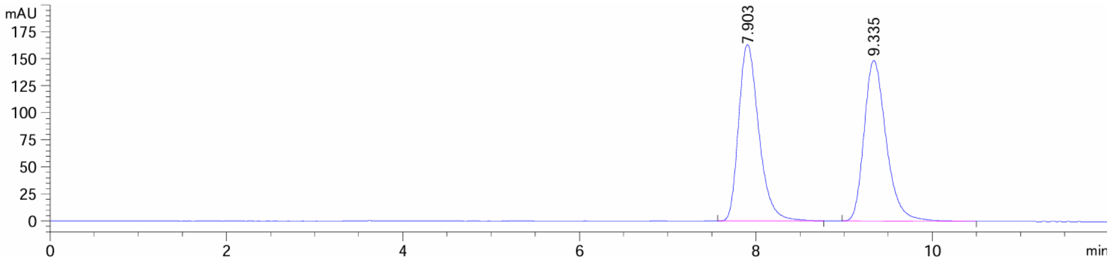

Peak #	RetTime [min]	Type	Width [min]	Area [mAU*s]	Height [mAU]	Area %
1	7.903	BB	0.2457	2596.42114	163.08972	50.1228
2	9.335	BB	0.2668	2583.69849	148.60049	49.8772

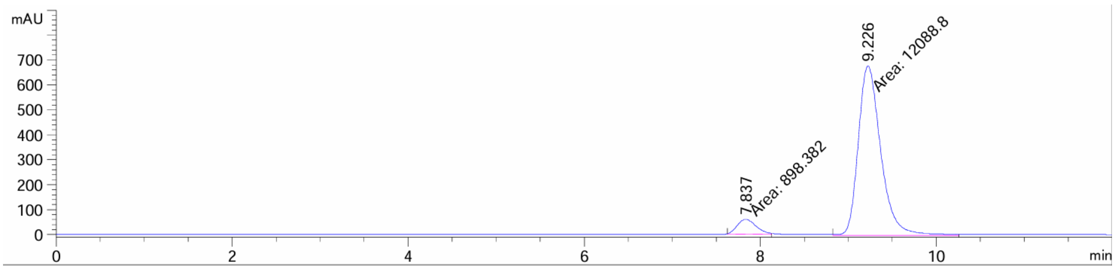

Peak #	RetTime [min]	Type	Width [min]	Area [mAU*s]	Height [mAU]	Area %
1	7.837	MM	0.2513	898.38153	59.58317	6.9175
2	9.226	MM	0.2966	1.20888e4	679.40259	93.0825

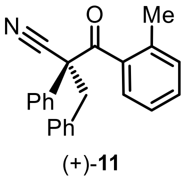

(+)-**11**

(+)-**11** was obtained as a white solid from the desymmetric addition of **S2** using the general procedure (41.1 mg, 63% Yield). R_f = 0.5 (Hexane/EtOAc = 5:1).

HPLC analysis (Chiralpak IC, hexane/^{i}PrOH = 99:1, 0.8 mL/min, 254 nm; t_r (minor) = 9.59 min, t_r (major) = 10.55 min) gave the isomeric composition of the product: 57:43 e.r. $[\alpha]_D^{20}$ = +11.1 (c = 1.0, CHCl$_3$).

^{1}H NMR (400 MHz, CDCl$_3$) δ 7.52 – 7.46 (m, 2H), 7.44 – 7.36 (m, 3H), 7.30 – 7.15 (m, 5H), 7.15 –

7.07 (m, 2H), 7.04 – 6.95 (m, 2H), 3.77 (d, *J* = 13.6 Hz, 1H), 3.39 (d, *J* = 13.6 Hz, 1H), 2.25 (s, 3H).

^{13}C NMR (101 MHz, $CDCl_3$) δ 195.4, 138.1, 135.8, 134.5, 134.4, 131.6, 131.5, 130.8, 129.4, 128.9, 128.1, 127.9, 127.5, 126.5, 125.0, 119.2, 61.0, 44.7, 20.4.

HRMS (ESI) calcd $C_{23}H_{20}NO^+$ $[M+H]^+$: 326.1539. Found: 326.159.

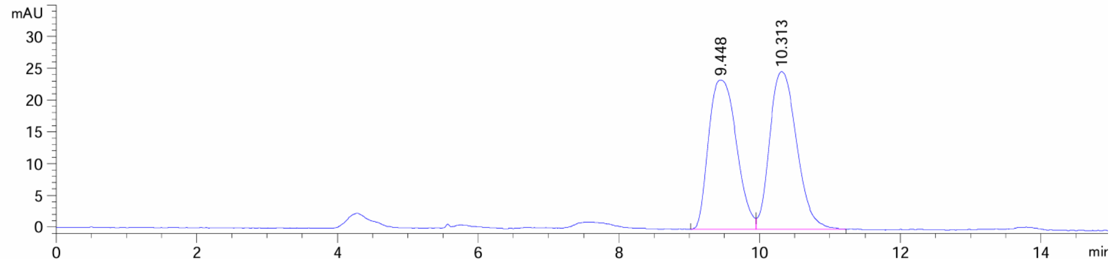

Peak #	RetTime [min]	Type	Width [min]	Area [mAU*s]	Height [mAU]	Area %
1	9.448	BV	0.4438	648.02881	23.49511	49.4454
2	10.313	VB	0.4255	662.56519	24.81392	50.5546

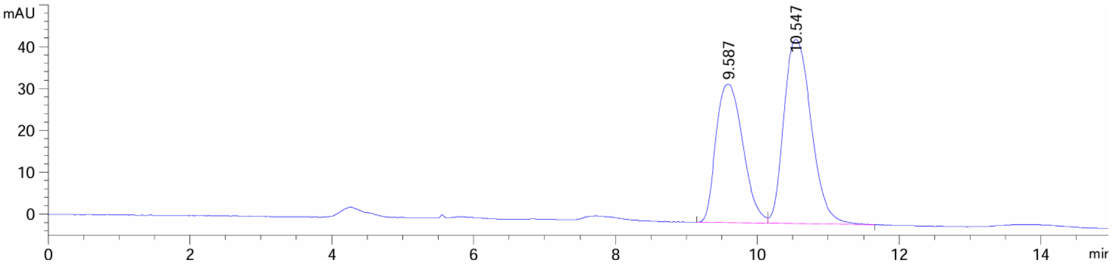

Peak #	RetTime [min]	Type	Width [min]	Area [mAU*s]	Height [mAU]	Area %
1	9.587	BV	0.4349	875.75415	33.07222	42.5281
2	10.547	VB	0.4281	1183.48059	43.95893	57.4719

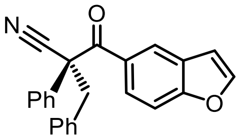

(+)-**12**

(+)-**12**was obtained as colorless oil from the desymmetric addition of **S2** using the general procedure (50.4 mg, 72% Yield). R_f = 0.4 (Hexane/EtOAc = 10:1).

HPLC analysis (Chiralpak IC-3, hexane/^{i}PrOH = 99:1, 1.0 mL/min, 254 nm; t_r (minor) = 12.12 min, t_r (major) = 14.59 min) gave the isomeric composition of the product: 94:6 e.r., $[\alpha]_D^{20}$ = +67.3 (*c* = 1.0, $CHCl_3$).

^{1}H NMR (400 MHz, $CDCl_3$) δ 8.16 (d, *J* = 1.9 Hz, 1H), 7.88 – 7.85 (m, 1H), 7.63 (d, *J* = 2.3 Hz, 1H), 7.42 (d, *J* = 8.8 Hz, 1H), 7.39 – 7.28 (m, 5H), 7.23 – 7.13 (m, 3H), 6.91 (d, *J* = 6.4 Hz, 2H), 6.76 (d, *J* = 2.2 Hz, 1H), 3.68 (d, *J* = 13.6 Hz, 1H), 3.45 (d, *J* = 13.6 Hz, 1H).

^{13}C NMR (101 MHz, $CDCl_3$) δ 190.5, 157.5, 146.5, 135.2, 134.2, 130.8, 129.5, 129.2, 128.7, 127.9, 127.3, 127.3, 126.8, 126.4, 124.7, 119.4, 111.5, 107.4, 58.2, 45.0.

HRMS (ESI) calcd $C_{24}H_{18}NO_2^+$ $[M+H]^+$: 352.1332. Found: 352.1332.

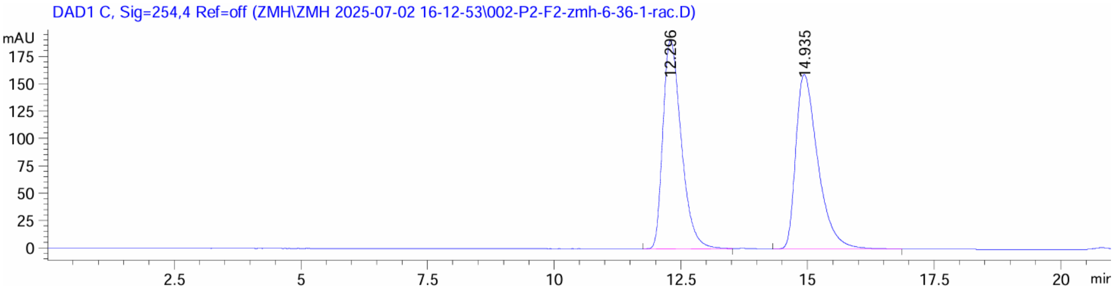

Peak #	RetTime [min]	Type	Width [min]	Area [mAU*s]	Height [mAU]	Area %
1	12.296	BB	0.3900	4808.03711	190.81866	49.8214
2	14.935	BB	0.4620	4842.51709	159.60147	50.1786

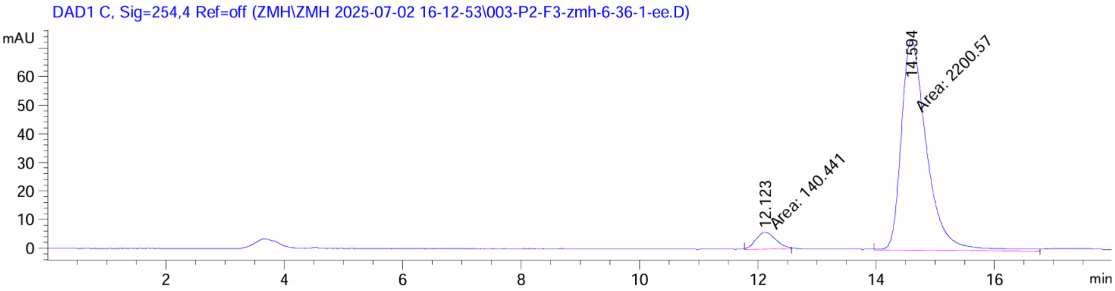

Peak #	RetTime [min]	Type	Width [min]	Area [mAU*s]	Height [mAU]	Area %
1	12.123	MM	0.3949	140.44063	5.92685	5.9992
2	14.594	MM	0.4984	2200.56519	73.58897	94.0008

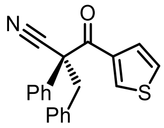

(+)-**13**

(+)-**13** was obtained as colorless oil from the desymmetric addition of **S2** using the general procedure (20.1 mg, 32% Yield). R_f = 0.5 (Hexane/EtOAc = 10:1).

HPLC analysis (Chiralpak IC-3, hexane/^{i}PrOH = 99:1, 1.0 mL/min, 254 nm; t_r (minor) = 11.81 min, t_r (major) = 16.93 min) gave the isomeric composition of the product: 94.5:5.5 e.r., $[\alpha]_D^{20}$ = +49.36 (*c* = 1.0, $CHCl_3$).

^{1}H NMR (400 MHz, CDCl$_3$) δ 8.04 – 7.97 (m, 1H), 7.46 (d, *J* = 5.1 Hz, 1H), 7.38 – 7.31 (m, 3H), 7.30 – 7.26 (m, 2H), 7.23 – 7.12 (m, 4H), 6.90 (d, *J* = 7.2 Hz, 2H), 3.65 (d, *J* = 13.7 Hz, 1H), 3.39 (d, *J* = 13.7 Hz, 1H).

^{13}C NMR (101 MHz, CDCl$_3$) δ 184.9, 137.8, 135.4, 134.9, 134.1, 130.7, 129.4, 128.8, 128.2, 128.0, 127.3, 126.6, 125.8, 119.2, 58.7, 44.1.

HRMS (ESI) calcd $C_{20}H_{16}NOS^+$ $[M+H]^+$: 318.0947. Found: 318.0949.

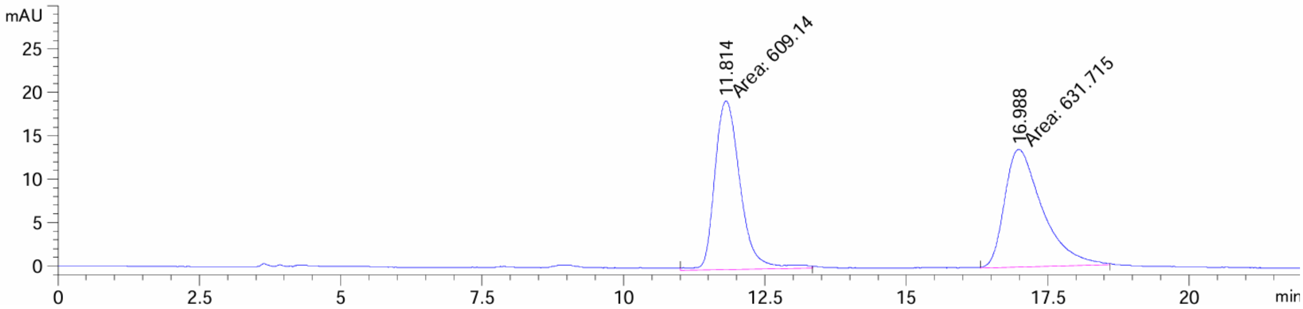

Peak #	RetTime [min]	Type	Width [min]	Area [mAU*s]	Height [mAU]	Area %
1	11.814	MM	0.5232	609.13983	19.40523	49.0904
2	16.988	MM	0.7783	631.71454	13.52791	50.9096

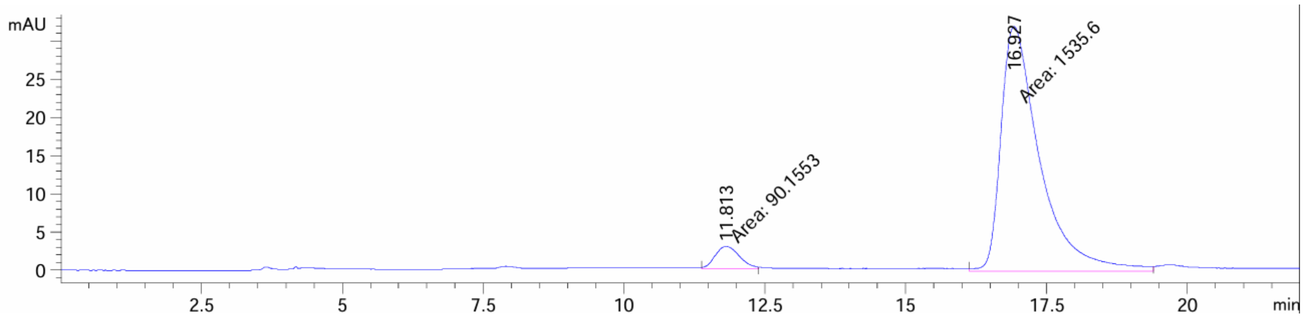

Peak #	RetTime [min]	Type	Width [min]	Area [mAU*s]	Height [mAU]	Area %
1	11.813	MM	0.5098	90.15527	2.94715	5.5454
2	16.927	MM	0.7997	1535.60046	32.00454	94.4546

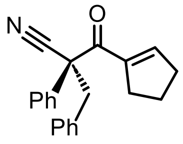

(+)-**14**

(+)-**14** was obtained as colorless oil from the desymmetric addition of **S2** using the general procedure (54.9 mg, 91% Yield). R_f = 0.7 (Hexane/EtOAc = 10:1).

HPLC analysis (Chiralpak IC-3, hexane/^{i}PrOH = 99:1, 1.0 mL/min, 254 nm; t_r (minor) = 8.07 min, t_r (major) = 9.82 min) gave the isomeric composition of the product: 96:4 e.r., $[\alpha]_D^{20}$ = +65.1 (c = 1.0, $CHCl_3$).

^{1}H NMR (400 MHz, $CDCl_3$) δ 7.39 – 7.28 (m, 3H), 7.23 – 7.09 (m, 5H), 6.88 (d, J = 6.6 Hz, 2H), 6.74 (s, 1H), 3.54 (d, J = 13.7 Hz, 1H), 3.29 (d, J = 13.7 Hz, 1H), 2.67 – 2.31 (m, 4H), 1.81 (p, J = 7.6 Hz, 2H).

^{13}C NMR (101 MHz, $CDCl_3$) δ 188.6, 148.8, 140.9, 135.3, 134.3, 130.6, 129.2, 128.4, 127.8, 127.2, 126.2, 119.4, 58.3, 44.1, 34.4, 31.9, 22.0.

HRMS (ESI) calcd $C_{21}H_{20}NO^{+}$ $[M+H]^{+}$: 302.1539. Found: 302.1539.

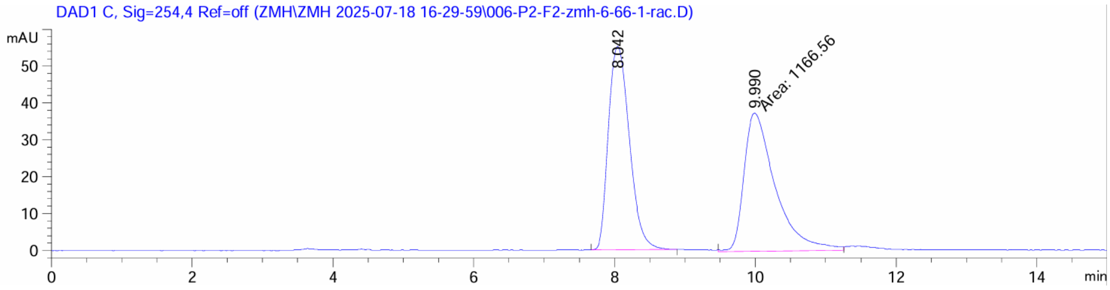

Peak #	RetTime [min]	Type	Width [min]	Area [mAU*s]	Height [mAU]	Area %
1	8.042	BB	0.3304	1146.86780	55.20967	49.5744
2	9.990	MM	0.5176	1166.55920	37.56269	50.4256

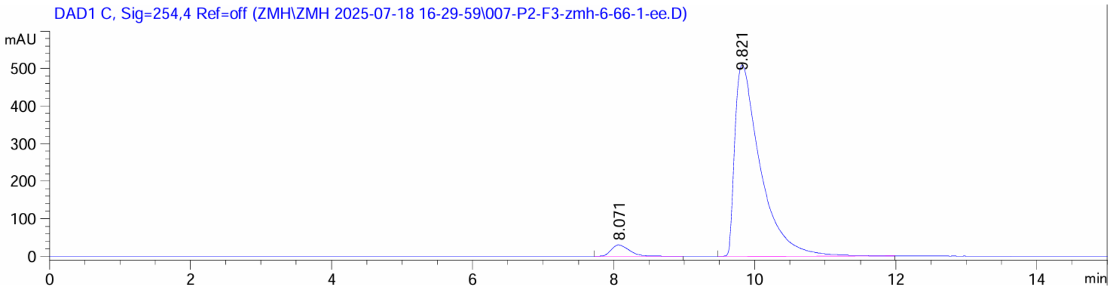

Peak #	RetTime [min]	Type	Width [min]	Area [mAU*s]	Height [mAU]	Area %
1	8.071	BB	0.2810	544.18024	30.08256	4.0756
2	9.821	BB	0.3715	1.28080e4	509.13126	95.9244

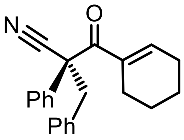

(+)-**15**

(+)-**15** was obtained as colorless oil from the desymmetric addition of **S2** using the general procedure

(53.0 mg, 84% Yield). R_f = 0.7 (Hexane/EtOAc = 10:1).

HPLC analysis (Chiralpak IC-3, hexane/^{i}PrOH = 99:1, 1.0 mL/min, 254 nm; t_r (minor) = 7.38 min, t_r (major) = 8.97 min) gave the isomeric composition of the product: 94.5:5.5 e.r., $[\alpha]_D^{20}$ = +87.6 (*c* = 1.0, $CHCl_3$).

^{1}H NMR (400 MHz, $CDCl_3$) δ 7.35 – 7.31 (m, 3H), 7.20 – 7.11 (m, 5H), 6.85 (d, *J* = 6.3 Hz, 3H), 3.51 (d, *J* = 13.7 Hz, 1H), 3.29 (d, *J* = 13.7 Hz, 1H), 2.44 – 2.30 (m, 1H), 2.28 – 1.92 (m, 3H), 1.65 – 1.40 (m, 4H).

^{13}C NMR (101 MHz, $CDCl_3$) δ 191.6, 145.4, 135.8, 135.3, 134.3, 130.7, 129.2, 128.3, 127.8, 127.1, 125.9, 119.6, 57.3, 44.9, 26.0, 24.1, 21.7, 21.0.

HRMS (ESI) calcd $C_{22}H_{22}NO^+$ $[M+H]^+$: 316.1696. Found: 316.1696.

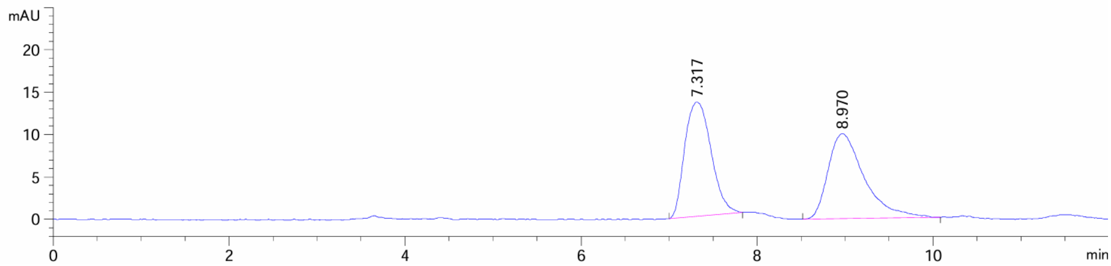

Peak #	RetTime [min]	Type	Width [min]	Area [mAU*s]	Height [mAU]	Area %
1	7.317	BB	0.3373	281.31567	13.48765	49.4545
2	8.970	BB	0.4078	287.52191	10.04770	50.5455

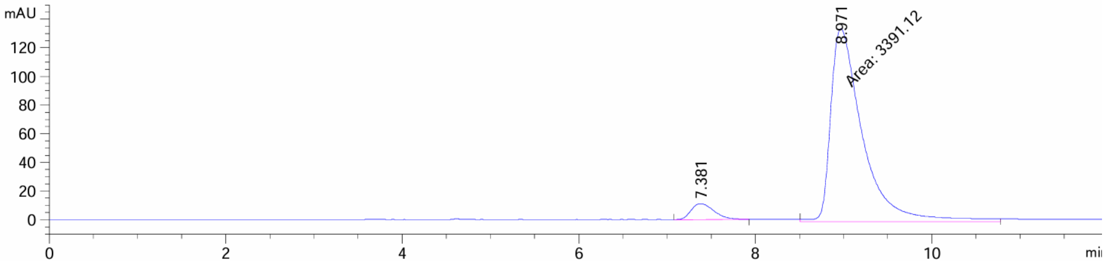

Peak #	RetTime [min]	Type	Width [min]	Area [mAU*s]	Height [mAU]	Area %
1	7.381	BB	0.2635	197.69318	11.11431	5.5086
2	8.971	MM	0.4200	3391.12109	134.56413	94.4914

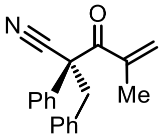

(+)-**16**

(+)-**16** was obtained as colorless oil from the desymmetric addition of **S2** using the general procedure (27.9 mg, 51% Yield). R_f = 0.7 (Hexane/EtOAc = 10:1).

HPLC analysis (Chiralpak IC-3, hexane/^{i}PrOH = 99:1, 1.0 mL/min, 254 nm; t_r (minor) = 7.29 min, t_r (major) = 8.43 min) gave the isomeric composition of the product: 98.5:1.5 e.r., $[\alpha]_D^{20}$ = +66.14 (*c* = 1.0, $CHCl_3$).

^{1}H NMR (400 MHz, $CDCl_3$) δ 7.38 – 7.28 (m, 3H), 7.23 – 7.10 (m, 5H), 6.91 – 6.80 (m, 2H), 5.87 – 5.83 (m, 1H), 5.79 (q, *J* = 1.4 Hz, 1H), 3.52 (d, *J* = 13.7 Hz, 1H), 3.31 (d, *J* = 13.7 Hz, 1H), 1.99 – 1.83 (m, 3H).

^{13}C NMR (101 MHz, $CDCl_3$) δ 192.6, 140.3, 135.3, 134.1, 130.7, 129.4, 129.3, 128.5, 127.9, 127.3, 126.0, 119.2, 57.9, 44.7, 19.1.

HRMS (ESI) calcd $C_{19}H_{18}NO^+$ $[M+H]^+$: 276.1383. Found: 276.1379.

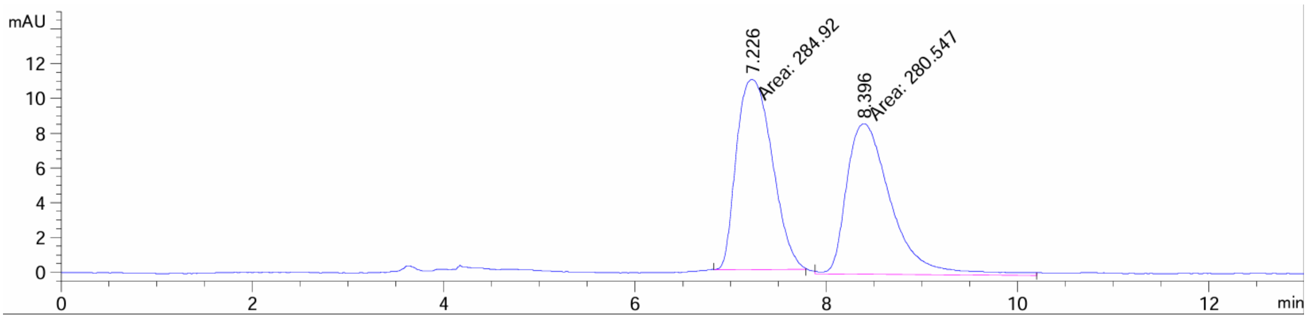

Peak #	RetTime [min]	Type	Width [min]	Area [mAU*s]	Height [mAU]	Area %
1	7.226	MM	0.4335	284.92038	10.95545	50.3867
2	8.396	MM	0.5395	280.54697	8.66767	49.6133

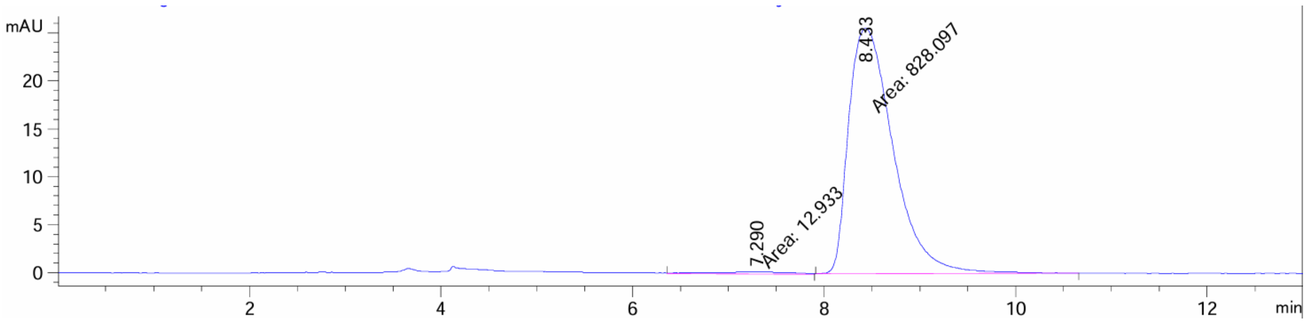

Peak #	RetTime [min]	Type	Width [min]	Area [mAU*s]	Height [mAU]	Area %
1	7.290	MM	1.0000	12.93304	2.15543e-1	1.5378
2	8.433	MM	0.5391	828.09735	25.59953	98.4622

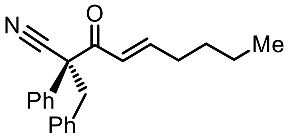

(+)-**17**

(+)-**17** was obtained as colorless oil from the desymmetric addition of **S2** using the general procedure (27.4 mg, 43% Yield). R_f = 0.7 (Hexane/EtOAc = 10:1).

HPLC analysis (Chiralpak IC-3, hexane/^{i}PrOH = 99:1, 1.0 mL/min, 254 nm; t_r (minor) = 8.07 min, t_r (major) = 9.15 min) gave the isomeric composition of the product: 90:10 e.r., $[\alpha]_D^{20}$ = +54.33 (c = 1.0, $CHCl_3$).

^{1}H NMR (600 MHz, $CDCl_3$) δ 7.40 – 7.33 (m, 3H), 7.32 – 7.28 (m, 2H), 7.22 – 7.15 (m, 3H), 7.14 – 7.06 (m, 1H), 6.96 (d, J = 6.5 Hz, 2H), 6.33 (d, J = 15.3 Hz, 1H), 3.59 (d, J = 13.9 Hz, 1H), 3.31 (d, J = 13.9 Hz, 1H), 2.14 (q, J = 7.3 Hz, 2H), 1.36 (dt, J = 15.1, 6.9 Hz, 2H), 1.25 (h, J = 7.3 Hz, 4H), 0.84 (t, J = 7.3 Hz, 3H).

^{13}C NMR (151 MHz, $CDCl_3$) δ 189.2, 152.6, 134.6, 133.5, 130.4, 129.2, 128.8, 128.0, 127.3, 127.0, 124.9, 118.8, 59.9, 42.3, 32.3, 29.8, 22.2, 13.7.

HRMS (ESI) calcd $C_{22}H_{24}NO^+$ $[M+H]^+$: 318.1852. Found: 318.1851.

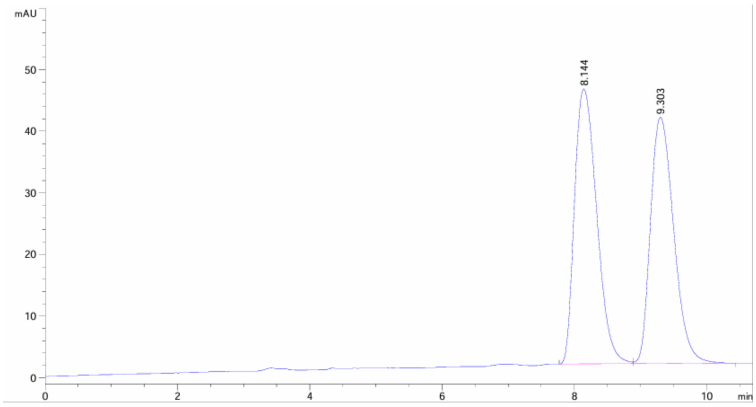

Peak #	RetTime [min]	Type	Width [min]	Area [mAU*s]	Height [mAU]	Area %
1	8.144	BV	0.3740	1040.71619	44.64369	51.6044
2	9.303	VB	0.3869	976.00549	39.96557	48.3956

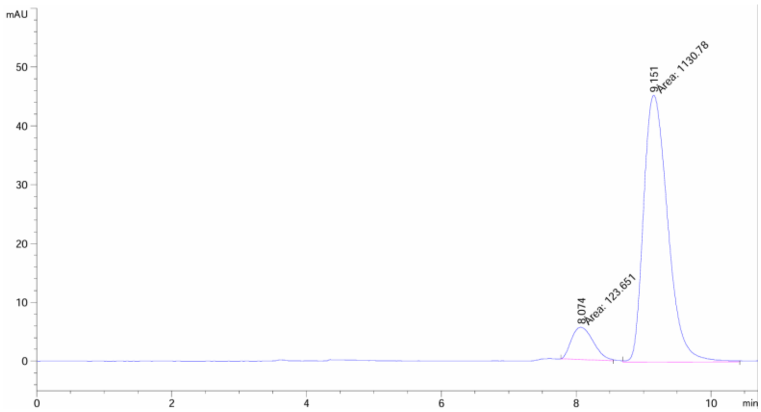

Peak #	RetTime [min]	Type	Width [min]	Area [mAU*s]	Height [mAU]	Area %
1	8.074	MM	0.3760	123.65095	5.48129	9.8571
2	9.151	MM	0.4154	1130.78149	45.36672	90.1429

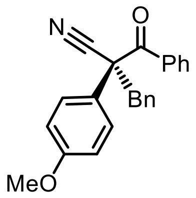

(+)-**18**

(+)-**18** was obtained as colorless oil from the desymmetric addition of **S18** using the general procedure (59.7 mg, 88% Yield). R_f = 0.6 (Hexane/EtOAc = 10:1).

HPLC analysis (Chiralpak IC-3, hexane/^{i}PrOH = 98:2, 1.0 mL/min, 254 nm; t_r (minor) = 7.97 min, t_r (major) = 9.13 min) gave the isomeric composition of the product: 93:7 e.r., $[\alpha]_D^{20}$ = +90.3 (c = 1.0, $CHCl_3$).

^{1}H NMR (400 MHz, $CDCl_3$) δ 7.83 (d, J = 7.8 Hz, 2H), 7.46 (t, J = 7.4 Hz, 1H), 7.32 (t, J = 7.7 Hz, 2H), 7.18 (d, J = 7.9 Hz, 5H), 6.92 (d, J = 6.1 Hz, 2H), 6.86 (d, J = 8.6 Hz, 2H), 3.78 (s, 3H), 3.61 (d, J = 13.6 Hz, 1H), 3.40 (d, J = 13.7 Hz, 1H).

^{13}C NMR (101 MHz, $CDCl_3$) δ 191.3, 159.7, 134.2, 134.0, 133.5, 130.7, 130.0, 128.4, 127.9, 127.7, 127.2, 126.6, 119.3, 114.7, 57.6, 55.3, 44.7.

HRMS (ESI) calcd $C_{23}H_{20}NO_2^+$ $[M+H]^+$: 342.1489. Found: 342.1489.

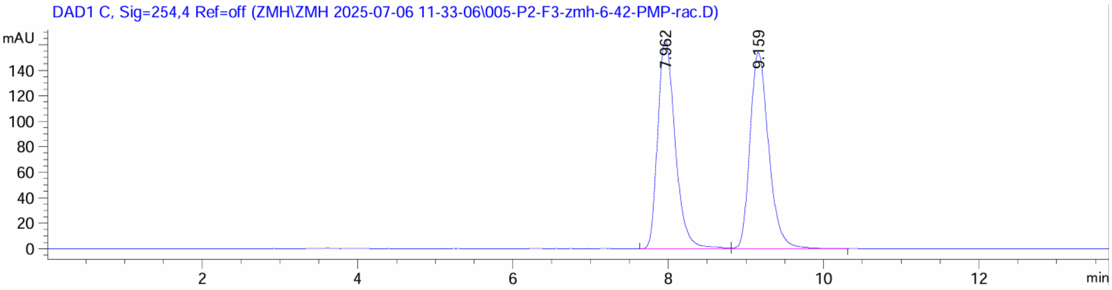

Peak #	RetTime [min]	Type	Width [min]	Area [mAU*s]	Height [mAU]	Area %
1	7.962	BV	0.2399	2553.41382	163.80637	49.8672
2	9.159	VB	0.2566	2567.01416	153.93178	50.1328

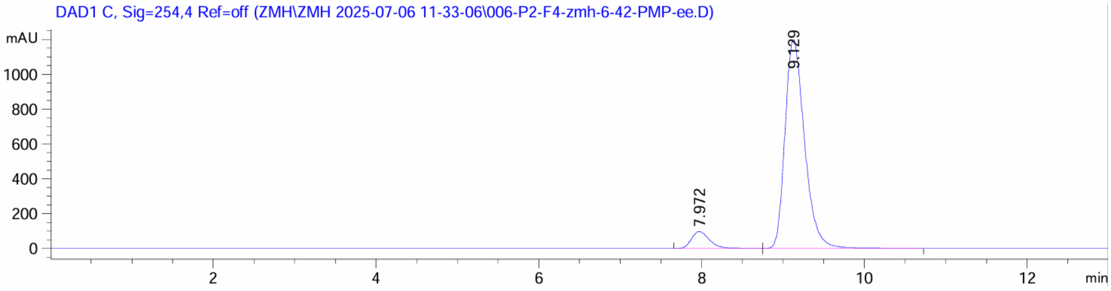

Peak #	RetTime [min]	Type	Width [min]	Area [mAU*s]	Height [mAU]	Area %
1	7.972	BV	0.2374	1486.07336	96.63835	6.9347
2	9.129	VB	0.2585	1.99433e4	1196.40295	93.0653

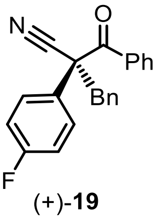

(+)-**19**

(+)-**19** was obtained as a white solid from the desymmetric addition of **S19** using the general procedure (53.4 mg, 81% Yield). R_f = 0.6 (Hexane/EtOAc = 10:1).

HPLC analysis (Chiralpak IC-3, hexane/^{i}PrOH = 99:1, 1.0 mL/min, 254 nm; t_r (minor) = 7.46 min, t_r (major) =8.79 min, gave the isomeric composition of the product: 93:7 e.r., $[\alpha]_D^{20}$ = +86.9 (c = 1.0, $CHCl_3$).

^{1}H NMR (400 MHz, $CDCl_3$) δ 7.87 – 7.76 (m, 2H), 7.53 – 7.43 (m, 1H), 7.33 (t, J = 7.9 Hz, 2H), 7.28 – 7.14 (m, 5H), 7.03 (t, J = 8.5 Hz, 2H), 6.95 – 6.86 (m, 2H), 3.63 (d, J = 13.7 Hz, 1H), 3.41 (d, J = 13.7 Hz, 1H).

^{13}C NMR (101 MHz, $CDCl_3$) δ 190.9, 162.6 (d, J = 249.3 Hz), 133.8, 133.6, 130.65 (d, J = 3.0 Hz), 130.63, 130.1, 128.5, 128.3 (d, J = 8.4 Hz) 128.0, 127.4, 119.0, 116.5 (d, J = 21.2 Hz), 57.5, 44.7.

^{19}F NMR (376 MHz, CDCl$_3$) δ -112.25.

HRMS (ESI) calcd $C_{22}H_{17}FNO^{+}$ [M+H]$^{+}$: 330.1289. Found: 330.1289.

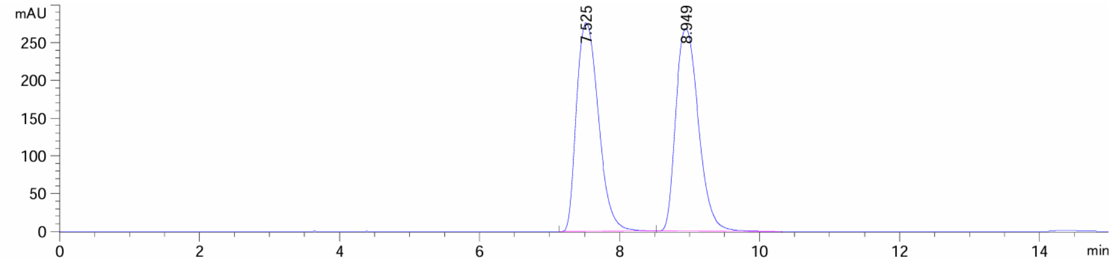

Peak #	RetTime [min]	Type	Width [min]	Area [mAU*s]	Height [mAU]	Area %
1	7.525	BB	0.3509	6036.10059	276.28497	50.0167
2	8.949	BB	0.3583	6032.06885	268.25995	49.9833

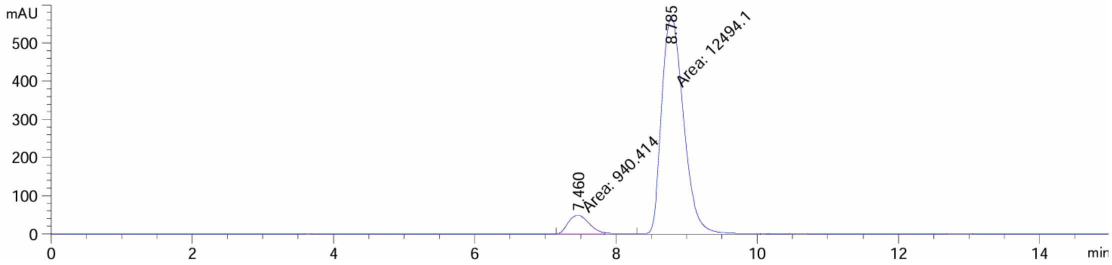

Peak #	RetTime [min]	Type	Width [min]	Area [mAU*s]	Height [mAU]	Area %
1	7.460	MM	0.3323	940.41394	47.16491	7.0000
2	8.785	MM	0.3659	1.24941e4	569.12402	93.0000

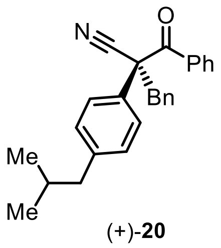

(+)-**20**

(+)-**20** was obtained as a white solid from the desymmetric addition of **S20** using the general procedure (60.7 mg, 83% Yield). R_f = 0.6 (Hexane/EtOAc = 10:1).

HPLC analysis (Chiralpak IB-3, hexane/^{i}PrOH = 99:1, 1.0 mL/min, 254 nm; t_r (minor) = 7.19 min, t_r (major) = 8.58 min) gave the isomeric composition of the product: 96:4 e.r., $[\alpha]_D^{20}$ = +106.9 (c = 1.0, CHCl$_3$).

^{1}H NMR (400 MHz, CDCl$_3$) δ 7.85 (d, *J* = 7.8 Hz, 2H), 7.46 (t, *J* = 7.4 Hz, 1H), 7.32 (t, *J* = 7.7 Hz, 2H), 7.19 – 7.09 (m, 7H), 6.89 (d, *J* = 7.3 Hz, 2H), 3.61 (d, *J* = 13.6 Hz, 1H), 3.42 (d, *J* = 13.6 Hz, 1H), 2.45 (d, *J* = 7.2 Hz, 2H), 1.88 – 1.78 (m, 1H), 0.88 – 0.85 (m, 6H).

^{13}C NMR (101 MHz, CDCl$_3$) δ 191.2, 142.4, 134.2, 134.0, 133.5, 132.0, 130.7, 130.1, 128.3, 127.8, 127.2, 126.2, 119.2, 58.0, 44.8, 44.7, 30.1, 22.2, 22.1.

HRMS (ESI) calcd $C_{26}H_{26}NO^{+}$ $[M+H]^{+}$: 368.2009. Found: 368.2009.

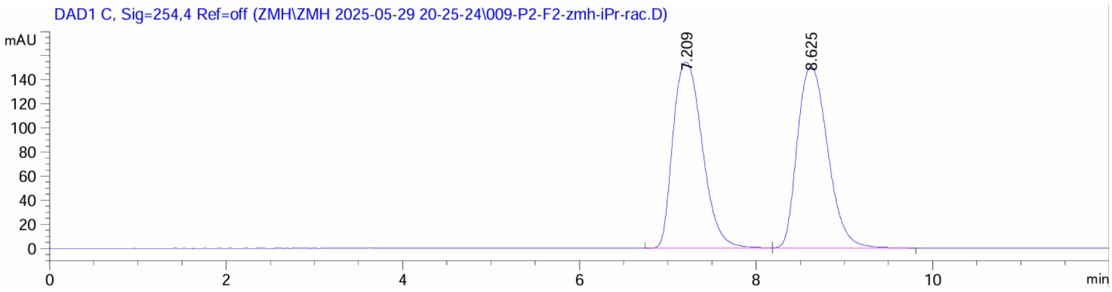

Peak #	RetTime [min]	Type	Width [min]	Area [mAU*s]	Height [mAU]	Area %
1	7.209	BV	0.3704	3571.77710	154.12469	49.7420
2	8.625	VB	0.3813	3608.83081	150.72505	50.2580

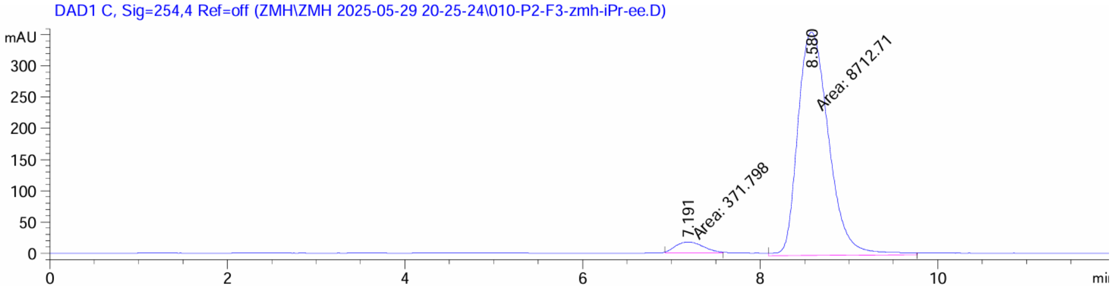

Peak #	RetTime [min]	Type	Width [min]	Area [mAU*s]	Height [mAU]	Area %
1	7.191	MM	0.3609	371.79849	17.17165	4.0927
2	8.580	MM	0.4052	8712.71191	358.33527	95.9073

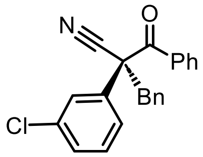

(+)-**21**

(+)-**21** was obtained as colorless oil from the desymmetric addition of **S21** using the general procedure (50.6 mg, 73% Yield). R_f = 0.5 (Hexane/EtOAc = 10:1).

HPLC analysis (Chiralpak IC-3, hexane/^{i}PrOH = 99:1, 1.0 mL/min, 254 nm; t_r (minor) = 7.34 min, t_r (major) = 8.36 min) gave the isomeric composition of the product: 91:9 e.r., $[\alpha]_D^{20}$ = +73.6 (*c* = 1.0,

CHCl3).

^{1}H NMR (400 MHz, $CDCl_3$) δ 7.83 (d, *J* = 7.8 Hz, 2H), 7.51 (t, *J* = 7.4 Hz, 1H), 7.41 – 7.26 (m, 5H), 7.24 – 7.17 (m, 4H), 6.93 (d, *J* = 7.0 Hz, 2H), 3.65 (d, *J* = 13.7 Hz, 1H), 3.40 (d, *J* = 13.7 Hz, 1H).

^{13}C NMR (101 MHz, $CDCl_3$) δ 190.5, 136.9, 135.5, 133.9, 133.60, 133.57, 130.7, 130.1, 129.1, 128.6, 128.1, 127.6, 126.5, 124.7, 118.7, 57.8, 44.7.

HRMS (ESI) calcd $C_{22}H_{17}ClNO^+$ $[M+H]^+$: 346.0993. Found: 346.0993.

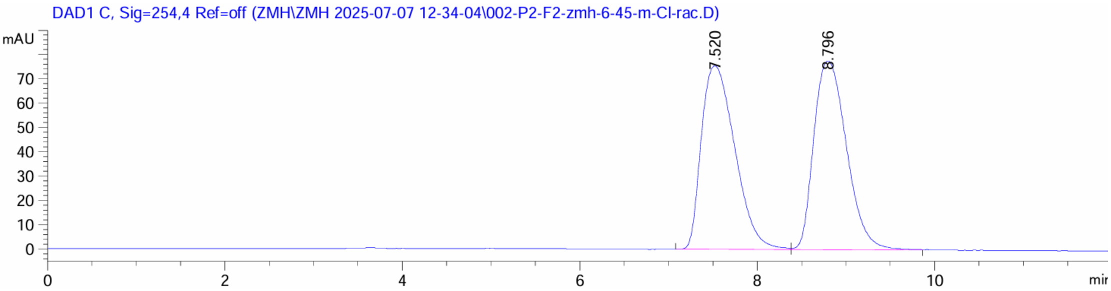

Peak #	RetTime [min]	Type	Width [min]	Area [mAU*s]	Height [mAU]	Area %
1	7.520	BV	0.4239	1980.43787	75.53928	49.9081
2	8.796	VB	0.4088	1987.73096	77.61507	50.0919

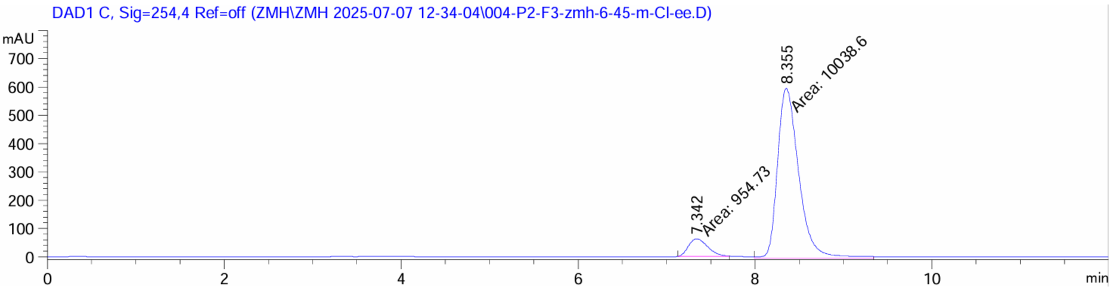

Peak #	RetTime [min]	Type	Width [min]	Area [mAU*s]	Height [mAU]	Area %
1	7.342	MM	0.2540	954.73029	62.65018	8.6846
2	8.355	MM	0.2790	1.00386e4	599.68225	91.3154

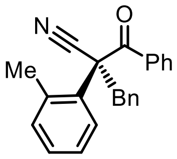

(+)-**22**

(+)-**22** was obtained as colorless oil from the desymmetric addition of **S22** using the general procedure (52.1 mg, 80% Yield). R_f = 0.6 (Hexane/EtOAc = 10:1).

HPLC analysis (Chiralpak IC-3, hexane/^{i}PrOH = 99:1, 1.0 mL/min, 254 nm; t_r (minor) = 11.24 min,

t_r (major) = 15.03 min) gave the isomeric composition of the product: 91:9 e.r., $[\alpha]_D^{20}$ = +127.1 (*c* = 2.0, $CHCl_3$).

^{1}H NMR (500 MHz, $CDCl_3$) δ 7.86 (d, *J* = 7.6 Hz, 2H), 7.47 (t, *J* = 7.4 Hz, 1H), 7.30 (t, *J* = 7.9 Hz, 2H), 7.23 – 7.05 (m, 7H), 6.85 (d, *J* = 7.0 Hz, 2H), 3.75 (d, *J* = 13.7 Hz, 1H), 3.50 (d, *J* = 13.7 Hz, 1H), 2.12 (s, 3H).

^{13}C NMR (126 MHz, $CDCl_3$) δ 191.7, 135.4, 134.1, 133.8, 133.7, 133.2, 132.9, 130.7, 130.1, 129.1, 128.7, 128.4, 127.9, 127.3, 126.8, 119.2, 58.4, 42.2, 20.4.

HRMS (ESI) calcd $C_{23}H_{20}NO^+$ $[M+H]^+$: 326.1539. Found: 326.1538.

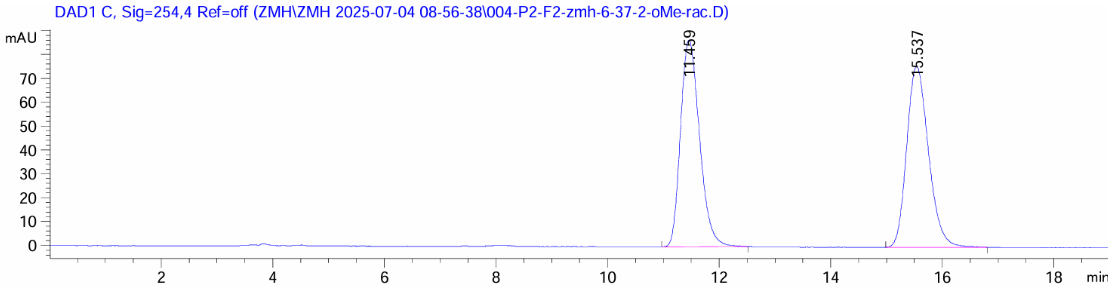

Peak #	RetTime [min]	Type	Width [min]	Area [mAU*s]	Height [mAU]	Area %
1	11.459	BB	0.3704	2054.38940	86.71217	50.0024
2	15.537	BB	0.4218	2054.19189	75.89300	49.9976

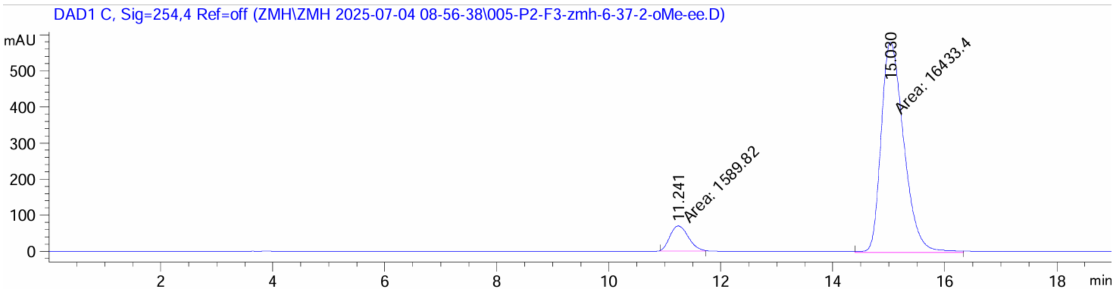

Peak #	RetTime [min]	Type	Width [min]	Area [mAU*s]	Height [mAU]	Area %
1	11.241	MM	0.3816	1589.81750	69.42757	8.8210
2	15.030	MM	0.4702	1.64334e4	582.52386	91.1790

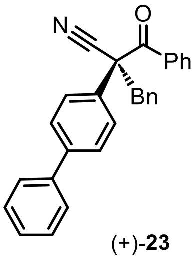

(+)-**23**

(+)-**23** was obtained as colorless oil from the desymmetric addition of **S23** using the general procedure (44.7 mg, 58% Yield). R_f = 0.7 (Hexane/EtOAc = 10:1).

HPLC analysis (Chiralpak IC-3, hexane/^{i}PrOH = 99:1, 1.0 mL/min, 254 nm; t_r (minor) = 9.90 min, t_r (major) = 11.50 min) gave the isomeric composition of the product: 91.5:8.5 e.r., $[\alpha]_D^{20}$ = +87.0 (*c* = 1.0, $CHCl_3$).

^{1}H NMR (400 MHz, $CDCl_3$) δ 7.88 (d, *J* = 8.1 Hz, 2H), 7.60 – 7.57 (m, 4H), 7.53 – 7.41 (m, 3H), 7.40 – 7.32 (m, 5H), 7.24 – 7.14 (m, 3H), 6.96 (d, *J* = 6.5 Hz, 2H), 3.69 (d, *J* = 13.7 Hz, 1H), 3.46 (d, *J* = 13.7 Hz, 1H).

^{13}C NMR (101 MHz, $CDCl_3$) δ 191.1, 141.5, 139.6, 134.1, 133.9, 133.73, 133.69, 130.8, 130.1, 128.9, 128.5, 128.00, 127.98, 127.9, 127.4, 127.0, 126.9, 119.2, 58.1, 44.8.

HRMS (ESI) calcd $C_{28}H_{22}NO^+$ $[M+H]^+$: 388.1696. Found: 388.1696.

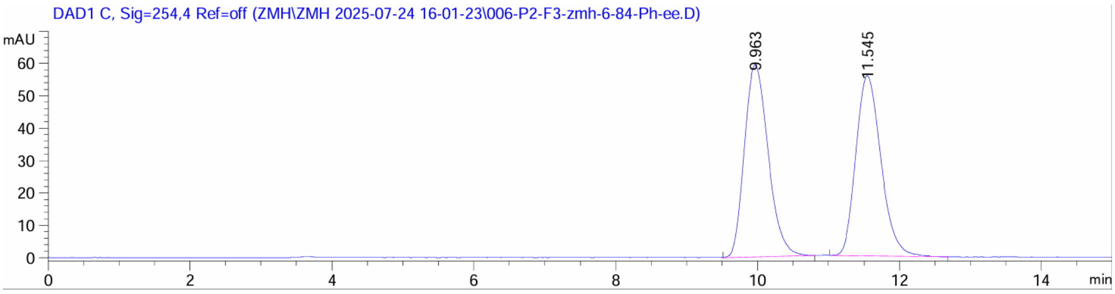

Peak #	RetTime [min]	Type	Width [min]	Area [mAU*s]	Height [mAU]	Area %
1	9.963	BB	0.3609	1383.15186	59.13395	50.2264
2	11.545	BB	0.3855	1370.68506	55.62195	49.7736

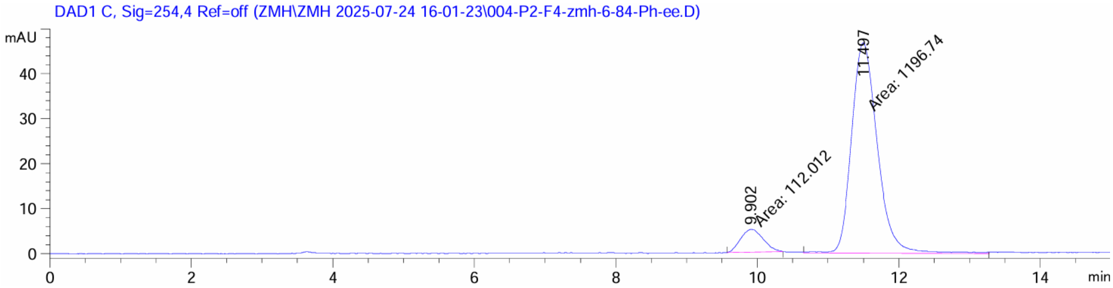

Peak #	RetTime [min]	Type	Width [min]	Area [mAU*s]	Height [mAU]	Area %
1	9.902	MM	0.3718	112.01164	5.02057	8.5587
2	11.497	MM	0.4251	1196.73596	46.91587	91.4413

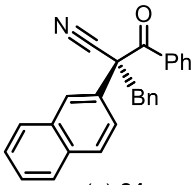

(+)-**24**

(+)-**24** was obtained as a white solid from the desymmetric addition of **S24** using the general procedure (46.3 mg, 64% Yield). R_f = 0.7 (Hexane/EtOAc = 10:1).

HPLC analysis (Chiralpak IC-3, hexane/^{i}PrOH = 99:1, 1.0 mL/min, 254 nm; t_r (minor) = 9.27 min, t_r (major) = 10.94 min) gave the isomeric composition of the product: 93:7 e.r., $[\alpha]_D^{20}$ = +107.8 (c = 1.0, $CHCl_3$).

^{1}H NMR (400 MHz, $CDCl_3$) δ 7.91 – 7.75 (m, 6H), 7.57 – 7.49 (m, 2H), 7.48 – 7.40 (m, 1H), 7.37 – 7.35 (m, 1H), 7.30 (t, J = 7.9 Hz, 2H), 7.21 – 7.08 (m, 3H), 6.94 – 6.87 (m, 2H), 3.74 (d, J = 13.7 Hz, 1H), 3.52 (d, J = 13.7 Hz, 1H).

^{13}C NMR (101 MHz, $CDCl_3$) δ 191.1, 134.1, 134.0, 133.7, 133.2, 132.9, 132.1, 130.7, 130.1, 129.6, 128.5, 128.3, 128.0, 127.7, 127.4, 127.04, 126.95, 126.2, 123.3, 119.2, 58.5, 44.7.

HRMS (ESI) calcd $C_{26}H_{20}NO^+$ $[M+H]^+$: 362.1539. Found: 362.1539.

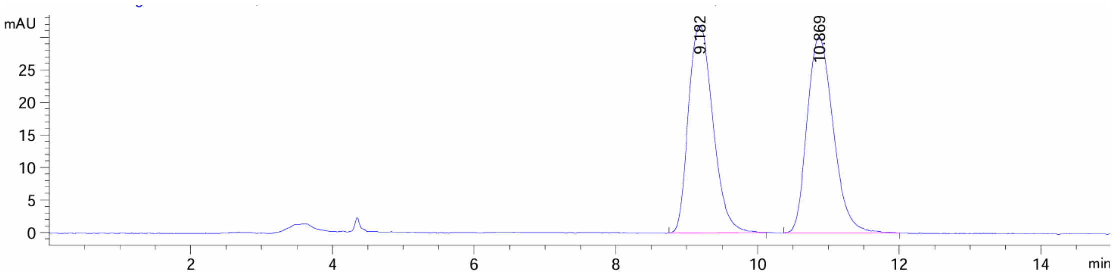

Peak #	RetTime [min]	Type	Width [min]	Area [mAU*s]	Height [mAU]	Area %
1	9.182	BB	0.3862	765.91577	31.88715	49.7127
2	10.869	BB	0.4061	774.76996	30.11746	50.2873

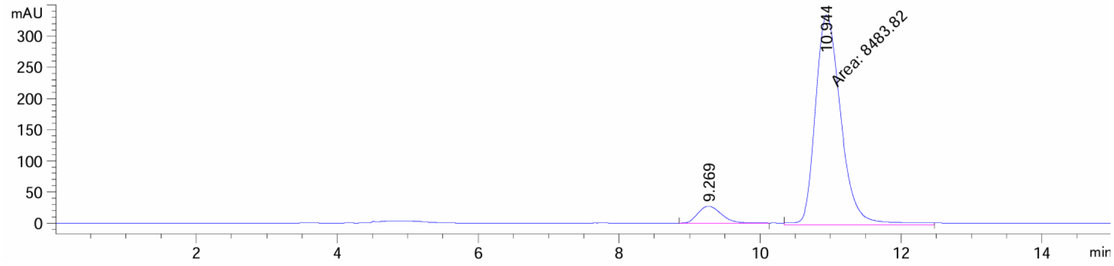

Peak #	RetTime [min]	Type	Width [min]	Area [mAU*s]	Height [mAU]	Area %
1	9.269	BB	0.3590	620.64246	27.11931	6.8169
2	10.944	MM	0.4202	8483.82227	336.51541	93.1831

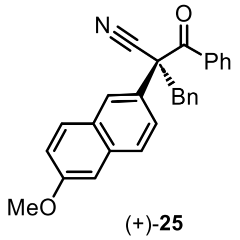

(+)-**25**

(+)-**25** was obtained as a white solid from the desymmetric addition of **S25** using the general procedure (60.8 mg, 78% Yield). R_f = 0.7 (Hexane/EtOAc = 5:1).

HPLC analysis (Chiralpak IC-3, hexane/^{i}PrOH = 97:3, 1.0 mL/min, 254 nm; t_r (minor) = 9.49 min, t_r (major) =10.87 min) gave the isomeric composition of the product: 95:5 e.r., $[\alpha]_D^{20}$ = +114.8 (*c* = 1.0, $CHCl_3$).

^{1}H NMR (400 MHz, $CDCl_3$) δ 7.90 – 7.83 (m, 2H), 7.76 – 7.63 (m, 3H), 7.48 – 7.40 (m, 1H), 7.35 – 7.26 (m, 3H), 7.21 – 7.08 (m, 5H), 6.93 – 6.87 (m, 2H), 3.92 (s, 3H), 3.71 (d, *J* = 13.7 Hz, 1H), 3.50 (d, *J* = 13.7 Hz, 1H).

^{13}C NMR (101 MHz, $CDCl_3$) δ 191.3, 158.5, 134.20, 134.16, 134.0, 133.6, 130.8, 130.1, 129.73, 129.69, 128.7, 128.4, 128.3, 127.9, 127.3, 125.9, 123.8, 119.9, 119.3, 105.5, 58.3, 55.4, 44.7.

HRMS (ESI) calcd $C_{27}H_{22}NO_2^+$ $[M+H]^+$: 392.1645. Found: 392.1641.

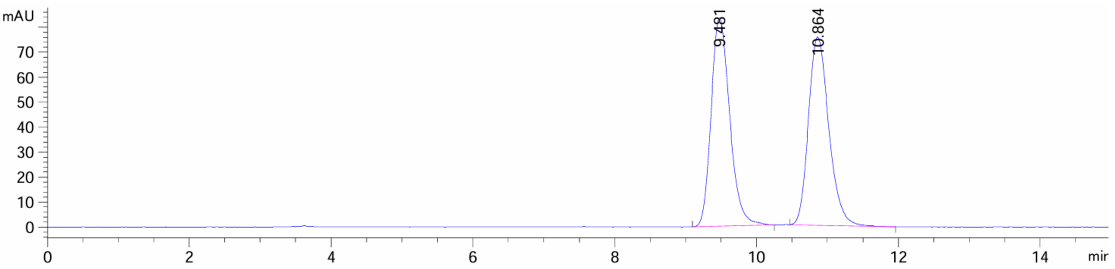

Peak #	RetTime [min]	Type	Width [min]	Area [mAU*s]	Height [mAU]	Area %
1	9.481	BB	0.2830	1522.12720	83.37684	50.3189
2	10.864	BB	0.3069	1502.83289	75.29619	49.6811

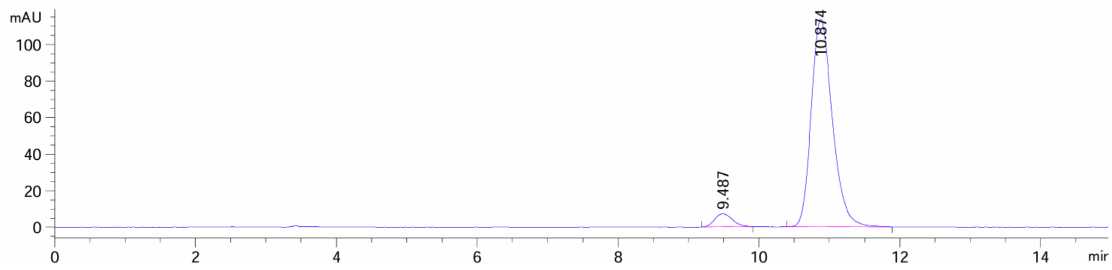

Peak #	RetTime [min]	Type	Width [min]	Area [mAU*s]	Height [mAU]	Area %
1	9.487	BB	0.2772	125.72468	7.14872	5.0469
2	10.874	BB	0.3212	2365.38208	113.45065	94.9531

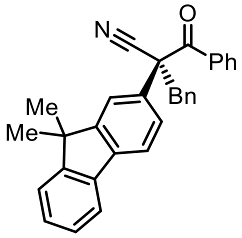

(+)-**26**

(+)-**26** was obtained as a white solid from the desymmetric addition of **S26** using the general procedure (59.2 mg, 69% Yield). R_f = 0.7 (Hexane/EtOAc = 10:1).

HPLC analysis (Chiralpak IC-3, hexane/^{i}PrOH = 99:1, 0.8 mL/min, 254 nm; t_r (minor) = 8.37 min, t_r (major) = 9.22 min) gave the isomeric composition of the product: 89:11 e.r., $[\alpha]_D^{20}$ = +136.6 (c = 1.0, $CHCl_3$).

^{1}H NMR (400 MHz, $CDCl_3$) δ 7.91 – 7.83 (m, 2H), 7.70 – 7.65 (m, 2H), 7.50 – 7.39 (m, 2H), 7.38 – 7.29 (m, 4H), 7.24 – 7.08 (m, 5H), 6.90 – 6.84 (m, 2H), 3.65 (d, J = 13.6 Hz, 1H), 3.49 (d, J = 13.7 Hz, 1H), 1.48 (s, 3H), 1.25 (s, 3H).

^{13}C NMR (101 MHz, $CDCl_3$) δ 191.4, 154.9, 153.8, 139.7, 139.0, 134.1, 134.0, 133.6, 133.5, 130.7, 130.1, 128.4, 127.9, 127.3, 127.2, 125.1, 122.7, 121.1, 120.8, 120.3, 119.4, 58.4, 47.1, 44.9, 26.9, 26.7.

HRMS (ESI) calcd $C_{31}H_{26}NO^+$ $[M+H]^+$: 428.2009. Found: 428.2009.

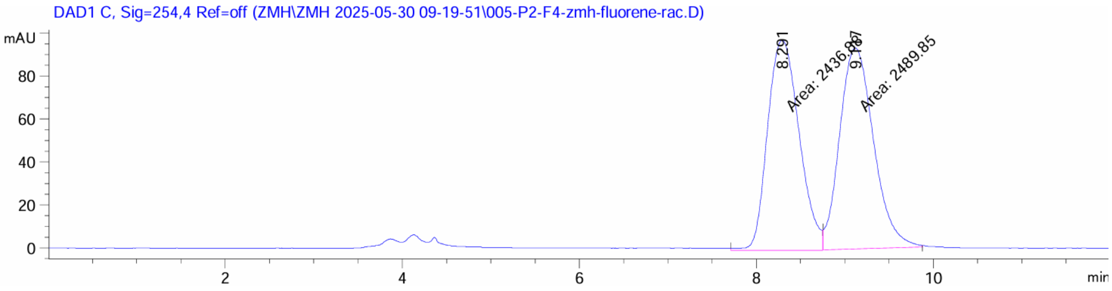

Peak #	RetTime [min]	Type	Width [min]	Area [mAU*s]	Height [mAU]	Area %
1	8.291	MM	0.4148	2436.87964	97.91459	49.4624
2	9.117	MM	0.4439	2489.84692	93.48583	50.5376

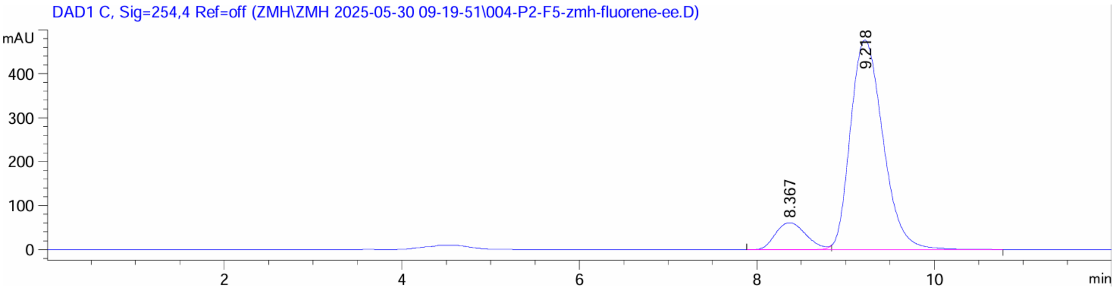

Peak #	RetTime [min]	Type	Width [min]	Area [mAU*s]	Height [mAU]	Area %
1	8.367	BV E	0.3768	1455.47144	61.35563	10.6930
2	9.218	VB R	0.4014	1.21560e4	476.90298	89.3070

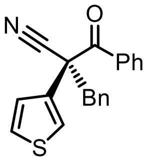

(+)-**27**

(+)-**27** was obtained as a white solid from the desymmetric addition of **S27** using the general procedure (52.1 mg, 82% Yield). R_f = 0.6 (Hexane/EtOAc = 10:1).

HPLC analysis (Chiralpak IC-3, hexane/^{i}PrOH = 99:1, 1.0 mL/min, 254 nm; t_r (minor) = 8.88 min, t_r (major) = 10.66 min) gave the isomeric composition of the product: 92:8 e.r., $[\alpha]_D^{20}$ = +112.4 (c = 1.0, $CHCl_3$).

^{1}H NMR (400 MHz, $CDCl_3$) δ 7.87 (d, J = 7.3 Hz, 2H), 7.54 – 7.46 (m, 1H), 7.40 – 7.32 (m, 3H), 7.25 – 7.16 (m, 4H), 7.01 – 6.92 (m, 3H), 3.60 (d, J = 13.6 Hz, 1H), 3.45 (d, J = 13.6 Hz, 1H).

^{13}C NMR (101 MHz, $CDCl_3$) δ 191.0, 135.3, 134.18, 134.16, 133.7, 130.6, 129.8, 128.4, 128.03, 128.01, 127.4, 125.4, 123.5, 119.5, 55.0, 44.1.

HRMS (ESI) calcd $C_{20}H_{16}NOS^+$ $[M+H]^+$: 318.0947. Found: 318.0947.

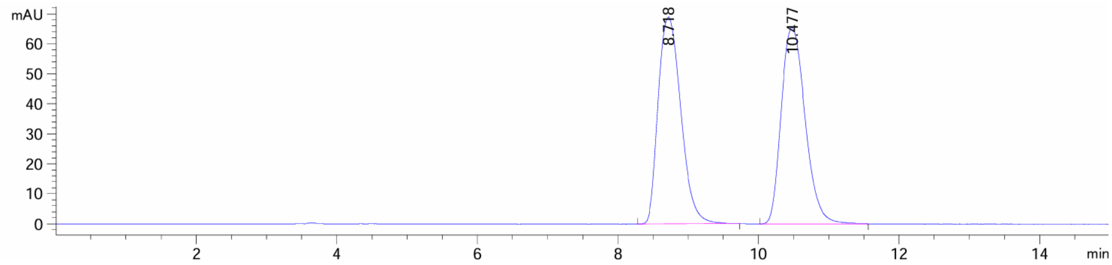

Peak #	RetTime [min]	Type	Width [min]	Area [mAU*s]	Height [mAU]	Area %
1	8.718	BB	0.3577	1547.52917	68.99459	49.9942
2	10.477	BB	0.3740	1547.88635	65.42670	50.0058

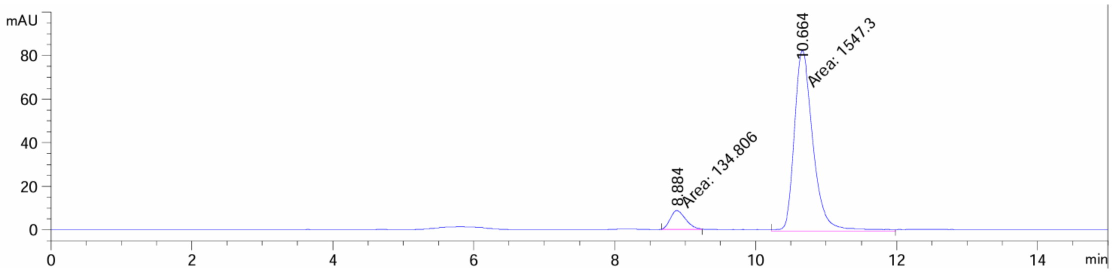

Peak #	RetTime [min]	Type	Width [min]	Area [mAU*s]	Height [mAU]	Area %
1	8.884	MM	0.2582	134.80588	8.70258	8.0141
2	10.664	MM	0.3108	1547.30481	82.98016	91.9859

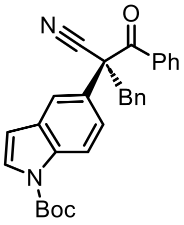

(+)-**28**

(+)-**28** was obtained as a white solid from the desymmetric addition of **S28** using the general procedure (55.3 mg, 61% Yield). R_f = 0.7 (Hexane/EtOAc = 3:1).

HPLC analysis (Chiralpak IC-3, hexane/^{i}PrOH = 97:3, 1.0 mL/min, 254 nm; t_r (minor) = 7.73 min, t_r (major) = 8.73 min) gave the isomeric composition of the product: 93:7 e.r., $[\alpha]_D^{20}$ = +51.6 (c = 1.0, $CHCl_3$).

^{1}H NMR (400 MHz, $CDCl_3$) δ 8.12 (d, J = 8.7 Hz, 1H), 7.88 – 7.78 (m, 2H), 7.62 (d, J = 3.7 Hz, 1H), 7.50 (d, J = 2.1 Hz, 1H), 7.48 – 7.41 (m, 1H), 7.29 (t, J = 7.8 Hz, 2H), 7.24 – 7.10 (m, 4H), 6.93 – 6.82 (m, 2H), 6.52 (d, J = 3.8 Hz, 1H), 3.68 (d, J = 13.7 Hz, 1H), 3.49 (d, J = 13.7 Hz, 1H), 1.66 (s, 9H).

^{13}C NMR (101 MHz, CDCl$_3$) δ 191.3, 149.4, 134.9, 134.3, 134.0, 133.5, 131.2, 130.8, 130.2, 129.0, 128.4, 127.9, 127.3, 127.2, 122.2, 119.4, 119.2, 116.1, 107.2, 84.2, 58.3, 45.0, 28.1.

HRMS (ESI) calcd $C_{29}H_{27}N_2O_3^+$ [M+H]$^+$: 451.2016. Found: 451.2012.

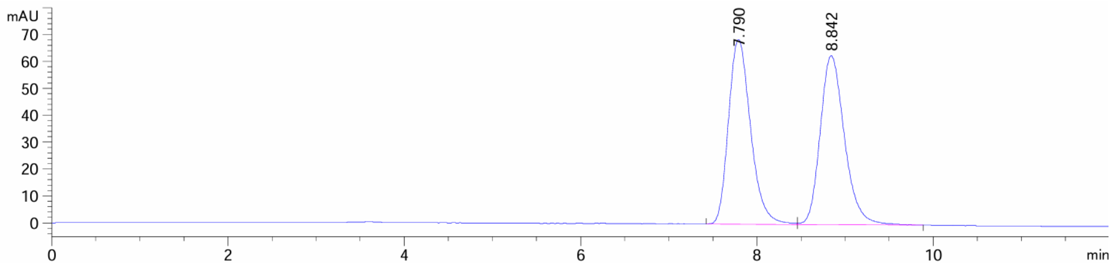

Peak #	RetTime [min]	Type	Width [min]	Area [mAU*s]	Height [mAU]	Area %
1	7.790	BV	0.2693	1196.69202	68.67110	49.8583
2	8.842	VB	0.2954	1203.49536	62.85396	50.1417

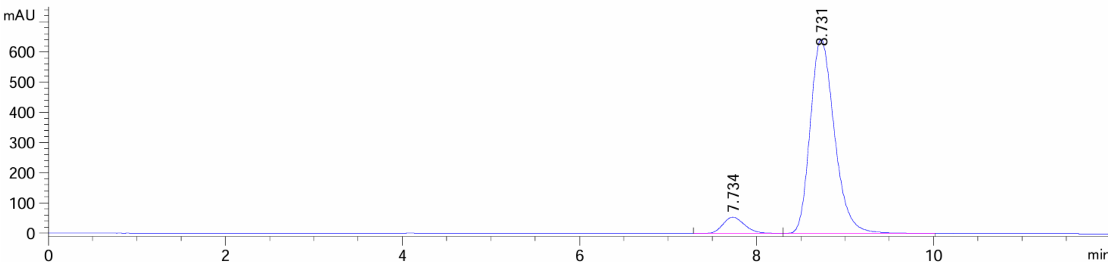

Peak #	RetTime [min]	Type	Width [min]	Area [mAU*s]	Height [mAU]	Area %
1	7.734	BV	0.2674	930.12482	53.88811	7.0701
2	8.731	VB	0.2937	1.22257e4	643.38275	92.9299

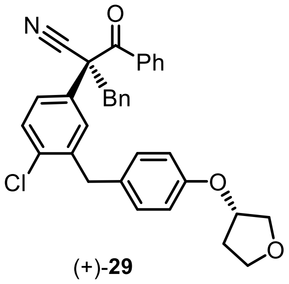

(+)-**29**

(+)-**29** was obtained as a white solid from the desymmetric addition of **S29** using the general procedure (66.7 mg, 64% Yield). R$_f$ = 0.5 (Hexane/EtOAc = 5:1).

HPLC analysis (Chiralpak IB-3, hexane/^{i}PrOH = 97/3, 1.0 mL/min, 254 nm; t$_r$ (minor) = 17.24 min, t$_r$ (major) = 19.24 min) gave the isomeric composition of the product: 87:13 d.r., $[\alpha]_D^{20}$ = +74.2 (*c* = 1.0, CHCl$_3$).

^{1}H NMR (500 MHz, CDCl$_3$) δ 7.81 (d, *J* = 7.1 Hz, 2H), 7.50 (t, *J* = 7.4 Hz, 1H), 7.38 – 7.29 (m, 3H), 7.26 – 7.20 (m, 1H), 7.19 – 7.14 (m, 2H), 7.09 (d, *J* = 2.5 Hz, 1H), 7.06 – 7.03 (m, 1H), 6.89 (d, *J* = 7.2 Hz, 2H), 6.84 (d, *J* = 8.6 Hz, 2H), 6.71 (d, *J* = 8.7 Hz, 2H), 4.90 – 4.87 (m, 1H), 4.02 – 3.93 (m, 5H), 3.92 – 3.86 (m, 1H), 3.60 (d, *J* = 13.7 Hz, 1H), 3.39 (d, *J* = 13.7 Hz, 1H), 2.25 – 2.07 (m, 2H).

^{13}C NMR (126 MHz, CDCl$_3$) δ 190.6, 155.9, 140.3, 134.9, 133.74, 133.70, 133.6, 133.5, 130.9, 130.64, 130.63, 130.1, 129.6, 129.3, 128.5, 128.1, 127.4, 125.6, 118.8, 115.4, 73.1, 67.2, 57.5, 44.4, 38.4, 33.0.

HRMS (ESI) calcd $C_{33}H_{29}ClNO_3^+$ $[M+H]^+$: 522.1830. Found: 522.1830.

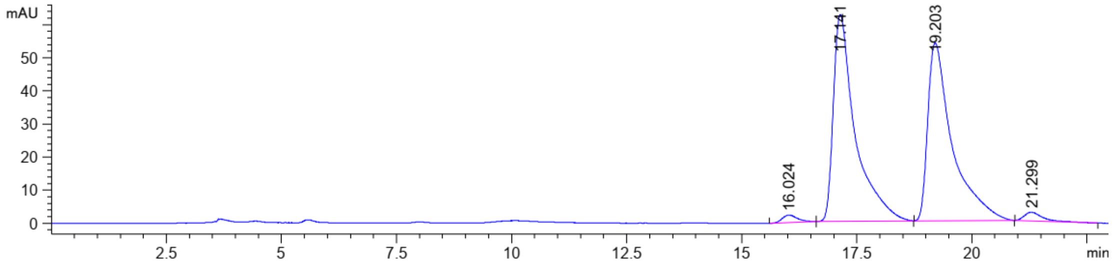

Peak #	RetTime [min]	Type	Width [min]	Area [mAU*s]	Height [mAU]	Area %
1	16.024	BB	0.3355	51.16005	2.26551	1.2257
2	17.141	BB	0.4775	2062.20190	62.41058	49.4060
3	19.203	BB	0.5294	1987.51465	53.74415	47.6166
4	21.299	BBA	0.3955	73.11516	2.62024	1.7517

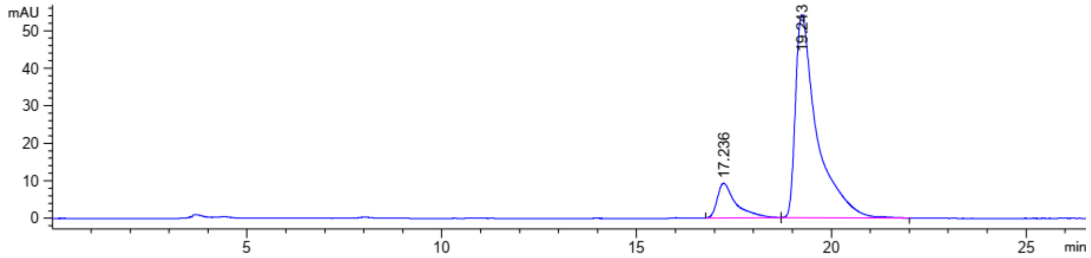

Peak #	RetTime [min]	Type	Width [min]	Area [mAU*s]	Height [mAU]	Area %
1	17.236	BB	0.4694	304.26599	9.21048	12.8152
2	19.243	BB	0.5509	2069.98486	54.03666	87.1848

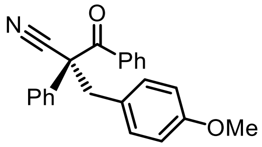

(+)-**30**

(+)-**30** was obtained as a white solid from the desymmetric addition of **S30** using the general procedure

(52.8 mg, 77% Yield). R_f = 0.6 (Hexane/EtOAc = 5:1).

HPLC analysis (Chiralpak IC-3, hexane/^{i}PrOH = 95:5, 1.0 mL/min, 254 nm; t_r (minor) =8.28 min, t_r (major) = 10.43 min) gave the isomeric composition of the product: 96:4 e.r., $[\alpha]_D^{20}$ = +84.7 (c = 1.0, $CHCl_3$).

^{1}H NMR (400 MHz, $CDCl_3$) δ 7.83 (d, J = 7.4 Hz, 2H), 7.51 – 7.43 (m, 1H), 7.40 – 7.26 (m, 7H), 6.82 (d, J = 8.7 Hz, 2H), 6.71 (d, J = 8.7 Hz, 2H), 3.75 (s, 3H), 3.61 (d, J = 13.8 Hz, 1H), 3.36 (d, J = 13.8 Hz, 1H).

^{13}C NMR (101 MHz, $CDCl_3$) δ 191.2, 158.9, 134.9, 134.0, 133.6, 131.8, 130.1, 129.4, 128.7, 128.4, 126.4, 126.0, 119.2, 113.4, 58.5, 55.1, 44.1.

HRMS (ESI) calcd $C_{23}H_{19}NNaO_2^+$ $[M+Na]^+$: 364.1308. Found: 364.1308.

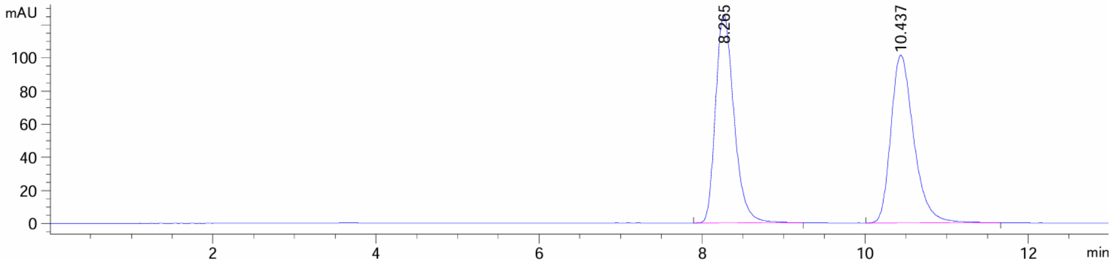

Peak #	RetTime [min]	Type	Width [min]	Area [mAU*s]	Height [mAU]	Area %
1	8.265	BB	0.2432	1972.85461	125.65765	49.9677
2	10.437	BB	0.2995	1975.40845	101.28268	50.0323

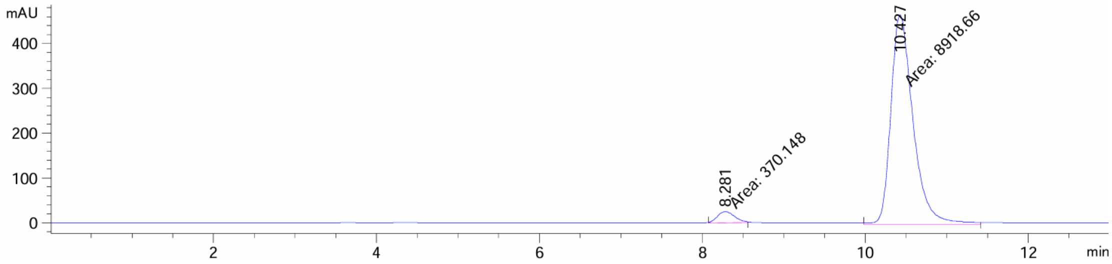

Peak #	RetTime [min]	Type	Width [min]	Area [mAU*s]	Height [mAU]	Area %
1	8.281	MM	0.2451	370.14801	25.17364	3.9849
2	10.427	MM	0.3176	8918.65625	468.06372	96.0151

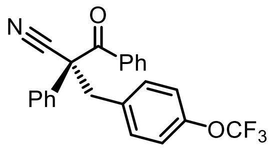

(+)-**31**

(+)-**31** was obtained as a white solid from the desymmetric addition of **S31** using the general procedure (56.3 mg, 71% Yield). R_f = 0.6 (Hexane/EtOAc = 10:1).

HPLC analysis (Chiralpak IC-3, hexane/^{i}PrOH = 98:2, 1.0 mL/min, 254 nm; t_r (minor) = 4.90 min, t_r (major) = 5.41 min) gave the isomeric composition of the product: 96:4 e.r., $[\alpha]_D^{20}$ = +102.9 (c = 1.0, $CHCl_3$).

^{1}H NMR (500 MHz, $CDCl_3$) δ 7.85 – 7.83 (m, 2H), 7.52 – 7.45 (m, 1H), 7.40 – 7.31 (m, 5H), 7.30 – 7.26 (m, 2H), 7.03 – 7.01 (m, 2H), 6.96 – 6.89 (m, 2H), 3.64 (d, J = 13.8 Hz, 1H), 3.42 (d, J = 13.8 Hz, 1H).

^{13}C NMR (126 MHz, $CDCl_3$) δ 190.7, 148.64 (q, J = 2.5 Hz), 134.5, 133.8, 133.7, 132.8, 132.1, 130.1, 129.6, 128.9, 128.5, 126.3, 120.4 (q, J = 257.0 Hz), 120.3, 118.9, 58.0, 44.0

^{19}F NMR (471 MHz, $CDCl_3$) δ -57.83.

HRMS (ESI) calcd $C_{23}H_{17}F_3NO_2^+$ [M+H]$^+$: 396.1206. Found: 396.1206.

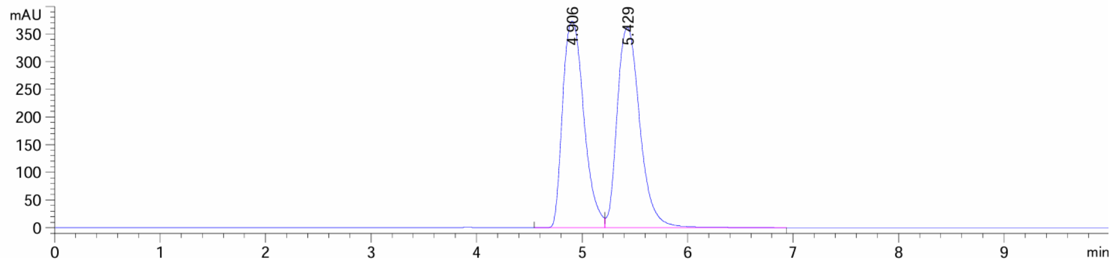

Peak #	RetTime [min]	Type	Width [min]	Area [mAU*s]	Height [mAU]	Area %
1	4.906	BV	0.2229	5217.26074	373.71796	49.0538
2	5.429	VB	0.2367	5418.52979	361.79736	50.9462

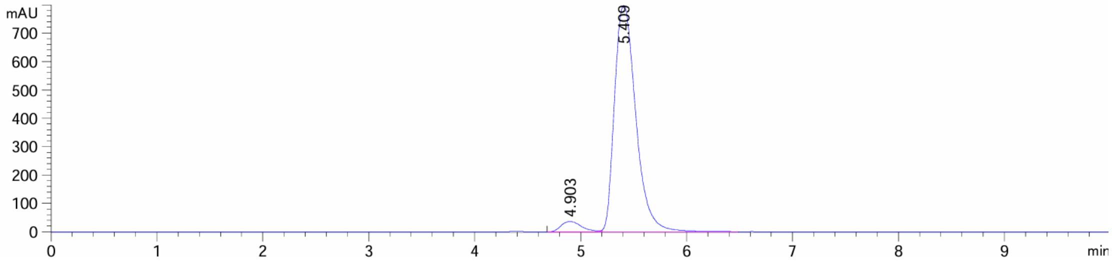

Peak #	RetTime [min]	Type	Width [min]	Area [mAU*s]	Height [mAU]	Area %
1	4.903	BV E	0.2079	473.59039	35.90120	4.0853
2	5.409	VB R	0.2186	1.11189e4	797.57635	95.9147

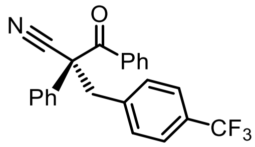

(+)-**32**

(+)-**32** was obtained as a white solid from the desymmetric addition of **S32** using the general procedure (69.2 mg, 91% Yield). R_f = 0.7 (Hexane/EtOAc = 10:1).

HPLC analysis (Chiralpak IC-3, hexane/^{i}PrOH = 99:1, 1.0 mL/min, 254 nm; t_r (minor) = 5.91 min, t_r (major) = 7.20 min) gave the isomeric composition of the product: 96:4 e.r., $[\alpha]_D^{20}$ = +122.4 (*c* = 1.0, $CHCl_3$).

^{1}H NMR (400 MHz, $CDCl_3$) δ 7.84 (d, *J* = 8.1 Hz, 2H), 7.52 – 7.46 (m, 1H), 7.43 (d, *J* = 8.1 Hz, 2H), 7.40 – 7.31 (m, 5H), 7.30 – 7.26 (m, 2H), 7.02 (d, *J* = 8.0 Hz, 2H), 3.69 (d, *J* = 13.6 Hz, 1H), 3.47 (d, *J* = 13.6 Hz, 1H).

^{13}C NMR (101 MHz, $CDCl_3$) δ 190.5, 138.2, 134.4, 133.9, 133.6, 131.1, 130.2, 129. 7, 129.6 (q, *J* = 32.3 Hz), 129.0, 128.5, 126.3, 124.8 (q, *J* = 3.8 Hz), 124.1 (q, *J* = 272.2 Hz), 118.8, 57.9, 44.5.

^{19}F NMR (376 MHz, $CDCl_3$) δ -62.54.

HRMS (ESI) calcd $C_{23}H_{17}F_3NO^+$ $[M+H]^+$: 380.1257. Found: 380.1257.

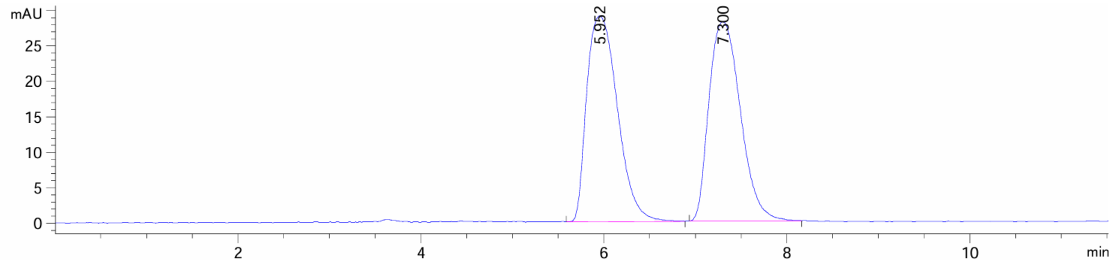

Peak #	RetTime [min]	Type	Width [min]	Area [mAU*s]	Height [mAU]	Area %
1	5.952	BB	0.3763	677.62158	29.02906	50.2813
2	7.300	BB	0.3844	670.04034	28.07964	49.7187

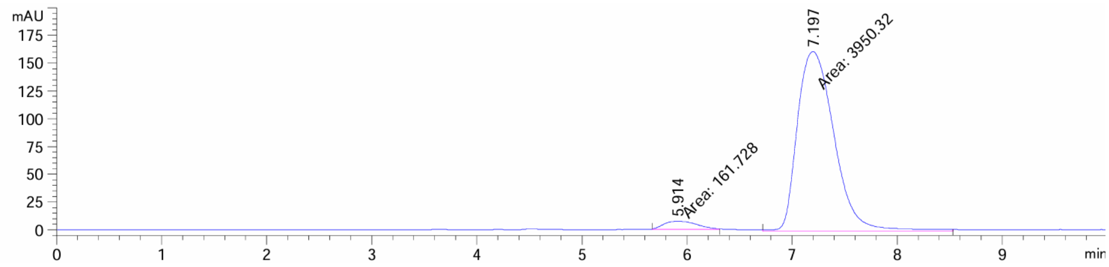

Peak #	RetTime [min]	Type	Width [min]	Area [mAU*s]	Height [mAU]	Area %
1	5.914	MM	0.3670	161.72774	7.34404	3.9330
2	7.197	MM	0.4072	3950.32129	161.67848	96.0670

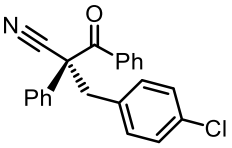

(+)-**33**

(+)-**33** was obtained as a white solid from the desymmetric addition of **S33** using the general procedure (57.1 mg, 83% Yield). R_f = 0.7 (Hexane/EtOAc = 10:1).

HPLC analysis (Chiralpak IC-3, hexane/^{i}PrOH = 99:1, 1.0 mL/min, 254 nm; t_r (minor) = 7.43 min, t_r (major) = 9.29 min) gave the isomeric composition of the product: 96:4 e.r., $[\alpha]_D^{20}$ = +107.6 (c = 1.0, $CHCl_3$).

^{1}H NMR (400 MHz, $CDCl_3$) δ 7.76 (d, J = 7.2 Hz, 2H), 7.44 – 7.36 (m, 1H), 7.33 – 7.19 (m, 7H), 7.07 (d, J = 8.4 Hz, 2H), 6.75 (d, J = 8.4 Hz, 2H), 3.53 (d, J = 13.7 Hz, 1H), 3.32 (d, J = 13.8 Hz, 1H).

^{13}C NMR (101 MHz, $CDCl_3$) δ 190.8, 134.5, 133.8, 133.7, 133.4, 132.6, 132.0, 130.1, 129.6, 128.9, 128.5, 128.1, 126.3, 118.9, 58.0, 44.1.

HRMS (ESI) calcd $C_{22}H_{17}ClNO^+$ $[M+H]^+$: 346.0993. Found: 346.0994.

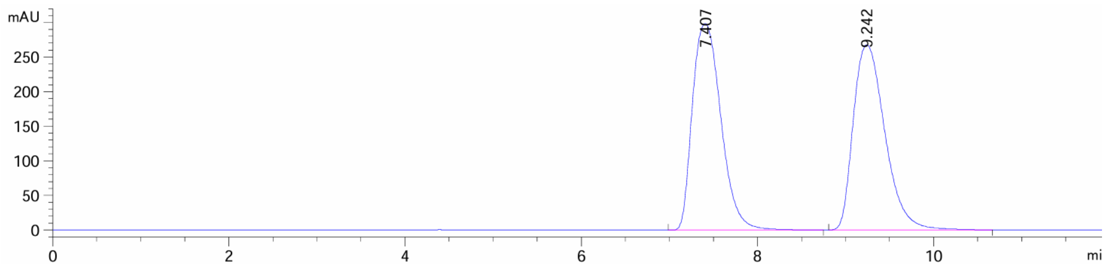

Peak #	RetTime [min]	Type	Width [min]	Area [mAU*s]	Height [mAU]	Area %
1	7.407	BB	0.3603	6708.42334	298.42984	50.1222
2	9.242	BB	0.3950	6675.72314	267.68436	49.8778

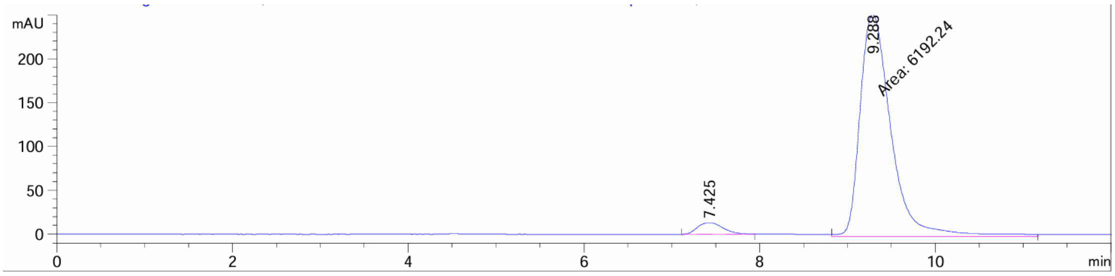

Peak #	RetTime [min]	Type	Width [min]	Area [mAU*s]	Height [mAU]	Area %
1	7.425	BB	0.3187	263.75510	13.10955	4.0854
2	9.288	MM	0.4082	6192.23584	252.79897	95.9146

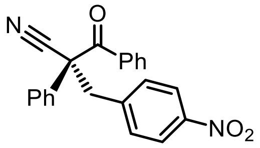

(+)-**34**

(+)-**34** was obtained as a white solid from the desymmetric addition of **S34** using the general procedure (46.4 mg, 65% Yield). R_f = 0.6 (Hexane/EtOAc = 5:1).

HPLC analysis (Chiralpak IB-3, hexane/^{i}PrOH = 90:10, 1.0 mL/min, 254 nm; t_r (minor) = 8.95 min, t_r (major) = 10.24 min) gave the isomeric composition of the product: 96:4 e.r., $[\alpha]_D^{20}$ = +72.4 (*c* = 1.0, $CHCl_3$).

^{1}H NMR (500 MHz, $CDCl_3$) δ 8.03 (d, *J* = 8.7 Hz, 2H), 7.89 – 7.80 (m, 2H), 7.51 (t, *J* = 7.4 Hz, 1H), 7.42 – 7.32 (m, 5H), 7.31 – 7.26 (m, 2H), 7.07 (d, *J* = 8.7 Hz, 2H), 3.72 (d, *J* = 13.6 Hz, 1H), 3.54 (d, *J* = 13.7 Hz, 1H).

^{13}C NMR (126 MHz, $CDCl_3$) δ 190.2, 147.4, 141.8, 134.1, 134.0, 133.4, 131.6, 130.2, 129.8, 129.2, 128.6, 126.3, 123.1, 118.6, 57.7, 44.4.

HRMS (ESI) calcd $C_{22}H_{17}N_2O_3^+$ $[M+H]^+$: 357.1234. Found: 357.1233.

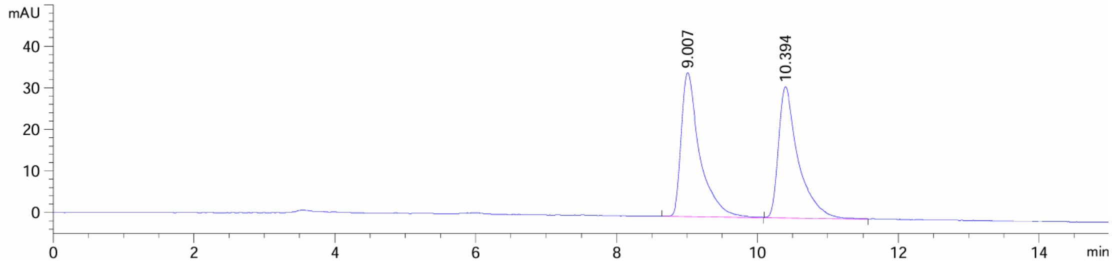

Peak #	RetTime [min]	Type	Width [min]	Area [mAU*s]	Height [mAU]	Area %
1	9.007	BB	0.2634	626.93060	34.59360	50.7681
2	10.394	BB	0.2815	607.95935	31.45413	49.2319

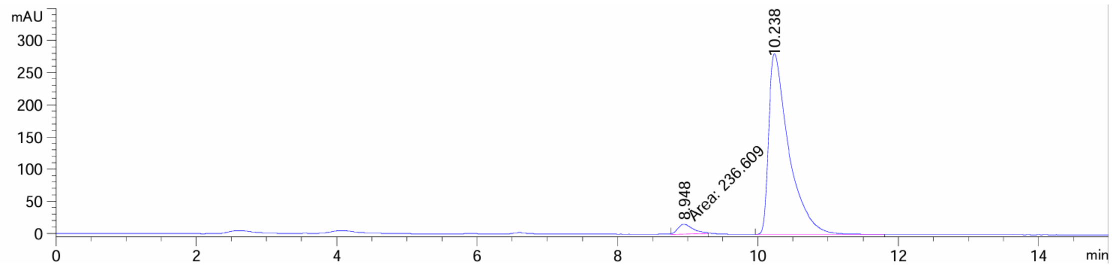

Peak #	RetTime [min]	Type	Width [min]	Area [mAU*s]	Height [mAU]	Area %
1	8.948	MM	0.2547	236.60870	15.48109	4.0542
2	10.238	BB	0.2884	5599.47314	281.12384	95.9458

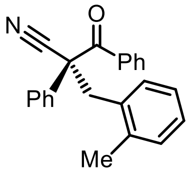

(+)-**35**

(+)-**35** was obtained as a white solid from the desymmetric addition of **S35** using the general procedure (49.7 mg, 76% Yield). R_f = 0.6 (Hexane/EtOAc = 10:1).

HPLC analysis (Chiralpak IC-3, hexane/^{i}PrOH = 99:1, 1.0 mL/min, 254 nm; t_r (minor) = 7.42 min, t_r (major) = 9.06 min) gave the isomeric composition of the product: 96:4 e.r., $[\alpha]_D^{20}$ = +102.7 (c = 1.0, $CHCl_3$).

^{1}H NMR (400 MHz, $CDCl_3$) δ 7.52 – 7.46 (m, 2H), 7.45 – 7.35 (m, 3H), 7.30 – 7.16 (m, 5H), 7.15 – 7.09 (m, 2H), 7.05 – 6.93 (m, 2H), 3.77 (d, J = 13.6 Hz, 1H), 3.39 (d, J = 13.6 Hz, 1H), 2.25 (s, 3H).

^{13}C NMR (101 MHz, $CDCl_3$) δ 195.4, 138.1, 135.8, 134.5, 134.4, 131.6, 131.5, 130.8, 129.4, 128.9, 128.1, 127.9, 127.5, 126.5, 125.0, 119.2, 61.0, 44.7, 20.4.

HRMS (ESI) calcd $C_{23}H_{20}NO^+$ $[M+H]^+$: 326.1539. Found: 326.1539.

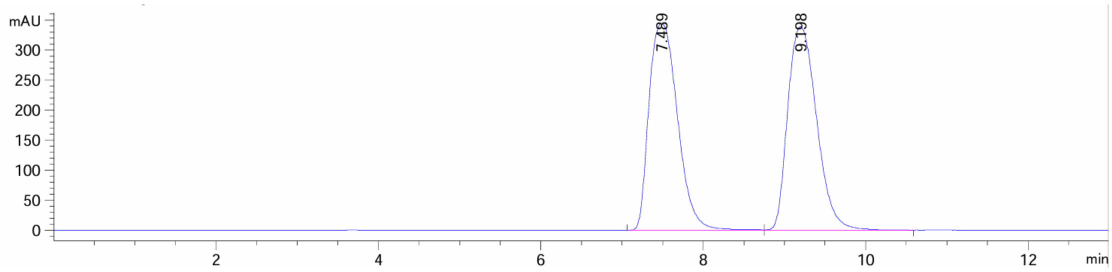

Peak #	RetTime [min]	Type	Width [min]	Area [mAU*s]	Height [mAU]	Area %
1	7.489	BV	0.3947	8416.02734	344.82632	50.0851
2	9.198	VB	0.3968	8387.44043	338.87091	49.9149

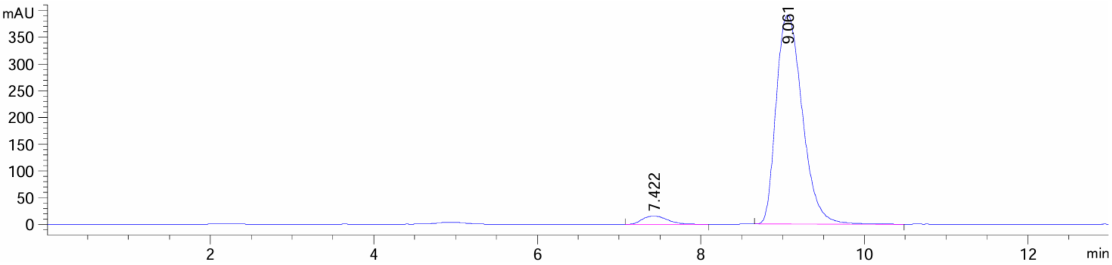

Peak #	RetTime [min]	Type	Width [min]	Area [mAU*s]	Height [mAU]	Area %
1	7.422	BB	0.3373	327.64447	15.33395	3.6414
2	9.061	BB	0.3508	8670.20801	390.80792	96.3586

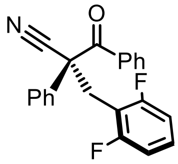

(+)-**36**

(+)-**36** was obtained as a white solid from the desymmetric addition of **S36** using the general procedure (54.3 mg, 78% Yield). R_f = 0.6 (Hexane/EtOAc = 10:1).

HPLC analysis (Chiralpak IC-3, hexane/^{i}PrOH = 99:1, 1.0 mL/min, 254 nm; t_r (minor) = 10.50 min, t_r (major) = 12.368 min) gave the isomeric composition of the product: 97:3 e.r., $[\alpha]_D^{20}$ = +50.4 (*c* = 1.0, $CHCl_3$).

^{1}H NMR (500 MHz, $CDCl_3$) δ 7.88 – 7.77 (m, 2H), 7.52 – 7.43 (m, 1H), 7.39 – 7.27 (m, 7H), 7.21 – 7.16 (m, 1H), 6.76 (t, *J* = 7.9 Hz, 2H), 3.82 (d, *J* = 14.1 Hz, 1H), 3.59 (d, *J* = 14.1 Hz, 1H).

^{13}C NMR (126 MHz, $CDCl_3$) δ 190.8, 161.9 (dd, *J* = 249.5 Hz, *J* = 8.8 Hz), 134.5, 133.7, 133.4, 130.3, 129.5 (t, *J* = 10.4 Hz), 129.4, 128.8, 128.4, 126.4, 118.6, 111.2 – 111.0 (m), 110.5 (t, *J* = 19.1 Hz), 56.6, 32.2.

^{19}F NMR (471 MHz, $CDCl_3$) δ -110.76.

HRMS (ESI) calcd $C_{22}H_{16}F_2NO^+$ $[M+H]^+$: 348.1194. Found: 348.1193.

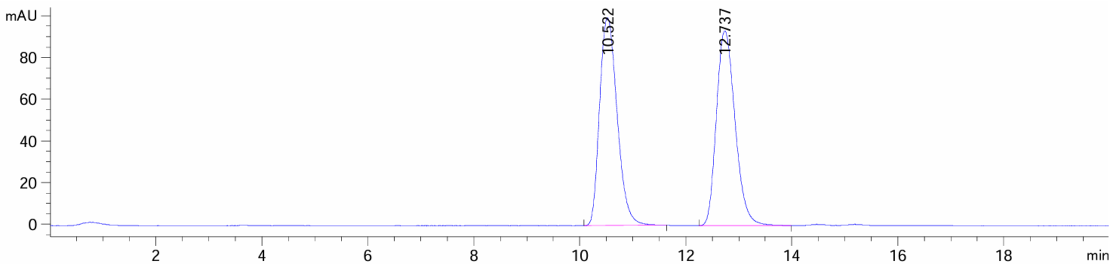

Peak #	RetTime [min]	Type	Width [min]	Area [mAU*s]	Height [mAU]	Area %
1	10.522	BB	0.3640	2288.62183	99.63811	49.9275
2	12.737	BB	0.3865	2295.27100	93.47728	50.0725

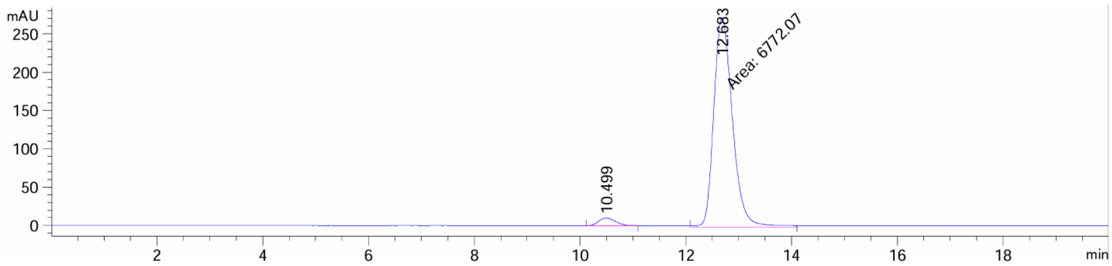

Peak #	RetTime [min]	Type	Width [min]	Area [mAU*s]	Height [mAU]	Area %
1	10.499	BB	0.3487	215.36996	9.78829	3.0822
2	12.683	MM	0.4131	6772.07422	273.19476	96.9178

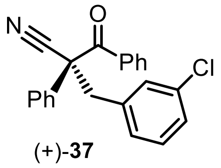

(+)-**37**

(+)-**37** was obtained as a white solid from the desymmetric addition of **S37** using the general procedure (63.2 mg, 92% Yield). R_f = 0.7 (Hexane/EtOAc = 10:1).

HPLC analysis (Chiralpak IC-3, hexane/^{i}PrOH = 99:1, 1.0 mL/min, 254 nm; t_r (minor) = 7.57 min, t_r (major) = 9.46 min) gave the isomeric composition of the product: 96:4 e.r., $[\alpha]_D^{20}$ = +36.4 (c = 1.0, $CHCl_3$).

^{1}H NMR (400 MHz, $CDCl_3$) δ 7.85 (d, J = 7.9 Hz, 2H), 7.47 (t, J = 7.4 Hz, 1H), 7.41 – 7.23 (m, 7H), 7.19 (d, J = 8.3 Hz, 1H), 7.11 (t, J = 7.8 Hz, 1H), 6.86 – 6.84 (m, 1H), 6.81 (d, J = 2.0 Hz, 1H), 3.61 (d, J = 13.7 Hz, 1H), 3.39 (d, J = 13.8 Hz, 1H).

^{13}C NMR (101 MHz, $CDCl_3$) δ 190.6, 136.1, 134.5, 133.7, 133.63, 133.60, 130.7, 130.1, 129.6, 129.1, 128.93, 128.90, 128.4, 127.6, 126.3, 118.8, 57.9, 44.3.

HRMS (ESI) calcd $C_{22}H_{17}ClNO^+$ $[M+H]^+$: 346.0993. Found: 346.0992.

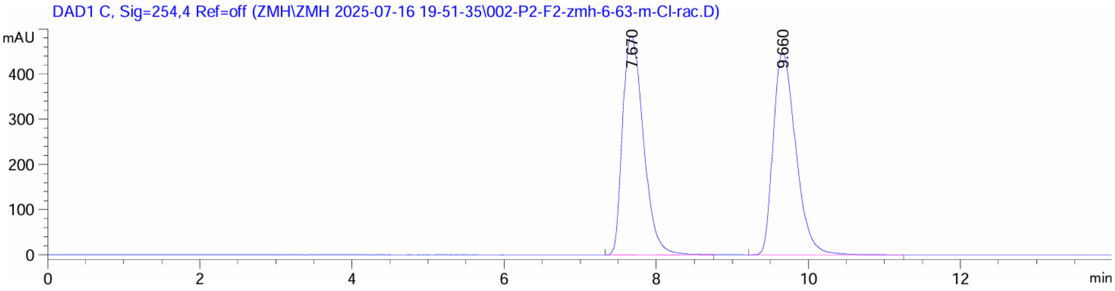

Peak #	RetTime [min]	Type	Width [min]	Area [mAU*s]	Height [mAU]	Area %
1	7.670	BB	0.3019	9228.03027	485.10199	49.8744
2	9.660	BB	0.3242	9274.50391	446.69131	50.1256

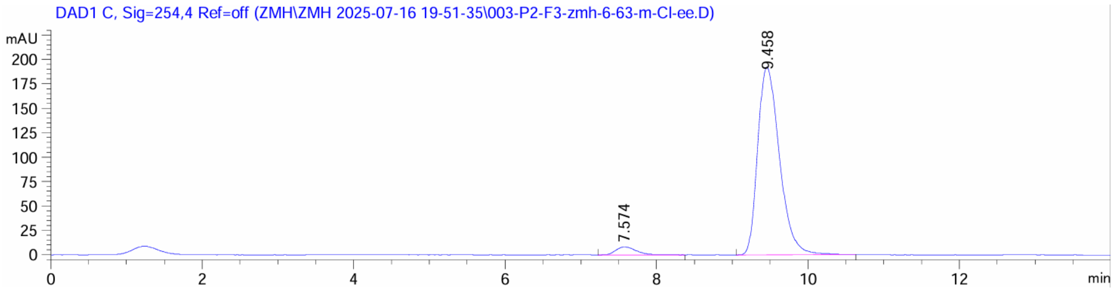

Peak #	RetTime [min]	Type	Width [min]	Area [mAU*s]	Height [mAU]	Area %
1	7.574	BB	0.2808	152.33594	8.12296	3.8153
2	9.458	BB	0.3112	3840.47314	192.12473	96.1847

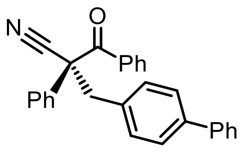

(+)-**38**

(+)-**38** was obtained as a white solid from the desymmetric addition of **S38** using the general procedure (58.5 mg, 76% Yield). R_f = 0.6 (Hexane/EtOAc = 10:1).

HPLC analysis (Chiralpak IC-3, hexane/^{i}PrOH = 99:1, 0.8 mL/min, 254 nm; t_r (minor) = 11.49 min, t_r (major) = 15.80 min) gave the isomeric composition of the product: 96:4 e.r., $[\alpha]_D^{20}$ = +34.2 (*c* = 1.0, $CHCl_3$).

^1H NMR (400 MHz, $CDCl_3$) δ 7.85 (d, *J* = 7.8 Hz, 2H), 7.55 (d, *J* = 7.6 Hz, 2H), 7.51 – 7.27 (m,

13H), 6.98 (d, J = 7.9 Hz, 2H), 3.69 (d, J = 13.7 Hz, 1H), 3.46 (d, J = 13.7 Hz, 1H).

^{13}C NMR (101 MHz, $CDCl_3$) δ 191.0, 140.6, 140.1, 134.8, 133.9, 133.6, 133.1, 131.1, 130.1, 129.5, 128.74, 128.68, 128.4, 127.2, 126.9, 126.6, 126.4, 119.1, 58.3, 44.5.

HRMS (ESI) calcd $C_{28}H_{22}NO^+$ $[M+H]^+$: 388.1696. Found: 388.1696.

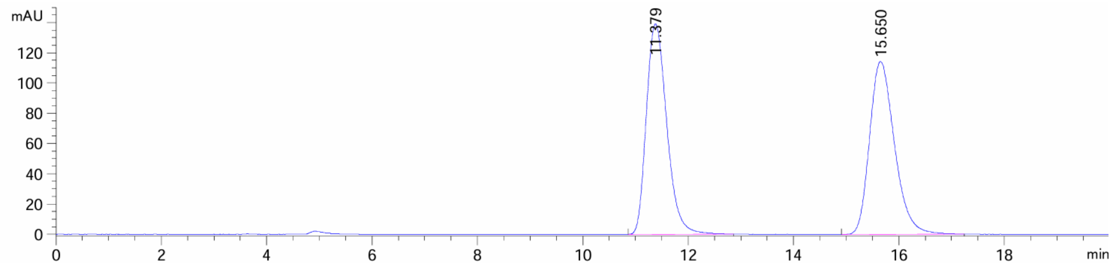

Peak #	RetTime [min]	Type	Width [min]	Area [mAU*s]	Height [mAU]	Area %
1	11.379	BB	0.4227	3750.03271	139.02708	49.8800
2	15.650	BB	0.5083	3768.08179	114.38589	50.1200

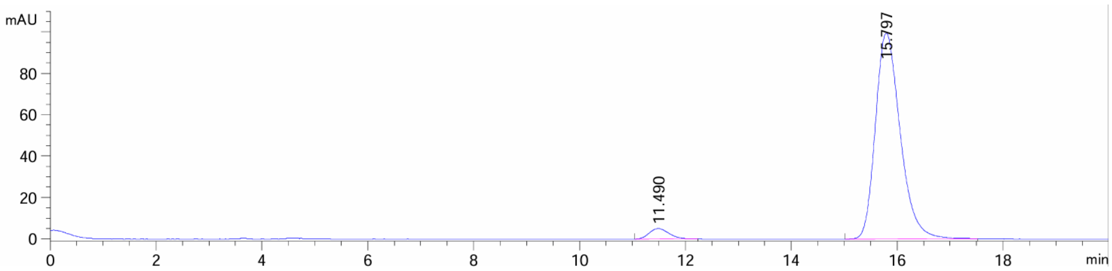

Peak #	RetTime [min]	Type	Width [min]	Area [mAU*s]	Height [mAU]	Area %
1	11.490	BB	0.3782	131.38026	5.03792	3.8973
2	15.797	BB	0.4997	3239.66553	99.53647	96.1027

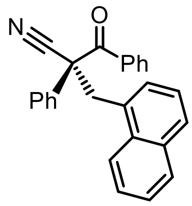

(+)-**39**

(+)-**39** was obtained as a white solid from the desymmetric addition of **S39** using the general procedure (45.8 mg, 63% Yield). R_f = 0.7 (Hexane/EtOAc = 10:1).

HPLC analysis (Chiralpak IC-3, hexane/^{i}PrOH = 99:1, 1.0 mL/min, 254 nm; t_r (minor) = 7.37 min, t_r (major) = 9.55 min) gave the isomeric composition of the product: 96.5:3.5 e.r., $[\alpha]_D^{20}$ = +107.8 (c = 1.0, $CHCl_3$).

^{1}H NMR (400 MHz, CDCl$_3$) δ 7.85 (d, *J* = 8.0 Hz, 2H), 7.75 (t, *J* = 8.4 Hz, 2H), 7.57 (d, *J* = 8.7 Hz, 1H), 7.47 (t, *J* = 7.5 Hz, 1H), 7.35 – 7.29 (m, 4H), 7.26 – 7.17 (m, 6H), 7.16 (d, *J* = 7.1 Hz, 1H), 4.14 (d, *J* = 14.4 Hz, 1H), 3.97 (d, *J* = 14.4 Hz, 1H).

^{13}C NMR (101 MHz, CDCl$_3$) δ 191.5, 135.1, 133.72, 133.66, 133.5, 132.7, 130.32, 130.26, 129.4, 129.3, 128.7, 128.41, 128.38, 128.2, 126.6, 125.5, 125.3, 124.9, 123.6, 119.4, 57.9, 40.1.

HRMS (ESI) calcd $C_{26}H_{19}NNaO^{+}$ $[M+Na]^{+}$: 384.1359. Found: 384.1359.

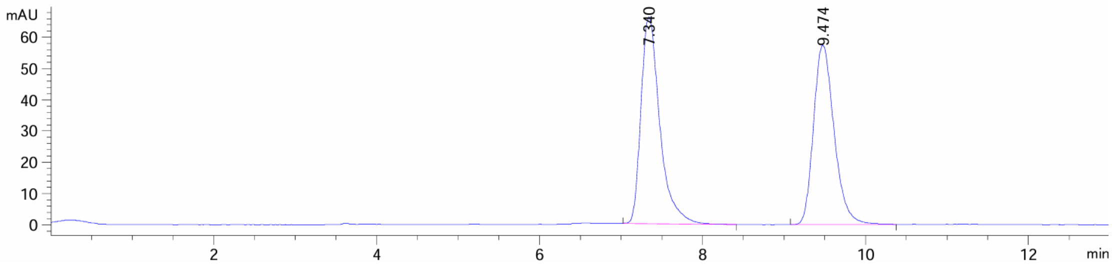

Peak #	RetTime [min]	Type	Width [min]	Area [mAU*s]	Height [mAU]	Area %
1	7.340	BB	0.2453	1062.07751	66.16065	51.3411
2	9.474	BB	0.2703	1006.59363	57.47905	48.6589

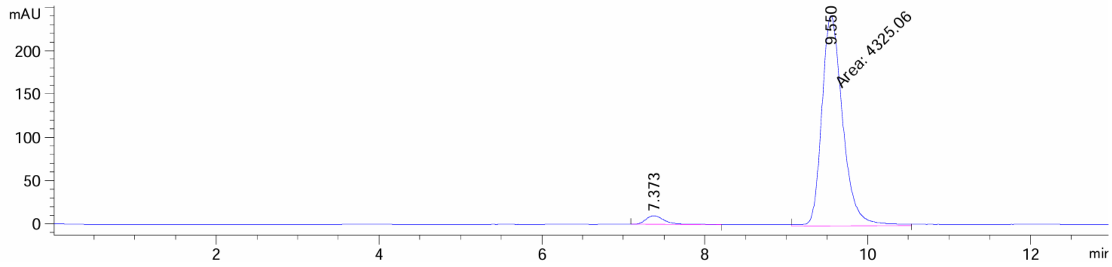

Peak #	RetTime [min]	Type	Width [min]	Area [mAU*s]	Height [mAU]	Area %
1	7.373	BB	0.2427	155.33885	9.92058	3.4671
2	9.550	MM	0.2973	4325.05518	242.44957	96.5329

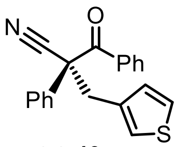

(+)-**40**

(+)-**40** was obtained as a white solid from the desymmetric addition of **S37** using the general procedure (47.2 mg, 74% Yield). R_f = 0.7 (Hexane/EtOAc = 10:1).

HPLC analysis (Chiralpak IC-3, hexane/^{i}PrOH = 99:1, 1.0 mL/min, 254 nm; t_r (minor) = 8.81 min, t_r

(major) =10.76 min) gave the isomeric composition of the product: 95:5 e.r., $[\alpha]_D^{20}$ = +106.2 (c = 1.0, $CHCl_3$).

^{1}H NMR (400 MHz, $CDCl_3$) δ 7.84 (d, J = 7.8 Hz, 2H), 7.48 (d, J = 7.6 Hz, 1H), 7.34 (d, J = 12.2 Hz, 7H), 7.12 (t, J = 3.1 Hz, 1H), 6.89 (s, 1H), 6.65 (d, J = 4.8 Hz, 1H), 3.72 (d, J = 14.1 Hz, 1H), 3.45 (d, J = 14.1 Hz, 1H).

^{13}C NMR (101 MHz, $CDCl_3$) δ 191.0, 135.0, 134.4, 133.9, 133.6, 130.0, 129.5, 129.4, 128.8, 128.4, 126.2, 124.80, 124.78, 119.4, 58.0, 39.6.

HRMS (ESI) calcd $C_{20}H_{16}NOS^+$ $[M+H]^+$: 318.0947. Found: 318.0947.

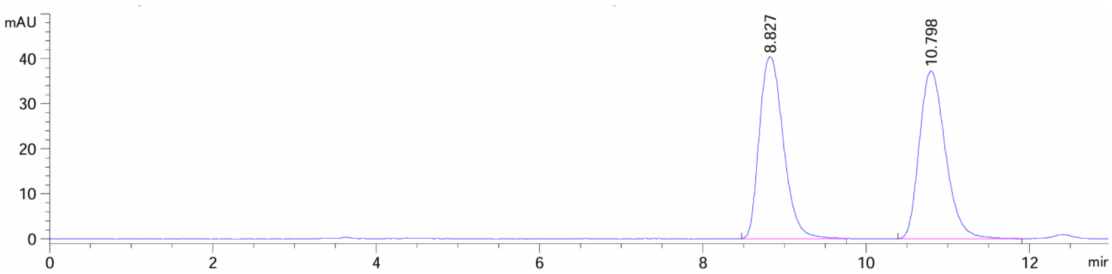

Peak #	RetTime [min]	Type	Width [min]	Area [mAU*s]	Height [mAU]	Area %
1	8.827	BB	0.3227	827.51056	40.44897	50.0654
2	10.798	BB	0.3446	825.35004	37.23442	49.9346

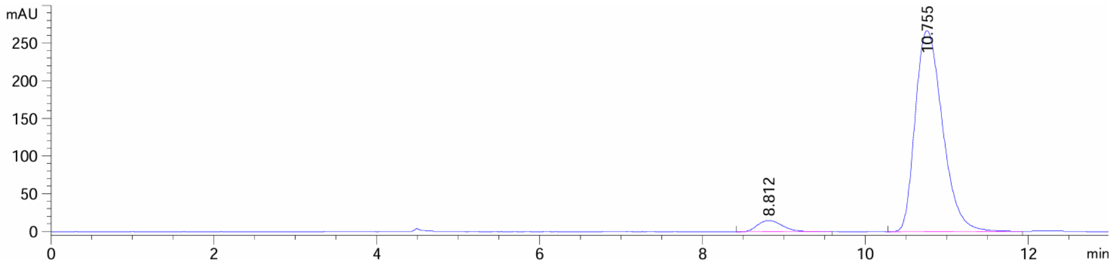

Peak #	RetTime [min]	Type	Width [min]	Area [mAU*s]	Height [mAU]	Area %
1	8.812	BB	0.3361	310.90527	14.62105	4.7750
2	10.755	BB	0.3654	6200.15137	266.48621	95.2250

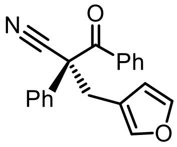

(+)-**41**

(+)-**41** was obtained as colorless oil from the desymmetric addition of **S41** using the general procedure (50.7 mg, 84% Yield). R_f = 0.7 (Hexane/EtOAc = 10:1).

HPLC analysis (Chiralpak IC-3, hexane/^{i}PrOH = 99:1, 1.0 mL/min, 254 nm; t_r (minor) = 9.05 min, t_r (major) = 10.63 min) gave the isomeric composition of the product: 95:5 e.r., $[\alpha]_D^{20}$ = +97.0 (*c* = 1.0, $CHCl_3$).

^{1}H NMR (500 MHz, $CDCl_3$) δ 7.84 (d, *J* = 8.3 Hz, 2H), 7.48 (t, *J* = 7.4 Hz, 1H), 7.44 – 7.31 (m, 7H), 7.25 (s, 1H), 7.14 (s, 1H), 6.02 (s, 1H), 3.54 (d, *J* = 14.4 Hz, 1H), 3.23 (d, *J* = 14.4 Hz, 1H).

^{13}C NMR (126 MHz, $CDCl_3$) δ 190.9, 142.4, 141.8, 135.0, 133.9, 133.7, 130.0, 129.6, 128.8, 128.4, 126.2, 119.4, 117.8, 112.0, 57.9, 35.2.

HRMS (ESI) calcd $C_{20}H_{16}NO_2^+$ $[M+H]^+$: 302.1176. Found: 302.1176.

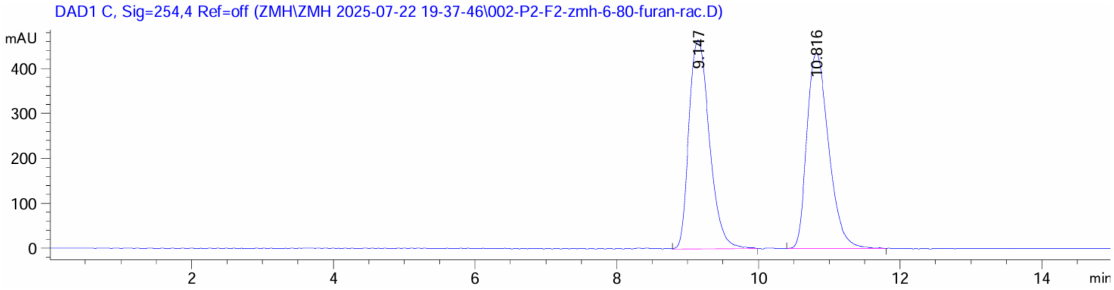

Peak #	RetTime [min]	Type	Width [min]	Area [mAU*s]	Height [mAU]	Area %
1	9.147	BV R	0.3183	9352.58887	464.78558	50.1699
2	10.816	BV R	0.3333	9289.23340	434.07703	49.8301

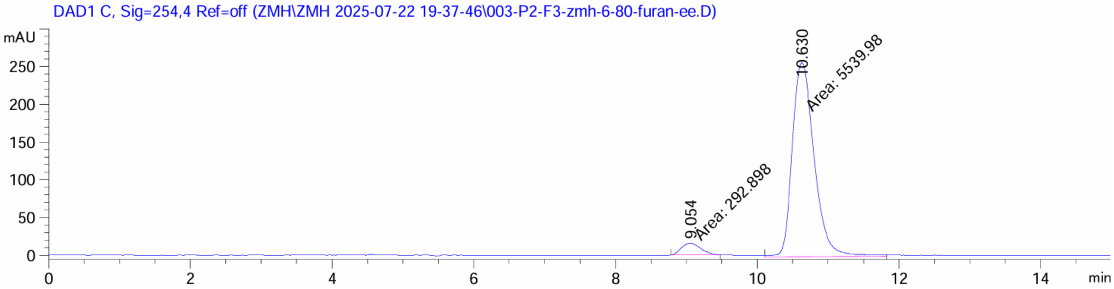

Peak #	RetTime [min]	Type	Width [min]	Area [mAU*s]	Height [mAU]	Area %
1	9.054	MM	0.3142	292.89835	15.53817	5.0215
2	10.630	MM	0.3591	5539.98193	257.12756	94.9785

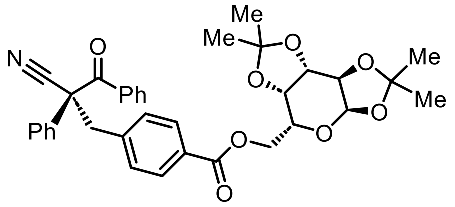

(+)-**42**

(+)-**42** was obtained as colorless oil from the desymmetric addition of **S42** using the general procedure (68.2 mg, 57% Yield). R_f = 0.7 (Hexane/EtOAc = 3:1).

HPLC analysis (Chiralpak IC-3, hexane/^{i}PrOH = 80:20, 1.0 mL/min, 254 nm; tr (minor) = 20.80 min, tr (major) = 24.16 min) gave the isomeric composition of the product: 95:5 d.r. $[\alpha]_D^{20}$ = +79.4 (c = 1.0, $CHCl_3$).

^{1}H NMR (400 MHz, $CDCl_3$) δ 7.86 – 7.83 (m, 4H), 7.49 (t, J = 7.4 Hz, 1H), 7.40 – 7.30 (m, 5H), 7.28 – 7.25 (m, 2H), 6.96 (d, J = 8.2 Hz, 2H), 5.56 (d, J = 5.0 Hz, 1H), 4.66 – 4.64 (m, 1H), 4.51 – 4.47 (m, 1H), 4.44 – 4.29 (m, 3H), 4.18 – 4.14 (m, 1H), 3.67 (d, J = 13.6 Hz, 1H), 3.49 (d, J = 13.6 Hz, 1H), 1.52 (s, 3H), 1.47 (s, 3H), 1.36 (s, 3H), 1.34 (s, 3H).

^{13}C NMR (101 MHz, $CDCl_3$) δ 190.6, 166.2, 139.5, 134.4, 133.8, 133.6, 130.7, 130.2, 129.6, 129.3, 129.1, 129.0, 128.5, 126.3, 118.8, 109.7, 108.8, 96.3, 71.1, 70.7, 70.5, 66.1, 63.8, 57.9, 44.6, 26.03, 25.95, 25.0, 24.5.

HRMS (ESI) calcd $C_{35}H_{36}NO_8^+$ $[M+H]^+$: 598.2435. Found: 598.2437.

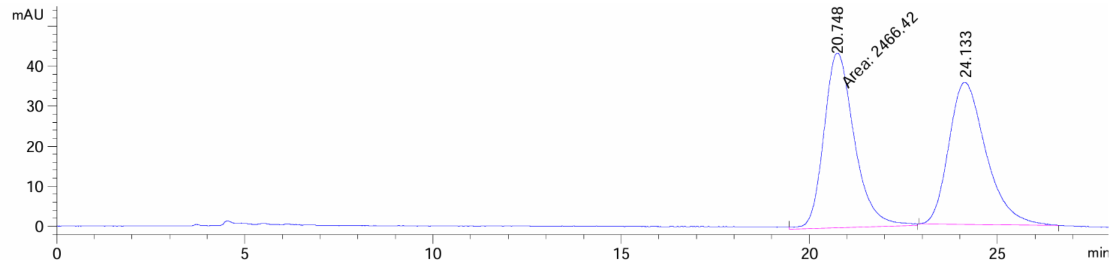

Peak #	RetTime [min]	Type	Width [min]	Area [mAU*s]	Height [mAU]	Area %
1	20.748	MM	0.9408	2466.42480	43.69199	50.2813
2	24.133	BB	0.8414	2438.82886	35.50450	49.7187

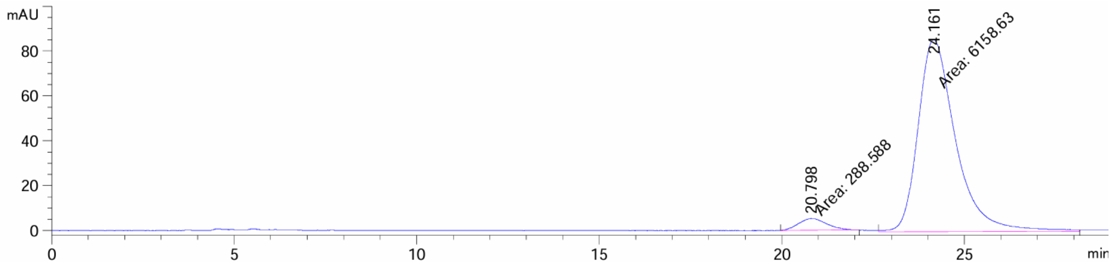

Peak #	RetTime [min]	Type	Width [min]	Area [mAU*s]	Height [mAU]	Area %
1	20.798	MM	0.9122	288.58807	5.27303	4.4762
2	24.161	MM	1.1970	6158.63330	85.74748	95.5238

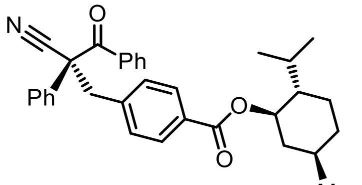

(+)-**43**

(+)-**43** was obtained as colorless oil from the desymmetric addition of **S43** using the general procedure (79.0 mg, 80% Yield). R_f = 0.7 (Hexane/EtOAc = 10:1).

HPLC analysis (Chiralpak IC-3, hexane/^{i}PrOH = 98/2, 1.0 mL/min, 254 nm; tr (minor) = 8.35 min, tr (major) = 11.74 min) gave the isomeric composition of the product: 95:5 d.r., $[\alpha]_D^{20}$ = +88.8 (*c* = 1.0, $CHCl_3$).

^{1}H NMR (400 MHz, $CDCl_3$) δ 7.84 (d, *J* = 7.9 Hz, 4H), 7.48 (t, *J* = 7.4 Hz, 1H), 7.43 – 7.27 (m, 7H), 6.96 (d, *J* = 7.8 Hz, 2H), 4.93 – 4.87 (m, 1H), 3.68 (d, *J* = 13.6 Hz, 1H), 3.48 (d, *J* = 13.6 Hz, 1H), 2.11 (d, *J* = 11.8 Hz, 1H), 2.00 – 1.93 (m, 1H), 1.78 – 1.66 (m, 2H), 1.53 (t, *J* = 11.7 Hz, 2H), 1.17 – 1.03 (m, 2H), 0.96 – 0.87 (m, 7H), 0.78 (d, *J* = 6.9 Hz, 3H).

^{13}C NMR (101 MHz, $CDCl_3$) δ 190.6, 165.9, 139.2, 134.4, 133.8, 133.6, 130.7, 130.1, 129.9, 129.6, 129.1, 128.9, 128.5, 126.4, 118.8, 74.8, 57.9, 47.2, 44.7, 40.9, 34.3, 31.4, 26.3, 23.5, 22.0, 20.8, 16.4.

HRMS (ESI) calcd $C_{33}H_{36}NO_3^+$ $[M+H]^+$: 494.2690. Found: 494.2690.

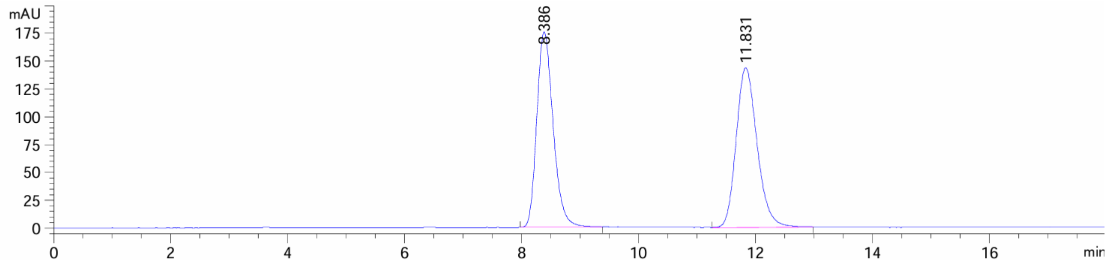

Peak #	RetTime [min]	Type	Width [min]	Area [mAU*s]	Height [mAU]	Area %
1	8.386	BB	0.3004	3406.01538	175.47971	48.9149
2	11.831	BB	0.3815	3557.13013	143.33849	51.0851

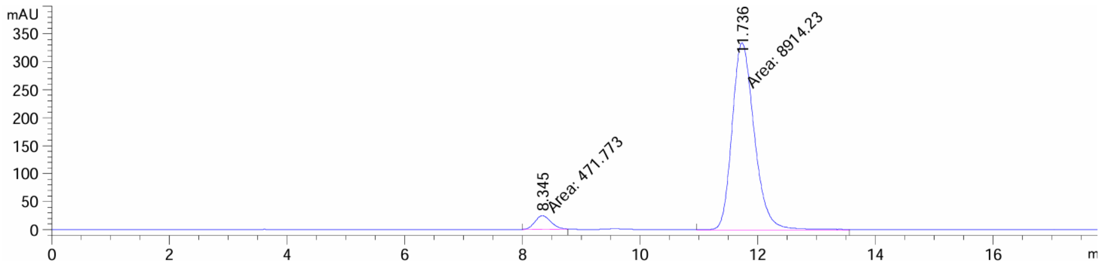

Peak #	RetTime [min]	Type	Width [min]	Area [mAU*s]	Height [mAU]	Area %
1	8.345	MM	0.3195	471.77301	24.60842	5.0263
2	11.736	MM	0.4431	8914.23242	335.32458	94.9737

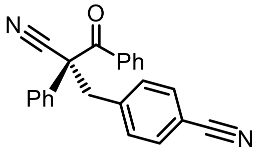

(+)-**44**

(+)-**44** was obtained as colorless oil from the desymmetric addition of **S44** using the general procedure (53.5 mg, 80% Yield). R_f = 0.7 (Hexane/EtOAc = 5:1).

HPLC analysis (Chiralpak IB-3, hexane/^{i}PrOH = 95:5, 1.0 mL/min, 230 nm; t_r (minor) = 11.13 min, t_r (major) = 12.72 min) gave the isomeric composition of the product: 95:5 e.r., $[\alpha]_D^{20}$ = +97.7 (*c* = 1.0, $CHCl_3$).

^{1}H NMR (400 MHz, $CDCl_3$) δ 7.84 (d, *J* = 7.8 Hz, 2H), 7.52 – 7.48 (m, 3H), 7.41 – 7.31 (m, 5H), 7.28 – 7.25 (m, 2H), 7.02 (d, *J* = 8.0 Hz, 2H), 3.67 (d, *J* = 13.7 Hz, 1H), 3.48 (d, *J* = 13.6 Hz, 1H).

^{13}C NMR (101 MHz, $CDCl_3$) δ 190.2, 139.7, 134.1, 133.9, 133.4, 131.7, 131.4, 130.1, 129.7, 129.1, 128.5, 126.2, 118.62, 118.59, 111.4, 57.7, 44.7.

HRMS (ESI) calcd $C_{23}H_{17}N_2O^+$ $[M+H]^+$: 337.1335. Found: 337.1336.

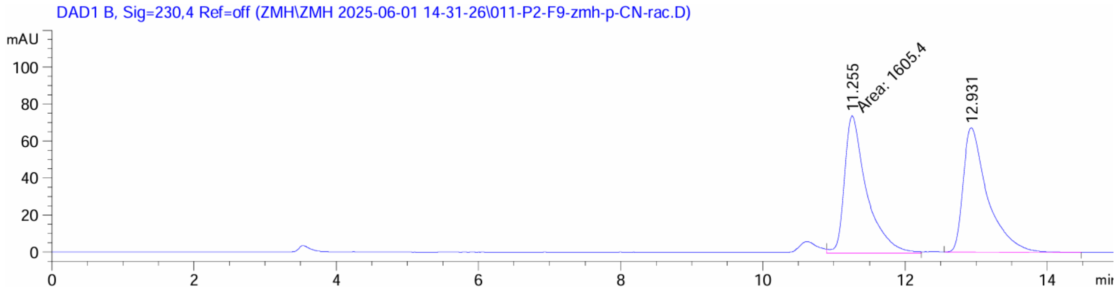

Peak #	RetTime [min]	Type	Width [min]	Area [mAU*s]	Height [mAU]	Area %
1	11.255	MM	0.3599	1605.39917	74.34938	50.0378
2	12.931	BB	0.3475	1602.97498	67.39281	49.9622

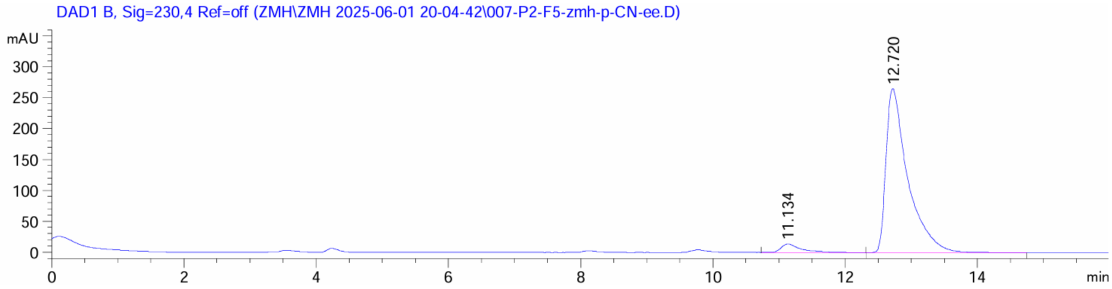

Peak #	RetTime [min]	Type	Width [min]	Area [mAU*s]	Height [mAU]	Area %
1	11.134	BB	0.3092	294.47690	13.78481	4.6041
2	12.720	BV R	0.3340	6101.50537	265.57947	95.3959

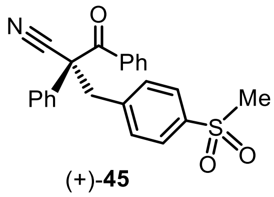

(+)-**45**

(+)-**45** was obtained as a white solid from the desymmetric addition of **S45** using the general procedure (51.1 mg, 66% Yield). R_f = 0.5 (Hexane/EtOAc = 3:1).

HPLC analysis (Chiralpak IC-3, hexane/^{i}PrOH = 70:30, 1.0 mL/min, 254 nm; t_r (minor) = 55.72 min, t_r (major) = 62.63 min) gave the isomeric composition of the product: 95:5 e.r., $[\alpha]_D^{20}$ = +99.3 (c = 1.0, $CHCl_3$).

^{1}H NMR (400 MHz, $CDCl_3$) δ 7.88 – 7.81 (m, 2H), 7.76 (d, J = 8.4 Hz, 2H), 7.52 – 7.48 (m, 1H), 7.42 – 7.27 (m, 7H), 7.13 (d, J = 8.4 Hz, 2H), 3.73 (d, J = 13.6 Hz, 1H), 3.50 (d, J = 13.6 Hz, 1H), 3.03 (s, 3H).

^{13}C NMR (101 MHz, $CDCl_3$) δ 190.2, 140.7, 139.6, 134.1, 134.0, 133.4, 131.7, 130.2, 129.8, 129.2, 128.5, 127.0, 126.2, 118.6, 57.7, 44.5, 44.4.

HRMS (ESI) calcd $C_{23}H_{20}ClNO_3S^+$ $[M+H]^+$: 390.1158. Found: 390.1158.

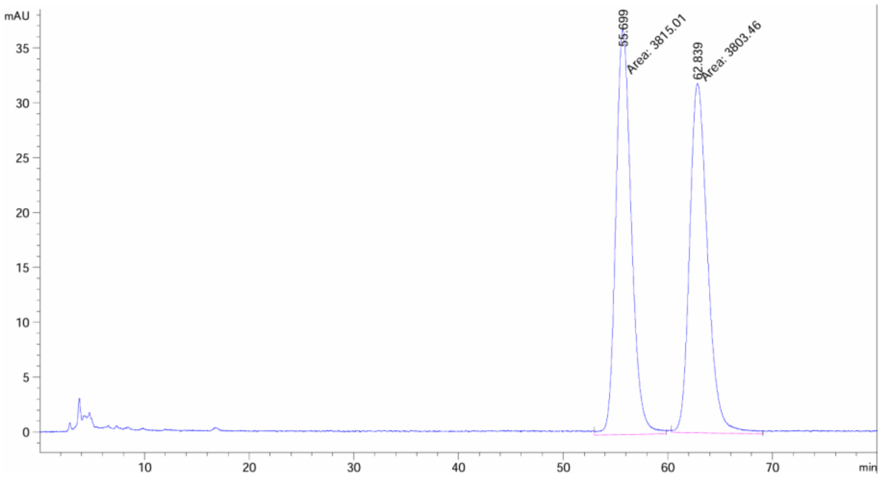

Peak #	RetTime [min]	Type	Width [min]	Area [mAU*s]	Height [mAU]	Area %
1	55.699	MM	1.7218	3815.01270	36.92750	50.0758
2	62.839	MM	1.9905	3803.46167	31.84640	49.9242

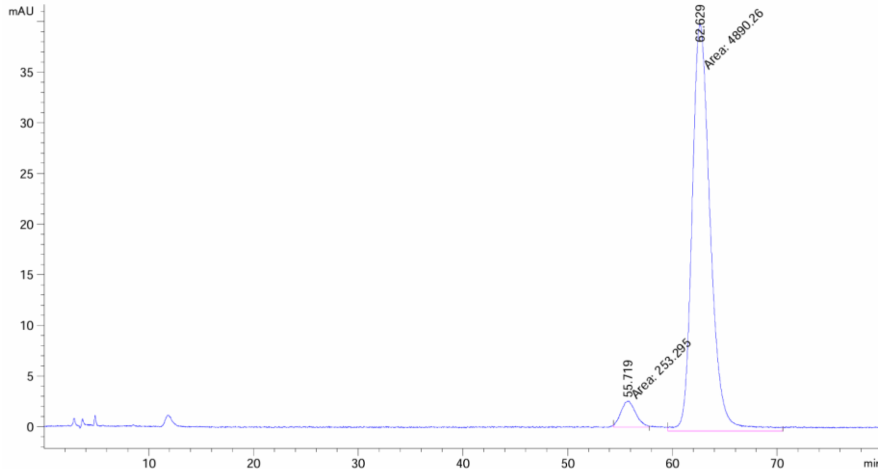

Peak #	RetTime [min]	Type	Width [min]	Area [mAU*s]	Height [mAU]	Area %
1	55.719	MM	1.6451	253.29482	2.56612	4.9245
2	62.629	MM	2.0305	4890.26025	40.14034	95.0755

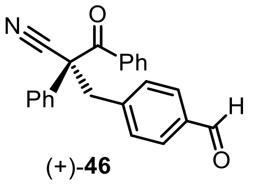

(+)-**46**

(+)-**46** was obtained as a white solid from the desymmetric addition of **S46** using the general procedure (46.9 mg, 69% Yield). R_f = 0.7 (Hexane/EtOAc = 5:1).

HPLC analysis (Chiralpak IC-3, hexane/^{i}PrOH = 90:10, 1.0 mL/min, 254 nm; t_r (minor) = 22.74 min,

t_r (major) = 25.12 min) gave the isomeric composition of the product: 96:4 e.r., $[\alpha]_D^{20}$ = +83.2 (c = 1.0, $CHCl_3$).

^{1}H NMR (400 MHz, $CDCl_3$) δ 9.95 (s, 1H), 7.85 (d, J = 7.9 Hz, 2H), 7.69 (d, J = 7.9 Hz, 2H), 7.49 (t, J = 7.4 Hz, 1H), 7.40 – 7.30 (m, 5H), 7.29 – 7.26 (m, 2H), 7.07 (d, J = 7.9 Hz, 2H), 3.71 (d, J = 13.5 Hz, 1H), 3.52 (d, J = 13.6 Hz, 1H).

^{13}C NMR (101 MHz, $CDCl_3$) δ 191.9, 190.4, 141.2, 135.4, 134.3, 133.8, 133.5, 131.4, 130.1, 129.6, 129.2, 129.0, 128.5, 126.3, 118.7, 57.8, 44.7.

HRMS (ESI) calcd $C_{23}H_{18}NO_2^+$ $[M+H]^+$: 340.1332. Found: 340.1332.

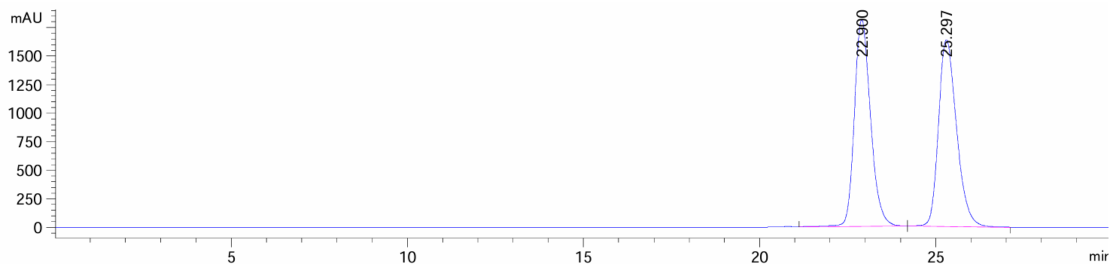

Peak #	RetTime [min]	Type	Width [min]	Area [mAU*s]	Height [mAU]	Area %
1	22.900	BB	0.4946	5.77842e4	1809.29285	49.7871
2	25.297	BB	0.5498	5.82783e4	1635.37671	50.2129

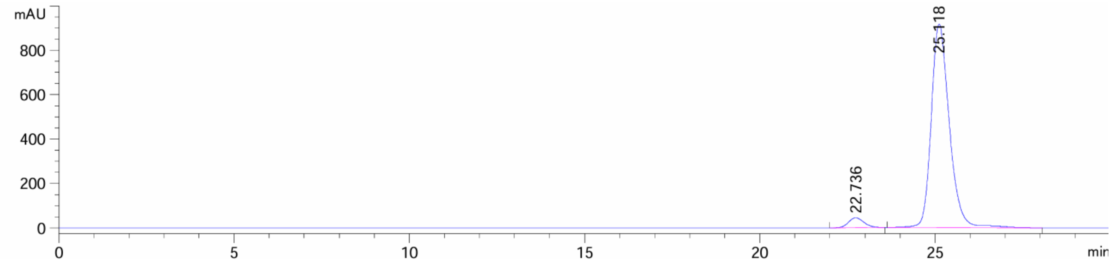

Peak #	RetTime [min]	Type	Width [min]	Area [mAU*s]	Height [mAU]	Area %
1	22.736	BB	0.4654	1348.19592	44.51020	3.9445
2	25.118	BB	0.5465	3.28311e4	915.27484	96.0555

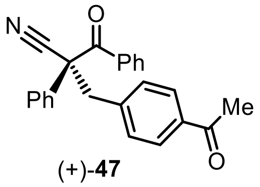

(+)-**47**

(+)-**47** was obtained as a white solid from the desymmetric addition of **S47** using the general procedure (50.3 mg, 71% Yield). R_f = 0.6 (Hexane/EtOAc = 5:1).

HPLC analysis (Chiralpak IC-3, hexane/^{i}PrOH = 90:10, 1.0 mL/min, 254 nm; t_r (minor) = 21.99 min, t_r (major) = 27.67 min) gave the isomeric composition of the product: 95:5 e.r., $[\alpha]_D^{20}$ = +87.1 (*c* = 1.0, $CHCl_3$).

^{1}H NMR (400 MHz, $CDCl_3$) δ 7.87 – 7.81 (m, 2H), 7.77 (d, *J* = 8.3 Hz, 2H), 7.52 – 7.46 (m, 1H), 7.40 – 7.30 (m, 5H), 7.31 – 7.26 (m, 2H), 7.00 (d, *J* = 8.3 Hz, 2H), 3.69 (d, *J* = 13.5 Hz, 1H), 3.49 (d, *J* = 13.6 Hz, 1H), 2.56 (s, 3H).

^{13}C NMR (101 MHz, $CDCl_3$) δ 197.83, 190.57, 139.68, 136.14, 134.38, 133.80, 133.56, 130.95, 130.13, 129.63, 128.96, 128.47, 127.93, 126.31, 118.81, 57.88, 44.62, 26.57.

HRMS (ESI) calcd $C_{24}H_{20}NO_2^+$ $[M+H]^+$: 354.1489. Found: 354.1489.

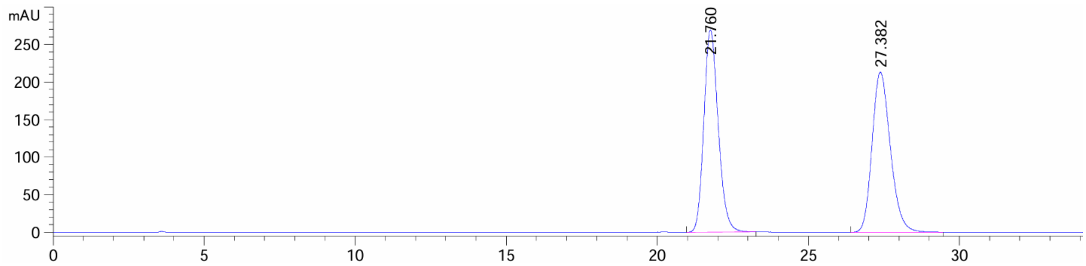

Peak #	RetTime [min]	Type	Width [min]	Area [mAU*s]	Height [mAU]	Area %
1	21.760	BB	0.5108	8885.57227	269.38589	49.7500
2	27.382	BB	0.6471	8974.87207	213.20016	50.2500

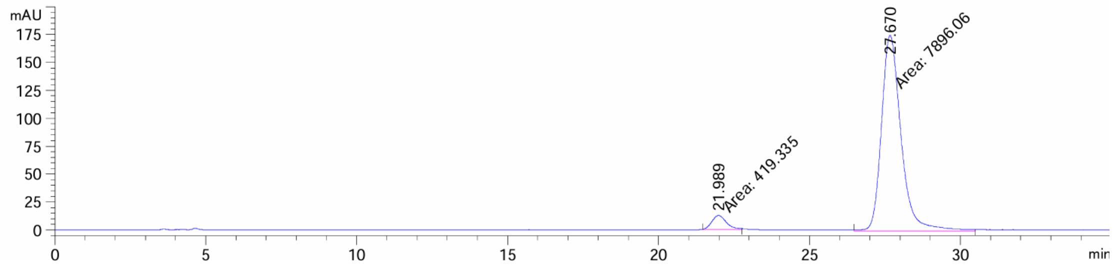

Peak #	RetTime [min]	Type	Width [min]	Area [mAU*s]	Height [mAU]	Area %
1	21.989	MM	0.5590	419.33505	12.50247	5.0429
2	27.670	MM	0.7515	7896.05908	175.10860	94.9571

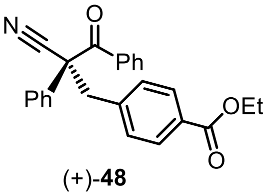

(+)-**48**

(+)-**48** was obtained as a white solid from the desymmetric addition of **S48** using the general procedure (42.4 mg, 55% Yield). R_f = 0.6 (Hexane/EtOAc = 3:1).

HPLC analysis (Chiralpak IB-3, hexane/^{i}PrOH = 95:5, 1.0 mL/min, 254 nm; t_r (minor) = 7.33 min, t_r (major) =8.14 min) gave the isomeric composition of the product: 95:5 e.r., $[\alpha]_D^{20}$ = +46.7 (*c* = 1.0, $CHCl_3$).

^{1}H NMR (400 MHz, $CDCl_3$) δ 7.89 – 7.80 (m, 4H), 7.53 – 7.43 (m, 1H), 7.40 – 7.31 (m, 5H), 7.30 – 7.22 (m, 2H), 6.96 (d, *J* = 8.1 Hz, 2H), 4.35 (q, *J* = 7.1 Hz, 2H), 3.68 (d, *J* = 13.6 Hz, 1H), 3.49 (d, *J* = 13.6 Hz, 1H), 1.37 (t, *J* = 7.1 Hz, 3H).

^{13}C NMR (101 MHz, $CDCl_3$) δ 190.7, 166.4, 139.3, 134.4, 133.8, 133.6, 130.7, 130.1, 129.61, 129.56, 129.1, 128.9, 128.5, 126.3, 118.8, 60.9, 57.9, 44.6, 14.3.

HRMS (ESI) calcd $C_{25}H_{22}NO_3^+$ $[M+H]^+$: 384.1594. Found: 384.1593.

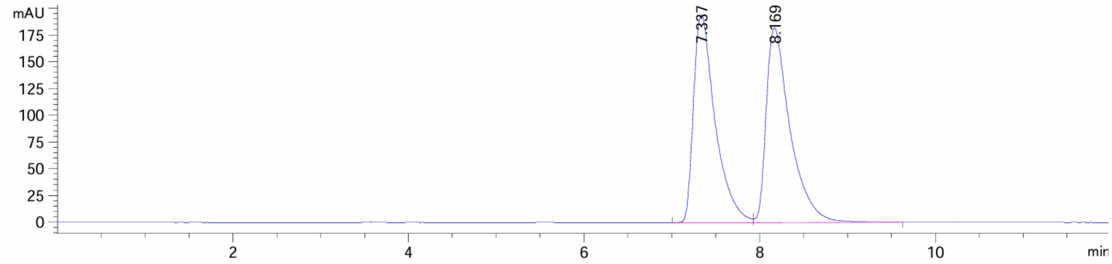

Peak #	RetTime [min]	Type	Width [min]	Area [mAU*s]	Height [mAU]	Area %
1	7.337	BV	0.2563	3331.39063	194.03235	49.1392
2	8.169	VB	0.2812	3448.11182	181.84273	50.8608

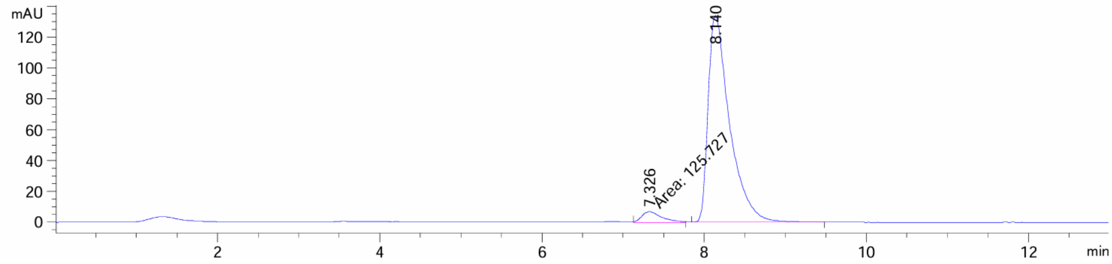

Peak #	RetTime [min]	Type	Width [min]	Area [mAU*s]	Height [mAU]	Area %
1	7.326	MM	0.2934	125.72677	7.14199	4.8575
2	8.140	VV R	0.2703	2462.59082	134.00793	95.1425

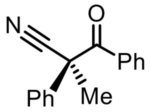

(+)-**49**

(+)-**49** was obtained as colorless oil from the desymmetric addition of **S49** using the general procedure (42.8 mg, 91% Yield). R_f = 0.7 (Hexane/EtOAc = 10:1).

HPLC analysis (Chiralpak IC-3, hexane/^{i}PrOH = 99/1, 1.0 mL/min, 254 nm; tr (minor) = 8.58 min, tr (major) = 9.33 min) gave the isomeric composition of the product: 87:13 e.r., $[\alpha]_D^{20}$ = +91.4 (*c* = 1.0, $CHCl_3$).

^{1}H NMR (400 MHz, $CDCl_3$) δ 7.86 (d, *J* = 7.8 Hz, 2H), 7.48 – 7.47 (m, 3H), 7.41 (t, *J* = 7.7 Hz, 2H), 7.38 – 7.28 (m, 3H), 1.92 (s, 3H).

^{13}C NMR (101 MHz, $CDCl_3$) δ 191.1, 137.4, 133.6, 133.4, 130.1, 129.7, 128.6, 128.4, 125.4, 120.5, 51.6, 27.4.

HRMS (ESI) calcd $C_{16}H_{14}NO^+$ $[M+H]^+$: 236.1070. Found: 236.1070.

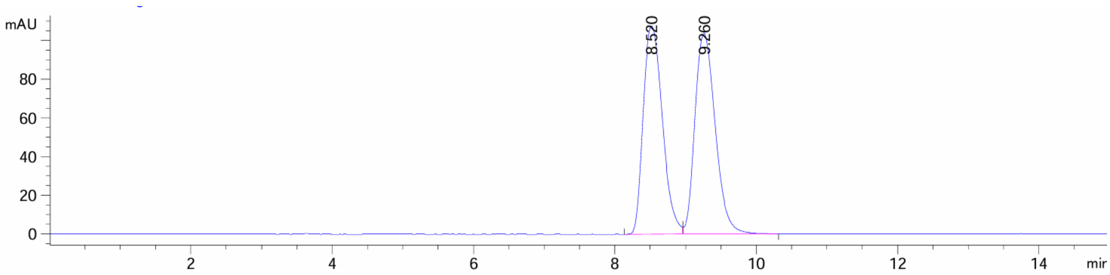

Peak #	RetTime [min]	Type	Width [min]	Area [mAU*s]	Height [mAU]	Area %
1	8.520	BV	0.2983	2031.59253	107.60322	49.4345
2	9.260	VB	0.3158	2078.07129	103.71334	50.5655

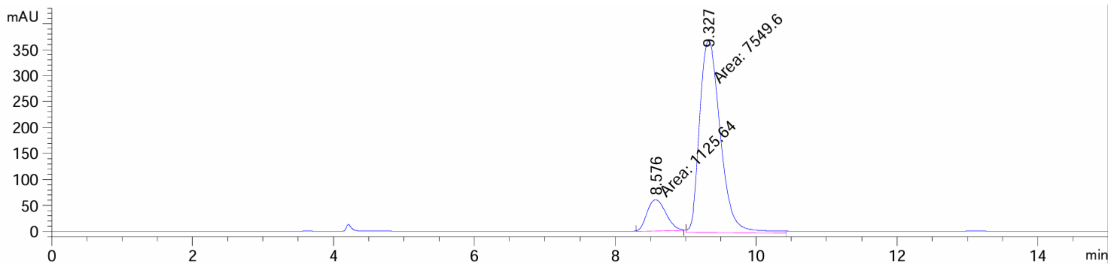

Peak #	RetTime [min]	Type	Width [min]	Area [mAU*s]	Height [mAU]	Area %
1	8.576	MM	0.3102	1125.64075	60.47396	12.9753
2	9.327	MM	0.3409	7549.60059	369.05038	87.0247

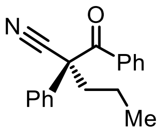

(+)-**50**

(+)-**50** was obtained as colorless oil from the desymmetric addition of **S50** using the general procedure (43.1 mg, 82% Yield). R_f = 0.7 (Hexane/EtOAc = 10:1).

HPLC analysis (Chiralpak IC-3, hexane/^{i}PrOH = 99/1, 1.0 mL/min, 254 nm; tr (minor) = 6.63 min, tr (major) = 7.35 min) gave the isomeric composition of the product: 92:8 e.r., $[\alpha]_D^{20}$ = +105.3 (*c* = 1.0, $CHCl_3$).

^{1}H NMR (600 MHz, $CDCl_3$) δ 7.83 (d, *J* = 7.8 Hz, 2H), 7.51 – 7.45 (m, 3H), 7.43 – 7.40 (m, 2H), 7.38 – 7.30 (m, 3H), 2.44 – 2.31 (m, 1H), 2.12 – 2.01 (m, 1H), 1.63 – 1.45 (m, 1H), 1.32 – 1.17 (m, 1H), 0.95 (t, *J* = 7.3 Hz, 3H).

^{13}C NMR (151 MHz, $CDCl_3$) δ 191.3, 135.7, 134.1, 133.5, 129.9, 129.6, 128.6, 128.4, 125.9, 119.8, 57.0, 41.3, 18.4, 13.8.

HRMS (ESI) calcd $C_{18}H_{18}NO^+$ $[M+H]^+$: 264.1383. Found: 264.1384.

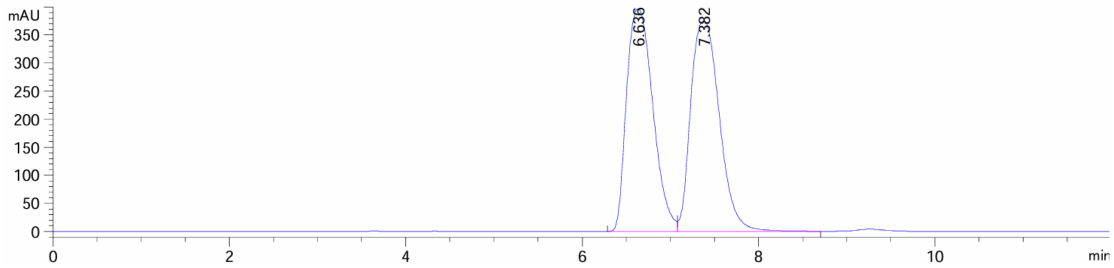

Peak #	RetTime [min]	Type	Width [min]	Area [mAU*s]	Height [mAU]	Area %
1	6.636	BV	0.3337	8216.73242	396.55664	49.2767
2	7.382	VB	0.3609	8457.96289	375.35709	50.7233

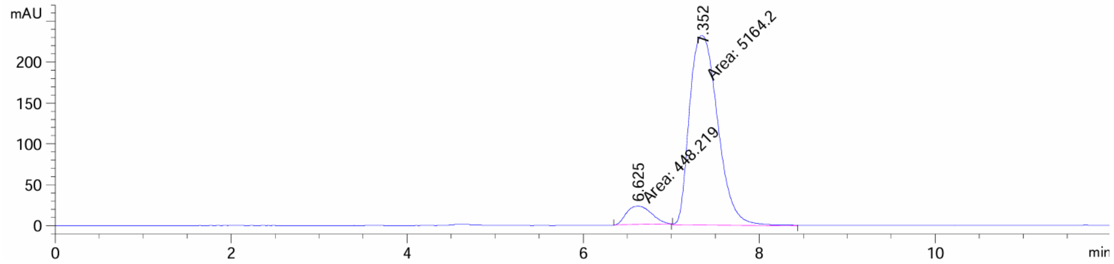

Peak #	RetTime [min]	Type	Width [min]	Area [mAU*s]	Height [mAU]	Area %
1	6.625	MM	0.3307	448.21887	22.59134	7.9862
2	7.352	MM	0.3711	5164.19727	231.96152	92.0138

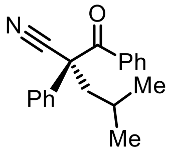

(+)-**51**

(+)-**51** was obtained as colorless oil from the desymmetric addition of **S51** using the general procedure (35.6 mg, 64% Yield). R_f = 0.6 (Hexane/EtOAc = 10:1).

HPLC analysis (Chiralpak IC-3, hexane/^{i}PrOH = 99/1, 1.0 mL/min, 254 nm; tr (minor) = 5.92 min, tr (major) = 6.56 min) gave the isomeric composition of the product: 93:7 e.r., $[\alpha]_D^{20}$ = +86.1 (c = 1.5, $CHCl_3$).

^{1}H NMR (400 MHz, $CDCl_3$) δ 7.81 (d, J = 7.1 Hz, 2H), 7.53 (d, J = 7.5 Hz, 2H), 7.49 (d, J = 7.4 Hz, 1H), 7.42 (t, J = 7.4 Hz, 2H), 7.38 – 7.30 (m, 3H), 2.44 – 2.38 (m, 1H), 2.11 – 2.06 (m, 1H), 1.84 – 1.74 (m, 1H), 1.01 (d, J = 6.7 Hz, 3H), 0.80 (d, J = 6.6 Hz, 3H).

^{13}C NMR (101 MHz, $CDCl_3$) δ 191.7, 135.8, 134.3, 133.4, 129.8, 129.7, 128.6, 128.4, 126.0, 120.1, 56.3, 47.0, 25.7, 23.7, 23.4.

HRMS (ESI) calcd $C_{19}H_{20}NO^+$ $[M+H]^+$: 278.1539. Found: 278.1533.

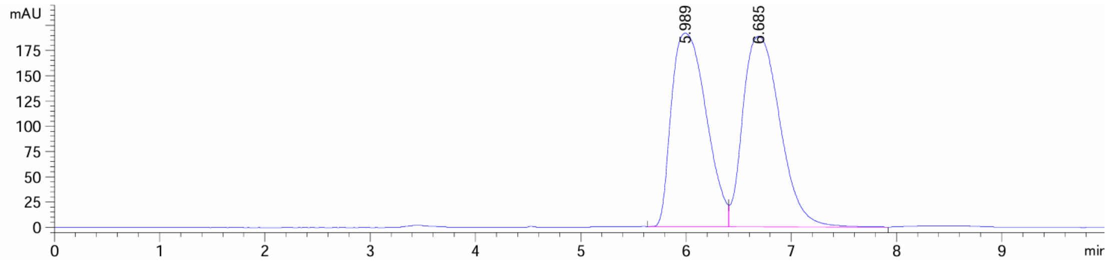

Peak #	RetTime [min]	Type	Width [min]	Area [mAU*s]	Height [mAU]	Area %
1	5.989	BV	0.3721	4401.72363	191.56970	48.7377
2	6.685	VB	0.3925	4629.73242	188.52867	51.2623

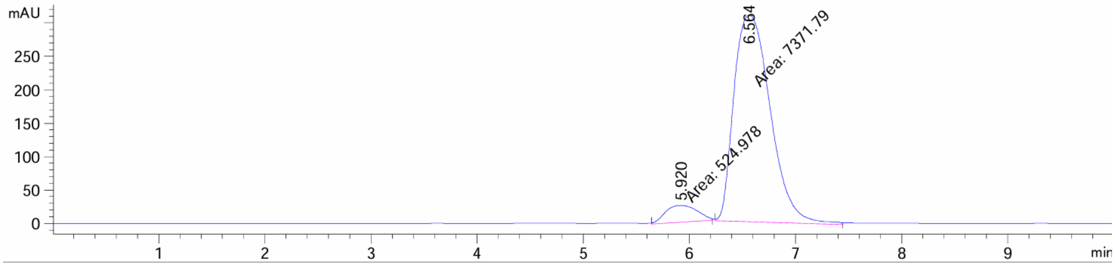

Peak #	RetTime [min]	Type	Width [min]	Area [mAU*s]	Height [mAU]	Area %
1	5.920	MM	0.3498	524.97815	25.01536	6.6480
2	6.564	MM	0.3973	7371.78955	309.24048	93.3520

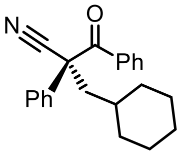

(+)-**52**

(+)-**52** was obtained as colorless oil from the desymmetric addition of **S52** using the general procedure (43.6 mg, 69% Yield). R_f = 0.6 (Hexane/EtOAc = 10:1).

HPLC analysis (Chiralpak IC-3, hexane/^{i}PrOH = 99/1, 1.0 mL/min, 254 nm; tr (minor) = 5.97 min, tr (major) = 6.83 min) gave the isomeric composition of the product: 93:7 e.r., $[\alpha]_D^{20}$ = +102.7 (c = 1.5, $CHCl_3$).

^{1}H NMR (500 MHz, $CDCl_3$) δ 7.81 (d, J = 7.9 Hz, 2H), 7.52 (d, J = 7.6 Hz, 2H), 7.48 (t, J = 7.1 Hz, 1H), 7.43 – 7.40 (m, 2H), 7.36 – 7.31 (m, 3H), 2.44 – 2.40 (m, 1H), 2.04 – 2.00 (m, 1H), 1.80 (d, J = 13.3 Hz, 1H), 1.68 (d, J = 13.4 Hz, 1H), 1.61 – 1.55 (m, 2H), 1.46 – 1.39 (m, 1H), 1.20 – 1.01 (m, 4H), 0.96 – 0.78 (m, 2H).

^{13}C NMR (101 MHz, $CDCl_3$) δ 191.7, 136.0, 134.3, 133.4, 129.8, 129.6, 128.6, 128.3, 126.0, 120.1, 56.1, 46.0, 35.1, 34.2, 33.9, 26.2, 26.1, 26.0.

HRMS (ESI) calcd $C_{22}H_{24}NO^+$ $[M+H]^+$: 318.1852. Found: 318.1855.

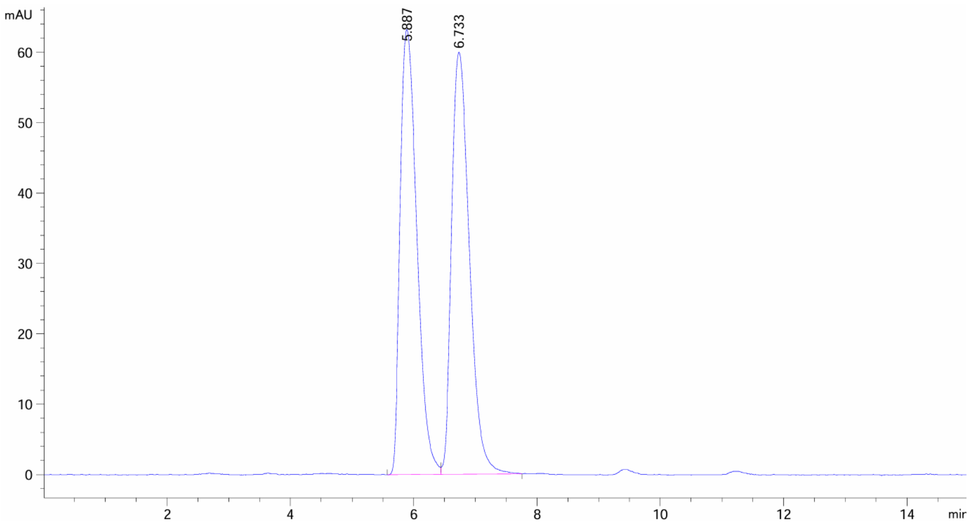

Peak #	RetTime [min]	Type	Width [min]	Area [mAU*s]	Height [mAU]	Area %
1	5.887	BV	0.3037	1190.11707	63.21268	49.7076
2	6.733	VB	0.3183	1204.11780	59.95419	50.2924

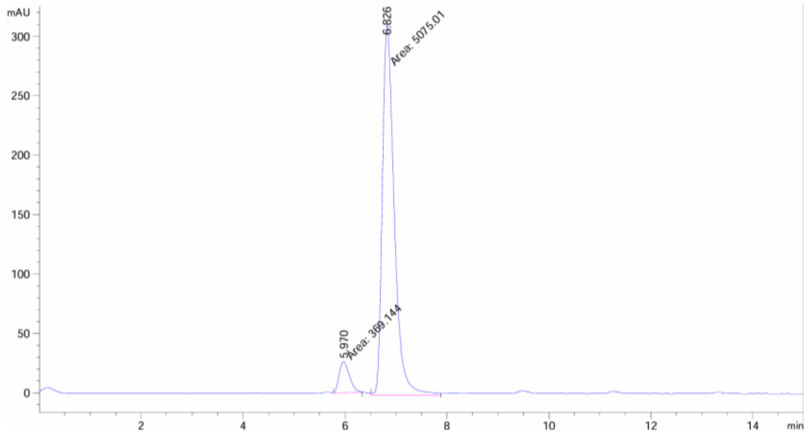

Peak #	RetTime [min]	Type	Width [min]	Area [mAU*s]	Height [mAU]	Area %
1	5.970	MM	0.2364	369.14420	26.02545	6.7806
2	6.826	MM	0.2715	5075.00928	311.54260	93.2194

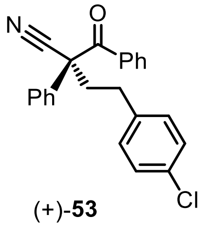

(+)-**53**

(+)-**53** was obtained as a white solid from the desymmetric addition of **S53** using the general procedure (55.2 mg, 77% Yield). R_f = 0.6 (Hexane/EtOAc = 10:1).

HPLC analysis (Chiralpak IC-3, hexane/^{i}PrOH = 99:1, 0.8 mL/min, 254 nm; t_r (minor) = 9.83 min, t_r (major) = 10.72 min) gave the isomeric composition of the product: 92:8 e.r., $[\alpha]_D^{20}$ = +83.0 (*c* = 1.0, $CHCl_3$).

^{1}H NMR (400 MHz, $CDCl_3$) δ 7.89 – 7.83 (m, 2H), 7.55 – 7.41 (m, 5H), 7.40 – 7.33 (m, 3H), 7.27 – 7.19 (m, 2H), 7.13 – 7.06 (m, 2H), 2.87 – 2.79 (m, 1H), 2.71 – 2.64 (m, 1H), 2.57 – 2.49 (m, 1H), 2.36 – 2.29 (m, 1H).

^{13}C NMR (101 MHz, $CDCl_3$) δ 190.7, 138.9, 135.3, 133.8, 133.7, 132.0, 130.0, 129.9, 129.8, 128.9, 128.6, 128.5, 125.8, 119.5, 56.6, 41.3, 30.9.

HRMS (ESI) calcd $C_{23}H_{19}ClNO^+$ $[M+H]^+$: 360.1150. Found: 360.1155.

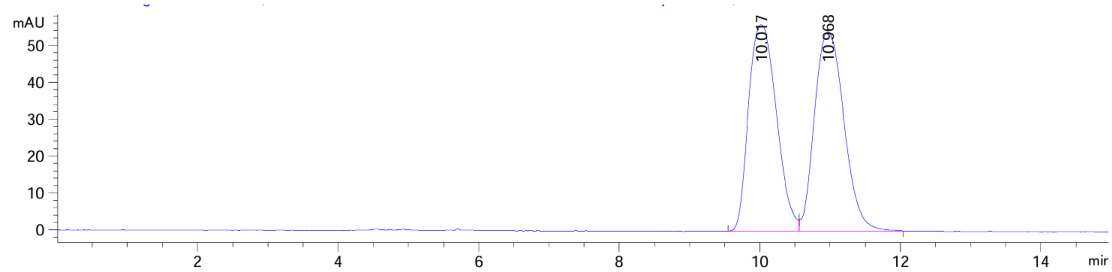

Peak #	RetTime [min]	Type	Width [min]	Area [mAU*s]	Height [mAU]	Area %
1	10.017	BV	0.4465	1537.26331	55.95144	49.4189
2	10.968	VB	0.4659	1573.41492	53.99868	50.5811

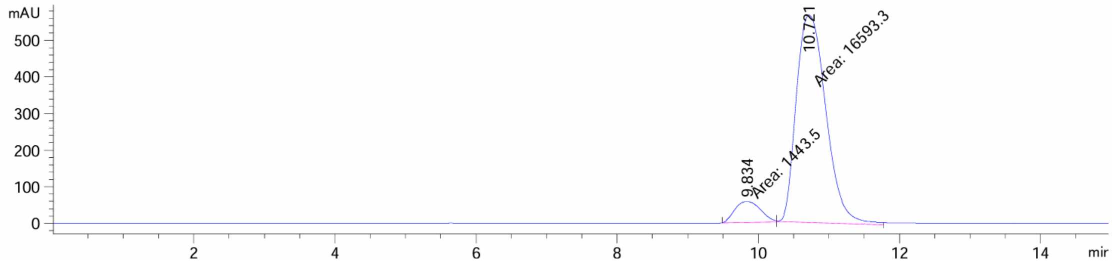

Peak #	RetTime [min]	Type	Width [min]	Area [mAU*s]	Height [mAU]	Area %
1	9.834	MM	0.4204	1443.49597	57.22712	8.0031
2	10.721	MM	0.4874	1.65933e4	567.42279	91.9969

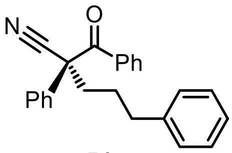

(+)-**54**

(+)-**54** was obtained as a white solid from the desymmetric addition of **S54** using the general procedure

(46.9 mg, 69% Yield). R_f = 0.6 (Hexane/EtOAc = 10:1).

HPLC analysis (Chiralpak IC-3, hexane/^{i}PrOH = 99:1, 1.0 mL/min, 254 nm; t_r (minor) = 8.01 min, t_r (major) = 9.62 min) gave the isomeric composition of the product: 91:9 e.r., $[\alpha]_D^{20}$ = +38.6 (*c* = 1.0, $CHCl_3$).

^{1}H NMR (400 MHz, $CDCl_3$) δ 7.86 – 7.79 (m, 2H), 7.50 – 7.43 (m, 3H), 7.43 – 7.37 (m, 2H), 7.37 – 7.30 (m, 3H), 7.26 – 7.22 (m, 2H), 7.20 – 7.07 (m, 3H), 2.72 – 2.57 (m, 2H), 2.47 – 2.40 (m, 1H), 2.18 – 2.10 (m, 1H), 1.95 – 1.84 (m, 1H), 1.62 – 1.51 (m, 1H).

^{13}C NMR (101 MHz, $CDCl_3$) δ 191.1, 141.2, 135.5, 134.0, 133.6, 129.9, 129.7, 128.7, 128.40, 128.35, 128.3, 125.94, 125.89, 119.6, 56.9, 38.9, 35.4, 26.5.

HRMS (ESI) calcd $C_{24}H_{21}NNaO^+$ $[M+Na]^+$: 362.1515. Found: 362.1515.

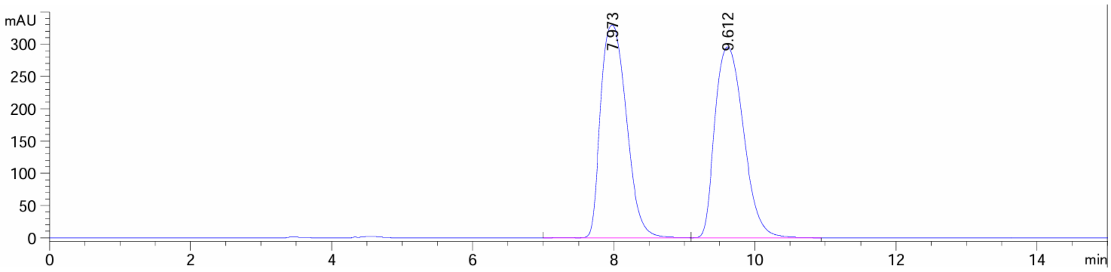

Peak #	RetTime [min]	Type	Width [min]	Area [mAU*s]	Height [mAU]	Area %
1	7.973	VB R	0.4129	8385.45020	329.52185	50.1133
2	9.612	BB	0.4575	8347.52734	295.56467	49.8867

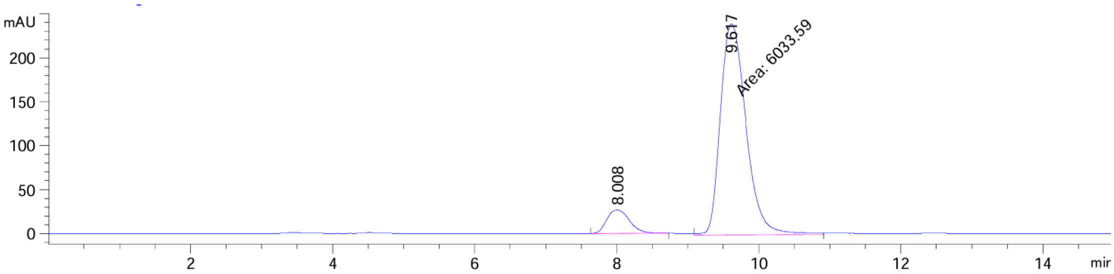

Peak #	RetTime [min]	Type	Width [min]	Area [mAU*s]	Height [mAU]	Area %
1	8.008	BB	0.3547	590.51117	26.63275	8.9146
2	9.617	MM	0.4188	6033.59180	240.10936	91.0854

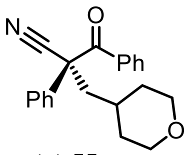

(+)-**55**

(+)-**55** was obtained as a white solid from the desymmetric addition of **S55** using the general procedure (43.5 mg, 68% Yield). R_f = 0.6 (Hexane/EtOAc = 10:1).

HPLC analysis (Chiralpak ID-3, hexane/^{i}PrOH = 99:1, 1.2 mL/min, 254 nm; t_r (minor) =22.03 min, t_r (major) = 23.95 min) gave the isomeric composition of the product: 94:6 e.r., $[\alpha]_D^{20}$ = +77.4 (*c* = 1.0, $CHCl_3$).

^{1}H NMR (400 MHz, $CDCl_3$) δ 7.83 (d, *J* = 7.2 Hz, 2H), 7.55 – 7.40 (m, 5H), 7.39 – 7.31 (m, 3H), 3.99 – 3.74 (m, 2H), 3.37 – 3.24 (m, 2H), 2.45 (dd, *J* = 14.3, 6.0 Hz, 1H), 2.10 (dd, *J* = 14.3, 5.4 Hz, 1H), 1.80 – 1.66 (m, 2H), 1.52 – 1.38 (m, 1H), 1.33 – 1.22 (m, 2H).

^{13}C NMR (101 MHz, $CDCl_3$) δ 191.2, 135.6, 134.0, 133.6, 129.9, 129.8, 128.8, 128.4, 125.9, 120.0, 67.8, 67.7, 55.8, 45.5, 33.9, 33.6, 32.5.

HRMS (ESI) calcd $C_{21}H_{22}NO_2^+$ $[M+H]^+$: 320.1645. Found: 320.1645.

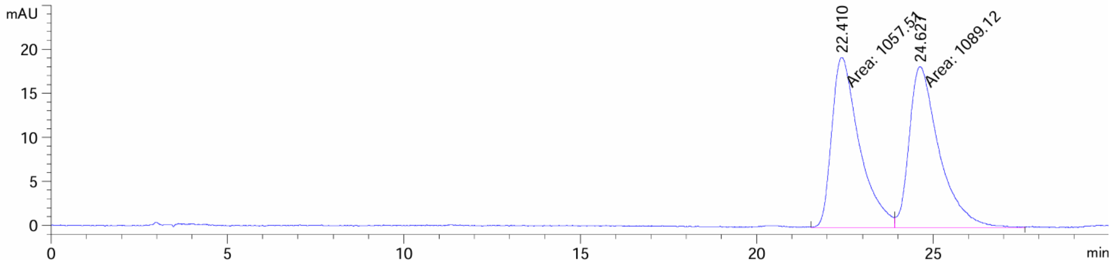

Peak #	RetTime [min]	Type	Width [min]	Area [mAU*s]	Height [mAU]	Area %
1	22.410	MF	0.9114	1057.51257	19.33815	49.2637
2	24.627	FM	0.9912	1089.12280	18.31363	50.7363

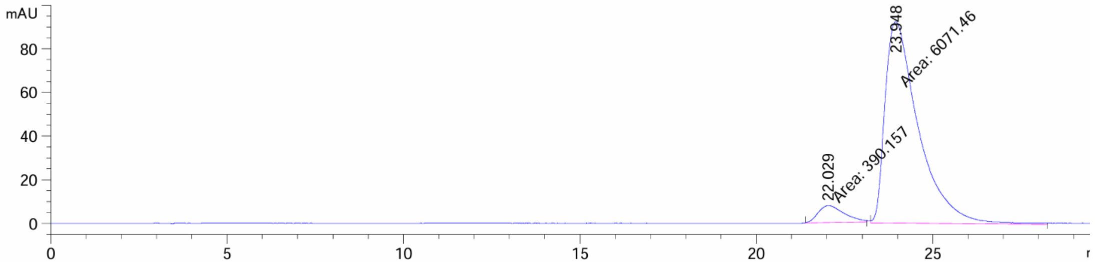

Peak #	RetTime [min]	Type	Width [min]	Area [mAU*s]	Height [mAU]	Area %
1	22.029	MM	0.8379	390.15747	7.76018	6.0381
2	23.948	MM	1.0975	6071.45605	92.20151	93.9619

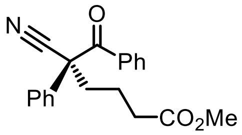

(+)-**56**

(+)-**56** was obtained as colorless oil from the desymmetric addition of **S56** using the general procedure (50.9 mg, 79% Yield). R_f = 0.6 (Hexane/EtOAc = 5:1).

HPLC analysis (Chiralpak IC-3, hexane/^{i}PrOH = 90:10, 1.0 mL/min, 254 nm; t_r (minor) = 18.55 min, t_r (major) = 32.89 min) gave the isomeric composition of the product: 92:8 e.r., $[\alpha]_D^{20}$ = +81.5 (*c* = 1.0, $CHCl_3$).

^{1}H NMR (400 MHz, $CDCl_3$) δ 7.85 – 7.83 (m, 2H), 7.50 – 7.46 (m, 3H), 7.44 – 7.40 (m, 2H), 7.39 – 7.30 (m, 3H), 3.64 (s, 3H), 2.47 – 2.27 (m, 3H), 2.20 – 2.13 (m, 1H), 1.97 – 1.79 (m, 1H), 1.65 – 1.49 (m, 1H).

^{13}C NMR (101 MHz, $CDCl_3$) δ 190.8, 173.0, 135.2, 133.8, 133.6, 129.9, 129.7, 128.8, 128.4, 125.8, 119.4, 56.6, 51.6, 38.5, 33.5, 20.4.

HRMS (ESI) calcd $C_{20}H_{20}NO_3^+$ $[M+H]^+$: 322.1438. Found: 322.1438.

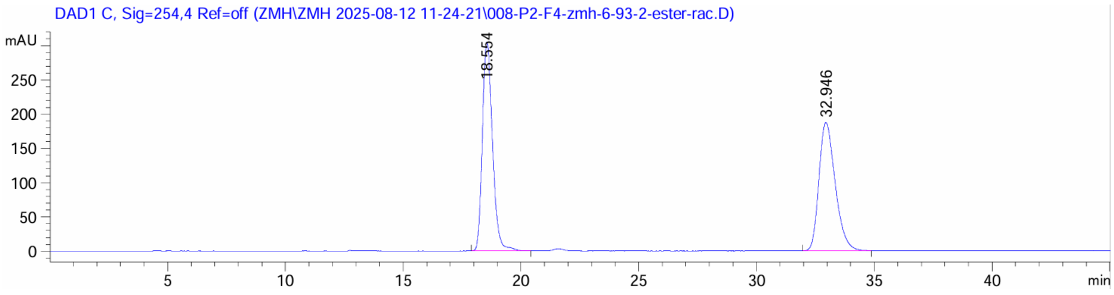

Peak #	RetTime [min]	Type	Width [min]	Area [mAU*s]	Height [mAU]	Area %
1	18.554	BB	0.4589	9125.62305	305.11700	50.3022
2	32.946	BB	0.7305	9015.97656	187.66597	49.6978

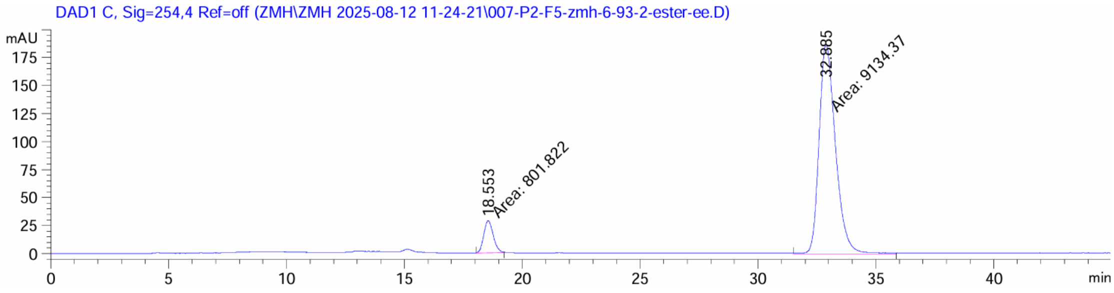

Peak #	RetTime [min]	Type	Width [min]	Area [mAU*s]	Height [mAU]	Area %
1	18.553	MM	0.4658	801.82184	28.68958	8.0697
2	32.885	MM	0.8138	9134.36914	187.07657	91.9303

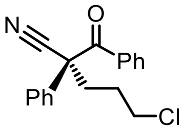

(+)-**57**

(+)-**57** was obtained as colorless oil from the desymmetric addition of **S57** using the general procedure (55.7 mg, 94% Yield). R_f = 0.7 (Hexane/EtOAc = 10:1).

HPLC analysis (Chiralpak IC-3, hexane/^{i}PrOH = 99:1, 1.0 mL/min, 254 nm; t_r (minor) = 8.64 min, t_r (major) = 10.01 min) gave the isomeric composition of the product: 85:15 e.r., $[\alpha]_D^{20}$ = +84.6 (*c* = 1.0, $CHCl_3$).

^{1}H NMR (400 MHz, $CDCl_3$) δ 7.85 (d, *J* = 7.1 Hz, 2H), 7.53 – 7.40 (m, 5H), 7.40 – 7.31 (m, 3H), 3.54 (t, *J* = 6.4 Hz, 2H), 2.57 – 2.49 (m, 1H), 2.33 – 2.25 (m, 1H), 2.10 – 2.00 (m, 1H), 1.77 – 1.66 (m, 1H).

^{13}C NMR (101 MHz, $CDCl_3$) δ 190.7, 135.2, 133.7, 133.7, 130.0, 129.8, 128.9, 128.5, 125.8, 119.3, 56.3, 44.1, 37.0, 28.2.

HRMS (ESI) calcd $C_{18}H_{17}ClNO^+$ $[M+H]^+$: 298.0993. Found: 298.0993.

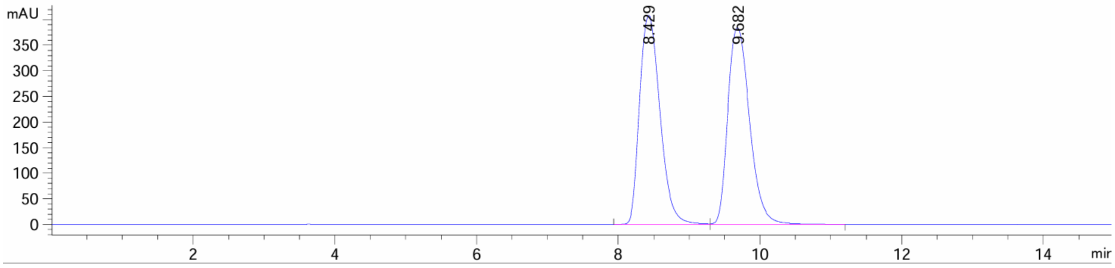

Peak #	RetTime [min]	Type	Width [min]	Area [mAU*s]	Height [mAU]	Area %
1	8.429	BV	0.3161	8192.55273	408.28601	49.9145
2	9.682	VB	0.3375	8220.60645	384.44672	50.0855

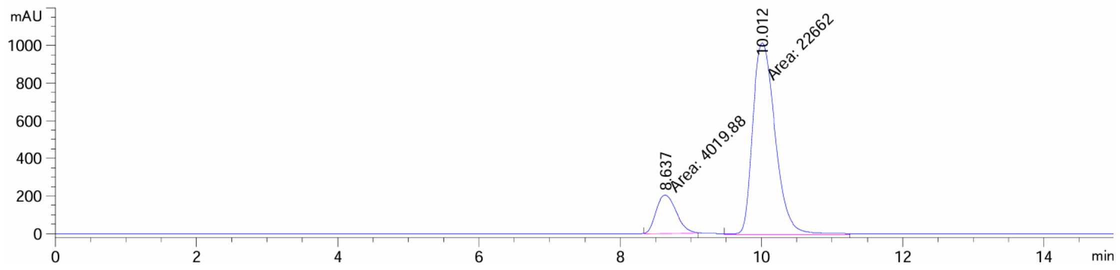

Peak #	RetTime [min]	Type	Width [min]	Area [mAU*s]	Height [mAU]	Area %
1	8.637	MM	0.3299	4019.88281	203.07307	15.0660
2	10.012	MM	0.3709	2.26620e4	1018.31116	84.9340

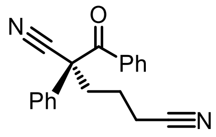

(+)-**58**

(+)-**58** was obtained as colorless oil from the desymmetric addition of **S58** using the general procedure (33.1 mg, 57% Yield). R_f = 0.5 (Hexane/EtOAc = 5:1).

HPLC analysis (Chiralpak IC-3, hexane/^{i}PrOH = 90:10, 1.0 mL/min, 254 nm; t_r (minor) = 42.37 min, t_r (major) = 44.75 min) gave the isomeric composition of the product: 92:8 e.r., $[\alpha]_D^{20}$ = +54.4 (c = 1.0, $CHCl_3$).

^{1}H NMR (400 MHz, $CDCl_3$) δ 7.85 (d, J = 7.1 Hz, 2H), 7.55 – 7.28 (m, 8H), 2.55 – 2.48 (m, 1H), 2.41 – 2.39 (m, 2H), 2.29 – 2.22 (m, 1H), 2.03 – 1.84 (m, 1H), 1.68 – 1.57 (m, 1H).

^{13}C NMR (101 MHz, $CDCl_3$) δ 190.3, 134.8, 133.9, 133.5, 130.03, 130.00, 129.1, 128.5, 125.7, 119.0, 118.7, 56.2, 38.3, 21.3, 17.0.

HRMS (ESI) calcd $C_{19}H_{17}N_2O^+$ $[M+H]^+$: 289.1335. Found: 289.1335.

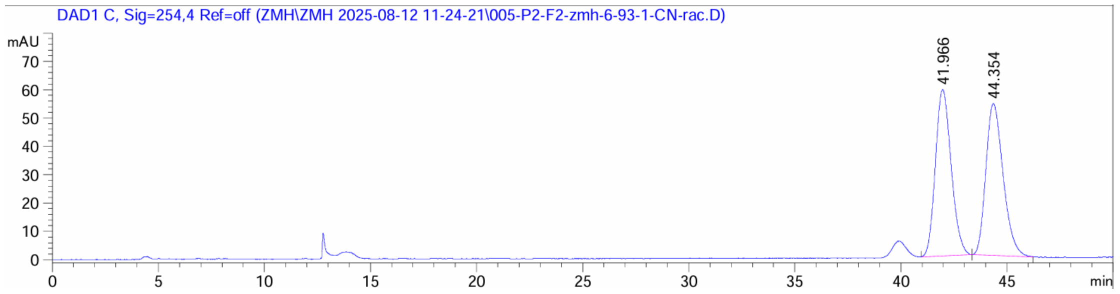

Peak #	RetTime [min]	Type	Width [min]	Area [mAU*s]	Height [mAU]	Area %
1	41.966	BB	0.7521	3028.70068	58.68650	49.9255
2	44.354	BB	0.7888	3037.73633	53.62830	50.0745

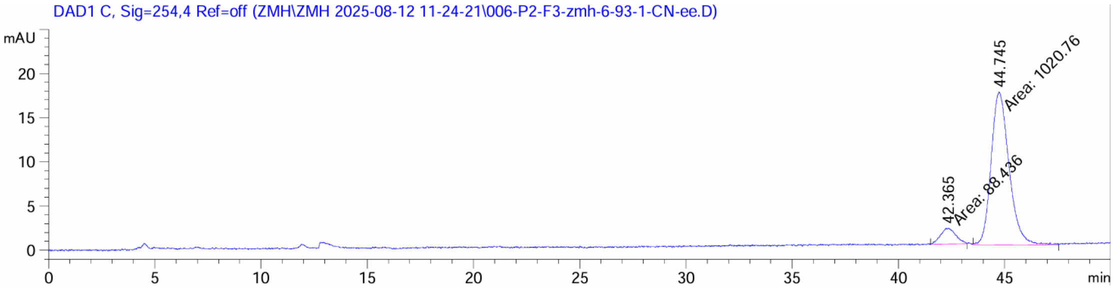

Peak #	RetTime [min]	Type	Width [min]	Area [mAU*s]	Height [mAU]	Area %
1	42.365	MM	0.8292	88.43602	1.77754	7.9730
2	44.745	MM	0.9831	1020.75818	17.30489	92.0270

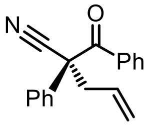

(+)-**59**

(+)-**59** was obtained as colorless oil from the desymmetric addition of **S59** using the general procedure (47.1 mg, 90% Yield). R_f = 0.8 (Hexane/EtOAc = 10:1).

HPLC analysis (Chiralpak IC-3, hexane/^{i}PrOH = 99:1, 1.0 mL/min, 254 nm; t_r (minor) = 6.90 min, t_r (major) = 7.51 min) gave the isomeric composition of the product: 94:6 e.r., $[\alpha]_D^{20}$ = +86.1 (*c* = 1.0, $CHCl_3$).

^{1}H NMR (400 MHz, $CDCl_3$) δ 7.88 – 7.81 (m, 2H), 7.53 – 7.39 (m, 5H), 7.39 – 7.30 (m, 3H), 5.77 – 5.56 (m, 1H), 5.23 – 5.11 (m, 2H), 3.14 (dd, *J* = 13.9, 7.1 Hz, 1H), 2.84 (dd, *J* = 13.9, 7.4 Hz, 1H).

^{13}C NMR (101 MHz, $CDCl_3$) δ 190.7, 135.0, 133.8, 133.7, 131.1, 130.0, 129.6, 128.7, 128.4, 126.0, 120.9, 119.1, 57.0, 43.7.

HRMS (ESI) calcd $C_{18}H_{16}NO^+$ $[M+H]^+$: 262.1226. Found: 262.1226.

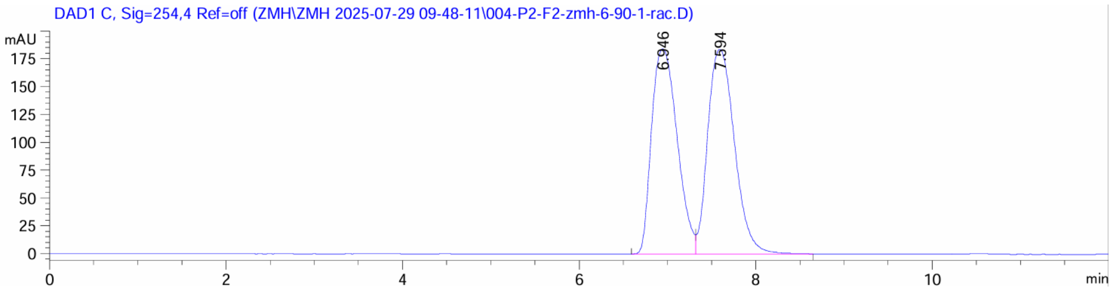

Peak #	RetTime [min]	Type	Width [min]	Area [mAU*s]	Height [mAU]	Area %
1	6.946	BV	0.3228	3705.59863	184.16174	48.8390
2	7.594	VB	0.3344	3881.77686	183.79503	51.1610

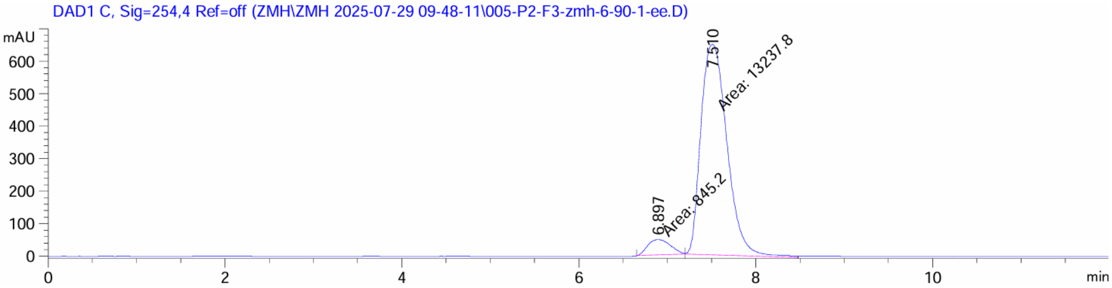

Peak #	RetTime [min]	Type	Width [min]	Area [mAU*s]	Height [mAU]	Area %
1	6.897	MM	0.2983	845.19989	47.22010	6.0016
2	7.510	MM	0.3405	1.32378e4	647.94073	93.9984

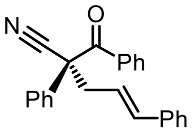

(+)-**60**

(+)-**60** was obtained as a white solid from the desymmetric addition of **S60** using the general procedure (37.9 mg, 56% Yield). R_f = 0.7 (Hexane/EtOAc = 5:1).

HPLC analysis (Chiralpak IC-3, hexane/^{i}PrOH = 99:1, 1.0 mL/min, 254 nm; t_r (minor) = 9.08 min, t_r (major) = 13.72 min) gave the isomeric composition of the product: 93:7 e.r., $[\alpha]_D^{20}$ = +112.6 (c = 1.0, $CHCl_3$).

^{1}H NMR (400 MHz, $CDCl_3$) δ 7.86 (d, J = 7.8 Hz, 2H), 7.51 – 7.27 (m, 11H), 7.26 – 7.16 (m, 2H), 6.47 (d, J = 15.8 Hz, 1H), 6.10 – 6.02 (m, 1H), 3.29 (dd, J = 14.0, 7.4 Hz, 1H), 2.98 (dd, J = 14.0, 7.6 Hz, 1H).

^{13}C NMR (101 MHz, $CDCl_3$) δ 190.8, 136.8, 135.8, 135.1, 133.8, 133.7, 130.0, 129.7, 128.8, 128.4, 127.6, 126.4, 126.0, 122.4, 119.2, 57.3, 43.0.

HRMS (ESI) calcd $C_{24}H_{20}NO^+$ [M+H]$^+$: 338.1539. Found: 338.1539.

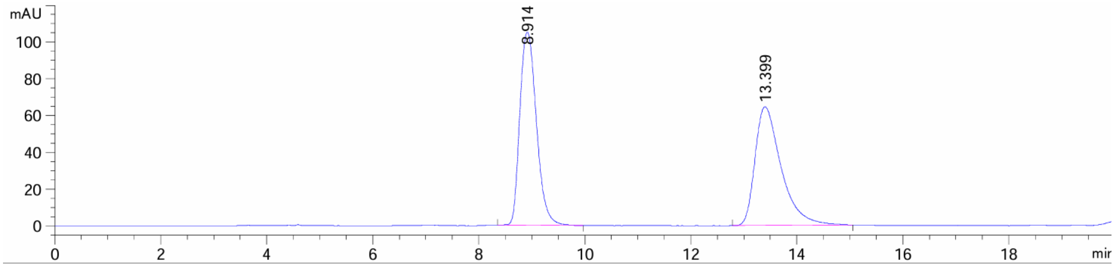

Peak #	RetTime [min]	Type	Width [min]	Area [mAU*s]	Height [mAU]	Area %
1	8.914	BB	0.3450	2282.03076	105.22998	50.7256
2	13.399	BB	0.5198	2216.74536	64.69152	49.2744

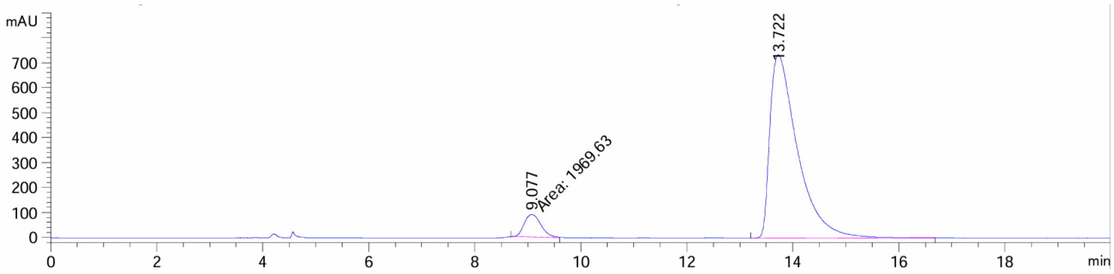

Peak #	RetTime [min]	Type	Width [min]	Area [mAU*s]	Height [mAU]	Area %
1	9.077	MM	0.3649	1969.62781	89.96652	6.9008
2	13.722	BB	0.5402	2.65724e4	734.23590	93.0992

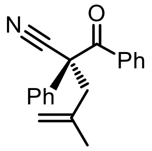

(+)-**61**

(+)-**61** was obtained as a white solid from the desymmetric addition of **S61** using the general procedure (39.1 mg, 71% Yield). R_f = 0.7 (Hexane/EtOAc = 10:1).

HPLC analysis (Chiralpak IC-3, hexane/^{i}PrOH = 99:1, 1.0 mL/min, 254 nm; t_r (minor) = 6.54 min, t_r (major) = 7.18 min) gave the isomeric composition of the product: 95:5 e.r., $[\alpha]_D^{20}$ = +74.1 (c = 1.0, $CHCl_3$).

^{1}H NMR (400 MHz, $CDCl_3$) δ 7.84 (d, J = 7.7 Hz, 2H), 7.54 – 7.30 (m, 8H), 4.93 (d, J = 1.4 Hz, 1H), 4.75 (s, 1H), 3.17 (d, J = 14.1 Hz, 1H), 2.86 (d, J = 14.1 Hz, 1H), 1.51 (s, 3H).

^{13}C NMR (101 MHz, $CDCl_3$) δ 191.0, 139.1, 135.1, 134.0, 133.5, 129.9, 129.6, 128.7, 128.4, 126.2, 119.6, 117.0, 56.7, 46.3, 23.7.

HRMS (ESI) calcd $C_{19}H_{18}NO^+$ $[M+H]^+$: 276.1383. Found: 276.1384.

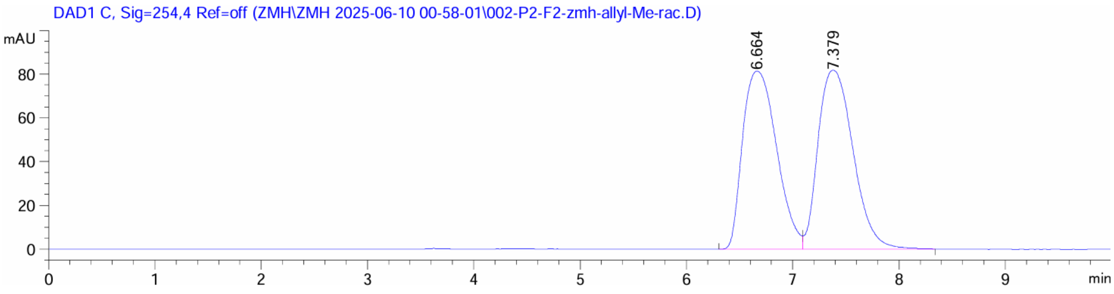

Peak #	RetTime [min]	Type	Width [min]	Area [mAU*s]	Height [mAU]	Area %
1	6.664	BV	0.3596	1810.77502	81.38798	49.1202
2	7.379	VB	0.3655	1875.64294	81.80642	50.8798

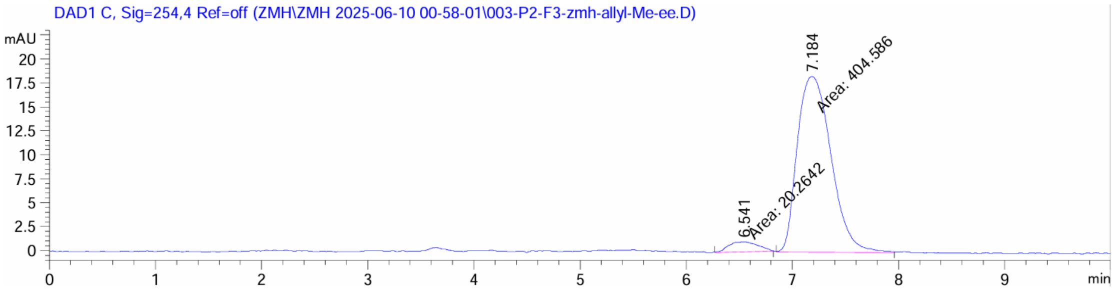

Peak #	RetTime [min]	Type	Width [min]	Area [mAU*s]	Height [mAU]	Area %
1	6.541	MM	0.3264	20.26420	1.03468	4.7697
2	7.184	MM	0.3682	404.58582	18.31161	95.2303

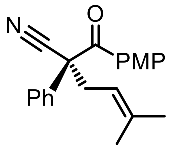

(+)-**62**

(+)-**62** was obtained as colorless oil from the desymmetric addition of **S62** using the general procedure (39.2 mg, 61% Yield). R_f = 0.8 (Hexane/EtOAc = 10:1).

HPLC analysis (Chiralpak ID-3, hexane/^{i}PrOH = 97:3, 1.0 mL/min, 254 nm; t_r (minor) = 8.07 min, t_r (major) = 9.45 min) gave the isomeric composition of the product: 91:9 e.r., $[\alpha]_D^{20}$ = +160.4 (*c* = 1.0, $CHCl_3$).

^{1}H NMR (500 MHz, $CDCl_3$) δ 7.86 (d, *J* = 9.0 Hz, 2H), 7.48 – 7.41 (m, 2H), 7.40 – 7.37 (dd, *J* = 8.6, 6.6 Hz, 2H), 7.36 – 7.29 (m, 1H), 6.80 (d, *J* = 9.0 Hz, 2H), 5.15 – 5.04 (m, 1H), 3.80 (s, 3H), 3.07 (dd, *J* = 14.4, 7.2 Hz, 1H), 2.79 (dd, *J* = 14.4, 7.9 Hz, 1H), 1.67 (s, 3H), 1.46 (s, 3H).

^{13}C NMR (126 MHz, CDCl$_3$) δ 189.5, 163.7, 137.7, 136.0, 132.6, 129.4, 128.3, 126.6, 126.1, 119.9, 116.9, 113.6, 56.6, 55.4, 38.2, 25.9, 17.9.

HRMS (ESI) calcd $C_{21}H_{22}NO_2^+$ [M+H]$^+$: 320.1645. Found: 320.1645.

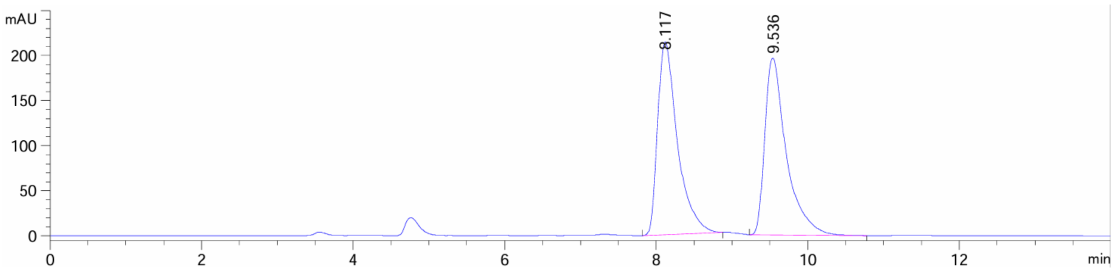

Peak #	RetTime [min]	Type	Width [min]	Area [mAU*s]	Height [mAU]	Area %
1	8.117	BB	0.2741	3887.38452	213.84151	50.1490
2	9.536	BB	0.2920	3864.27808	195.99614	49.8510

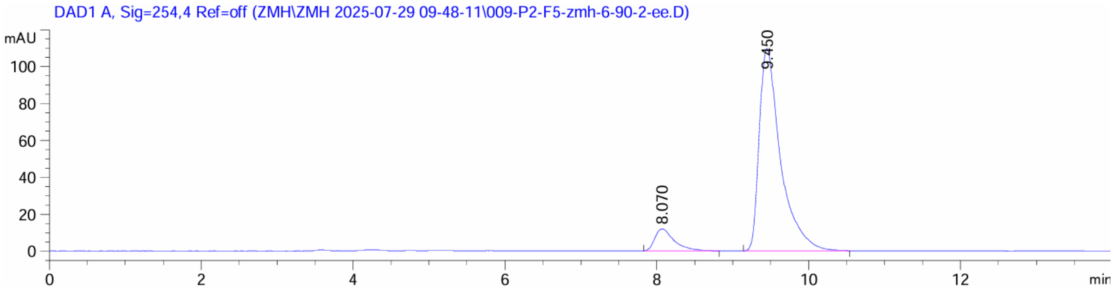

Peak #	RetTime [min]	Type	Width [min]	Area [mAU*s]	Height [mAU]	Area %
1	8.070	BB	0.2582	210.47345	11.91055	8.9855
2	9.450	BB	0.2884	2131.89966	109.87308	91.0145

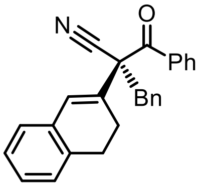

(–)-**63**

(+)-**63** was obtained as a white solid from the desymmetric addition of **S63** using the general procedure (46.6 mg, 64% Yield). R_f = 0.5 (Hexane/EtOAc = 20:1).

HPLC analysis (Chiralpak IC-3, hexane/^{i}PrOH = 99:1, 1.0 mL/min, 254 nm; t_r (minor) = 10.84 min, t_r (major) = 12.37 min) gave the isomeric composition of the product: 92:8 e.r., $[\alpha]_D^{20}$ = –33.5 (*c* = 1.0, CHCl$_3$).

^{1}H NMR (500 MHz, CDCl$_3$) δ 8.13 – 8.05 (m, 2H), 7.58 – 7.51 (m, 1H), 7.44 – 7.38 (m, 2H), 7.27 (t,

J = 2.6 Hz, 5H), 7.18 – 7.16 (m, 2H), 7.09 – 7.08 (m, 1H), 7.04 – 7.02 (m, 1H), 6.64 (s, 1H), 3.57 (d, *J* = 13.8 Hz, 1H), 3.34 (d, *J* = 13.8 Hz, 1H), 2.82 – 2.76 (m, 1H), 2.68 – 2.62 (m, 1H), 2.48 – 2.41 (m, 1H), 2.07 – 2.01 (m, 1H).

^{13}C NMR (126 MHz, $CDCl_3$) δ 191.3, 134.4, 134.3, 134.0, 133.2, 132.8, 130.8, 129.5, 128.6, 128.3, 128.20, 128.17, 127.5, 127.3, 127.1, 126.8, 118.8, 59.3, 40.9, 27.7, 24.2

HRMS (ESI) calcd $C_{26}H_{22}NO^+$ $[M+H]^+$: 364.1696. Found: 364.1696.

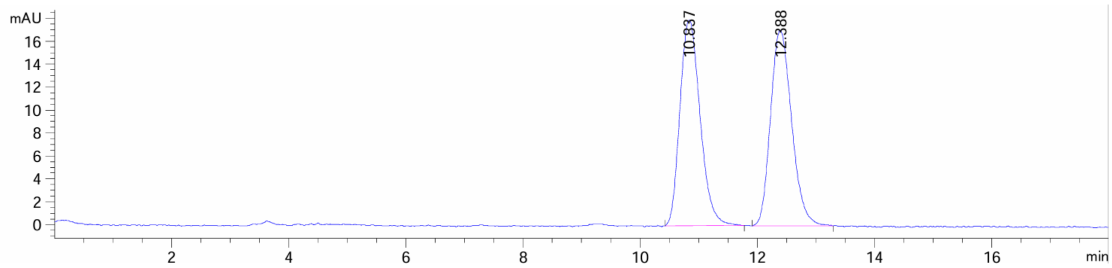

Peak #	RetTime [min]	Type	Width [min]	Area [mAU*s]	Height [mAU]	Area %
1	10.837	BB	0.3729	428.64493	17.93031	50.0729
2	12.388	BB	0.3923	427.39639	17.06116	49.9271

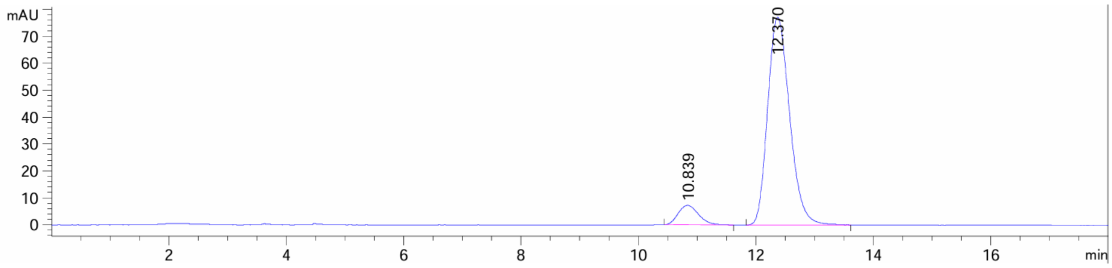

Peak #	RetTime [min]	Type	Width [min]	Area [mAU*s]	Height [mAU]	Area %
1	10.839	BB	0.3735	174.74060	7.24238	8.0203
2	12.370	BB	0.4028	2003.99133	77.22188	91.9797

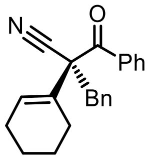

(+)-**64**

(+)-**64** was obtained as colorless oil from the desymmetric addition of **S64** using the general procedure (43.7 mg, 69% Yield). R_f = 0.5 (Hexane/EtOAc = 10:1).

HPLC analysis (Chiralpak IC-3, hexane/^{i}PrOH = 99/1, 1.0 mL/min, 254 nm; tr (minor) = 10.76 min, tr (major) = 14.15 min) gave the isomeric composition of the product: 86:14 e.r., $[\alpha]_D^{20}$ = +67.0 (c = 1.0, $CHCl_3$).

^{1}H NMR (400 MHz, $CDCl_3$) δ 8.06 (d, J = 7.8 Hz, 2H), 7.57 (t, J = 7.4 Hz, 1H), 7.43 (t, J = 7.7 Hz, 2H), 7.34 – 7.22 (m, 5H), 5.85 (t, J = 3.8 Hz, 1H), 3.39 (d, J = 13.7 Hz, 1H), 3.31 (d, J = 13.8 Hz, 1H), 2.36 – 1.82 (m, 4H), 1.57 – 1.40 (m, 4H).

^{13}C NMR (101 MHz, $CDCl_3$) δ 191.7, 134.7, 134.5, 133.7, 131.4, 130.8, 129.3, 129.0, 128.5, 128.0, 127.3, 119.2, 59.3, 40.8, 25.5, 25.4, 22.4, 21.4.

HRMS (ESI) calcd $C_{22}H_{22}NO^+$ $[M+H]^+$: 316.1696. Found: 316.1695.

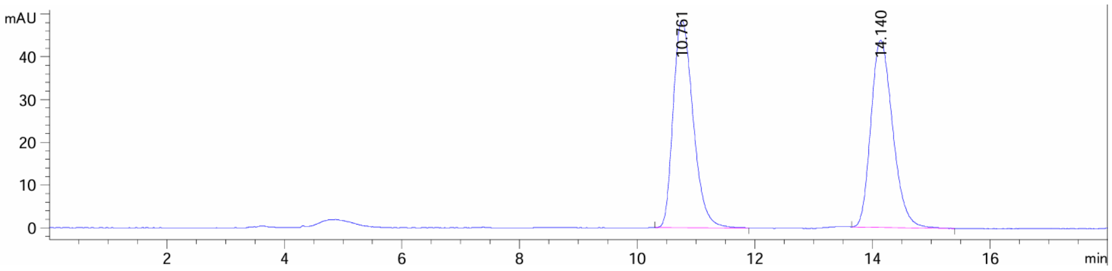

Peak #	RetTime [min]	Type	Width [min]	Area [mAU*s]	Height [mAU]	Area %
1	10.761	BB	0.3671	1144.02930	48.51808	50.3520
2	14.140	BB	0.3989	1128.03613	43.73803	49.6480

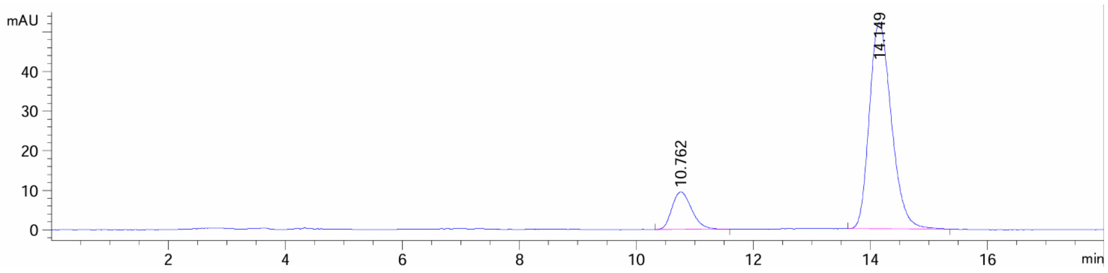

Peak #	RetTime [min]	Type	Width [min]	Area [mAU*s]	Height [mAU]	Area %
1	10.762	BB	0.3361	221.19281	9.48567	13.9532
2	14.149	BB	0.4052	1364.05249	52.15052	86.0468

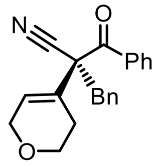

(+)-**65**

(+)-**65** was obtained as colorless oil from the desymmetric addition of **S65** using the general procedure (46.7 mg, 74% Yield). R_f = 0.6 (Hexane/EtOAc = 5:1).

HPLC analysis (Chiralpak IC-3, hexane/^{i}PrOH = 95/5, 1.0 mL/min, 254 nm; tr (minor) = 12.12 min, tr (major) = 15.88 min) gave the isomeric composition of the product: 85:15 e.r., $[\alpha]_D^{20}$ = +76.3 (*c* = 1.5, $CHCl_3$).

^{1}H NMR (400 MHz, $CDCl_3$) δ 8.04 (d, *J* = 7.1 Hz, 2H), 7.58 (t, *J* = 7.4 Hz, 1H), 7.44 (t, *J* = 7.8 Hz, 2H), 7.38 – 7.20 (m, 5H), 5.88 – 5.86 (m, 1H), 4.24 – 4.18 (m, 1H), 4.09 – 4.03 (m, 1H), 3.76 – 3.71 (m, 1H), 3.67 – 3.62 (m, 1H), 3.44 (d, *J* = 13.8 Hz, 1H), 3.32 (d, *J* = 13.8 Hz, 1H), 2.36 – 2.19 (m, 1H), 1.82 – 1.67 (m, 1H).

^{13}C NMR (101 MHz, $CDCl_3$) δ 191.0, 134.3, 134.2, 134.0, 130.7, 129.7, 129.3, 128.6, 128.1, 127.6, 127.2, 118.7, 65.2, 63.7, 58.6, 40.6, 25.4.

HRMS (ESI) calcd $C_{21}H_{20}NO_2^+$ $[M+H]^+$: 318.1489. Found: 318.1488.

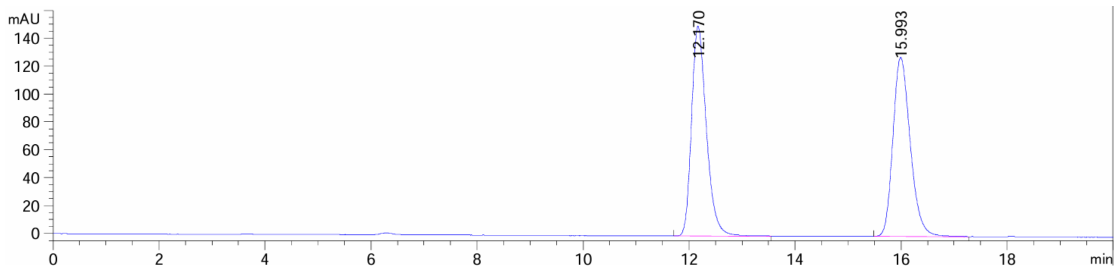

Peak #	RetTime [min]	Type	Width [min]	Area [mAU*s]	Height [mAU]	Area %
1	12.170	BB	0.2991	2927.78076	150.38466	50.0861
2	15.993	BB	0.3511	2917.71802	128.41664	49.9139

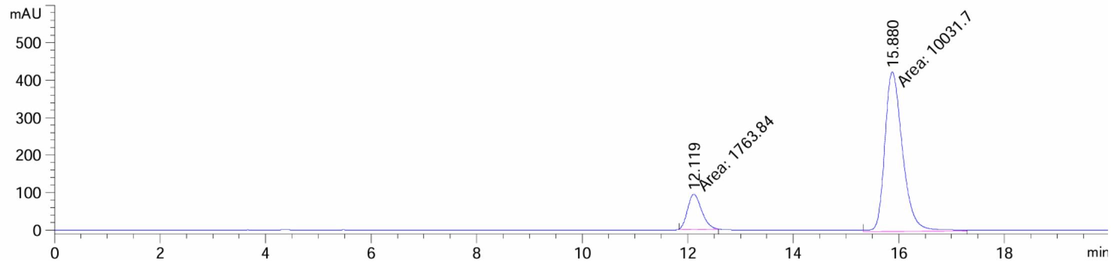

Peak #	RetTime [min]	Type	Width [min]	Area [mAU*s]	Height [mAU]	Area %
1	12.119	MM	0.3113	1763.84192	94.42163	14.9534
2	15.880	MM	0.3925	1.00317e4	425.97818	85.0466

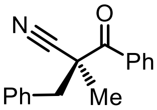

(+)-**66**

(+)-**66** was obtained as colorless oil from the desymmetric addition of **S66** using the general procedure (40.6 mg, 82% Yield). R_f = 0.6 (Hexane/EtOAc = 10:1).

HPLC analysis (Chiralpak IC-3, hexane/^{i}PrOH = 99/1, 1.0 mL/min, 254 nm; tr (major) = 8.08 min, tr (major) = 8.87 min) gave the isomeric composition of the product: 54:46 e.r. $[\alpha]_D^{20}$ = +7.9 (c = 1.0, $CHCl_3$).

^{1}H NMR (400 MHz, $CDCl_3$) δ 7.97 (d, J = 7.1 Hz, 2H), 7.57 (t, J = 7.4 Hz, 1H), 7.46 – 7.42 (m, 2H), 7.36 – 7.26 (m, 5H), 3.48 (d, J = 13.6 Hz, 1H), 3.09 (d, J = 13.6 Hz, 1H), 1.68 (s, 3H).

^{13}C NMR (101 MHz, $CDCl_3$) δ 194.8, 134.7, 134.3, 133.5, 130.4, 129.1, 128.54, 128.51, 127.8, 121.6, 47.5, 43.8, 24.0.

HRMS (ESI) calcd $C_{17}H_{16}NO^+$ $[M+H]^+$: 250.1226. Found: 250.1226.

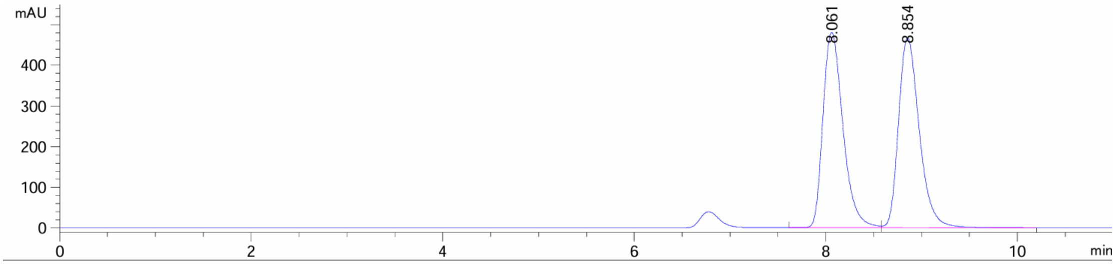

Peak #	RetTime [min]	Type	Width [min]	Area [mAU*s]	Height [mAU]	Area %
1	8.061	BV	0.2250	6963.63037	480.63824	49.7732
2	8.854	VB	0.2333	7027.08057	467.57220	50.2268

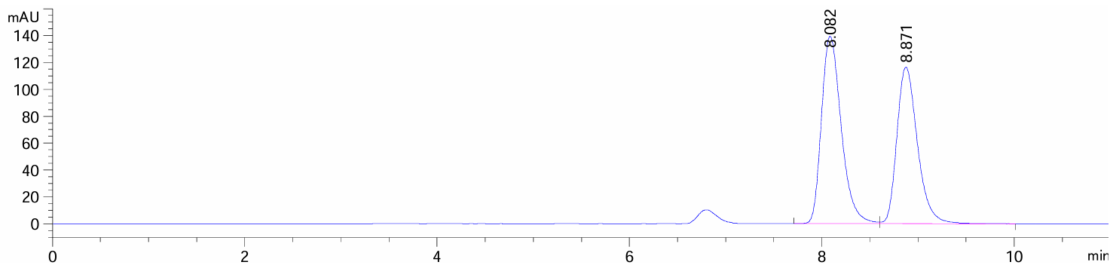

Peak #	RetTime [min]	Type	Width [min]	Area [mAU*s]	Height [mAU]	Area %
1	8.082	BV	0.2234	2000.41467	139.41350	53.6563
2	8.871	VB	0.2290	1727.78442	116.50062	46.3437

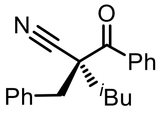

(+)-**67**

(+)-**67** was obtained as colorless oil from the desymmetric addition of **S67** using the general procedure (26.9 mg, 46% Yield). R_f = 0.5 (Hexane/EtOAc = 10:1).

HPLC analysis (Chiralpak ID-3, hexane/^{i}PrOH = 99:1, 1.0 mL/min, 254 nm; t_r (minor) = 8.00 min, t_r (major) = 10.80 min) gave the isomeric composition of the product: 61:39 e.r., $[\alpha]_D^{20}$ = +8.97 (*c* = 1.0, $CHCl_3$).

^{1}H NMR (400 MHz, $CDCl_3$) δ 7.56 – 7.42 (m, 3H), 7.35 – 7.23 (m, 7H), 3.41 (d, *J* = 13.3 Hz, 1H), 3.12 (d, *J* = 13.3 Hz, 1H), 2.40 – 2.26 (m, 1H), 1.99 – 1.79 (m, 2H), 1.00 (d, *J* = 6.5 Hz, 3H), 0.86 (d, *J* = 6.5 Hz, 3H).

^{13}C NMR (101 MHz, $CDCl_3$) δ 197.32, 136.64, 134.32, 132.85, 130.44, 128.65, 128.09, 127.85, 121.43, 53.43, 47.04, 45.98, 26.19, 23.45, 23.32.

HRMS (ESI) calcd $C_{20}H_{22}NO^+$ $[M+H]^+$: 292.1696. Found: 292.1693.

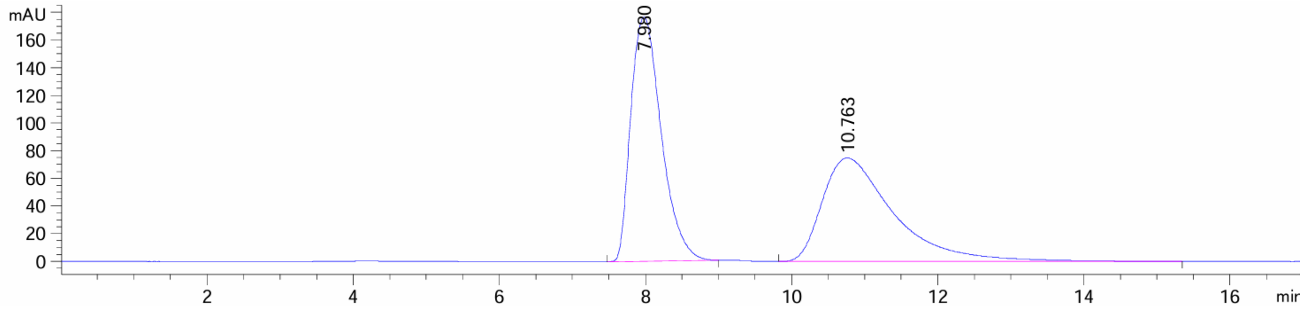

Peak #	RetTime [min]	Type	Width [min]	Area [mAU*s]	Height [mAU]	Area %
1	7.980	BB	0.4589	5123.53906	176.37720	50.1430
2	10.763	BB	0.9956	5094.32471	74.89396	49.8570

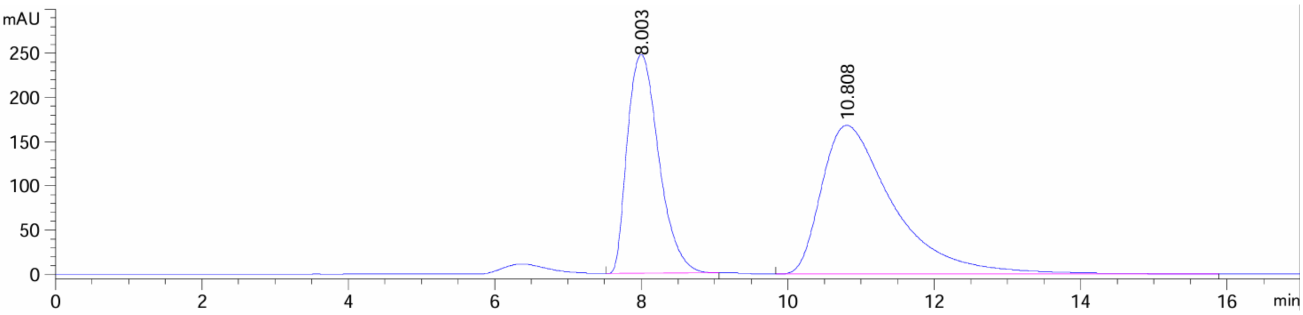

Peak #	RetTime [min]	Type	Width [min]	Area [mAU*s]	Height [mAU]	Area %
1	8.003	BB	0.4769	7399.89209	247.41527	39.1458
2	10.808	BB	1.0139	1.15035e4	168.20296	60.8542

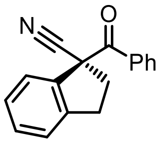

(+)-**68**

(+)-**68** was obtained as colorless oil from the desymmetric addition of **S68** using the general procedure (38.2 mg, 77% Yield). R_f = 0.6 (Hexane/EtOAc = 5:1).

HPLC analysis (Chiralpak IC-3, hexane/^{i}PrOH = 95/5, 1.0 mL/min, 254 nm; tr (minor) = 16.86 min, tr (major) = 17.72 min) gave the isomeric composition of the product: 76:24 e.r., $[\alpha]_D^{20}$ = +57.2 (*c* = 1.0, $CHCl_3$).

^{1}H NMR (400 MHz, $CDCl_3$) δ 8.03 (d, *J* = 7.1 Hz, 2H), 7.60 (t, *J* = 7.4 Hz, 1H), 7.48 (t, *J* = 7.8 Hz, 2H), 7.41 (d, *J* = 7.6 Hz, 1H), 7.37 – 7.32 (m, 2H), 7.31 – 7.27 (m, 1H), 3.29 – 3.08 (m, 2H), 3.04 – 2.87 (m, 2H).

^{13}C NMR (101 MHz, $CDCl_3$) δ 191.9, 143.5, 138.3, 133.8, 133.7, 129.7, 129.6, 128.7, 127.6, 125.6,

125.3, 120.7, 57.1, 37.8, 31.1.

HRMS (ESI) calcd $C_{17}H_{14}NO^+$ $[M+H]^+$: 248.1070. Found: 248.1070.

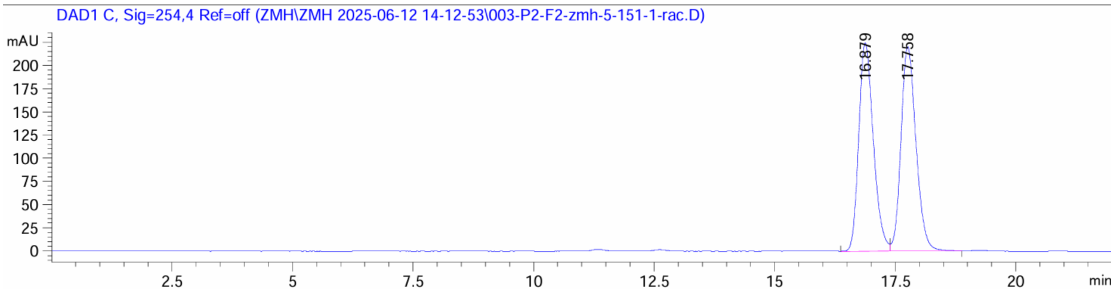

Peak #	RetTime [min]	Type	Width [min]	Area [mAU*s]	Height [mAU]	Area %
1	16.879	BV	0.3252	4721.47900	224.63312	49.6197
2	17.758	VB	0.3360	4793.85889	220.23160	50.3803

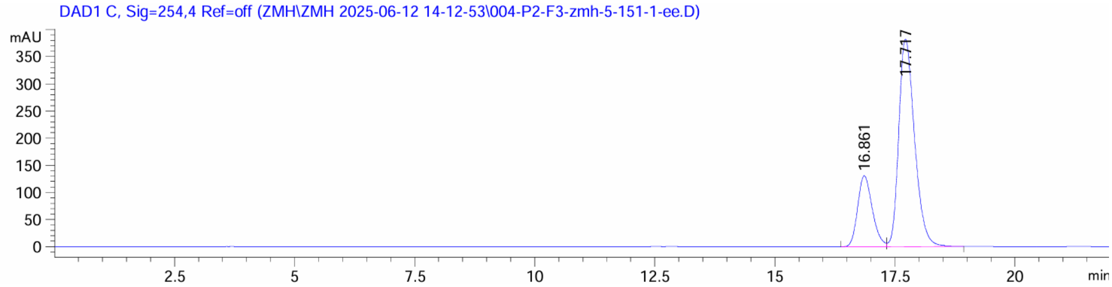

Peak #	RetTime [min]	Type	Width [min]	Area [mAU*s]	Height [mAU]	Area %
1	16.861	BV	0.3265	2764.46924	130.82518	23.9657
2	17.717	VB	0.3537	8770.64648	382.17627	76.0343

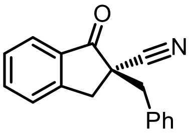

(+)-**69**

(+)-**69** was obtained as colorless oil from the desymmetric addition of **S69** using the general procedure (33.2 mg, 67% Yield). R_f = 0.2 (Hexane/EtOAc = 10:1). The characterization data matches those reported in literature.[5]

HPLC analysis (Chiralpak AD-H, hexane/^{i}PrOH = 95:5, 1.0 mL/min, 254 nm; t_r (minor) = 15.30 min, t_r (major) = 21.38 min) gave the isomeric composition of the product: 67:33 e.r., $[\alpha]_D^{20}$ = +68.5 (*c* = 1.0, $CHCl_3$).

[5] Chen, Z. H., Sun, R. Z., Yao, F., Hu, X. D., Xiang, L. X., Cong, H., Liu, W. B. Enantioselective nickel-catalyzed reductive aryl/alkenyl-cyano cyclization coupling to all-carbon quaternary stereocenters. *J. Am. Chem. Soc.* **2022**, *144*, 4776–4782.

^{1}H NMR (400 MHz, CDCl$_3$) δ 7.85 (d, *J* = 7.7 Hz, 1H), 7.66 (t, *J* = 7.5 Hz, 1H), 7.49 – 7.40 (m, 2H), 7.36 – 7.26 (m, 5H), 3.52 – 3.30 (m, 3H), 2.98 (d, *J* = 13.8 Hz, 1H).

^{13}C NMR (101 MHz, CDCl$_3$) δ 197.5, 150.6, 136.4, 134.2, 133.3, 130.0, 128.72, 128.67, 127.9, 126.6, 125.6, 119.6, 48.9, 41.3, 36.7.

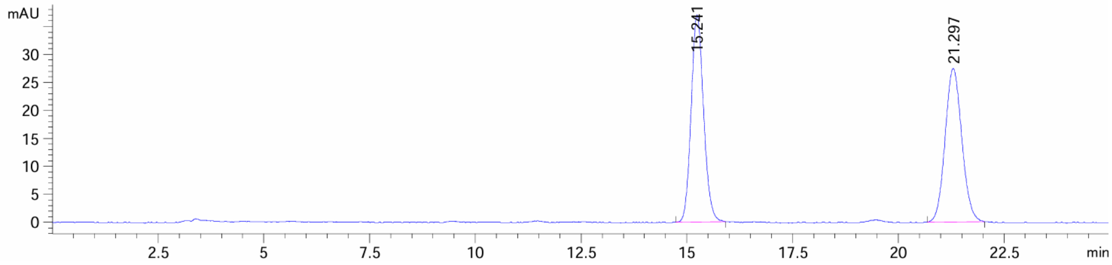

Peak #	RetTime [min]	Type	Width [min]	Area [mAU*s]	Height [mAU]	Area %
1	15.241	BB	0.3149	757.82202	37.01549	50.1034
2	21.297	BB	0.4165	754.69348	27.47118	49.8966

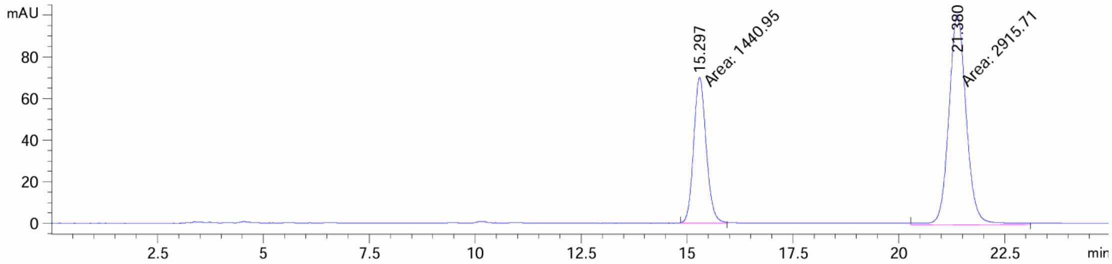

Peak #	RetTime [min]	Type	Width [min]	Area [mAU*s]	Height [mAU]	Area %
1	15.297	MM	0.3433	1440.95471	69.96227	33.0747
2	21.380	MM	0.4819	2915.70776	100.84247	66.9253

Section 7. Synthetic applications

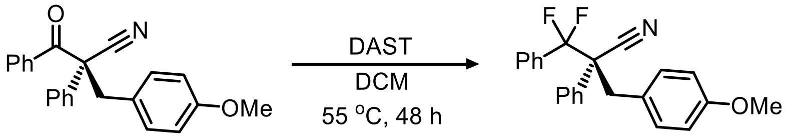

(+)-**30** (+)-**70**

Following the literature procedure[6], to an oven-dried 10 mL Schlenk tube were added (+)-**30** (34.1 mg, 0.1 mmol, 100 mol%), DCM (2 mL), and diethylaminosulfur trifluoride (DAST, 483.6 mg, 3.0 mmol,

[6] Komatsuda, M., Ohki, H., Kondo, H., Jr., Suto, A., Yamaguchi, J. Ring-opening fluorination of isoxazoles. *Org Lett.* **2022**, *24*, 3270–3274.

3000 mol%). The reaction mixture was stirred at 55 ºC for 48 h, then cooled to room temperature, quenched with saturated NH_4Cl aqueous solution, and extracted with DCM (5 mL × 3). The organic phase was combined, washed with brine, dried with Na_2SO_4, filtered, and concentrated. The residue was purified by flash column chromatography to afford (+)-**70** (18.3 mg, 50% Yield). R_f = 0.6 (Hexane/EtOAc = 5:1).

HPLC analysis (Chiralpak AD-H, hexane/^{i}PrOH = 99:1, 1.0 mL/min, 205 nm; t_r (minor) = 15.08 min, t_r (major) = 16.52 min) gave the isomeric composition of the product: 96:4 e.r., $[\alpha]_D^{20}$ = +41.2 (*c* = 1.0, $CHCl_3$).

^{1}H NMR (600 MHz, $CDCl_3$) δ 7.37 – 7.21 (m, 8H), 7.17 (d, *J* = 6 Hz, 2H), 6.95 (d, *J* = 8.7 Hz, 2H), 6.66 (d, *J* = 8.7 Hz, 2H), 3.72 – 3.65 (m, 5H).

^{13}C NMR (151 MHz, $CDCl_3$) δ 158.8, 132.7 (t, *J* = 25.7 Hz), 131.6, 131.0 (d, *J* = 2.9 Hz), 130.4, 128.9, 128.6, 128.4, 127.7, 126.8 (t, *J* = 6.3 Hz), 125.8, 120.7 (t, *J* = 256.9 Hz), 117.9 (d, *J* = 7.5 Hz), 113.5, 59.4 (dd, *J* = 31.5, 26.7 Hz), 55.1, 37.34 (t, *J* = 3.0 Hz).

^{19}F NMR (565 MHz, $CDCl_3$) δ -96.42 (d, *J* = 241.2 Hz), -103.43 (d, *J* = 241.2 Hz).

HRMS (ESI) calcd $C_{23}H_{20}F_2NO^+$ $[M+H]^+$: 364.1507. Found: 364.1506.

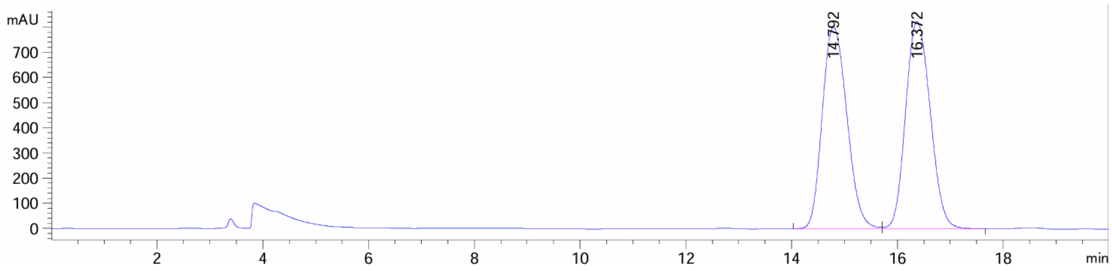

Peak #	RetTime [min]	Type	Width [min]	Area [mAU*s]	Height [mAU]	Area %
1	14.792	BV	0.5170	2.59016e4	801.44971	49.1979
2	16.372	VB	0.5163	2.67462e4	824.90051	50.8021

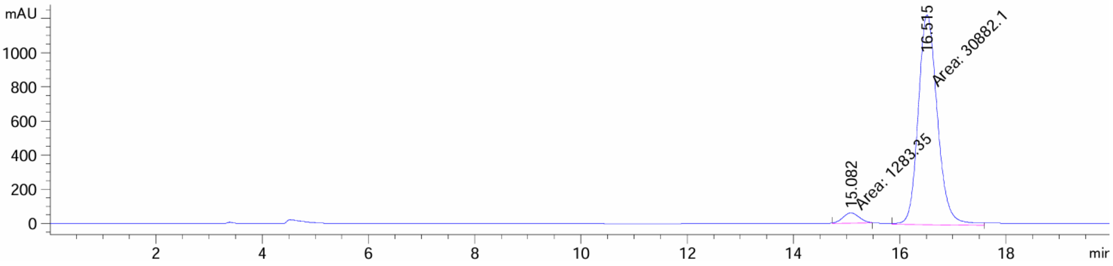

Peak #	RetTime [min]	Type	Width [min]	Area [mAU*s]	Height [mAU]	Area %
1	15.082	MM	0.3561	1283.34924	60.06793	3.9898
2	16.515	MM	0.4191	3.08821e4	1228.03162	96.0102

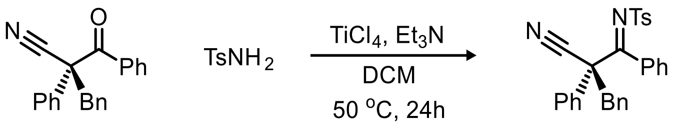

(+)-**2** (+)-**71**

Following the literature procedure[7], to an oven-dried 10 mL Schlenk tube were added (+)-**2** (31.1 mg, 0.1 mmol, 100 mol%) and $TsNH_2$ (20.5 mg, 0.12 mmol, 120 mol%). The tube was sealed with a rubber septum and evacuated/refilled with nitrogen for three times. DCM (0.5 mL) was added and the resulting solution was cooled to 0 ºC, followed by the addition of NEt_3 (21 µL, 0.15 mmol, 150 mol%) and $TiCl_4$ (0.1 mL, 1 M in DCM, 100 mol%). The reaction mixture was stirred under nitrogen at 50 ºC for 24 h, then quenched with saturated NH_4Cl aqueous solution and extracted with DCM (5 mL × 3). The organic phase was combined, washed with brine, dried with Na_2SO_4, filtered, and concentrated. The residue was purified by flash column chromatography to afford (+)-**71** (24.1 mg, 52% Yield). R_f = 0.5 (Hexane/EtOAc = 5:1).

HPLC analysis (Chiralpak IC, hexane/^{i}PrOH = 90:10, 1.0 mL/min, 254 nm; t_r (minor) = 34.45 min, t_r (major) = 43.54 min) gave the isomeric composition of the product: 96:4 e.r., $[\alpha]_D^{20}$ = +34.7 (*c* = 1.0, $CHCl_3$).

^{1}H NMR (400 MHz, $CDCl_3$) δ 7.74 (d, *J* = 8.1 Hz, 2H), 7.42 – 7.31 (m, 6H), 7.28 (d, *J* = 8.0 Hz, 2H), 7.24 – 7.11 (m, 5H), 6.99 (d, *J* = 7.2 Hz, 2H), 6.80 (d, *J* = 7.7 Hz, 2H), 3.75 (d, *J* = 13.8 Hz, 1H), 3.46 (d, *J* = 13.8 Hz, 1H), 2.43 (s, 3H).

^{13}C NMR (101 MHz, $CDCl_3$) δ 178.56, 144.32, 136.85, 134.15, 133.03, 130.56, 130.47, 129.58, 129.24, 129.21, 128.03, 127.59, 127.56, 127.45, 127.20, 126.83, 117.71, 60.54, 44.22, 21.58.

HRMS (ESI) calcd $C_{29}H_{25}N_2O_2S^+$ $[M+H]^+$: 465.1631. Found: 465.1633.

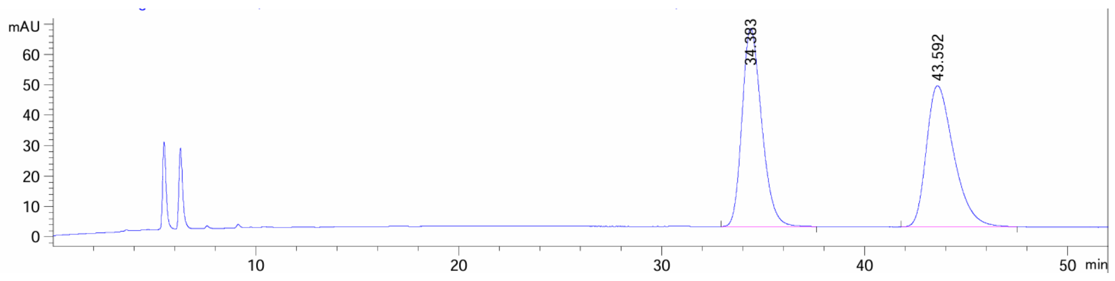

[7] Chen, J., Zhang, Z., Li, B., Li, F., Wang, Y., Zhao, M., Gridnev, I. D., Imamoto, T., Zhang, W. $Pd(OAc)_2$-catalyzed asymmetric hydrogenation of sterically hindered N-tosylimines. *Nat. Commun.* **2018**, *9*, 5000.

Peak #	RetTime [min]	Type	Width [min]	Area [mAU*s]	Height [mAU]	Area %
1	34.383	BB	1.0255	4383.65576	65.10648	50.2388
2	43.592	BB	1.3640	4341.99072	46.38882	49.7612

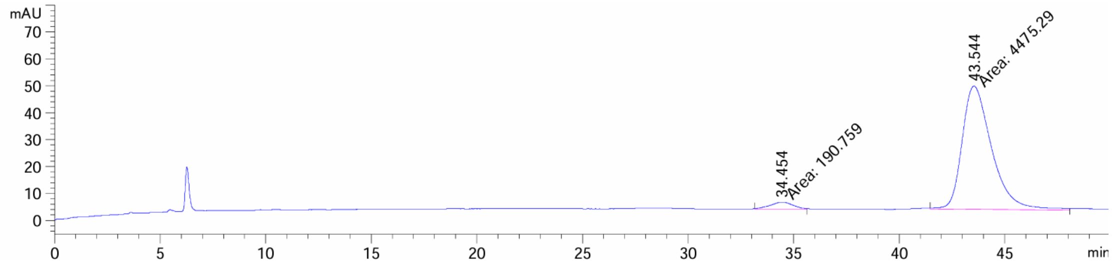

Peak #	RetTime [min]	Type	Width [min]	Area [mAU*s]	Height [mAU]	Area %
1	34.454	MM	1.2343	190.75909	2.57589	4.0882
2	43.544	MM	1.6300	4475.28662	45.75994	95.9118

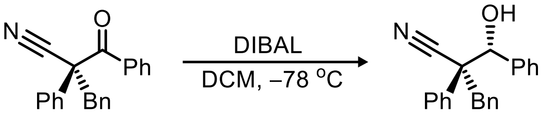

(+)-**2** (+)-**72**

To a 10 mL round bottom flask was added (+)-**2** (31.1 mg, 0.1 mmol, 100 mol%). The flask was sealed with a rubber septum and evacuated/refilled with nitrogen for three times. Dry DCM (2 mL) was added and the resulting solution was stirred at −78 °C for 30 min, followed by the addition of DIBAL (1.5 mL, 1 M in hexane, 150 mol%). The reaction mixture was stirred at −78 °C for 3 h, then quenched with potassium sodium tartrate aqueous solution and extracted with DCM (5 mL × 3). The organic phase was combined, washed with brine, dried with Na_2SO_4, filtered, and concentrated. The residue was purified by flash column chromatography to afford (+)-**72** (24.6 mg, 79% Yield). R_f = 0.3 (Hexane/EtOAc = 5:1).

HPLC analysis (Chiralpak IC-3, hexane/^{i}PrOH = 99:1, 1.0 mL/min, 205 nm; t_r (major) = 8.66 min, t_r (minor) = 11.35 min) gave the isomeric composition of the product: 95.5:4.5 e.r., $[\alpha]_D^{20}$ = +35.3 (*c* = 1.0, $CHCl_3$). Crude NMR analysis indicated a d.r. value of >20:1. The relative stereochemistry of the product was postulated based on a comparison of the DIBAL reduction product of **49** to a literature-reported compound (see below for detailed comparison).

^{1}H NMR (400 MHz, $CDCl_3$) δ 7.43 (d, *J* = 5.8 Hz, 2H), 7.32 (t, *J* = 7.1 Hz, 8H), 7.14 (d, *J* = 5.7 Hz, 3H), 7.01 (d, *J* = 6.5 Hz, 2H), 5.09 (s, 1H), 3.28 – 3.18 (m, 2H), 2.27 (br, 1H).

^{13}C NMR (101 MHz, $CDCl_3$) δ 138.0, 134.68, 134.66, 130.3, 129.0, 128.6, 128.4, 128.1, 128.0, 127.9, 127.8, 127.1, 119.9, 78.4, 56.7, 42.0.

HRMS (ESI) calcd $C_{22}H_{20}NO^+$ $[M+H]^+$: 314.1539. Found: 314.1539.

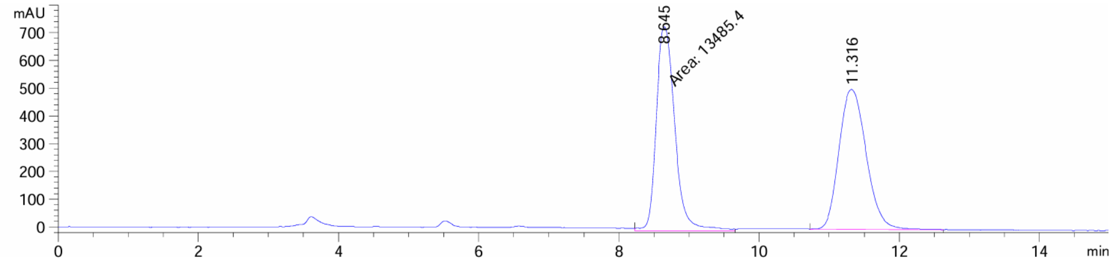

Peak #	RetTime [min]	Type	Width [min]	Area [mAU*s]	Height [mAU]	Area %
1	8.645	MM	0.3058	1.34854e4	735.05957	49.8099
2	11.316	BV R	0.4244	1.35884e4	504.29666	50.1901

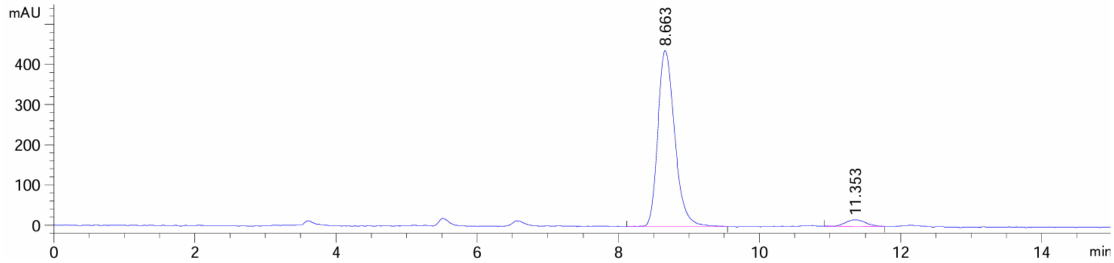

Peak #	RetTime [min]	Type	Width [min]	Area [mAU*s]	Height [mAU]	Area %
1	8.663	VV R	0.2586	7325.85986	439.18222	95.5535
2	11.353	VB R	0.2578	340.90128	17.01440	4.4465

Determination of the diastereoselectivity of the DIBAL reduction

To determine the relative configuration of the DIBAL reduction products (**72** and **79**) and their downstream derivatives, we have chosen desymmetrization product **49** as the model substrate for the reduction and compared the resulting data with literature-reported ones. The assignment is also consistent with the Felkin-Anh model where the nitrile substituent is placed antiperiplanar to the hydride nucleophile to align its σ* orbital with the π/π* orbital for stabilizing the incoming anion. Subsequently, related compounds from DIBAL reduction in this work (i.e., **72**, **75**, **79**-**81**) are assigned accordingly.

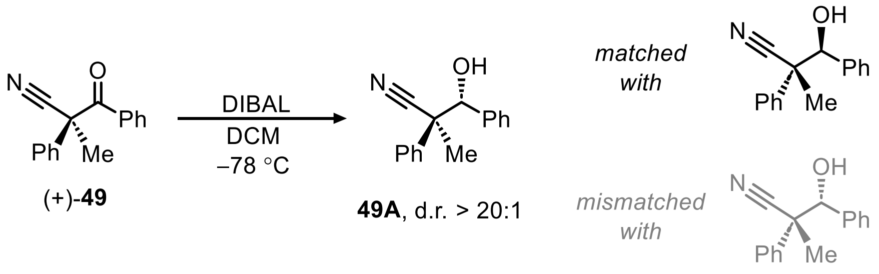

49A'

Chem. Eur. J. **2014**, *20*, 9268
Eur. J. Org. Chem. **2015**, 3495

49B

Felkin-Anh Model

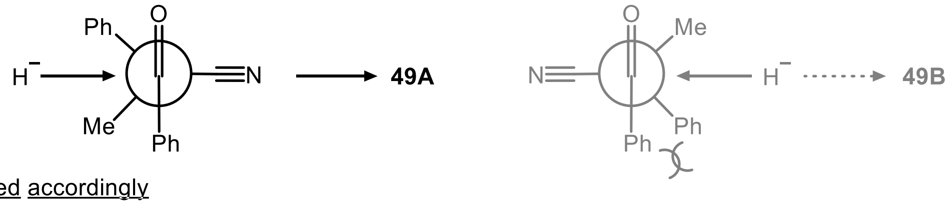

Assigned accordingly

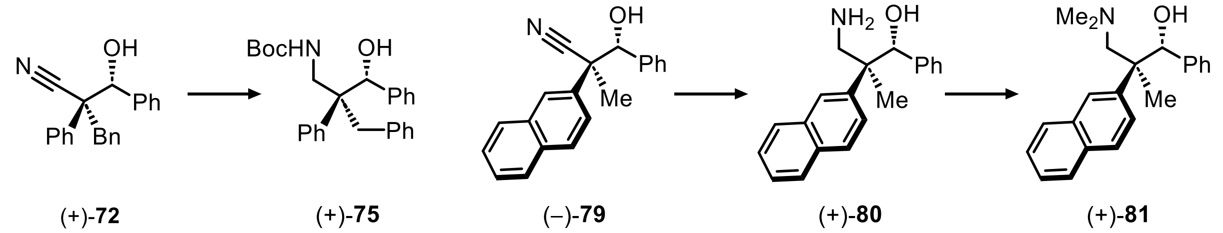

H-NMR of **49A**	H-NMR of **49A'**<br>(*Chem. Eur. J.* **2014**, *20*, 9268)	H-NMR of **49B**<br>(*Eur. J. Org. Chem.* **2015**, 3495)
7.49 (d, *J* = 7.5 Hz, 2H)	7.49 (dt, *J* = 8.3 Hz, 1.7, 2H)	7.31-7.23 (m, 8H)
7.43-7.37 (m, 3H)	7.43-7.37 (m, 3H)	7.17-7.09 (m, 2H)
7.37-7.29 (m, 5H)	7.37-7.30 (m, 5H)	
4.88 (s, 1H)	4.87 (s, 1H)	4.83 (s, 1H)
2.27 (br, 1H)	2.24 (br, 1H)	2.46 (br, 1H)
1.61 (s, 3H)	1.61 (s, 3H)	1.86 (s, 3H)
C-NMR of **49A**	C-NMR of **49A'**<br>(*Chem. Eur. J.* **2014**, *20*, 9268)	C-NMR of **49B**<br>(*Eur. J. Org. Chem.* **2015**, 3495)
137.78	137.77	138.17
137.54	137.44	136.81
128.91	128.73 (2)	128.51
128.88		128.44
128.42	128.27	128.13
128.13	127.97	127.79
127.57	127.50	127.22
126.75	126.68	126.91
121.73	121.75	121.95
79.93	79.64	79.83
49.51	49.37	49.08
22.60	22.45	22.07

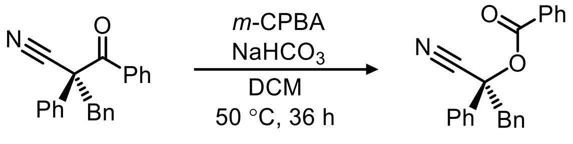

(+)-**2** (+)-**73**

To a solution of (+)-**2** (31.1 mg, 0.1 mmol, 100 mol%) in DCM were added *m*-CPBA (86.3 mg, 0.375 mmol, 375 mol%, 75%) and $NaHCO_3$ (84.0 mg, 1.0 mmol, 1000 mol%). The resulting mixture was stirred at 50 °C for 36 h, then cooled to room temperature. The reaction mixture was quenched with saturated NH_4Cl aqueous solution and extracted with DCM (3 × 2 mL). The organic phase was combined, washed with brine, dried with Na_2SO_4, filtered, and concentrated. The residue was purified by flash column chromatography to afford (+)-**73** (22.6 mg, 69% Yield). R_f = 0.3 (Hexane/EtOAc = 5:1).

HPLC analysis (Chiralpak IC-3, hexane/^{i}PrOH = 95:5, 1.0 mL/min, 254 nm; t_r (major) = 16.39 min, t_r (minor) = 24.25 min) gave the isomeric composition of the product: 94:6 e.r., $[\alpha]_D^{20}$ = +89.5 (*c* = 1.0, $CHCl_3$).

^{1}H NMR (400 MHz, $CDCl_3$) δ 8.05 (d, *J* = 7.0 Hz, 2H), 7.67 – 7.58 (m, 1H), 7.48 (t, *J* = 7.8 Hz, 2H), 7.44 – 7.42 (m, 2H), 7.39 – 7.33 (m, 3H), 7.30 – 7.23 (m, 3H), 7.20 – 7.10 (m, 2H), 3.66 (d, *J* = 13.7 Hz, 1H), 3.50 (d, *J* = 13.7 Hz, 1H).

^{13}C NMR (101 MHz, $CDCl_3$) δ 163.9, 136.6, 133.9, 132.4, 131.0, 129.9, 129.2, 128.9, 128.8, 128.7, 128.2, 127.9, 124.9, 117.1, 77.6, 48.6.

HRMS (ESI) calcd $C_{22}H_{18}NO_2^+$ $[M+H]^+$: 328.1332. Found: 328.1332.

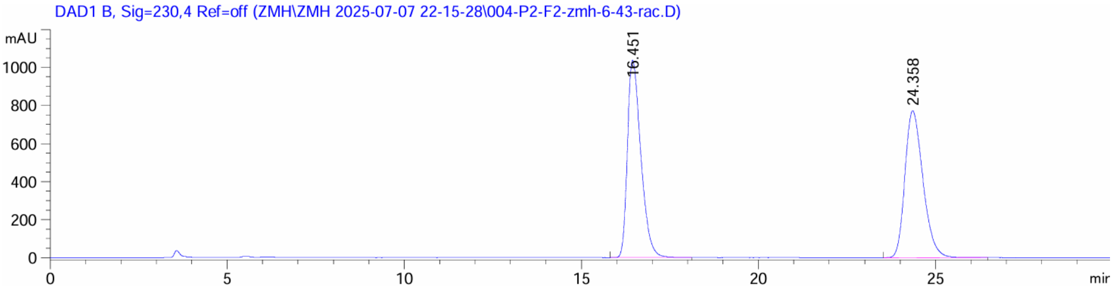

Peak #	RetTime [min]	Type	Width [min]	Area [mAU*s]	Height [mAU]	Area %
1	16.451	BB	0.4074	2.73473e4	1037.97290	49.9756
2	24.358	BB	0.5468	2.73740e4	773.56689	50.0244

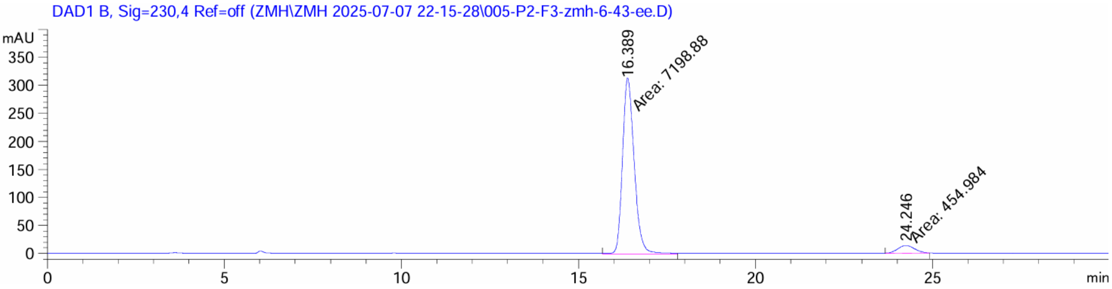

Peak #	RetTime [min]	Type	Width [min]	Area [mAU*s]	Height [mAU]	Area %
1	16.389	MM	0.3817	7198.87695	314.37021	94.0555
2	24.246	MM	0.5532	454.98383	13.70668	5.9445

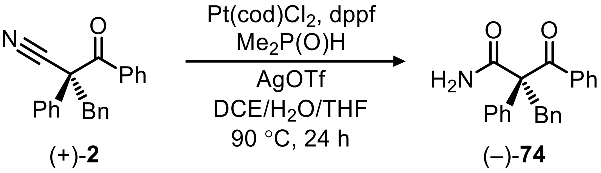

(+)-**2** (–)-**74**

Following the literature procedure[8], to an oven-dried 10 mL Schlenk tube were added Pt(cod)Cl_2 (1.9 mg, 0.005 mmol, 5 mol%) and 1,1′-ferrocenediyl-bis(diphenylphosphine) (dppf, 2.8 mg, 0.005 mmol, 5 mol%). The tube was sealed with a rubber septum and evacuated/refilled with nitrogen for three times. DCE (2 mL) was added and the resulting solution was stirred at room temperature for 10 min, followed by the addition of AgOTf (2.6 mg, 0.01 mmol, 10 mol%), dimethylphosphine oxide (0.5 mg, 0.01 mmol, 5 mol%), (+)-**2** (31.1 mg, 0.1 mmol, 100 mol%), THF (1 mL), and H_2O (0.3 mL). The reaction mixture was stirred under nitrogen at 90 °C for 24 h, then quenched with saturated NH_4Cl aqueous solution and extracted with DCM (5 mL × 3). The organic phase was combined, washed with brine, dried with Na_2SO_4, filtered, and concentrated. The residue was purified by flash column chromatography to afford (–)-**74** (23.6 mg, 72% Yield). R_f = 0.3 (Hexane/EtOAc = 5:1).

HPLC analysis (Chiralpak IC-3, hexane/^{i}PrOH = 70:30, 1.0 mL/min, 254 nm; t_r (minor) = 23.54 min, t_r (major) = 27.75 min) gave the isomeric composition of the product: 95.5:4.5 e.r., $[\alpha]_D^{20}$ = –44.1 (*c* = 1.0, $CHCl_3$).

^{1}H NMR (400 MHz, $CDCl_3$) δ 7.48 – 7.42 (m, 5H), 7.40 – 7.29 (m, 3H), 7.27 – 7.23 (m, 2H), 7.17 – 7.03 (m, 3H), 6.74 (d, *J* = 7.4 Hz, 2H), 4.38 (d, *J* = 13.5 Hz, 1H), 3.57 (d, *J* = 13.5 Hz, 1H).

^{13}C NMR (101 MHz, $CDCl_3$) δ 200.9, 173.3, 139.1, 136.8, 135.8, 132.6, 130.0, 129.9, 129.0, 128.1, 128.0, 127.5, 126.8, 66.5, 41.8.

HRMS (ESI) calcd $C_{22}H_{20}NO_2^+$ $[M+H]^+$: 330.1489. Found: 330.1489.

[8] Xing, X., Xu, C., Chen, B., Li, C., Virgil, S. C., Grubbs, R. H. Highly active platinum catalysts for nitrile and cyanohydrin hydration: catalyst design and ligand screening via high-throughput techniques. *J. Am. Chem. Soc.* **2018**, *140*, 17782–17789.

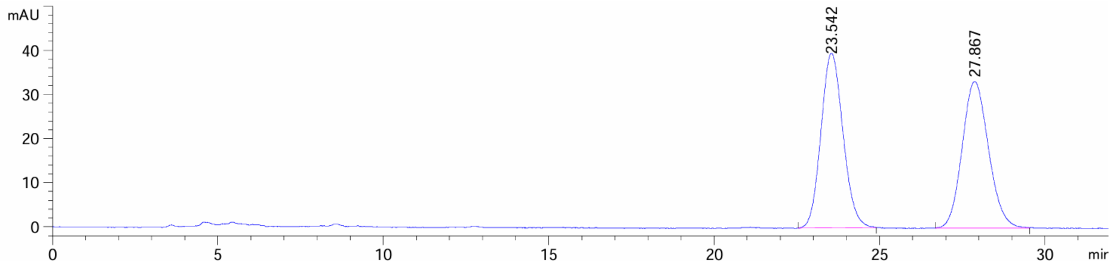

Peak #	RetTime [min]	Type	Width [min]	Area [mAU*s]	Height [mAU]	Area %
1	23.542	BB	0.6825	1835.95898	39.62691	49.9923
2	27.867	BB	0.6901	1836.52515	33.16074	50.0077

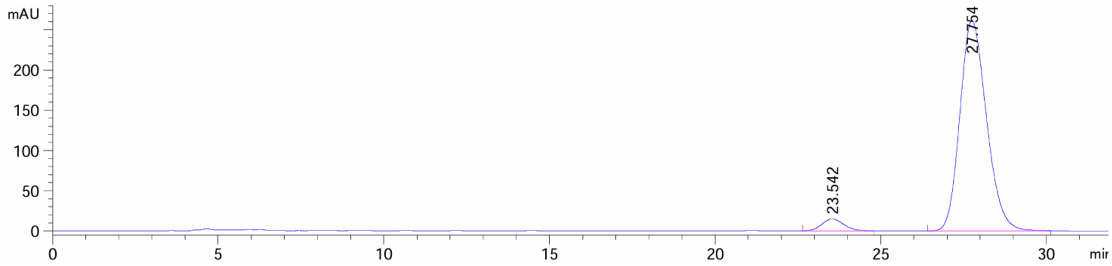

Peak #	RetTime [min]	Type	Width [min]	Area [mAU*s]	Height [mAU]	Area %
1	23.542	BB	0.6064	678.05115	14.74759	4.4374
2	27.754	BB	0.8495	1.46024e4	261.47443	95.5626

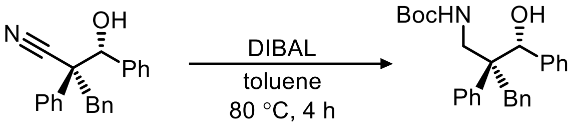

(+)-**72** (+)-**75**

To a 10 mL round bottom flask was added (+)-**72** (31.3 mg, 0.1 mmol, 100 mol%). The flask was sealed with a rubber septum and evacuated/refilled with nitrogen for three times. Dry toluene (1 mL) was added, followed by the addition of DIBAL (0.3 mL, 1 M in hexane, 300 mol%) dropwise at room temperature. The reaction mixture was stirred at 80 °C for 4 h, then quenched with saturated potassium sodium tartrate aqueous solution and extracted with DCM (5 mL × 3). The organic phase was combined, washed with brine, dried with Na_2SO_4, filtered, and concentrated. The residue was dissolved in DCM (1 mL), followed by the addition of NEt_3 (28 μL, 0.2 mmol, 200 mol%) and Boc_2O (87.2 mg, 0.4 mmol, 400 mol%). The reaction mixture was stirred overnight at room temperature. Upon complete consumption of the starting material, the reaction mixture was concentrated under reduced pressure and purified by flash chromatography to afford (+)-**75** (30.3 mg, 73% Yield). R_f = 0.4 (Hexane/EtOAc = 10:1).

HPLC analysis (Chiralpak ID-3, hexane/^{i}PrOH = 85:15, 1.0 mL/min, 254 nm; t_r (major) = 25.99 min, t_r (minor) = 33.82 min, t_r (major) = 60.85 min, t_r (minor) = 68.02 min) gave the isomeric composition

of the product: 95:5 e.r. and >20:1 d.r., $[\alpha]_D^{20}$ = +26.9 (c = 1.0, $CHCl_3$).

^{1}H NMR (600 MHz, $CDCl_3$) δ 7.42 – 7.37 (m, 3H), 7.33 – 7.29 (m, 3H), 7.22 – 7.18 (m, 2H), 7.13 – 7.06 (m, 3H), 7.02 (t, J = 7.6 Hz, 2H), 6.56 (d, J = 7.1 Hz, 2H), 5.66 (s, 1H), 4.19 (d, J = 13.1 Hz, 1H), 3.72 (d, J = 13.2 Hz, 1H), 3.14 (d, J = 14.1 Hz, 1H), 2.76 (d, J = 14.1 Hz, 1H), 1.53 (s, 9H).

^{13}C NMR (151 MHz, $CDCl_3$) δ 151.8, 148.6, 138.2, 135.4, 135.2, 129.9, 128.6, 128.2, 128.0, 127.9, 127.8, 127.5, 126.8, 87.7, 84.0, 48.0, 44.7, 39.4, 27.9.

HRMS (ESI) calcd $C_{27}H_{31}NNaO_3^+$ $[M+Na]^+$: 440.2196. Found: 440.2198.

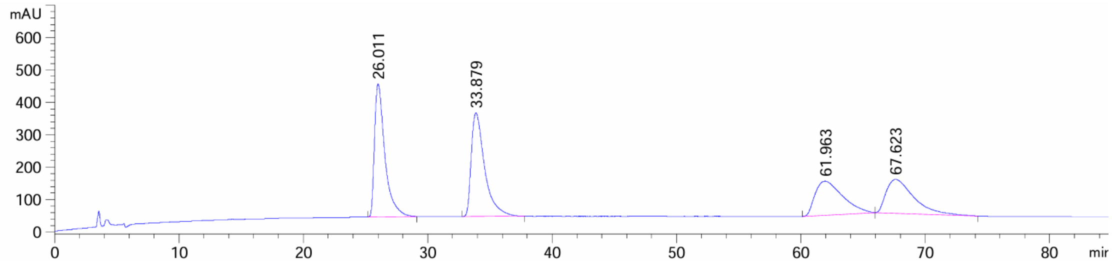

Peak #	RetTime [min]	Type	Width [min]	Area [mAU*s]	Height [mAU]	Area %
1	26.011	BB	0.8547	2.37317e4	410.34534	29.6660
2	33.879	BB	1.0862	2.39124e4	319.12335	29.8918
3	61.963	BB	1.8010	1.61319e4	105.20146	20.1657
4	67.623	BB	1.8236	1.62204e4	104.69087	20.2764

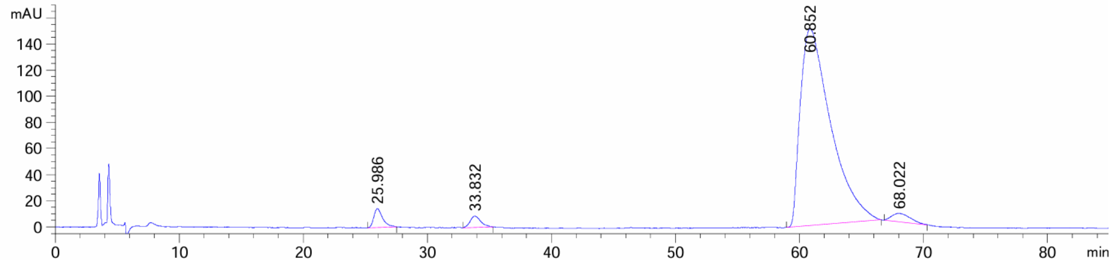

Peak #	RetTime [min]	Type	Width [min]	Area [mAU*s]	Height [mAU]	Area %
1	25.986	BB	0.5949	717.91064	14.46766	2.5948
2	33.832	BB	0.7182	517.98615	8.70531	1.8722
3	60.852	BB	2.0180	2.57655e4	150.60072	93.1254
4	68.022	BB	1.2290	666.13330	6.37557	2.4076

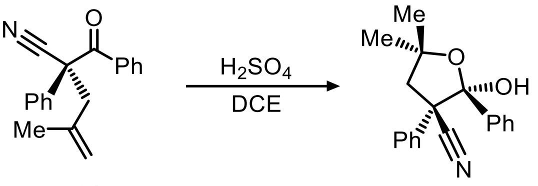

(+)-**61** (–)-**76**

To a solution of (+)-**61** (27.5 mg, 0.1 mmol, 100 mol%) in DCE (1 mL) was added H_2SO_4 (conc.) (20 μL). The reaction mixture was stirred at room temperature for 20 min, then quenched with saturated $NaHCO_3$ aqueous solution and extracted with DCM (5 mL × 3). The organic phase was combined, washed with brine, dried with Na_2SO_4, filtered, and concentrated. The residue was purified by flash column chromatography to afford (–)-**76** (24.2 mg, 83% Yield). R_f = 0.3 (Hexane/EtOAc = 5:1).

HPLC analysis (Chiralpak IG-3, hexane/^{i}PrOH = 90:10, 1.0 mL/min, 254 nm; t_r (major) = 7.57 min, t_r (minor) = 8.32 min) gave the isomeric composition of the product: 95:5 e.r., $[\alpha]_D^{20}$ = –20.7 (c = 1.0, $CHCl_3$).

^{1}H NMR (400 MHz, $CDCl_3$) δ 7.45 – 7.26 (m, 10H), 3.22 (d, J = 12.8 Hz, 1H), 2.70 (d, J = 12.8 Hz, 1H), 2.65 (br, 1H), 1.77 (s, 3H), 1.67 (s, 3H).

^{13}C NMR (101 MHz, $CDCl_3$) δ 138.0, 132.1, 129.3, 128.5, 128.4, 127.9, 127.7, 127.2, 121.9, 106.0, 81.3, 59.1, 47.5, 32.2, 29.1.

HRMS (ESI) calcd $C_{19}H_{20}NO_2^+$ $[M+H]^+$: 294.1489. Found: 294.1487.

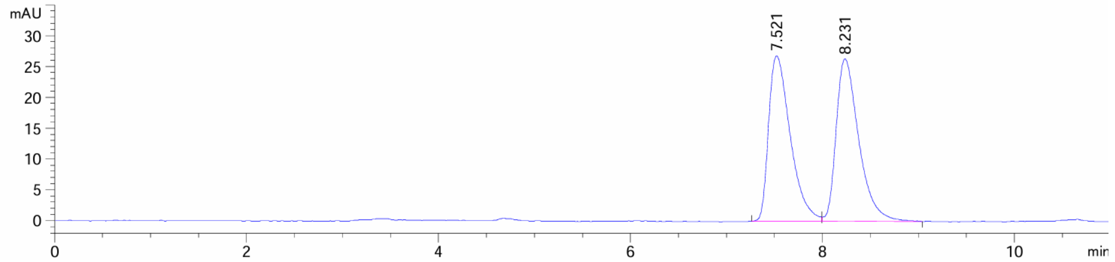

Peak #	RetTime [min]	Type	Width [min]	Area [mAU*s]	Height [mAU]	Area %
1	7.521	BV	0.2431	421.10492	26.82509	49.3674
2	8.231	VB	0.2493	431.89743	26.34283	50.6326

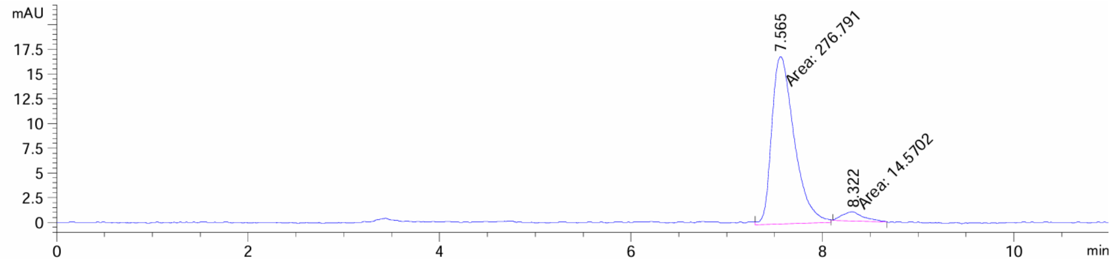

Peak #	RetTime [min]	Type	Width [min]	Area [mAU*s]	Height [mAU]	Area %
1	7.565	MM	0.2725	276.79053	16.92707	94.9993
2	8.322	MM	0.2594	14.57020	9.36114e-1	5.0007

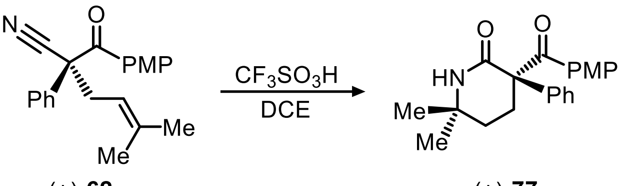

(+)-**62** (+)-**77**

To a solution of (+)-**62** (31.9 mg, 0.1 mmol, 100 mol%) in DCE (1 mL) was added CF_3SO_3H (18 μL, 0.2 mmol, 200 mol%). The reaction mixture was stirred at room temperature for 1 h, then quenched with saturated $NaHCO_3$ aqueous solution and extracted with DCM (5 mL × 3). The organic phase was combined, washed with brine, dried with Na_2SO_4, filtered, and concentrated. The residue was purified by flash column chromatography to afford (+)-**77** (24.3 mg, 72% Yield). R_f = 0.3 (Hexane/EtOAc = 2:1).

HPLC analysis (Chiralpak IB-3, hexane/^{i}PrOH = 95:5, 1.0 mL/min, 254 nm; t_r (minor) = 12..86 min, t_r (major) = 14.13 min) gave the isomeric composition of the product: 92:8 e.r., $[\alpha]_D^{20}$ = +82.0 (c = 1.0, $CHCl_3$).

^{1}H NMR (500 MHz, $CDCl_3$) δ 7.90 – 7.84 (m, 2H), 7.50 – 7.45 (m, 2H), 7.41 (t, J = 7.7 Hz, 2H), 7.38 – 7.30 (m, 1H), 6.84 – 6.78 (m, 2H), 3.80 (s, 3H), 2.52 – 2.49 (m, 1H), 2.20 – 2.14 (m, 1H), 1.75 – 1.69 (m, 1H), 1.45 – 1.39 (m, 1H), 1.31 (br, 1H), 1.22 (s, 3H), 1.21 (s, 3H).

^{13}C NMR (126 MHz, $CDCl_3$) δ 189.3, 163.8, 136.2, 132.5, 129.7, 128.6, 126.6, 125.8, 119.9, 113.7, 70.4, 56.2, 55.5, 38.8, 34.6, 29.3, 29.2.

HRMS (ESI) calcd $C_{21}H_{23}NNaO_3^+$ $[M+Na]^+$: 360.1570. Found: 360.1570.

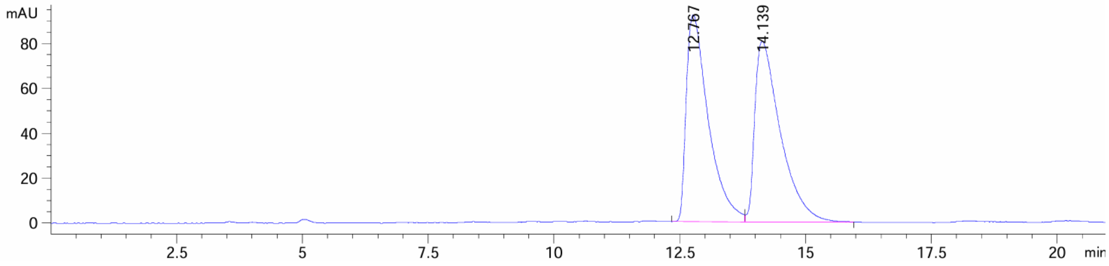

Peak #	RetTime [min]	Type	Width [min]	Area [mAU*s]	Height [mAU]	Area %
1	12.767	BV	0.4595	2821.75317	92.08607	49.7055
2	14.139	VV R	0.5239	2855.18848	80.43111	50.2945

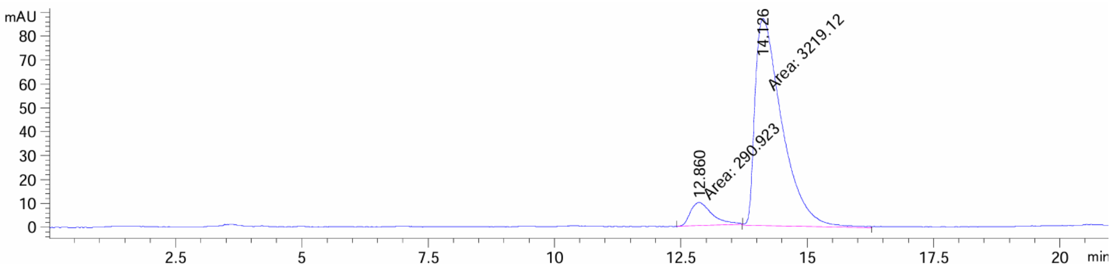

Peak #	RetTime [min]	Type	Width [min]	Area [mAU*s]	Height [mAU]	Area %
1	12.860	MM	0.4998	290.92297	9.70059	8.2883
2	14.126	MM	0.6192	3219.12256	86.64622	91.7117

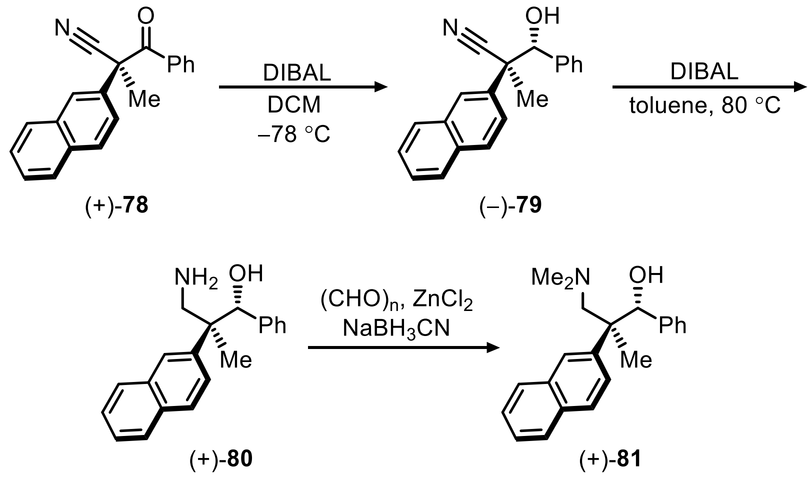

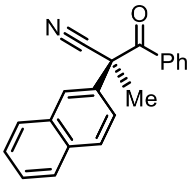

(+)-**78**

(+)-**78** was obtained as a white solid from the desymmetric addition of **S78** using the general procedure with **L8** was used instead of **L1** (41.7 mg, 73% Yield). R_f = 0.5 (Hexane/EtOAc = 10:1).

HPLC analysis (Chiralpak IC-3, hexane/^{i}PrOH = 99:1, 1.0 mL/min, 254 nm; t_r (minor) = 9.27 min, t_r (major) = 10.94 min) gave the isomeric composition of the product: 84:16 e.r., $[\alpha]_D^{20}$ = +122.1 (c = 1.0, $CHCl_3$).

^{1}H NMR (400 MHz, $CDCl_3$) δ 8.06 (d, J = 2.0 Hz, 1H), 7.94 – 7.85 (m, 4H), 7.85 – 7.80 (m, 1H), 7.56 – 7.49 (m, 2H), 7.47 – 7.42 (m, 2H), 7.30 (t, J = 7.8 Hz, 2H), 2.00 (s, 3H).

^{13}C NMR (101 MHz, $CDCl_3$) δ 191.1, 134.7, 133.7, 133.43, 133.41, 132.8, 130.1, 129.9, 128.5, 128.1,

127.7, 127.03, 126.99, 124.8, 122.5, 120.6, 51.7, 27.4.

HRMS (ESI) calcd $C_{20}H_{16}NO^+$ $[M+H]^+$: 286.1226. Found: 286.1226.

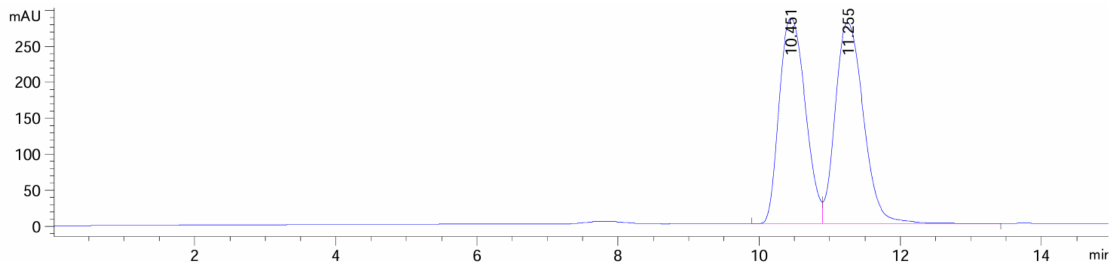

Peak #	RetTime [min]	Type	Width [min]	Area [mAU*s]	Height [mAU]	Area %
1	10.451	BV	0.4179	7346.43262	285.83374	48.6736
2	11.255	VB	0.4412	7746.82910	279.66376	51.3264

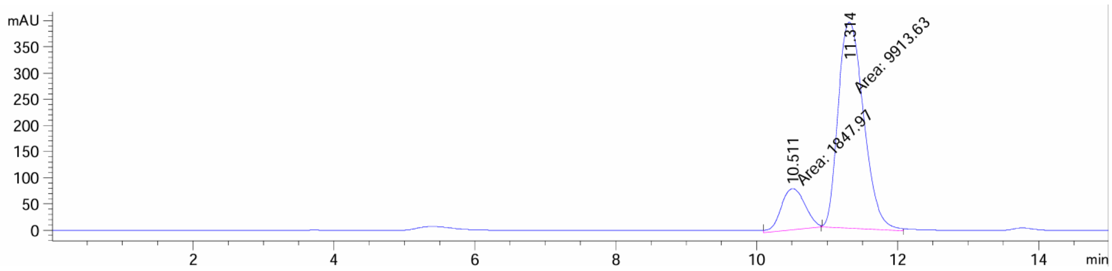

Peak #	RetTime [min]	Type	Width [min]	Area [mAU*s]	Height [mAU]	Area %
1	10.511	MM	0.3932	1847.97253	78.32333	15.7119
2	11.314	MM	0.4200	9913.62891	393.40482	84.2881

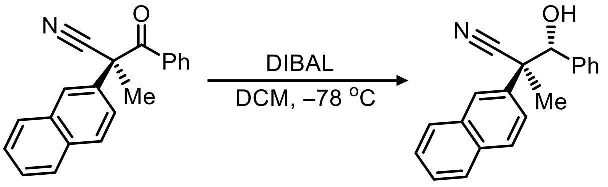

(+)-**78** (–)-**79**

To a 10 mL round bottom flask was added (+)-**78** (28.5 mg, 0.1 mmol, 100 mol%). The flask was sealed with a rubber septum and evacuated/refilled with nitrogen for three times. Dry DCM (2 mL) was added and the resulting solution was stirred at −78 °C for 30 min, followed by the addition of DIBAL (1.5 mL, 1 M in hexane, 150 mol%). The reaction mixture was stirred at −78 °C for 3 h, then quenched with potassium sodium tartrate aqueous solution and extracted with DCM (3 × 2 mL). The organic phase was combined, washed with brine, dried with Na_2SO_4, filtered, and concentrated. The residue was purified by flash column chromatography to afford (–)-**79** (19.6 mg, 68% Yield). R_f = 0.3 (Hexane/EtOAc = 5:1).

HPLC analysis (Chiralpak IC-3, hexane/^{i}PrOH = 90:10, 1.0 mL/min, 254 nm; t_r (minor) = 13.04 min, t_r (major) = 14.41 min) gave the isomeric composition of the product: 84:16 e.r., $[\alpha]_D^{20}$ = –34.6 (c = 1.0, $CHCl_3$). Crude NMR analysis indicated a d.r. value of >20:1. The relative stereochemistry of the product was postulated based on a comparison of the DIBAL reduction product of **49** to a literature-reported compound.

^{1}H NMR (400 MHz, $CDCl_3$) δ 8.03 (d, J = 2.0 Hz, 1H), 7.96 – 7.84 (m, 3H), 7.59 – 7.56 (m, 3H), 7.44 – 7.30 (m, 5H), 4.99 (d, J = 2.6 Hz, 1H), 2.47 (d, J = 3.1 Hz, 1H), 1.71 (s, 3H).

^{13}C NMR (101 MHz, $CDCl_3$) δ 137.8, 134.8, 133.0, 132.8, 128.9, 128.8, 128.2, 128.1, 127.6, 127.5, 126.7, 126.5, 123.6, 121.7, 79.6, 49.7, 22.8.

HRMS (ESI) calcd $C_{20}H_{18}NO^+$ $[M+H]^+$: 288.1383. Found: 288.1383.

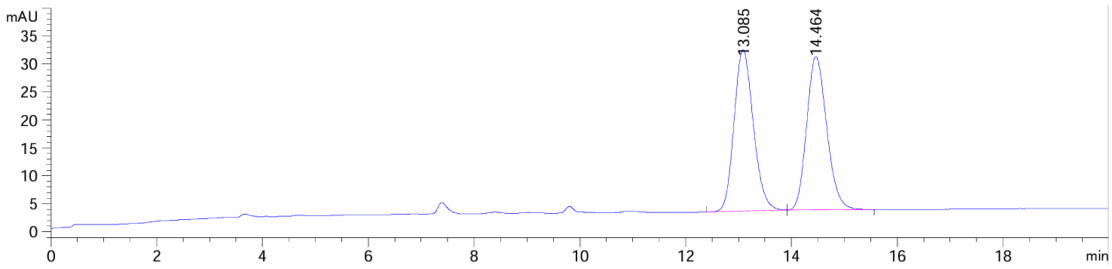

Peak #	RetTime [min]	Type	Width [min]	Area [mAU*s]	Height [mAU]	Area %
1	13.085	BB	0.3921	732.49078	28.86159	50.2349
2	14.464	BB	0.4106	725.63910	27.43406	49.7651

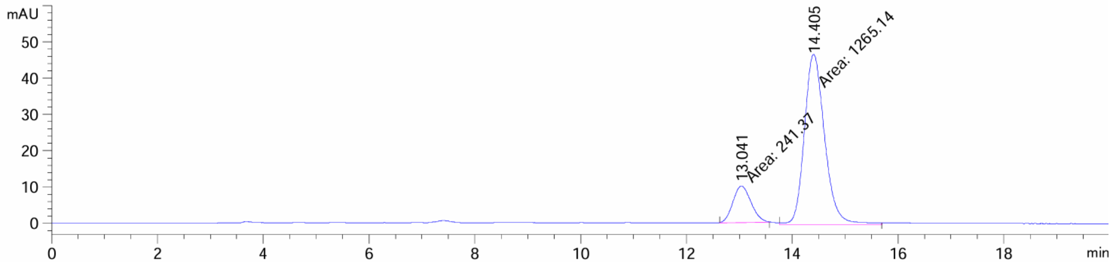

Peak #	RetTime [min]	Type	Width [min]	Area [mAU*s]	Height [mAU]	Area %
1	13.041	MM	0.3984	241.36951	10.09821	16.0218
2	14.405	MM	0.4487	1265.13623	46.99352	83.9782

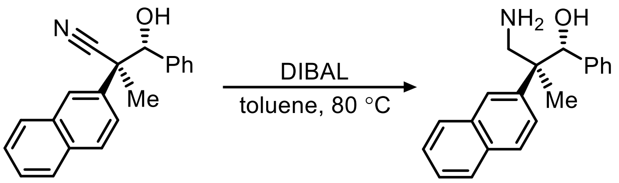

(–)-**79** (+)-**80**

To a 10 mL round bottom flask was added (–)-**79** (28.7 mg, 0.1 mmol, 100 mol%). The flask was sealed with a rubber septum and evacuated/refilled with nitrogen for three times. Dry toluene (1 mL) was added, followed by the addition of DIBAL (0.3 mL, 1 M in hexane, 300 mol%) dropwise at room temperature. The reaction mixture was stirred at 80 °C for 4 h, then quenched with saturated potassium sodium tartrate aqueous solution and extracted with DCM (5 mL × 3). The organic phase was combined, washed with brine, dried with Na_2SO_4, filtered, and concentrated. The residue was purified by flash chromatography to afford (+)-**80** (21.1 mg, 73% Yield). R_f = 0.3 (Hexane/EtOAc = 10:1).

HPLC analysis of *N*-Boc-protected derivative (Chiralpak IB-3, hexane/^{i}PrOH = 90:10, 1.0 mL/min, 254 nm; t_r (minor) = 11.85 min, t_r (major) = 13.11 min, t_r (major) = 15.12 min, t_r (minor) = 38.54 min) gave the isomeric composition of the product: 83:17 e.r., > 20:1 d.r., $[\alpha]_D^{20}$ = +31.7 (*c* = 1.0, MeOH).

^{1}H NMR (500 MHz, DMSO-*d*$_6$) δ 7.90 – 7.80 (m, 3H), 7.68 – 7.57 (m, 2H), 7.47 (td, *J* = 6.9, 6.1, 3.8 Hz, 2H), 7.06 – 6.99 (m, 3H), 6.88 – 6.81 (m, 2H), 5.13 (s, 1H), 3.42 – 3.26 (m, 2H), 1.33 (s, 3H).

^{13}C NMR (126 MHz, DMSO-*d*$_6$) δ 141.9, 140.0, 132.8, 131.7, 128.0, 127.4, 127.2, 126.8, 126.6, 126.4, 125.84, 125.77, 125.7, 78.9, 48.4, 46.4, 16.1.

HRMS (ESI) calcd $C_{20}H_{22}NO^+$ $[M+H]^+$: 292.1696. Found: 292.1696.

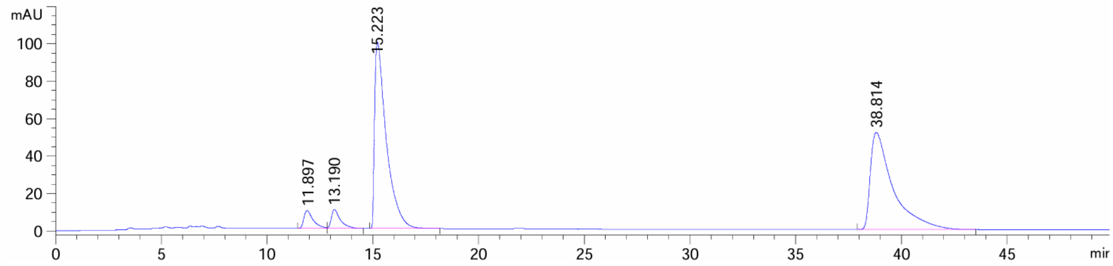

Peak #	RetTime [min]	Type	Width [min]	Area [mAU*s]	Height [mAU]	Area %
1	11.897	BV	0.4521	290.60052	9.51955	3.4931
2	13.190	VB	0.4326	299.48145	10.08057	3.5998
3	15.223	BB	0.5614	3853.27979	99.59992	46.3172
4	38.814	BB	1.0544	3875.95776	51.83122	46.5898

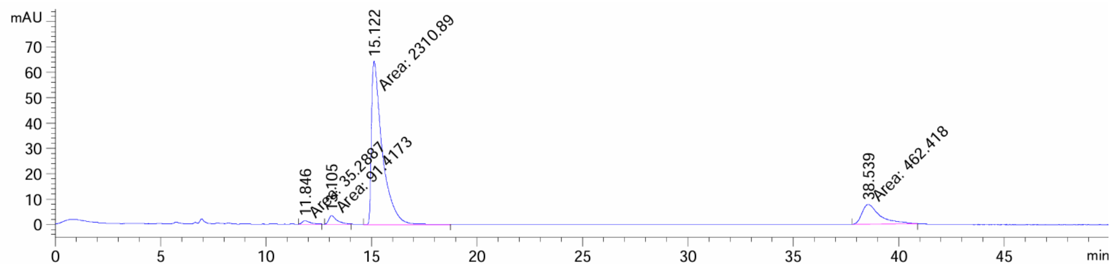

Peak #	RetTime [min]	Type	Width [min]	Area [mAU*s]	Height [mAU]	Area %
1	11.846	MM	0.4340	35.28865	1.35507	1.2168
2	13.105	MM	0.4456	91.41727	3.41897	3.1523
3	15.122	MM	0.5976	2310.89160	64.45236	79.6855
4	38.539	MM	0.9955	462.41760	7.74181	15.9454

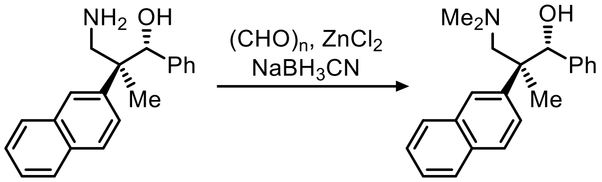

(+)-**80** (+)-**81**

To a solution of (+)-**80** (29.1 mg, 0.1 mmol, 100 mol%) in MeOH (1 mL) were added paraformaldehyde (9.0 mg, 0.3 mmol, 300 mol%), $ZnCl_2$ (40.9 mg, 0.3 mmol, 300 mol%), and $NaBH_3CN$ (18.8 mg, 0.3 mmol, 300 mol%). The reaction mixture was stirred at room temperature for 4 h, then quenched with 1M NaOH and extracted with EtOAc (5 mL × 3). The organic phase was combined, washed with brine, dried with Na_2SO_4, filtered, and concentrated. The residue was purified by flash column chromatography to afford (+)-**81** (16.7 mg, 52% Yield). R_f = 0.6 (DCM/MeOH = 10:1). The crude NMR of reaction indicated a d.r. value of >20:1. The enantiopurity was not determined due to the difficulty of isomer separation. $[\alpha]_D^{20}$ = +31.7 (*c* = 1.0, MeOH).

^{1}H NMR (600 MHz, $CDCl_3$) δ 7.86 – 7.73 (m, 4H), 7.55 (dd, *J* = 8.6, 1.9 Hz, 1H), 7.45 (dd, *J* = 6.2, 3.2 Hz, 2H), 7.15 – 6.97 (m, 5H), 5.51 (s, 1H), 3.32 (d, *J* = 13.8 Hz, 1H), 2.73 (d, *J* = 13.7 Hz, 1H), 2.38 (s, 6H), 1.37 (s, 3H).

^{13}C NMR (101 MHz, $CDCl_3$) δ 141.9, 141.6, 133.3, 132.0, 128.2, 127.7, 127.4, 127.3, 127.1, 126.7, 126.1, 125.9, 125.8, 125.4, 81.4, 70.3, 47.9, 45.9, 29.7, 19.7.

HRMS (ESI) calcd $C_{22}H_{26}NO^+$ $[M+H]^+$: 320.2009. Found: 320.2009.

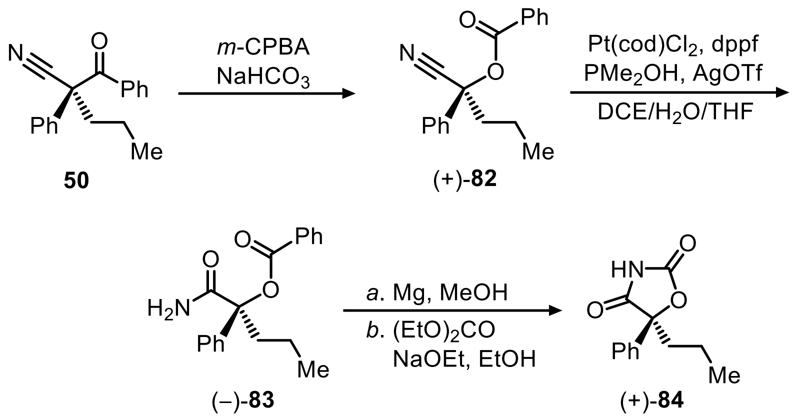

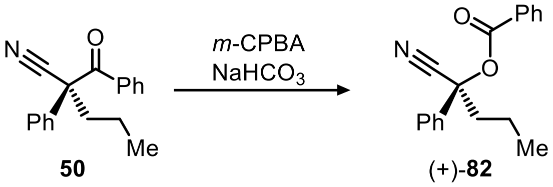

To a solution of (+)-**50** (52.6 mg, 0.2 mmol, 100 mol%) in DCM were added *m*-CPBA (75%, 172.6 mg, 0.75 mmol, 375 mol%) and $NaHCO_3$ (42.0 mg, 0.5 mmol, 500 mol%). The resulting mixture was stirred at 50 °C for 36 h, then cooled to room temperature. The reaction mixture was quenched with saturated NH_4Cl aqueous solution and extracted with EtOAc (5 mL × 3). The organic phase was combined, washed with brine, dried with Na_2SO_4, filtered, and concentrated. The residue was purified by flash column chromatography to afford (+)-**82** (44.5 mg, 80% Yield). R_f = 0.5 (Hexane/EtOAc = 10:1).

HPLC analysis (Chiralpak ID-3, hexane/^{i}PrOH = 99:1, 1.0 mL/min, 254 nm; t_r (minor) = 14.66 min, t_r (major) = 15.91 min) gave the isomeric composition of the product: 92.5:7.5 e.r., $[\alpha]_D^{20}$ = +125.1 (*c* = 1.2, $CHCl_3$).

^{1}H NMR (600 MHz, $CDCl_3$) δ 8.06 – 8.04 (m, 2H), 7.64 – 7.58 (m, 1H), 7.58 – 7.52 (m, 2H), 7.47 (t, *J* = 7.8 Hz, 2H), 7.43 – 7.33 (m, 3H), 2.41 – 2.36 (m, 1H), 2.21 – 2.16 (m, 1H), 1.78 – 1.65 (m, 1H), 1.51 – 1.45 (m, 1H), 1.00 (t, *J* = 7.4 Hz, 3H).

^{13}C NMR (151 MHz, $CDCl_3$) δ 163.9, 137.4, 133.8, 129.8, 129.0, 128.90, 128.87, 128.6, 124.6, 117.5, 77.5, 44.7, 17.6, 13.6.

HRMS (ESI) calcd $C_{18}H_{18}NO_2^+$ $[M+H]^+$: 280.1332. Found: 280.1332.

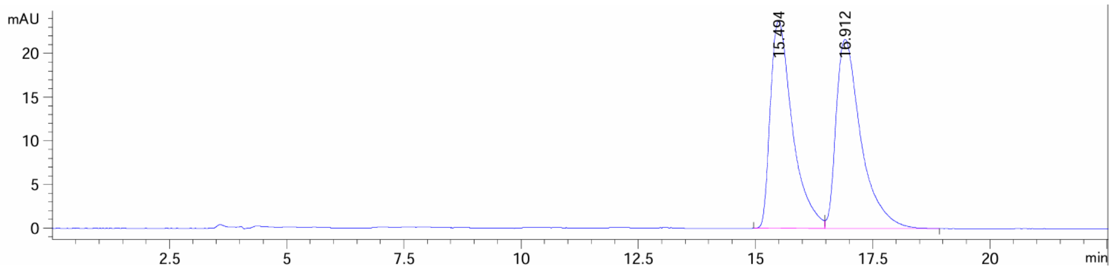

Peak #	RetTime [min]	Type	Width [min]	Area [mAU*s]	Height [mAU]	Area %
1	15.494	BV	0.4973	780.17981	23.74420	49.3550
2	16.912	VB	0.5554	800.57288	21.65243	50.6450

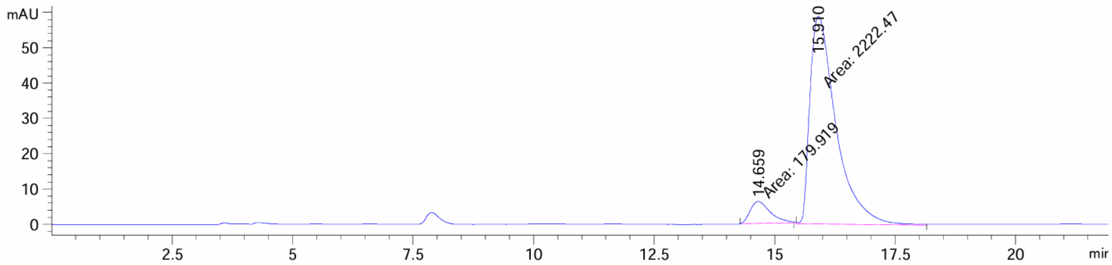

Peak #	RetTime [min]	Type	Width [min]	Area [mAU*s]	Height [mAU]	Area %
1	14.659	MM	0.4840	179.91853	6.19507	7.4891
2	15.910	MM	0.6292	2222.47168	58.86773	92.5109

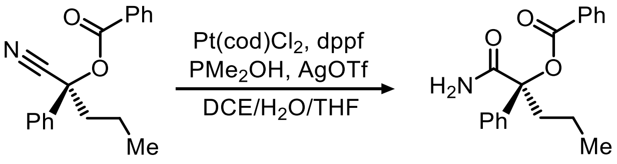

(+)-**82** (–)-**83**

To an oven-dried 10 mL Schlenk tube were added Pt(cod)Cl_2 (5.6 mg, 0.015 mmol, 5 mol%), and dppf (8.3 mg, 0.015 mmol, 5 mol%). The tube was sealed with a rubber septum and evacuated/refilled with nitrogen for three times. DCE (2 mL) was added and the resulting solution was stirred at room temperature for 10 min, followed by the addition of AgOTf (7.7 mg, 0.03 mmol, 10 mol%), dimethylphosphine oxide (1.5 mg, 0.03 mmol, 5 mol%), (+)-**82** (83.7 mg, 0.3 mmol, 100 mol%), THF (1 mL), and H_2O (0.3 mL). The reaction mixture was stirred under nitrogen at 90 °C for 24 h, then quenched with saturated NH_4Cl aqueous solution and extracted with DCM (5 mL × 3). The organic phase was combined, washed with brine, dried with Na_2SO_4, filtered, and concentrated. The residue was purified by flash column chromatography to afford (–)-**83** (65.7 mg, 74% Yield). R_f = 0.3 (Hexane/EtOAc = 5:1).

HPLC analysis (Chiralpak ID-3, hexane/^{i}PrOH = 90:10, 1.0 mL/min, 254 nm; t_r (minor) = 27.90 min, t_r (major) = 31.25 min) gave the isomeric composition of the product: 92:8 e.r., $[\alpha]_D^{20}$ = –40.9 (*c* = 0.8, $CHCl_3$).

^{1}H NMR (400 MHz, CDCl$_3$) δ 8.18 – 8.01 (m, 2H), 7.68 – 7.59 (m, 1H), 7.57 – 7.55 (m, 2H), 7.50 (t, *J* = 7.7 Hz, 2H), 7.42 – 7.27 (m, 3H), 6.25 (br, 2H), 2.86 – 2.78 (m, 1H), 2.60 – 2.52 (m, 1H), 1.39 – 1.05 (m, 2H), 0.87 (t, *J* = 7.3 Hz, 3H).

^{13}C NMR (101 MHz, CDCl$_3$) δ 173.7, 164.0, 139.5, 133.5, 130.0, 129.6, 128.7, 128.5, 128.0, 124.8, 86.9, 36.1, 16.4, 13.9.

HRMS (ESI) calcd $C_{18}H_{20}NO_3^+$ $[M+H]^+$: 298.1438. Found: 298.1437.

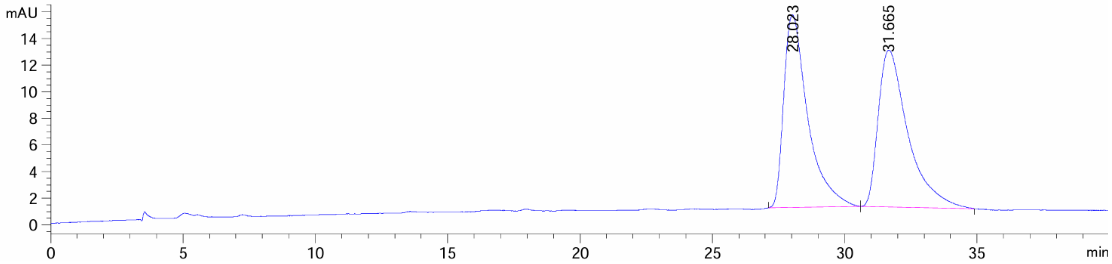

Peak #	RetTime [min]	Type	Width [min]	Area [mAU*s]	Height [mAU]	Area %
1	28.023	BB	0.8973	916.02979	14.49460	49.7810
2	31.665	BB	1.0582	924.08789	11.81857	50.2190

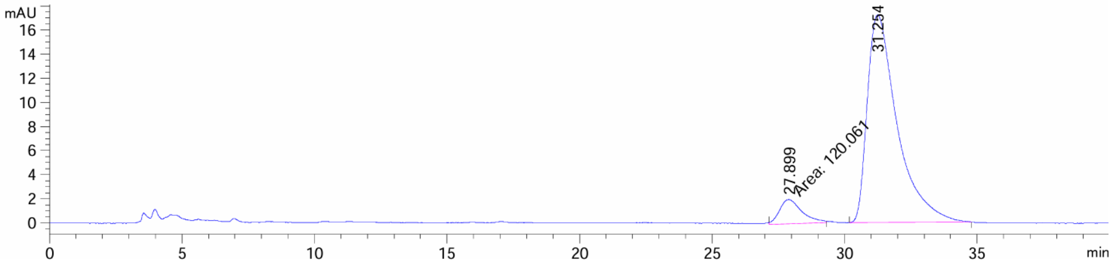

Peak #	RetTime [min]	Type	Width [min]	Area [mAU*s]	Height [mAU]	Area %
1	27.899	MM	0.9883	120.06104	2.02478	8.1399
2	31.254	BB	1.1076	1354.90271	17.22703	91.8601

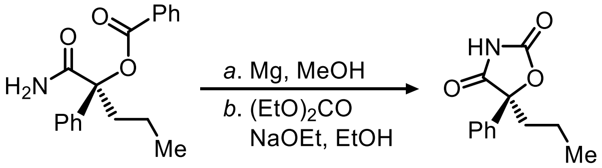

(–)-**83** (+)-**84**

To a solution of (–)-**83** (29.7 mg, 0.1 mmol, 100 mol%) in MeOH was added magnesium powder (12.0 mg, 0.5 mmol, 500 mol%). The resulting mixture was stirred at 50 ºC for overnight, then quenched with saturated NH_4Cl aqueous solution and extracted with EtOAc (5 mL × 3). The organic phase was

combined, washed with brine, dried with Na_2SO_4, filtered, and concentrated to afford the crude deprotection product.

To an oven-dried 10 mL Schlenk tube was added sodium (150 mg), then the tube was sealed with a rubber septum and evacuated/refilled with nitrogen for three times. EtOH (25 mL) was added at 0 °C, and the resulting mixture was stirred at room temperature until the sodium was completely dissolved in ethanol. The solution of crude product above in 1 mL ethanol was added dropwise to the mixture, followed by the addition of diethyl carbonate (60.5 μL, 0.5 mmol, 500 mol%). The resulting mixture was stirred at 100 °C overnight, and then concentrated under reduced pressure. The residue was dissolved in water, and the pH was adjusted to 7 using 1 N HCl. The aqueous phase was extracted with EtOAc (5 mL × 3). The organic phase was combined, washed with brine, dried with Na_2SO_4, filtered, and concentrated. The residue was purified by flash column chromatography to afford (+)-**84** (13.3 mg, 61% Yield). R_f = 0.5 (Hexane/EtOAc = 1:1).

HPLC analysis (Chiralpak IC, hexane/^{i}PrOH = 95:5, 1.0 mL/min, 254 nm; t_r (minor) = 8.60 min, t_r (major) = 9.63 min) gave the isomeric composition of the product: 92:8 e.r., $[\alpha]_D^{20}$ = +60.8 (*c* = 0.8, $CHCl_3$).

^{1}H NMR (400 MHz, $CDCl_3$) δ 8.22 (br, 1H), 7.57 (d, *J* = 7.8 Hz, 2H), 7.46 – 7.33 (m, 3H), 2.18 – 2.14 (m, 2H), 1.43 – 1.35 (m, 2H), 0.94 (t, *J* = 7.4 Hz, 3H).

^{13}C NMR (101 MHz, $CDCl_3$) δ 174.5, 153.9, 135.5, 128.9, 128.8, 124.5, 90.3, 40.7, 16.6, 13.6.

HRMS (ESI) calcd $C_{12}H_{14}NO_3^+$ $[M+H]^+$: 220.0968. Found: 220.0971.

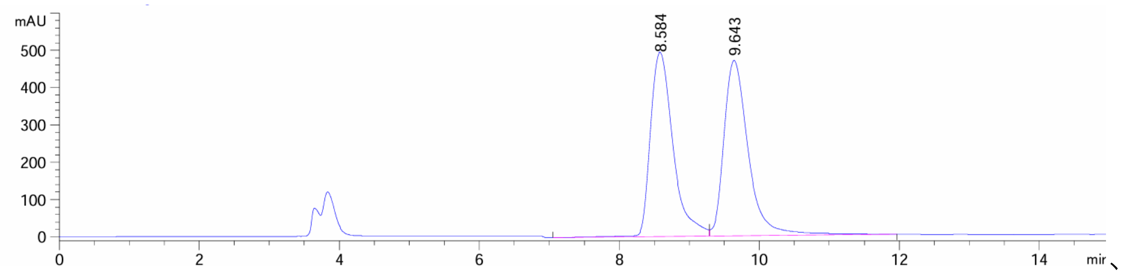

Peak #	RetTime [min]	Type	Width [min]	Area [mAU*s]	Height [mAU]	Area %
1	8.584	BV	0.3499	1.11054e4	494.62872	49.2422
2	9.643	VB	0.3739	1.14472e4	470.44464	50.7578

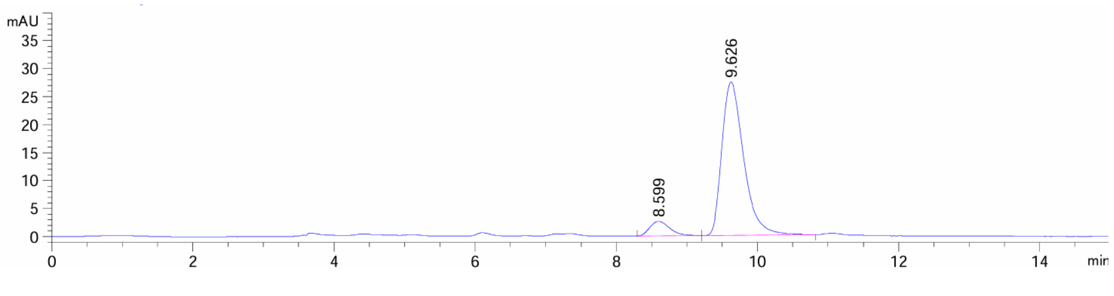

Peak #	RetTime [min]	Type	Width [min]	Area [mAU*s]	Height [mAU]	Area %
1	8.599	BB	0.3049	50.62399	2.58027	7.9102
2	9.626	BB	0.3330	589.35706	27.39876	92.0898

Section 8. Mechanistic studies

8.1 Control experiments

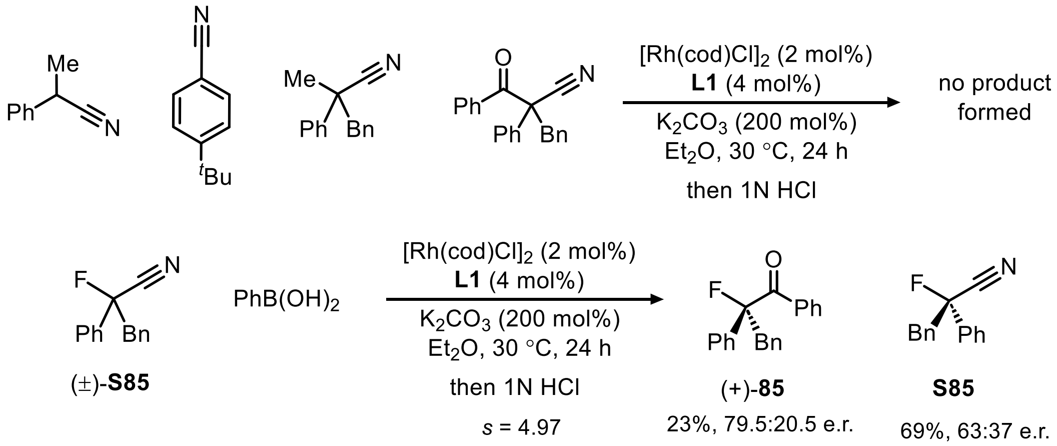

Supplementary Figure S6. Control experiments of mono-nitriles.

Under standard desymmetrization conditions, the four nitrile reactants above failed to give any addition products. The negative results of these control experiments demonstrated the high chemoselectivity of the rhodium catalyst for malononitriles. Nevertheless, when one of the nitrile groups in the malononitrile is replaced with a fluoro substituent, the catalyst was able to reduce the nitrile with a moderate stereoselectivity in a kinetic resolution fashion.

Procedure for the resolution

To an oven-dried 4 mL vial equipped with a stir bar were added **L1** (5.9 mg, 0.008 mmol, 4 mol%) and $[Rh(COD)Cl]_2$ (2.0 mg, 0.004 mmol, 2 mol%). The vial was sealed with a rubber septum and evacuated/refilled with nitrogen for three times. Dry Et_2O (2 mL) was added to the vial via a syringe, then the resulting catalyst solution was stirred at room temperature for 15 min.

To a separate oven-dried 15 mL Schlenk tube equipped with a stir bar was added fluorocyano compounds (0.2 mmol, 100 mol%), K_2CO_3 (55.2 mg, 0.4 mmol, 200 mol%) and phenylboronic acid (0.4 mmol, 200 mol%). The tube was sealed with a rubber septum and evacuated/refilled with nitrogen for three times. Dry Et_2O (2 mL) was added to the reaction tube, followed by the addition of above prepared catalyst solution. The reaction mixture was stirred at 30 °C for 24 h, and followed by the addition of aqueous HCl (1 N, 1 mL) and THF (1 mL). The resulting mixture was stirred at room temperature for 15 minutes, then extracted with EtOAc (10 mL × 3). The organic phase was combined, washed with brine, dried with Na_2SO_4, filtered, and concentrated. The residue was purified by flash column chromatography to afford the product (–)-**82** (14.2 mg, 23% Yield). R_f = 0.7 (Hexane/EtOAc

= 10:1). The starting material was recovered in 69% yield (30.9 mg).

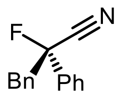

S85

HPLC analysis (Chiralpak ID-3, hexane/^{i}PrOH = 99/1, 1.0 mL/min, 254 nm; t_r (minor) = 5.83 min, t_r (major) = 6.48 min) gave the isomeric composition of the product: 63:37 e.r.

^{1}H NMR (400 MHz, $CDCl_3$) δ 7.44 – 7.40 (m, 5H), 7.30 – 7.27 (m, 3H), 7.14 – 7.12 (m, 2H), 3.56 – 3.32 (m, 2H).

^{13}C NMR (101 MHz, $CDCl_3$) δ 135.6 (d, J = 22.7 Hz), 131.9 (d, J = 3.6 Hz), 130.6, 129.9 (d, J = 1.7 Hz), 128.7, 128.4, 128.0, 124.8 (d, J = 6.1 Hz), 116.9 (d, J = 33.4 Hz), 91.9 (d, J = 186.6 Hz), 48.1 (d, J = 25.8 Hz).

^{19}F NMR (376 MHz, $CDCl_3$) δ -146.43 (dd, J = 22.8, 16.2 Hz).

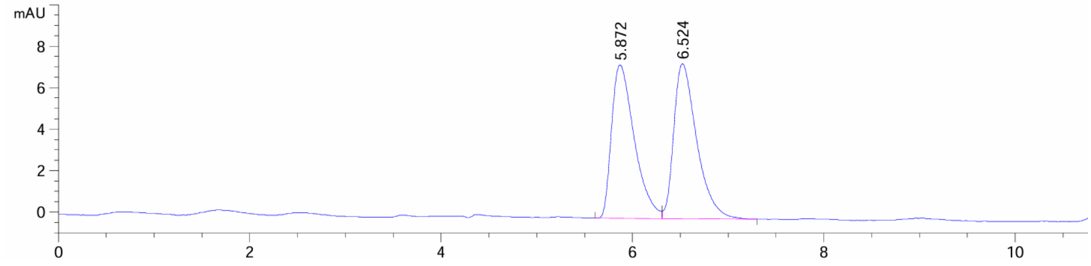

Peak #	RetTime [min]	Type	Width [min]	Area [mAU*s]	Height [mAU]	Area %
1	5.872	BV	0.2548	122.24825	7.39884	49.3857
2	6.524	VB	0.2514	125.28927	7.47919	50.6143

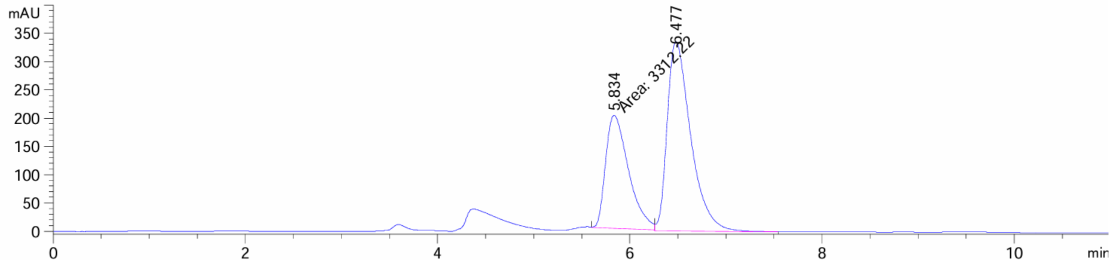

Peak #	RetTime [min]	Type	Width [min]	Area [mAU*s]	Height [mAU]	Area %
1	5.834	MM	0.2759	3312.22192	200.06174	36.6935
2	6.477	VB	0.2606	5714.50146	335.62277	63.3065

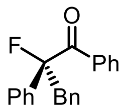

(+)-**85**

HPLC analysis (Chiralpak AD-H, hexane/^{i}PrOH = 99:1, 1.0 mL/min, 254 nm; t_r (major) = 11.60 min, t_r (minor) = 15.59 min) gave the isomeric composition of the product: 79.5:20.5 e.r., $[\alpha]_D^{20}$ = +45.2 (*c* = 1.0, $CHCl_3$).

^{1}H NMR (400 MHz, $CDCl_3$) δ 7.79 (d, *J* = 8.3 Hz, 2H), 7.50 – 7.41 (m, 3H), 7.41 – 7.30 (m, 5H), 7.24 – 7.17 (m, 3H), 7.13 – 7.06 (m, 2H), 3.76 (dd, *J* = 24.7, 14.4 Hz, 1H), 3.48 (dd, *J* = 26.0, 14.4 Hz, 1H).

^{13}C NMR (151 MHz, $CDCl_3$) δ 198.1 (d, *J* = 28.2 Hz), 138.3 (d, *J* = 22.2 Hz), 135.0 (d, *J* = 3.5 Hz), 134.6, 132.9, 130.9, 129.9 (d, *J* = 6.0 Hz), 128.6 (d, *J* = 1.7 Hz), 128.3, 128.1, 127.8, 126.7, 124.2 (d, *J* = 8.8 Hz), 102.8 (d, *J* = 191.8 Hz), 45.5 (d, *J* = 22.4 Hz).

^{19}F NMR (376 MHz, $CDCl_3$) δ -160.85 (t, *J* = 26.3 Hz).

HRMS (ESI) calcd $C_{21}H_{17}FNaO^{+}$ $[M+Na]^{+}$: 327.1156. Found: 327.1155.

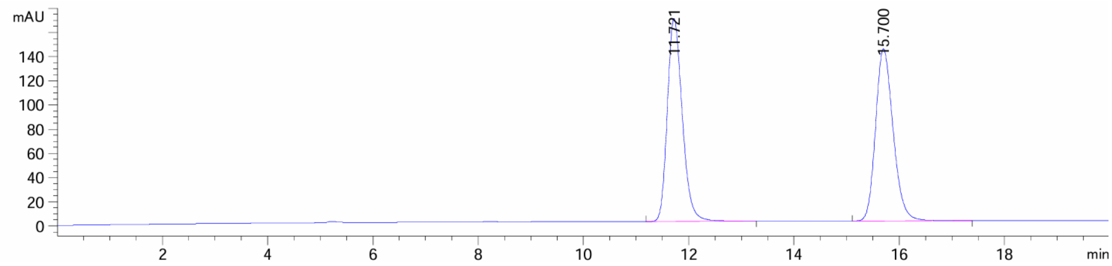

Peak #	RetTime [min]	Type	Width [min]	Area [mAU*s]	Height [mAU]	Area %
1	11.721	BB	0.3042	3310.91040	167.76904	50.0244
2	15.700	BB	0.3568	3307.67651	142.49205	49.9756

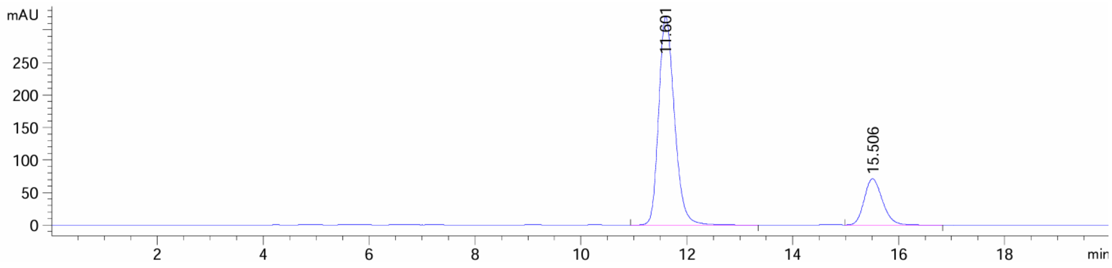

Peak #	RetTime [min]	Type	Width [min]	Area [mAU*s]	Height [mAU]	Area %
1	11.601	BB	0.3191	6621.88623	320.41818	79.5835
2	15.506	BB	0.3712	1698.78687	70.98917	20.4165

8.2 probing matrix for π-π interaction

We conducted a crossed-pair matrix of desymmetrization reactions using malononitriles and boronic acids bearing aryl substituents with varied electronic properties (Supplementary Figure S7). This design allowed us to test potential donor-acceptor π-π complementarity by combining electron-donating (EDG) and electron-withdrawing (EWG) groups on both coupling partners. Across all combinations (EDG/EDG, EWG/EWG, EDG/EWG, and EWG/EDG), the reactions consistently delivered high enantioselectivity, with no discernible influence from the electronic nature of either the boronic acid or the malononitrile aryl motif. These results suggest that π-π interactions are not a dominant factor in enantiocontrol for this transformation.

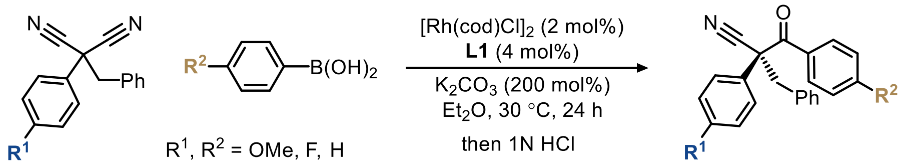

	R^1 = F	R^1 = H	R^1 = OMe
R^2 = F	(+)-**86**, 71%<br>93:7 e.r.	(+)-**4**, 75%<br>92:8 e.r.	(+)-**87**, 69%<br>93:7 e.r.
R^2 = H	(+)-**19**, 81%<br>93:7 e.r.	(+)-**2**, 90%<br>95.5:4.5 e.r.	(+)-**18**, 88%<br>93:7 e.r.
R^2 = OMe	(+)-**88**, 60%<br>92:8 e.r.	(+)-**3**, 62%<br>95:5 e.r.	(+)-**89**, 52%<br>92:8 e.r.

Supplementary Figure S7. Crossed-pair matrix for probing π-π interaction.

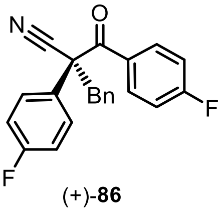

(+)-**86**

(+)-**86** was obtained as colorless oil from the desymmetric addition of **S19** using the general procedure (49.1 mg, 71% Yield). R_f = 0.5 (Hexane/EtOAc = 10:1).

HPLC analysis (Chiralpak IC-3, hexane/^{i}PrOH = 99:1, 1.0 mL/min, 254 nm; t_r (minor) = 7.15 min, t_r (major) = 8.32 min) gave the isomeric composition of the product: 93:7 e.r., $[\alpha]_D^{20}$ = +87.31 (*c* = 1.0, $CHCl_3$).

^{1}H NMR (400 MHz, $CDCl_3$) δ 7.94 – 7.76 (m, 2H), 7.24 – 7.15 (m, 5H), 7.03 (q, *J* = 8.7 Hz, 4H), 6.89 (d, *J* = 7.5 Hz, 2H), 3.61 (d, *J* = 13.7 Hz, 1H), 3.39 (d, *J* = 13.7 Hz, 1H).

^{13}C NMR (101 MHz, $CDCl_3$) δ 189.3, 165.8 (d, *J* = 258.6 Hz), 162.7 (d, *J* = 251.5 Hz), 133.6, 132.9

(d, J = 9.1 Hz), 130.7, 130.5 (d, J = 4.0 Hz), 129.9 (d, J = 3.0 Hz), 128.3 (d, J = 8.1 Hz), 128.1, 127.5, 118.9, 116.6 (d, J = 22.2 Hz), 115.8 (d, J = 22.2 Hz), 57.4, 44.7.

^{19}F NMR (376 MHz, $CDCl_3$) δ -102.64, -112.02.

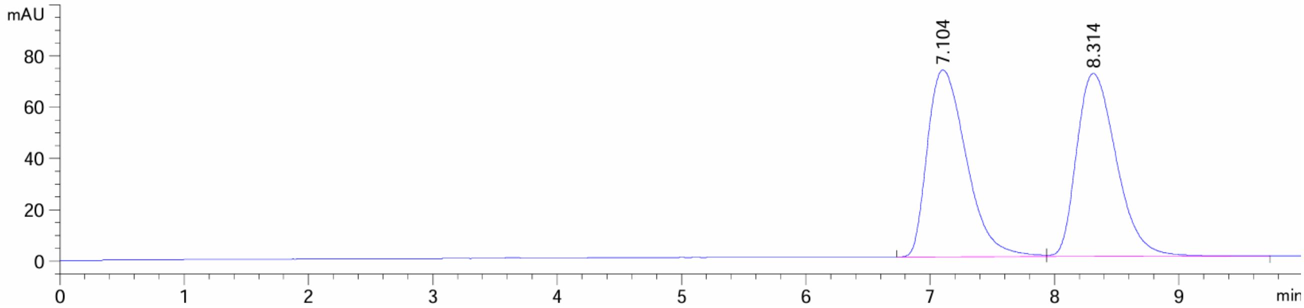

Peak #	RetTime [min]	Type	Width [min]	Area [mAU*s]	Height [mAU]	Area %
1	7.104	BV	0.3447	1590.71814	72.87683	50.0766
2	8.314	VB	0.3490	1585.85120	71.43989	49.9234

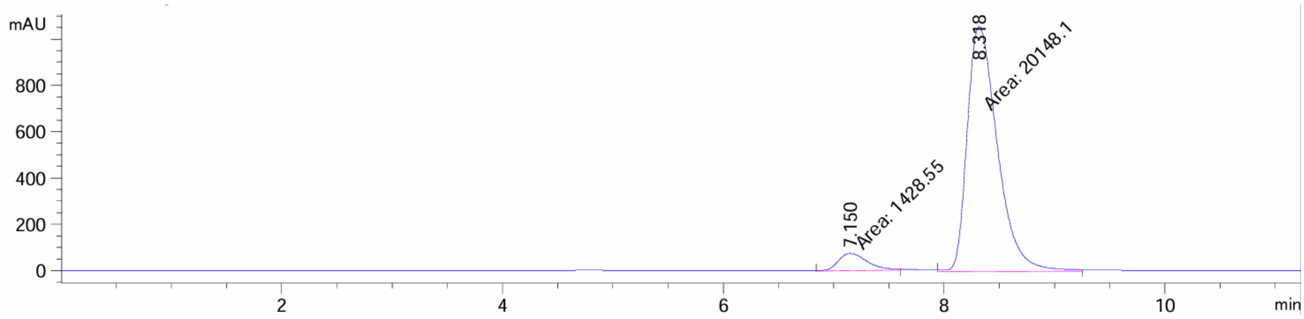

Peak #	RetTime [min]	Type	Width [min]	Area [mAU*s]	Height [mAU]	Area %
1	7.150	MM	0.3115	1428.54712	76.43556	6.6208
2	8.318	MM	0.3169	2.01481e4	1059.73999	93.3792

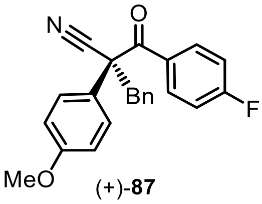

(+)-**87**

(+)-**87** was obtained as colorless oil from the desymmetric addition of **S18** using the general procedure (49.2 mg, 69% Yield). R_f = 0.6 (Hexane/EtOAc = 5:1).

HPLC analysis (Chiralpak IC-3, hexane/^{i}PrOH = 95:5, 1.0 mL/min, 254 nm; t_r (minor) = 6.79 min, t_r (major) = 7.57 min) gave the isomeric composition of the product: 93:7 e.r., $[\alpha]_D^{20}$ = +90.93 (c = 1.5, $CHCl_3$).

^{1}H NMR (400 MHz, CDCl$_3$) δ 7.92 – 7.85 (m, 2H), 7.24 – 7.10 (m, 5H), 7.00 (t, *J* = 8.6 Hz, 2H), 6.93 – 6.89 (m, 2H), 6.89 – 6.84 (m, 2H), 3.79 (s, 3H), 3.60 (d, *J* = 13.7 Hz, 1H), 3.39 (d, *J* = 13.7 Hz, 1H).

^{13}C NMR (101 MHz, CDCl$_3$) δ 189.7, 165.6 (d, *J* = 258.6 Hz), 159.8, 134.1, 132.9 (d, *J* = 10.1 Hz), 130.7, 130.2 (d, *J* = 3.0 Hz), 127.9, 127.6, 127.3, 126.4, 119.2, 115.6 (d, *J* = 22.2 Hz), 114.8, 57.5, 55.3, 44.6.

^{19}F NMR (376 MHz, CDCl$_3$) δ -103.24.

HRMS (ESI) calcd $C_{23}H_{19}FNO_2^+$ [M+H]$^+$: 360.1394. Found: 360.1396.

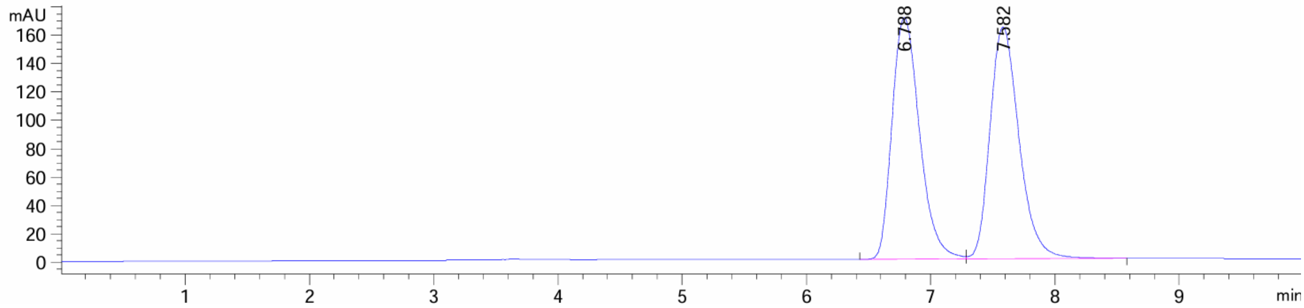

Peak #	RetTime [min]	Type	Width [min]	Area [mAU*s]	Height [mAU]	Area %
1	6.788	BV	0.2415	2623.33521	170.47424	49.6188
2	7.582	VB	0.2541	2663.64819	163.48544	50.3812

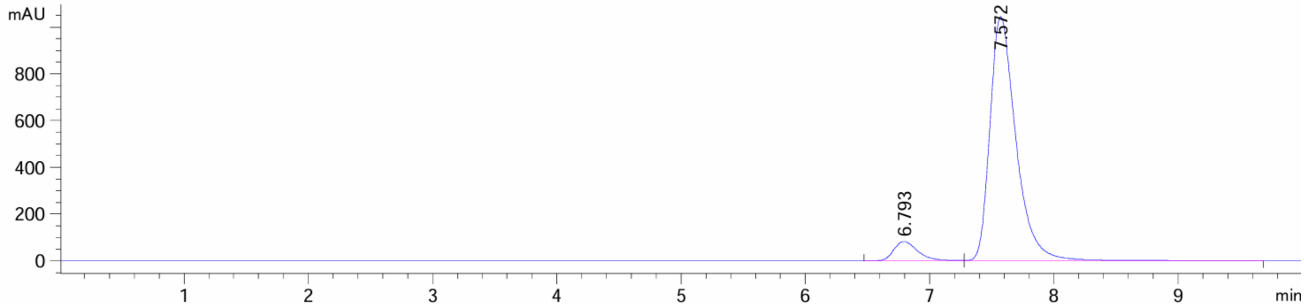

Peak #	RetTime [min]	Type	Width [min]	Area [mAU*s]	Height [mAU]	Area %
1	6.793	BV	0.2071	1114.83020	82.81463	6.8302
2	7.572	VB	0.2199	1.52073e4	1044.27563	93.1698

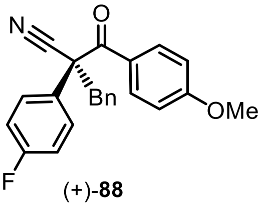

(+)-**88**

(+)-**88** was obtained as colorless oil from the desymmetric addition of **S19** using the general procedure (43.3 mg, 60% Yield). R_f = 0.6 (Hexane/EtOAc = 5:1).

HPLC analysis (Chiralpak IC-3, hexane/^{i}PrOH = 95:5, 1.0 mL/min, 230 nm; t_r (minor) = 8.72 min, t_r (major) = 9.70 min) gave the isomeric composition of the product: 92:8 e.r., $[\alpha]_D^{20}$ = +91.36 (*c* = 1.7, $CHCl_3$).

^{1}H NMR (400 MHz, $CDCl_3$) δ 7.85 (d, *J* = 8.9 Hz, 2H), 7.25 – 7.11 (m, 5H), 7.02 (t, *J* = 8.5 Hz, 2H), 6.88 (d, *J* = 6.8 Hz, 2H), 6.81 (d, *J* = 9.0 Hz, 2H), 3.80 (s, 3H), 3.60 (d, *J* = 13.7 Hz, 1H), 3.38 (d, *J* = 13.7 Hz, 1H).

^{13}C NMR (101 MHz, $CDCl_3$) δ 189.0, 163.9, 162.5 (d, *J* = 250.5 Hz), 133.9, 132.7, 131.2 (d, *J* = 3.0 Hz), 130.7, 128.3, 128.1 (d, *J* = 8.1 Hz), 127.4, 126.2, 119.3, 116.4 (d, *J* = 22.2 Hz), 113.8, 57.0, 55.5, 44.8.

^{19}F NMR (376 MHz, $CDCl_3$) δ -112.61.

HRMS (ESI) calcd $C_{23}H_{19}FNO_2^+$ $[M+H]^+$: 360.1394. Found: 360.1390.

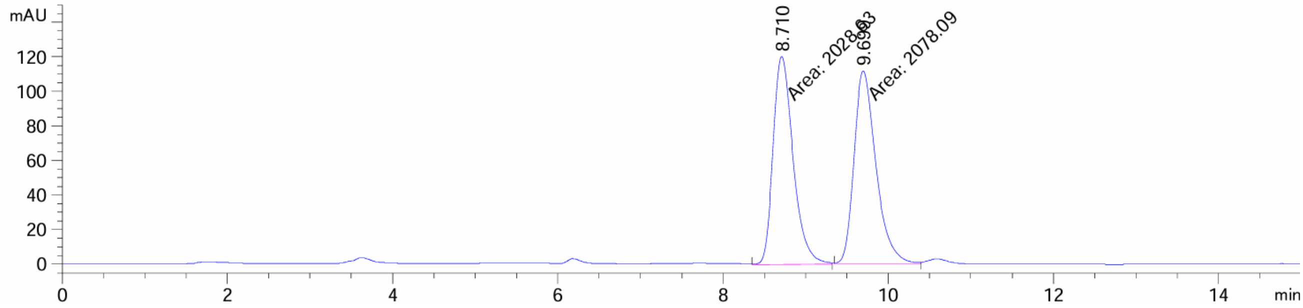

Peak #	RetTime [min]	Type	Width [min]	Area [mAU*s]	Height [mAU]	Area %
1	8.710	MM	0.2809	2028.63464	120.36350	49.3978
2	9.699	MF	0.3100	2078.09448	111.73056	50.6022

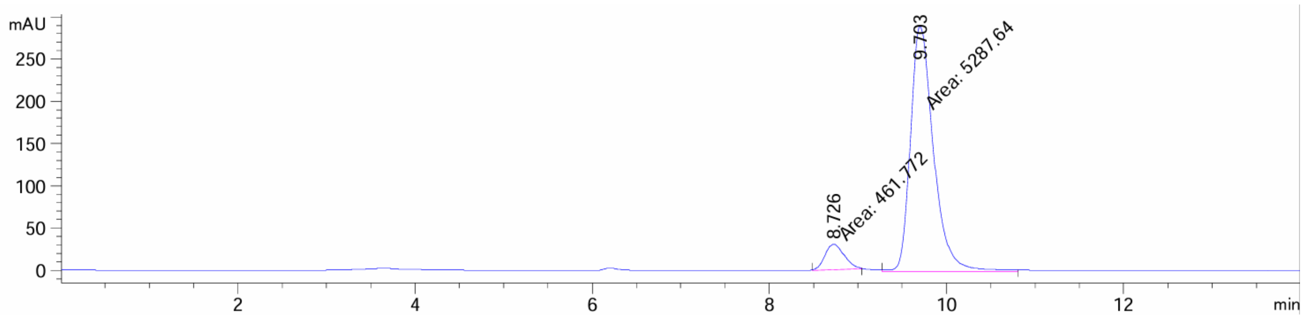

Peak #	RetTime [min]	Type	Width [min]	Area [mAU*s]	Height [mAU]	Area %
1	8.726	MM	0.2544	461.77176	30.25782	8.0316
2	9.703	MM	0.3031	5287.64453	290.75769	91.9684

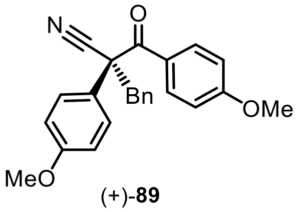

(+)-**89**

(+)-**89** was obtained as colorless oil from the desymmetric addition of **S18** using the general procedure (38.4 mg, 52% Yield). R_f = 0.3 (Hexane/EtOAc = 5:1).

HPLC analysis (Chiralpak IC-3, hexane/^{i}PrOH = 95:5, 1.0 mL/min, 254 nm; t_r (minor) = 10.08 min, t_r (major) = 11.03 min) gave the isomeric composition of the product: 92:8 e.r., $[\alpha]_D^{20}$ = +105.01 (*c* = 1.5, $CHCl_3$).

^{1}H NMR (400 MHz, $CDCl_3$) δ 7.92 – 7.80 (m, 2H), 7.24 – 7.09 (m, 5H), 6.95 – 6.87 (m, 2H), 6.88 – 6.76 (m, 4H), 3.80 (s, 3H), 3.79 (s, 3H), 3.59 (d, *J* = 13.7 Hz, 1H), 3.38 (d, *J* = 13.7 Hz, 1H).

^{13}C NMR (101 MHz, $CDCl_3$) δ 189.5, 163.7, 159.6, 134.4, 132.7, 130.8, 127.9, 127.6, 127.2, 126.5, 119.6, 114.7, 113.6, 57.1, 55.4, 55.3, 44.7.

HRMS (ESI) calcd $C_{24}H_{22}NO_3^+$ $[M+H]^+$: 372.1594. Found: 372.1589.

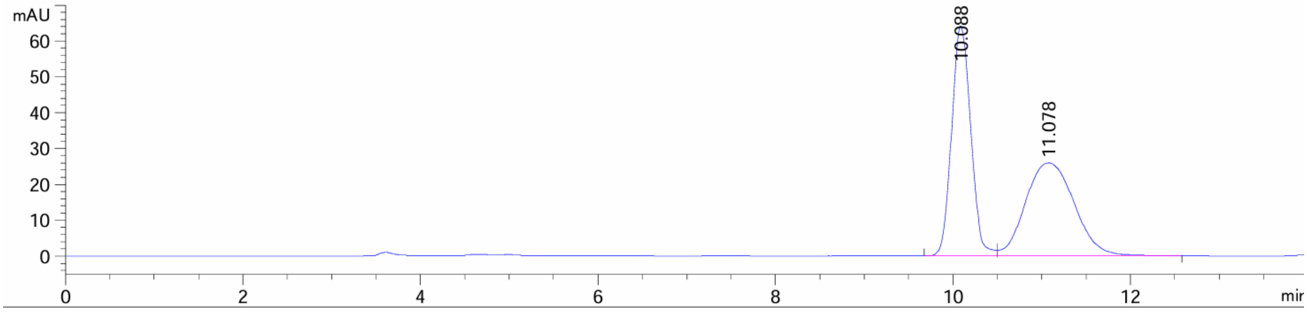

Peak #	RetTime [min]	Type	Width [min]	Area [mAU*s]	Height [mAU]	Area %
1	10.088	BV	0.2328	961.27112	64.16114	49.3718
2	11.078	VB	0.6056	985.73315	25.92734	50.6282

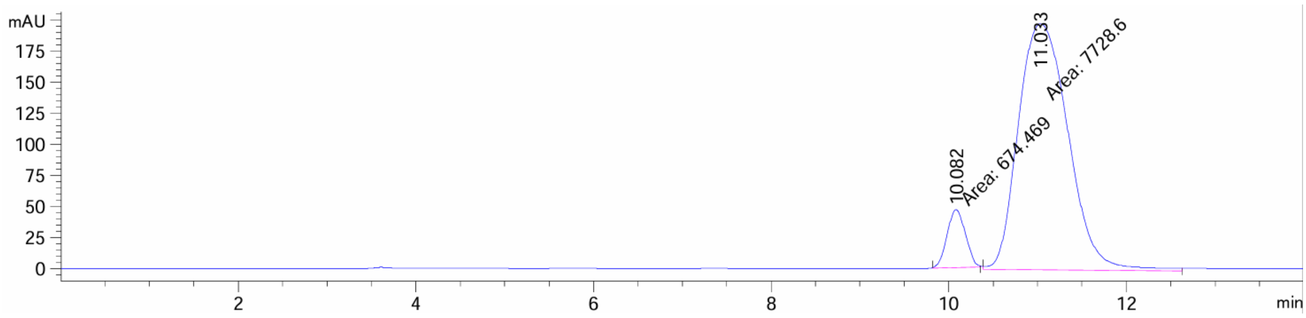

Peak #	RetTime [min]	Type	Width [min]	Area [mAU*s]	Height [mAU]	Area %
1	10.082	MM	0.2405	674.46936	46.73951	8.0265
2	11.033	MM	0.6516	7728.60303	197.68703	91.9735

8.3 DFT calculation

All DFT calculations were done within the Gaussian 16[9] and ORCA[10] program. The geometries were optimized using the B3LYP-D3[11] density functional with the def2-SVP[12] basis set. Vibrational frequency calculations were performed at the same level of theory to confirm that the stationary point is a transition state and thermal corrections at 298 K. Single-point energies were calculated at the wb97M-V[13] level with def2-TZVP[12], RIJCOSX[14] approximation to accelerate, def2/J[15] as auxiliary

---

[9] Frisch M J, Trucks G W, Schlegel H B, et al, Gaussian, Inc., Wallingford CT, 2016.

[10] Neese, F. Software Update: The ORCA Program System—Version 6.0. *Wiley Interdiscip. Rev. Comput. Mol. Sci.* **2025**, *15*, e70019.

[11] (a) Grimme, S.; Antony, J.; Ehrlich, S.; Krieg, H. A Consistent and Accurate Ab Initio Parametrization of Density Functional Dispersion Correction (DFT-D) for the 94 Elements H-Pu. *J. Chem. Phys.* **2010**, *132*, 154104. (b) Becke, A. D. Density-functional Thermochemistry. III. The Role of Exact Exchange. *J. Chem. Phys.* **1993**, *98*, 5648–5652. (c) Lee, C.; Yang, W.; Parr, R. G. Development of the Colle-Salvetti Correlation-Energy Formula into a Functional of the Electron Density. *Phys. Rev. B* **1988**, *37*, 785–789.

[12] (a) Pritchard, B. P.; Altarawy, D.; Didier, B.; Gibsom, T. D.; Windus, T. L. A New Basis Set Exchange: An Open, Up-to-Date Resource for the Molecular Sciences Community. *J. Chem. Inf. Model.* **2019**, *59*, 4814–4820. (b) Feller, D. The Role of Databases in Support of Computational Chemistry Calculations. *J. Comput. Chem.* **1996**, *17*, 1571–1586. (c) Schuchardt, K. L.; Didier, B. T.; Elsethagen, T.; Sun, L.; Gurumoorthi, V.; Chase, J.; Li, J.; Windus, T. L. Basis Set Exchange: A Community Database for Computational Sciences. *J. Chem. Inf. Model.* **2007**, *47*, 1045–1052. (d) Andrae, D.; Häußermann, U.; Dolg, M.; Stoll, H.; Preuß, H. Energy-Adjustedab Initio Pseudopotentials for the Second and Third Row Transition Elements. *Theor. Chim. Acta* **1990**, *77*, 123–141. (e) Weigend, F.; Ahlrichs, R. Balanced Basis Sets of Split Valence, Triple Zeta Valence and Quadruple Zeta Valence Quality for H to Rn: Design and Assessment of Accuracy. *Phys. Chem. Chem. Phys.* **2005**, *7*, 3297.

[13] Mardirossian, N.; Head-Gordon, M. ωB97M-V: A Combinatorially Optimized, Range-Separated Hybrid, Meta-GGA Density Functional with VV10 Nonlocal Correlation. *J. Chem. Phys.* **2016**, *144*, 214110.

[14] (a) Helmich-Paris, B.; de Souza, B.; Neese, F.; Izsák, R. An Improved Chain of Spheres for Exchange Algorithm. *J. Chem. Phys.* **2021**, *155*, 104109. (b) Izsák, R.; Neese, F. An Overlap Fitted Chain of Spheres Exchange Method. *J. Chem. Phys.* **2011**, *135*, 144105. (c) Neese, F.; Wennmohs, F.; Hansen, A.; Becker, U. Efficient, Approximate and Parallel Hartree–Fock and Hybrid DFT Calculations. A 'Chain-of-Spheres' Algorithm for the Hartree–Fock Exchange. *Chem. Phys.* **2009**, *356*, 98–109. (d) Izsák, R.; Neese, F.; Klopper, W. Robust Fitting Techniques in the Chain of Spheres Approximation to the Fock Exchange: The Role of the Complementary Space. *J. Chem. Phys.* **2013**, *139*, 094111.

[15] Weigend, F. Accurate Coulomb-Fitting Basis Sets for H to Rn. *Phys. Chem. Chem. Phys.* **2006**, *8*, 1057–1065.

basis, and SMD[16] solvation model of Et_2O. Optimized structures are illustrated with CYLview20[17]. The Reduced Density Gradient(RDG) analysis[18] was performed using Multiwfn[19]. Bonding interactions and interactions between ligands were removed from the visualization, and the results were rendered using the VMD scheme[20].

The calculation revealed that the transmetallation step is facilitated by the carbonate ligand of the rhodium complex (Supplementary Figure S8). The carbonate anion is shown to activate the approaching boronic acid and form a borate anion (**Int-2**), followed by the transfer of phenyl to generate the arylrhodium complex **Int-3**. The barriers of both transition states are moderate and the overall transmetallation is exergonic.

The head-on complexation of either nitrile group to the rhodium center (**Int-4-S** and **Int-4-R**) are both found facile, indicating a fast pre-equilibrium between these two intermediates via an dissociative mechanism (Supplementary Figure S9). As such, the subsequent migratory insertion of the nitrile is the stereodetermining step.

---

[16] (a) Garcia-Ratés, M.; Neese, F. Effect of the Solute Cavity on the Solvation Energy and Its Derivatives within the Framework of the Gaussian Charge Scheme. *J. Comput. Chem.* **2020**, *41*, 922–939. (b) Barone, V.; Cossi, M. Quantum Calculation of Molecular Energies and Energy Gradients in Solution by a Conductor Solvent Model. *J. Phys. Chem. A* **1998**, *102*, 1995–2001. (c) Marenich, A. V.; Cramer, C. J.; Truhlar, D. G. Universal Solvation Model Based on Solute Electron Density and on a Continuum Model of the Solvent Defined by the Bulk Dielectric Constant and Atomic Surface Tensions. *J. Phys. Chem. B* **2009**, *113*, 6378–6396.

[17] CYLview20; Legault, C. Y., Université de Sherbrooke, 2020 (http://www.Cylview.Org).

[18] Johnson, E. R.; Keinan, S.; Mori-Sánchez, P.; Contreras-García, J.; Cohen, A. J.; Yang, W. Revealing Noncovalent Interactions. *J. Am. Chem. Soc.* **2010**, *132*, 6498–6506.

[19] (a) Lu, T.; Chen, F. Multiwfn: A Multifunctional Wavefunction Analyzer. *J. Comput. Chem.* **2012**, *33*, 580–592. (b) Lu, T. A Comprehensive Electron Wavefunction Analysis Toolbox for Chemists, Multiwfn. *J. Chem. Phys.* **2024**, *161*, 082503. (c) Lu, T.; Chen, Q. Interaction Region Indicator: A Simple Real Space Function Clearly Revealing Both Chemical Bonds and Weak Interactions. *Chem.-Methods.* **2021**, *1*, 231–239.

[20] (a) Humphrey, W.; Dalke, A.; Schulten, K. VMD – Visual Molecular Dynamics. *J. Mol. Graph.* **1996**, *14*, 33–38. (b) Sharma, R.; Zeller, M.; Pavlovic, V. I.; Huang, T. S.; Lo, Z.; Chu, S.; Zhao, Y.; Phillips, J. C.; Schulten, K. Speech/Gesture Interface to a Visual-Computing Environment. *IEEECGA* **2000**, *20*, 29–37. (c) Varshney, A.; Brooks, F. P.; Wright, W. V. Linearly Scalable Computation of Smooth Molecular Surfaces. *IEEE Comput. Graph. Appl.* **1994**, *14*, 19–25. (d) Eargle, J.; Wright, D.; Luthey-Schulten, Z. Multiple Alignment of Protein Structures and Sequences for VMD. *Bioinformatics* **2006**, *22*, 504–506. (e) Frishman, D.; Argos, P. Knowledge-Based Secondary Structure Assignment. *Proteins: Struct. Funct. Genet.* **1995**, *23*, 566–579. (f) Sanner, M.; Olsen, A.; Spehner, J.-C. Fast and Robust Computation of Molecular Surfaces. In *Proc. 11th ACM Symp. Comput. Geom.*; ACM: New York, **1995**; pp C6–C7. (g) Stone, J. An Efficient Library for Parallel Ray Tracing and Animation. Master's Thesis, Computer Science Department, University of Missouri-Rolla, **1998**. (h) Stone, J.; Gullingsrud, J.; Grayson, P.; Schulten, K. A System for Interactive Molecular Dynamics Simulation. In *2001 ACM Symposium on Interactive 3D Graphics*; Hughes, J. F., Séquin, C. H., Eds.; ACM SIGGRAPH: New York, **2001**; pp 191–194.

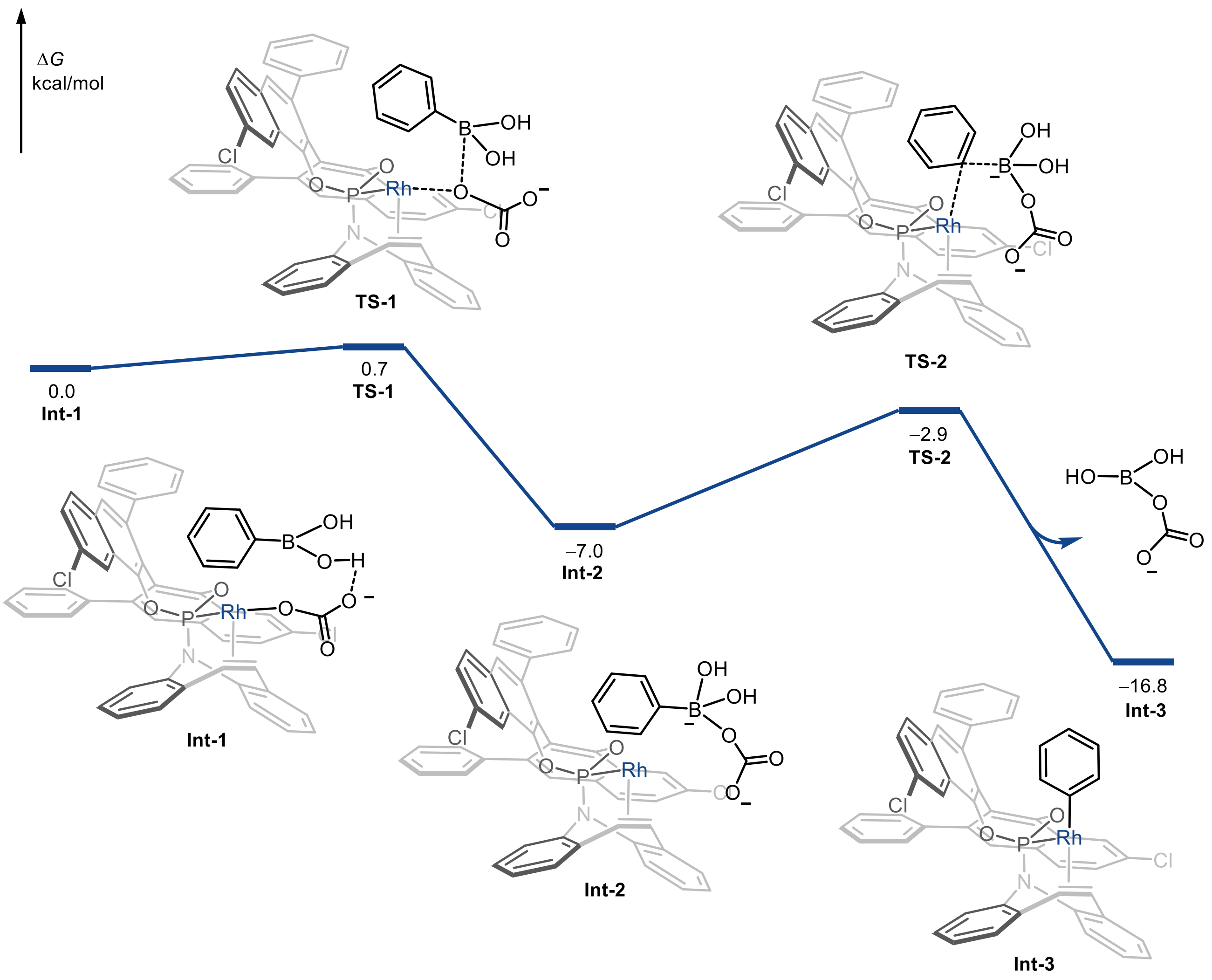

Supplementary Figure S8. Computed free-energy profile for transmetallation step (Gibbs free energies in kcal/mol, relative to **Int-1**)

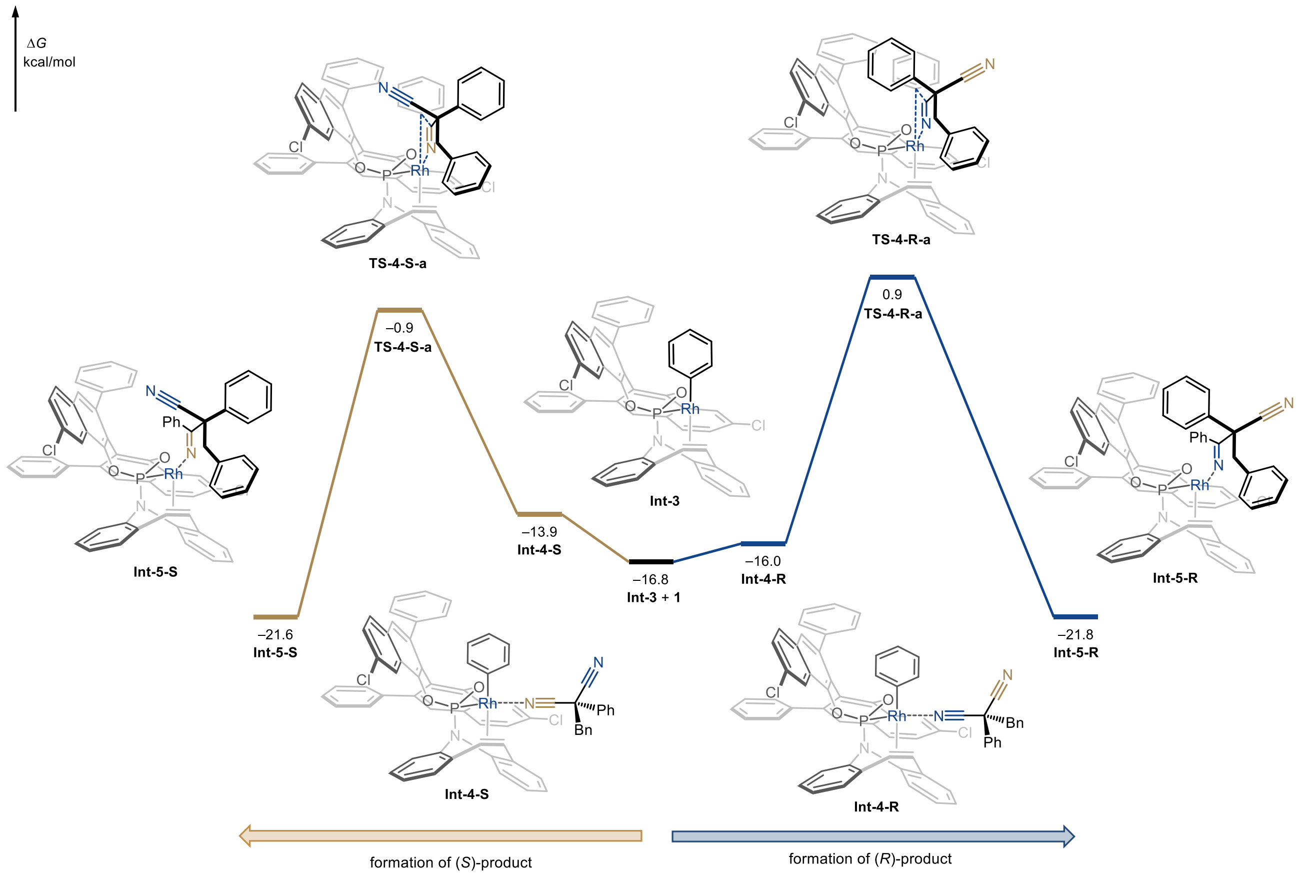

Supplementary Figure S9. Computed free-energy profile for the enantio-determining migratory insertion pathway (Gibbs free energies in kcal/mol, relative to **Int-1**).

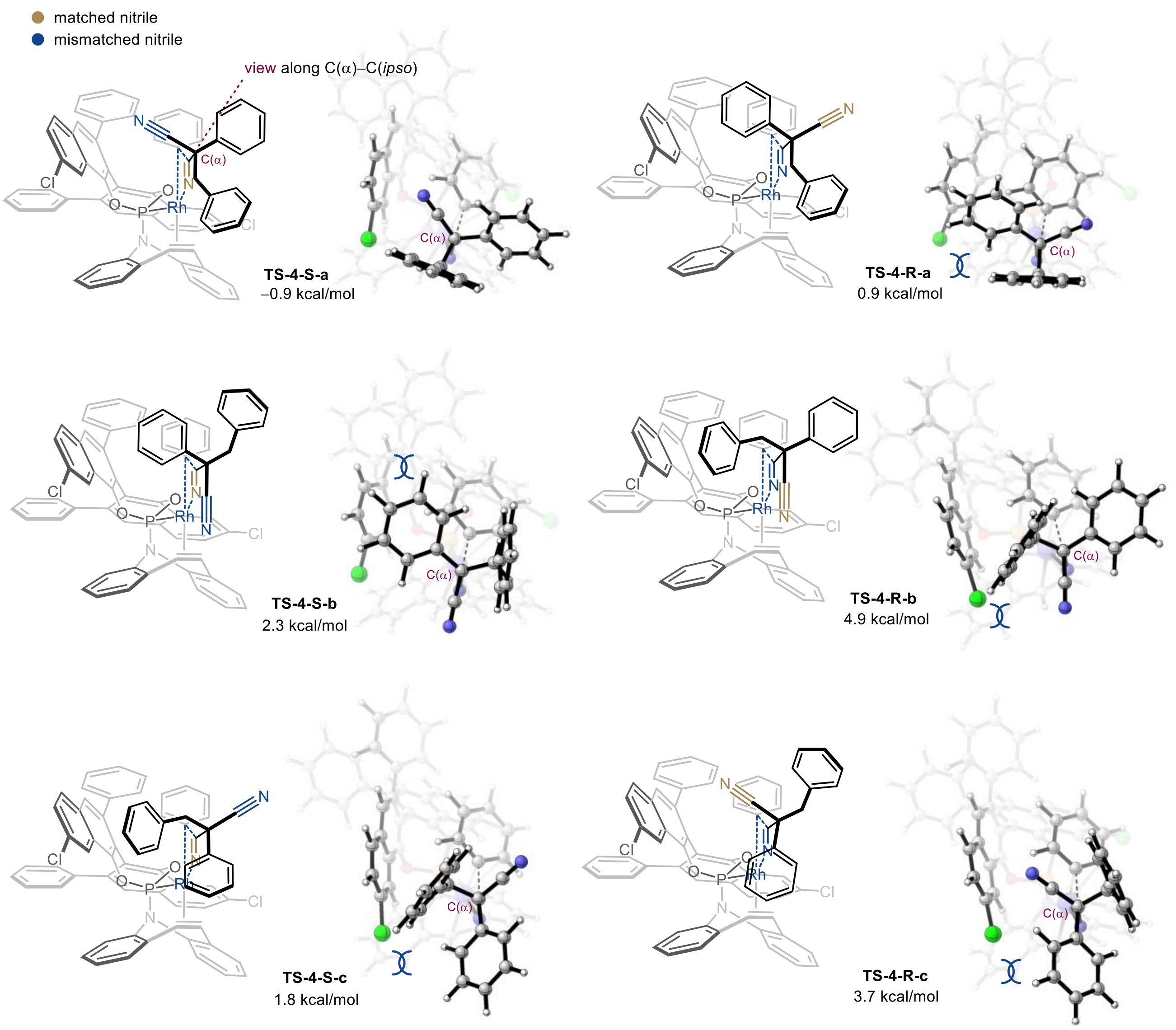

Supplementary Figure S10. Comparison of the key transition states.

The DFT calculation identified six four-membered transition states for the migratory insertion, arising from three rotamers for each enantiotopic nitrile group upon binding to the rhodium center (Supplementary Figure S10). Among the three transition states that lead to the desired enantiomers, **TS-4-S-a** directs the small, spectator nitrile towards the chlorinated naphthalene while the large phenyl and benzyl locate in a more open space. The other two transition states (i.e., **TS-4-S-b** and **TS-4-S-c**) rotate the phenyl and benzyl towards the naphthalene, respectively, resulting in enhanced steric repulsion. Meanwhile, the three transition states leading to minor enantiomers (**TS-4-R-a/b/c**) all have the phenyl or benzyl group within close proximity of the naphthalene motif.

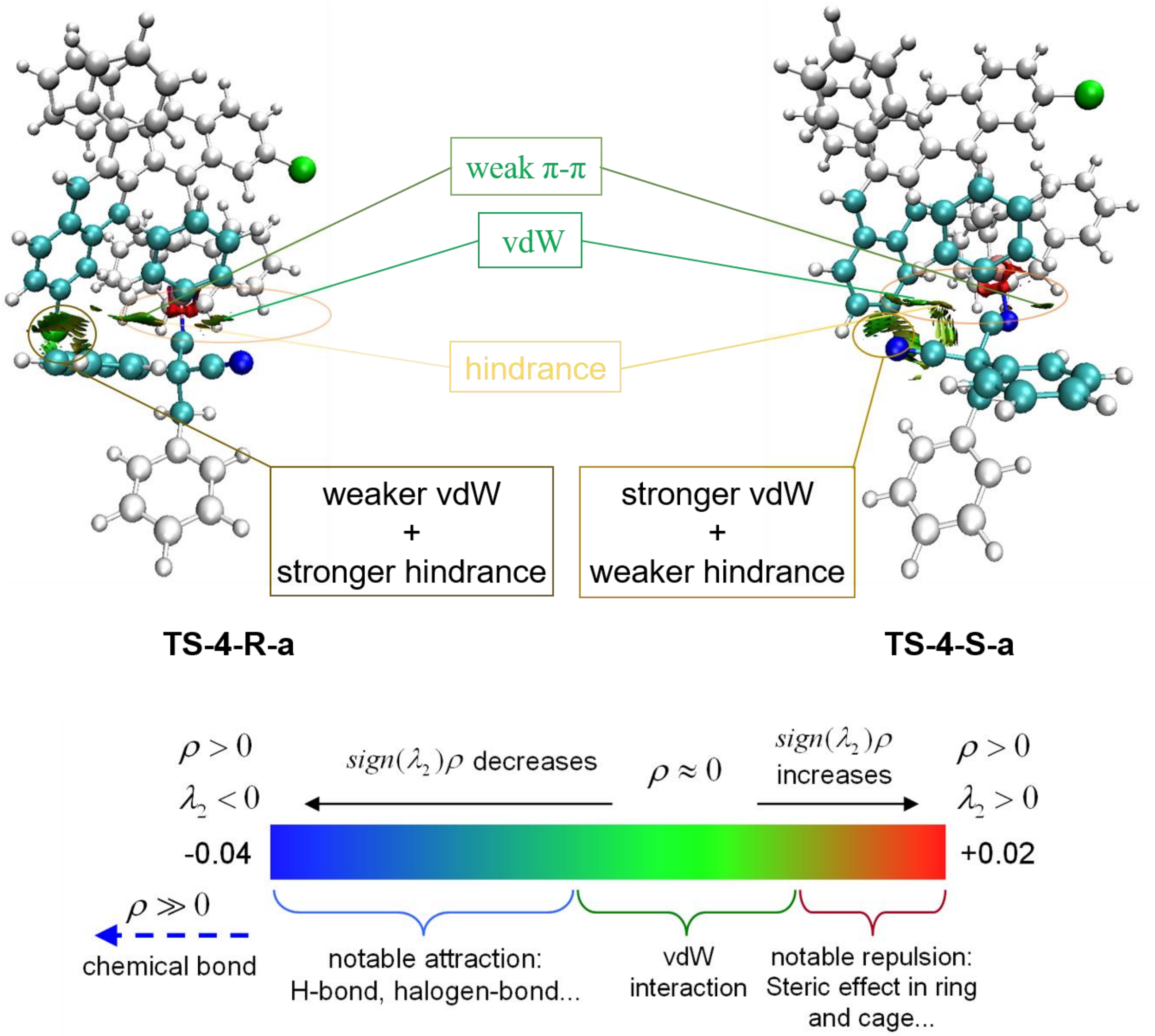

Supplementary Figure S11. NCI analysis of **TS-4-R-a** and **TS-4-S-a**.

NCI analysis of **TS-4-S-a** and **TS-4-R-a** revealed steric repulsion as the dominant factor influencing the enantioselectivity (Supplementary Figure S11). Particularly, the steric clash between the phenyl substituent of malononitrile and the chlorinated naphthalene contributes significantly to the elevated energy of transition state. On the other hand, only weak π-π interaction between the phenyl nucleophile and substituents of malononitrile

Cartesian Coordinates (Å) of Optimized Structures

Int-1

P	-0.793201	0.53281	0.372559
O	0.157172	-0.50454	1.303611
O	0.417573	1.158596	-0.60001
N	-0.926756	1.721337	1.651216

C	-1.46456	1.114459	2.846146
C	-0.689951	1.089838	4.007704
H	0.286851	1.576588	3.983658
C	-1.154368	0.432999	5.148507
H	-0.545825	0.414883	6.056534
C	-2.39213	-0.221796	5.112926
H	-2.756829	-0.751748	5.997159
C	-3.160775	-0.208183	3.949235
H	-4.122874	-0.726372	3.920657
C	-2.723014	0.466751	2.793019
C	-3.561618	0.468687	1.576579
H	-4.443193	-0.177698	1.629543
C	-3.677725	1.547918	0.642683
H	-4.627914	1.579034	0.080885
C	-2.939041	2.819621	0.713192
C	-3.546817	3.99827	0.237905
H	-4.54573	3.925924	-0.199551
C	-2.893204	5.227723	0.322907
H	-3.382874	6.129564	-0.05454
C	-1.61241	5.309527	0.882729
H	-1.090908	6.267537	0.934813
C	-0.982679	4.147899	1.33592
H	0.027455	4.172925	1.746965
C	-1.6304	2.913185	1.243882
C	0.900455	-1.445729	0.647103
C	2.09864	-1.060464	0.061217
C	2.544405	0.358688	0.205531
C	1.700217	1.396877	-0.187437
C	2.401195	-3.360127	-0.686567
H	2.958016	-4.104614	-1.26013
C	2.856336	-2.050645	-0.660008
C	3.853917	0.695655	0.705195
C	4.286242	2.010341	0.669512
H	5.269629	2.261594	1.073203
C	1.193947	-3.762433	-0.059409
C	0.405166	-2.778479	0.61004
C	-0.849715	-3.122036	1.173675
C	-1.298596	-4.418418	1.062721
C	-0.526972	-5.417401	0.419304
C	0.694501	-5.091911	-0.127058
H	-1.458751	-2.355332	1.650952
H	-0.925472	-6.430344	0.344348
H	1.283525	-5.854336	-0.643735
C	3.116395	5.390735	-0.446962

C	1.811716	5.060764	-0.893541
H	3.46027	6.423869	-0.519261
C	4.083404	-1.70757	-1.429443
C	5.21953	-2.53415	-1.361513
C	4.134487	-0.576045	-2.264933
C	6.368485	-2.240118	-2.097079
H	5.211276	-3.393612	-0.687966
C	5.284866	-0.278694	-2.998138
H	3.262777	0.074926	-2.341669
C	6.408604	-1.107779	-2.916614
H	7.244198	-2.889505	-2.014418
H	5.299605	0.605484	-3.640767
H	7.309999	-0.872044	-3.488745
C	4.746876	-0.331079	1.308855
C	6.1128	-0.371683	0.978593
C	4.257007	-1.260319	2.244823
C	6.963478	-1.313139	1.560204
H	6.497498	0.314004	0.220703
C	5.106776	-2.203932	2.824622
H	3.201457	-1.236971	2.520566
C	6.463489	-2.236125	2.48369
H	8.018153	-1.338899	1.27341
H	4.705238	-2.91759	3.548768
H	7.12651	-2.979723	2.93419
Rh	-2.748838	-0.113142	-0.335973
C	-2.564353	-0.347712	-2.955223
C	-0.722726	-1.852372	-2.500978
C	-1.645408	0.632281	-3.378937
H	-3.635173	-0.174321	-3.080591
C	0.179437	-0.896891	-2.960191
H	-0.361608	-2.824586	-2.163363
C	-0.280531	0.354841	-3.394791
H	-2.016895	1.604182	-3.714152
H	1.24906	-1.120252	-2.965828
H	0.431654	1.114665	-3.728452
C	1.325985	3.77313	-0.814319
H	0.320388	3.531733	-1.152808
C	2.150885	2.748479	-0.279479
C	3.927642	4.40065	0.064575
H	4.937496	4.643874	0.405975
C	3.478517	3.053733	0.152229
Cl	0.804089	6.337956	-1.545578
Cl	-2.878457	-4.848857	1.675118
C	-5.462187	-0.204912	-1.524317

O	-4.573685	-0.828553	-0.683846
O	-5.890871	0.925964	-1.239955
O	-5.758511	-0.87917	-2.566804
C	-2.122772	-1.616682	-2.500649
B	-3.118058	-2.854155	-2.249384
O	-4.382502	-2.942667	-2.640144
H	-4.977428	-2.058916	-2.652529
O	-2.487848	-3.952705	-1.671363
H	-3.163318	-4.620707	-1.494905

TS-1

P	-0.756239	0.543879	0.349634
O	0.1665	-0.497835	1.293556
O	0.474139	1.215229	-0.553483
N	-1.003756	1.676506	1.652985
C	-1.578519	1.006677	2.799103
C	-0.853883	0.960908	3.991784
H	0.103616	1.483755	4.031103
C	-1.338343	0.236234	5.081883
H	-0.768059	0.200727	6.013848
C	-2.545084	-0.465463	4.962431
H	-2.923791	-1.050219	5.80527
C	-3.265251	-0.428406	3.769132
H	-4.202864	-0.981967	3.674778
C	-2.808788	0.315975	2.662822
C	-3.598767	0.342235	1.418697
H	-4.471519	-0.317228	1.393164
C	-3.689875	1.437365	0.514798
H	-4.633861	1.454415	-0.043312
C	-3.003908	2.732096	0.651839
C	-3.631224	3.904771	0.187183
H	-4.613068	3.818295	-0.285076
C	-3.02217	5.151531	0.328702
H	-3.528176	6.047052	-0.042095
C	-1.76572	5.259125	0.937413
H	-1.276218	6.230524	1.032312
C	-1.119186	4.1065	1.389041
H	-0.127814	4.151584	1.842042
C	-1.724409	2.855445	1.243025
C	0.907252	-1.444763	0.639239
C	2.106754	-1.064716	0.054825
C	2.579415	0.341863	0.237738
C	1.755563	1.411527	-0.111606
C	2.364906	-3.352051	-0.745998

H	2.901489	-4.090438	-1.346212
C	2.84227	-2.05172	-0.693354
C	3.894778	0.634587	0.75119
C	4.346005	1.943124	0.782146
H	5.333494	2.158385	1.196455
C	1.158974	-3.750613	-0.112297
C	0.396261	-2.770933	0.593399
C	-0.850673	-3.108461	1.176013
C	-1.322818	-4.395197	1.046779
C	-0.585873	-5.385895	0.352027
C	0.631458	-5.066926	-0.210908
H	-1.438181	-2.345996	1.685013
H	-1.003323	-6.389671	0.258407
H	1.195504	-5.825259	-0.760453
C	3.217245	5.397372	-0.13105
C	1.90924	5.109559	-0.59694
H	3.572564	6.429113	-0.139918
C	4.059561	-1.705028	-1.475362
C	5.189682	-2.541578	-1.444453
C	4.102727	-0.557539	-2.289301
C	6.327591	-2.240365	-2.194206
H	5.186058	-3.415014	-0.789002
C	5.242275	-0.253396	-3.036527
H	3.231761	0.097178	-2.340109
C	6.361348	-1.091638	-2.991003
H	7.199797	-2.897502	-2.140913
H	5.252009	0.642688	-3.66257
H	7.254187	-0.850706	-3.574288
C	4.777181	-0.431589	1.300743
C	6.139308	-0.475859	0.955497
C	4.283762	-1.394135	2.200364
C	6.982561	-1.453042	1.486867
H	6.5264	0.23762	0.224957
C	5.125969	-2.373442	2.729826
H	3.231352	-1.369745	2.487417
C	6.478757	-2.408878	2.374081
H	8.034025	-1.480101	1.188683
H	4.72146	-3.112684	3.426092
H	7.135688	-3.180317	2.784908
Rh	-2.619347	-0.180252	-0.495152
C	-2.406339	0.133752	-2.874357
C	-0.816274	-1.630356	-2.349442
C	-1.354212	0.898611	-3.420907
H	-3.442386	0.457939	-3.014016

C	0.210357	-0.884773	-2.91447
H	-0.622576	-2.628259	-1.953903
C	-0.060419	0.389897	-3.445794
H	-1.568078	1.88062	-3.852115
H	1.226831	-1.284455	-2.941781
H	0.752376	0.982347	-3.877037
C	1.409922	3.824694	-0.599143
H	0.403957	3.615362	-0.95717
C	2.222246	2.760457	-0.125461
C	4.017113	4.368805	0.318438
H	5.029401	4.579282	0.674036
C	3.552121	3.024433	0.325479
Cl	0.916955	6.435791	-1.171287
Cl	-2.87665	-4.824436	1.717736
C	-5.503358	-0.365905	-1.491079
O	-4.371118	-1.101442	-1.022553
O	-6.138516	0.235863	-0.615915
O	-5.691941	-0.414108	-2.730486
C	-2.163846	-1.156701	-2.305937
B	-3.39475	-2.243999	-2.120674
O	-4.233573	-2.535187	-3.181549
H	-4.849789	-1.756928	-3.273545
O	-2.96992	-3.311341	-1.300862
H	-3.679779	-3.96429	-1.271884

Int-2

P	-0.765615	0.538639	0.411868
O	0.160175	-0.504538	1.343165
O	0.447899	1.203971	-0.511452
N	-1.026762	1.668124	1.712177
C	-1.668473	0.997037	2.82338
C	-0.996884	0.923336	4.045395
H	-0.028885	1.420897	4.12933
C	-1.54677	0.206273	5.109221
H	-1.017618	0.149899	6.0641
C	-2.766843	-0.459227	4.934777
H	-3.197793	-1.036969	5.757051
C	-3.433624	-0.395922	3.711751
H	-4.38108	-0.923112	3.574488
C	-2.90961	0.338501	2.629545
C	-3.64151	0.382029	1.34826
H	-4.540761	-0.240071	1.296461
C	-3.656844	1.484793	0.436898
H	-4.573147	1.531365	-0.163381

C	-2.957362	2.768417	0.625243
C	-3.541598	3.95724	0.146457
H	-4.499872	3.894109	-0.375338
C	-2.92118	5.192126	0.33611
H	-3.393696	6.100578	-0.047064
C	-1.695236	5.271112	1.0076
H	-1.196699	6.233493	1.139449
C	-1.089381	4.102142	1.472977
H	-0.119077	4.123812	1.971376
C	-1.707517	2.863805	1.281033
C	0.902292	-1.447889	0.679794
C	2.098569	-1.061391	0.093754
C	2.564769	0.348065	0.268279
C	1.733947	1.411176	-0.082398
C	2.373663	-3.349512	-0.699102
H	2.915574	-4.085598	-1.297391
C	2.839778	-2.045496	-0.652547
C	3.882354	0.651393	0.770142
C	4.326682	1.962523	0.786883
H	5.315945	2.186454	1.192263
C	1.168876	-3.753771	-0.066915
C	0.397351	-2.776458	0.63305
C	-0.846974	-3.121986	1.215619
C	-1.306899	-4.413674	1.093257
C	-0.562687	-5.401339	0.403122
C	0.652186	-5.07434	-0.1612
H	-1.442896	-2.362347	1.718412
H	-0.97218	-6.408682	0.313148
H	1.222547	-5.830012	-0.707764
C	3.176524	5.402696	-0.151771
C	1.866267	5.105004	-0.60505
H	3.527815	6.435597	-0.174724
C	4.044416	-1.688243	-1.448709
C	5.188951	-2.504607	-1.420296
C	4.056385	-0.551187	-2.278205
C	6.312591	-2.192721	-2.187179
H	5.207421	-3.370117	-0.754578
C	5.181665	-0.236509	-3.042441
H	3.170962	0.083963	-2.329519
C	6.316271	-1.053857	-2.998779
H	7.19693	-2.833665	-2.136388
H	5.167733	0.650679	-3.68095
H	7.197747	-0.804811	-3.595804
C	4.773511	-0.405456	1.323411

C	6.134836	-0.443203	0.974112
C	4.289393	-1.36517	2.231067
C	6.986288	-1.410865	1.50977
H	6.514957	0.267525	0.237268
C	5.139773	-2.335049	2.764812
H	3.237642	-1.346156	2.520914
C	6.491706	-2.363772	2.405316
H	8.036994	-1.43295	1.208506
H	4.742305	-3.07228	3.467232
H	7.155108	-3.127828	2.819514
Rh	-2.603876	-0.155729	-0.474758
C	-2.233952	0.06007	-3.038877
C	-0.79511	-1.786168	-2.415944
C	-1.121528	0.744293	-3.562098
H	-3.238652	0.475264	-3.167267
C	0.30293	-1.120152	-2.955185
H	-0.682692	-2.773325	-1.964947
C	0.140146	0.153517	-3.523705
H	-1.252072	1.733468	-4.010544
H	1.292432	-1.582306	-2.92695
H	1.00697	0.683234	-3.931328
C	1.371359	3.81849	-0.588928
H	0.363716	3.601827	-0.93783
C	2.192288	2.762305	-0.111634
C	3.98377	4.382221	0.302829
H	4.997844	4.60036	0.648566
C	3.52449	3.03613	0.326465
Cl	0.866588	6.420975	-1.189249
Cl	-2.857992	-4.852268	1.767497
C	-5.459509	-0.320861	-1.703684
O	-4.300253	-1.074074	-1.218171
O	-6.049592	0.291505	-0.810485
O	-5.651937	-0.396625	-2.927644
C	-2.102072	-1.230679	-2.453561
B	-3.460224	-2.139715	-2.055293
O	-4.218392	-2.635948	-3.136993
H	-4.829481	-1.91168	-3.393374
O	-2.997811	-3.147201	-1.129176
H	-3.738553	-3.73653	-0.942495

TS-2

P	0.82114	0.544149	-0.439137
O	-0.149934	-0.422479	-1.414221
O	-0.384813	1.253237	0.46902

N	1.182621	1.665426	-1.728154
C	1.791117	0.950585	-2.830636
C	1.129268	0.92192	-4.06008
H	0.204376	1.493643	-4.156263
C	1.627624	0.153468	-5.11323
H	1.10372	0.132312	-6.072449
C	2.785103	-0.611217	-4.920842
H	3.172142	-1.233277	-5.732564
C	3.444324	-0.590487	-3.692409
H	4.341084	-1.19673	-3.541357
C	2.975843	0.197211	-2.621947
C	3.70334	0.197397	-1.338357
H	4.530047	-0.516482	-1.272678
C	3.794979	1.295795	-0.43977
H	4.68672	1.272964	0.198186
C	3.184122	2.623442	-0.62798
C	3.8427	3.767146	-0.135568
H	4.794001	3.636179	0.386491
C	3.301565	5.041475	-0.306341
H	3.831461	5.912502	0.088759
C	2.081309	5.20716	-0.972126
H	1.641887	6.199932	-1.088184
C	1.403623	4.084856	-1.453456
H	0.436038	4.17479	-1.949655
C	1.941869	2.806698	-1.281342
C	-0.906535	-1.369753	-0.777441
C	-2.083734	-0.975387	-0.158111
C	-2.524224	0.445924	-0.303777
C	-1.663776	1.489517	0.037012
C	-2.399358	-3.28225	0.562424
H	-2.947439	-4.025081	1.146582
C	-2.832282	-1.965719	0.571894
C	-3.839607	0.780094	-0.792118
C	-4.249971	2.102214	-0.815878
H	-5.238084	2.347865	-1.211427
C	-1.218731	-3.694379	-0.109622
C	-0.434275	-2.711036	-0.785998
C	0.797673	-3.060663	-1.391863
C	1.23199	-4.364771	-1.319375
C	0.47044	-5.361365	-0.660506
C	-0.731555	-5.029039	-0.071616
H	1.403358	-2.295745	-1.87447
H	0.855789	-6.38144	-0.615529
H	-1.31338	-5.792017	0.452415

C	-2.987546	5.524051	0.039962
C	-1.680315	5.196374	0.481071
H	-3.307133	6.567459	0.044393
C	-4.0148	-1.604532	1.398342
C	-5.176349	-2.396445	1.371411
C	-3.988484	-0.485678	2.252074
C	-6.280666	-2.078261	2.163478
H	-5.22468	-3.245952	0.686844
C	-5.094687	-0.164901	3.041175
H	-3.085809	0.124049	2.307796
C	-6.247005	-0.957393	2.998987
H	-7.179146	-2.699338	2.113481
H	-5.051249	0.707209	3.698912
H	-7.11324	-0.70315	3.615912
C	-4.767769	-0.253984	-1.328497
C	-6.122873	-0.255136	-0.953868
C	-4.327473	-1.225372	-2.245939
C	-7.010401	-1.198745	-1.473636
H	-6.469472	0.465419	-0.210076
C	-5.213837	-2.171446	-2.763657
H	-3.281274	-1.23497	-2.555495
C	-6.559009	-2.164058	-2.378587
H	-8.055372	-1.192566	-1.152306
H	-4.849745	-2.918595	-3.473699
H	-7.250553	-2.909682	-2.780235
Rh	2.581417	-0.234639	0.498678
C	1.888465	0.068877	3.219626
C	0.45836	-1.688109	2.388063
C	0.848299	0.534555	4.032416
H	2.873041	0.542692	3.285555
C	-0.575306	-1.249952	3.215848
H	0.326775	-2.569573	1.760734
C	-0.38758	-0.121032	4.025395
H	1.005666	1.400347	4.682975
H	-1.532087	-1.778625	3.226755
H	-1.202917	0.236005	4.662823
C	-1.225591	3.895047	0.489679
H	-0.220613	3.655422	0.831178
C	-2.083981	2.853887	0.046285
C	-3.831311	4.518693	-0.380522
H	-4.843104	4.759738	-0.717762
C	-3.412119	3.159365	-0.382496
Cl	-0.632358	6.495155	1.019493
Cl	2.772305	-4.808188	-2.016289

C	5.313176	-0.541239	2.02788
O	4.166128	-1.256896	1.393085
O	5.979008	0.089121	1.214564
O	5.390047	-0.690748	3.254548
C	1.712365	-1.041293	2.357245
B	3.23969	-2.205371	2.092466
O	3.651382	-2.6418	3.345561
H	4.332665	-1.99197	3.642619
O	2.730304	-3.207337	1.212989
H	3.094341	-4.053841	1.498124

Int-3

P	1.356986	0.1717	0.0297
O	0.402142	-0.468097	-1.174644
O	0.291199	1.25608	0.677625
N	2.424569	1.048834	-1.014072
C	3.053362	0.148802	-1.956725
C	2.741355	0.26983	-3.312218
H	2.080525	1.08283	-3.617214
C	3.238252	-0.652065	-4.234546
H	2.987792	-0.55558	-5.293591
C	4.033909	-1.71467	-3.789824
H	4.410239	-2.452954	-4.502209
C	4.3436	-1.840927	-2.43642
H	4.956596	-2.679103	-2.095369
C	3.875733	-0.908764	-1.489671
C	4.225245	-1.074917	-0.064943
H	4.735436	-2.021108	0.153781
C	4.407828	-0.042285	0.878684
H	5.048357	-0.280463	1.736363
C	4.268001	1.402456	0.619985
C	5.075853	2.320984	1.317879
H	5.78814	1.942624	2.055973
C	4.989204	3.692171	1.078675
H	5.628171	4.380813	1.63707
C	4.087463	4.185864	0.130014
H	3.999715	5.258583	-0.049761
C	3.265281	3.295715	-0.56365
H	2.532286	3.651382	-1.288953
C	3.341328	1.922357	-0.319854
C	-0.681128	-1.179795	-0.692389
C	-1.795969	-0.477484	-0.260447
C	-1.827431	1.003794	-0.467113
C	-0.792343	1.803951	0.011207

C	-2.766602	-2.598358	0.442761
H	-3.555577	-3.153226	0.95424
C	-2.86426	-1.218693	0.357172
C	-2.916125	1.645192	-1.163112
C	-2.941088	3.025578	-1.267296
H	-3.754635	3.502427	-1.818123
C	-1.638849	-3.31639	-0.028854
C	-0.555513	-2.590197	-0.607126
C	0.62777	-3.260242	-1.010377
C	0.723862	-4.620076	-0.819195
C	-0.34318	-5.369428	-0.260971
C	-1.500099	-4.724202	0.120124
H	1.45426	-2.696076	-1.439067
H	-0.226931	-6.445946	-0.126765
H	-2.318717	-5.292154	0.568783
C	-0.948532	6.035612	-0.26705
C	0.132316	5.401459	0.397052
H	-0.969982	7.124334	-0.335003
C	-4.030271	-0.54491	0.986663
C	-5.332937	-1.029624	0.777056
C	-3.855772	0.562161	1.836894
C	-6.429867	-0.42099	1.388463
H	-5.486526	-1.866285	0.092272
C	-4.953161	1.172935	2.446979
H	-2.849853	0.939055	2.02815
C	-6.245337	0.685849	2.222889
H	-7.436061	-0.803172	1.198338
H	-4.796399	2.030625	3.106093
H	-7.104244	1.166519	2.698264
C	-4.006042	0.867052	-1.814248
C	-5.347697	1.247616	-1.635839
C	-3.726626	-0.233911	-2.643671
C	-6.380208	0.545849	-2.259552
H	-5.582503	2.075798	-0.963837
C	-4.759362	-0.937808	-3.265696
H	-2.690784	-0.535769	-2.806426
C	-6.090661	-0.552769	-3.074236
H	-7.417226	0.847744	-2.092442
H	-4.521489	-1.790019	-3.907374
H	-6.898473	-1.107558	-3.558404
Rh	2.467608	-1.08168	1.321281
C	0.857534	-1.822464	2.298148
C	0.972994	-3.211775	2.5166
C	-0.263717	-1.162982	2.837705

C	0.015655	-3.912898	3.25935
H	1.806733	-3.776834	2.079311
C	-1.221486	-1.860821	3.580984
H	-0.395058	-0.09235	2.667938
C	-1.08699	-3.237353	3.792122
H	0.123465	-4.991436	3.405896
H	-2.087856	-1.327861	3.983604
H	-1.843576	-3.783221	4.362342
C	0.207456	4.02924	0.506699
H	1.039027	3.557968	1.025894
C	-0.816535	3.226623	-0.062674
C	-1.955267	5.265579	-0.807443
H	-2.796305	5.744944	-1.31498
C	-1.925895	3.846026	-0.716718
Cl	1.392131	6.403441	1.088293
Cl	2.21465	-5.445531	-1.232219

$B(OH)_2(CO_3)$

B	-0.792972	0.182975	-0.403341
O	0.58789	0.212027	-0.065717
H	0.700448	-0.349084	0.708779
O	-1.406329	-1.071957	-0.0821
H	-2.359544	-0.933742	-0.071289
O	-1.650191	1.392874	0.101713
C	-1.733855	1.743807	-1.187309
O	-1.051602	0.762195	-1.796805
O	-2.294308	2.697357	-1.683395

1 (substrate)

C	0.205505	-1.272086	-1.149551
H	0.440076	-2.331943	-1.329969
H	-0.239689	-0.867599	-2.070123
C	-2.086045	-1.996451	-0.559375
N	-2.965439	-2.595661	-1.014901
C	-1.351595	0.245553	0.221965
C	-2.241229	0.887536	-0.650339
C	-0.764455	0.970026	1.266122
C	-2.537694	2.241804	-0.47967
H	-2.711689	0.328645	-1.46287
H	-0.063935	0.48132	1.945506
C	-1.949407	2.964171	0.563315
H	-3.236402	2.731429	-1.162296
H	-2.184061	4.022871	0.697977
C	1.444805	-0.500295	-0.772172

C	2.46574	-1.107393	-0.024291
C	1.579909	0.850336	-1.130972
C	3.59592	-0.379734	0.357827
H	2.370931	-2.155927	0.268613
C	2.710041	1.578233	-0.752609
H	0.785792	1.336857	-1.703122
C	3.720619	0.964721	-0.005426
H	4.382242	-0.866283	0.940176
H	2.800646	2.628542	-1.040632
H	4.605637	1.533213	0.291444
C	-0.939689	-1.2142	-0.049918
C	-0.462957	-1.8792	1.179617
N	-0.043396	-2.400003	2.124154
C	-1.065022	2.324234	1.435241
H	-0.600819	2.879642	2.253546

Int4-R

P	0.767256	1.248711	0.608868
O	1.513939	0.224358	1.692286
O	1.873985	1.191384	-0.616641
N	1.058095	2.704475	1.51207
C	0.573835	2.566128	2.86762
C	1.497033	2.57749	3.915218
H	2.545584	2.758806	3.673317
C	1.082677	2.324466	5.223376
H	1.810112	2.33001	6.038605
C	-0.263136	2.033519	5.478055
H	-0.592677	1.811239	6.496057
C	-1.186677	2.017359	4.434372
H	-2.232688	1.774444	4.637233
C	-0.797865	2.298643	3.109046
C	-1.811815	2.280251	2.039357
H	-2.785889	1.904552	2.364548
C	-1.804172	3.057515	0.866816
H	-2.778667	3.23442	0.400596
C	-0.771042	4.036793	0.478712
C	-1.13475	5.171116	-0.272713
H	-2.182086	5.299792	-0.559044
C	-0.190631	6.125351	-0.651407
H	-0.502598	6.993619	-1.237331
C	1.151191	5.970526	-0.286927
H	1.901997	6.700618	-0.594835
C	1.537009	4.849246	0.450187
H	2.579265	4.688114	0.72874

C	0.596407	3.886178	0.824973
C	1.551874	-1.088532	1.263711
C	2.510366	-1.453885	0.33073
C	3.557532	-0.451038	-0.042855
C	3.197364	0.820816	-0.485461
C	1.506773	-3.676335	0.283739
H	1.441937	-4.673364	-0.156869
C	2.453105	-2.789842	-0.20494
C	4.963941	-0.755527	0.067907
C	5.902462	0.183066	-0.327787
H	6.964037	-0.046949	-0.214598
C	0.539652	-3.30482	1.252605
C	0.547768	-1.967324	1.748071
C	-0.486097	-1.511138	2.605692
C	-1.523067	-2.362093	2.916624
C	-1.550971	-3.702204	2.451336
C	-0.524676	-4.161764	1.651672
H	-0.475656	-0.488377	2.975527
H	-2.400074	-4.33724	2.705674
H	-0.548738	-5.186963	1.273365
C	6.494563	2.429238	-1.230493
C	6.103971	3.669008	-1.688734
C	4.721776	3.975855	-1.770987
H	7.55729	2.18131	-1.167287
H	6.837659	4.417445	-1.992049
C	3.320336	-3.216338	-1.333803
C	3.969315	-4.46237	-1.310049
C	3.472096	-2.399256	-2.468642
C	4.759849	-4.876378	-2.383221
H	3.885521	-5.090311	-0.420456
C	4.265609	-2.81203	-3.54046
H	2.944169	-1.445527	-2.514946
C	4.915629	-4.050483	-3.500873
H	5.270868	-5.841581	-2.338361
H	4.369586	-2.165582	-4.415764
H	5.538902	-4.371334	-4.339675
C	5.455757	-2.038503	0.643216
C	6.474719	-2.757373	-0.00615
C	4.951723	-2.53993	1.856727
C	6.970359	-3.944703	0.534376
H	6.848587	-2.400806	-0.968281
C	5.444962	-3.729333	2.396256
H	4.171	-1.989142	2.38361
C	6.454494	-4.43828	1.736384

H	7.750022	-4.496646	0.003301
H	5.040158	-4.101873	3.340704
H	6.836656	-5.371584	2.157972
Rh	-1.339552	0.966238	0.213924
C	-0.927172	-0.554375	-1.127197
C	-1.382204	-1.872042	-0.917799
C	-0.276953	-0.294818	-2.352903
C	-1.20747	-2.876604	-1.877263
H	-1.871532	-2.135988	0.02296
C	-0.117289	-1.290068	-3.325932
H	0.124055	0.701549	-2.553335
C	-0.580833	-2.588999	-3.09356
H	-1.556158	-3.892173	-1.664616
H	0.384731	-1.05091	-4.268364
H	-0.44199	-3.369864	-3.845676
C	-4.202207	-0.422617	0.028062
N	-3.302765	0.295581	0.135148
C	-5.058114	-2.006293	-1.687855
H	-5.846805	-2.763541	-1.807968
H	-4.082927	-2.513134	-1.715493
C	-5.133505	-0.956316	-2.766244
C	-6.368066	-0.565512	-3.309806
C	-3.959307	-0.336124	-3.223344
C	-6.429808	0.432657	-4.285109
H	-7.287053	-1.044213	-2.96191
C	-4.022383	0.664218	-4.196837
H	-2.990927	-0.633493	-2.817602
C	-5.256295	1.052922	-4.727693
H	-7.396886	0.726207	-4.701364
H	-3.097961	1.134761	-4.540339
H	-5.304316	1.833984	-5.491001
C	-6.656359	-0.838333	-0.023424
C	-6.893753	0.519219	-0.271238
C	-7.727617	-1.679462	0.305594
C	-8.191403	1.030873	-0.189004
H	-6.068207	1.179815	-0.542889
C	-9.024064	-1.165392	0.38397
H	-7.550759	-2.738441	0.508112
C	-9.259337	0.190888	0.136755
H	-8.365266	2.091429	-0.385274
H	-9.852292	-1.82838	0.645222
H	-10.273563	0.592222	0.201597
C	5.53673	1.456431	-0.829249
C	3.757118	3.064075	-1.400363

H	2.700527	3.310032	-1.47898
C	4.150782	1.792083	-0.907172
C	-5.249311	-1.428886	-0.214056
C	-4.998082	-2.542835	0.725366
N	-4.799257	-3.457617	1.405826
Cl	4.246323	5.5599	-2.353137
Cl	-2.868438	-1.761548	3.867918

Int5-R

P	0.900557	1.233193	0.514398
O	1.490357	0.042295	1.531954
O	1.939813	1.010111	-0.762091
N	1.524033	2.552718	1.457702
C	1.082982	2.429971	2.830914
C	2.035258	2.237307	3.833221
H	3.089386	2.250343	3.551113
C	1.633442	2.00212	5.148932
H	2.38156	1.850489	5.930712
C	0.268587	1.93534	5.454006
H	-0.05443	1.733688	6.478412
C	-0.684161	2.121488	4.452941
H	-1.748407	2.062075	4.695071
C	-0.303087	2.387052	3.123172
C	-1.34525	2.592686	2.09468
H	-2.363642	2.429748	2.459716
C	-1.241947	3.464058	0.967704
H	-2.189027	3.881026	0.612421
C	-0.059731	4.266597	0.597665
C	-0.237002	5.503871	-0.051006
H	-1.252829	5.840313	-0.274828
C	0.852611	6.300597	-0.402243
H	0.685919	7.255892	-0.906151
C	2.154558	5.877754	-0.113377
H	3.01399	6.485836	-0.401172
C	2.354551	4.648568	0.51871
H	3.358097	4.281391	0.736744
C	1.266508	3.843755	0.865349
C	1.410475	-1.237287	1.037513
C	2.381304	-1.670142	0.145459
C	3.506373	-0.738238	-0.179663
C	3.236616	0.550531	-0.638001
C	1.218639	-3.81165	0.03615
H	1.110411	-4.808614	-0.396497
C	2.25862	-3.0006	-0.395341

C	4.885875	-1.133859	-0.033459
C	5.890472	-0.268718	-0.432707
H	6.932686	-0.564432	-0.295027
C	0.234865	-3.370479	0.958789
C	0.321223	-2.040509	1.468133
C	-0.671774	-1.537187	2.347962
C	-1.732517	-2.34525	2.692193
C	-1.844266	-3.671319	2.200434
C	-0.875198	-4.168352	1.354107
H	-0.591687	-0.523107	2.736671
H	-2.697951	-4.282786	2.492351
H	-0.961964	-5.187055	0.967842
C	6.64432	1.898205	-1.402965
C	6.344862	3.142944	-1.912751
C	4.988579	3.543775	-2.019892
H	7.686379	1.578831	-1.320511
H	7.131448	3.825	-2.239083
C	3.173905	-3.514816	-1.449086
C	3.675228	-4.8266	-1.377721
C	3.531971	-2.721583	-2.554628
C	4.51184	-5.328736	-2.375116
H	3.441715	-5.441265	-0.506012
C	4.37064	-3.222906	-3.55186
H	3.147782	-1.703582	-2.633168
C	4.865955	-4.528186	-3.465467
H	4.903987	-6.345332	-2.290548
H	4.635047	-2.59058	-4.403181
H	5.525376	-4.918865	-4.244606
C	5.272927	-2.434125	0.57962
C	6.261845	-3.233087	-0.020703
C	4.692303	-2.876838	1.781868
C	6.652456	-4.442423	0.555699
H	6.694285	-2.920978	-0.973661
C	5.080895	-4.087975	2.357345
H	3.936342	-2.261507	2.272586
C	6.060316	-4.877442	1.745206
H	7.41012	-5.056685	0.062699
H	4.619231	-4.413823	3.292916
H	6.360924	-5.827301	2.19477
Rh	-1.302562	1.440752	0.243816
C	-2.263314	0.14917	-1.418855
C	-1.788721	-1.178173	-1.258123
C	-1.551834	1.029544	-2.278692
C	-0.667906	-1.609449	-1.961903

H	-2.309876	-1.855544	-0.580711
C	-0.419664	0.575866	-2.977394
H	-1.950847	2.026946	-2.472022
C	0.011743	-0.737788	-2.826211
H	-0.305501	-2.628885	-1.826763
H	0.113963	1.260556	-3.639707
H	0.8906	-1.092869	-3.368832
C	-3.520003	0.680742	-0.737279
N	-3.350298	1.555721	0.157953
C	-6.041475	1.034796	-0.58782
H	-5.86992	2.064789	-0.932635
H	-5.889175	1.045748	0.499343
C	-7.434035	0.569796	-0.939307
C	-8.054523	0.993498	-2.125382
C	-8.124469	-0.324445	-0.105214
C	-9.329057	0.535194	-2.470003
H	-7.527597	1.682663	-2.790125
C	-9.398871	-0.783971	-0.446238
H	-7.651243	-0.669713	0.817189
C	-10.005531	-0.355823	-1.631277
H	-9.795222	0.876442	-3.398005
H	-9.919917	-1.480021	0.216463
H	-11.002976	-0.713994	-1.899338
C	-5.065666	-1.318881	-0.727856
C	-4.869313	-1.619724	0.629016
C	-5.45469	-2.341377	-1.601231
C	-5.078749	-2.91527	1.104128
H	-4.54428	-0.838956	1.320142
C	-5.653519	-3.641887	-1.126122
H	-5.612274	-2.120944	-2.658609
C	-5.471946	-3.932633	0.227798
H	-4.928533	-3.12187	2.165448
H	-5.959794	-4.427802	-1.82113
H	-5.635585	-4.947256	0.600137
C	5.617127	1.01043	-0.977262
C	3.959206	2.719396	-1.618391
H	2.923629	3.039914	-1.708489
C	4.258509	1.441107	-1.077679
C	-4.905841	0.138583	-1.186905
C	-4.932725	0.245104	-2.654576
N	-4.932875	0.379661	-3.805725
Cl	4.631939	5.128865	-2.673153
Cl	-2.97873	-1.718766	3.75362

Int4-S

P	1.108522	1.298019	0.335339
O	1.450336	0.148457	1.496007
O	2.410721	1.073584	-0.656539
N	1.464747	2.667223	1.346354
C	0.714412	2.600285	2.579387
C	1.405869	2.429962	3.780558
H	2.496577	2.42052	3.747624
C	0.709767	2.239863	4.974917
H	1.256155	2.102435	5.911046
C	-0.689756	2.196879	4.959817
H	-1.242931	2.027259	5.886981
C	-1.38296	2.362698	3.76205
H	-2.475084	2.316538	3.755488
C	-0.703134	2.583286	2.547139
C	-1.476764	2.759905	1.30556
H	-2.546843	2.570854	1.429472
C	-1.10955	3.534351	0.189213
H	-1.930989	3.890789	-0.440584
C	0.132074	4.31813	0.048739
C	0.12343	5.509272	-0.70272
H	-0.80868	5.825574	-1.17872
C	1.272684	6.286488	-0.845412
H	1.236184	7.204121	-1.437962
C	2.468911	5.891838	-0.236671
H	3.379146	6.481765	-0.358635
C	2.503973	4.709167	0.504667
H	3.427296	4.36148	0.969855
C	1.357642	3.922227	0.642967
C	1.371065	-1.148002	1.028778
C	2.424841	-1.649313	0.279904
C	3.668658	-0.825386	0.158216
C	3.607599	0.491654	-0.292923
C	1.12782	-3.687844	-0.045451
H	0.99708	-4.656548	-0.531698
C	2.270474	-2.952399	-0.313935
C	4.959626	-1.356561	0.524553
C	6.094266	-0.581517	0.351437
H	7.06322	-0.98243	0.656489
C	0.068212	-3.18795	0.753387
C	0.179111	-1.872094	1.292994
C	-0.90828	-1.287503	1.992887
C	-2.077858	-2.002232	2.118534
C	-2.217716	-3.313377	1.600117

C	-1.153195	-3.892171	0.941715
H	-0.818336	-0.280291	2.394889
H	-3.169595	-3.834681	1.69845
H	-1.258346	-4.893886	0.518034
C	7.109498	2.829447	-0.822342
C	5.836537	3.358867	-1.154856
H	7.998169	3.448309	-0.95553
C	3.264202	-3.494483	-1.276469
C	3.697917	-4.827904	-1.185515
C	3.756968	-2.695555	-2.323898
C	4.609077	-5.346826	-2.106651
H	3.347983	-5.447332	-0.356921
C	4.670493	-3.213885	-3.243412
H	3.399244	-1.670225	-2.42716
C	5.103073	-4.540285	-3.136693
H	4.94771	-6.38151	-2.008985
H	5.039554	-2.579887	-4.05375
H	5.820395	-4.944134	-3.855983
C	5.119022	-2.706103	1.134695
C	6.118727	-3.577513	0.667767
C	4.312985	-3.126333	2.207962
C	6.303871	-4.83284	1.248695
H	6.726283	-3.281043	-0.189887
C	4.495603	-4.383308	2.787353
H	3.54094	-2.459118	2.59433
C	5.490071	-5.243106	2.308851
H	7.075316	-5.501122	0.857748
H	3.858806	-4.690713	3.620812
H	5.629067	-6.228669	2.760735
Rh	-0.903742	1.392593	-0.453452
C	-0.52404	-0.194425	-1.72833
C	-1.257124	-1.394752	-1.642423
C	0.430315	-0.102665	-2.763799
C	-1.051797	-2.450691	-2.537336
H	-1.990475	-1.535935	-0.845039
C	0.631151	-1.151159	-3.670202
H	1.045028	0.795402	-2.859629
C	-0.105292	-2.334786	-3.558884
H	-1.634102	-3.370135	-2.423588
H	1.379902	-1.046653	-4.461513
H	0.064204	-3.159835	-4.256019
C	-5.961943	0.381104	0.623776
H	-5.234888	0.266109	1.439632
H	-6.144599	1.455186	0.472879

C	-3.964173	0.567752	-0.810848
N	-2.921162	1.068079	-0.794615
C	-6.142433	0.14551	-1.936739
C	-6.15098	1.425348	-2.507715
C	-7.007047	-0.83834	-2.429695
C	-7.018785	1.718234	-3.56194
H	-5.474455	2.198531	-2.134365
C	-7.87363	-0.542136	-3.485373
H	-7.010167	-1.836106	-1.987666
C	-7.883428	0.734585	-4.052703
H	-7.013399	2.717024	-4.004719
H	-8.544138	-1.317198	-3.863774
H	-8.559691	0.962687	-4.880056
C	-7.242251	-0.347219	0.941401
C	-7.217642	-1.502547	1.739223
C	-8.469027	0.095149	0.421472
C	-8.39706	-2.201356	2.009061
H	-6.266887	-1.854165	2.14721
C	-9.648376	-0.602366	0.692599
H	-8.497165	0.989862	-0.205675
C	-9.61425	-1.75382	1.485817
H	-8.3644	-3.098449	2.632233
H	-10.596704	-0.245792	0.282829
H	-10.536624	-2.30019	1.698734
C	4.684926	2.616722	-1.004816
H	3.716382	3.032291	-1.273705
C	4.767311	1.295882	-0.490385
C	7.200729	1.541126	-0.3413
H	8.177663	1.122039	-0.086694
C	6.040326	0.737093	-0.163464
C	-5.259077	-0.129412	-0.707224
C	-4.942011	-1.566876	-0.576927
N	-4.714576	-2.693692	-0.445446
Cl	5.74343	5.000295	-1.763823
Cl	-3.453824	-1.256295	2.929901

Int5-S

P	0.949096	1.190377	0.523336
O	1.471935	-0.01893	1.555661
O	1.999003	0.923389	-0.736773
N	1.602747	2.501696	1.456527
C	1.193237	2.396273	2.840115
C	2.165355	2.209595	3.823904
H	3.213249	2.206313	3.51906

C	1.789292	2.002325	5.152034
H	2.552385	1.854994	5.92006
C	0.43085	1.959194	5.487913
H	0.127968	1.781257	6.522804
C	-0.541499	2.13781	4.50425
H	-1.600942	2.097064	4.770341
C	-0.185957	2.373701	3.162176
C	-1.248433	2.570011	2.152364
H	-2.258248	2.40469	2.538691
C	-1.171254	3.430207	1.015818
H	-2.123727	3.851958	0.680545
C	0.009405	4.221113	0.61547
C	-0.171511	5.456086	-0.036361
H	-1.189302	5.798445	-0.242004
C	0.91586	6.24466	-0.412336
H	0.745275	7.198347	-0.918097
C	2.220259	5.816605	-0.143485
H	3.078549	6.418083	-0.448238
C	2.42414	4.590137	0.492552
H	3.429367	4.220414	0.697481
C	1.338776	3.791721	0.861843
C	1.369232	-1.295701	1.049254
C	2.362934	-1.749172	0.192811
C	3.518366	-0.843139	-0.094807
C	3.285709	0.445049	-0.572939
C	1.131757	-3.842545	-0.016162
H	1.008558	-4.825738	-0.475725
C	2.223069	-3.065477	-0.374551
C	4.8845	-1.263889	0.093928
C	5.916453	-0.418959	-0.278873
H	6.948563	-0.732563	-0.108441
C	0.1077	-3.371726	0.845665
C	0.220677	-2.058724	1.395279
C	-0.832028	-1.517081	2.179606
C	-1.98499	-2.252345	2.355243
C	-2.119132	-3.56144	1.826931
C	-1.081861	-4.109644	1.103108
H	-0.737726	-0.519003	2.604643
H	-3.05439	-4.102318	1.96643
H	-1.188272	-5.110716	0.67814
C	6.476416	2.975586	-1.76338
C	5.131205	3.397732	-1.917904
H	7.284684	3.642596	-2.067043
C	3.166681	-3.591406	-1.397038

C	3.648956	-4.908923	-1.309891
C	3.564496	-2.802901	-2.492166
C	4.510412	-5.420906	-2.280999
H	3.377629	-5.520273	-0.446706
C	4.42833	-3.314107	-3.46264
H	3.18818	-1.782618	-2.584585
C	4.90729	-4.624424	-3.35959
H	4.887507	-6.442202	-2.185082
H	4.724984	-2.686168	-4.306634
H	5.586364	-5.02277	-4.117753
C	5.226251	-2.570432	0.719941
C	6.218889	-3.388707	0.152634
C	4.596247	-3.001908	1.901232
C	6.565757	-4.60568	0.740853
H	6.689223	-3.085309	-0.785076
C	4.941211	-4.22061	2.488465
H	3.836235	-2.371999	2.366421
C	5.925128	-5.029083	1.909227
H	7.3272	-5.234887	0.273132
H	4.441335	-4.537754	3.407224
H	6.191167	-5.984975	2.367715
Rh	-1.256283	1.398672	0.305074
C	-2.249456	0.03718	-1.271166
C	-1.618605	-1.234727	-1.290898
C	-1.729078	1.062279	-2.113456
C	-0.529506	-1.468618	-2.123286
H	-1.983827	-2.025646	-0.63857
C	-0.625491	0.804463	-2.949551
H	-2.265017	2.00885	-2.197812
C	-0.03178	-0.45191	-2.954436
H	-0.043913	-2.445619	-2.108597
H	-0.243954	1.600315	-3.592901
H	0.831532	-0.648827	-3.594403
C	-5.825963	0.15647	0.487925
H	-5.344482	0.008887	1.464353
H	-5.929719	1.241051	0.35291
C	-3.455373	0.394255	-0.404967
N	-3.29622	1.353433	0.405288
C	-5.325719	-0.039175	-2.021327
C	-5.381461	1.299653	-2.441788
C	-5.773818	-1.040744	-2.889913
C	-5.881547	1.628808	-3.70244
H	-5.030568	2.088243	-1.771782
C	-6.273441	-0.711109	-4.154471

H	-5.741729	-2.085777	-2.576368
C	-6.330409	0.622258	-4.565107
H	-5.919107	2.675897	-4.013943
H	-6.622633	-1.505562	-4.818825
H	-6.721069	0.878324	-5.553166
C	-7.166574	-0.53327	0.436898
C	-7.41049	-1.670403	1.223538
C	-8.184443	-0.075556	-0.415688
C	-8.639172	-2.333225	1.160939
H	-6.622794	-2.040216	1.885219
C	-9.413713	-0.736182	-0.480395
H	-8.00604	0.80479	-1.038137
C	-9.644774	-1.868221	0.307955
H	-8.811283	-3.216784	1.781205
H	-10.194819	-0.365096	-1.149248
H	-10.606732	-2.385239	0.258854
C	4.075667	2.592813	-1.546628
H	3.048798	2.928665	-1.673409
C	4.335529	1.31424	-0.986935
C	6.737915	1.729204	-1.236808
H	7.771262	1.393214	-1.118288
C	5.682732	0.861252	-0.839228
C	-4.814854	-0.337628	-0.603151
C	-4.610362	-1.780643	-0.406926
N	-4.465543	-2.914107	-0.213983
Cl	4.821967	4.985928	-2.589418
Cl	-3.337953	-1.545052	3.213075

TS-4-S-a

P	0.898626	1.164997	0.572433
O	1.400521	-0.077806	1.575747
O	1.934049	0.906085	-0.700714
N	1.603391	2.429801	1.533580
C	1.185594	2.313771	2.913400
C	2.146050	2.065060	3.895154
H	3.193235	2.019541	3.591280
C	1.758476	1.851913	5.218899
H	2.512534	1.656136	5.985057
C	0.398833	1.866437	5.552726
H	0.086146	1.685597	6.584215
C	-0.562120	2.106541	4.571128
H	-1.622697	2.112178	4.836012

C	-0.193852	2.347685	3.233318
C	-1.243266	2.612716	2.225853
H	-2.259474	2.493746	2.611397
C	-1.117936	3.483281	1.102910
H	-2.048453	3.951940	0.767959
C	0.095796	4.234729	0.726242
C	-0.028680	5.492225	0.105239
H	-1.029522	5.882537	-0.098303
C	1.094018	6.243462	-0.242317
H	0.967904	7.216080	-0.724565
C	2.377244	5.754181	0.024382
H	3.261546	6.327326	-0.259714
C	2.524486	4.503818	0.628634
H	3.511295	4.085559	0.829647
C	1.402975	3.743536	0.969259
C	1.308846	-1.344389	1.046599
C	2.309805	-1.778529	0.189000
C	3.460338	-0.863346	-0.086624
C	3.221742	0.427171	-0.554610
C	1.095475	-3.877530	-0.057807
H	0.980896	-4.853913	-0.533764
C	2.180351	-3.085430	-0.401379
C	4.828288	-1.282260	0.092136
C	5.856292	-0.435316	-0.286505
H	6.890213	-0.747125	-0.123888
C	0.067060	-3.429370	0.810777
C	0.165048	-2.121434	1.375700
C	-0.899573	-1.595609	2.154600
C	-2.044513	-2.345397	2.316253
C	-2.160020	-3.652882	1.780846
C	-1.113947	-4.183760	1.057837
H	-0.820736	-0.597071	2.581914
H	-3.088075	-4.207298	1.916109
H	-1.206889	-5.182133	0.623639
C	6.398723	2.953192	-1.790863
C	5.051797	3.373556	-1.934465
H	7.203356	3.618538	-2.107578
C	3.125470	-3.583544	-1.436424
C	3.623526	-4.896515	-1.373914
C	3.507098	-2.772372	-2.520852
C	4.484925	-5.382356	-2.358483
H	3.363934	-5.525132	-0.519518
C	4.370826	-3.257530	-3.504752
H	3.117649	-1.755553	-2.594425

C	4.865764	-4.563610	-3.426181
H	4.874373	-6.400663	-2.281818
H	4.655015	-2.612300	-4.339966
H	5.544740	-4.941442	-4.194882
C	5.174544	-2.589470	0.714020
C	6.167837	-3.404635	0.143367
C	4.547278	-3.024963	1.895368
C	6.518583	-4.621896	0.728785
H	6.635645	-3.098551	-0.794741
C	4.896053	-4.243906	2.479803
H	3.786321	-2.397735	2.362671
C	5.881026	-5.048944	1.897535
H	7.280737	-5.248451	0.258612
H	4.398222	-4.564043	3.398646
H	6.150167	-6.005025	2.353846
Rh	-1.291352	1.469394	0.352603
C	-1.965402	0.121779	-1.381054
C	-1.354216	-1.145231	-1.417723
C	-1.549565	1.143886	-2.255685
C	-0.314522	-1.376033	-2.316808
H	-1.684775	-1.932499	-0.742643
C	-0.492204	0.899336	-3.150884
H	-2.076987	2.099995	-2.277947
C	0.119285	-0.352150	-3.174904
H	0.178027	-2.350229	-2.336314
H	-0.157230	1.690351	-3.826313
H	0.945955	-0.541651	-3.864514
C	-5.840704	0.131875	0.530787
H	-5.349448	-0.045889	1.497619
H	-6.024687	1.212345	0.457020
C	-3.519230	0.582236	-0.311534
N	-3.288903	1.532576	0.438622
C	-5.324786	0.144310	-1.986419
C	-5.402977	1.497384	-2.350522
C	-5.795075	-0.828568	-2.875312
C	-5.946591	1.869217	-3.581280
H	-5.034321	2.263014	-1.663942
C	-6.337872	-0.455315	-4.109163
H	-5.745396	-1.884279	-2.603032
C	-6.416593	0.892429	-4.466244
H	-6.000106	2.926844	-3.851902
H	-6.702579	-1.226760	-4.791902
H	-6.840119	1.182633	-5.431040
C	-7.123657	-0.652923	0.427104

C	-7.275271	-1.858608	1.130564
C	-8.174151	-0.216343	-0.396243
C	-8.448086	-2.609442	1.014886
H	-6.460474	-2.210667	1.768716
C	-9.347614	-0.965209	-0.513195
H	-8.065188	0.716771	-0.954668
C	-9.487623	-2.165020	0.191992
H	-8.550063	-3.545506	1.570100
H	-10.156076	-0.610387	-1.157783
H	-10.405859	-2.751271	0.101698
C	4.000492	2.571585	-1.545012
H	2.972080	2.906464	-1.661673
C	4.266987	1.296088	-0.981241
C	6.666507	1.710369	-1.259039
H	7.701208	1.375473	-1.149479
C	5.615930	0.843795	-0.846769
C	-4.790693	-0.214100	-0.589638
C	-4.468117	-1.647282	-0.499140
N	-4.243652	-2.778907	-0.400358
Cl	4.735326	4.955668	-2.616480
Cl	-3.414507	-1.657657	3.167105

TS-4-R-a

P	-0.816982	1.251007	-0.580364
O	-1.415839	0.047235	-1.580148
O	-1.768654	0.971500	0.752161
N	-1.561583	2.542097	-1.477830
C	-1.170920	2.462950	-2.869075
C	-2.150813	2.219195	-3.833156
H	-3.189832	2.157594	-3.505190
C	-1.793088	2.028326	-5.168507
H	-2.563290	1.835964	-5.919309
C	-0.441772	2.058990	-5.533655
H	-0.151498	1.893871	-6.574294
C	0.539270	2.296083	-4.571615
H	1.593119	2.315277	-4.861528
C	0.201182	2.516580	-3.221992
C	1.271503	2.784296	-2.238385
H	2.281120	2.688465	-2.648268
C	1.154436	3.621874	-1.094573
H	2.085422	4.088424	-0.759070
C	-0.058507	4.345068	-0.665089

C	0.067275	5.585775	-0.011074
H	1.068103	5.983818	0.176519
C	-1.055241	6.309917	0.389891
H	-0.928881	7.269670	0.897068
C	-2.338771	5.808604	0.147661
H	-3.221274	6.360388	0.476062
C	-2.486455	4.573438	-0.486737
H	-3.472389	4.143562	-0.667025
C	-1.364402	3.841546	-0.883464
C	-1.327571	-1.239542	-1.113654
C	-2.264301	-1.685678	-0.192290
C	-3.366963	-0.758384	0.210034
C	-3.069533	0.514045	0.694198
C	-1.137195	-3.848709	-0.199039
H	-1.026791	-4.859768	0.199044
C	-2.134573	-3.030130	0.309571
C	-4.753379	-1.147720	0.124306
C	-5.732448	-0.299027	0.611884
H	-6.781561	-0.588125	0.520015
C	-0.193968	-3.397962	-1.157984
C	-0.272701	-2.048246	-1.614880
C	0.697834	-1.531640	-2.512011
C	1.721590	-2.350656	-2.933295
C	1.812222	-3.702154	-2.511671
C	0.870650	-4.208869	-1.641164
H	0.632811	-0.496950	-2.845963
H	2.630487	-4.324419	-2.874975
H	0.944959	-5.245344	-1.302551
C	-6.424322	1.823209	1.717500
C	-6.092781	3.043156	2.266034
C	-4.731842	3.439546	2.311879
H	-7.470029	1.506981	1.682148
H	-6.857366	3.708565	2.669877
C	-2.992374	-3.549115	1.408067
C	-3.529037	-4.846941	1.343289
C	-3.253744	-2.772441	2.551913
C	-4.308194	-5.351988	2.384991
H	-3.369471	-5.446823	0.444907
C	-4.035057	-3.276856	3.593293
H	-2.833877	-1.768444	2.626282
C	-4.567520	-4.568026	3.513484
H	-4.730074	-6.357152	2.306434
H	-4.225393	-2.658055	4.473911
H	-5.182185	-4.960730	4.327486

C	-5.175489	-2.421031	-0.521221
C	-6.137299	-3.242671	0.092373
C	-4.656331	-2.813886	-1.768169
C	-6.561903	-4.424937	-0.515502
H	-6.520849	-2.970558	1.077984
C	-5.078625	-3.997963	-2.375286
H	-3.921897	-2.180210	-2.268218
C	-6.031133	-4.810085	-1.750352
H	-7.297777	-5.057540	-0.012683
H	-4.664516	-4.284770	-3.345284
H	-6.358416	-5.738713	-2.225008
Rh	1.362849	1.585673	-0.390229
C	1.893970	0.174527	1.300217
C	1.568855	-1.179558	1.101838
C	1.379157	0.874402	2.405905
C	0.693398	-1.811510	1.985620
H	1.985466	-1.725270	0.254448
C	0.483616	0.238850	3.274075
H	1.681928	1.907315	2.591438
C	0.146373	-1.100272	3.063076
H	0.422559	-2.856738	1.829292
H	0.062577	0.785954	4.121073
H	-0.543130	-1.604161	3.745125
C	3.553827	0.939953	0.519127
N	3.348385	1.815128	-0.309000
C	6.062806	1.051582	0.582619
H	5.955014	2.102411	0.888074
H	6.010398	1.033704	-0.514424
C	7.367509	0.473224	1.073110
C	7.925981	0.895467	2.289867
C	8.029448	-0.525291	0.340309
C	9.115258	0.334419	2.762972
H	7.417600	1.664547	2.876740
C	9.218797	-1.086974	0.810266
H	7.600828	-0.870848	-0.603537
C	9.765252	-0.658952	2.024398
H	9.535189	0.675469	3.712727
H	9.720270	-1.862878	0.225990
H	10.696135	-1.097497	2.393229
C	4.846135	-1.199023	0.667487
C	4.708566	-1.505341	-0.694525
C	5.084462	-2.231756	1.580218
C	4.829040	-2.823466	-1.136211
H	4.496413	-0.715122	-1.418404

C	5.193120	-3.553848	1.137394
H	5.190249	-2.005186	2.642486
C	5.071252	-3.853400	-0.221132
H	4.725048	-3.037068	-2.201056
H	5.379195	-4.350326	1.862107
H	5.161435	-4.886151	-0.567484
C	-5.425487	0.956299	1.193134
C	-3.729554	2.636899	1.810163
H	-2.689753	2.953867	1.852173
C	-4.062729	1.384068	1.230911
C	4.795281	0.273849	1.099955
C	4.718867	0.409106	2.564680
N	4.679125	0.558434	3.712305
Cl	-4.334659	4.991383	3.019099
Cl	2.956981	-1.707506	-3.999043

TS-4-S-b

P	0.843085	1.275470	0.499018
O	1.453240	0.114834	1.542441
O	1.743510	0.903238	-0.848537
N	1.648752	2.601862	1.281818
C	1.363594	2.599886	2.700235
C	2.410932	2.407109	3.602621
H	3.423636	2.320506	3.205194
C	2.149523	2.298614	4.969265
H	2.971376	2.146681	5.673131
C	0.828155	2.360731	5.428312
H	0.613760	2.261201	6.495333
C	-0.219845	2.545671	4.527259
H	-1.250036	2.589978	4.890027
C	0.021505	2.682604	3.146325
C	-1.115889	2.896776	2.226903
H	-2.093848	2.825330	2.710885
C	-1.085021	3.676899	1.034715
H	-2.038072	4.133422	0.751121
C	0.095798	4.370568	0.484398
C	-0.072572	5.578320	-0.219766
H	-1.083230	5.972596	-0.355458
C	1.020879	6.275950	-0.732616
H	0.861532	7.210812	-1.275778
C	2.317318	5.781144	-0.553840
H	3.176668	6.312173	-0.967055

C	2.506390	4.578352	0.130029
H	3.502234	4.155430	0.266351
C	1.413129	3.871533	0.637187
C	1.330657	-1.191525	1.139092
C	2.245240	-1.696770	0.225716
C	3.346757	-0.802954	-0.249100
C	3.043676	0.440563	-0.800424
C	1.059857	-3.825804	0.321052
H	0.919404	-4.847512	-0.038714
C	2.082852	-3.058378	-0.215992
C	4.731951	-1.197487	-0.176725
C	5.702991	-0.385799	-0.738207
H	6.752429	-0.676834	-0.656898
C	0.125536	-3.307937	1.254965
C	0.253809	-1.949161	1.673886
C	-0.706366	-1.369401	2.543541
C	-1.780002	-2.127117	2.959087
C	-1.924011	-3.482555	2.565695
C	-0.984331	-4.056785	1.736375
H	-0.601560	-0.331219	2.855456
H	-2.786546	-4.049646	2.914128
H	-1.100213	-5.096886	1.421328
C	6.038563	2.848762	-2.599260
C	4.679041	3.251249	-2.634052
H	6.796147	3.483447	-3.061508
C	2.925297	-3.648791	-1.290476
C	3.442836	-4.949005	-1.156514
C	3.188853	-2.941930	-2.478119
C	4.206853	-5.523336	-2.173260
H	3.279419	-5.494978	-0.225029
C	3.955055	-3.515459	-3.494651
H	2.785560	-1.935990	-2.605184
C	4.469478	-4.807778	-3.345563
H	4.614410	-6.528788	-2.041233
H	4.148082	-2.950162	-4.409969
H	5.072682	-5.254537	-4.140019
C	5.160093	-2.436908	0.527543
C	6.102134	-3.298244	-0.061852
C	4.664917	-2.758323	1.804368
C	6.530641	-4.450307	0.598893
H	6.466220	-3.082436	-1.068552
C	5.091288	-3.912215	2.464489
H	3.946941	-2.092239	2.285825
C	6.023728	-4.764652	1.863471

H	7.250610	-5.115167	0.115047
H	4.696399	-4.143404	3.457067
H	6.354085	-5.669607	2.379855
Rh	-1.356354	1.614194	0.457099
C	-2.216955	0.173724	-1.100322
C	-1.675217	-1.112912	-0.926406
C	-1.778185	0.995890	-2.156297
C	-0.707675	-1.575100	-1.819333
H	-2.004444	-1.736572	-0.095933
C	-0.795206	0.523529	-3.041468
H	-2.224814	1.981364	-2.307577
C	-0.270917	-0.757763	-2.872548
H	-0.273383	-2.566600	-1.682994
H	-0.443627	1.160145	-3.857015
H	0.496516	-1.127756	-3.557194
C	-5.451034	0.611364	-1.844470
H	-5.451816	1.710963	-1.908771
H	-4.624779	0.267344	-2.481441
C	-3.700554	0.890583	-0.073032
N	-3.337069	1.818207	0.648799
C	-5.021187	-1.250183	-0.055049
C	-4.621382	-2.175980	-1.029181
C	-5.317543	-1.718908	1.233412
C	-4.535861	-3.537495	-0.728830
H	-4.350632	-1.840359	-2.027783
C	-5.234803	-3.079837	1.534357
H	-5.611191	-1.015254	2.013738
C	-4.848520	-3.996265	0.552965
H	-4.221467	-4.240410	-1.504250
H	-5.468082	-3.418122	2.546621
H	-4.786260	-5.062183	0.786753
C	-6.767928	0.063465	-2.354094
C	-7.992040	0.605630	-1.925914
C	-6.796855	-0.981574	-3.291127
C	-9.204168	0.098717	-2.400507
H	-7.999497	1.430688	-1.211563
C	-8.007613	-1.487355	-3.772035
H	-5.858775	-1.402973	-3.660047
C	-9.217567	-0.952595	-3.322305
H	-10.144078	0.532948	-2.050205
H	-8.003562	-2.301263	-4.501726
H	-10.166552	-1.347563	-3.694018
C	3.685840	2.487036	-2.059731
H	2.647168	2.808737	-2.093130

C	4.027014	1.269380	-1.414163
C	6.377477	1.661648	-1.986853
H	7.422045	1.340938	-1.959274
C	5.387999	0.834747	-1.385498
C	-5.070493	0.257807	-0.361214
C	-6.030338	0.922006	0.542790
N	-6.800665	1.446659	1.229655
Cl	4.271565	4.761732	-3.420722
Cl	-3.007909	-1.406007	3.974469

TS-4-R-b

P	-0.963905	-1.256416	0.548069
O	-1.310136	-0.010007	1.616026
O	-1.949531	-0.798254	-0.707999
N	-1.823092	-2.462539	1.456334
C	-1.355879	-2.483236	2.825906
C	-2.243884	-2.147960	3.849480
H	-3.281293	-1.938879	3.582954
C	-1.794528	-2.055077	5.167493
H	-2.492143	-1.791213	5.966016
C	-0.442132	-2.276423	5.454927
H	-0.078953	-2.189237	6.481994
C	0.447863	-2.604659	4.432936
H	1.503677	-2.771566	4.661388
C	0.012618	-2.729710	3.099043
C	0.986360	-3.097399	2.049637
H	2.019802	-3.142320	2.403590
C	0.705477	-3.871758	0.889336
H	1.551425	-4.440047	0.490982
C	-0.612846	-4.416338	0.509179
C	-0.686056	-5.639492	-0.184504
H	0.242540	-6.158012	-0.437717
C	-1.913451	-6.197120	-0.542378
H	-1.940692	-7.147473	-1.081349
C	-3.105689	-5.542331	-0.215048
H	-4.069509	-5.962637	-0.507553
C	-3.056311	-4.321795	0.461730
H	-3.966826	-3.775359	0.710439
C	-1.828812	-3.755980	0.815245
C	-1.024347	1.255052	1.165185
C	-1.919228	1.874631	0.304019
C	-3.190170	1.163121	-0.037170

C	-3.147735	-0.130699	-0.552710
C	-0.403513	3.783118	0.210290
H	-0.128956	4.753530	-0.209024
C	-1.574825	3.173771	-0.214220
C	-4.478822	1.786748	0.136333
C	-5.618934	1.127547	-0.291054
H	-6.593866	1.593515	-0.132795
C	0.503366	3.156558	1.103800
C	0.196981	1.848026	1.587258
C	1.128359	1.143239	2.393585
C	2.340902	1.729425	2.686879
C	2.663517	3.035038	2.233165
C	1.754900	3.731319	1.463863
H	0.892775	0.140487	2.747092
H	3.636122	3.463194	2.480014
H	2.005310	4.730530	1.098645
C	-6.736895	-0.828373	-1.354298
C	-6.657109	-2.079036	-1.927459
C	-5.390650	-2.702389	-2.066103
H	-7.707989	-0.338095	-1.247852
H	-7.549784	-2.597541	-2.280613
C	-2.392211	3.850725	-1.256462
C	-2.679122	5.223375	-1.157034
C	-2.858982	3.148112	-2.382827
C	-3.416569	5.873667	-2.147196
H	-2.356667	5.773134	-0.270336
C	-3.598324	3.798479	-3.372749
H	-2.635472	2.085009	-2.484333
C	-3.882385	5.163350	-3.258028
H	-3.643420	6.937528	-2.041595
H	-3.951503	3.235491	-4.240464
H	-4.464704	5.670691	-4.031393
C	-4.629745	3.108781	0.803709
C	-5.463301	4.093353	0.244617
C	-3.976549	3.393620	2.016482
C	-5.632965	5.328328	0.872021
H	-5.946577	3.901002	-0.715647
C	-4.143815	4.629972	2.643000
H	-3.339866	2.635202	2.475167
C	-4.970352	5.603704	2.072024
H	-6.272019	6.085816	0.411238
H	-3.629341	4.831325	3.586058
H	-5.097677	6.572584	2.561687
Rh	1.147256	-1.851984	0.234433

C	1.877927	-0.506449	-1.400606
C	1.396069	0.817752	-1.395623
C	1.419695	-1.417581	-2.372372
C	0.455236	1.216489	-2.342494
H	1.730940	1.520736	-0.635410
C	0.453293	-1.011480	-3.310248
H	1.847677	-2.421054	-2.428981
C	-0.020847	0.298484	-3.293987
H	0.067386	2.236946	-2.325794
H	0.087284	-1.721215	-4.056344
H	-0.770098	0.617616	-4.023170
C	3.448894	-1.223814	-0.416455
N	3.113433	-2.219897	0.214630
C	4.545977	1.057008	-0.280009
H	3.949551	1.123561	0.638717
H	3.934032	1.462502	-1.093177
C	5.811642	1.866681	-0.136121
C	6.357743	2.108058	1.134563
C	6.469815	2.383402	-1.263561
C	7.532730	2.851297	1.275281
H	5.859839	1.700416	2.017571
C	7.646367	3.124015	-1.124315
H	6.057691	2.199115	-2.258460
C	8.180995	3.360848	0.146171
H	7.945363	3.028555	2.271813
H	8.147067	3.518832	-2.012249
H	9.101007	3.940977	0.255528
C	5.348715	-0.707030	-1.987804
C	4.688291	-0.181557	-3.111553
C	6.521689	-1.447951	-2.183283
C	5.190631	-0.394661	-4.396601
H	3.772721	0.396693	-2.990963
C	7.026233	-1.657098	-3.470689
H	7.052515	-1.860825	-1.323968
C	6.363852	-1.132786	-4.582407
H	4.660099	0.021382	-5.256828
H	7.945979	-2.233127	-3.599570
H	6.758994	-1.296067	-5.588209
C	-5.571594	-0.153631	-0.894669
C	-4.234308	-2.090581	-1.631480
H	-3.269410	-2.579852	-1.745529
C	-4.308365	-0.808496	-1.025866
C	4.772867	-0.467010	-0.579626
C	5.672394	-1.002606	0.460741

N	6.381463	-1.376543	1.295765
Cl	-5.313708	-4.289178	-2.802915
Cl	3.540933	0.824771	3.584283

TS-4-S-c

P	0.949386	1.302095	0.356429
O	1.198281	0.126728	1.528079
O	2.108066	0.825759	-0.732320
N	1.632326	2.591217	1.300624
C	1.001655	2.647418	2.601689
C	1.774373	2.409622	3.739659
H	2.846301	2.251162	3.610004
C	1.173043	2.345513	4.997545
H	1.781042	2.157508	5.885728
C	-0.214333	2.496856	5.109632
H	-0.695268	2.430499	6.088776
C	-0.988659	2.729011	3.973378
H	-2.072160	2.840786	4.065311
C	-0.401159	2.824445	2.696693
C	-1.257246	3.087890	1.520871
H	-2.326524	3.095760	1.748973
C	-0.878259	3.828738	0.364735
H	-1.697453	4.335407	-0.154589
C	0.447863	4.426964	0.113466
C	0.543226	5.615724	-0.635035
H	-0.372899	6.068960	-1.023056
C	1.775927	6.220247	-0.881515
H	1.820297	7.141695	-1.467468
C	2.951656	5.648636	-0.383408
H	3.922347	6.104225	-0.587109
C	2.881826	4.463791	0.352551
H	3.782230	3.981404	0.734792
C	1.650124	3.850818	0.595119
C	0.986344	-1.164613	1.111836
C	1.981103	-1.803393	0.384690
C	3.276462	-1.085475	0.170986
C	3.284201	0.188447	-0.393429
C	0.508989	-3.739510	0.203074
H	0.294133	-4.729921	-0.203877
C	1.713423	-3.128385	-0.113460
C	4.540311	-1.679936	0.530520
C	5.717109	-1.013625	0.233047

H	6.669360	-1.457043	0.531808
C	-0.494167	-3.098949	0.975939
C	-0.260393	-1.766770	1.434328
C	-1.271198	-1.059670	2.137119
C	-2.484709	-1.673096	2.358728
C	-2.742818	-2.997362	1.920611
C	-1.759939	-3.691645	1.246587
H	-1.086404	-0.041718	2.477231
H	-3.722041	-3.442568	2.102112
H	-1.957576	-4.707048	0.894060
C	6.914609	2.166865	-1.344779
C	5.668345	2.761501	-1.668949
H	7.838191	2.693645	-1.589599
C	2.656796	-3.835898	-1.020242
C	2.935620	-5.201064	-0.832027
C	3.260538	-3.172170	-2.104429
C	3.796223	-5.881230	-1.694361
H	2.506810	-5.720218	0.027589
C	4.122590	-3.852554	-2.966568
H	3.048025	-2.115911	-2.275366
C	4.396094	-5.209231	-2.763905
H	4.012850	-6.938040	-1.519448
H	4.580052	-3.319507	-3.803889
H	5.074062	-5.740000	-3.437094
C	4.623293	-2.978554	1.253826
C	5.532995	-3.964977	0.834050
C	3.827302	-3.238690	2.384000
C	5.638205	-5.178401	1.515245
H	6.130853	-3.792294	-0.063365
C	3.930357	-4.453529	3.064152
H	3.128420	-2.478127	2.736109
C	4.833892	-5.429844	2.630894
H	6.339357	-5.938341	1.161219
H	3.304353	-4.635922	3.941337
H	4.910830	-6.382089	3.161941
Rh	-1.134486	1.768258	-0.239052
C	-1.633346	0.349130	-1.924046
C	-1.066732	-0.936532	-1.811389
C	-1.089197	1.278528	-2.833149
C	0.029481	-1.282048	-2.597753
H	-1.457529	-1.650921	-1.088990
C	0.031693	0.926105	-3.606208
H	-1.568411	2.249220	-2.978601
C	0.581901	-0.347939	-3.489048

H	0.473655	-2.274298	-2.500758
H	0.457404	1.648838	-4.306521
H	1.450744	-0.625199	-4.091604
C	-4.324143	-1.274998	-0.759590
H	-3.500397	-1.779785	-1.278324
H	-3.991051	-1.074824	0.264885
C	-3.298486	0.984422	-1.153339
N	-3.104381	2.005152	-0.494000
C	-5.804112	0.805908	-0.890855
C	-5.865492	1.126053	0.474915
C	-6.921373	1.050325	-1.696369
C	-7.024186	1.679037	1.019250
H	-5.004811	0.941982	1.117600
C	-8.083238	1.605918	-1.148722
H	-6.893695	0.800680	-2.758554
C	-8.139377	1.921745	0.209390
H	-7.055709	1.922322	2.084449
H	-8.947303	1.788220	-1.792611
H	-9.047109	2.354721	0.637470
C	-5.541758	-2.165323	-0.743124
C	-5.927109	-2.880906	-1.887938
C	-6.315308	-2.287573	0.421862
C	-7.065457	-3.691213	-1.870654
H	-5.334843	-2.795779	-2.802335
C	-7.450598	-3.102105	0.442184
H	-6.023021	-1.735599	1.318234
C	-7.831088	-3.803942	-0.705637
H	-7.354087	-4.239107	-2.771429
H	-8.042078	-3.185251	1.357725
H	-8.720761	-4.438921	-0.692206
C	4.474203	2.137439	-1.377716
H	3.525616	2.603157	-1.636057
C	4.486958	0.874079	-0.729983
C	6.936790	0.933075	-0.731302
H	7.892567	0.464917	-0.482228
C	5.731261	0.248329	-0.411315
C	-4.539165	0.131631	-1.441018
C	-4.611312	-0.056714	-2.900512
N	-4.640843	-0.234758	-4.044612
Cl	5.665158	4.328558	-2.450961
Cl	-3.768824	-0.782027	3.157061

TS-4-R-c

P	0.877078	1.237738	0.356874
O	1.400334	0.176917	1.540376
O	1.919729	0.804366	-0.861760
N	1.559599	2.648261	1.108428
C	1.128461	2.749308	2.485315
C	2.080536	2.667572	3.502816
H	3.131053	2.582838	3.219600
C	1.680830	2.664786	4.839969
H	2.428286	2.599466	5.634317
C	0.317724	2.721409	5.154970
H	-0.004229	2.704355	6.199237
C	-0.634772	2.794501	4.139205
H	-1.698016	2.833816	4.390063
C	-0.254329	2.822716	2.783455
C	-1.294691	2.917238	1.737342
H	-2.314416	2.851507	2.126302
C	-1.161968	3.596405	0.491111
H	-2.092258	3.994410	0.074470
C	0.049706	4.289176	0.010760
C	-0.076967	5.431237	-0.802996
H	-1.078207	5.775283	-1.076147
C	1.044261	6.128288	-1.252889
H	0.916905	7.011190	-1.884325
C	2.327851	5.699148	-0.898477
H	3.210732	6.228745	-1.260677
C	2.477277	4.561038	-0.103212
H	3.464578	4.187988	0.170956
C	1.357213	3.854145	0.341415
C	1.349145	-1.158657	1.213646
C	2.371969	-1.692995	0.443117
C	3.495753	-0.797886	0.027164
C	3.220277	0.391459	-0.644604
C	1.218621	-3.839229	0.523832
H	1.137888	-4.882138	0.209348
C	2.286682	-3.080979	0.068502
C	4.875077	-1.139891	0.270498
C	5.878525	-0.337382	-0.245658
H	6.920823	-0.586658	-0.035578
C	0.163795	-3.287334	1.295702
C	0.219873	-1.904458	1.646802
C	-0.874604	-1.286812	2.306844
C	-2.010619	-2.025580	2.557631
C	-2.081453	-3.406413	2.241684

C	-1.005699	-4.021973	1.639222
H	-0.830146	-0.229495	2.563183
H	-3.001596	-3.953794	2.444080
H	-1.065792	-5.079835	1.372016
C	6.626976	1.636082	-1.569243
C	6.323562	2.761876	-2.303896
C	4.965126	3.112725	-2.511398
H	7.670885	1.354554	-1.408731
H	7.108748	3.386259	-2.732915
C	3.259876	-3.711939	-0.863130
C	3.792233	-4.982564	-0.583718
C	3.635566	-3.076416	-2.060879
C	4.680978	-5.596107	-1.467557
H	3.536820	-5.472017	0.358476
C	4.526754	-3.688942	-2.944089
H	3.220275	-2.096494	-2.302253
C	5.055634	-4.950168	-2.649816
H	5.096495	-6.576886	-1.222742
H	4.805968	-3.179158	-3.869756
H	5.756092	-5.427391	-3.339954
C	5.257323	-2.314538	1.100642
C	6.278489	-3.181910	0.674453
C	4.635947	-2.567162	2.337067
C	6.662608	-4.273359	1.454588
H	6.742062	-3.021952	-0.301297
C	5.018162	-3.660519	3.116479
H	3.852967	-1.895197	2.692432
C	6.031154	-4.519818	2.677449
H	7.446333	-4.945394	1.095888
H	4.524339	-3.839057	4.075049
H	6.326563	-5.377867	3.286747
Rh	-1.313907	1.482260	0.072033
C	-1.926716	-0.137713	-1.412103
C	-1.308479	-1.390641	-1.241925
C	-1.517259	0.723596	-2.449061
C	-0.269415	-1.763757	-2.093001
H	-1.630681	-2.056273	-0.443529
C	-0.460442	0.339081	-3.294782
H	-2.042289	1.665601	-2.625660
C	0.158098	-0.895722	-3.111222
H	0.229111	-2.724538	-1.948987
H	-0.129933	1.008940	-4.092513
H	0.985254	-1.193333	-3.760938
C	-3.544351	0.501124	-0.449685

N	-3.312103	1.568847	0.108331
C	-5.196222	-0.323869	-2.153414
H	-4.308441	-0.603786	-2.739299
H	-5.421136	0.727553	-2.383615
C	-6.366687	-1.209608	-2.504733
C	-6.155934	-2.530487	-2.930189
C	-7.685717	-0.744267	-2.380534
C	-7.235978	-3.366460	-3.225796
H	-5.134652	-2.909022	-3.021138
C	-8.766853	-1.577706	-2.676376
H	-7.864683	0.278134	-2.039749
C	-8.545007	-2.892122	-3.099653
H	-7.053272	-4.392394	-3.555588
H	-9.787124	-1.198928	-2.574476
H	-9.390653	-3.544853	-3.331434
C	-5.937701	0.118372	0.267884
C	-6.367040	1.451296	0.167201
C	-6.599866	-0.740447	1.152522
C	-7.442080	1.909564	0.929270
H	-5.847960	2.140653	-0.499151
C	-7.677061	-0.279982	1.917121
H	-6.278486	-1.778110	1.249662
C	-8.104105	1.044094	1.807261
H	-7.760941	2.951236	0.840582
H	-8.182689	-0.966499	2.600778
H	-8.946053	1.403570	2.404312
C	5.601701	0.821972	-1.011927
C	3.937235	2.359638	-1.985646
H	2.899553	2.640583	-2.152375
C	4.240295	1.206324	-1.214943
C	-4.789873	-0.369778	-0.629986
C	-4.421935	-1.757103	-0.298364
N	-4.146510	-2.859938	-0.079046
Cl	4.603505	4.544716	-3.452995
Cl	-3.424132	-1.236641	3.221207

Section 9. Crystal structures and crystallographic data

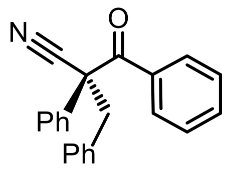

(+)-**2**

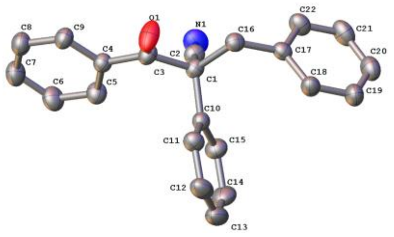

Supplementary Figure S12. Crystal structure for (+)-**2** (CCDC 2469018).

**Table 1 Crystal data and structure refinement for 250701zmh_1.**	
Identification code	250701zmh_1
Empirical formula	$C_{22}H_{17}NO$
Formula weight	311.36
Temperature/K	296
Crystal system	orthorhombic
Space group	$P2_12_12_1$
a/Å	6.7896(3)
b/Å	15.1434(7)
c/Å	17.0449(8)
α/°	90
β/°	90
γ/°	90
Volume/Å^3	1752.52(14)
Z	4
ρ_{calc}g/cm^3	1.180
μ/mm^{-1}	0.563
F(000)	656.0
Crystal size/mm^3	0.17 × 0.17 × 0.05
Radiation	CuKα (λ = 1.54178)
2θ range for data collection/°	7.81 to 144.382

Index ranges	-8 ⩽ h ⩽ 8, -17 ⩽ k ⩽ 18, -21 ⩽ l ⩽ 20
Reflections collected	16283
Independent reflections	3411 [R_{int} = 0.0475, R_{sigma} = 0.0340]
Data/restraints/parameters	3411/0/217
Goodness-of-fit on F^2	1.129
Final R indexes [I>=2σ (I)]	R_1 = 0.0782, wR_2 = 0.1864
Final R indexes [all data]	R_1 = 0.0836, wR_2 = 0.1950
Largest diff. peak/hole / e $Å^{-3}$	0.30/-0.38
Flack parameter	0.08(17)

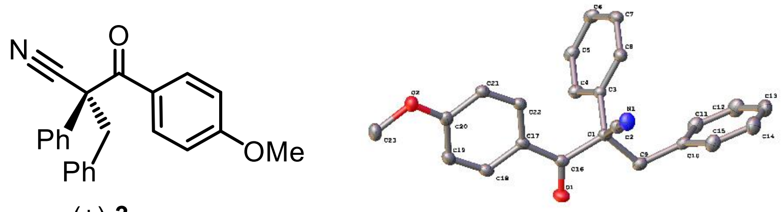

(+)-**3**

Supplementary Figure S13. Crystal structure for (+)-**3** (CCDC 2501542).

**Table 1 Crystal data and structure refinement for zmh_4.**	
Identification code	zmh_4
Empirical formula	$C_{23}H_{19}NO_2$
Formula weight	341.39
Temperature/K	170.00
Crystal system	monoclinic
Space group	$P2_1$
a/Å	8.6102(3)
b/Å	10.5392(3)
c/Å	10.1345(3)
α/°	90
β/°	96.1400(10)
γ/°	90
Volume/$Å^3$	914.38(5)
Z	2
ρ_{calc}g/cm^3	1.240

μ/mm^{-1}	0.415
F(000)	360.0
Crystal size/mm^3	0.18 × 0.17 × 0.12
Radiation	GaKα (λ = 1.34139)
2θ range for data collection/°	7.632 to 121.324
Index ranges	-11 ≤ h ≤ 11, -13 ≤ k ≤ 13, -13 ≤ l ≤ 13
Reflections collected	14065
Independent reflections	4191 [R_{int} = 0.0564, R_{sigma} = 0.0589]
Data/restraints/parameters	4191/1/236
Goodness-of-fit on F^2	1.063
Final R indexes [I>=2σ (I)]	R_1 = 0.0328, wR_2 = 0.0871
Final R indexes [all data]	R_1 = 0.0340, wR_2 = 0.0881
Largest diff. peak/hole / e Å^{-3}	0.18/-0.12
Flack parameter	0.03(13)

(+)-**76**

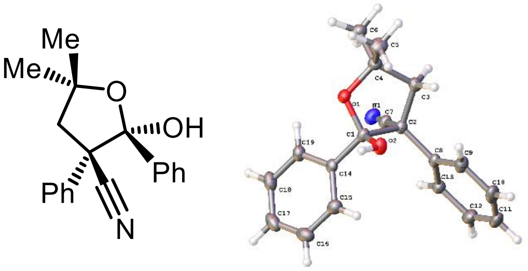

Supplementary Figure S14. Crystal structure for (+)-**76** (CCDC 2481529).

**Table 1 Crystal data and structure refinement for 280819zmh_3.**	
Identification code	280819zmh_3
Empirical formula	$C_{19}H_{19}NO_2$
Formula weight	293.35
Temperature/K	273.15
Crystal system	orthorhombic
Space group	$P2_12_12_1$
a/Å	9.2876(2)
b/Å	12.3449(2)
c/Å	14.2973(3)

$\alpha/°$	90
$\beta/°$	90
$\gamma/°$	90
Volume/$Å^3$	1639.25(6)
Z	4
$\rho_{calc}g/cm^3$	1.189
μ/mm^{-1}	0.610
F(000)	624.0
Crystal size/mm^3	0.17 × 0.17 × 0.05
Radiation	CuKα (λ = 1.54178)
2θ range for data collection/°	9.464 to 140.224
Index ranges	-11 ≤ h ≤ 11, -15 ≤ k ≤ 15, -17 ≤ l ≤ 17
Reflections collected	33258
Independent reflections	3122 [R_{int} = 0.0539, R_{sigma} = 0.0247]
Data/restraints/parameters	3122/0/203
Goodness-of-fit on F^2	1.066
Final R indexes [I>=2σ (I)]	R_1 = 0.0292, wR_2 = 0.0762
Final R indexes [all data]	R_1 = 0.0303, wR_2 = 0.0773
Largest diff. peak/hole / e $Å^{-3}$	0.19/-0.13
Flack parameter	0.07(7)

Section 10. Copies of spectra

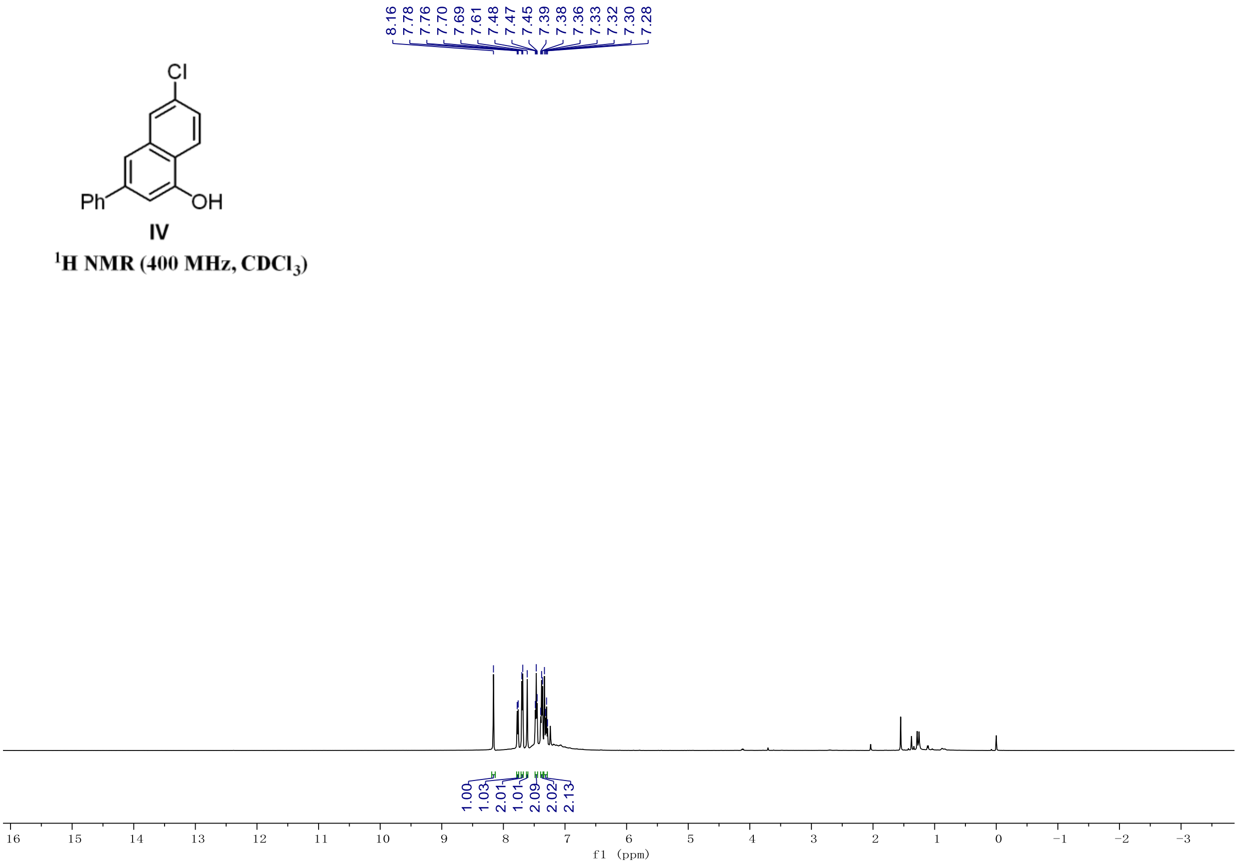

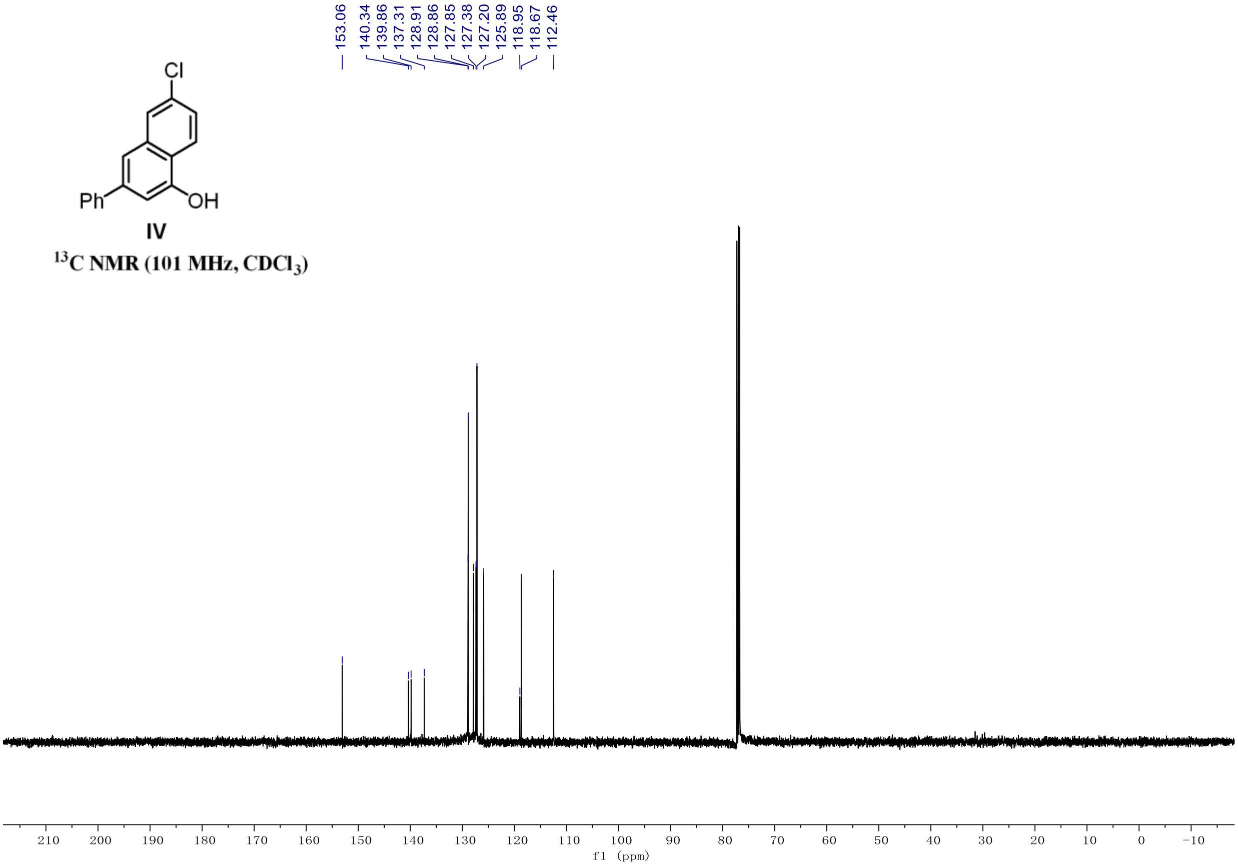

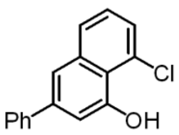

V

^{1}H NMR (400 MHz, $CDCl_3$)

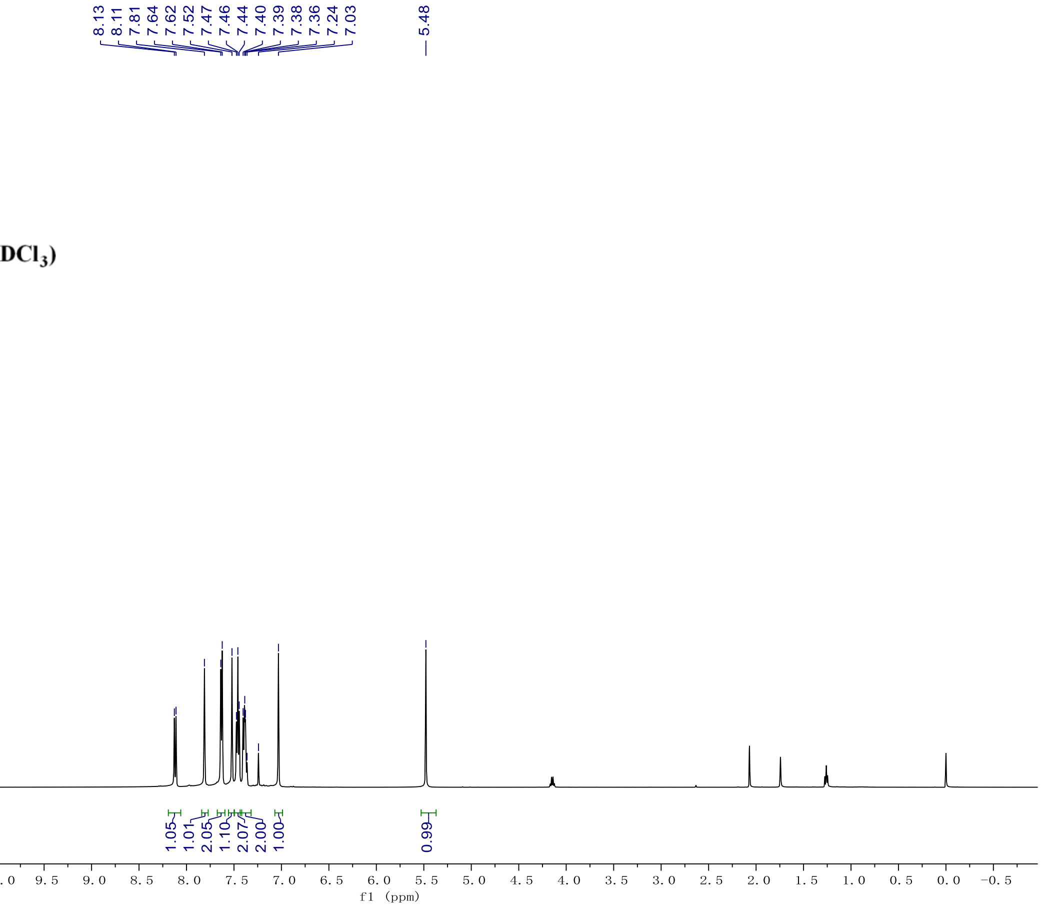

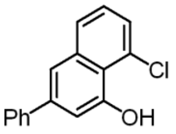

V

^{13}C NMR (101 MHz, $CDCl_3$)

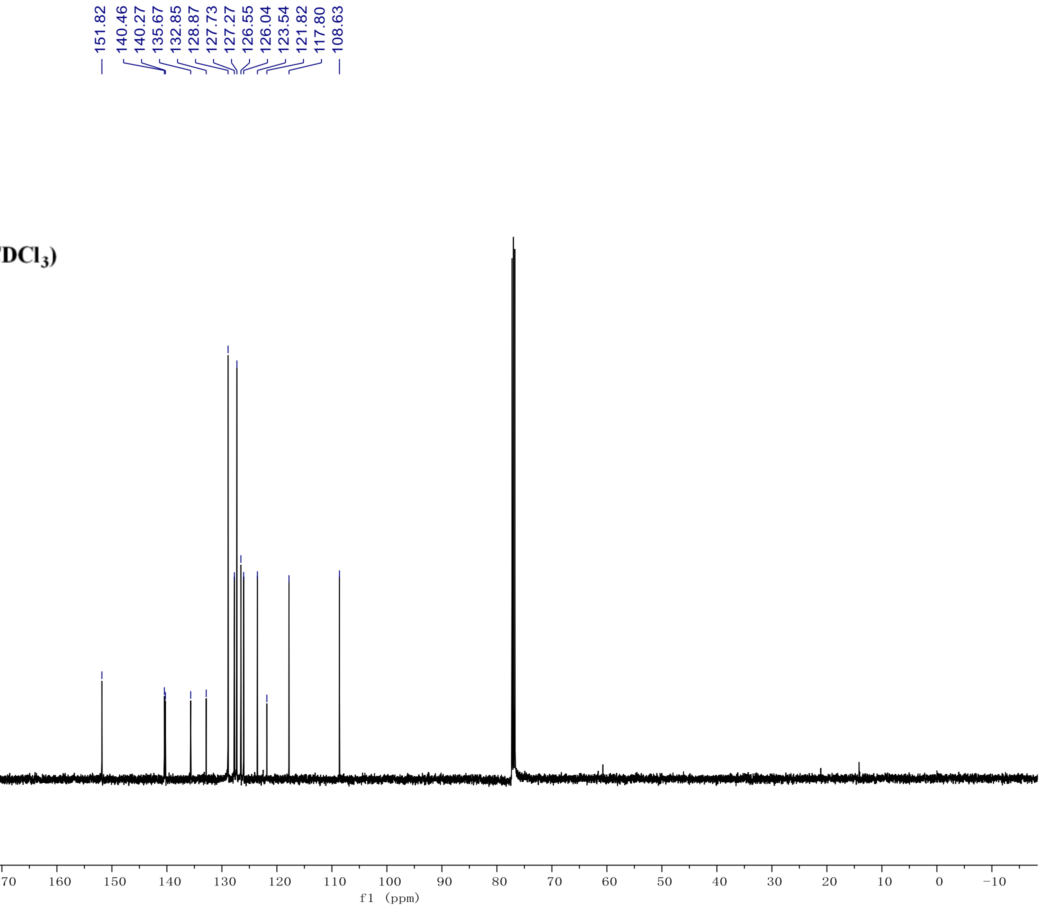

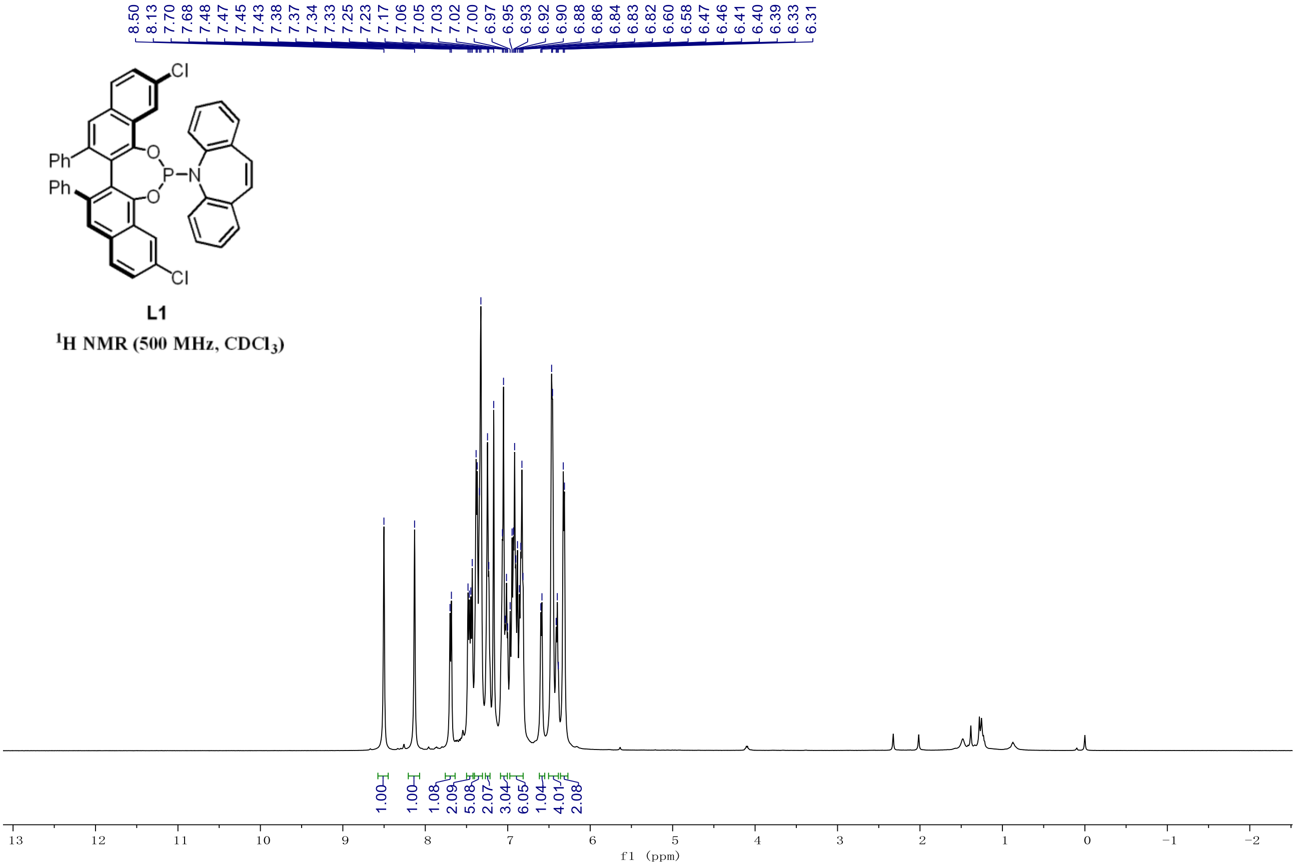

L1

^{1}H NMR (500 MHz, CDCl$_3$)

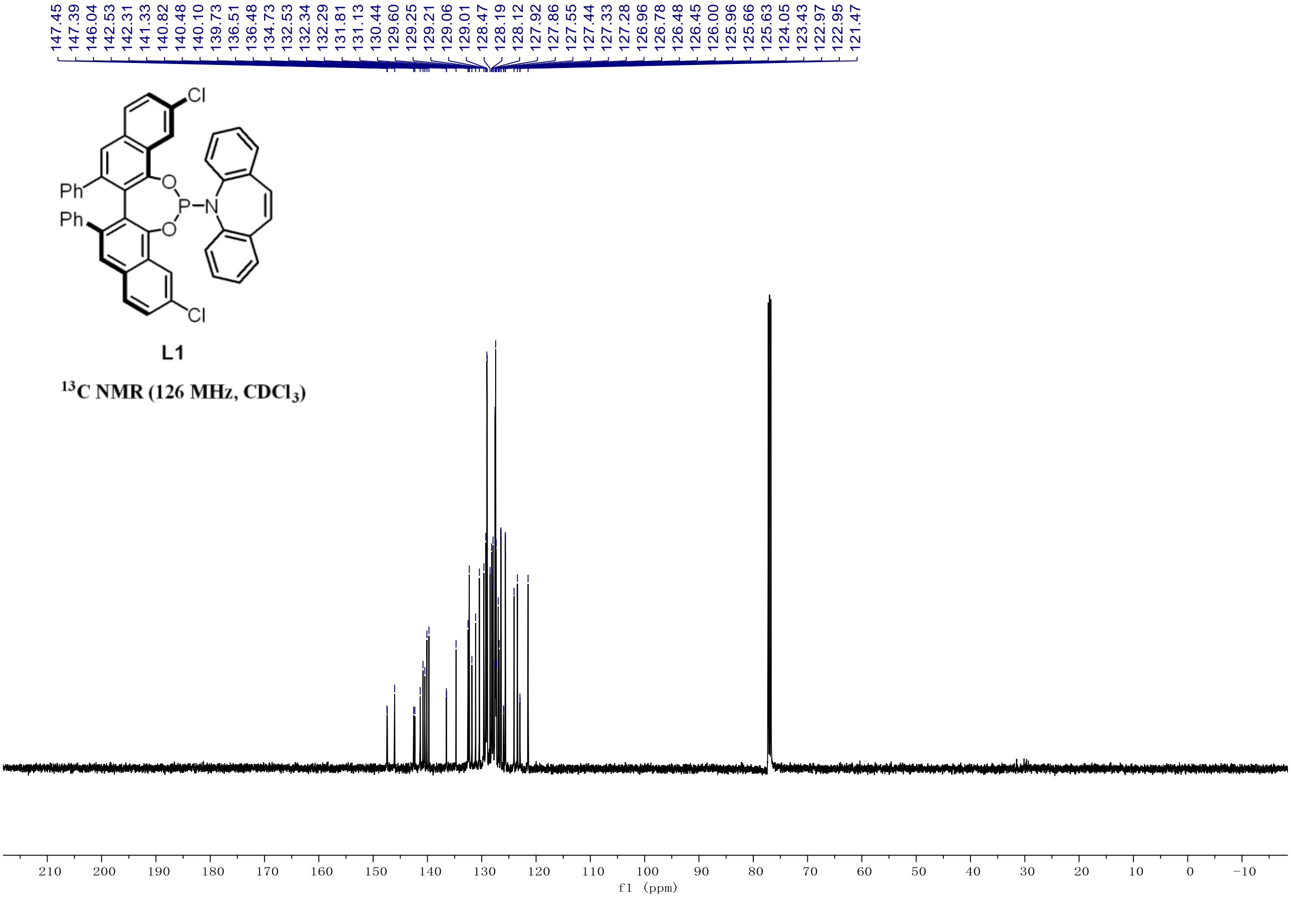

L1

^{13}C NMR (126 MHz, CDCl$_3$)

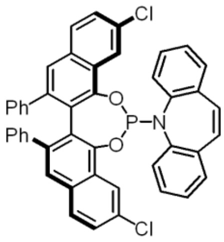

L1

^{31}P NMR (202 MHz, $CDCl_3$)

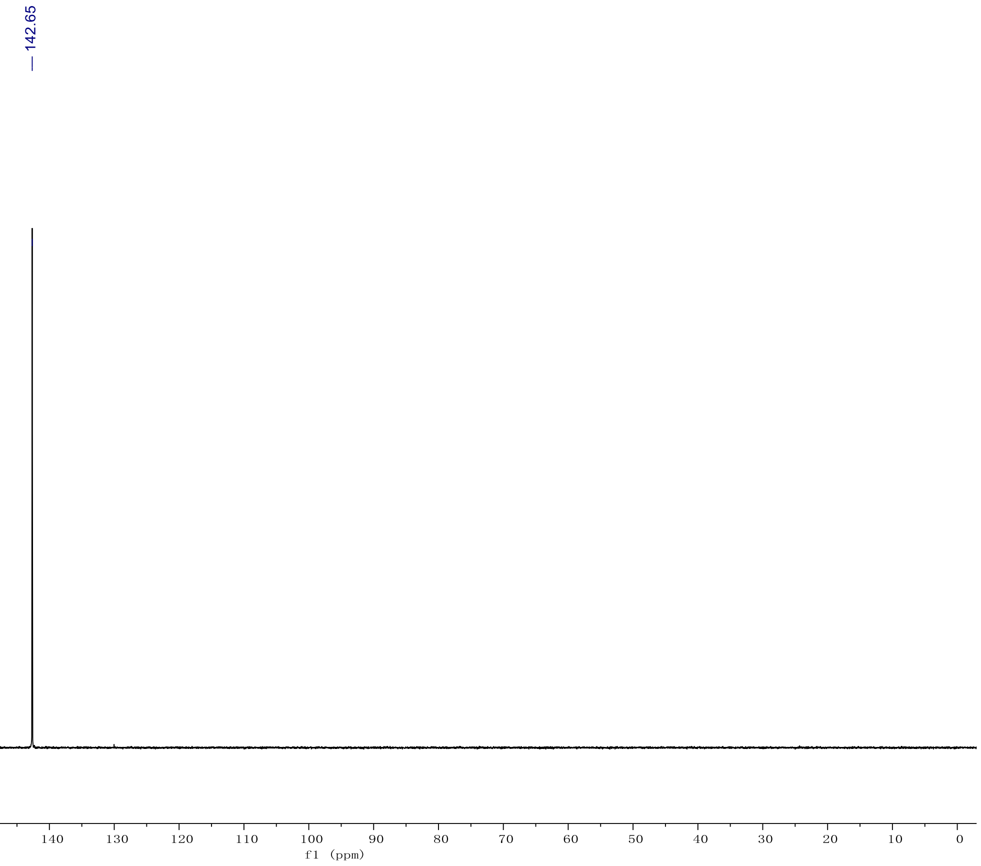

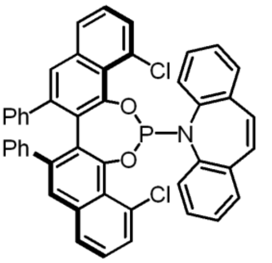

L6

^{1}H NMR (600 MHz, $CDCl_3$)

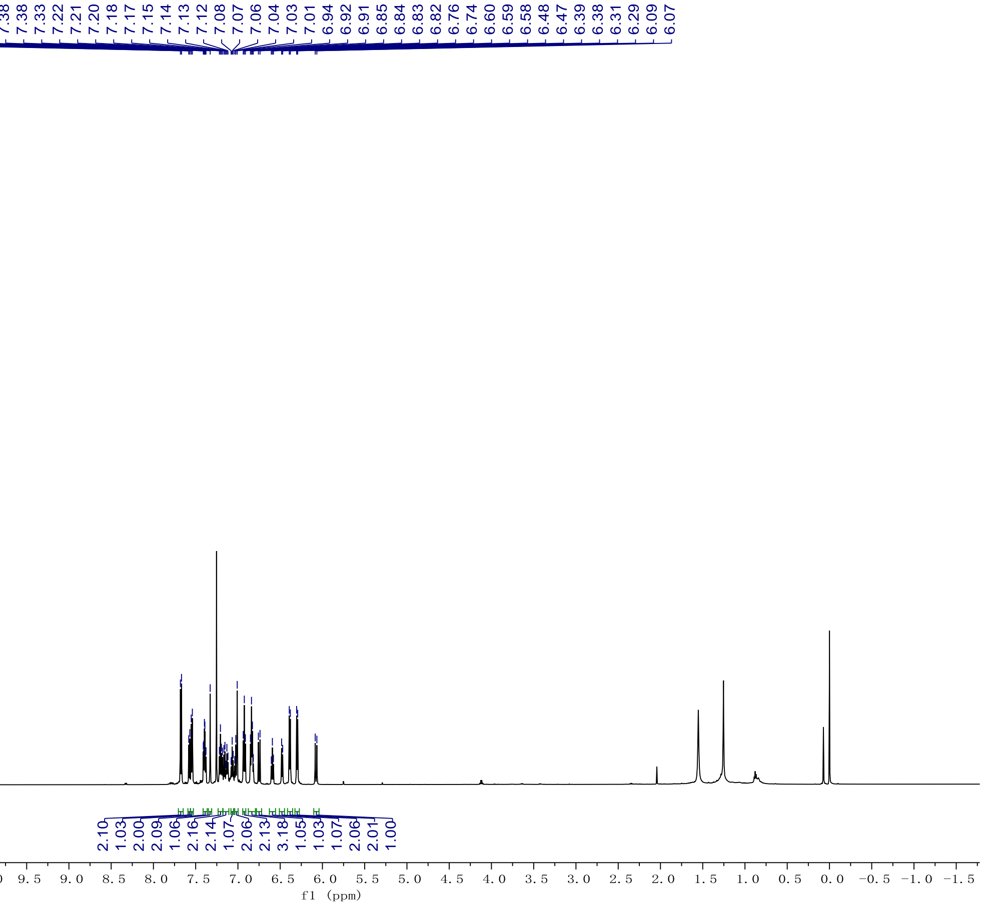

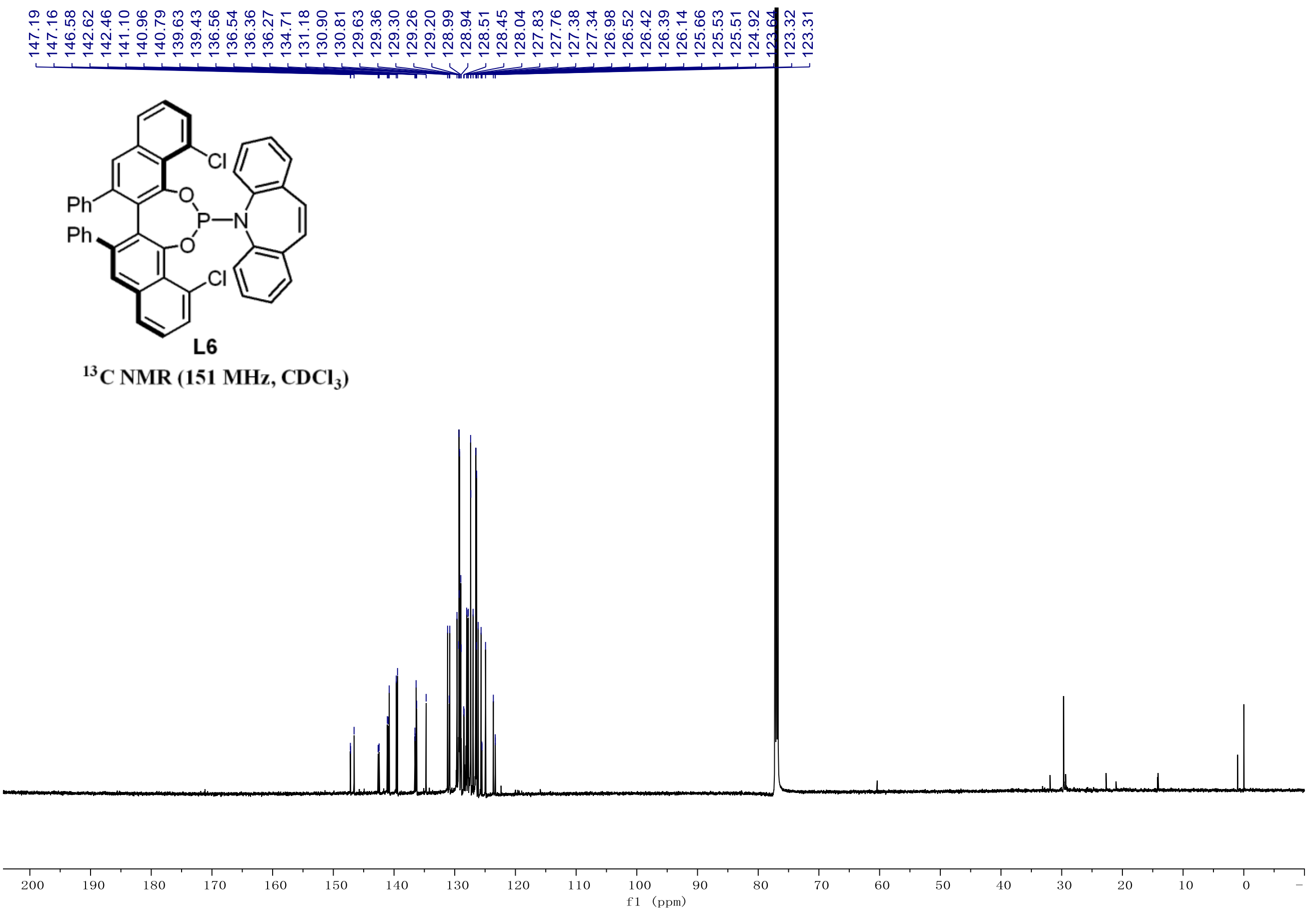
147.19
147.16
146.58
142.62
142.46
141.10
140.96
140.79
139.63
139.43
136.56
136.54
136.36
136.27
134.71
131.18
130.90
130.81
129.63
129.36
129.30
129.26
129.20
128.99
128.94
128.51
128.45
128.04
127.83
127.76
127.38
127.34
126.98
126.52
126.42
126.39
126.14
125.66
125.53
125.51
124.92
123.64
123.32
123.31
Ph
Ph
O
P
O
N
Cl
Cl
L6
^{13}C NMR (151 MHz, CDCl$_3$)
200 190 180 170 160 150 140 130 120 110 100 90 80 70 60 50 40 30 20 10 0
f1 (ppm)

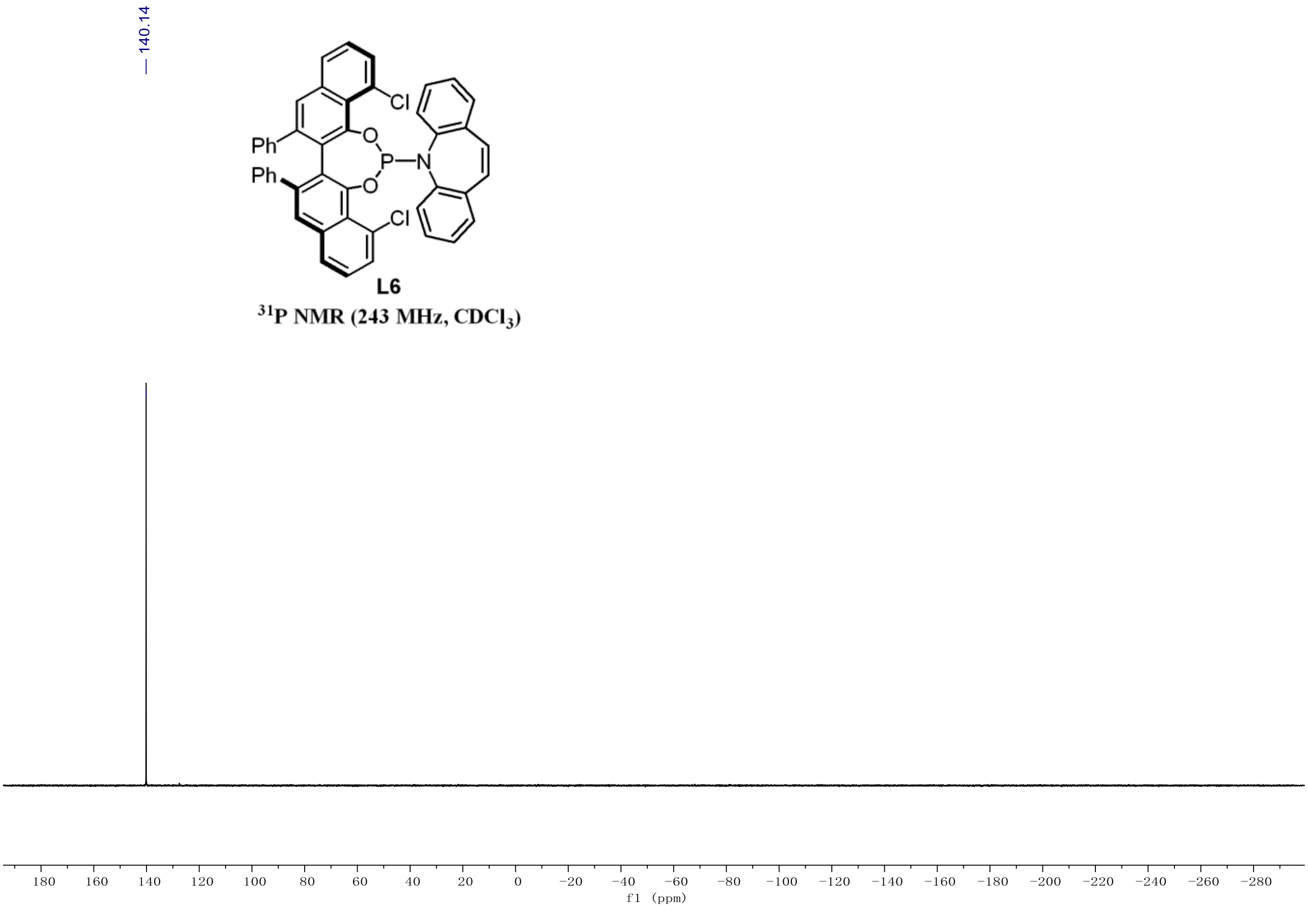
140.14
Ph
Ph
O
P
O
N
Cl
Cl
L6
^{31}P NMR (243 MHz, CDCl$_3$)
180 160 140 120 100 80 60 40 20 0 −20 −40 −60 −80 −100 −120 −140 −160 −180 −200 −220 −240 −260 −280
f1 (ppm)

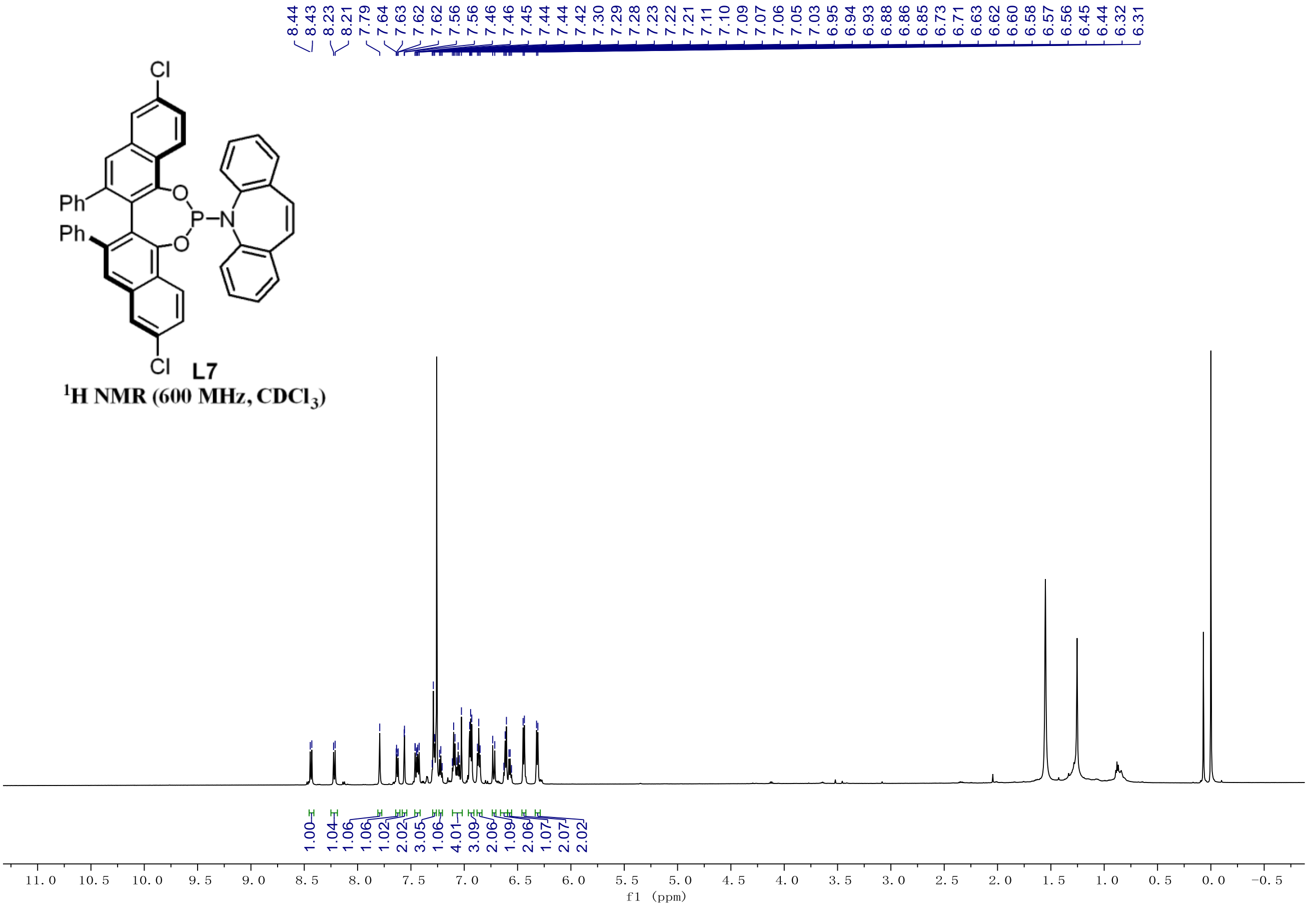
8.44
8.43
8.23
8.21
7.79
7.64
7.63
7.62
7.62
7.56
7.56
7.46
7.45
7.44
7.42
7.30
7.29
7.28
7.23
7.22
7.21
7.11
7.10
7.09
7.07
7.06
7.05
7.03
6.95
6.94
6.93
6.88
6.86
6.85
6.73
6.71
6.63
6.62
6.60
6.58
6.57
6.56
6.45
6.44
6.32
6.31
Cl
Ph
Ph
O
P
N
O
Cl
L7
^{1}H NMR (600 MHz, $CDCl_3$)
1.00
1.04
1.06
1.06
1.02
2.02
3.05
1.06
4.01
3.09
2.06
1.09
2.06
1.07
2.07
2.02
11.0 10.5 10.0 9.5 9.0 8.5 8.0 7.5 7.0 6.5 6.0 5.5 5.0 4.5 4.0 3.5 3.0 2.5 2.0 1.5 1.0 0.5 0.0 -0.5
f1 (ppm)

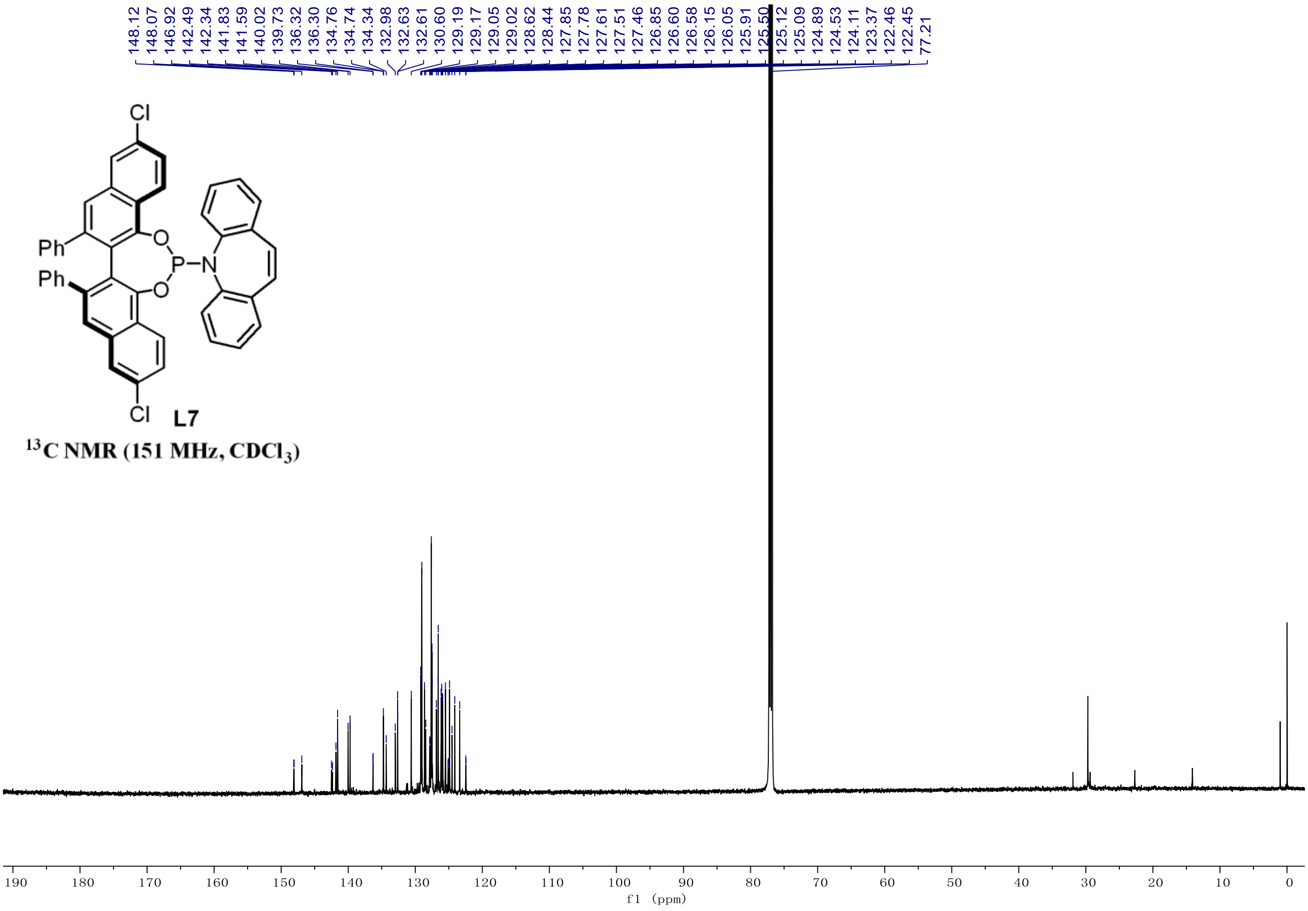
148.12
148.07
146.92
142.49
142.34
141.83
141.59
140.02
139.73
136.32
136.30
134.76
134.74
134.34
132.98
132.63
132.61
130.60
129.19
129.17
129.05
129.02
128.62
128.44
127.85
127.78
127.61
127.51
127.46
126.85
126.60
126.58
126.15
126.05
125.91
125.50
125.12
125.09
124.89
124.53
124.11
123.37
122.46
122.45
77.21
Cl
Ph
Ph
O
P
N
O
Cl
L7
^{13}C NMR (151 MHz, $CDCl_3$)
190 180 170 160 150 140 130 120 110 100 90 80 70 60 50 40 30 20 10 0
f1 (ppm)

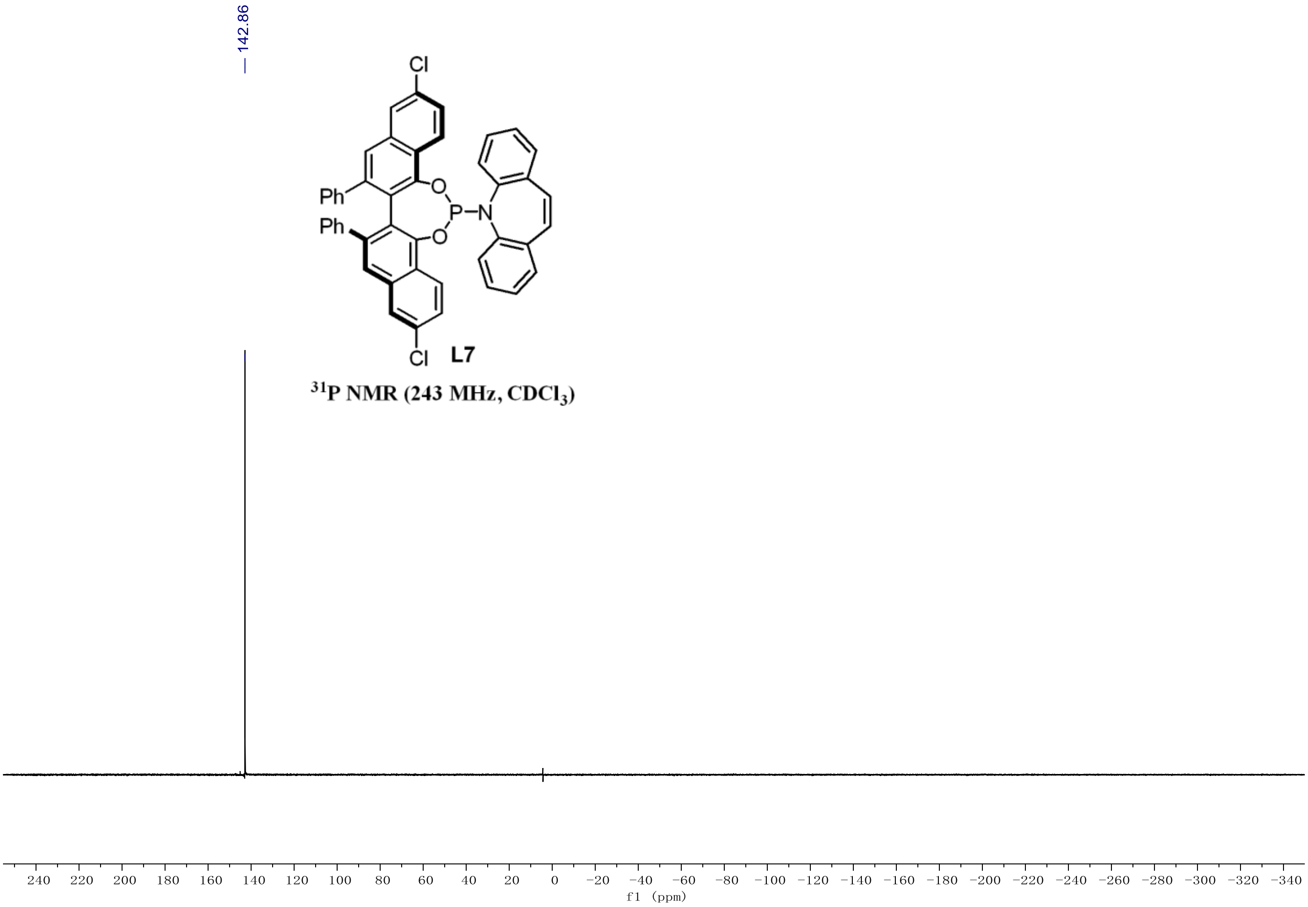

142.86
Cl
Ph
Ph
O
P
N
O
Cl
L7
31P NMR (243 MHz, CDCl3)
240 220 200 180 160 140 120 100 80 60 40 20 0 −20 −40 −60 −80 −100 −120 −140 −160 −180 −200 −220 −240 −260 −280 −300 −320 −340
f1 (ppm)

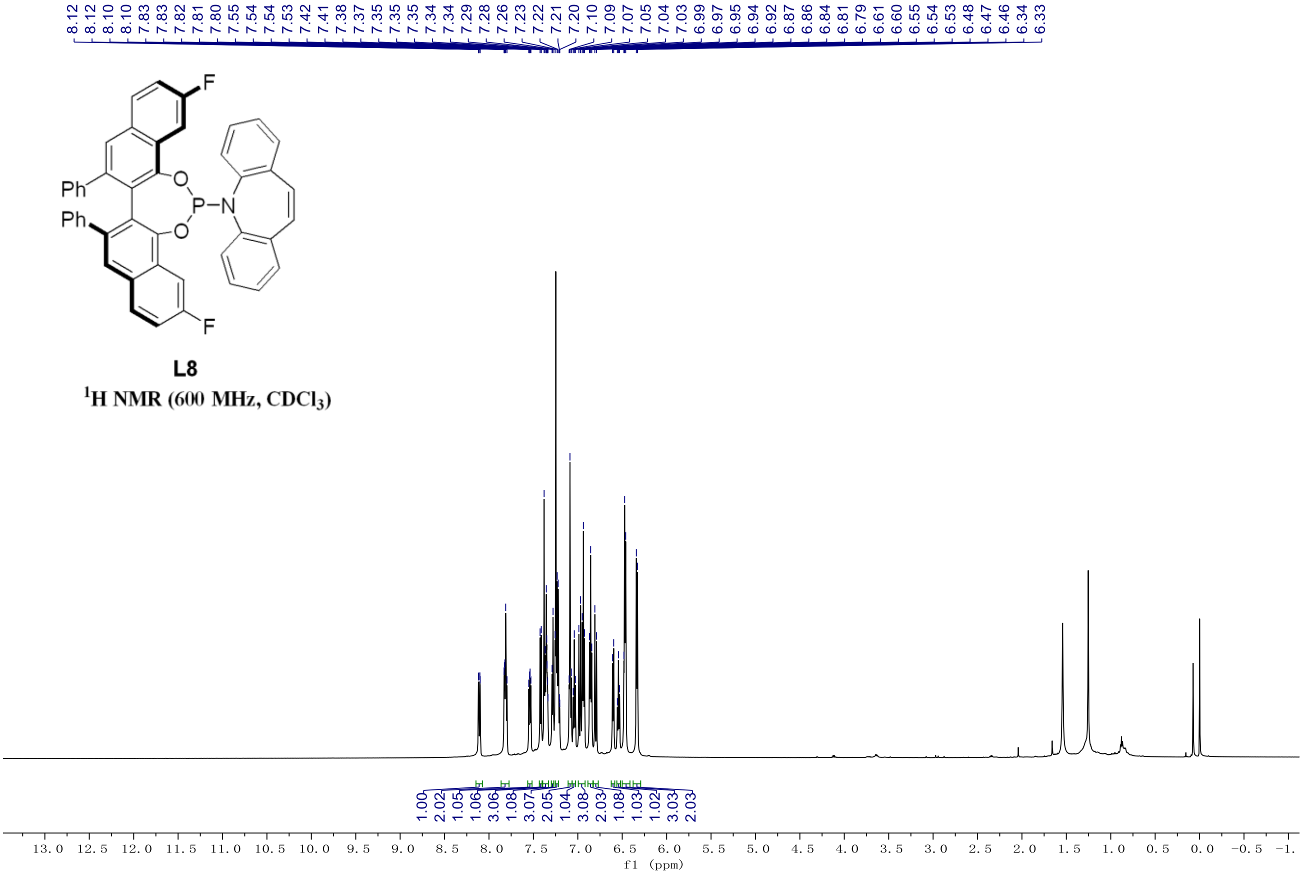

8.12 8.12 8.10 7.83 7.83 7.82 7.81 7.80 7.55 7.54 7.54 7.53 7.42 7.41 7.38 7.37 7.35 7.35 7.35 7.34 7.29 7.28 7.26 7.23 7.22 7.21 7.20 7.10 7.09 7.07 7.05 7.04 7.03 6.99 6.97 6.95 6.94 6.92 6.87 6.86 6.84 6.81 6.79 6.61 6.60 6.55 6.54 6.53 6.48 6.47 6.46 6.34 6.33
F
Ph
Ph
O
P
N
O
F
L8
1H NMR (600 MHz, CDCl3)
1.00 2.02 1.05 1.06 3.06 1.08 3.07 2.05 1.04 3.08 2.03 1.08 1.03 1.02 3.03 2.03
13.0 12.5 12.0 11.5 11.0 10.5 10.0 9.5 9.0 8.5 8.0 7.5 7.0 6.5 6.0 5.5 5.0 4.5 4.0 3.5 3.0 2.5 2.0 1.5 1.0 0.5 0.0 −0.5 −1.
f1 (ppm)

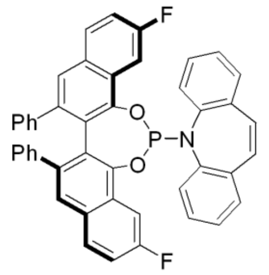

L8

^{13}C NMR (151 MHz, CDCl$_3$)

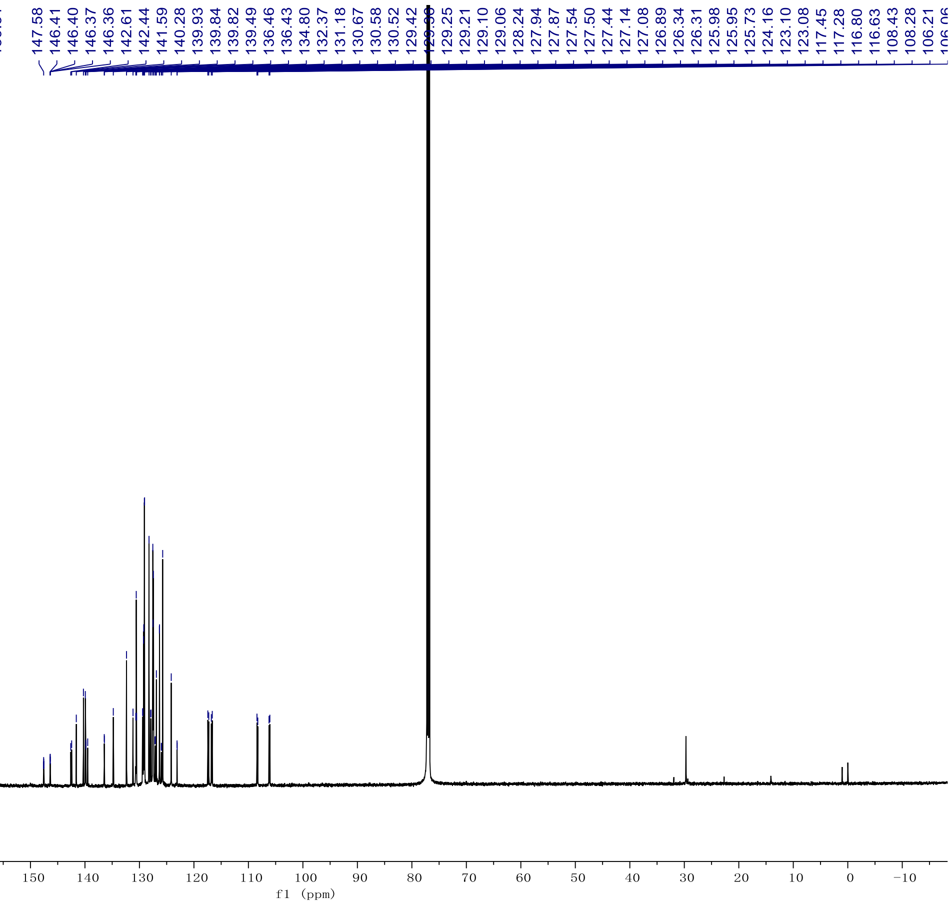

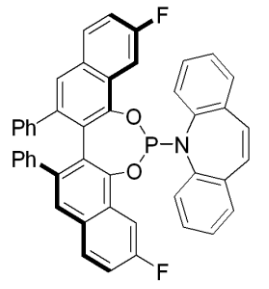

L8

^{31}P NMR (243 MHz, CDCl$_3$)

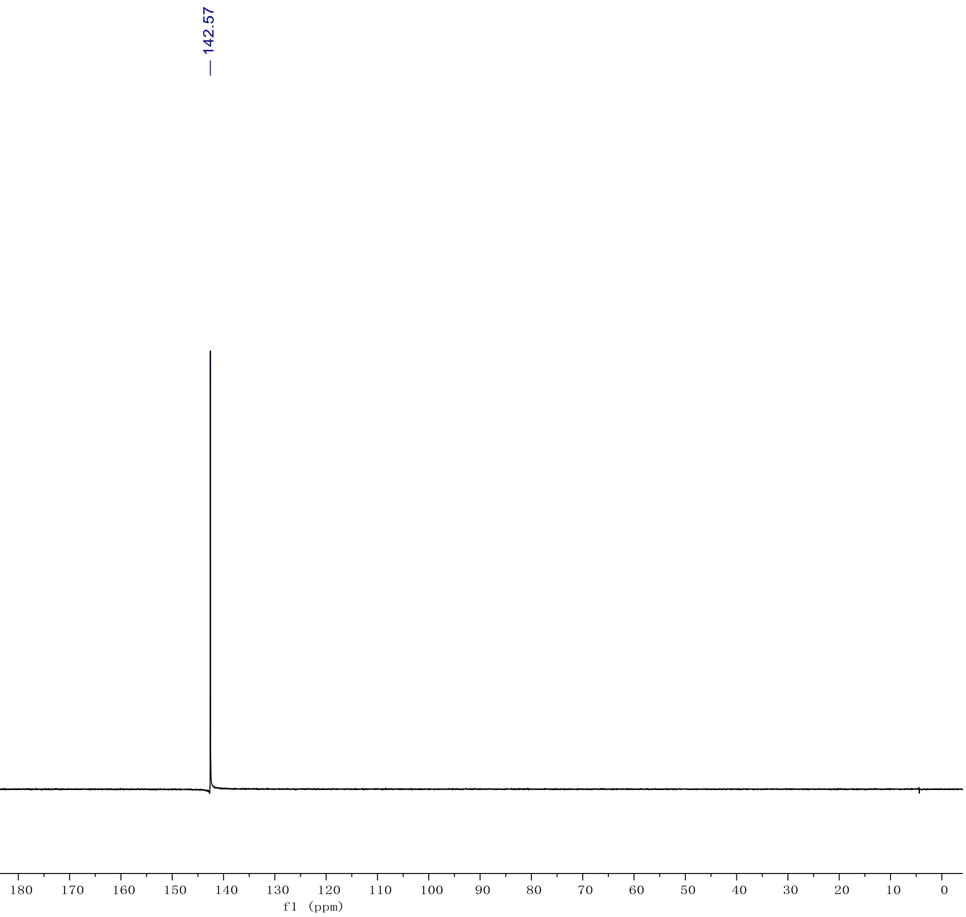

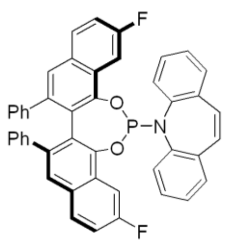

L8

^{19}F NMR (565 MHz, $CDCl_3$)

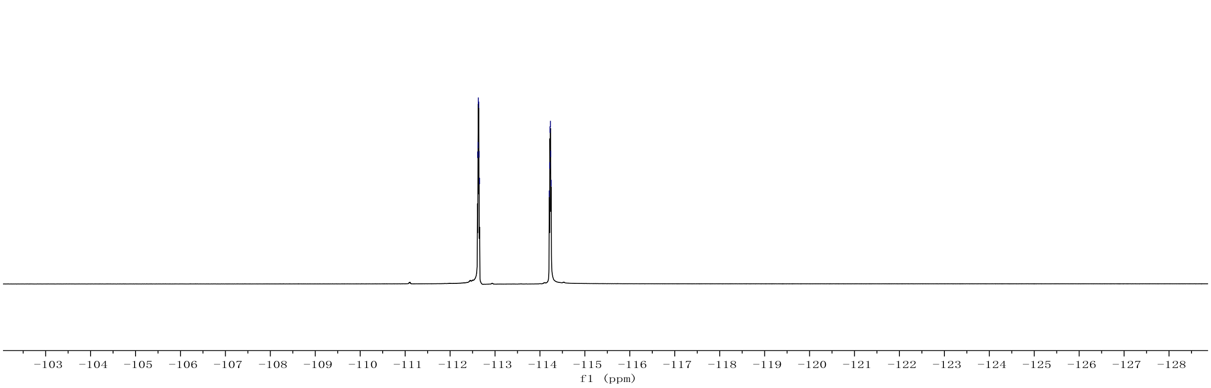

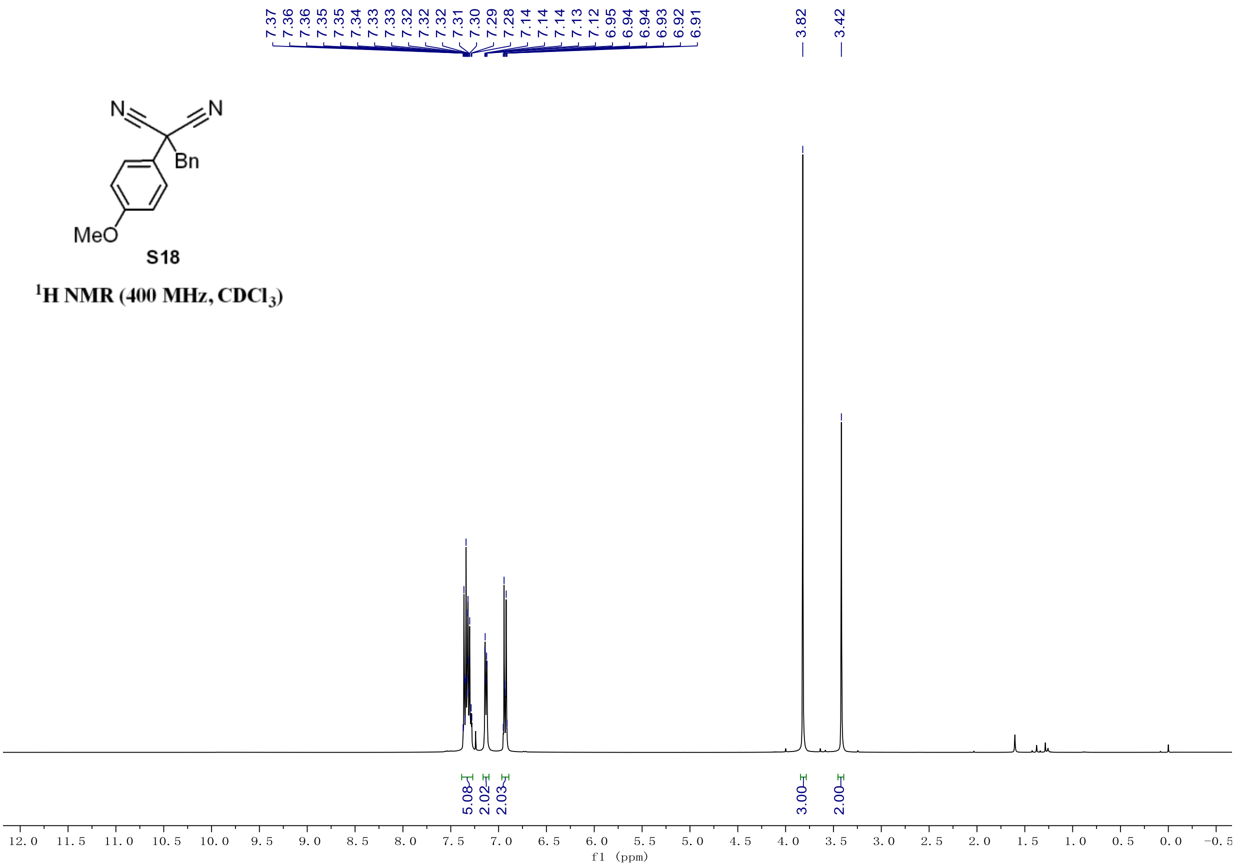

S18

^{1}H NMR (400 MHz, CDCl$_3$)

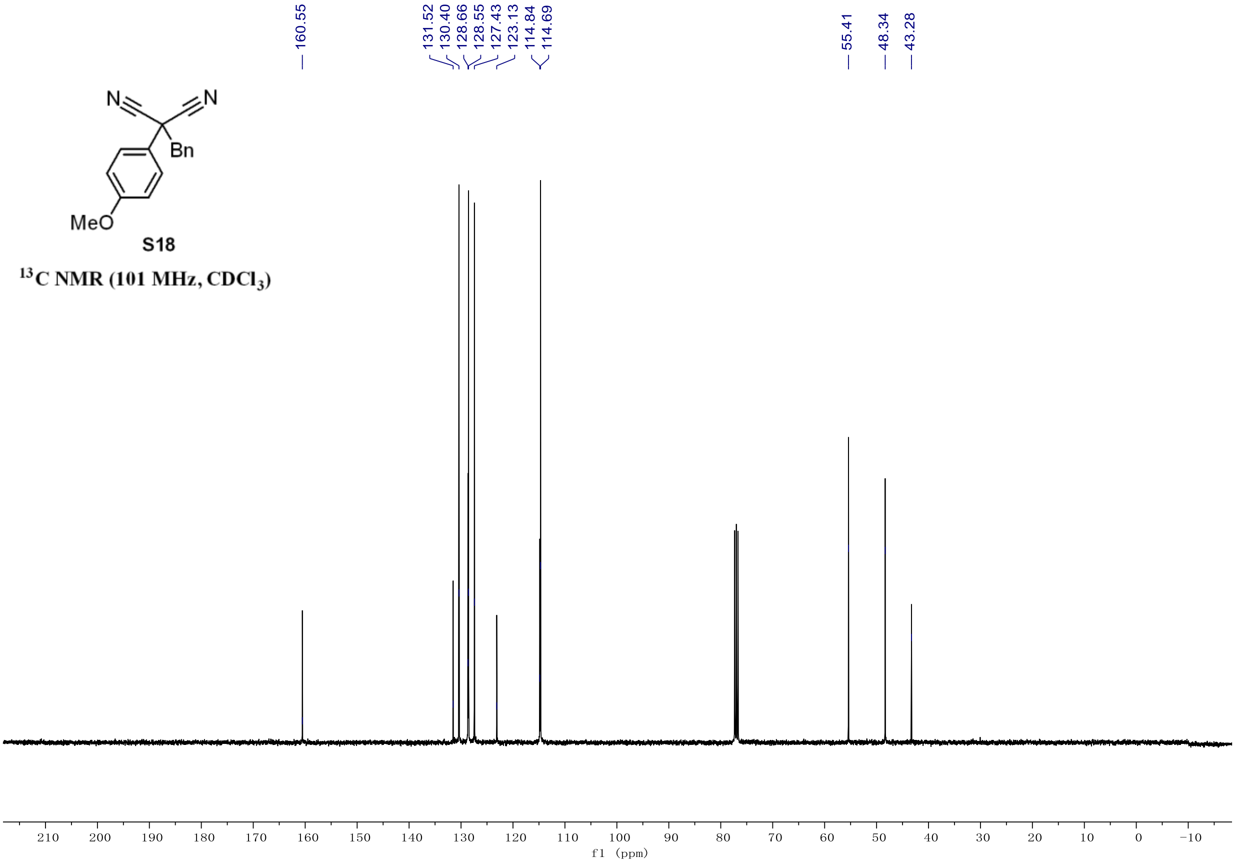

S18

^{13}C NMR (101 MHz, CDCl$_3$)

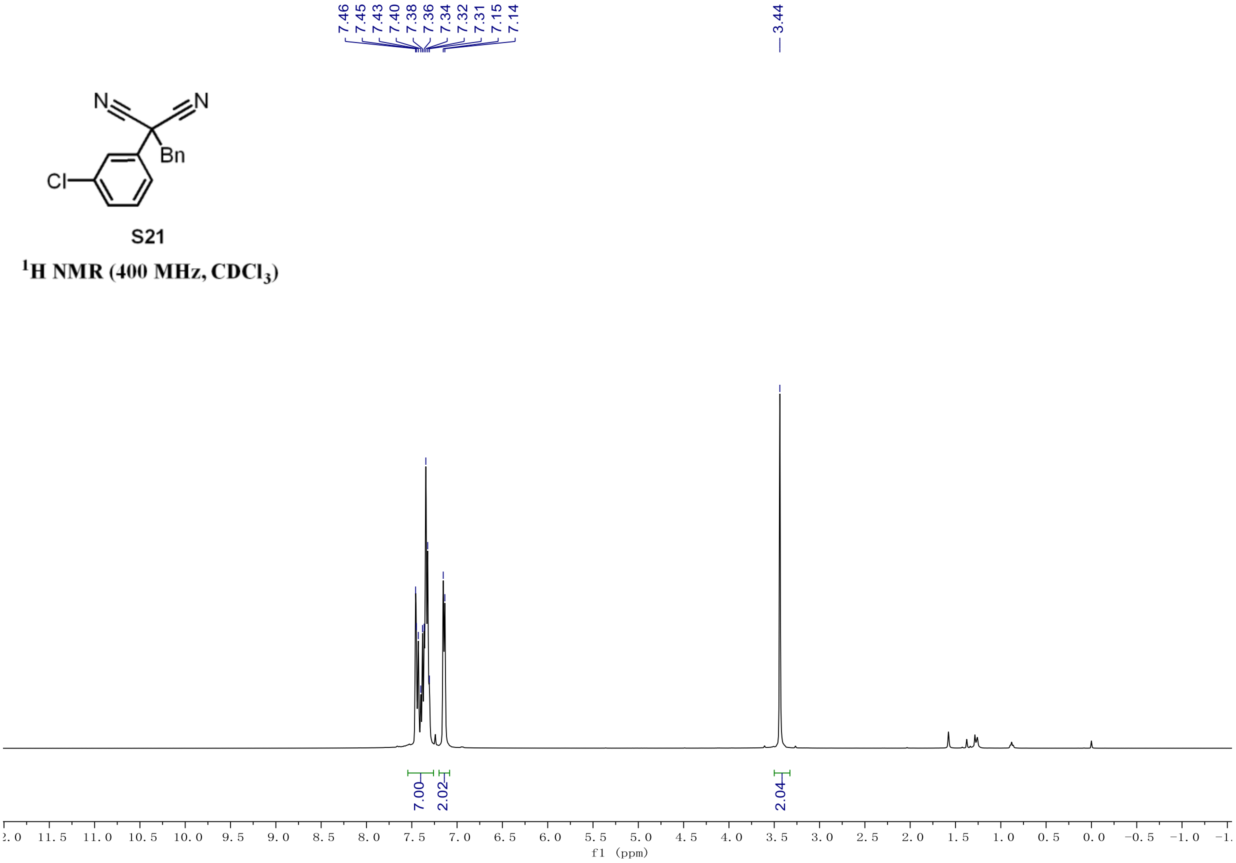

7.46
7.45
7.43
7.40
7.38
7.36
7.34
7.32
7.31
7.15
7.14
3.44
Bn
Cl
S21
^{1}H NMR (400 MHz, $CDCl_3$)
7.00
2.02
2.04
f1 (ppm)

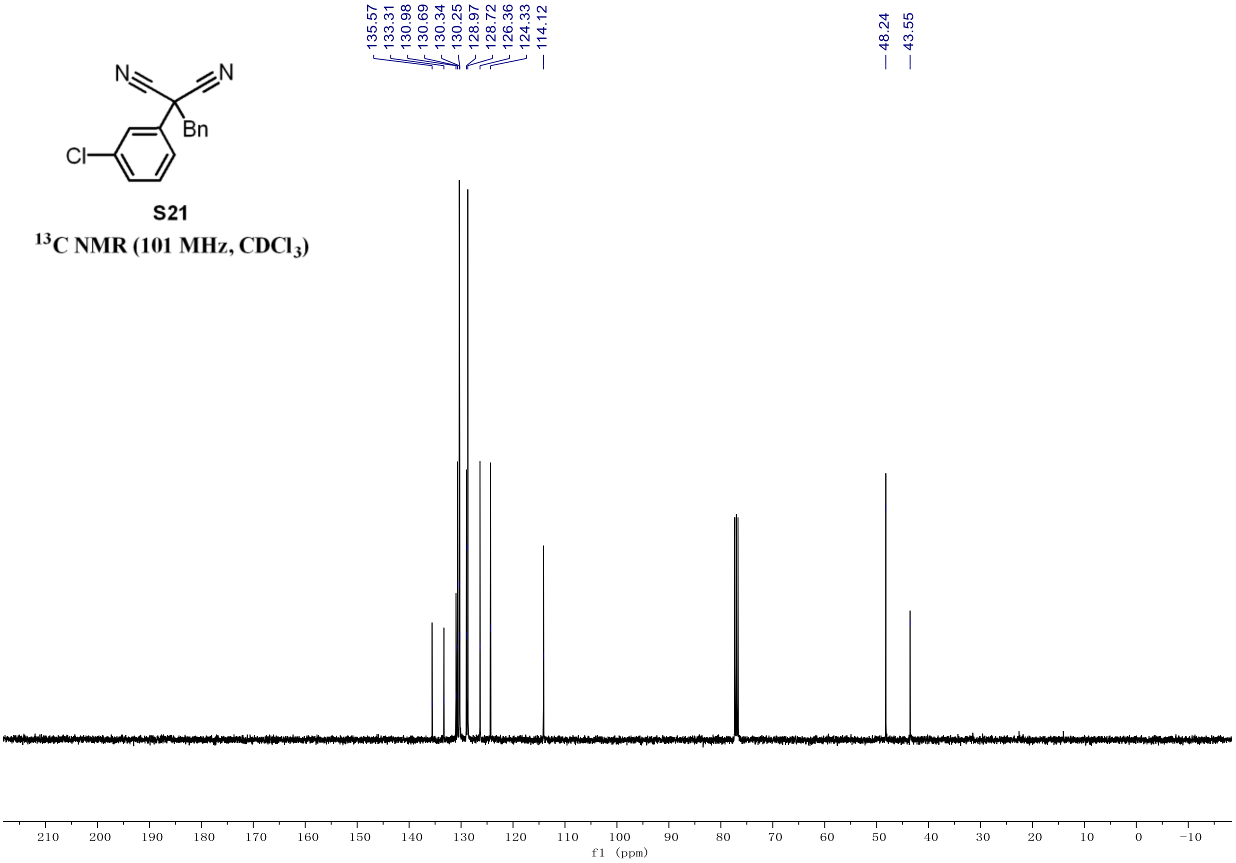

135.57
133.31
130.98
130.69
130.34
130.25
128.97
128.72
126.36
124.33
114.12
48.24
43.55
Bn
Cl
S21
^{13}C NMR (101 MHz, $CDCl_3$)
f1 (ppm)

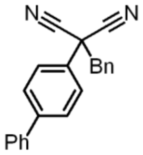

S23

^{1}H NMR (400 MHz, CDCl$_3$)

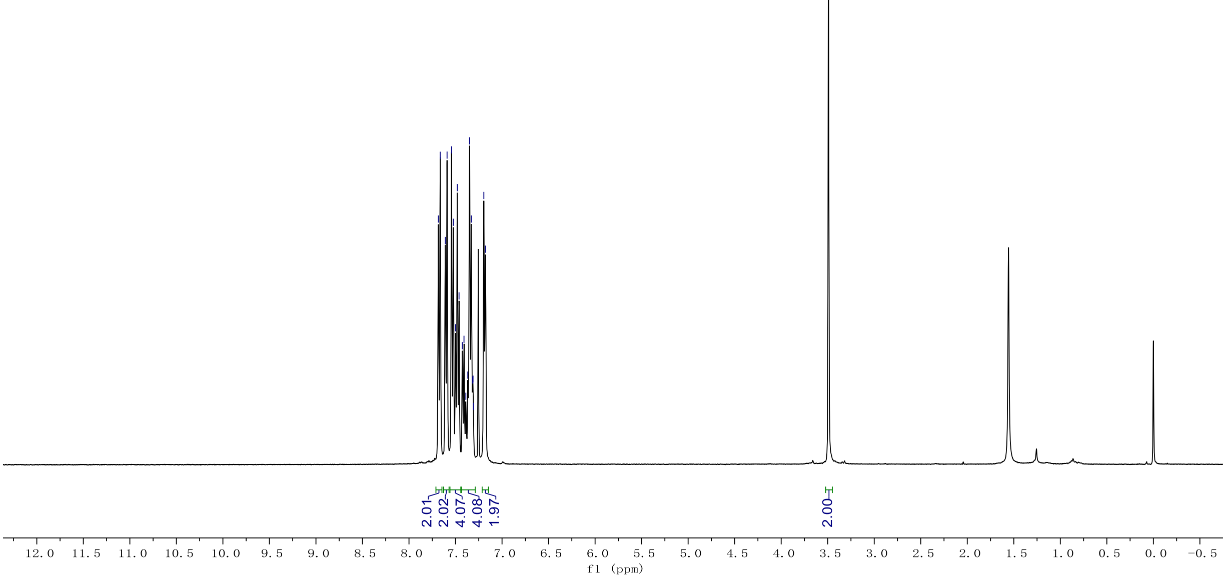

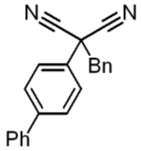

S23

^{13}C NMR (101 MHz, CDCl$_3$)

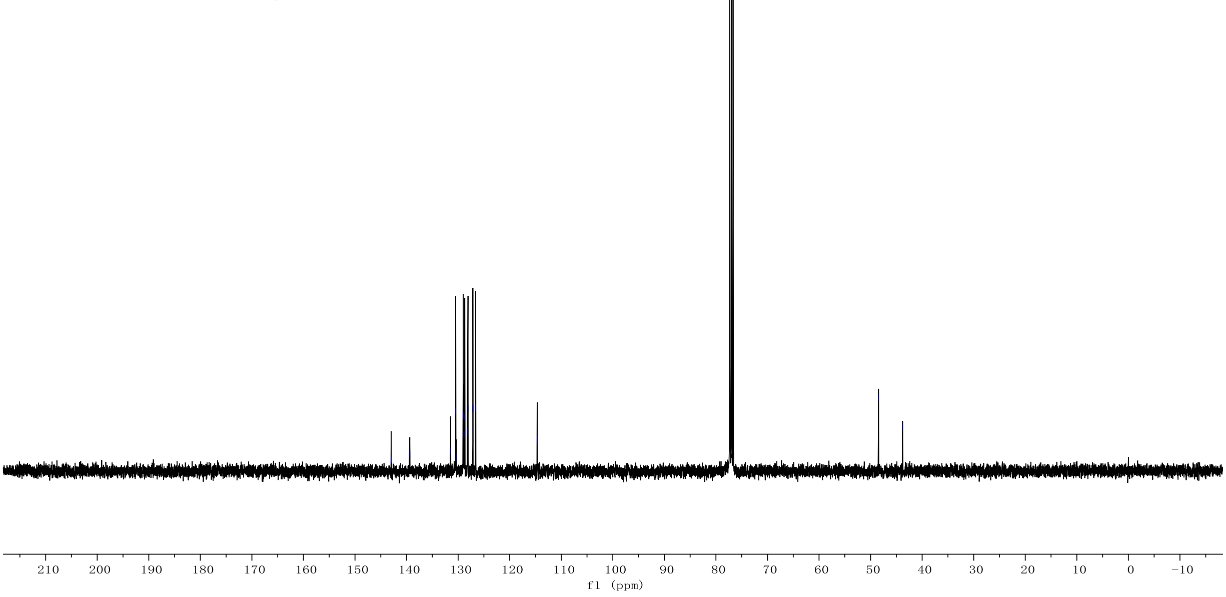

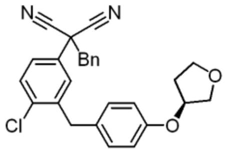

S29

^{1}H NMR (400 MHz, $CDCl_3$)

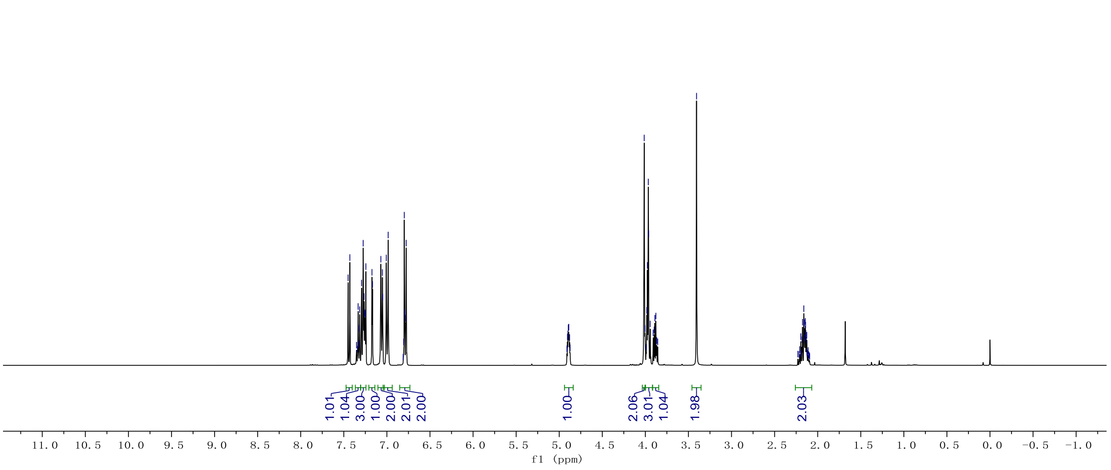

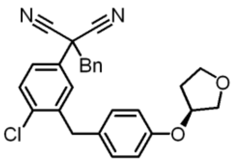

S29

^{13}C NMR (101 MHz, $CDCl_3$)

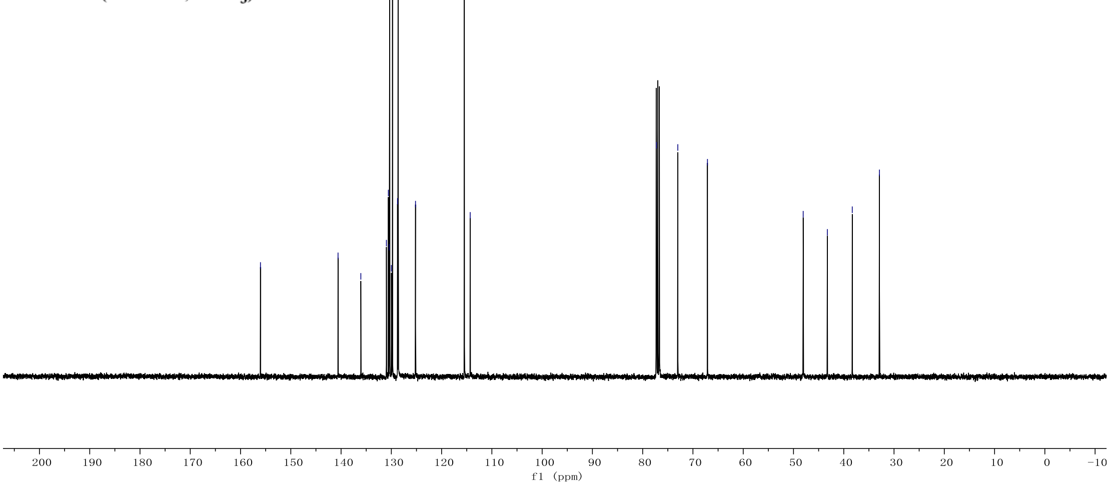

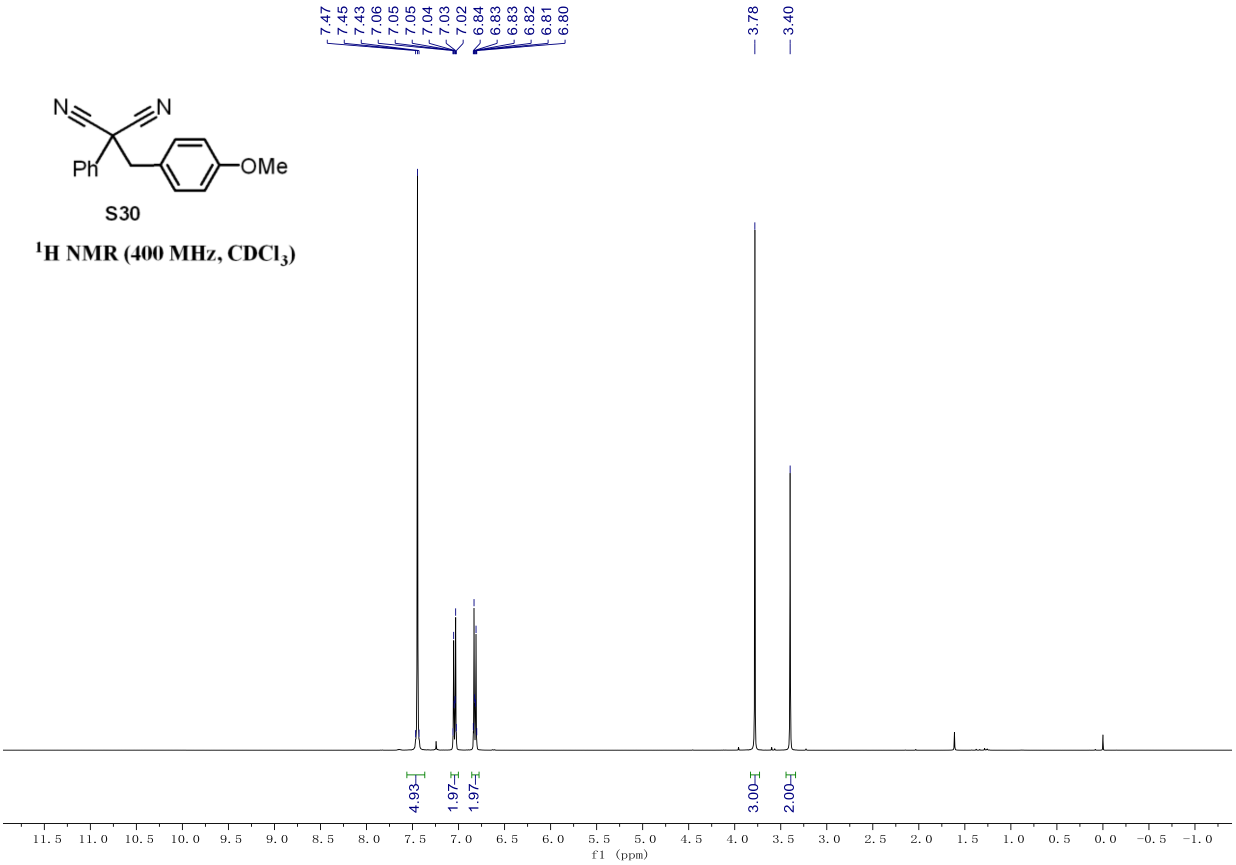

7.47
7.45
7.43
7.06
7.05
7.05
7.04
7.03
7.02
6.84
6.83
6.83
6.82
6.81
6.80
3.78
3.40
N
N
Ph
OMe
S30
1H NMR (400 MHz, CDCl3)
4.93
1.97
1.97
3.00
2.00
11.5 11.0 10.5 10.0 9.5 9.0 8.5 8.0 7.5 7.0 6.5 6.0 5.5 5.0 4.5 4.0 3.5 3.0 2.5 2.0 1.5 1.0 0.5 0.0 -0.5 -1.0
f1 (ppm)

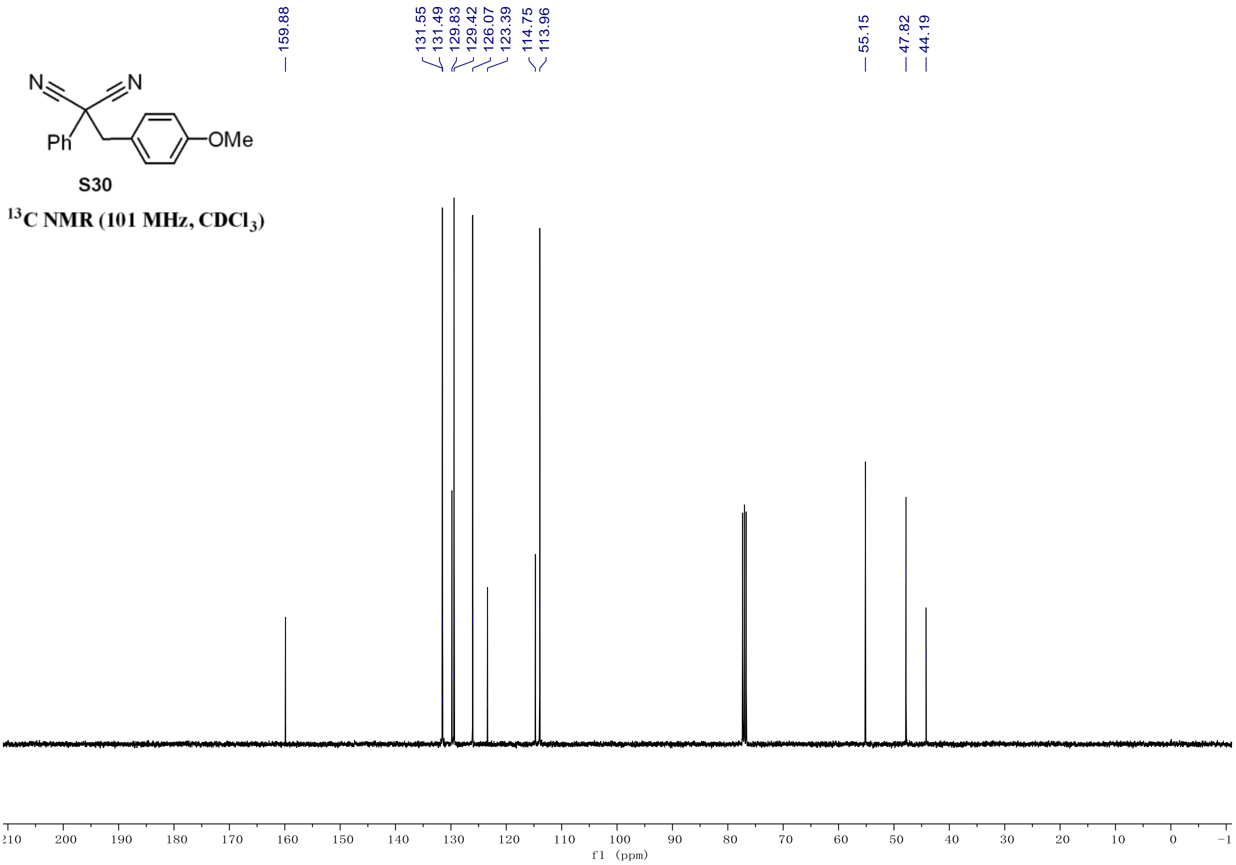

159.88
131.55
131.49
129.83
129.42
126.07
123.39
114.75
113.96
55.15
47.82
44.19
N
N
Ph
OMe
S30
13C NMR (101 MHz, CDCl3)
210 200 190 180 170 160 150 140 130 120 110 100 90 80 70 60 50 40 30 20 10 0 -1
f1 (ppm)

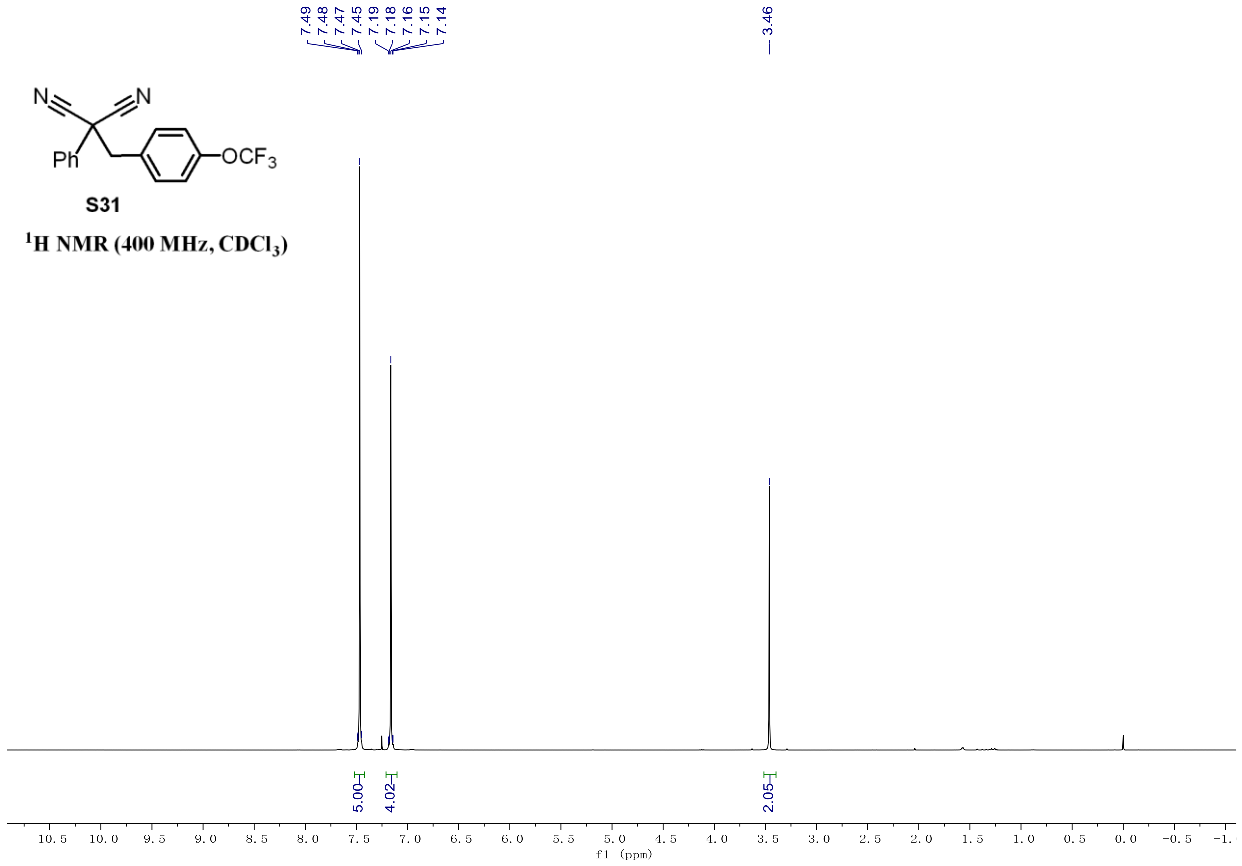
7.49
7.48
7.47
7.45
7.19
7.18
7.16
7.15
7.14
3.46
Ph
OCF3
S31
1H NMR (400 MHz, CDCl3)
5.00
4.02
2.05
10.5 10.0 9.5 9.0 8.5 8.0 7.5 7.0 6.5 6.0 5.5 5.0 4.5 4.0 3.5 3.0 2.5 2.0 1.5 1.0 0.5 0.0 -0.5 -1.
f1 (ppm)

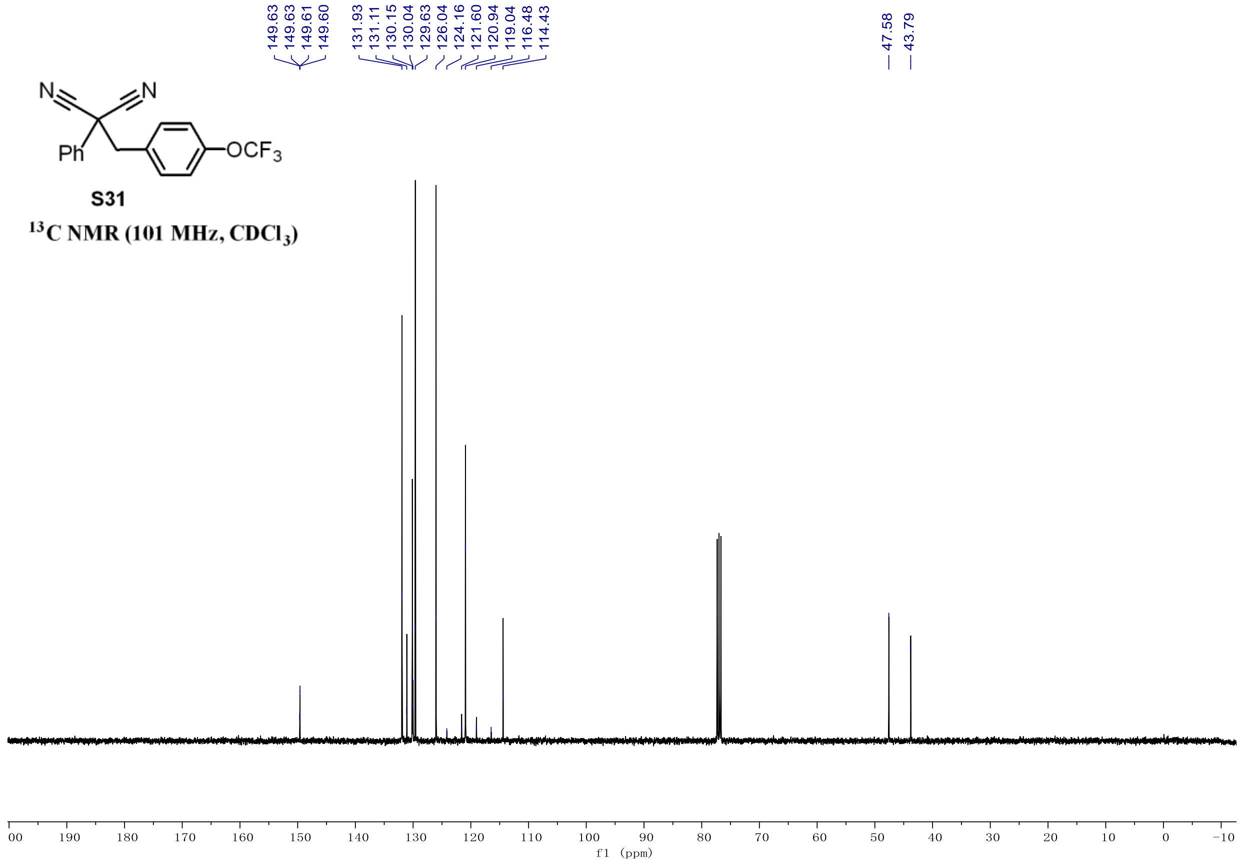
149.63
149.63
149.61
149.60
131.93
131.11
130.15
130.04
129.63
126.04
124.16
121.60
120.94
119.04
116.48
114.43
47.58
43.79
Ph
OCF3
S31
13C NMR (101 MHz, CDCl3)
00 190 180 170 160 150 140 130 120 110 100 90 80 70 60 50 40 30 20 10 0 -10
f1 (ppm)

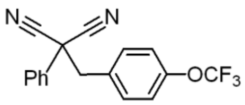

S31

^{19}**F NMR (376 MHz, CDCl$_3$)**

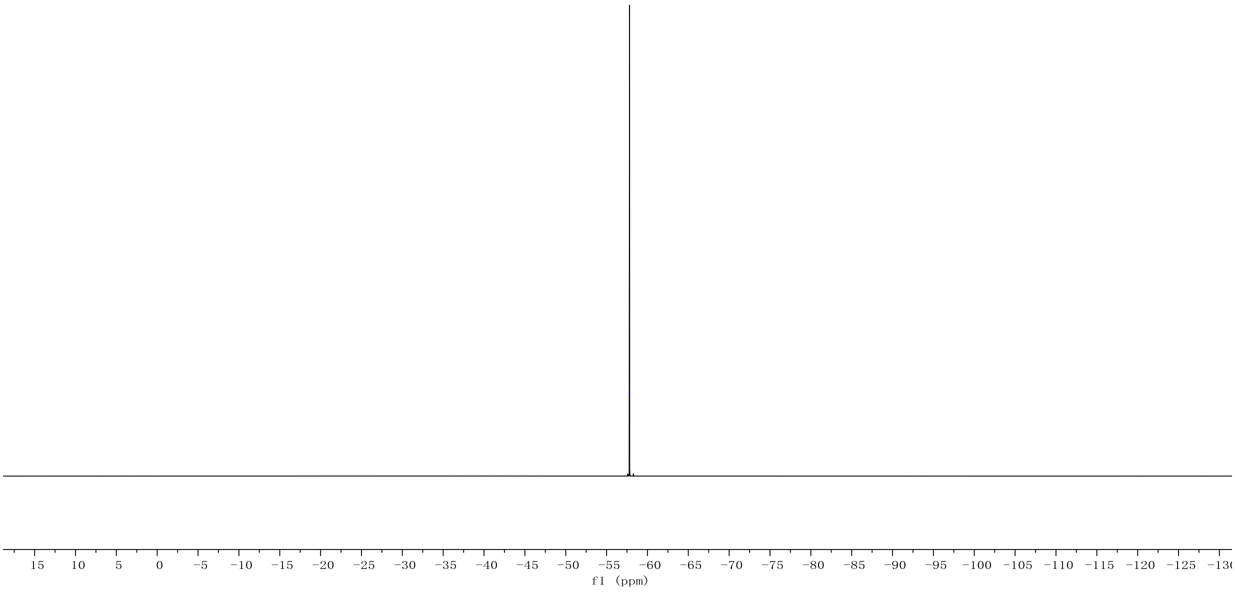

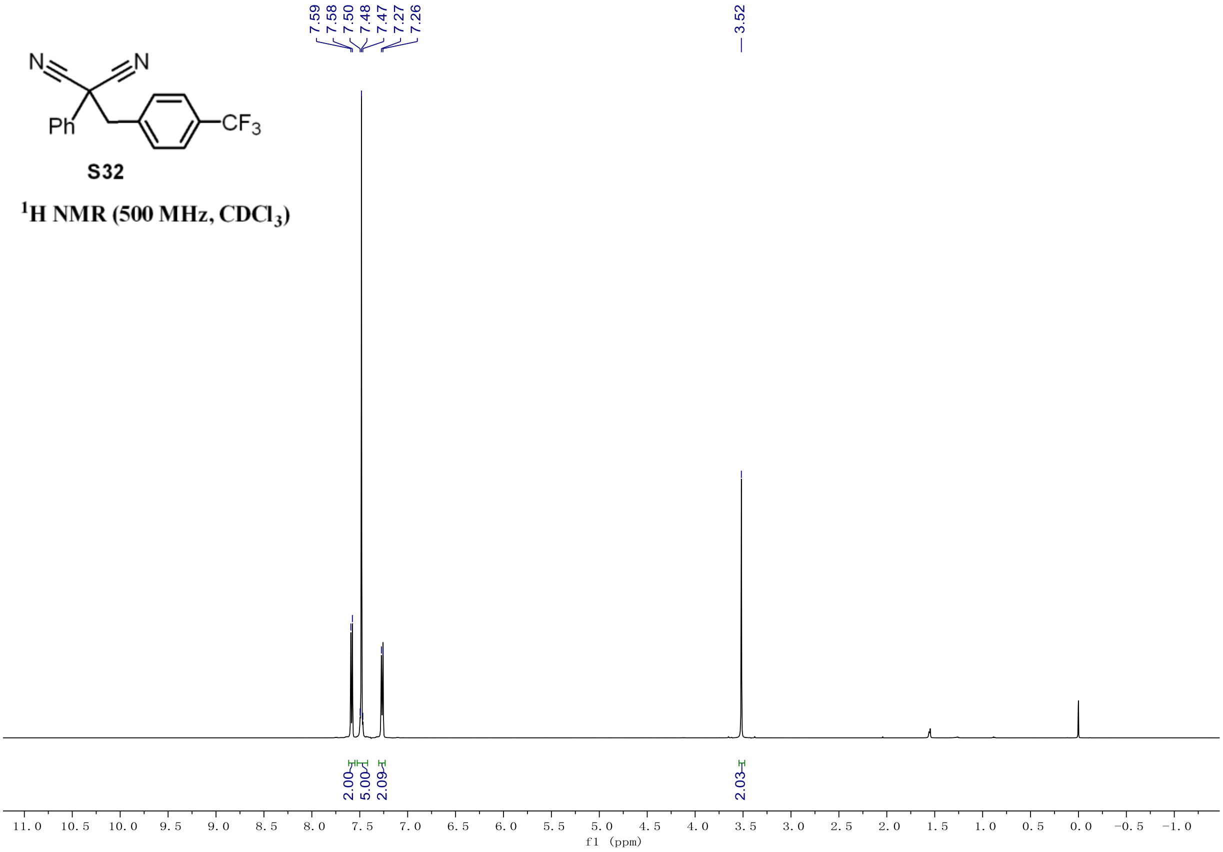
S32
^{1}H NMR (500 MHz, CDCl$_3$)
7.59
7.58
7.50
7.48
7.47
7.27
7.26
3.52
2.00
5.00
2.09
2.03
11.0 10.5 10.0 9.5 9.0 8.5 8.0 7.5 7.0 6.5 6.0 5.5 5.0 4.5 4.0 3.5 3.0 2.5 2.0 1.5 1.0 0.5 0.0 -0.5 -1.0
f1 (ppm)

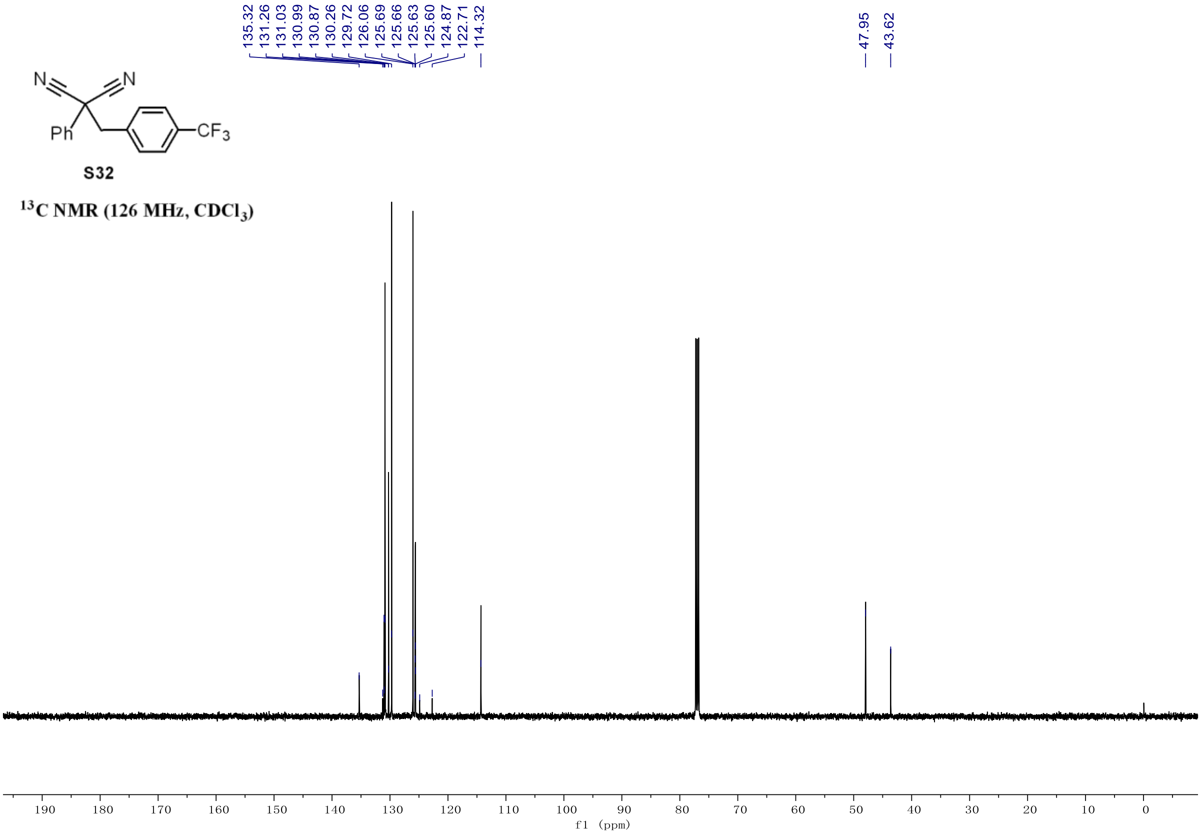
S32
^{13}C NMR (126 MHz, CDCl$_3$)
135.32
131.26
131.03
130.99
130.87
130.26
129.72
126.06
125.69
125.66
125.63
125.60
124.87
122.71
114.32
47.95
43.62
190 180 170 160 150 140 130 120 110 100 90 80 70 60 50 40 30 20 10 0
f1 (ppm)

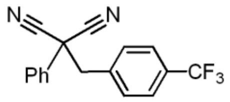

S32

^{19}F NMR (471 MHz, $CDCl_3$)

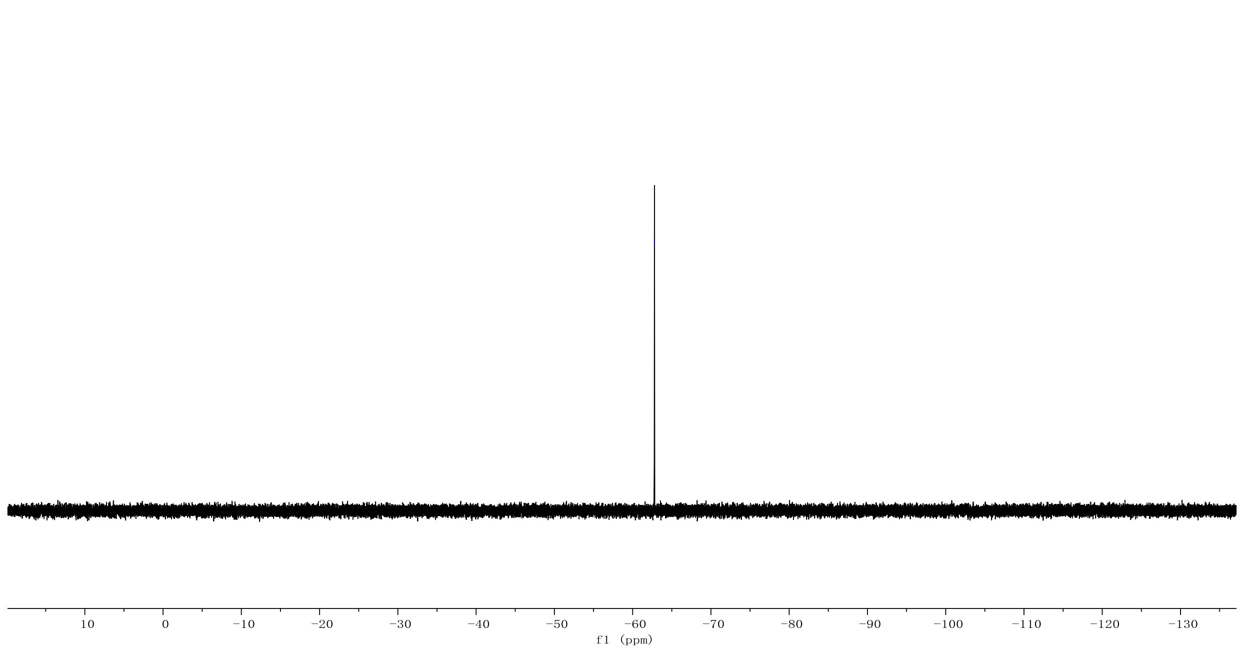

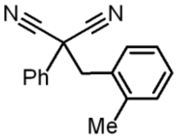

S35

^{1}H NMR (500 MHz, CDCl$_3$)

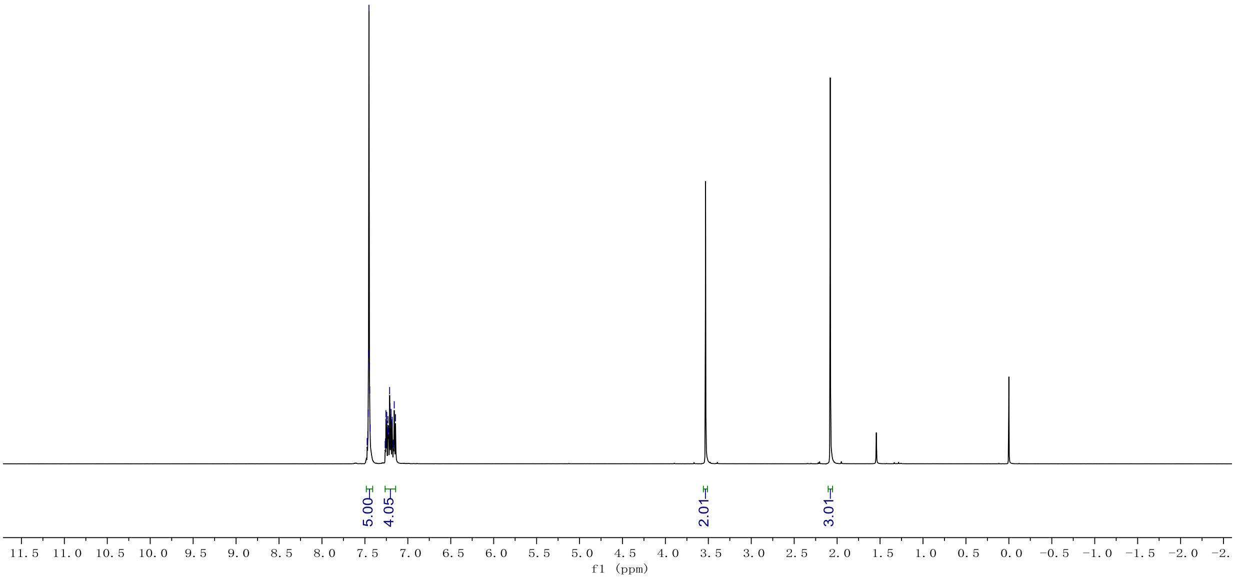

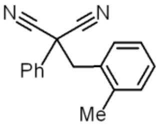

S35

^{13}C NMR (126 MHz, CDCl$_3$)

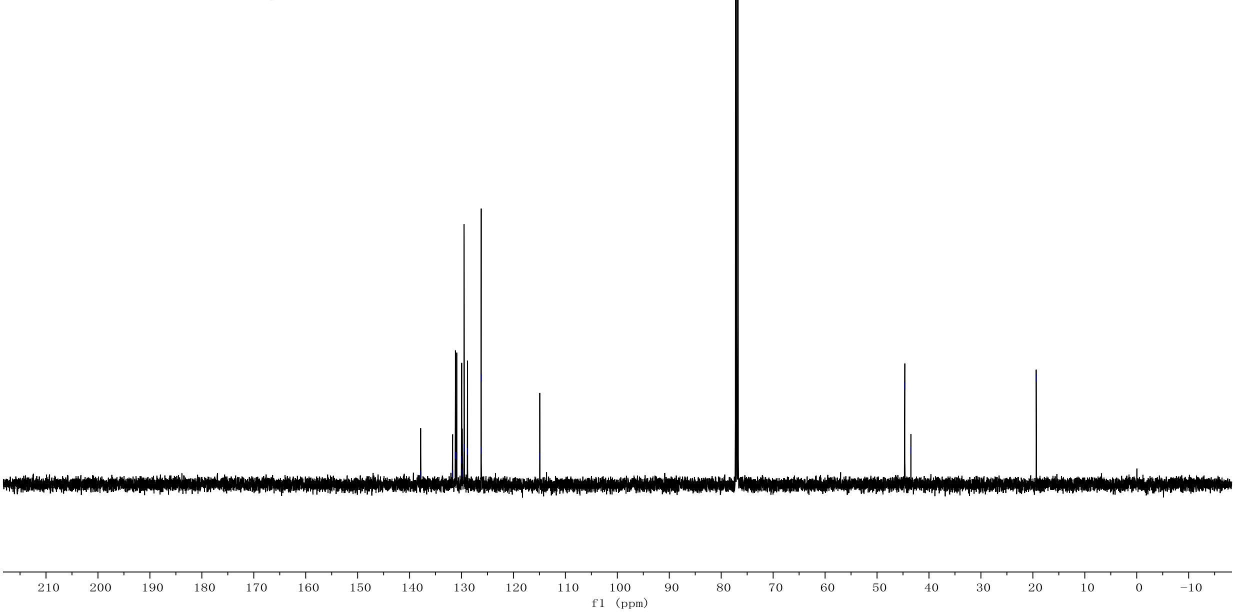

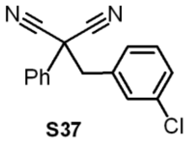

S37

^{1}**H NMR (400 MHz, CDCl$_3$)**

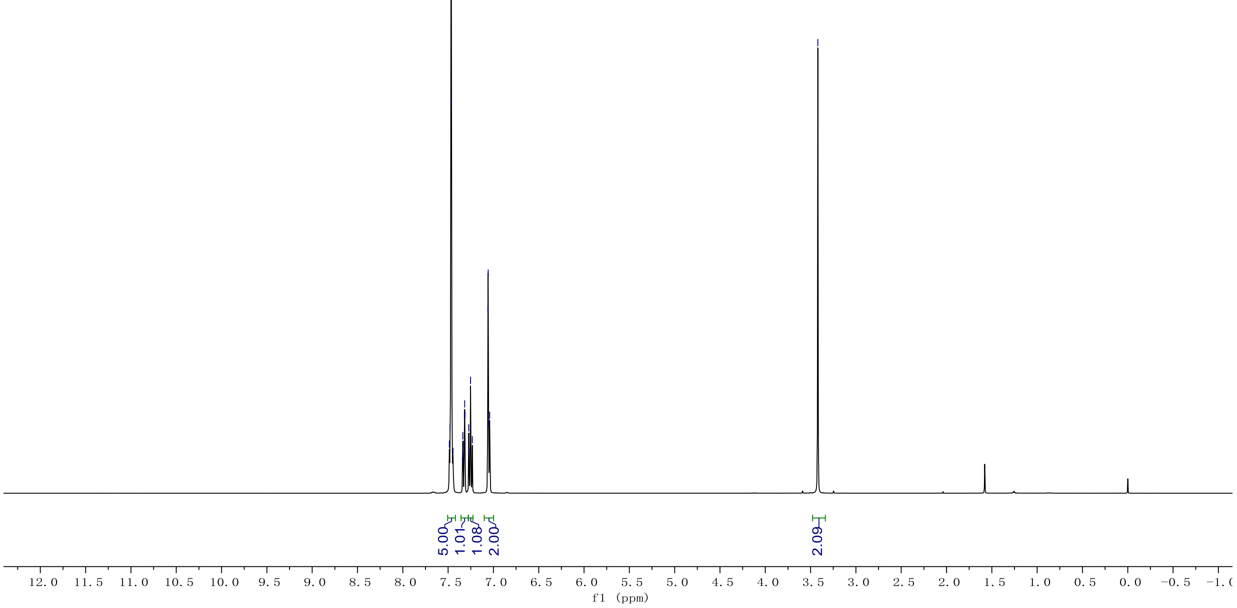

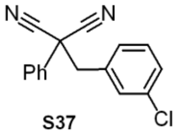

S37

^{13}**C NMR (101 MHz, CDCl$_3$)**

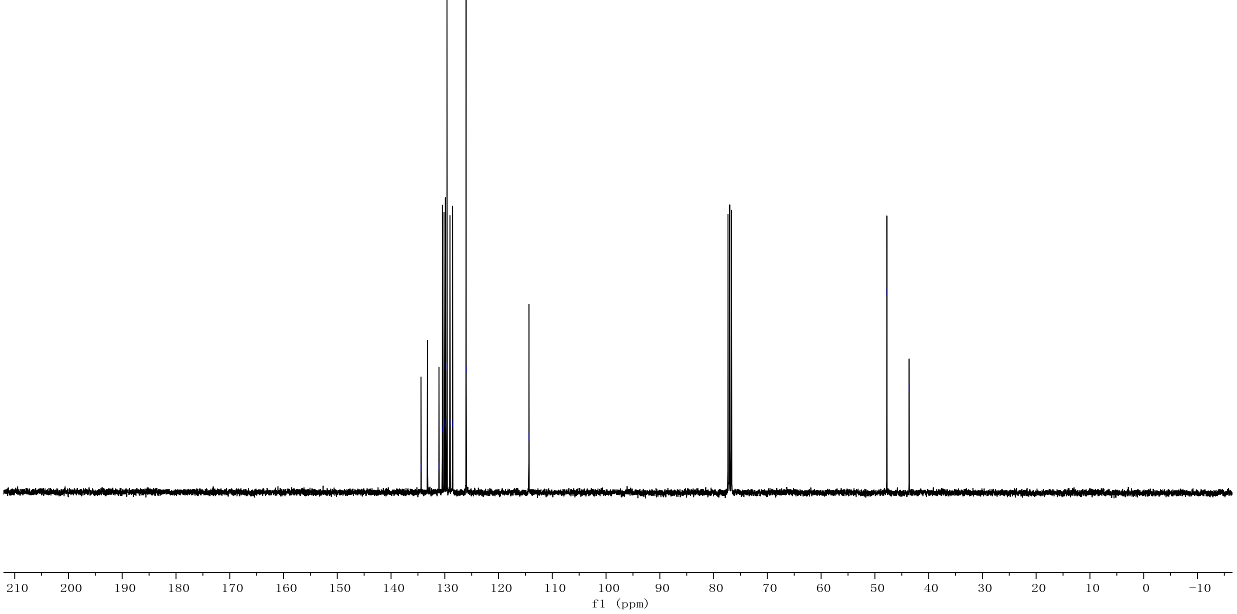

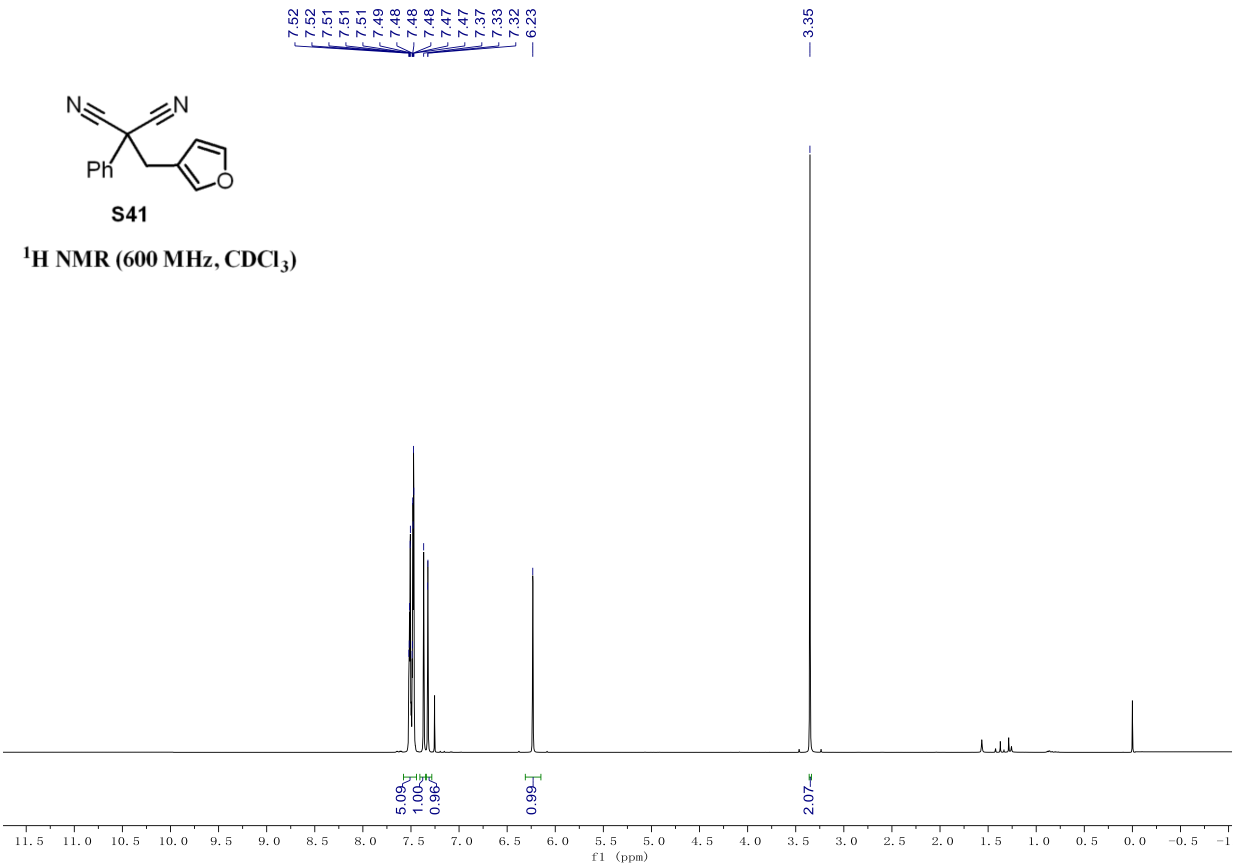
7.52
7.52
7.51
7.51
7.49
7.48
7.48
7.47
7.47
7.37
7.33
7.32
6.23
3.35
Ph
S41
N
N
O
1H NMR (600 MHz, CDCl3)
5.09
1.00
0.96
0.99
2.07
f1 (ppm)

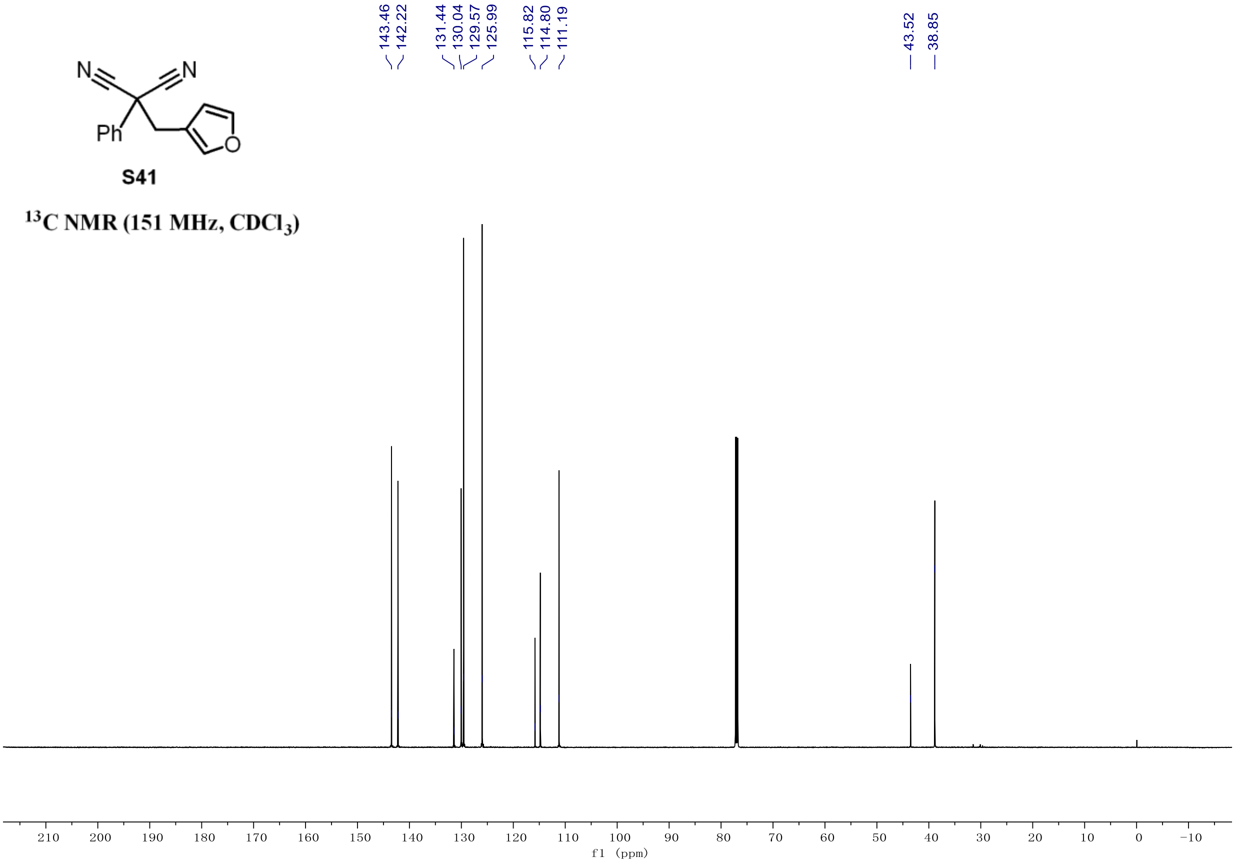
143.46
142.22
131.44
130.04
129.57
125.99
115.82
114.80
111.19
43.52
38.85
Ph
S41
N
N
O
13C NMR (151 MHz, CDCl3)
f1 (ppm)

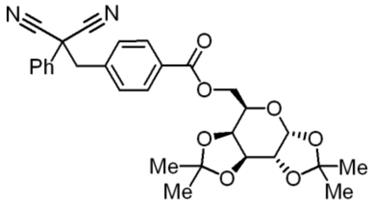

S42

^{1}H NMR (500 MHz, $CDCl_3$)

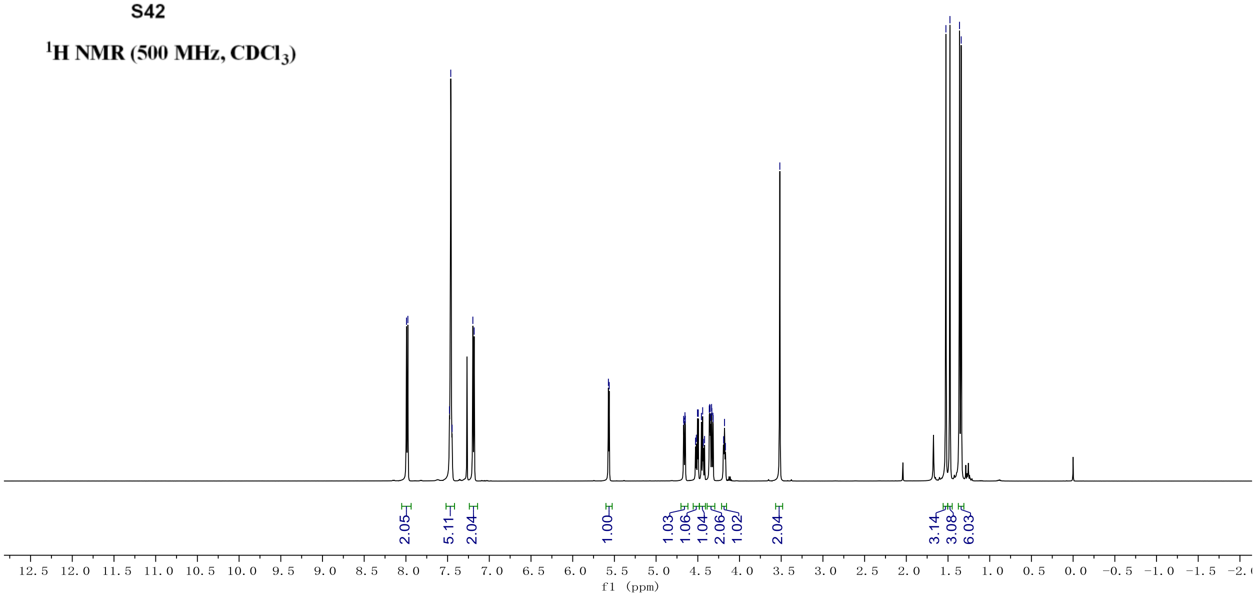

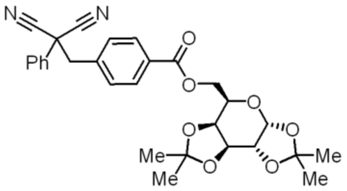

S42

^{13}C NMR (126 MHz, $CDCl_3$)

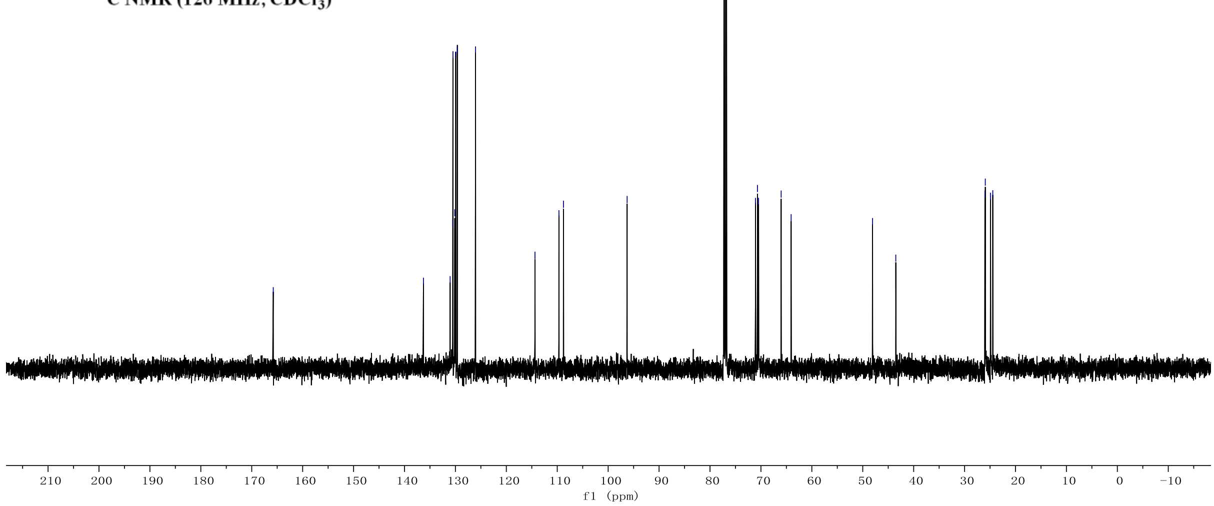

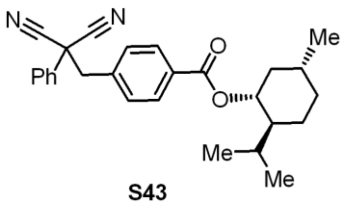

S43

^{1}H NMR (500 MHz, $CDCl_3$)

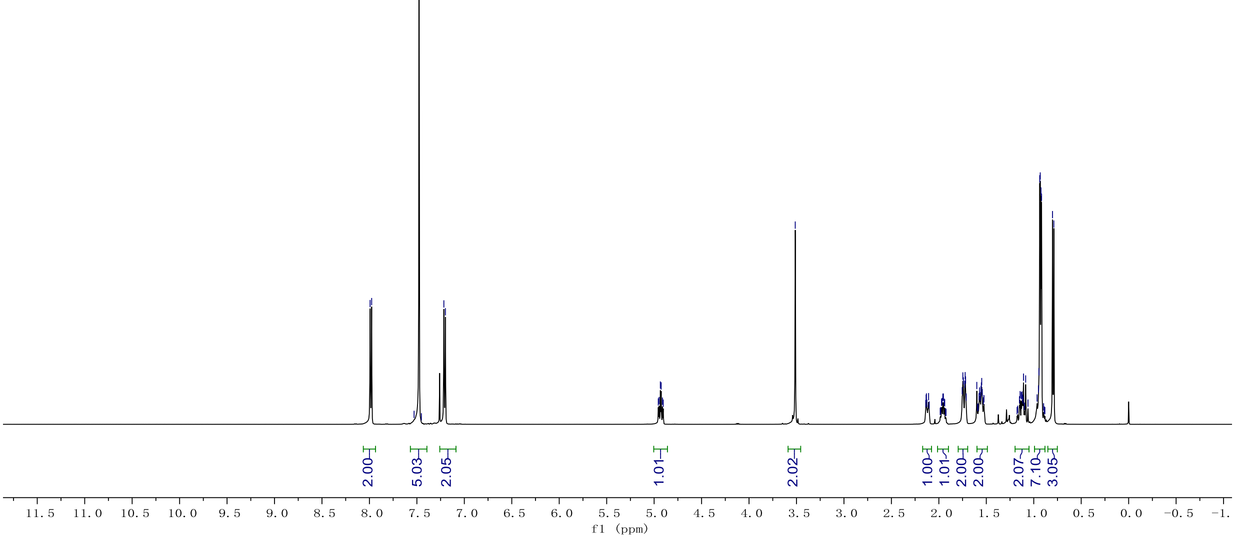

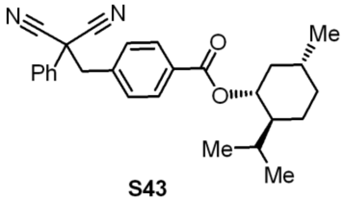

S43

^{1}H NMR (500 MHz, $CDCl_3$)

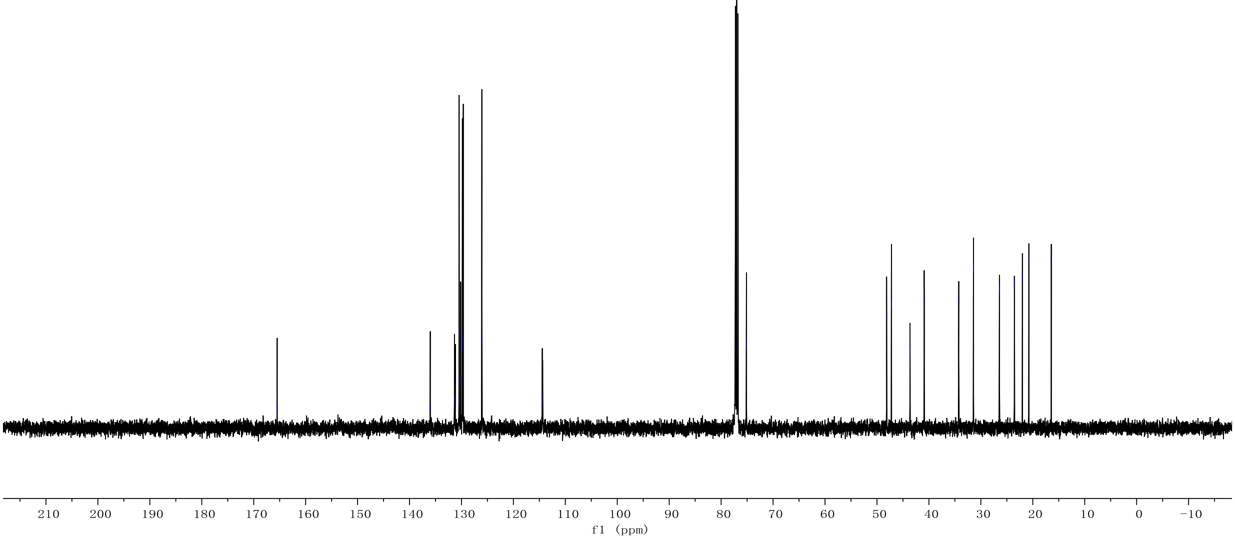

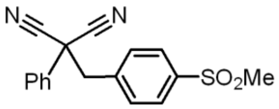

S45

^{1}H NMR (400 MHz, CDCl$_3$)

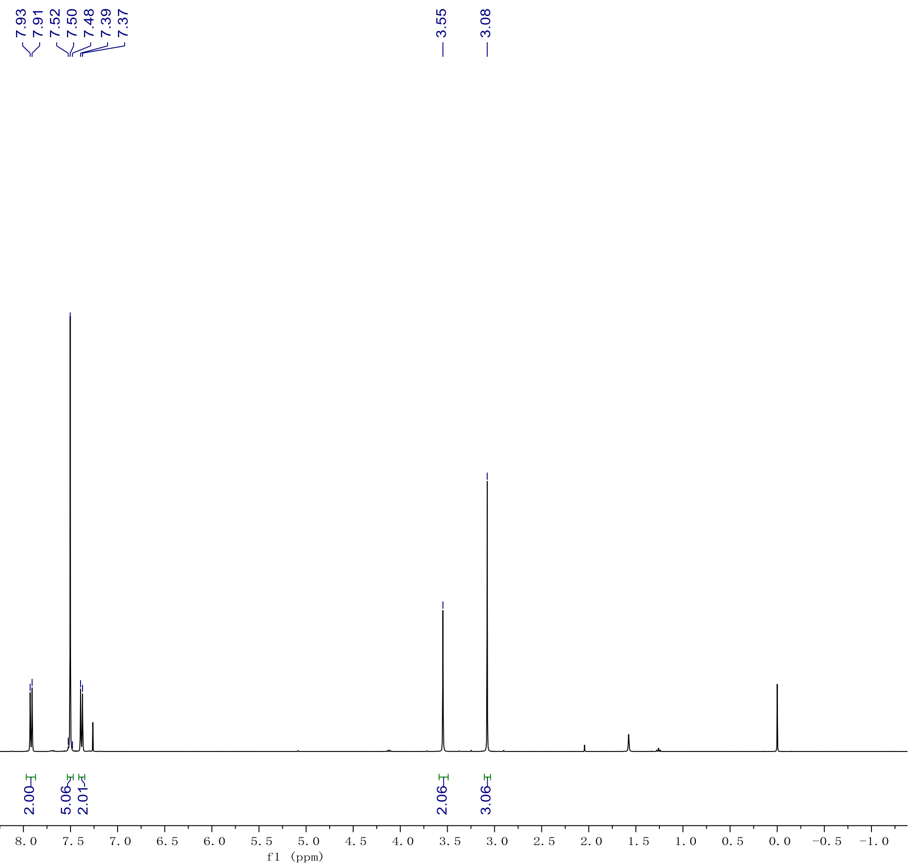

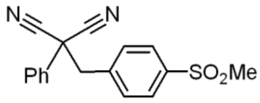

S45

^{13}C NMR (101 MHz, CDCl$_3$)

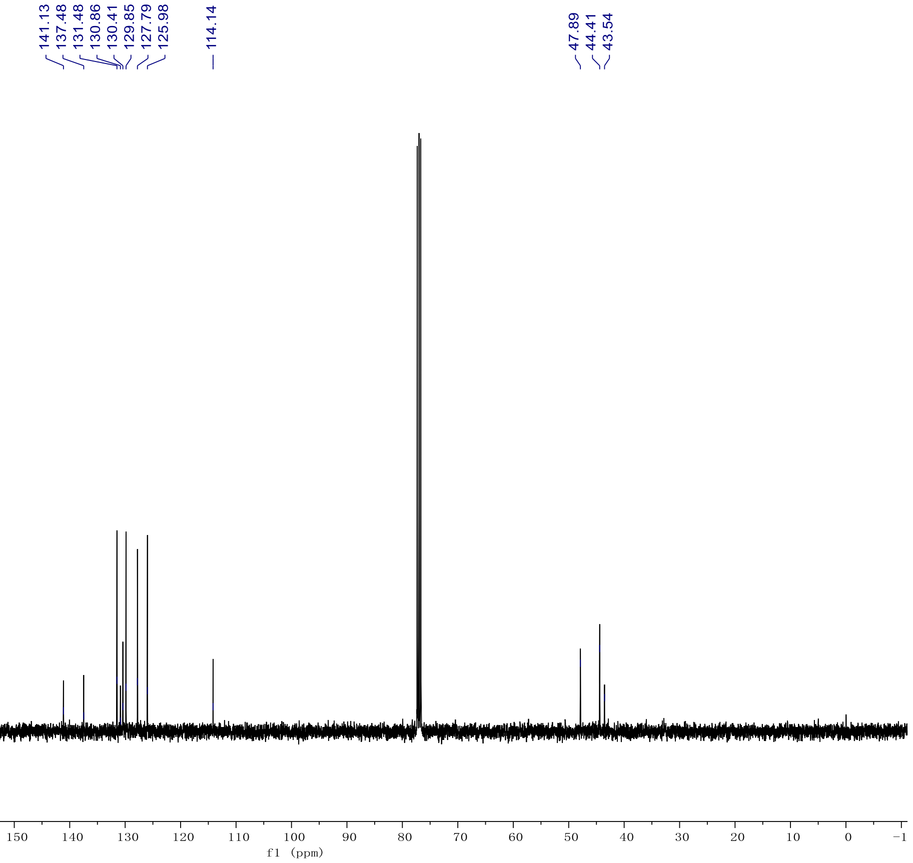

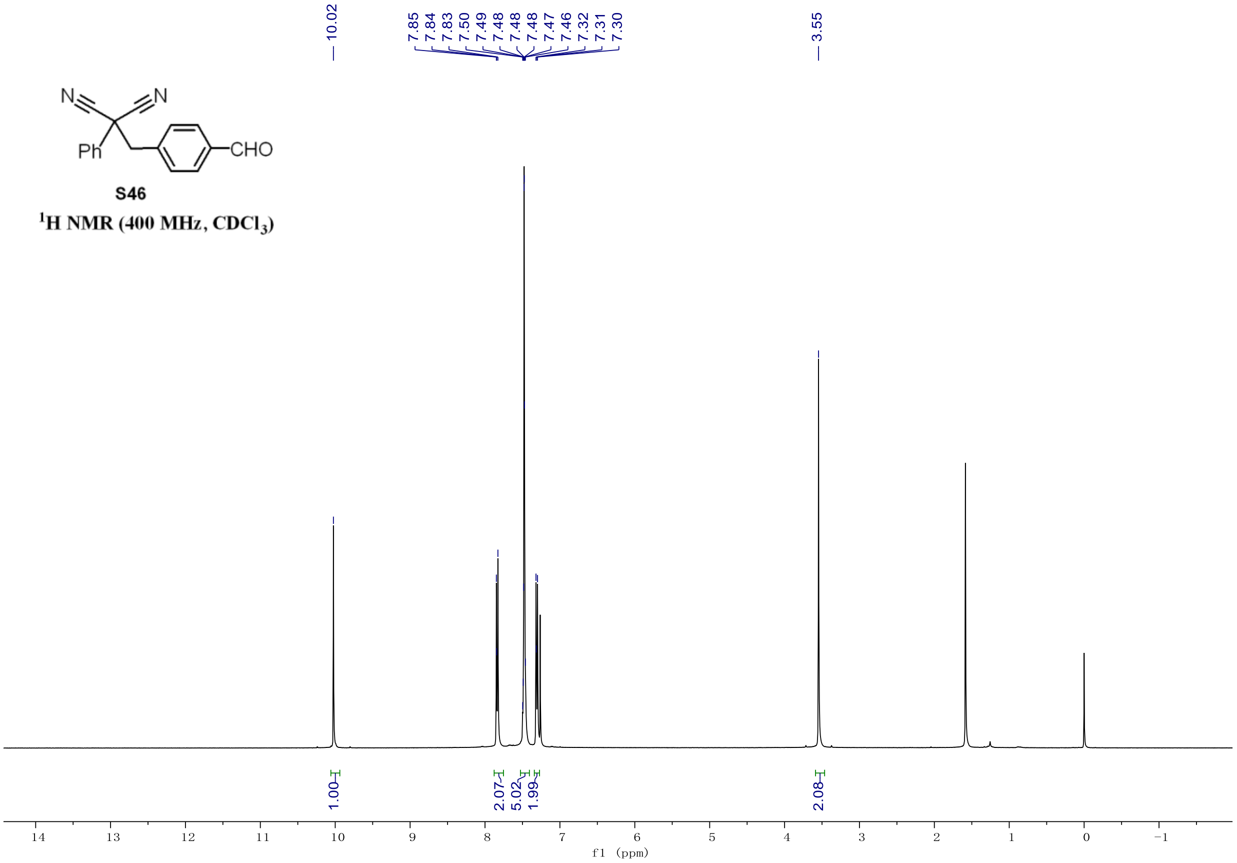

S46

^{1}H NMR (400 MHz, $CDCl_3$)

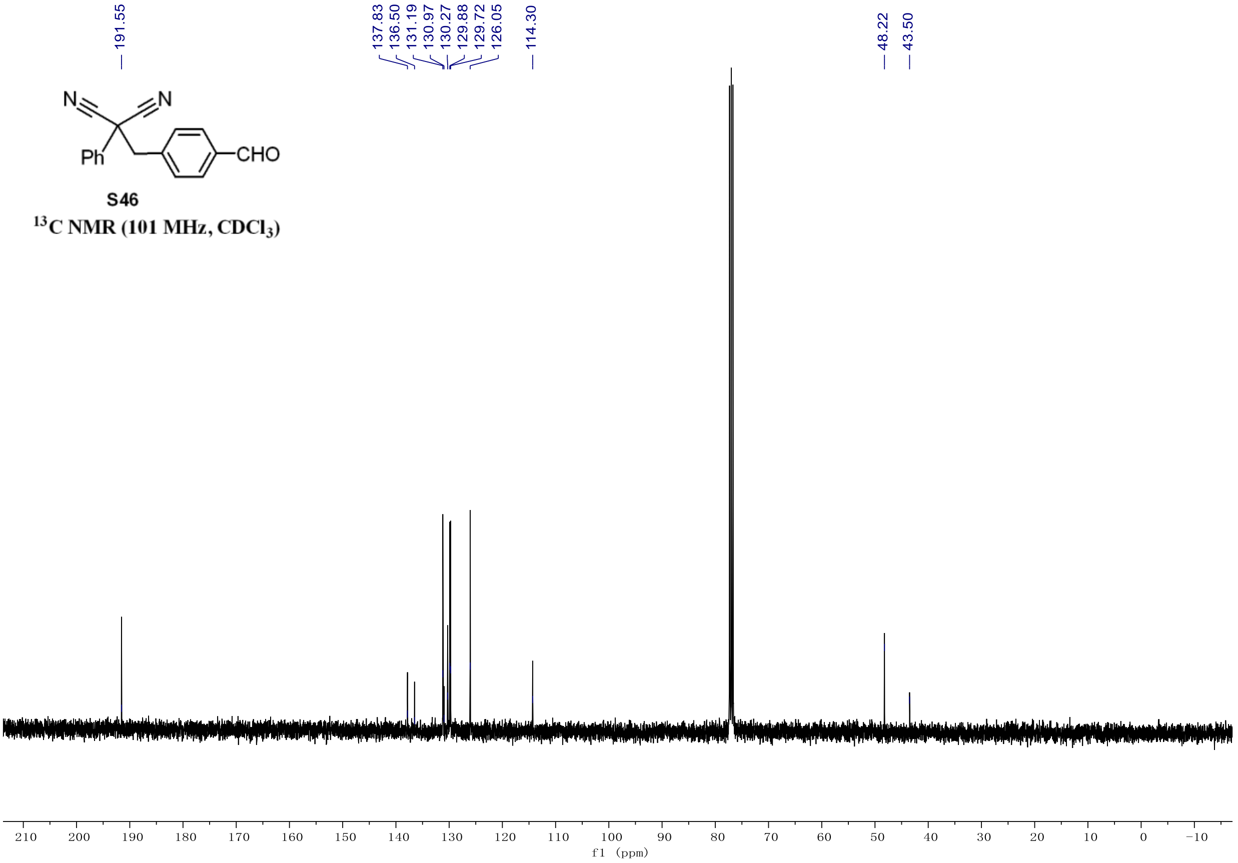

S46

^{13}C NMR (101 MHz, $CDCl_3$)

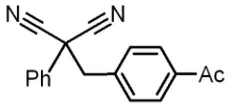

S47

^{1}H NMR (500 MHz, $CDCl_3$)

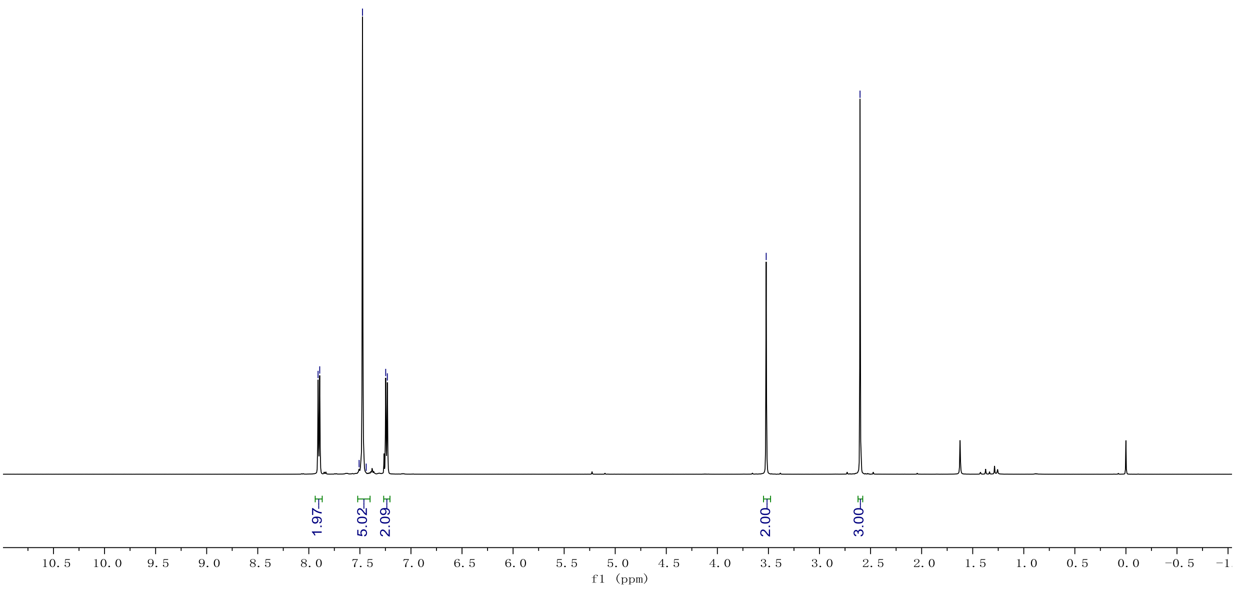

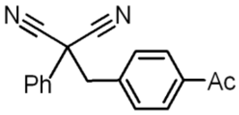

S47

^{13}C NMR (126 MHz, $CDCl_3$)

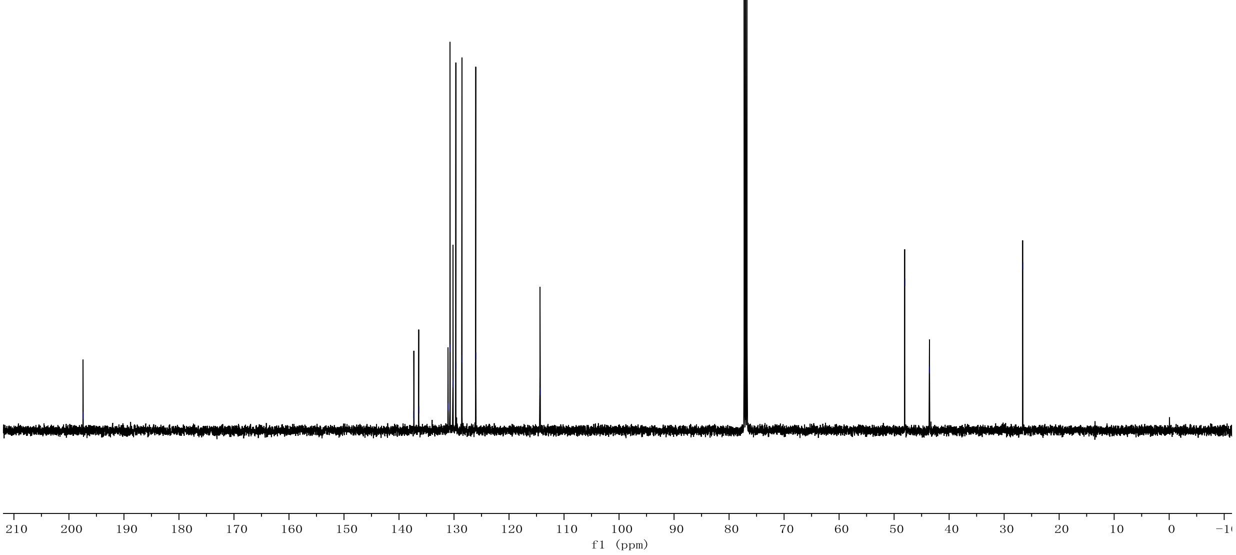

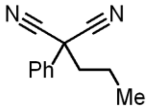

S50

^{1}H NMR (400 MHz, CDCl$_3$)

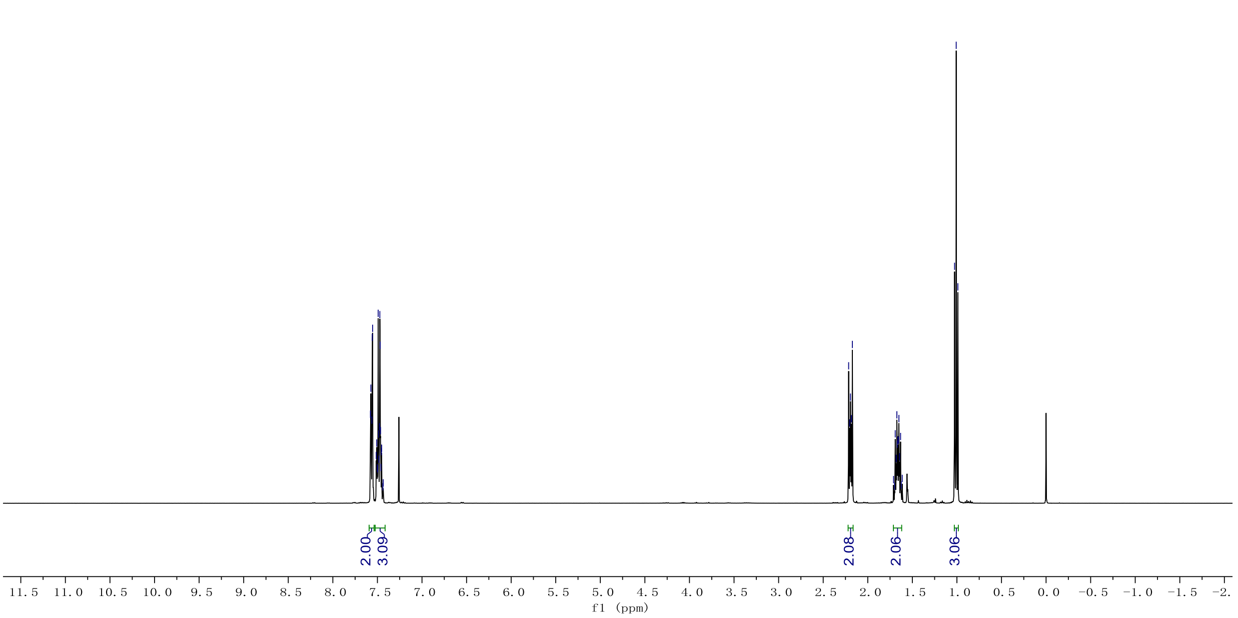

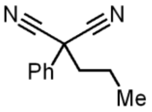

S50

^{13}C NMR (101 MHz, CDCl$_3$)

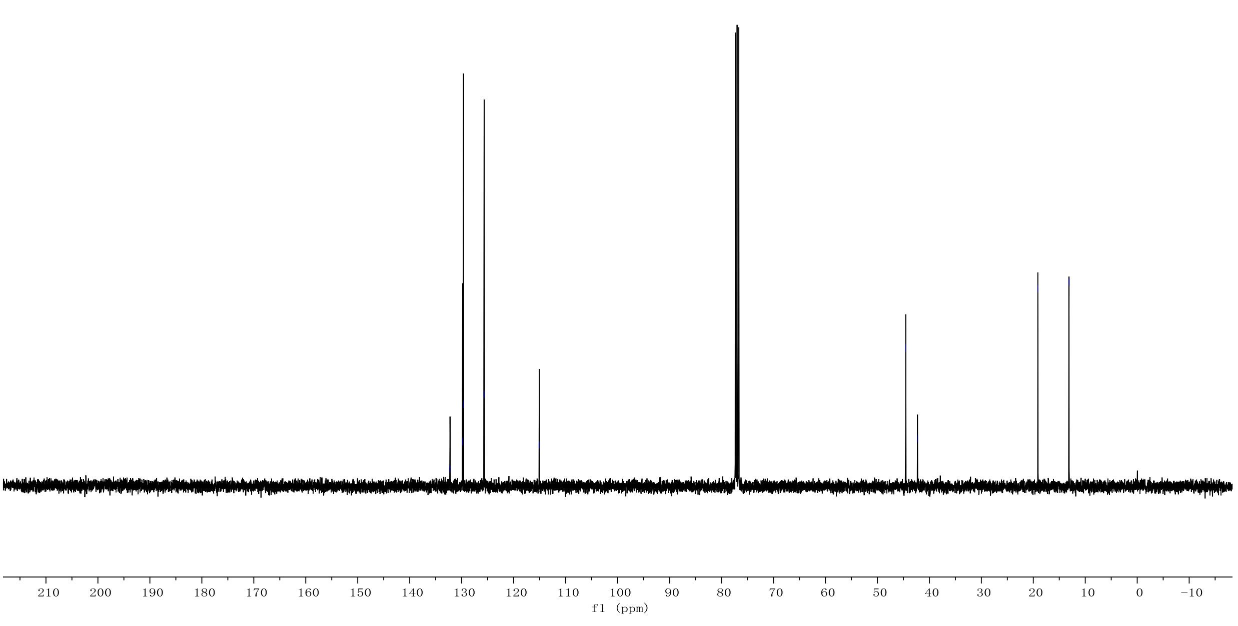

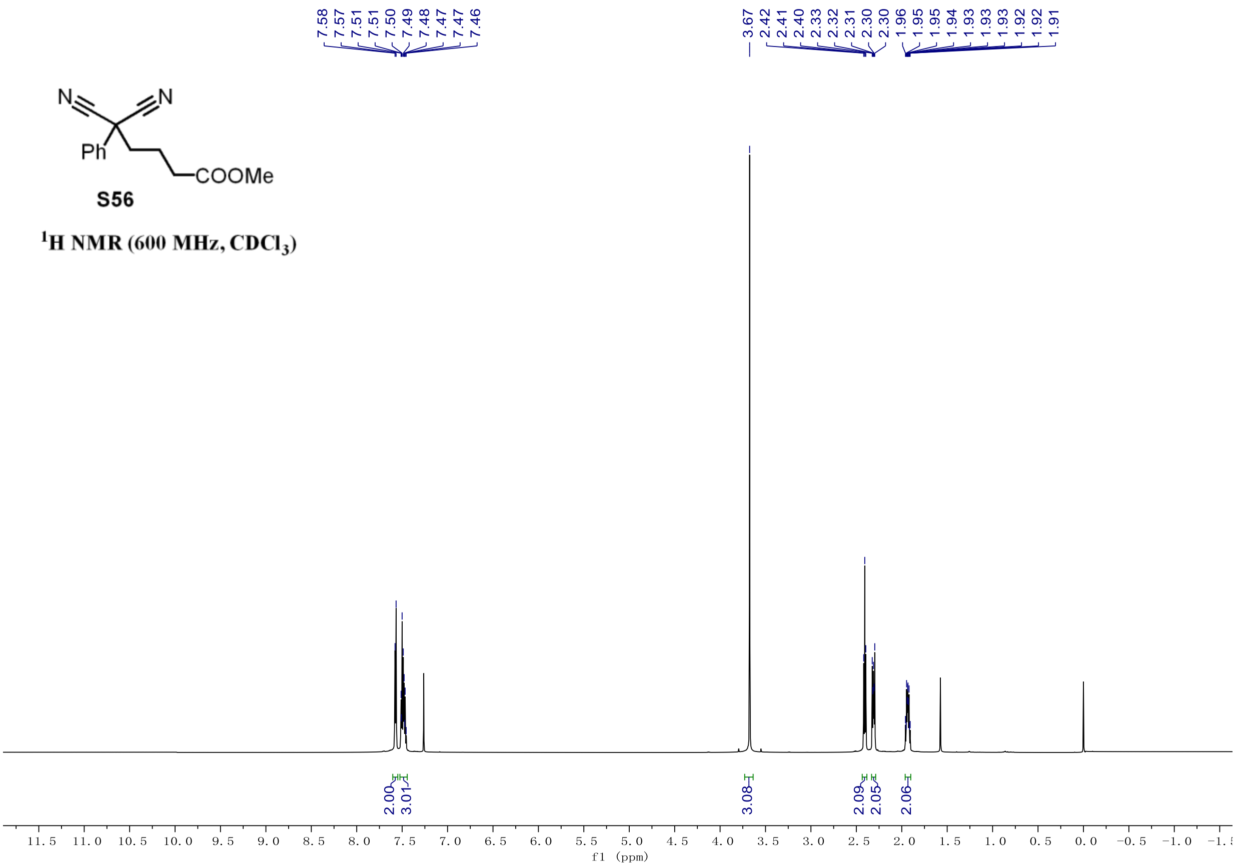
7.58
7.57
7.51
7.51
7.50
7.49
7.48
7.47
7.47
7.46
3.67
2.42
2.41
2.40
2.33
2.32
2.31
2.30
2.30
1.96
1.95
1.95
1.94
1.93
1.93
1.93
1.92
1.92
1.91
N
N
Ph
COOMe
S56
1H NMR (600 MHz, CDCl3)
2.00
3.01
3.08
2.09
2.05
2.06
11.5 11.0 10.5 10.0 9.5 9.0 8.5 8.0 7.5 7.0 6.5 6.0 5.5 5.0 4.5 4.0 3.5 3.0 2.5 2.0 1.5 1.0 0.5 0.0 −0.5 −1.0
f1 (ppm)

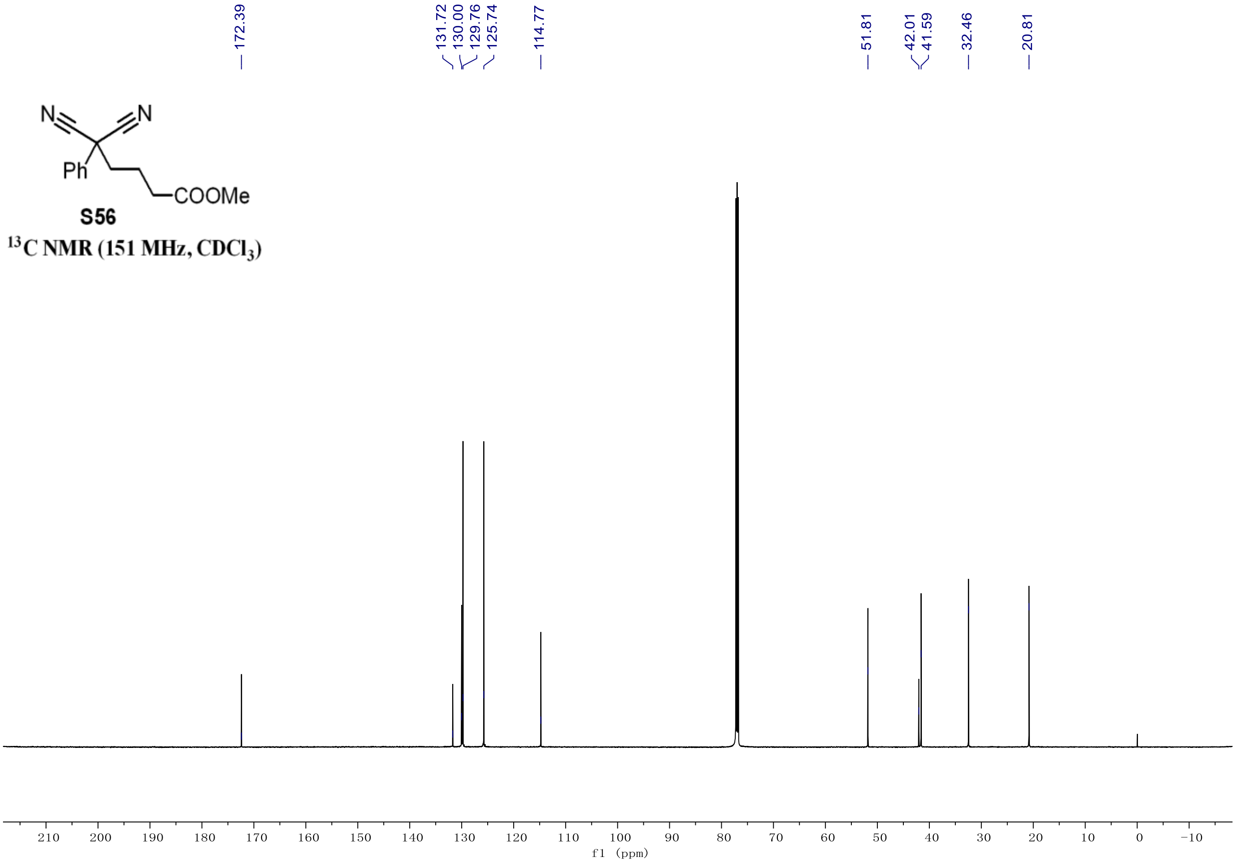
172.39
131.72
130.00
129.76
125.74
114.77
51.81
42.01
41.59
32.46
20.81
N
N
Ph
COOMe
S56
13C NMR (151 MHz, CDCl3)
210 200 190 180 170 160 150 140 130 120 110 100 90 80 70 60 50 40 30 20 10 0 −10
f1 (ppm)

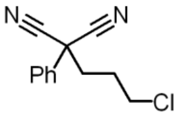

S57

^{1}H NMR (500 MHz, $CDCl_3$)

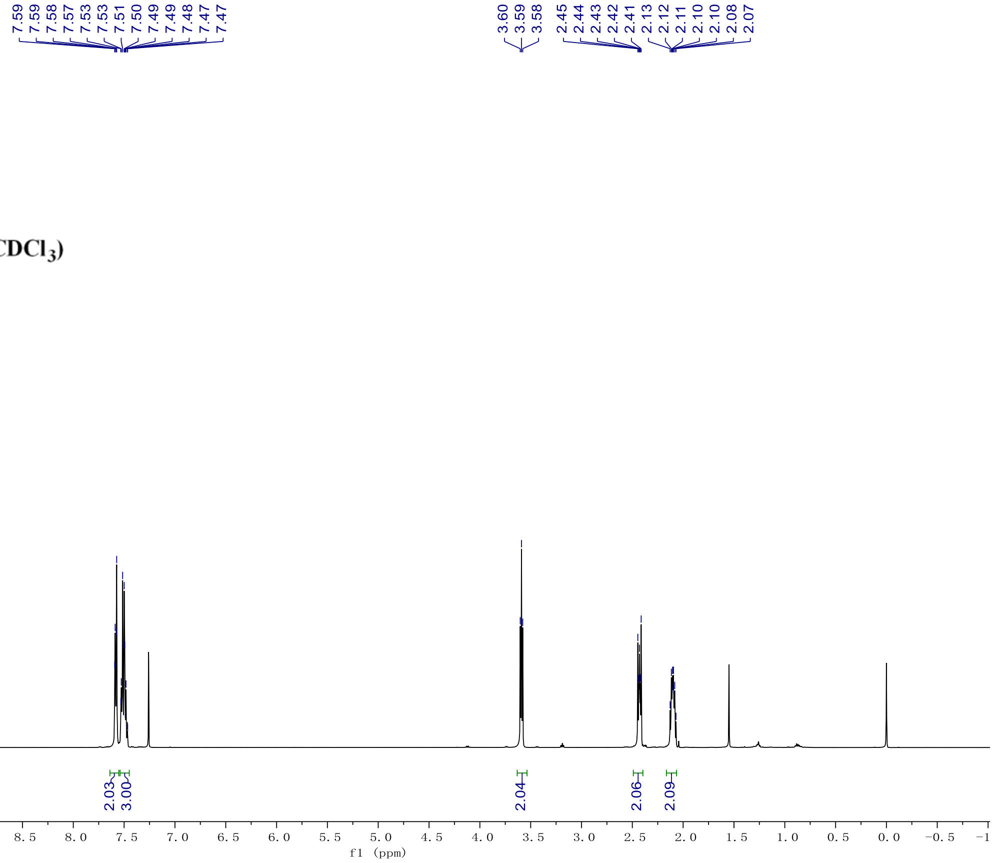

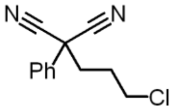

S57

^{13}C NMR (126 MHz, $CDCl_3$)

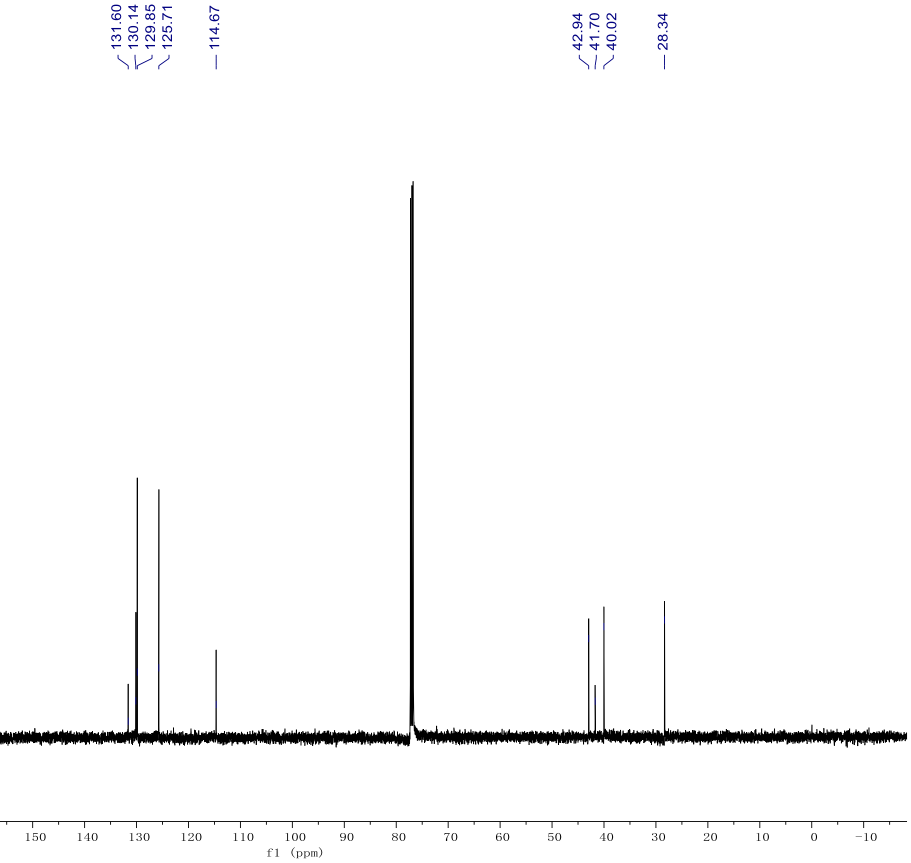

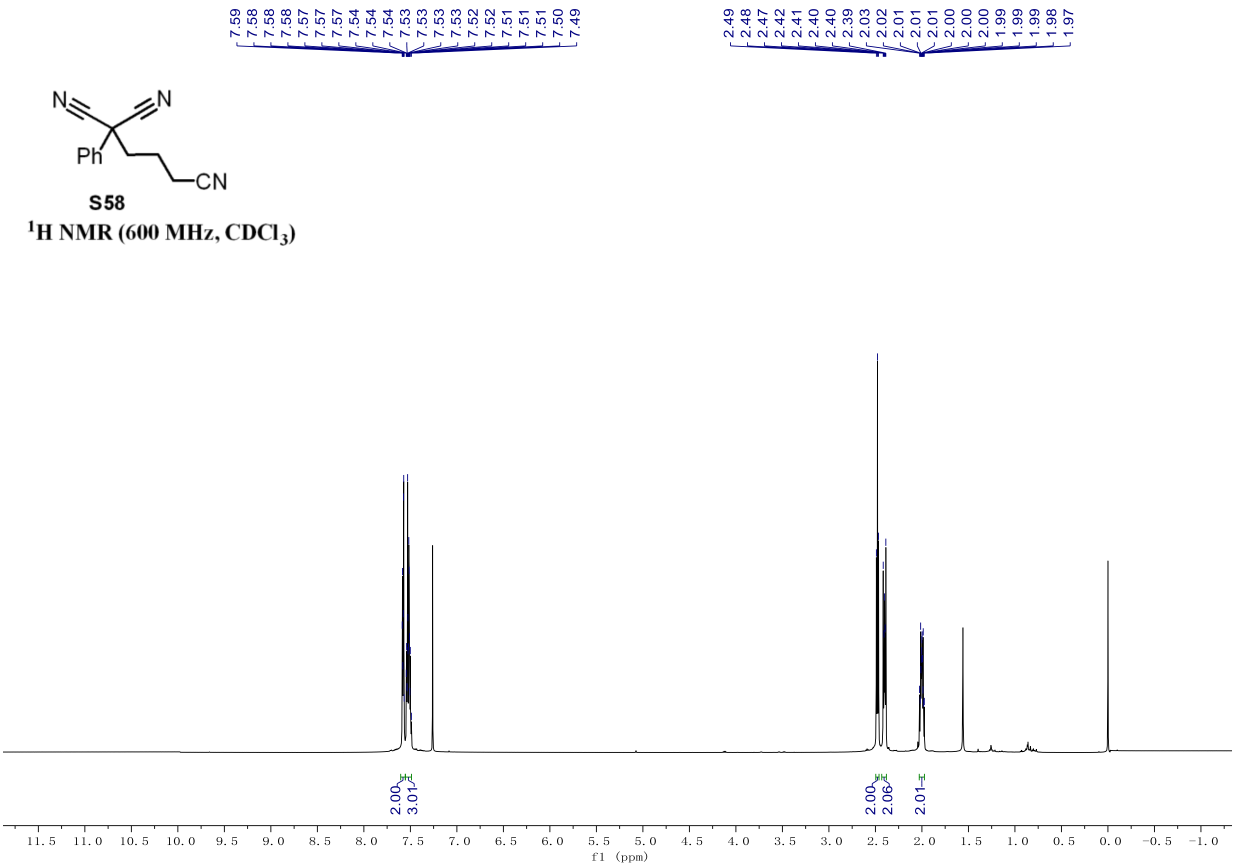

S58

^{1}H NMR (600 MHz, $CDCl_3$)

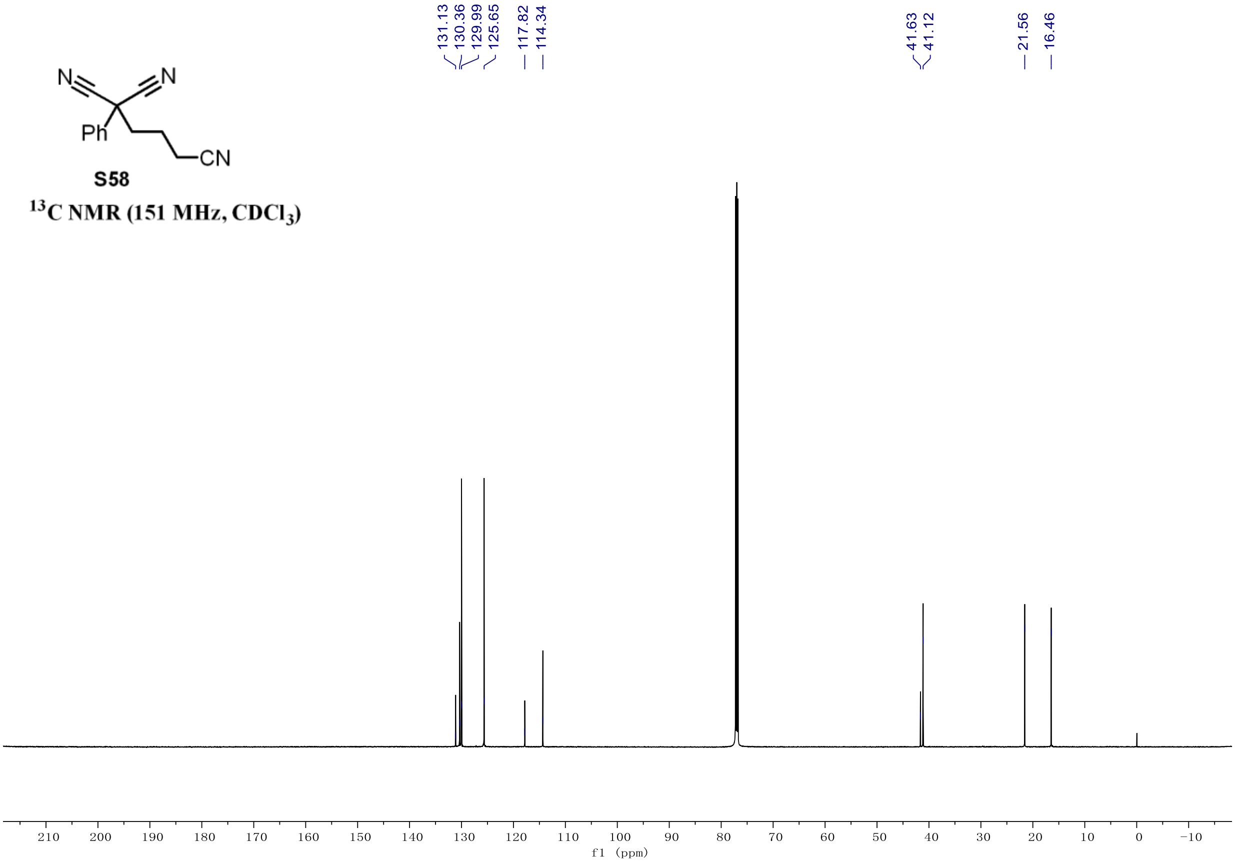

S58

^{13}C NMR (151 MHz, $CDCl_3$)

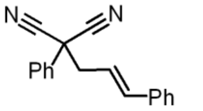

S60

^{1}H NMR (400 MHz, CDCl$_3$)

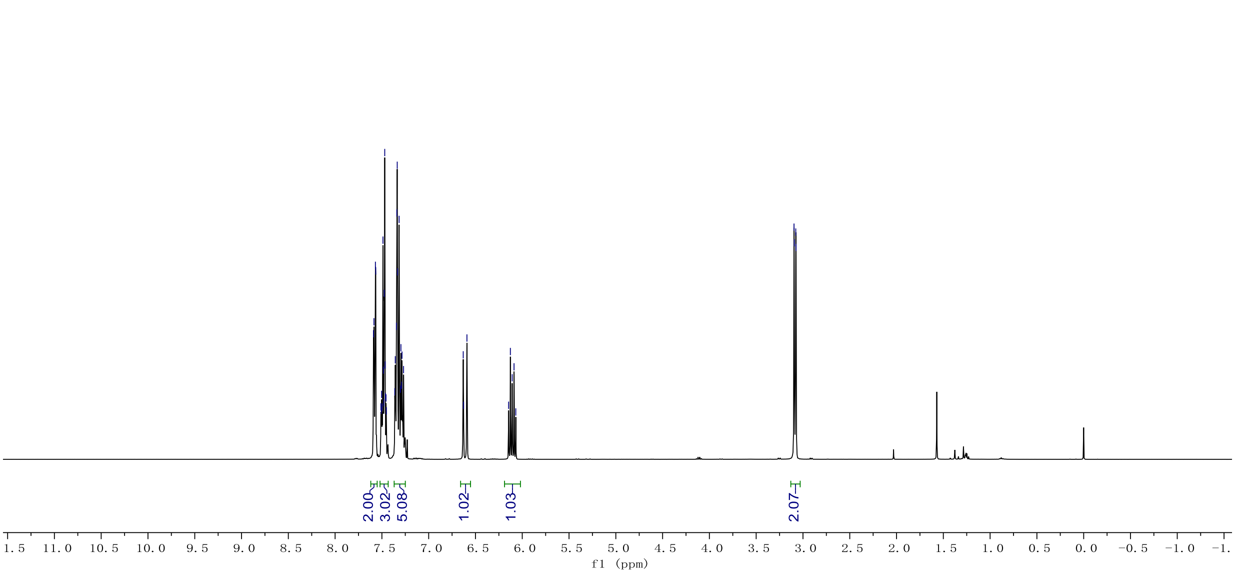

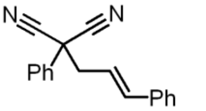

S60

^{13}C NMR (101 MHz, CDCl$_3$)

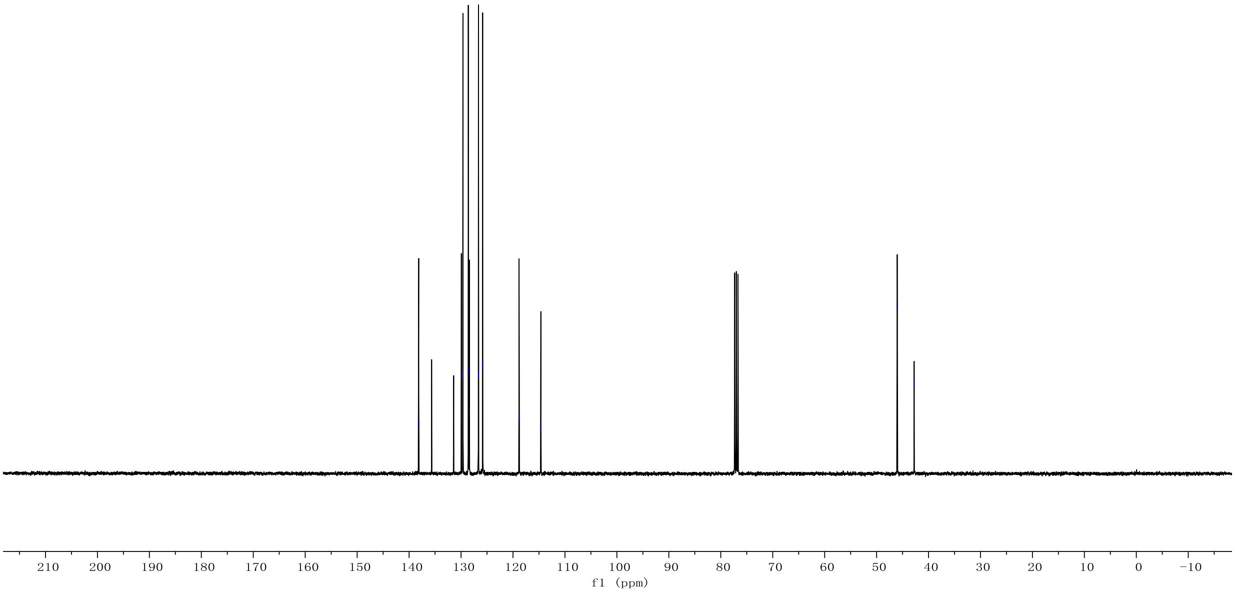

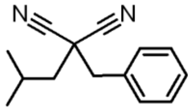

S67

^{1}H NMR (400 MHz, $CDCl_3$)

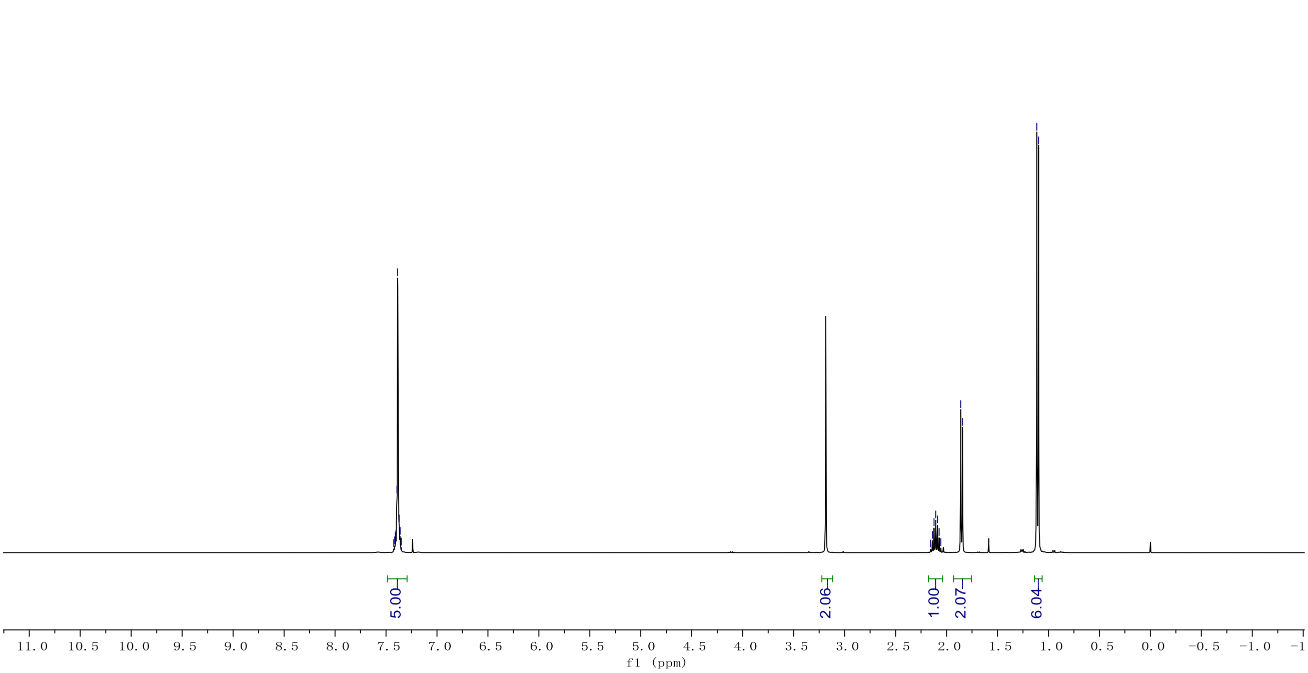

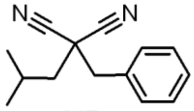

S67

^{13}C NMR (101 MHz, $CDCl_3$)

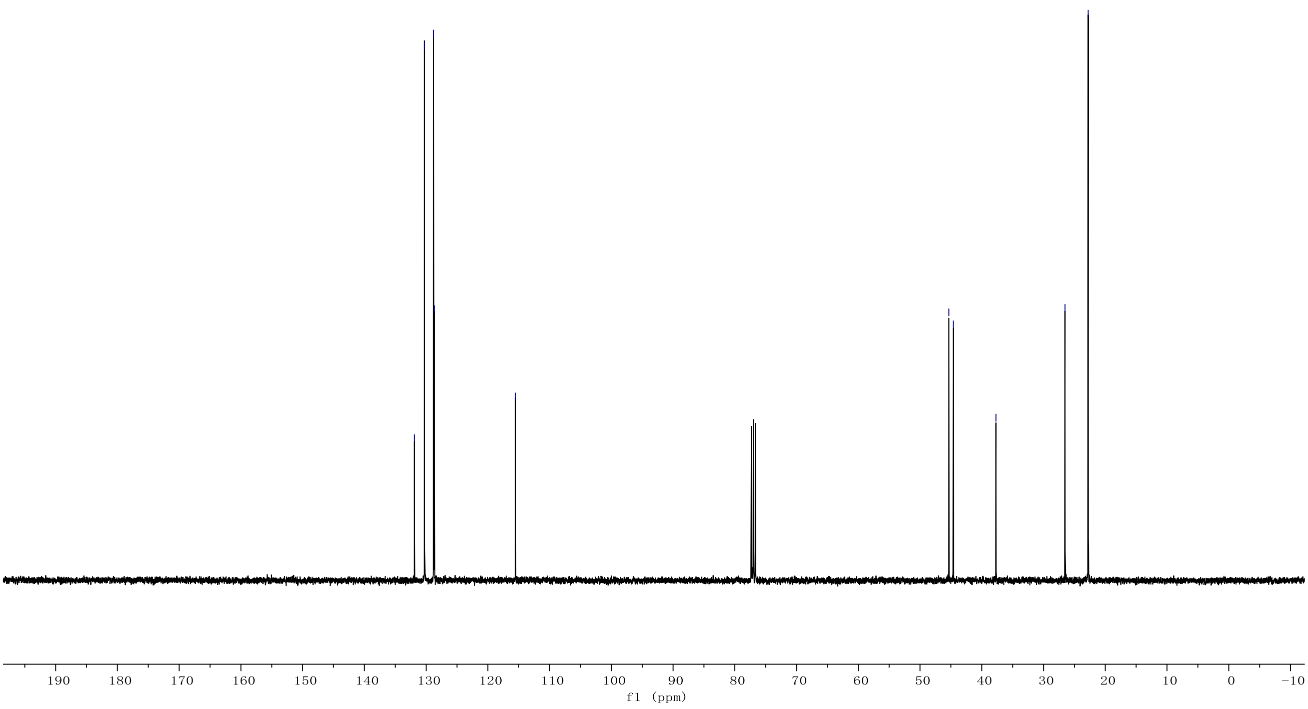

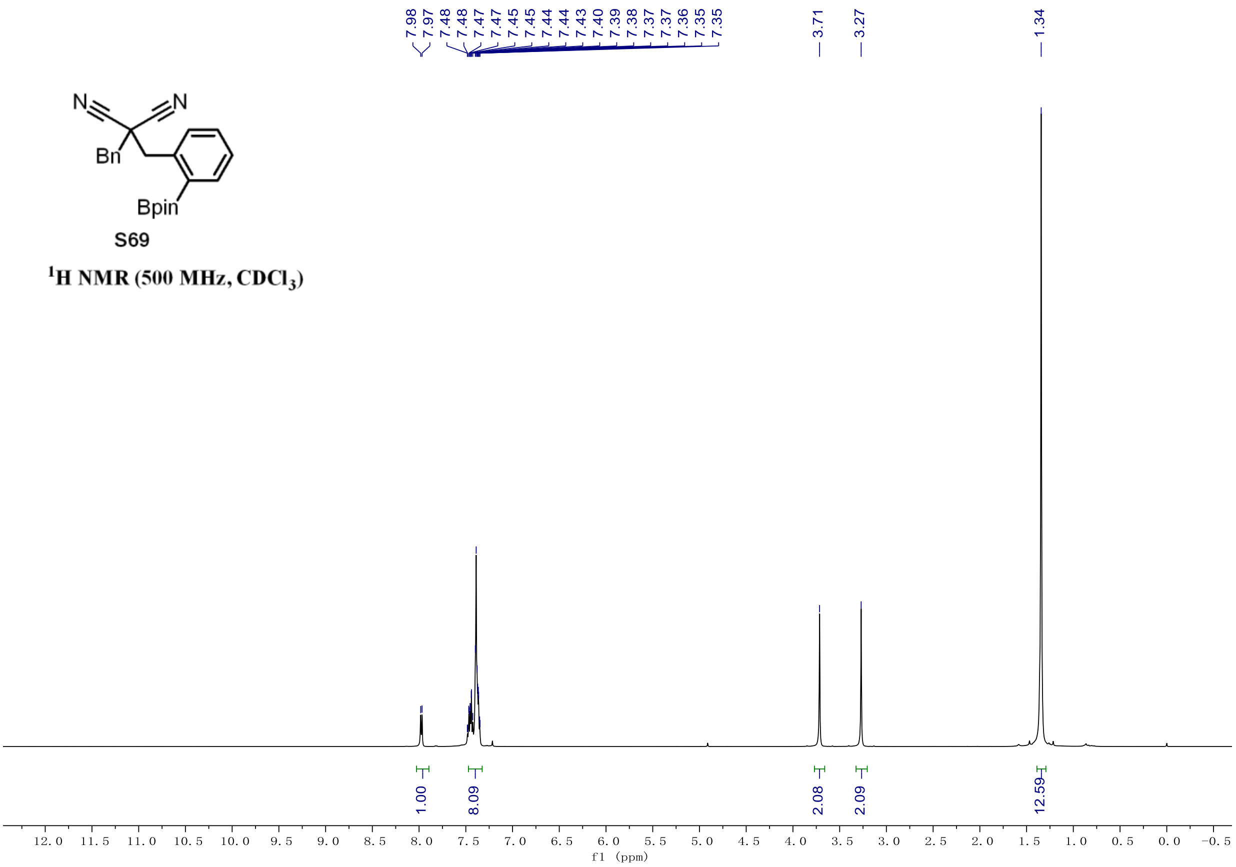
S69
^{1}H NMR (500 MHz, CDCl$_3$)
7.98 7.97 7.48 7.48 7.47 7.47 7.45 7.45 7.44 7.44 7.43 7.40 7.39 7.38 7.37 7.37 7.36 7.35 7.35
3.71
3.27
1.34
1.00 8.09 2.08 2.09 12.59
f1 (ppm)

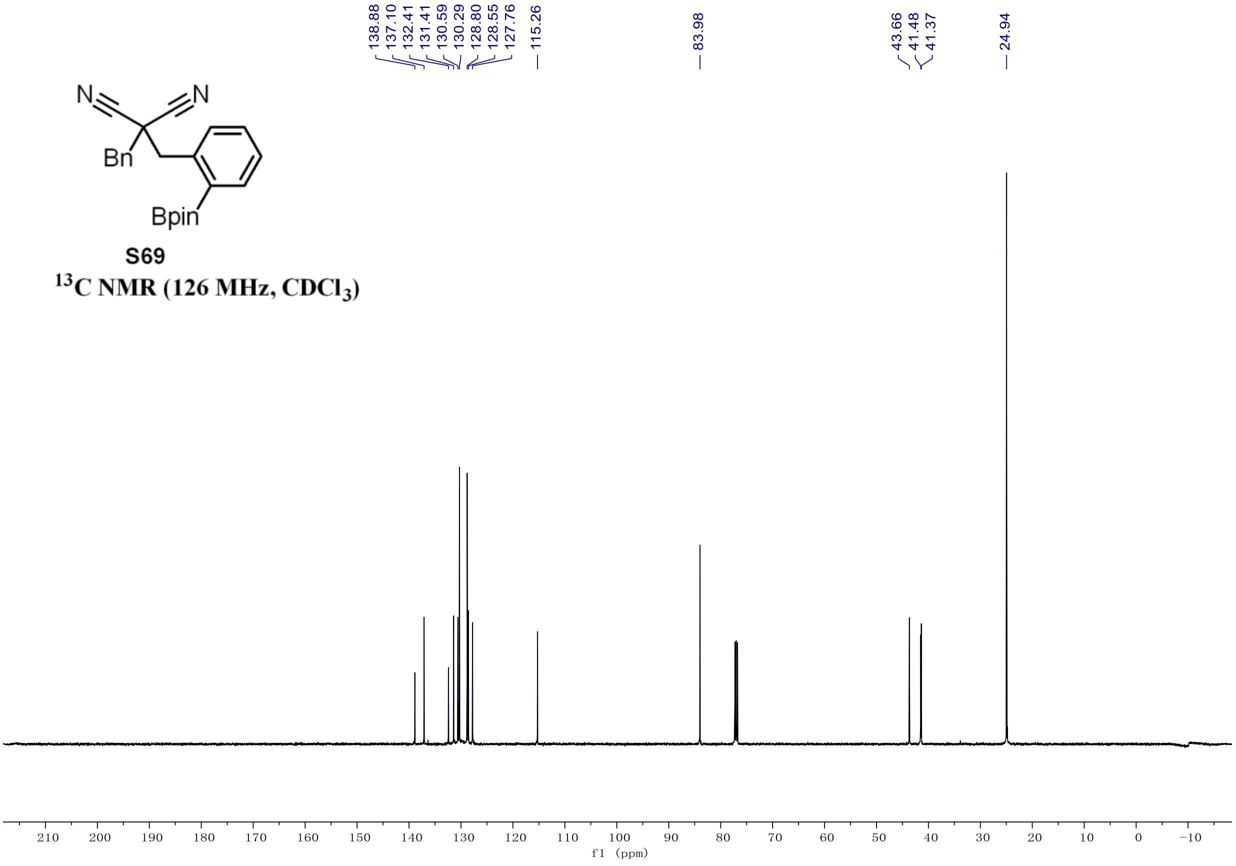
S69
^{13}C NMR (126 MHz, CDCl$_3$)
138.88 137.10 132.41 131.41 130.59 130.29 128.80 128.55 127.76
115.26
83.98
43.66 41.48 41.37
24.94
f1 (ppm)

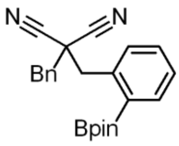

S69

^{11}B NMR (160 MHz, $CDCl_3$)

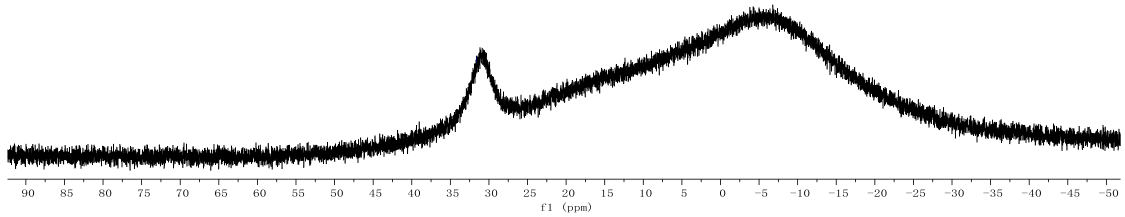

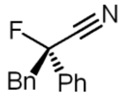

S85

^{1}H NMR (400 MHz, $CDCl_3$)

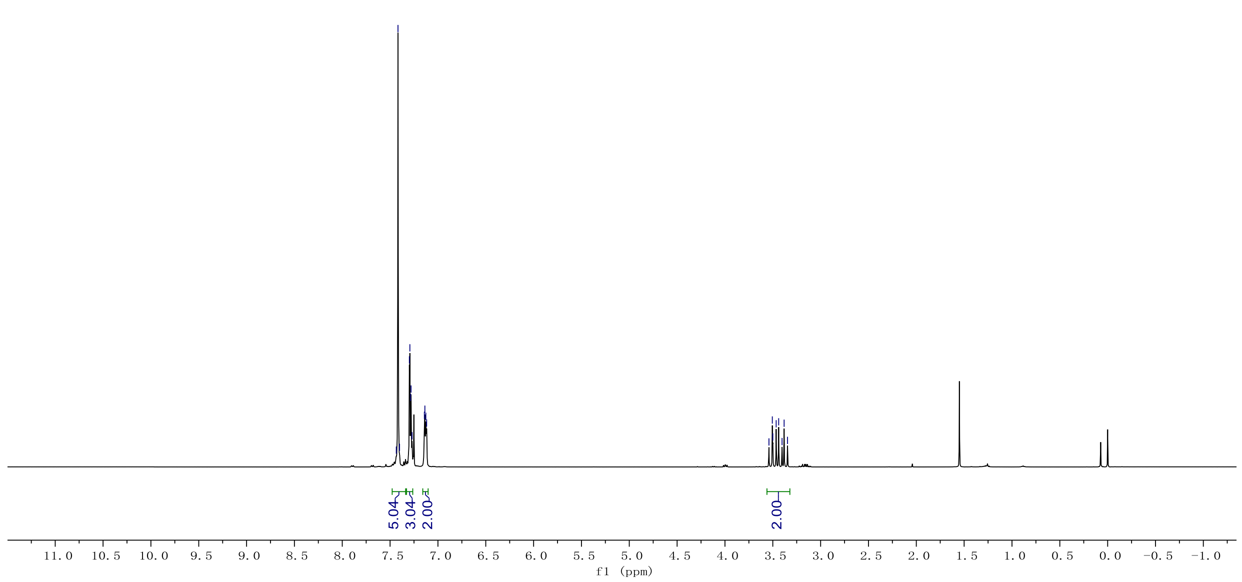

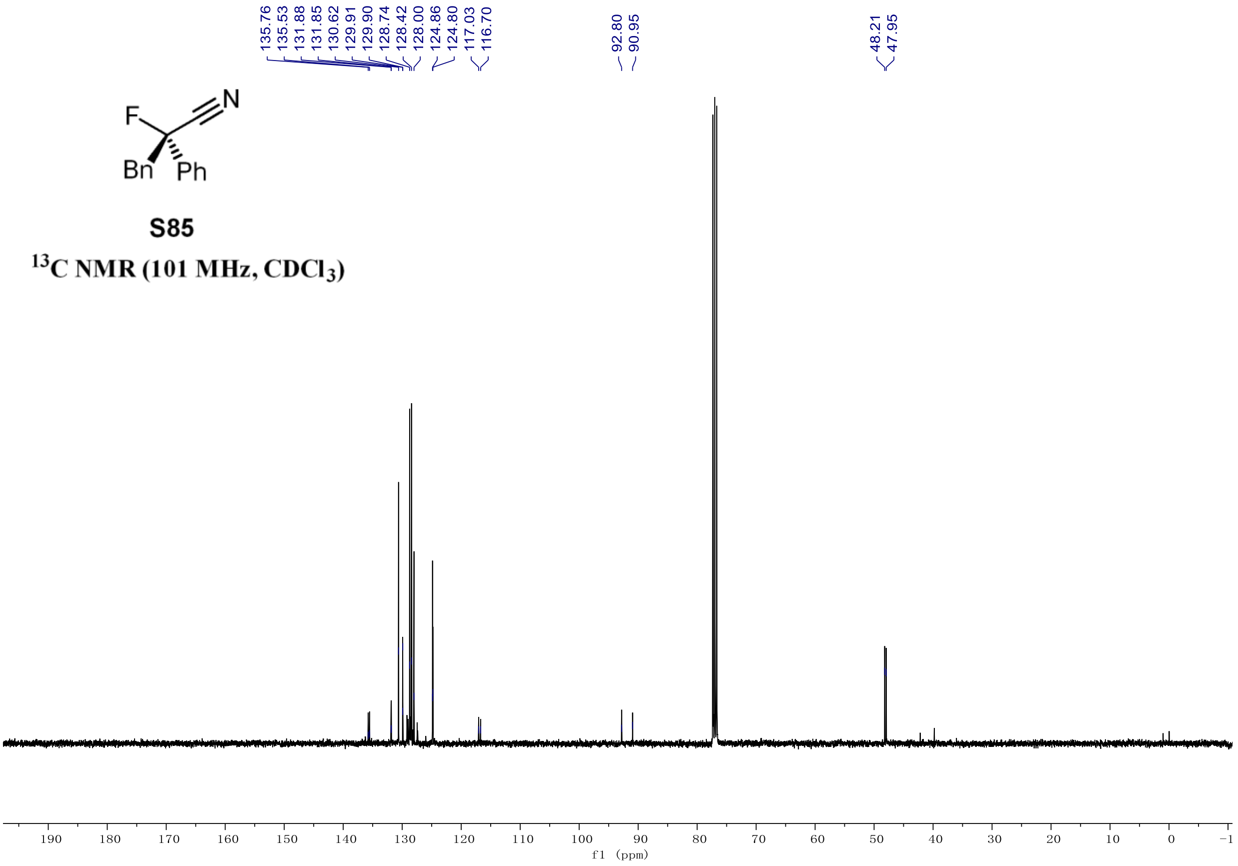

S85

^{13}C NMR (101 MHz, $CDCl_3$)

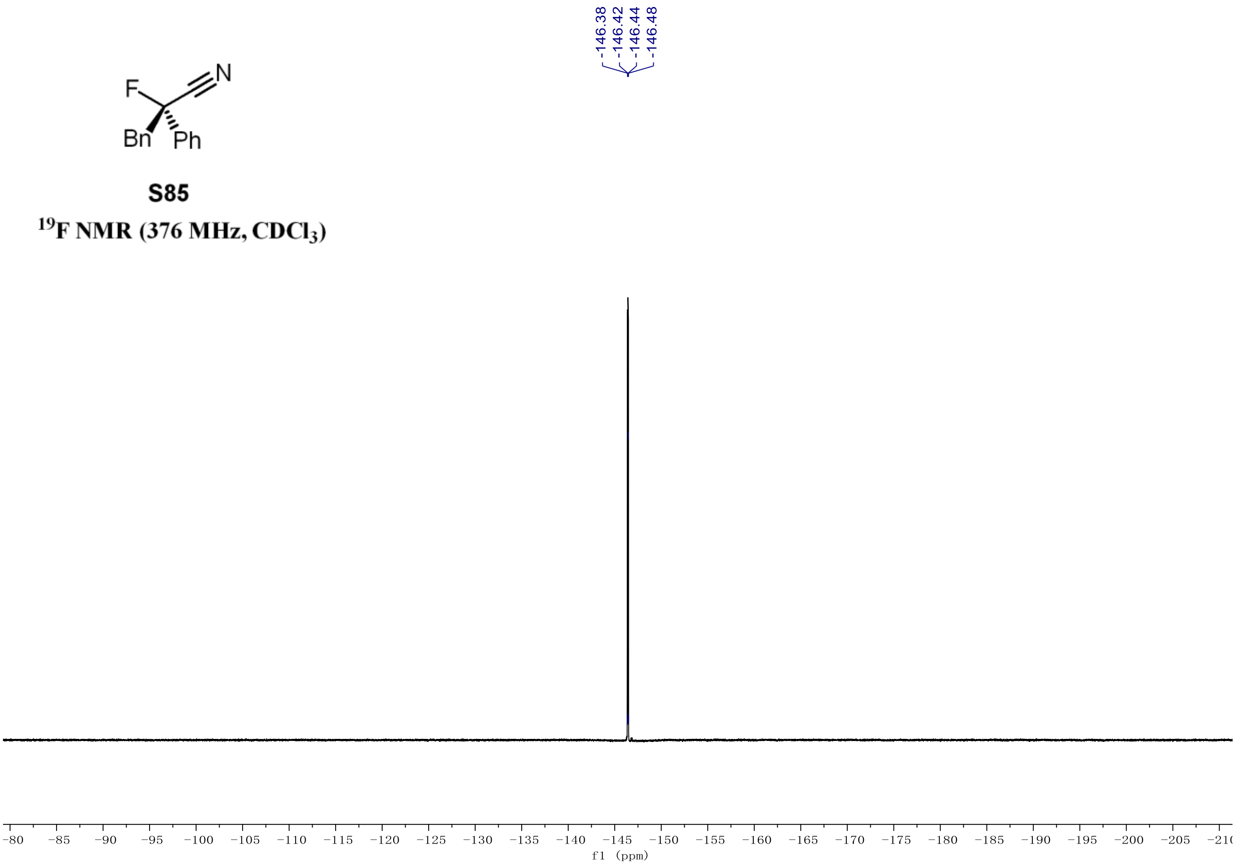

S85

^{19}F NMR (376 MHz, $CDCl_3$)

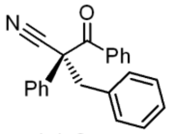

(+)-**2**

^{1}H NMR (500 MHz, CDCl$_3$)

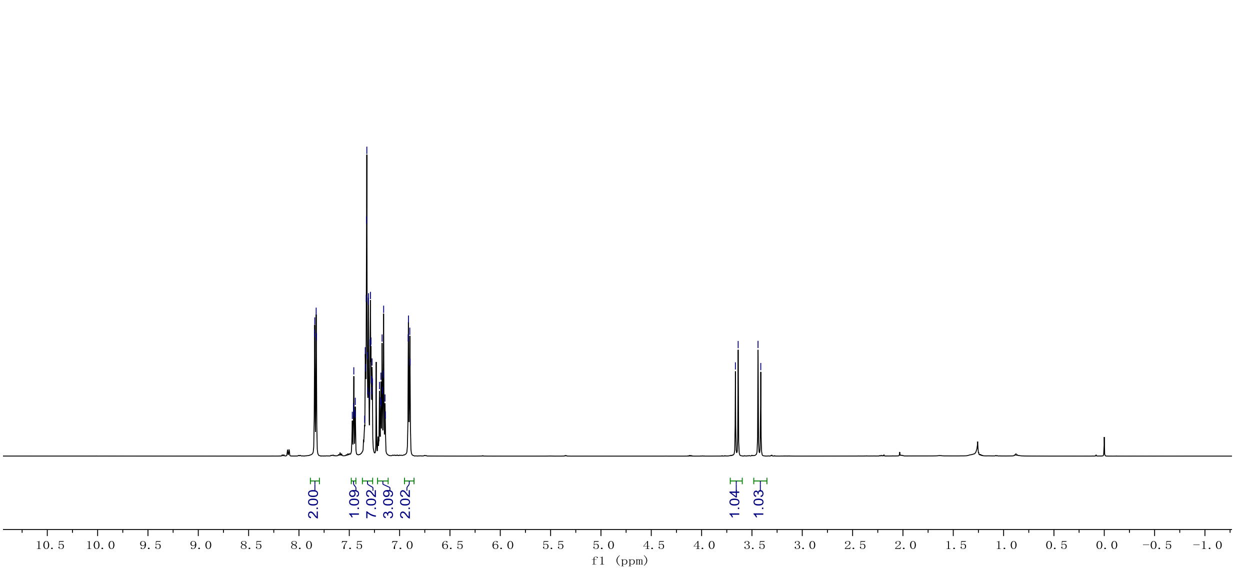

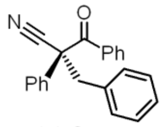

(+)-**2**

^{13}C NMR (126 MHz, CDCl$_3$)

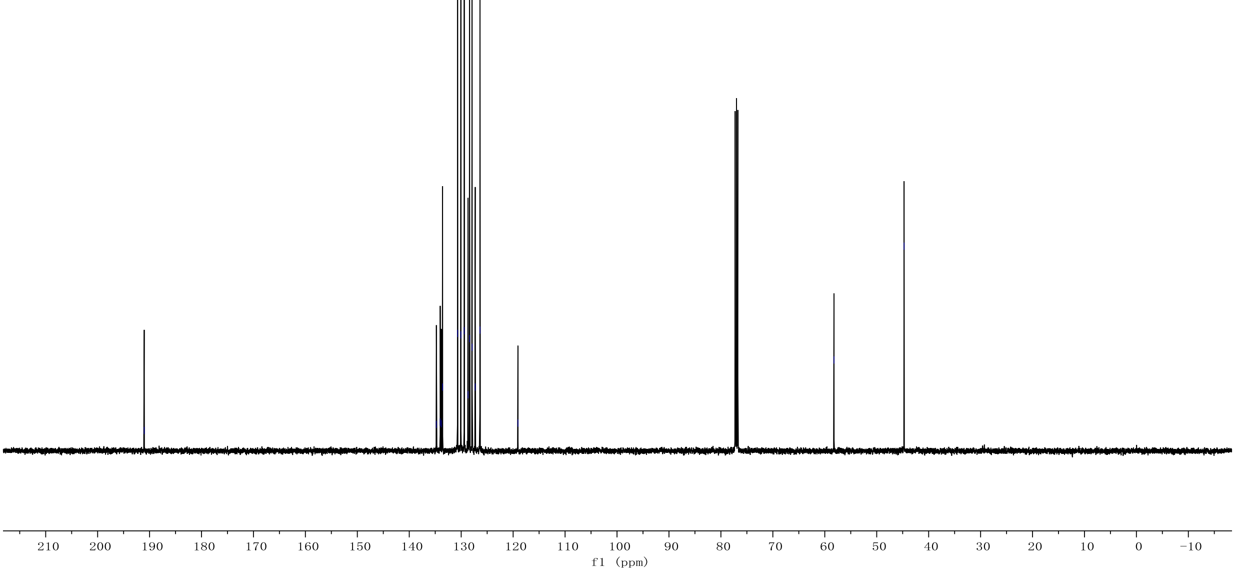

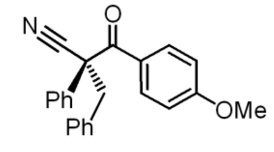

(+)-**3**

^{1}H NMR (400 MHz, CDCl$_3$)

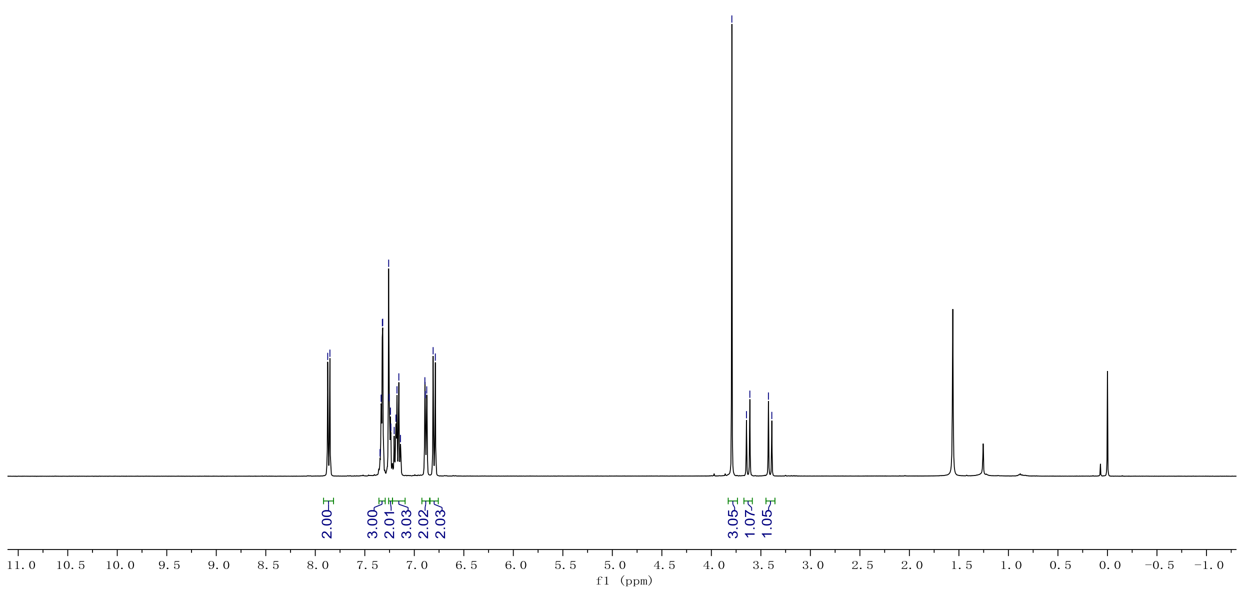

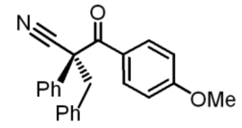

(+)-**3**

^{13}C NMR (101 MHz, CDCl$_3$)

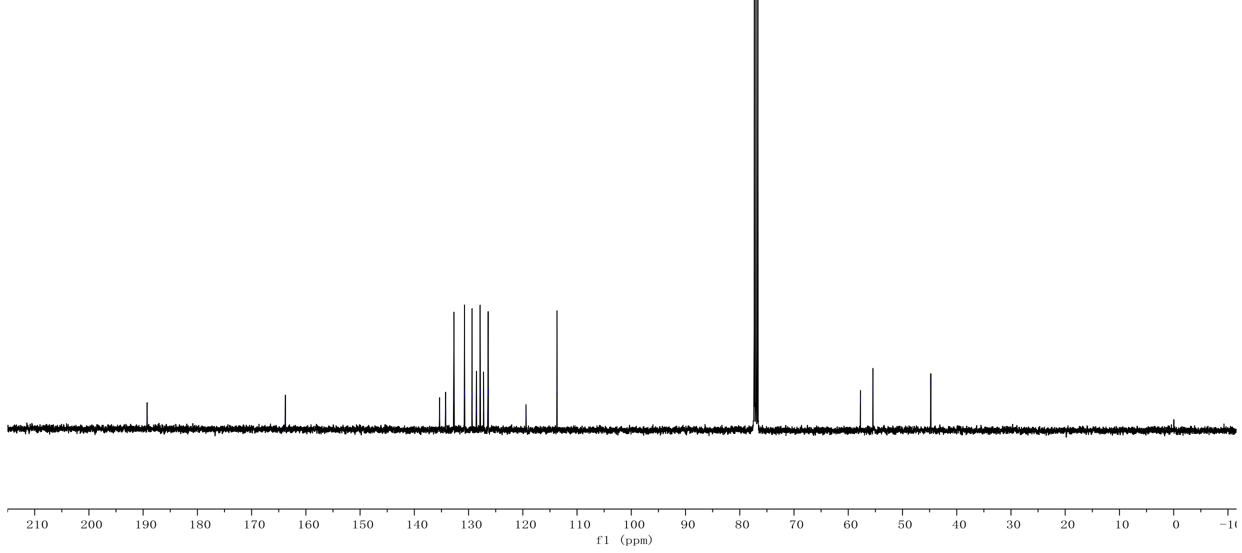

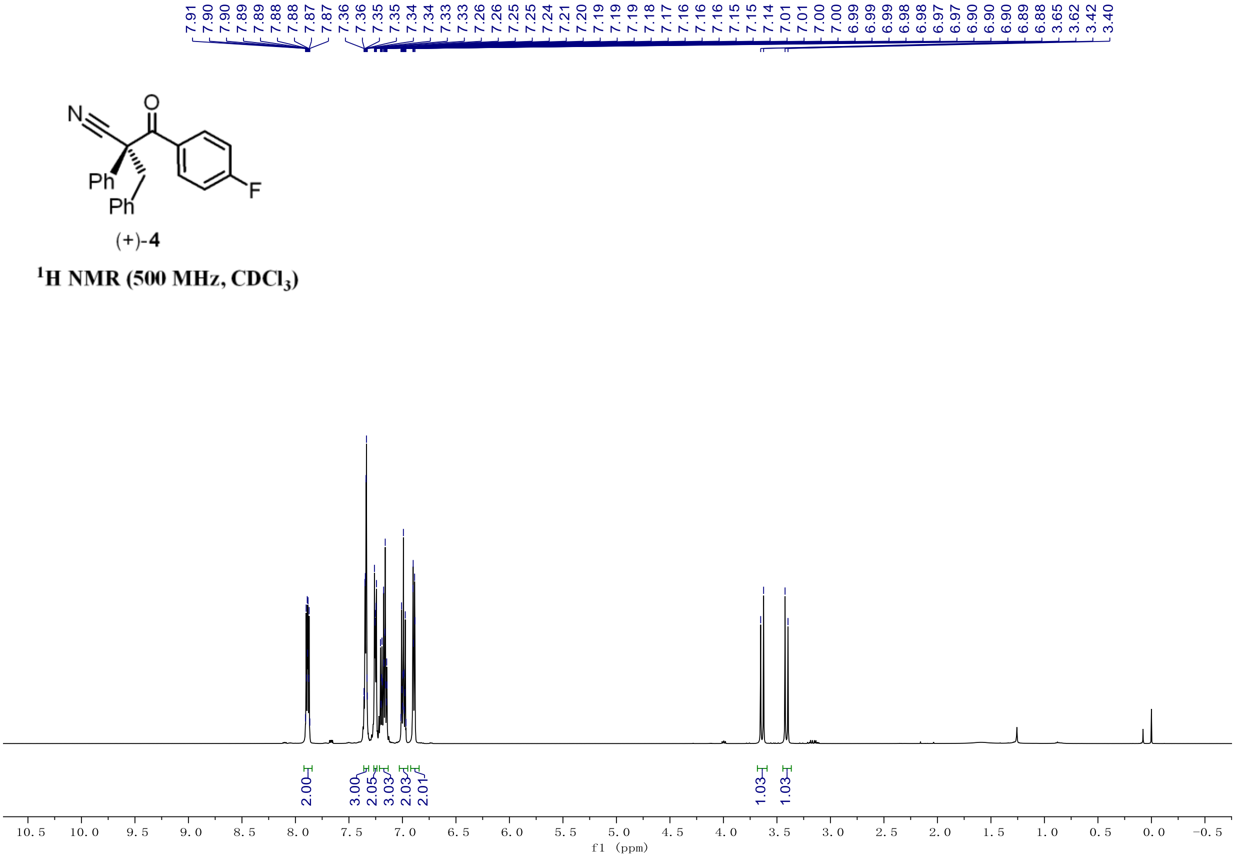
7.91 7.90 7.90 7.89 7.89 7.88 7.88 7.87 7.87 7.36 7.36 7.35 7.35 7.34 7.34 7.33 7.33 7.26 7.26 7.25 7.25 7.24 7.21 7.20 7.19 7.19 7.18 7.17 7.16 7.16 7.15 7.15 7.14 7.01 7.00 6.99 6.99 6.98 6.98 6.97 6.97 6.90 6.90 6.89 6.88 3.65 3.62 3.42 3.40
N
O
Ph
Ph
F
(+)-4
^{1}H NMR (500 MHz, CDCl$_3$)
2.00 3.00 2.05 3.03 2.03 2.01 1.03 1.03
10.5 10.0 9.5 9.0 8.5 8.0 7.5 7.0 6.5 6.0 5.5 5.0 4.5 4.0 3.5 3.0 2.5 2.0 1.5 1.0 0.5 0.0 -0.5
f1 (ppm)

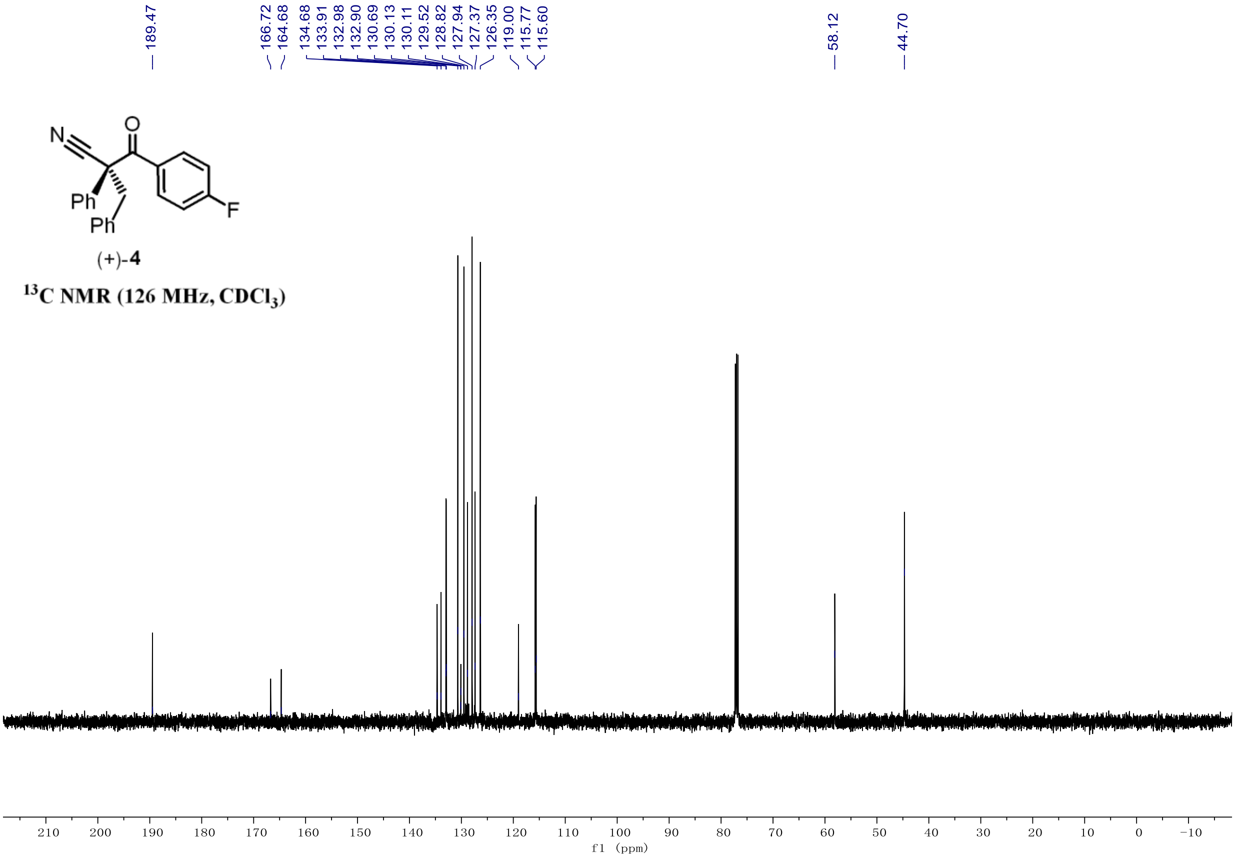
189.47
166.72 164.68 134.68 133.91 132.98 132.90 130.69 130.13 130.11 129.52 128.82 127.94 127.37 126.35 119.00 115.77 115.60
58.12
44.70
N
O
Ph
Ph
F
(+)-4
^{13}C NMR (126 MHz, CDCl$_3$)
210 200 190 180 170 160 150 140 130 120 110 100 90 80 70 60 50 40 30 20 10 0 -10
f1 (ppm)

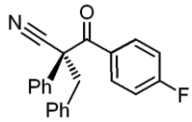

(+)-**4**

^{19}F NMR (471 MHz, CDCl$_3$)

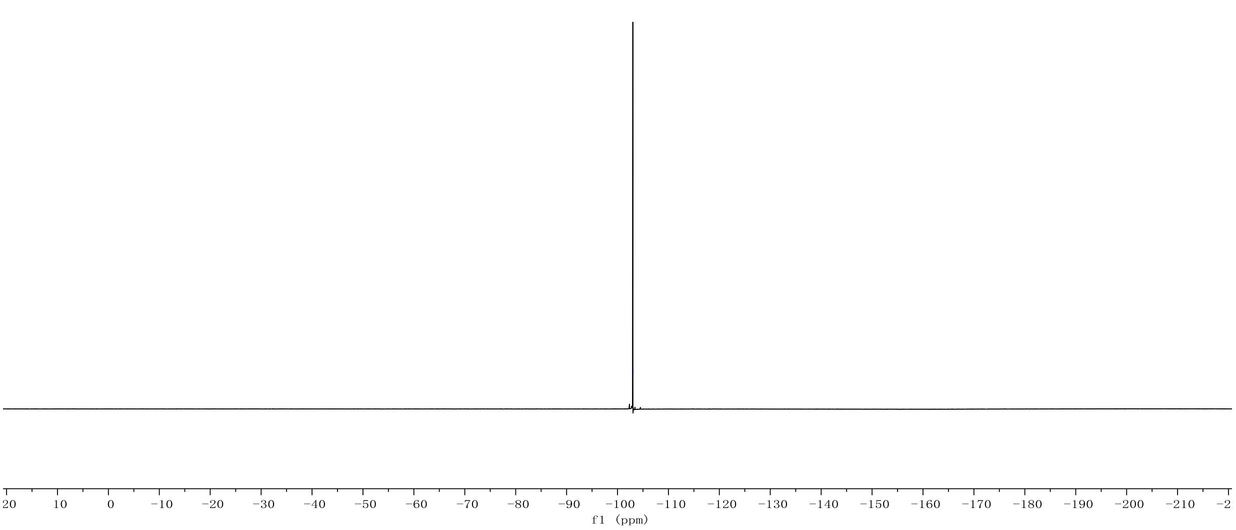

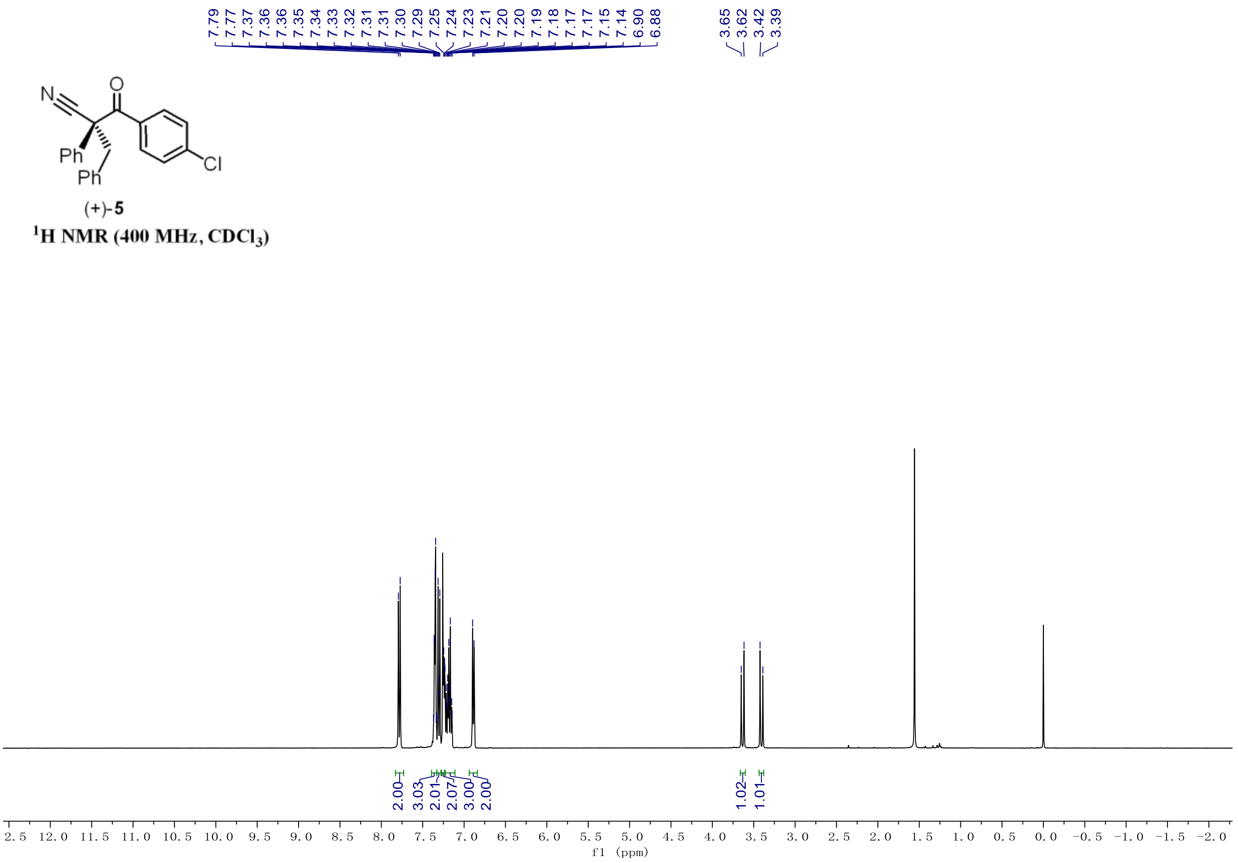
7.79 7.77 7.37 7.36 7.36 7.35 7.34 7.33 7.32 7.31 7.31 7.30 7.29 7.25 7.24 7.23 7.21 7.20 7.20 7.19 7.18 7.17 7.17 7.15 7.14 6.90 6.88
3.65 3.62 3.42 3.39
(+)-5
^{1}H NMR (400 MHz, CDCl$_3$)
2.00 3.03 2.01 2.07 3.00 2.00
1.02 1.01
f1 (ppm)

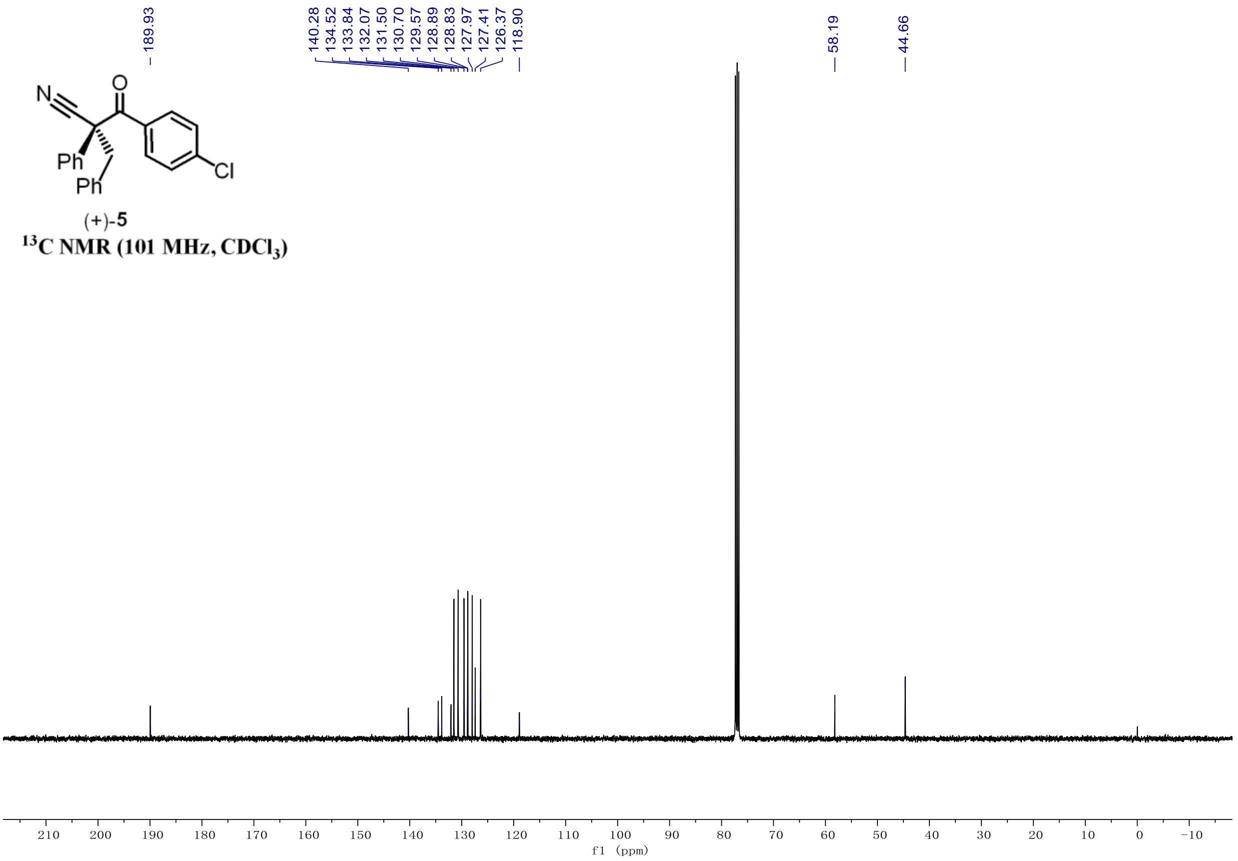
189.93
140.28 134.52 133.84 132.07 131.50 130.70 129.57 128.89 128.83 127.97 127.41 126.37 118.90
58.19
44.66
(+)-5
^{13}C NMR (101 MHz, CDCl$_3$)
f1 (ppm)

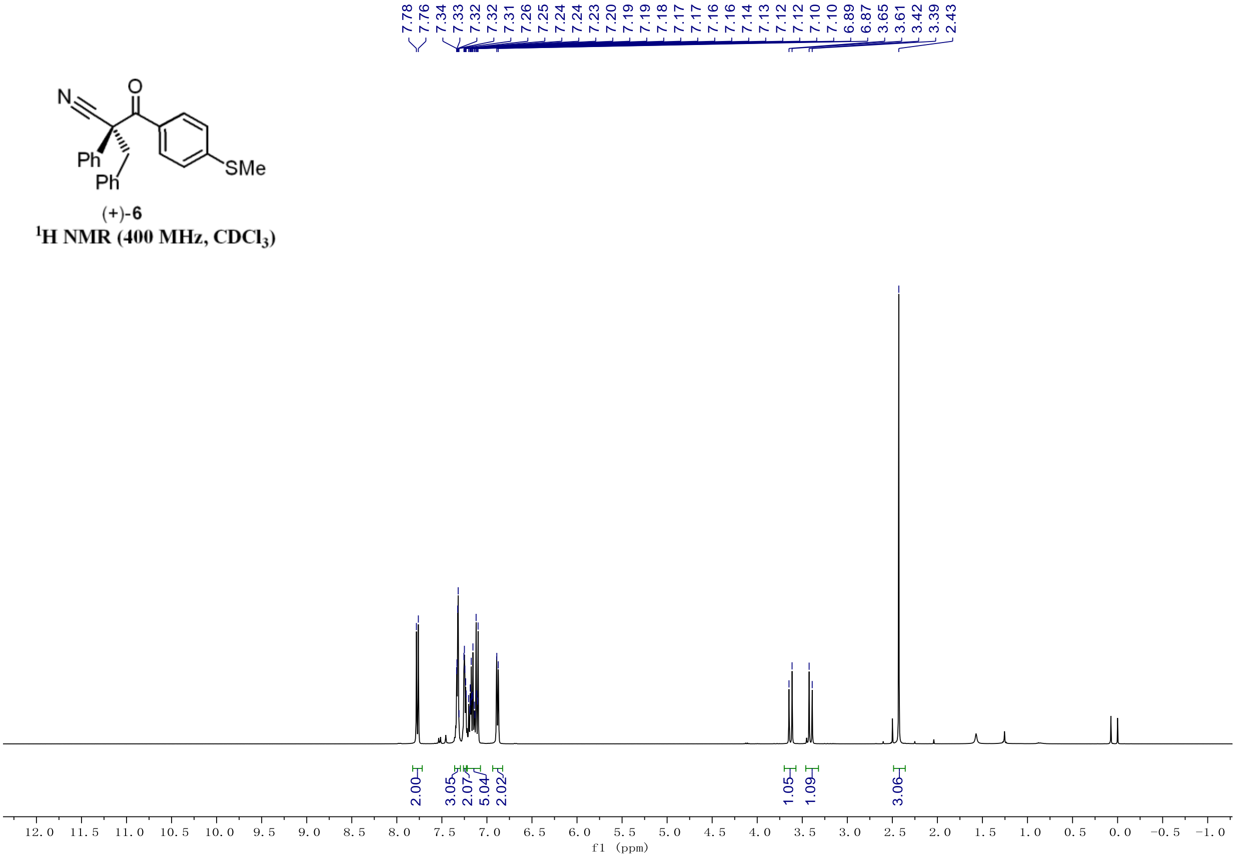

(+)-**6**

^{1}H NMR (400 MHz, $CDCl_3$)

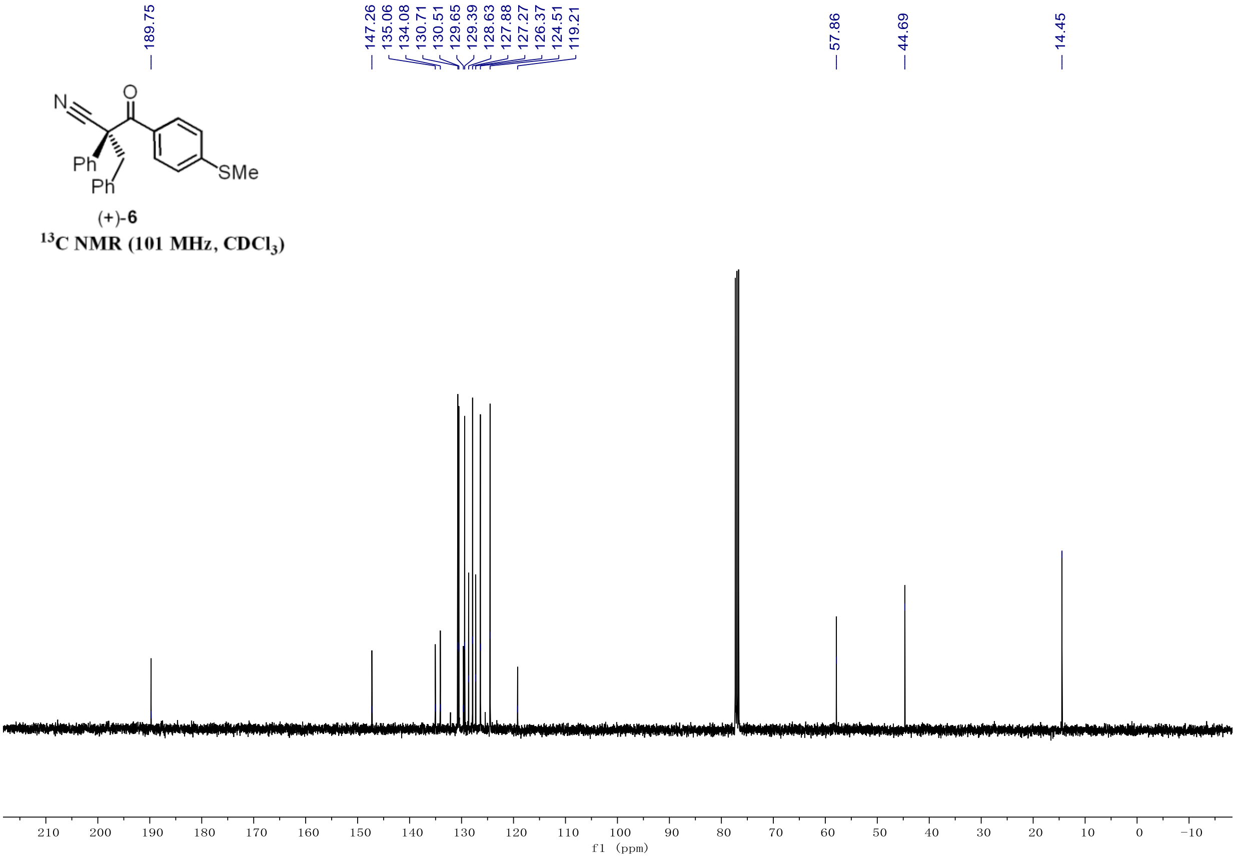

(+)-**6**

^{13}C NMR (101 MHz, $CDCl_3$)

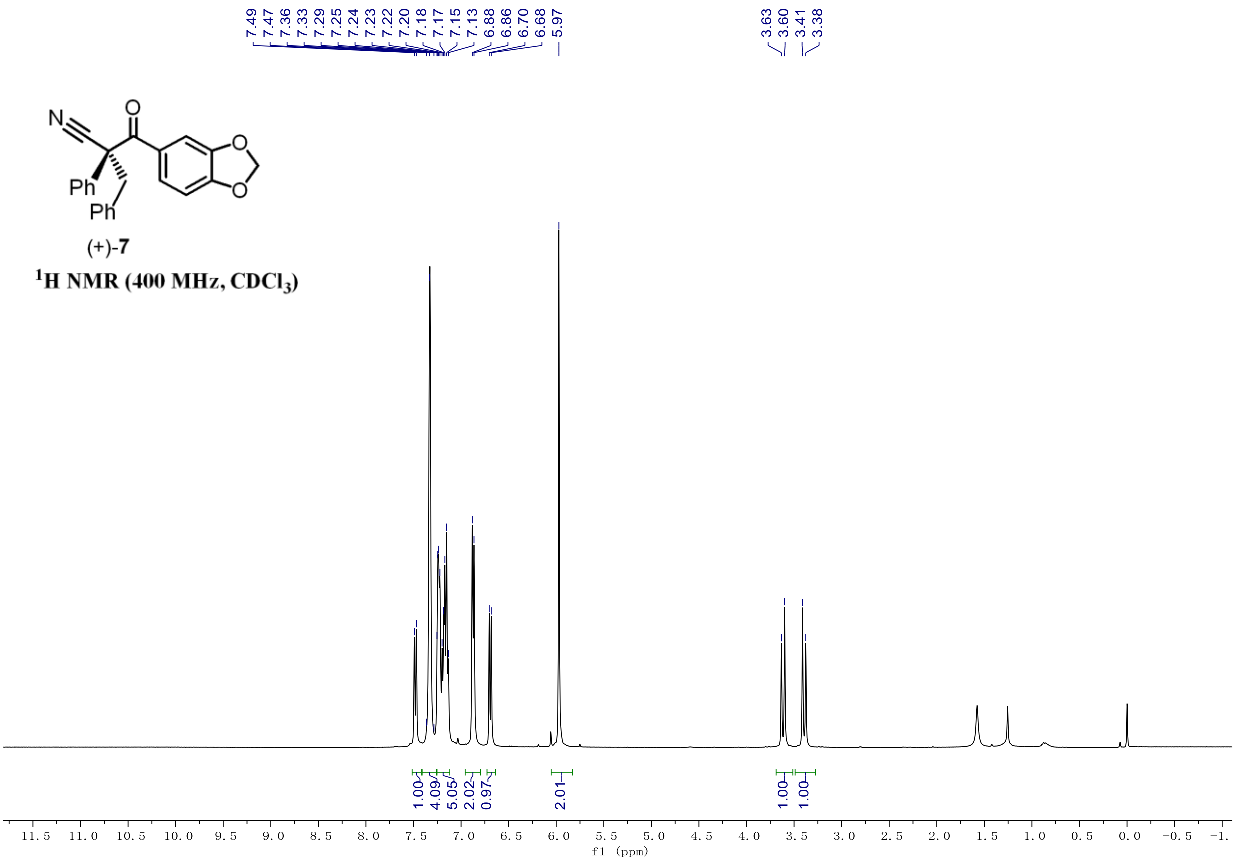
7.49 7.47 7.36 7.33 7.29 7.25 7.24 7.23 7.22 7.20 7.18 7.17 7.15 7.13 6.88 6.86 6.70 6.68 5.97
3.63 3.60 3.41 3.38
N
O
O
O
Ph
Ph
(+)-7
^{1}H NMR (400 MHz, CDCl$_3$)
1.00 4.09 5.05 2.02 0.97 2.01 1.00 1.00
f1 (ppm)

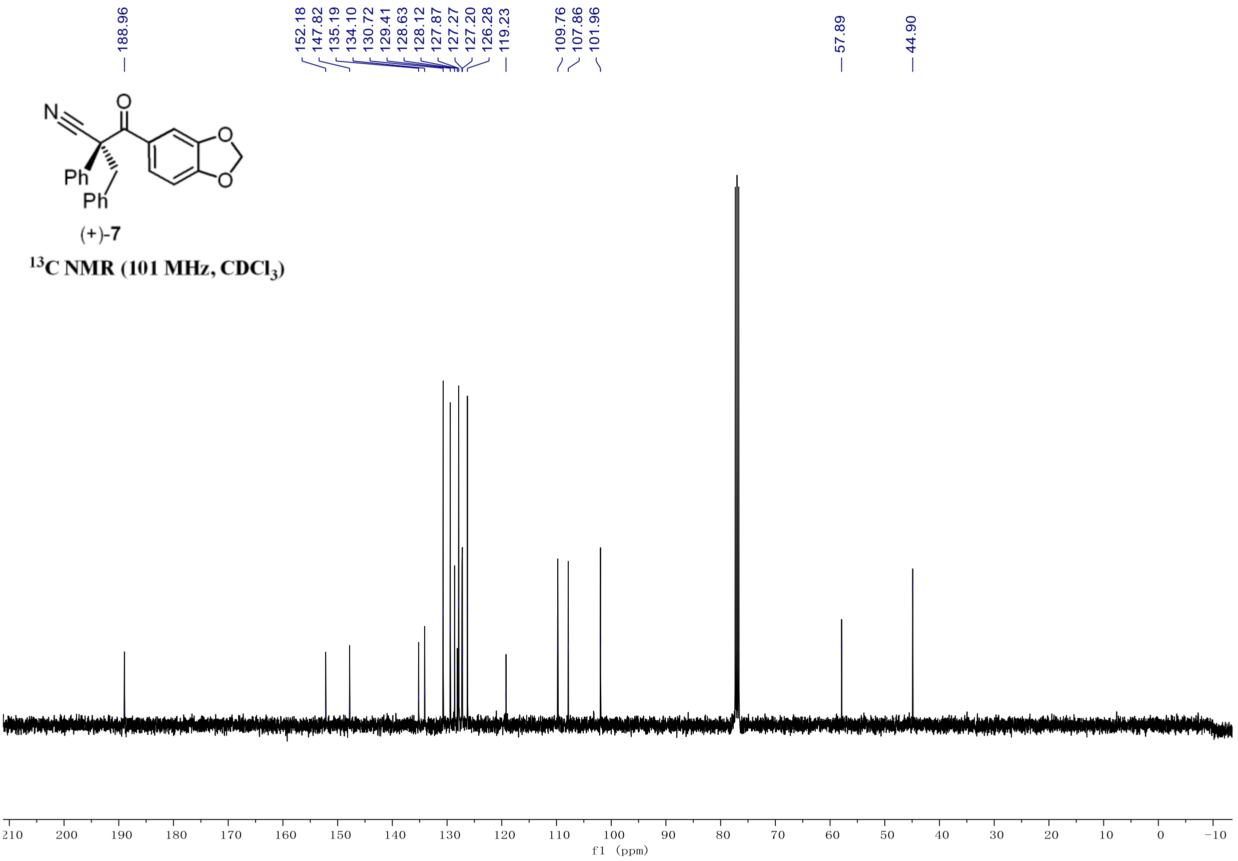
188.96
152.18 147.82 135.19 134.10 130.72 129.41 128.63 128.12 127.87 127.27 127.20 126.28 119.23
109.76 107.86 101.96
57.89
44.90
N
O
O
O
Ph
Ph
(+)-7
^{13}C NMR (101 MHz, CDCl$_3$)
f1 (ppm)

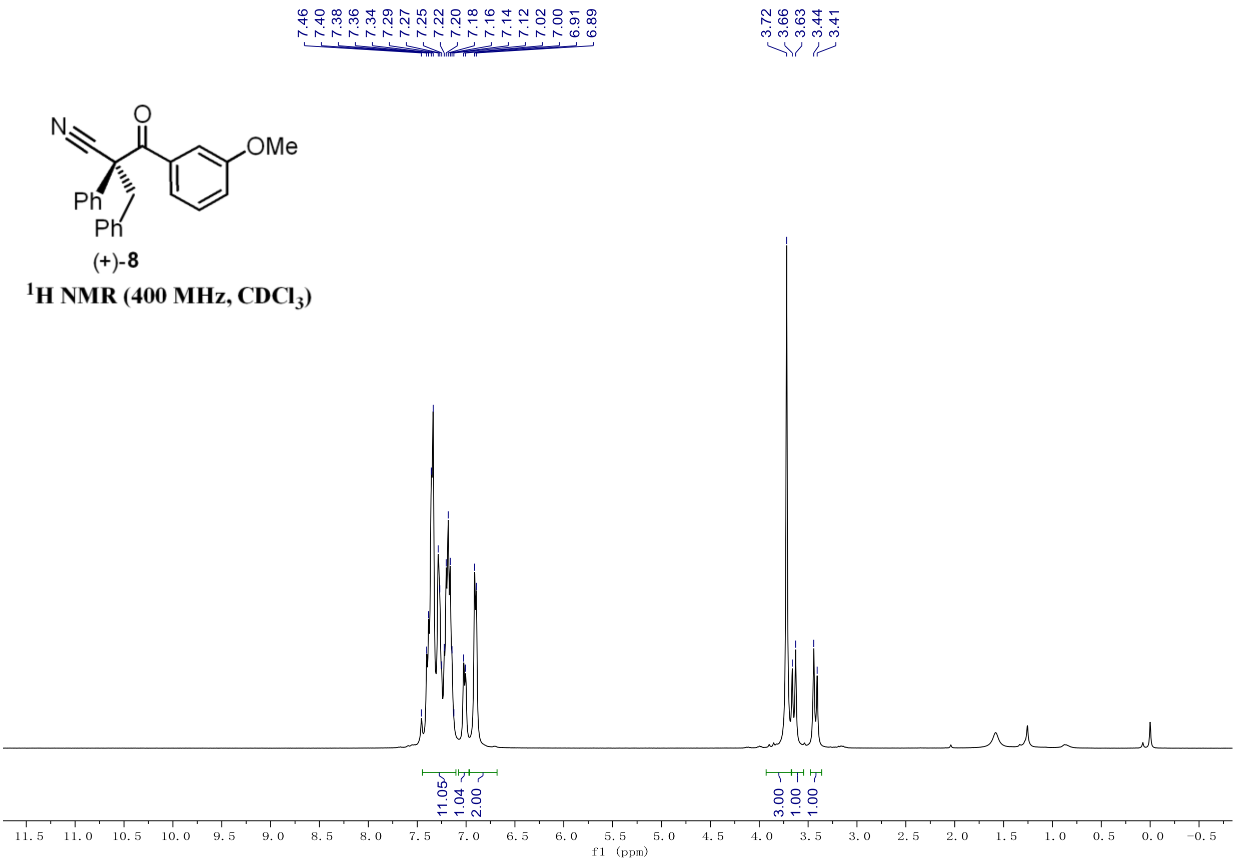
7.46 7.40 7.38 7.36 7.34 7.29 7.27 7.25 7.22 7.20 7.18 7.16 7.14 7.12 7.02 7.00 6.91 6.89
3.72 3.66 3.63 3.44 3.41
N
O
OMe
Ph
Ph
(+)-8
1H NMR (400 MHz, CDCl3)
11.05 1.04 2.00
3.00 1.00 1.00
f1 (ppm)

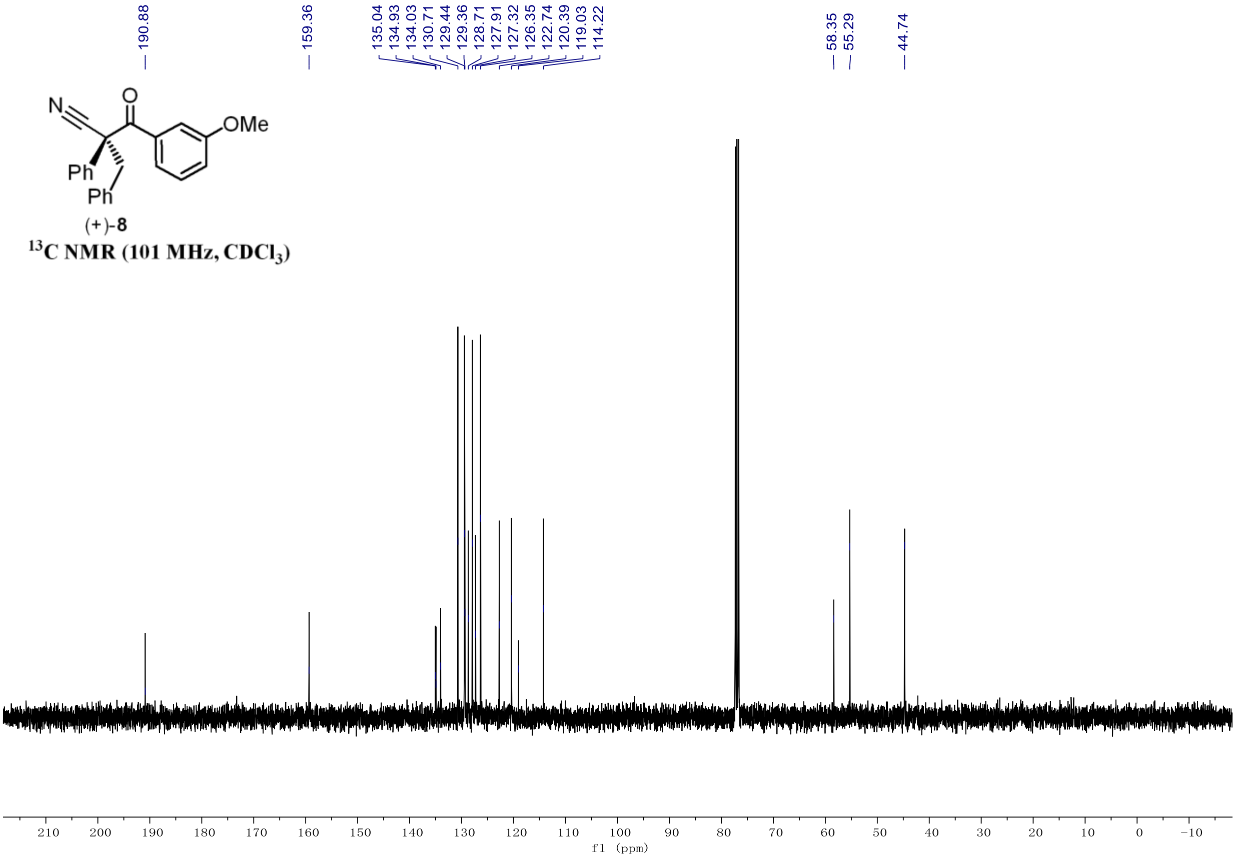
190.88
159.36
135.04 134.93 134.03 130.71 129.44 129.36 128.71 127.91 127.32 126.35 122.74 120.39 119.03 114.22
58.35 55.29
44.74
N
O
OMe
Ph
Ph
(+)-8
13C NMR (101 MHz, CDCl3)
f1 (ppm)

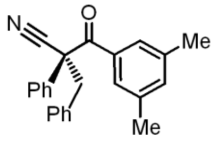

(+)-**9**

^{1}H NMR (400 MHz, CDCl$_3$)

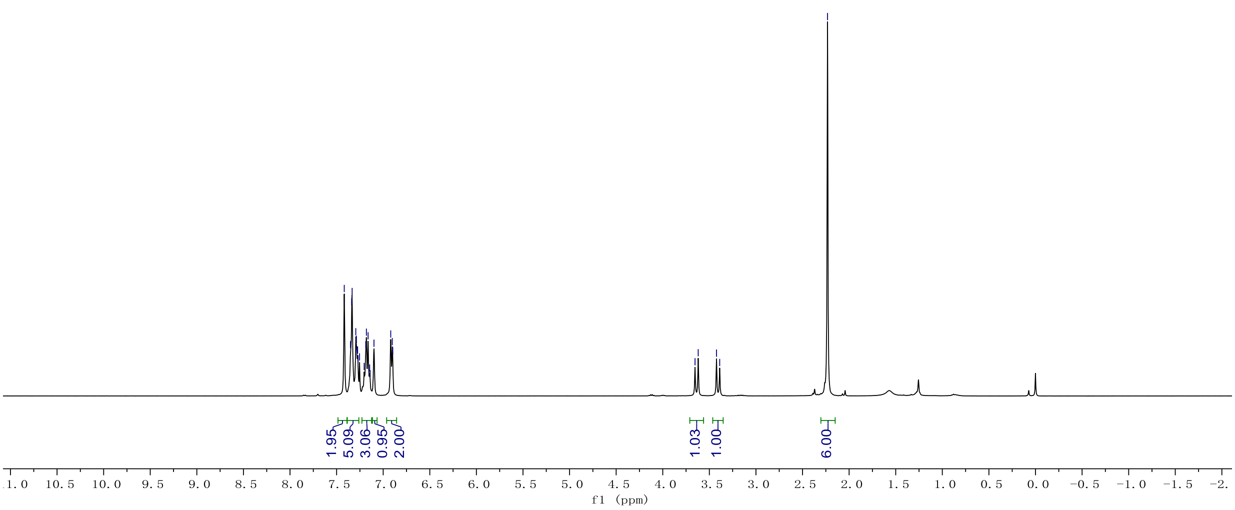

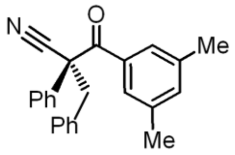

(+)-**9**

^{13}C NMR (101 MHz, CDCl$_3$)

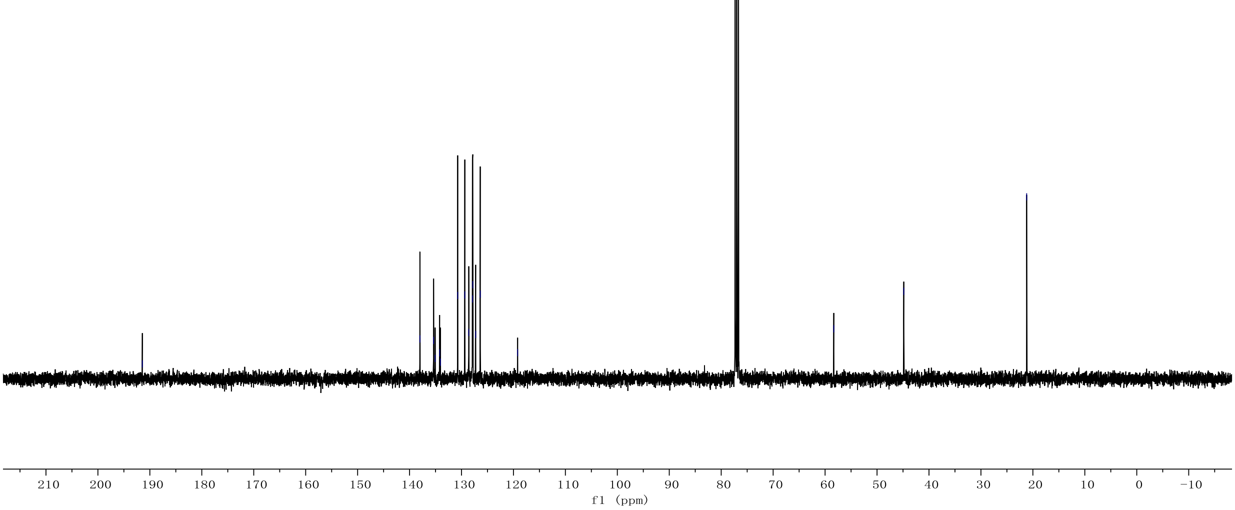

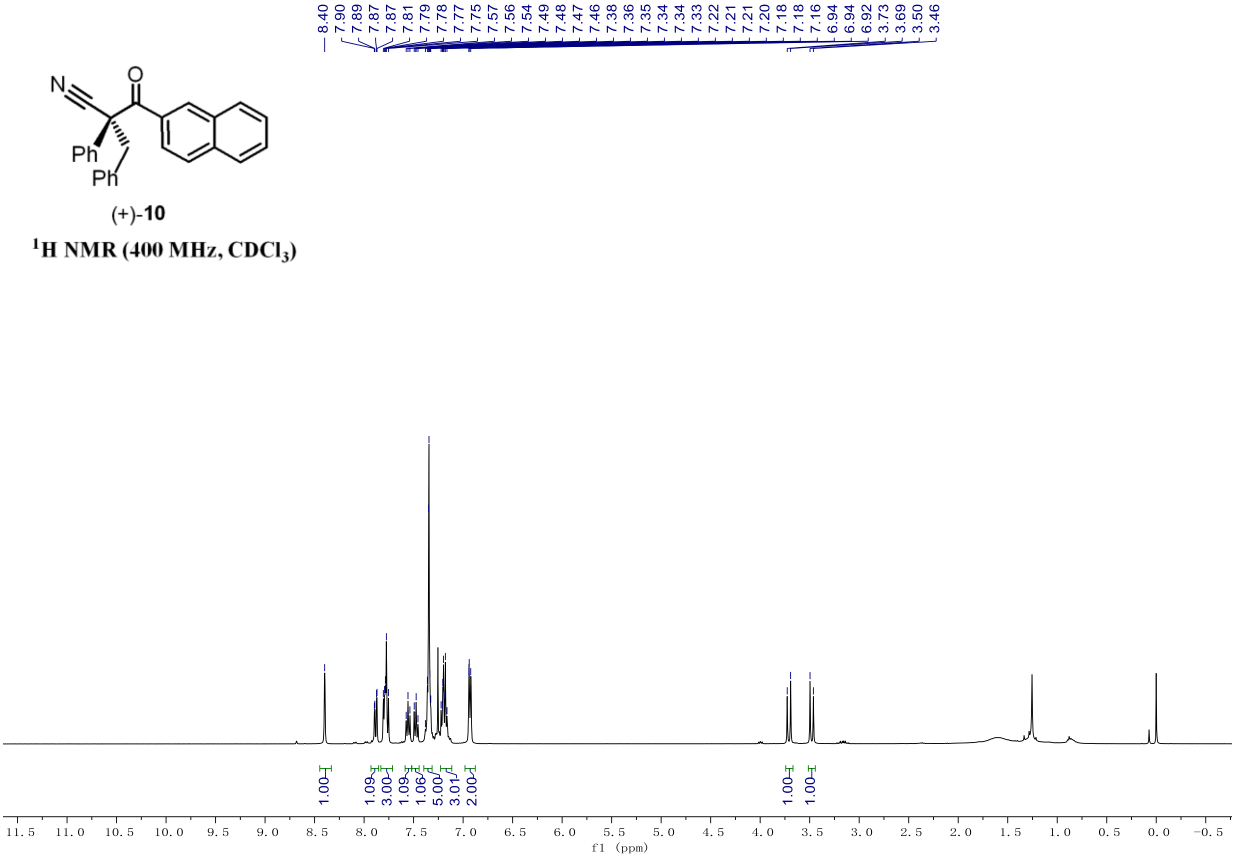

8.40 7.90 7.89 7.87 7.87 7.81 7.79 7.78 7.77 7.75 7.57 7.56 7.54 7.49 7.48 7.47 7.46 7.38 7.36 7.35 7.34 7.34 7.33 7.22 7.21 7.21 7.20 7.18 7.16 6.94 6.94 6.92 3.73 3.69 3.50 3.46
(+)-10
^{1}H NMR (400 MHz, CDCl$_3$)
1.00 1.09 3.00 1.09 1.06 5.00 3.01 2.00 1.00 1.00
f1 (ppm)

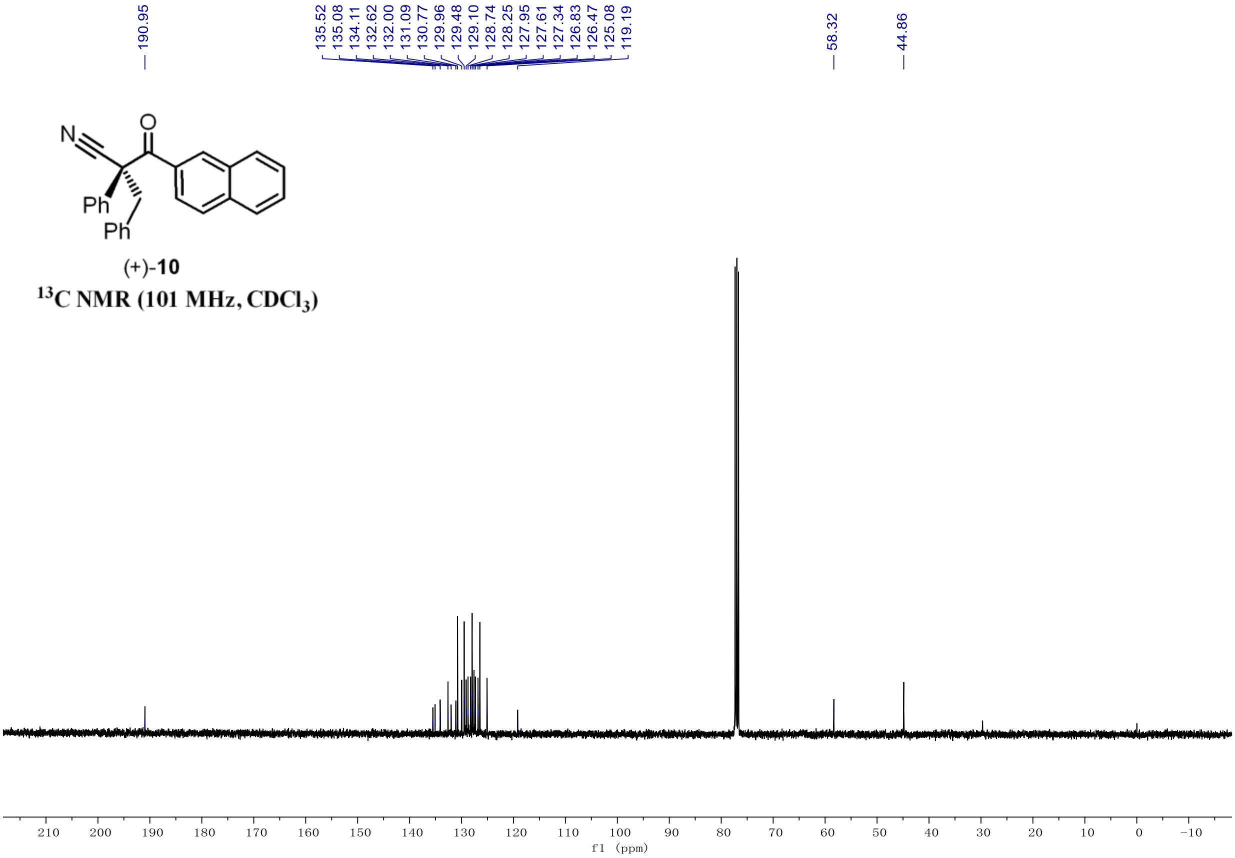

190.95
135.52 135.08 134.11 132.62 132.00 131.09 130.77 129.96 129.48 129.10 128.74 128.25 127.95 127.61 127.34 126.83 126.47 125.08 119.19
58.32
44.86
(+)-10
^{13}C NMR (101 MHz, CDCl$_3$)
f1 (ppm)

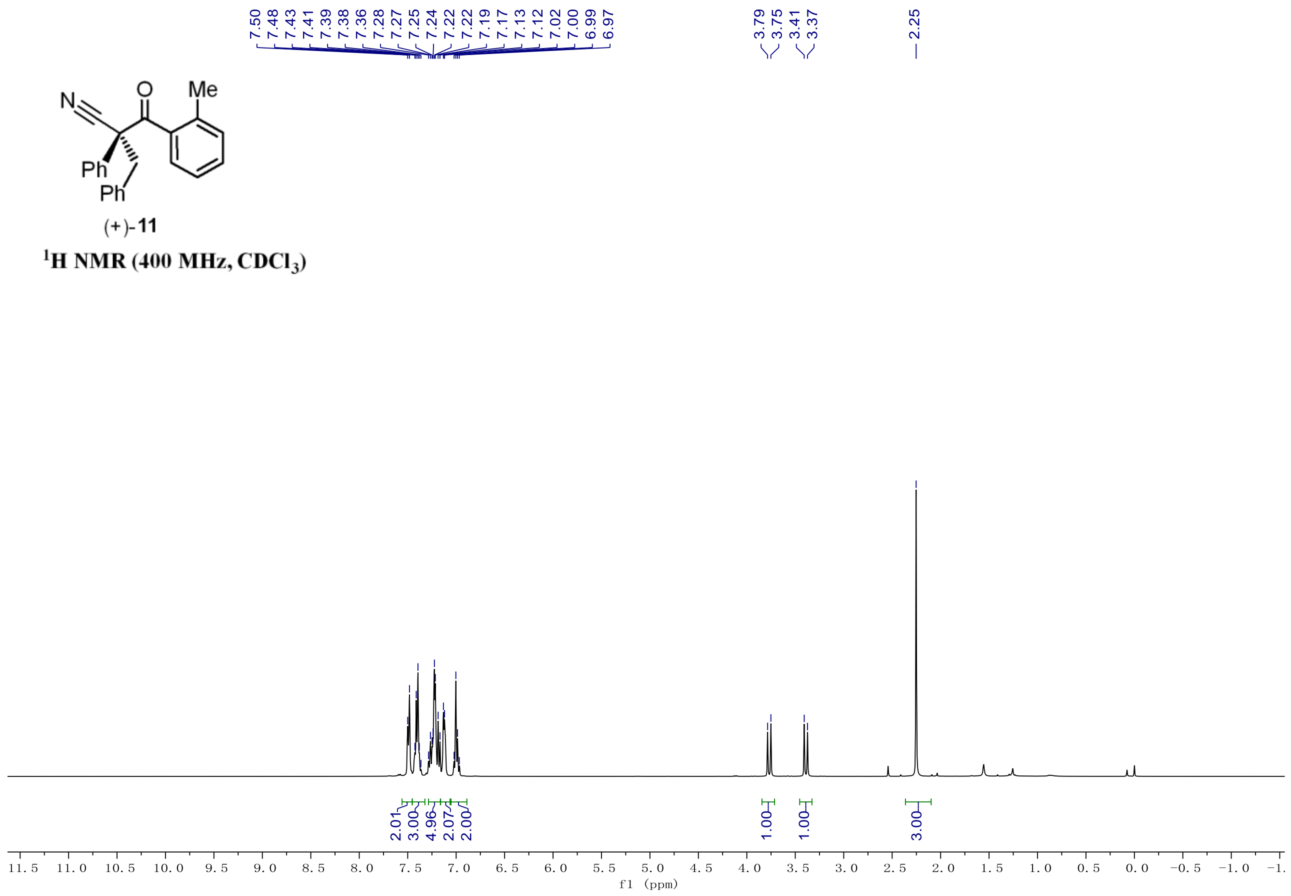
N
O
Me
Ph
Ph
(+)-11
¹H NMR (400 MHz, CDCl₃)
7.50
7.48
7.43
7.41
7.39
7.38
7.36
7.28
7.27
7.25
7.24
7.22
7.22
7.19
7.17
7.13
7.12
7.02
7.00
6.99
6.97
3.79
3.75
3.41
3.37
2.25
2.01
3.00
4.96
2.07
2.00
1.00
1.00
3.00
f1 (ppm)

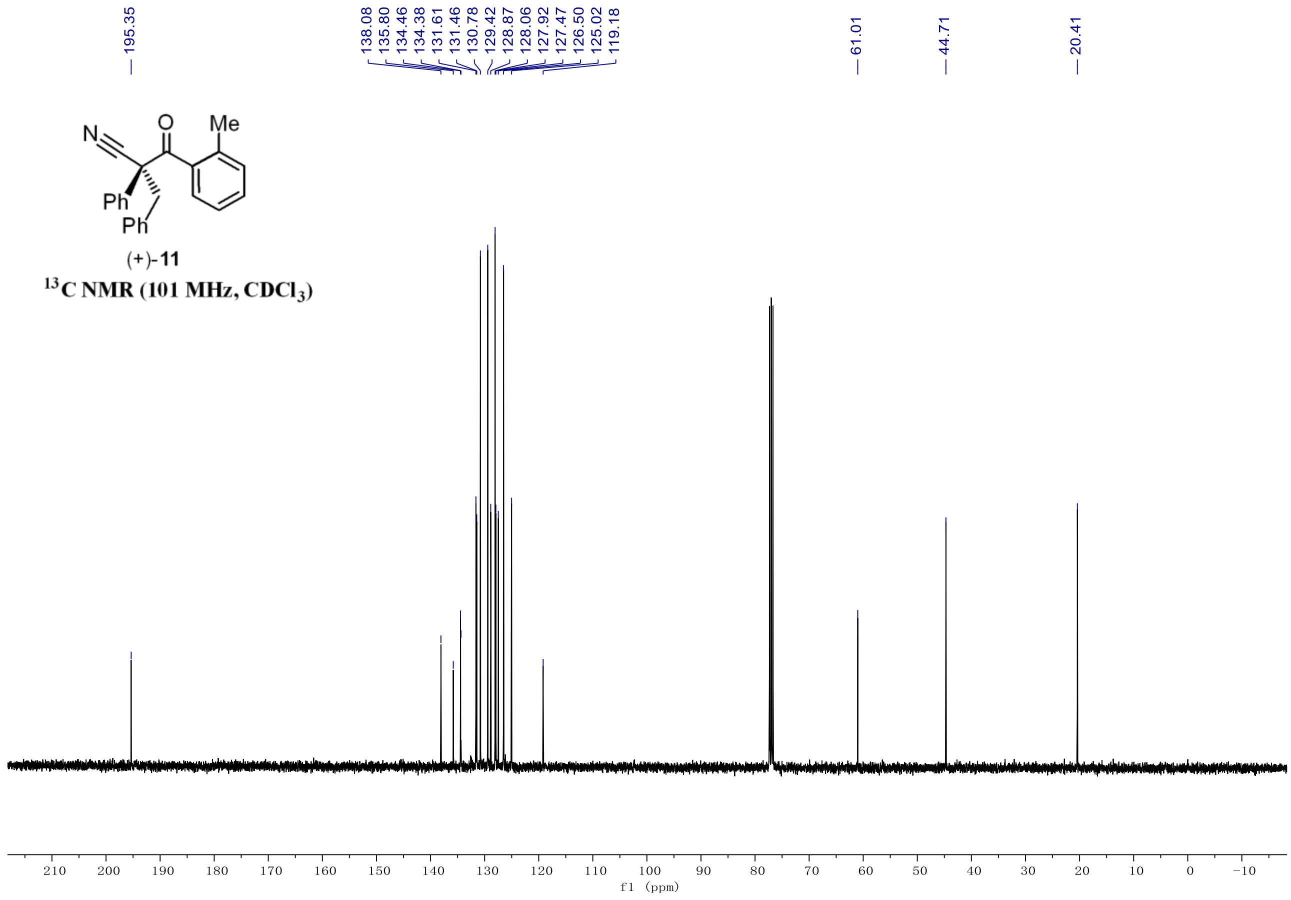
N
O
Me
Ph
Ph
(+)-11
¹³C NMR (101 MHz, CDCl₃)
195.35
138.08
135.80
134.46
134.38
131.61
131.46
130.78
129.42
128.87
128.06
127.92
127.47
126.50
125.02
119.18
61.01
44.71
20.41
f1 (ppm)

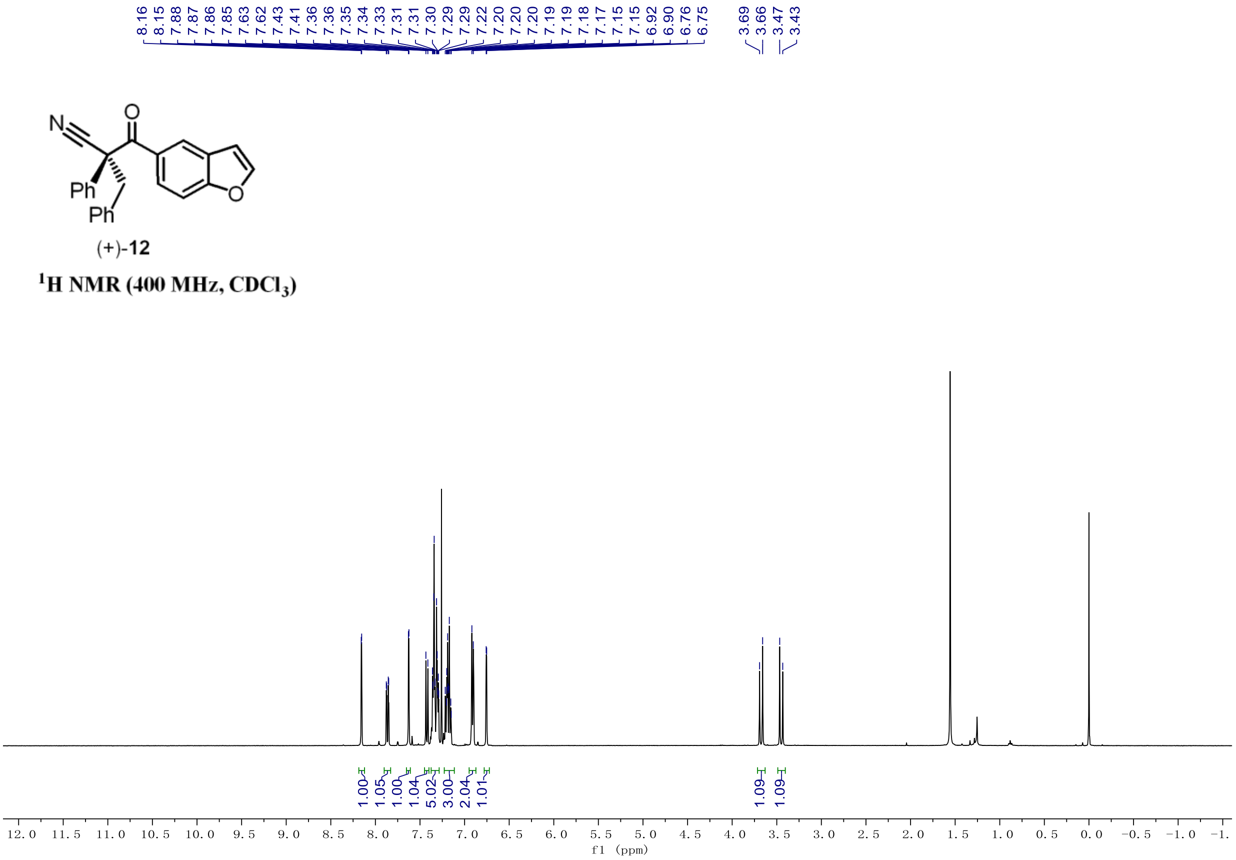
8.16 8.15 7.88 7.87 7.86 7.85 7.63 7.62 7.43 7.41 7.36 7.35 7.34 7.33 7.31 7.31 7.30 7.29 7.29 7.22 7.20 7.20 7.20 7.19 7.19 7.18 7.17 7.15 7.15 6.92 6.90 6.76 6.75
3.69 3.66 3.47 3.43
N
O
Ph
Ph
O
(+)-12
1H NMR (400 MHz, CDCl3)
1.00 1.05 1.00 1.04 5.02 3.00 2.04 1.01
1.09 1.09
12.0 11.5 11.0 10.5 10.0 9.5 9.0 8.5 8.0 7.5 7.0 6.5 6.0 5.5 5.0 4.5 4.0 3.5 3.0 2.5 2.0 1.5 1.0 0.5 0.0 -0.5 -1.0 -1.
f1 (ppm)

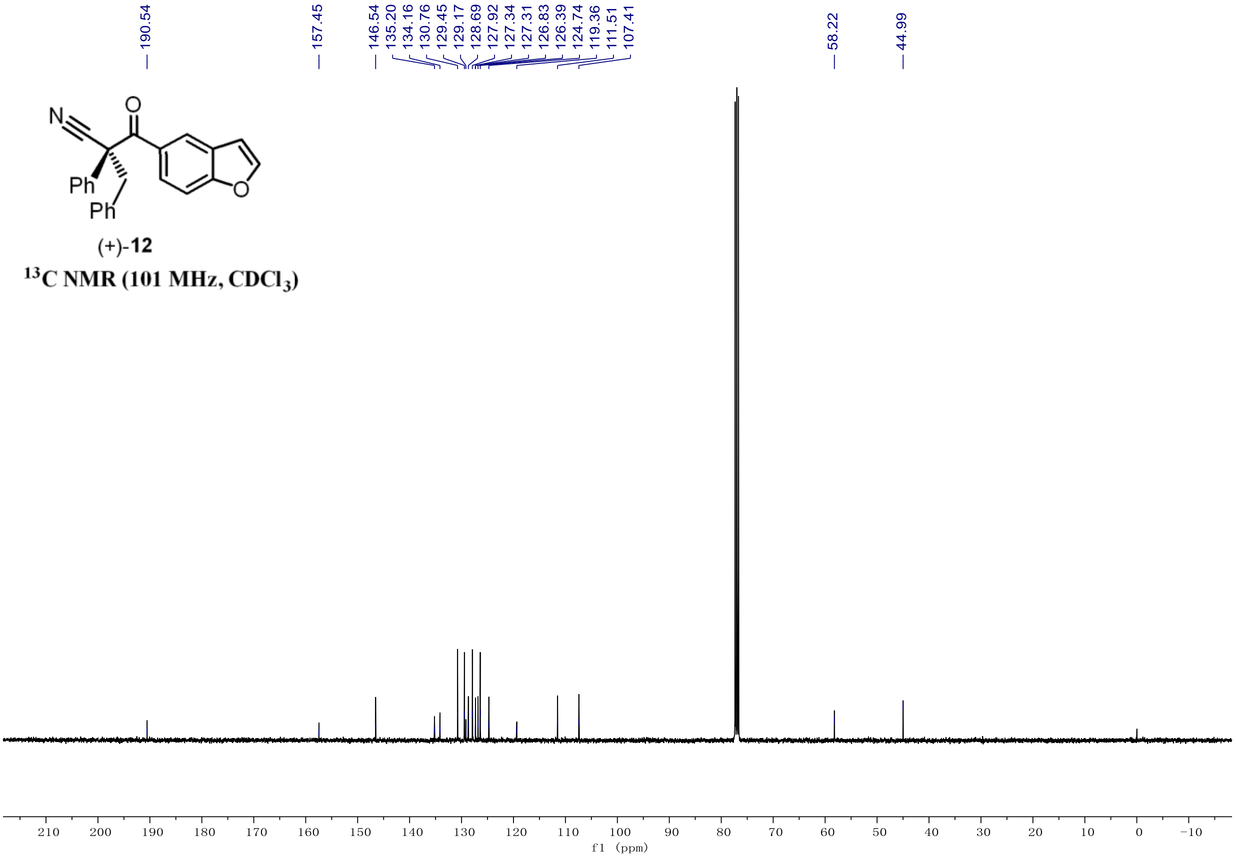
190.54
157.45
146.54 135.20 134.16 130.76 129.45 129.17 128.69 127.92 127.34 127.31 126.83 126.39 124.74 119.36 111.51 107.41
58.22
44.99
N
O
Ph
Ph
O
(+)-12
13C NMR (101 MHz, CDCl3)
210 200 190 180 170 160 150 140 130 120 110 100 90 80 70 60 50 40 30 20 10 0 -10
f1 (ppm)

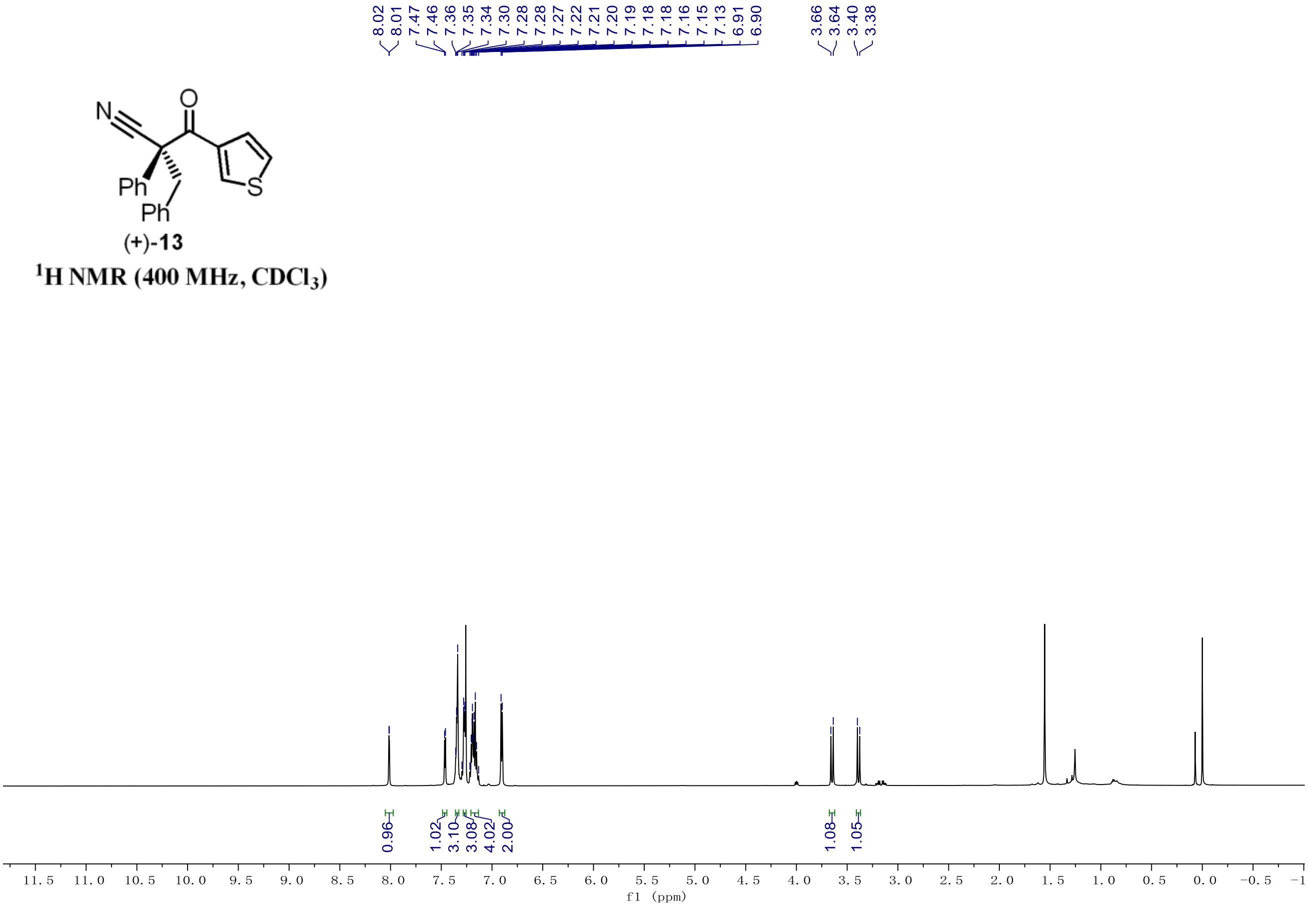

8.02
8.01
7.47
7.46
7.36
7.35
7.34
7.30
7.28
7.27
7.22
7.21
7.20
7.19
7.18
7.18
7.16
7.15
7.13
6.91
6.90
3.66
3.64
3.40
3.38
(+)-13
^{1}H NMR (400 MHz, $CDCl_3$)
0.96
1.02
3.10
3.08
4.02
2.00
1.08
1.05
f1 (ppm)

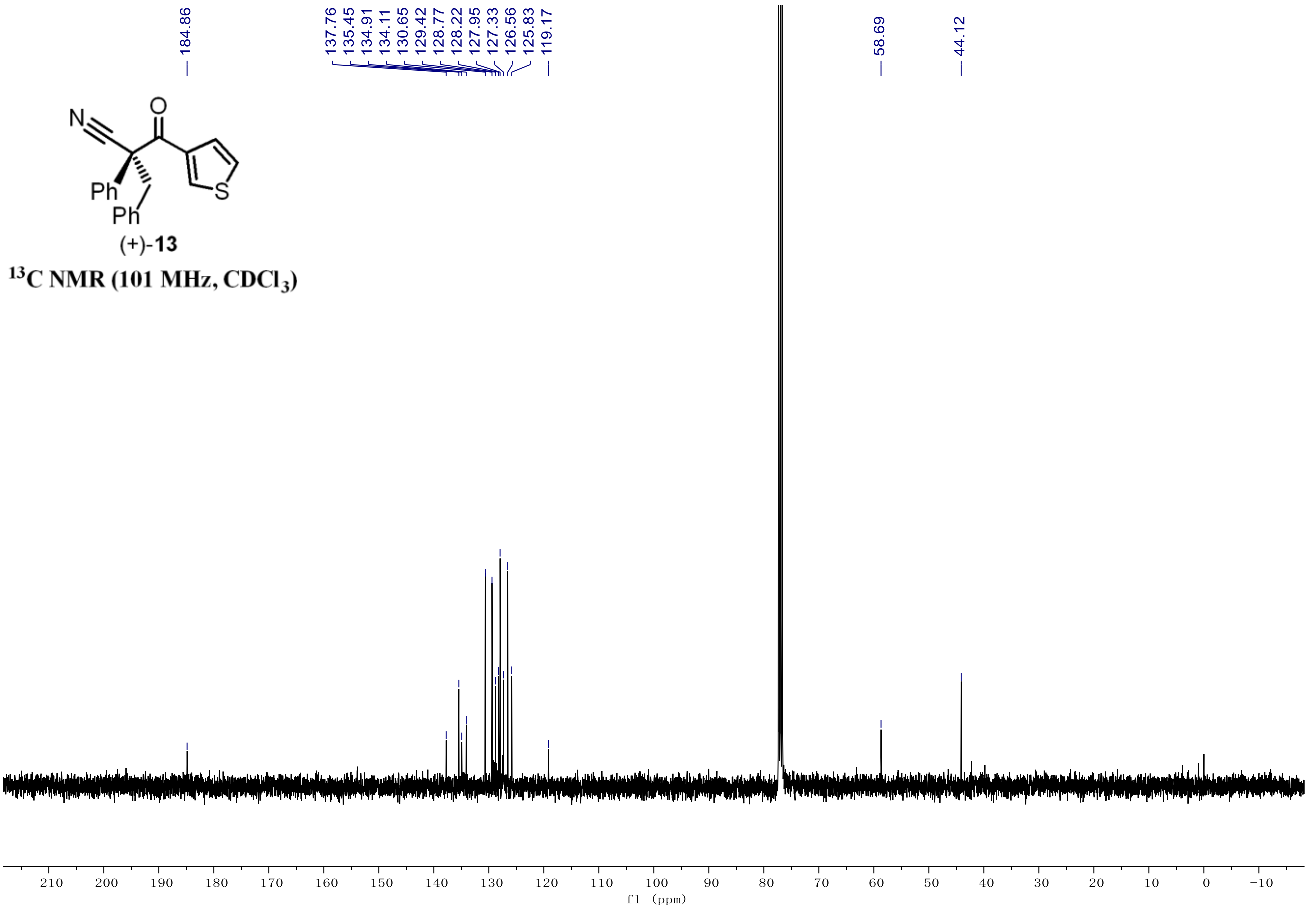

184.86
137.76
135.45
134.91
134.11
130.65
129.42
128.77
128.22
127.95
127.33
126.56
125.83
119.17
58.69
44.12
(+)-13
^{13}C NMR (101 MHz, $CDCl_3$)
f1 (ppm)

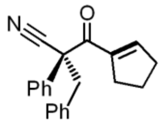

(+)-**14**

^{1}H NMR (400 MHz, $CDCl_3$)

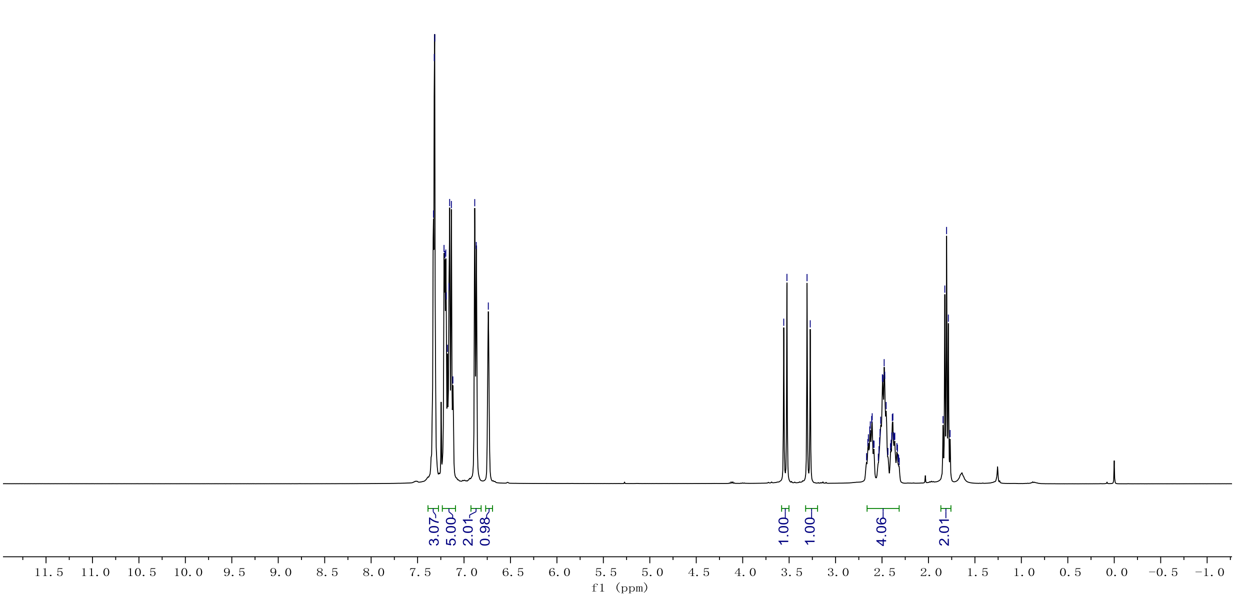

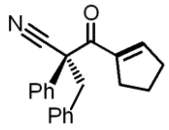

(+)-**14**

^{13}C NMR (101 MHz, $CDCl_3$)

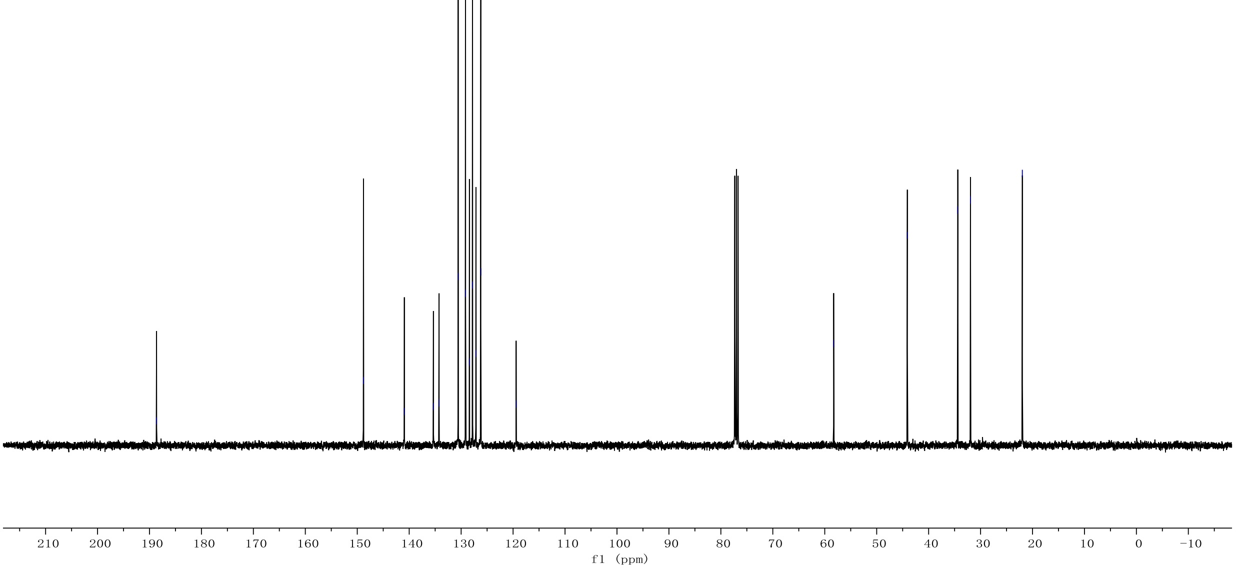

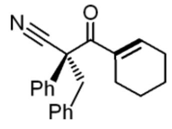

(+)-**15**

^{1}H NMR (400 MHz, $CDCl_3$)

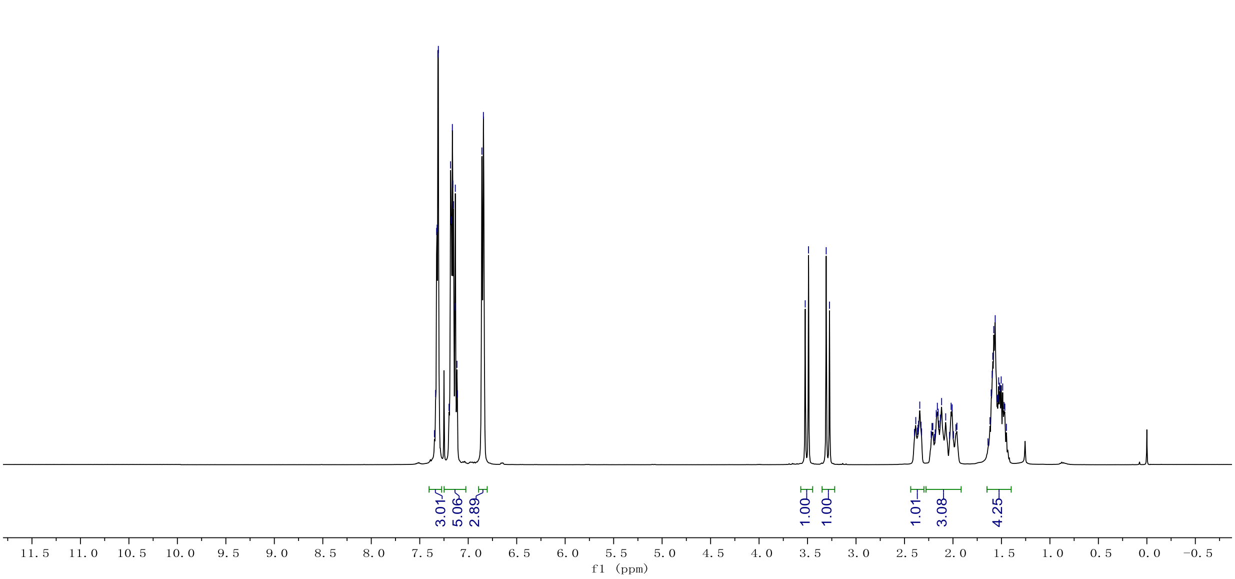

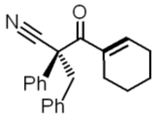

(+)-**15**

^{13}C NMR (101 MHz, $CDCl_3$)

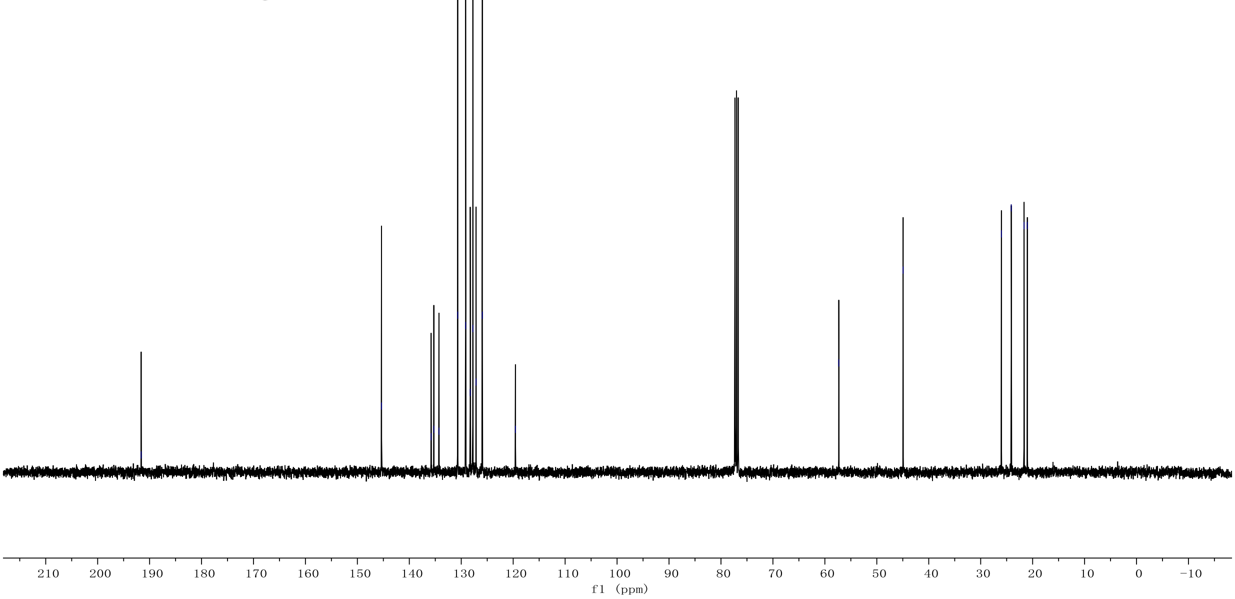

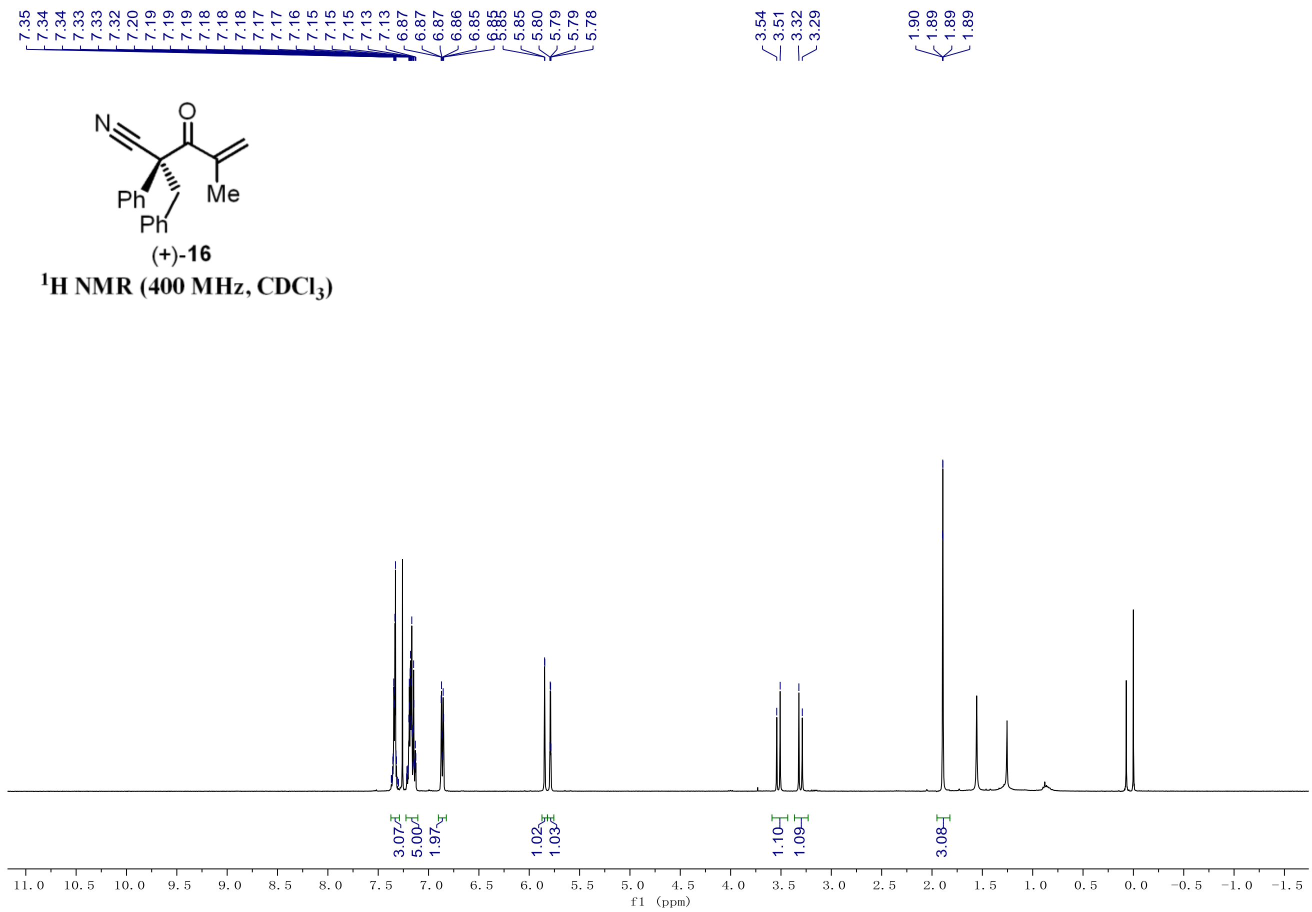

(+)-**16**

^{1}H NMR (400 MHz, $CDCl_3$)

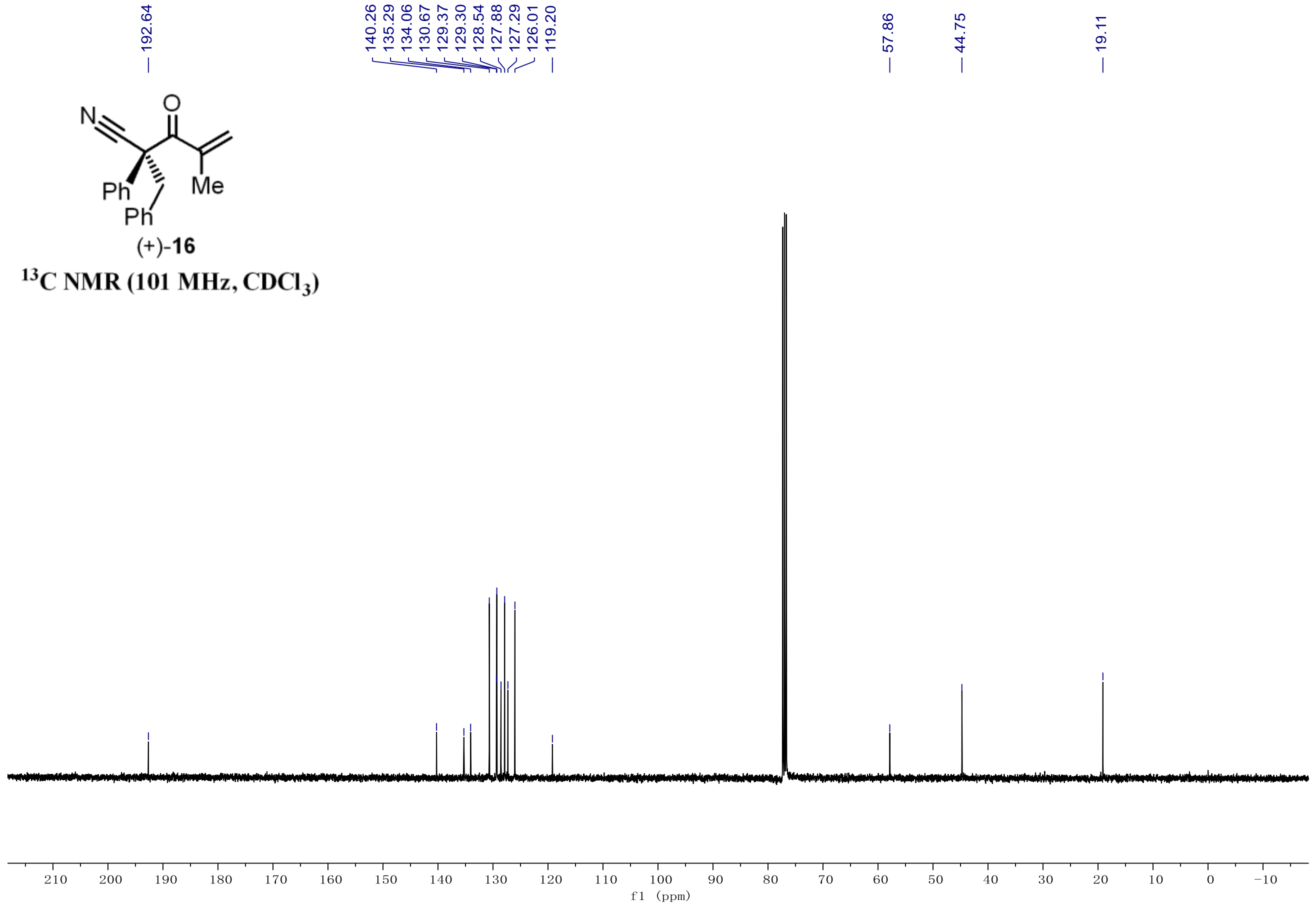

(+)-**16**

^{13}C NMR (101 MHz, $CDCl_3$)

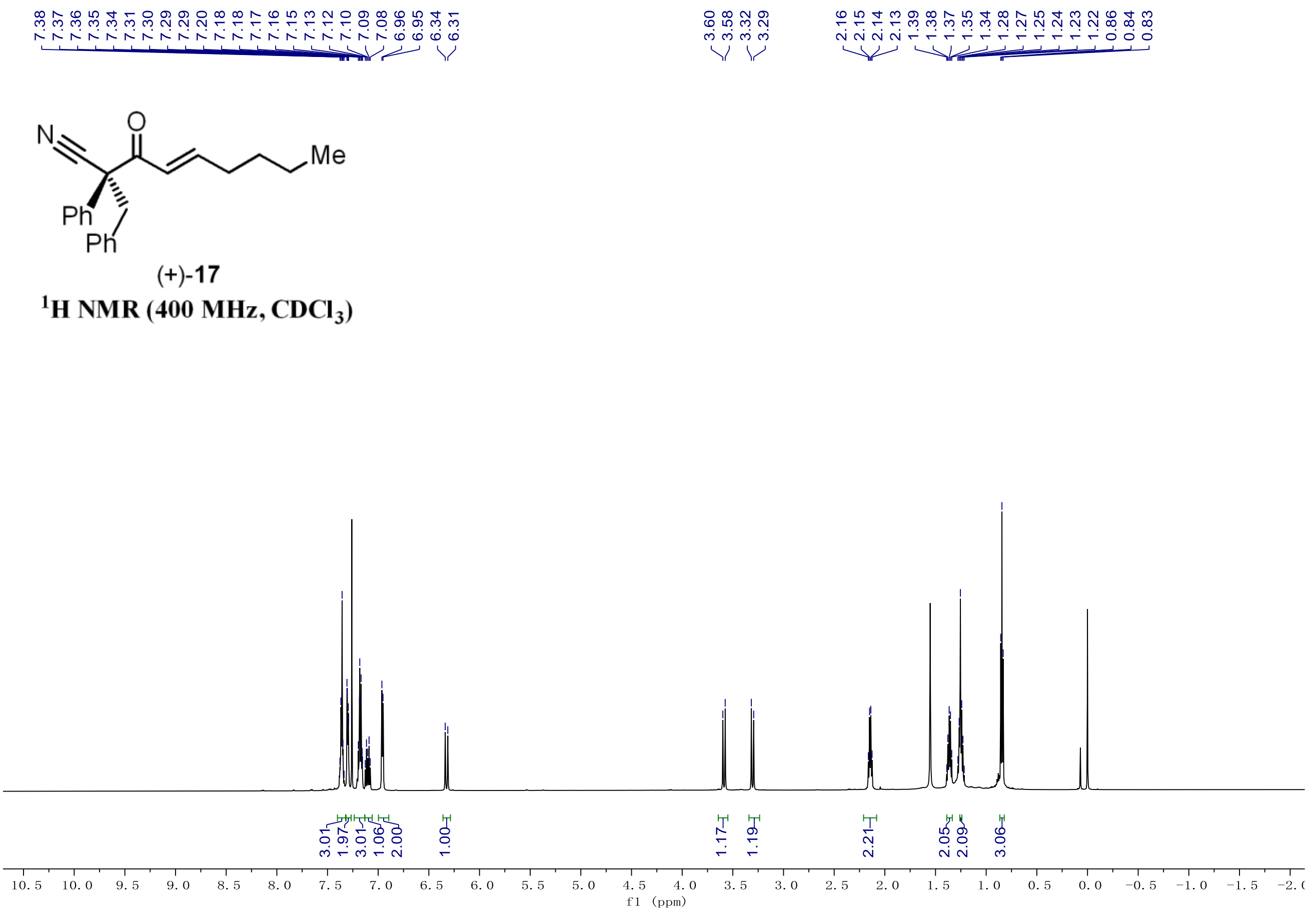
7.38 7.37 7.36 7.35 7.34 7.31 7.30 7.29 7.29 7.20 7.18 7.18 7.17 7.16 7.15 7.13 7.12 7.10 7.09 7.08 6.96 6.95 6.34 6.31
3.60 3.58 3.32 3.29
2.16 2.15 2.14 2.13 1.39 1.38 1.37 1.35 1.34 1.28 1.27 1.25 1.24 1.23 1.22 0.86 0.84 0.83
N
O
Me
Ph
Ph
(+)-17
^{1}H NMR (400 MHz, CDCl$_3$)
3.01 1.97 3.01 1.06 2.00 1.00 1.17 1.19 2.21 2.05 2.09 3.06
f1 (ppm)

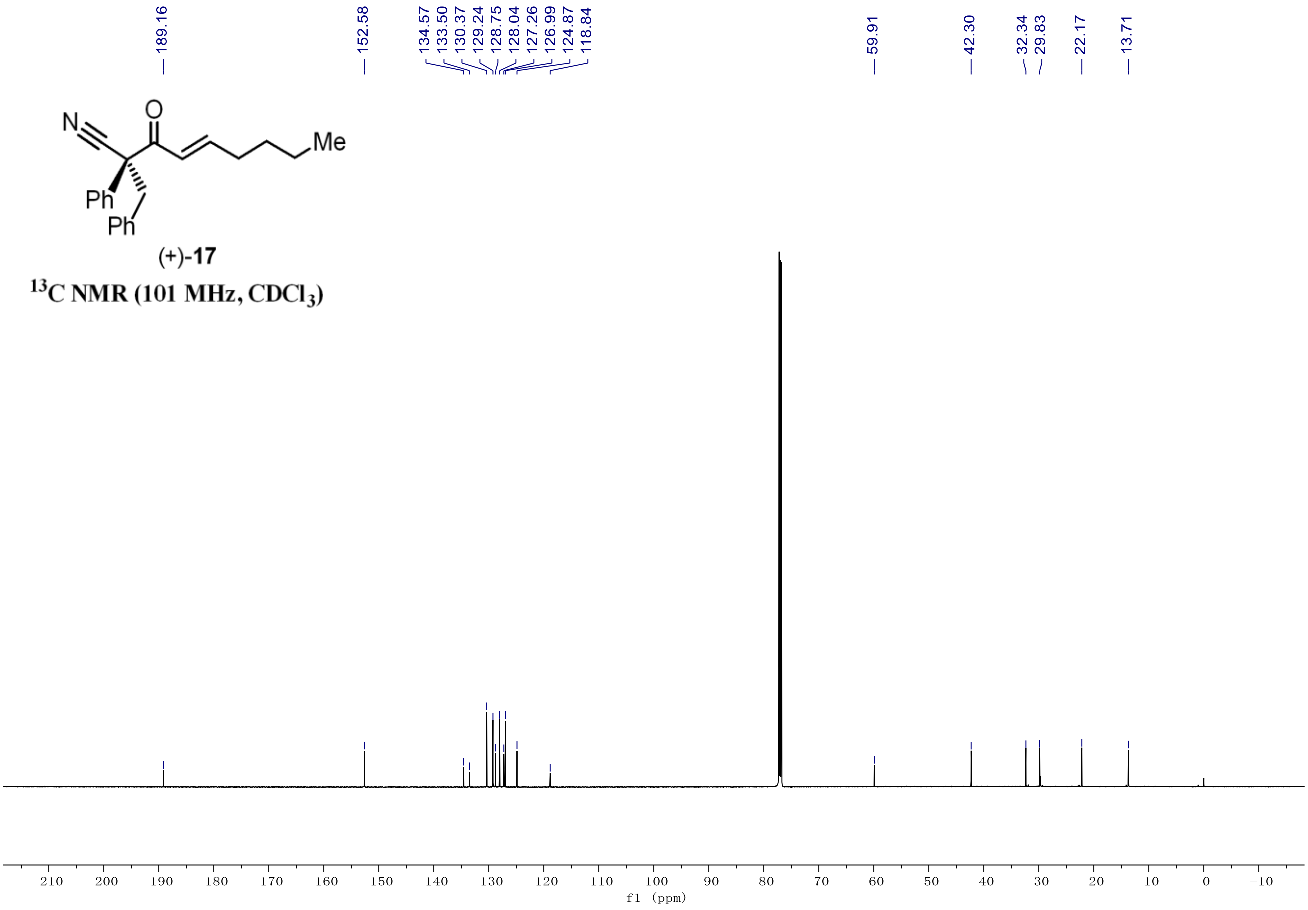
189.16
152.58
134.57 133.50 130.37 129.24 128.75 128.04 127.26 126.99 124.87 118.84
59.91
42.30
32.34 29.83
22.17
13.71
N
O
Me
Ph
Ph
(+)-17
^{13}C NMR (101 MHz, CDCl$_3$)
f1 (ppm)

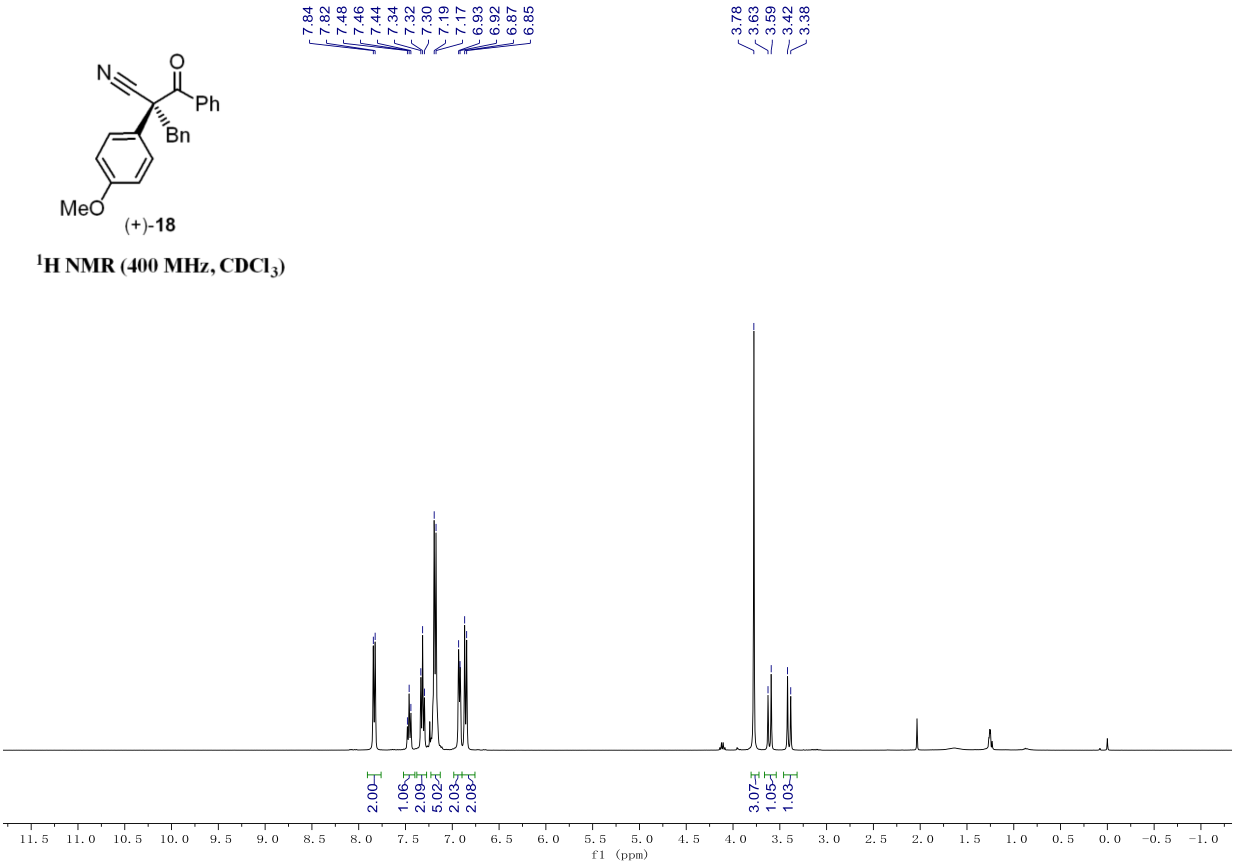

(+)-**18**

^{1}H NMR (400 MHz, $CDCl_3$)

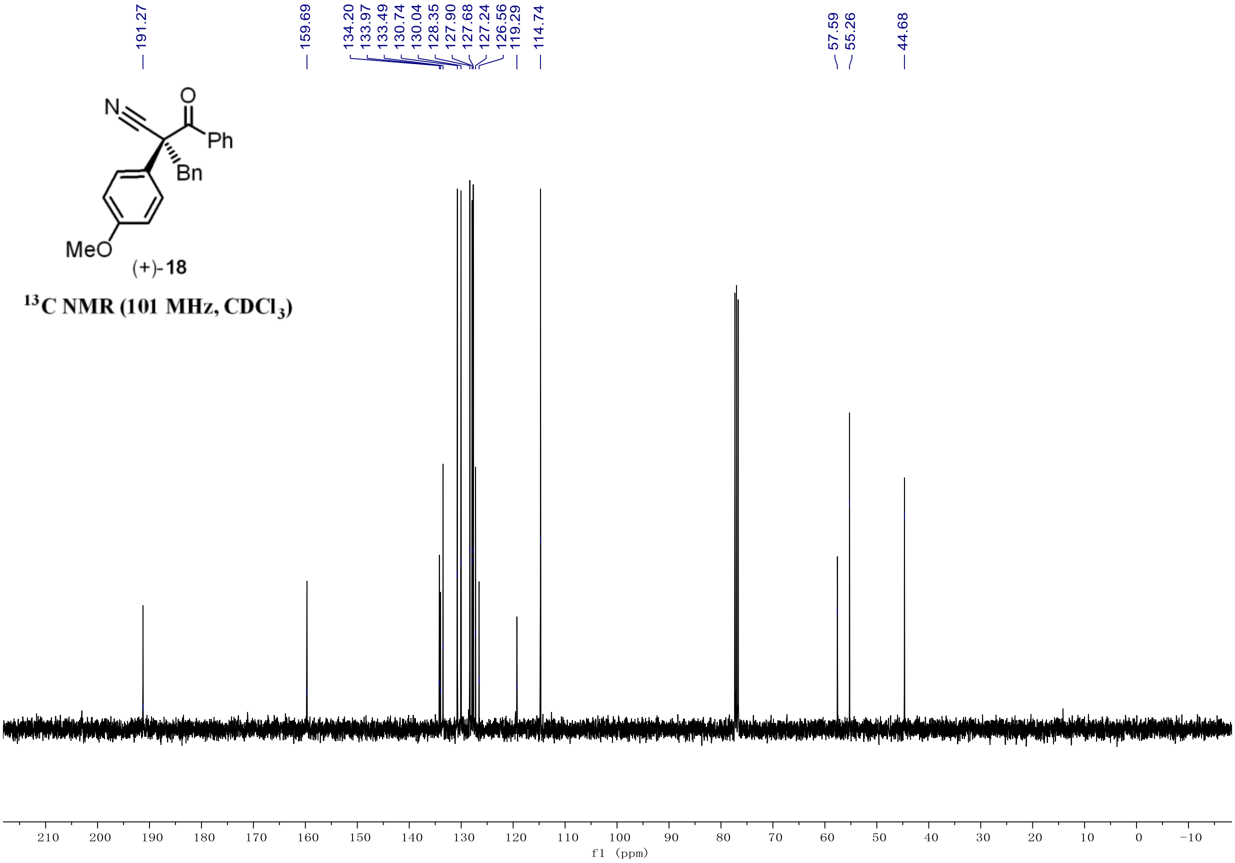

(+)-**18**

^{13}C NMR (101 MHz, $CDCl_3$)

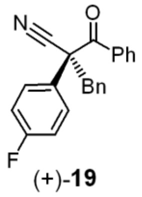

(+)-**19**

^{1}**H NMR (400 MHz, CDCl$_3$)**

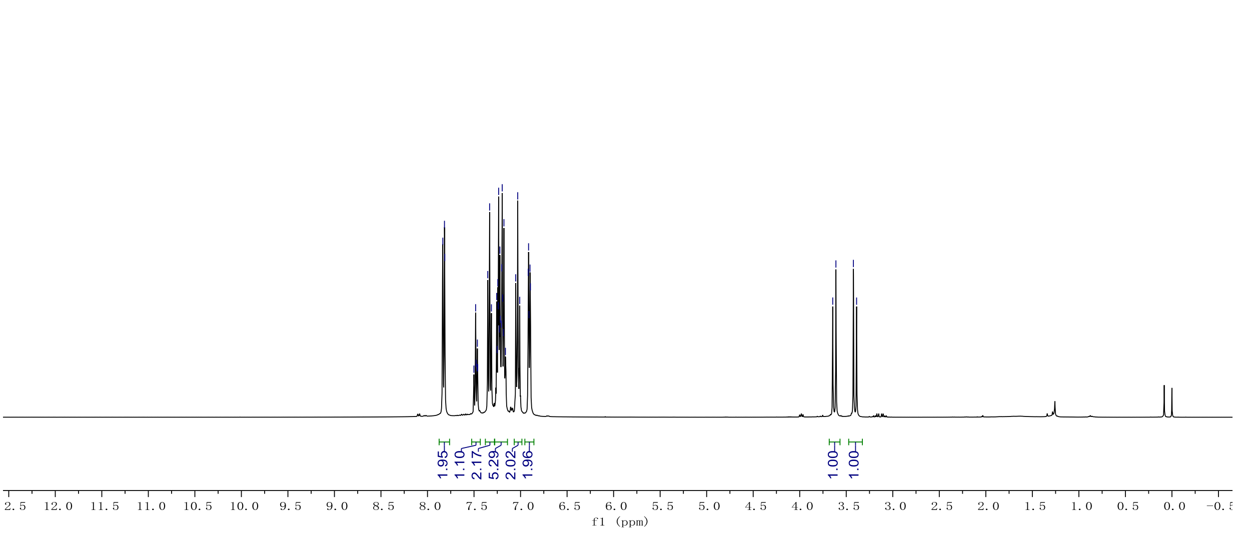

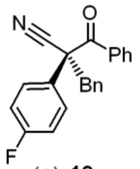

(+)-**19**

^{13}**C NMR (101 MHz, CDCl$_3$)**

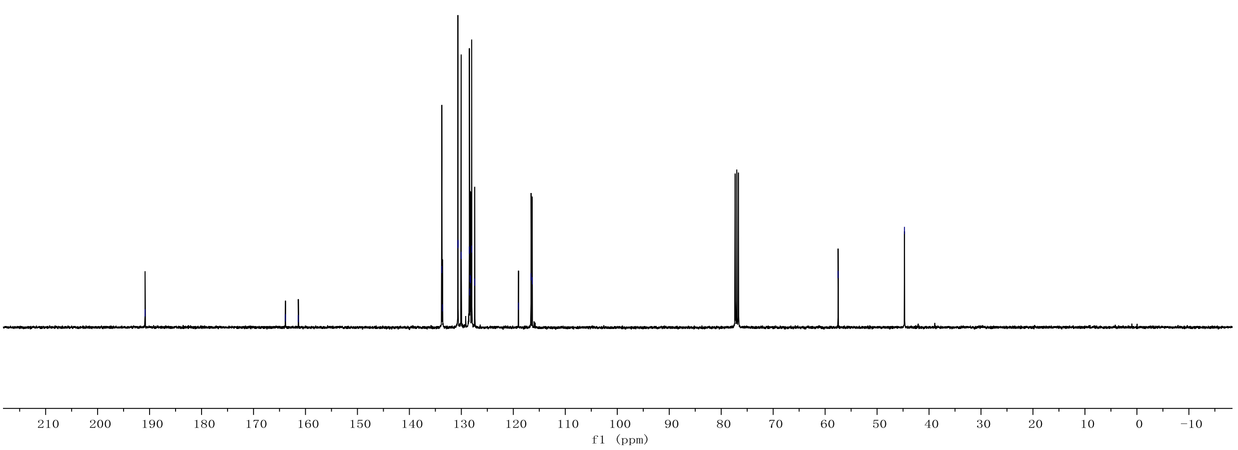

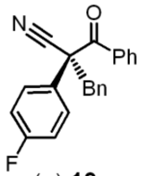

(+)-**19**

^{19}F NMR (376 MHz, CDCl$_3$)

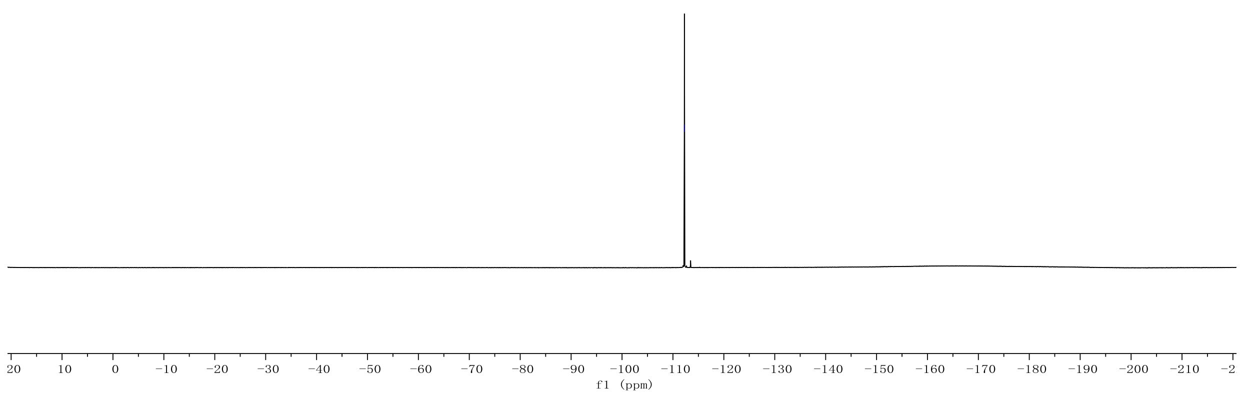

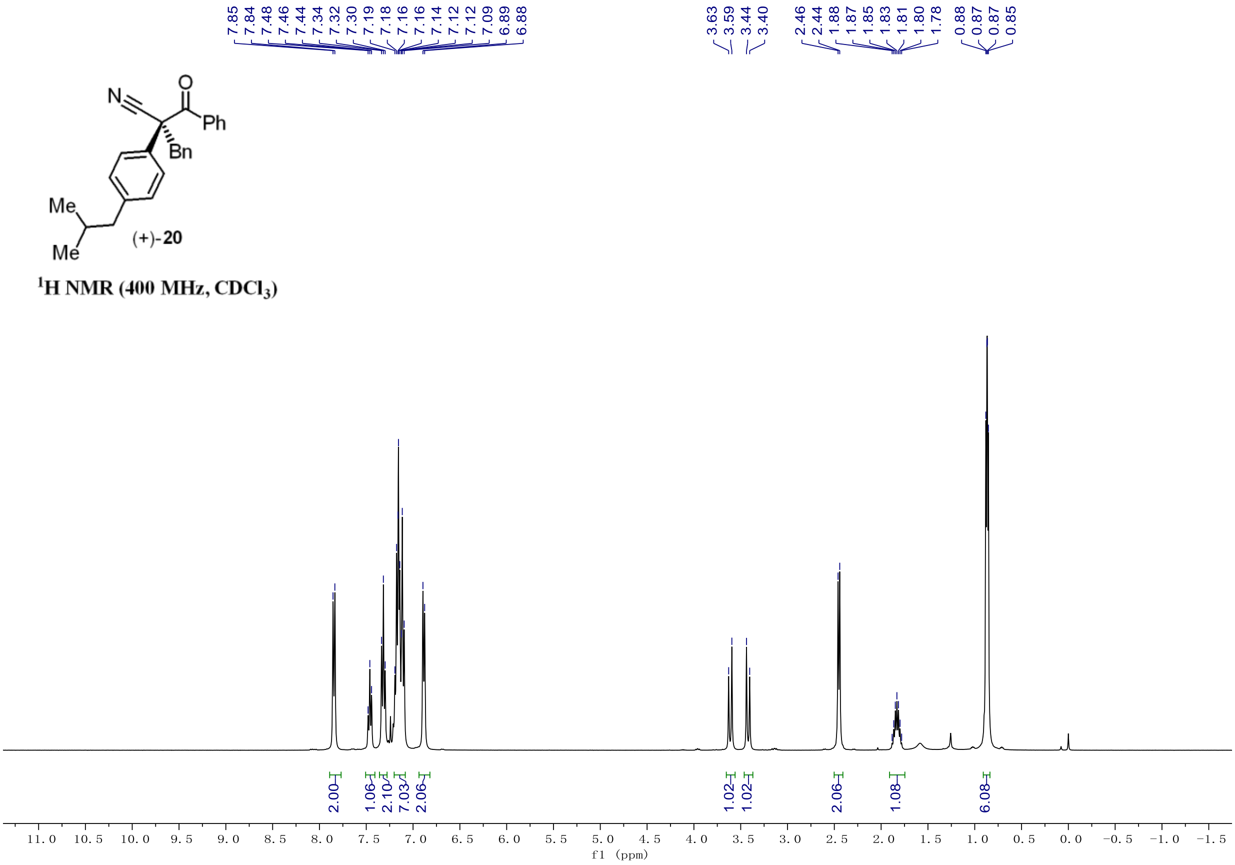

(+)-**20**

^{1}H NMR (400 MHz, $CDCl_3$)

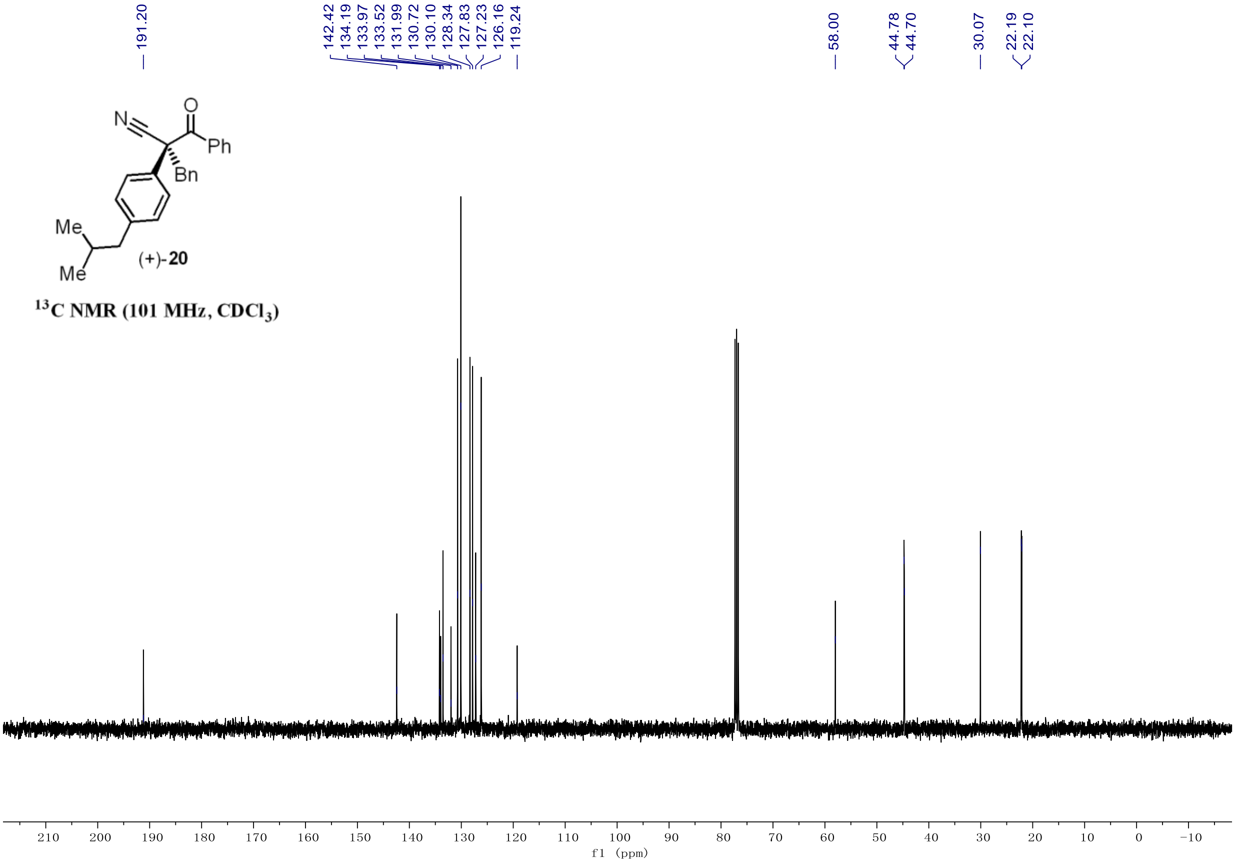

(+)-**20**

^{13}C NMR (101 MHz, $CDCl_3$)

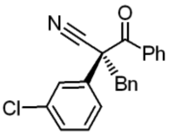

(+)-**21**

^{1}H NMR (400 MHz, $CDCl_3$)

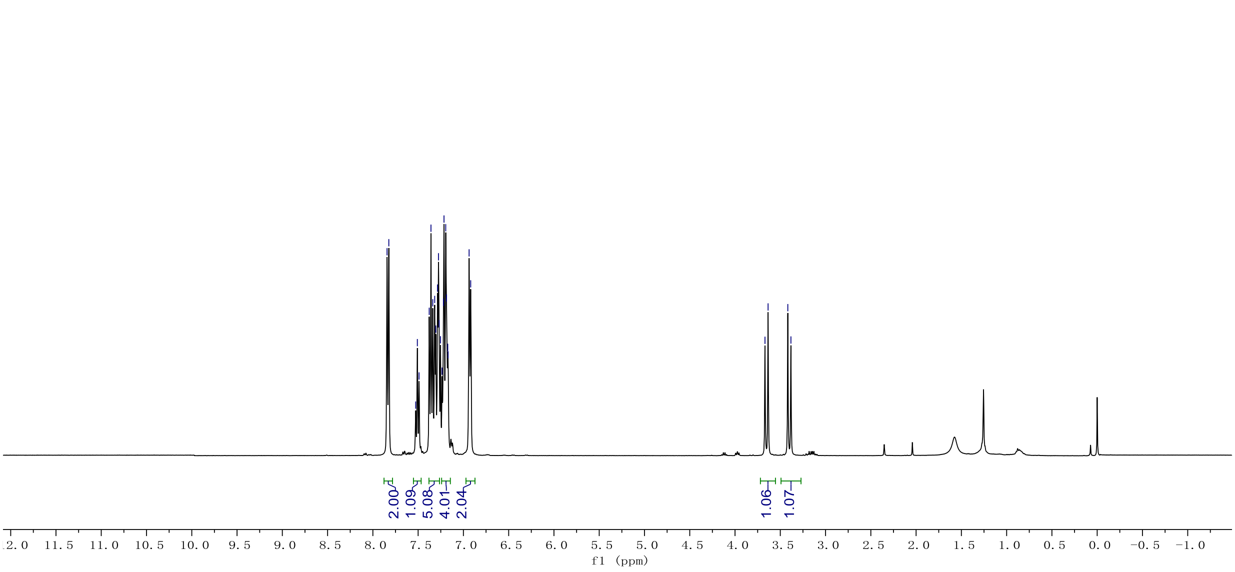

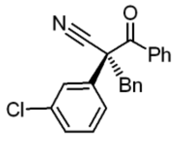

(+)-**21**

^{13}C NMR (101 MHz, $CDCl_3$)

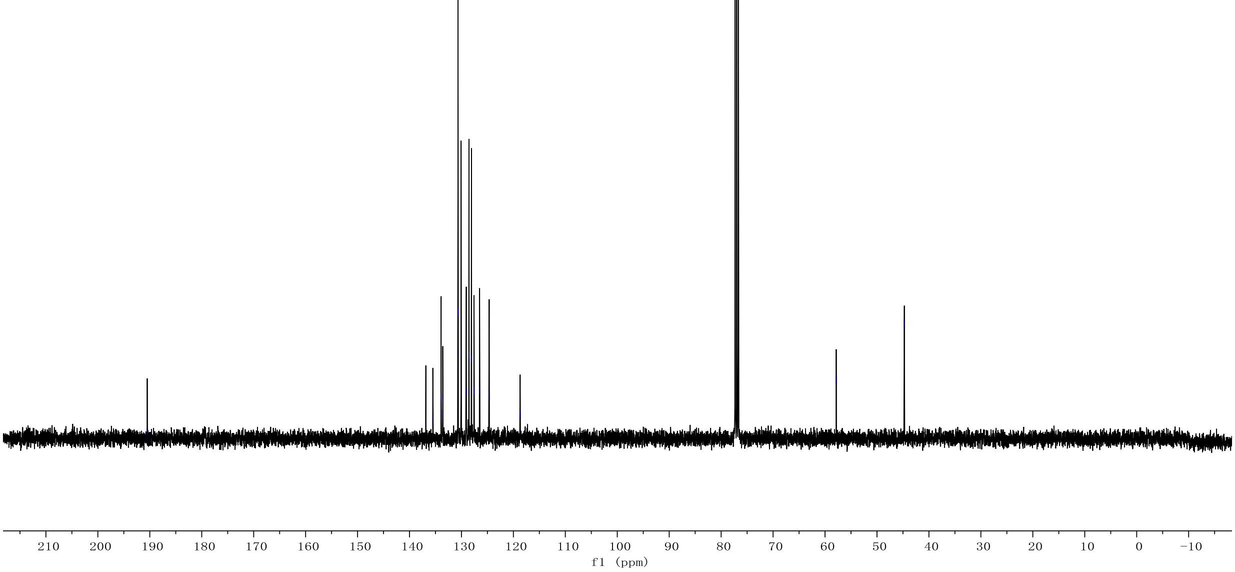

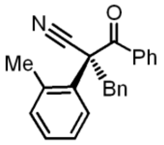

(+)-**22**

^{1}H NMR (500 MHz, CDCl$_3$)

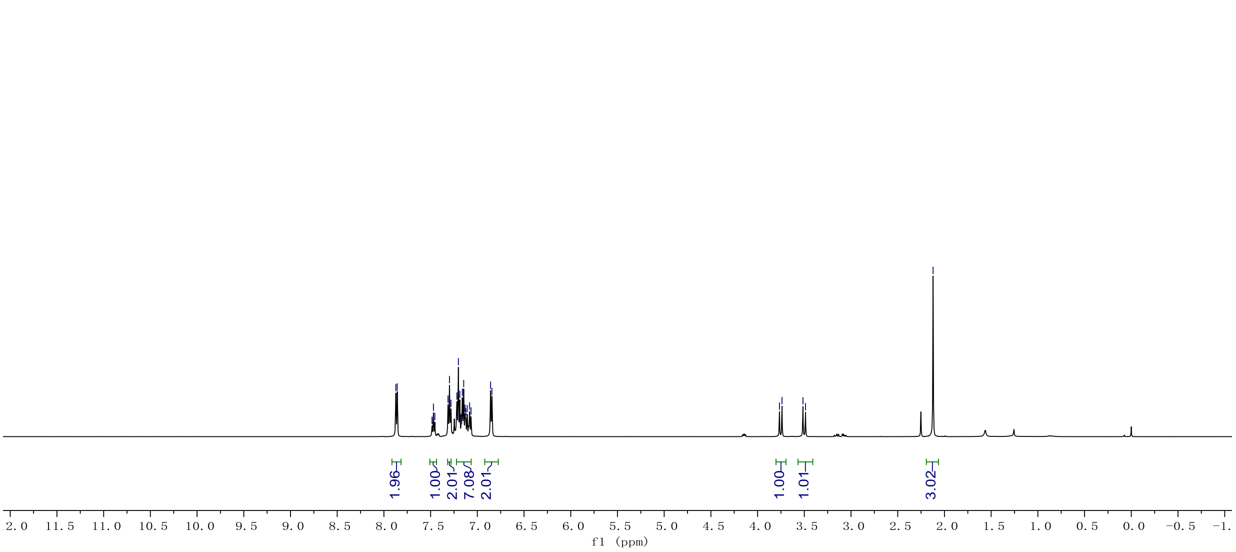

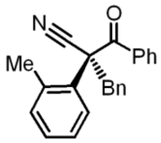

(+)-**22**

^{13}C NMR (126 MHz, CDCl$_3$)

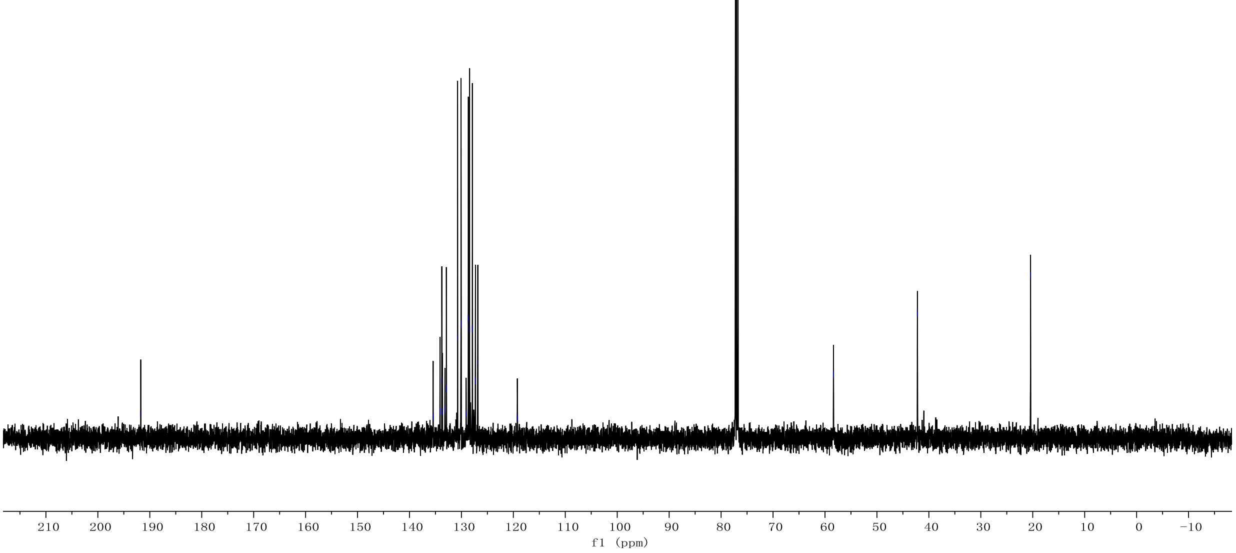

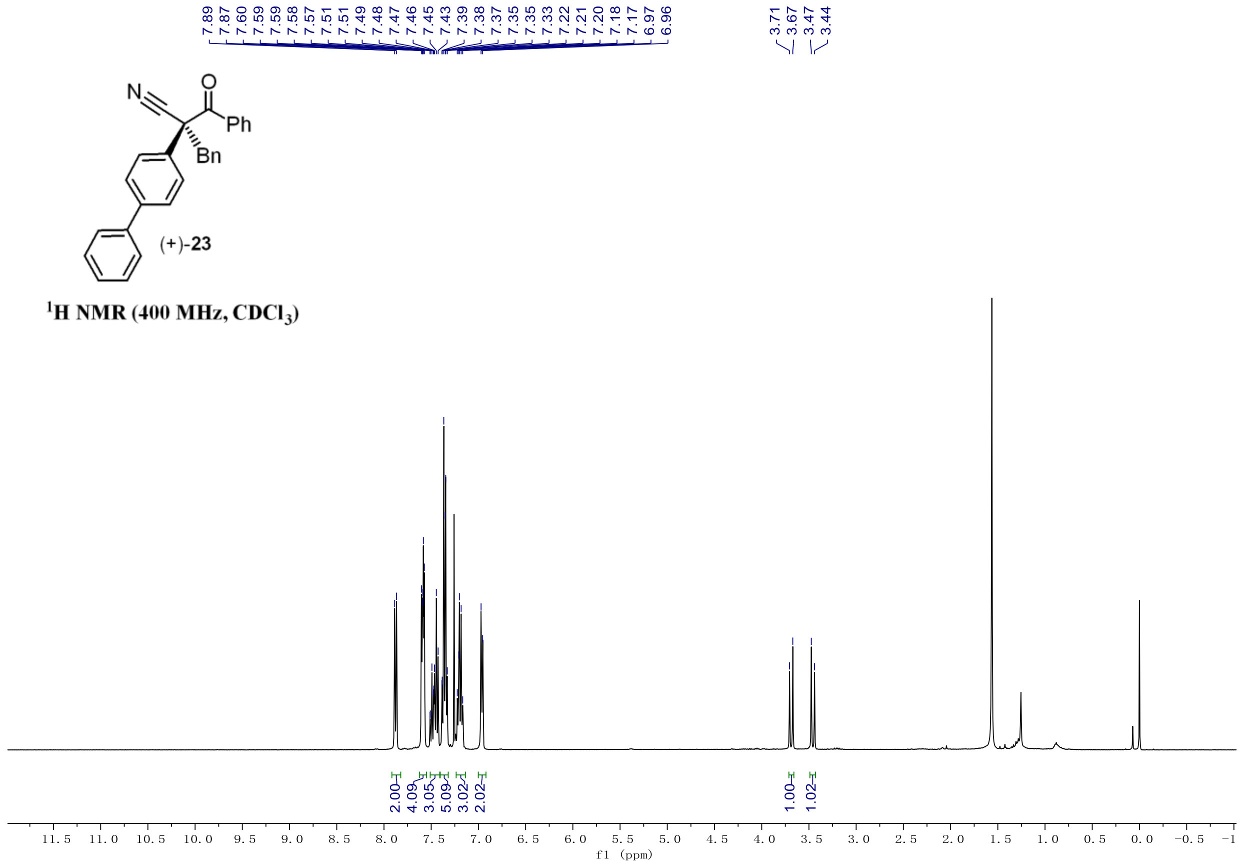

^{1}H NMR (400 MHz, $CDCl_3$)

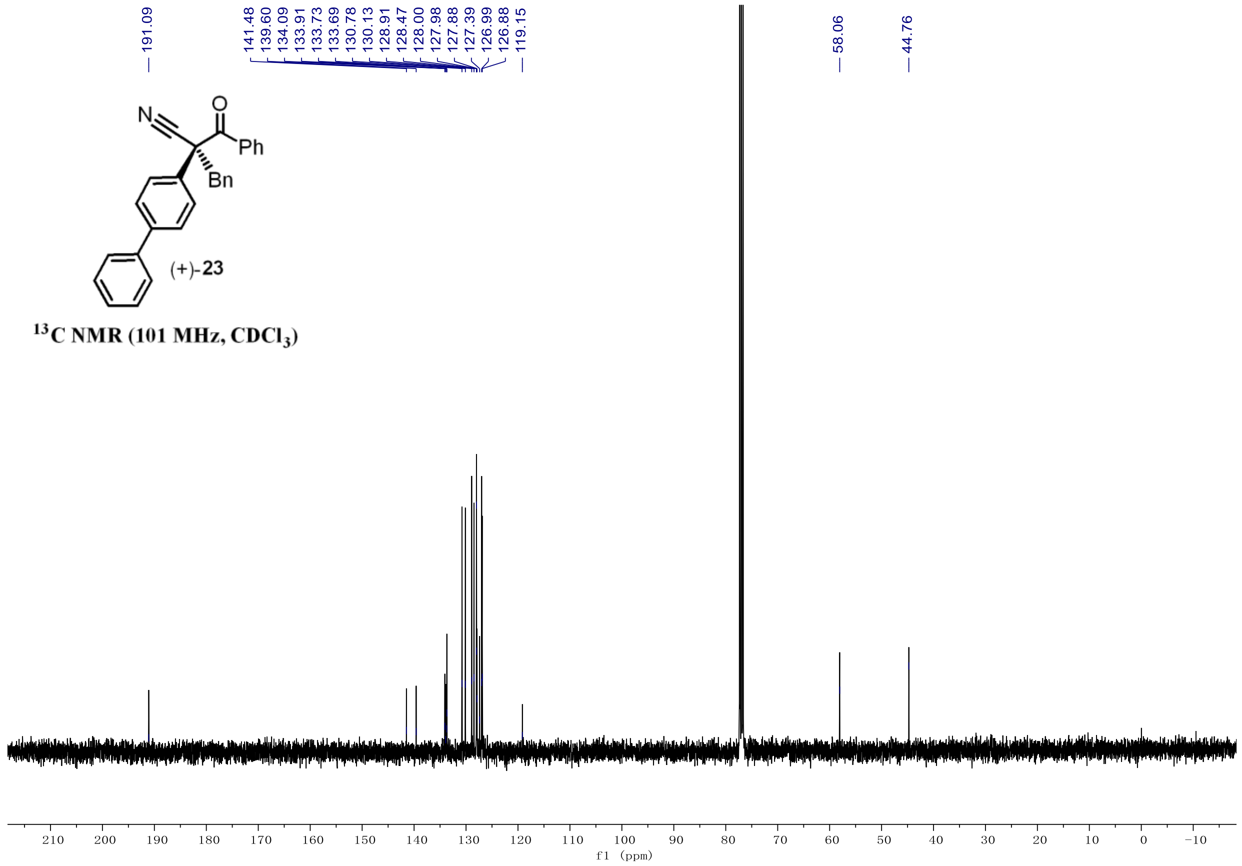

^{13}C NMR (101 MHz, $CDCl_3$)

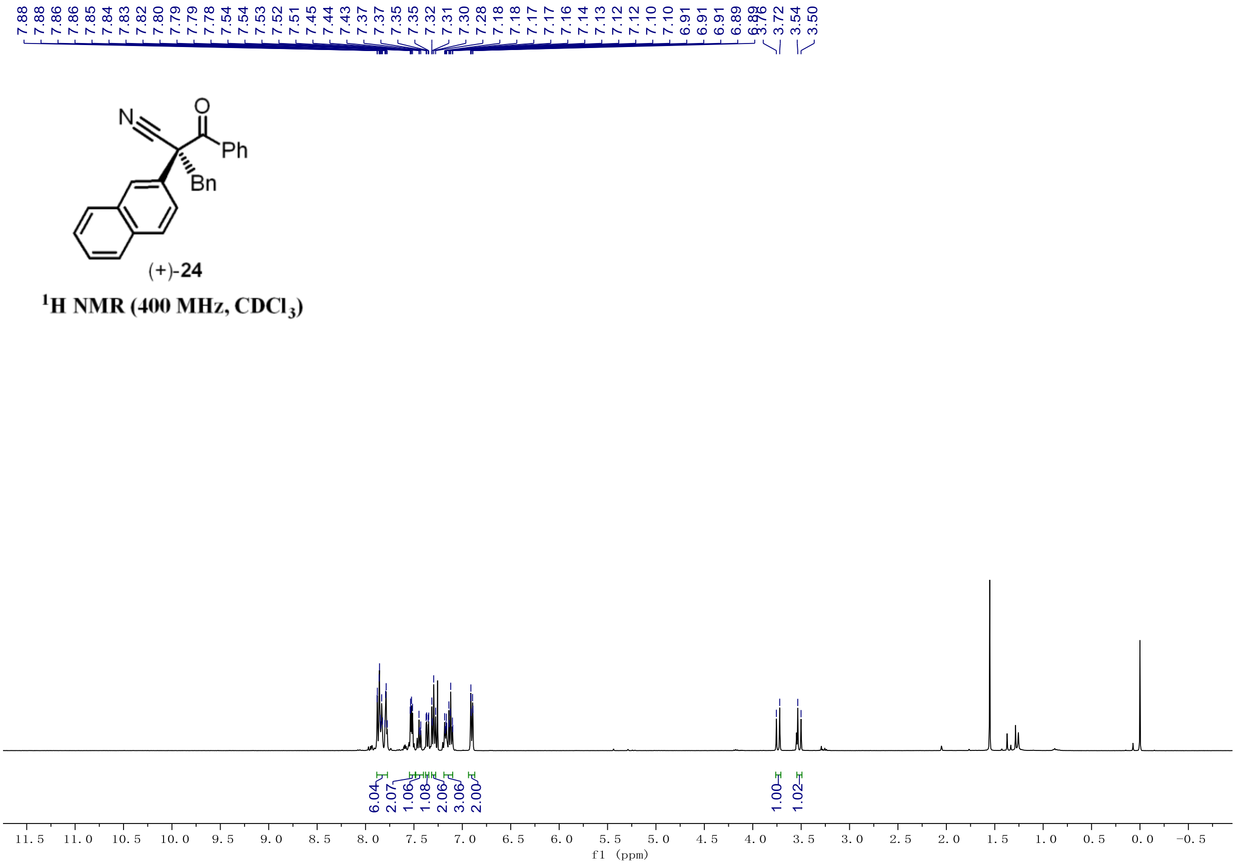
7.88 7.88 7.86 7.86 7.85 7.84 7.83 7.83 7.82 7.80 7.79 7.79 7.78 7.54 7.54 7.53 7.52 7.51 7.45 7.44 7.43 7.37 7.37 7.35 7.35 7.32 7.31 7.30 7.28 7.18 7.18 7.17 7.17 7.16 7.14 7.13 7.12 7.12 7.10 7.10 6.91 6.91 6.89 6.89 3.76 3.72 3.54 3.50
N
O
Ph
Bn
(+)-24
^{1}H NMR (400 MHz, CDCl$_3$)
6.04 2.07 1.06 1.08 2.06 3.06 2.00 1.00 1.02
11.5 11.0 10.5 10.0 9.5 9.0 8.5 8.0 7.5 7.0 6.5 6.0 5.5 5.0 4.5 4.0 3.5 3.0 2.5 2.0 1.5 1.0 0.5 0.0 -0.5
f1 (ppm)

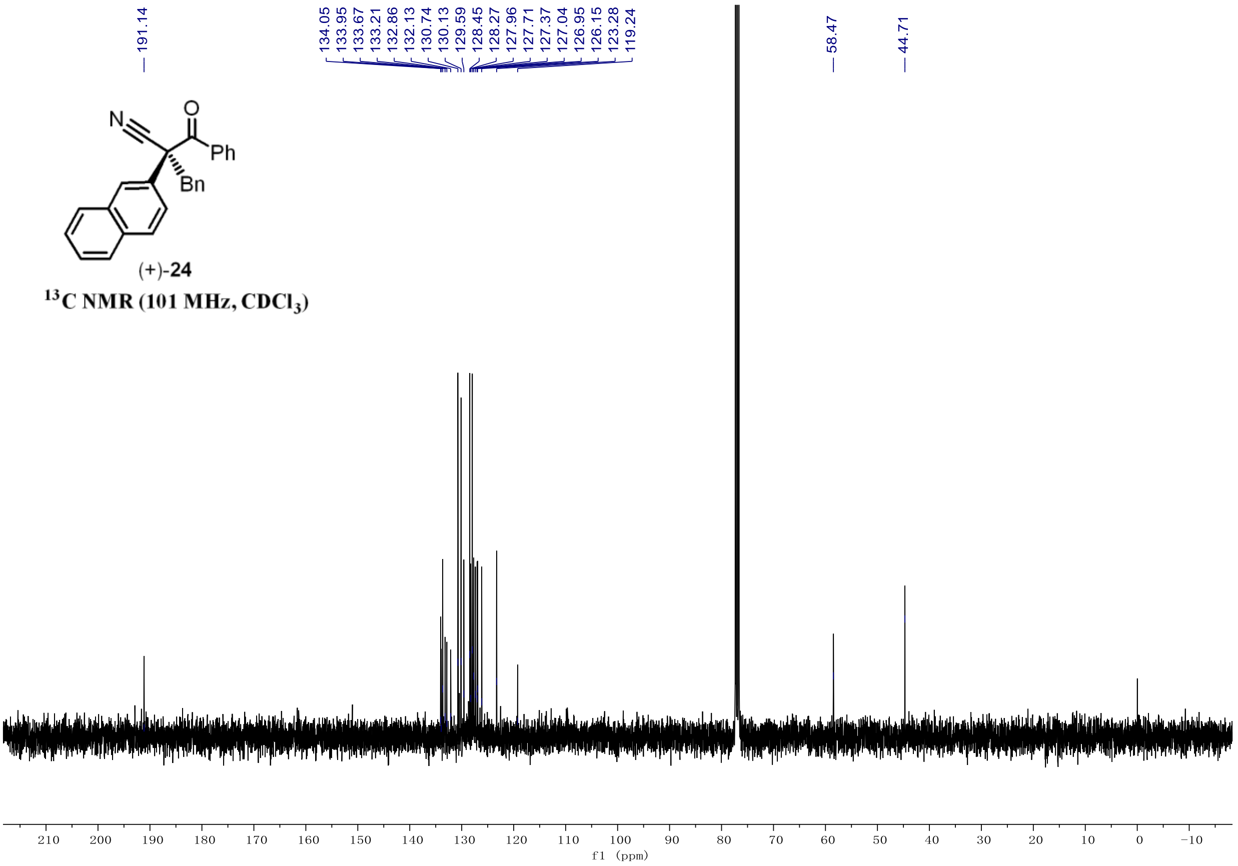
191.14
134.05 133.95 133.67 133.21 132.86 132.13 130.74 130.13 129.59 128.45 128.27 127.96 127.71 127.37 127.04 126.95 126.15 123.28 119.24
58.47
44.71
N
O
Ph
Bn
(+)-24
^{13}C NMR (101 MHz, CDCl$_3$)
210 200 190 180 170 160 150 140 130 120 110 100 90 80 70 60 50 40 30 20 10 0 -10
f1 (ppm)

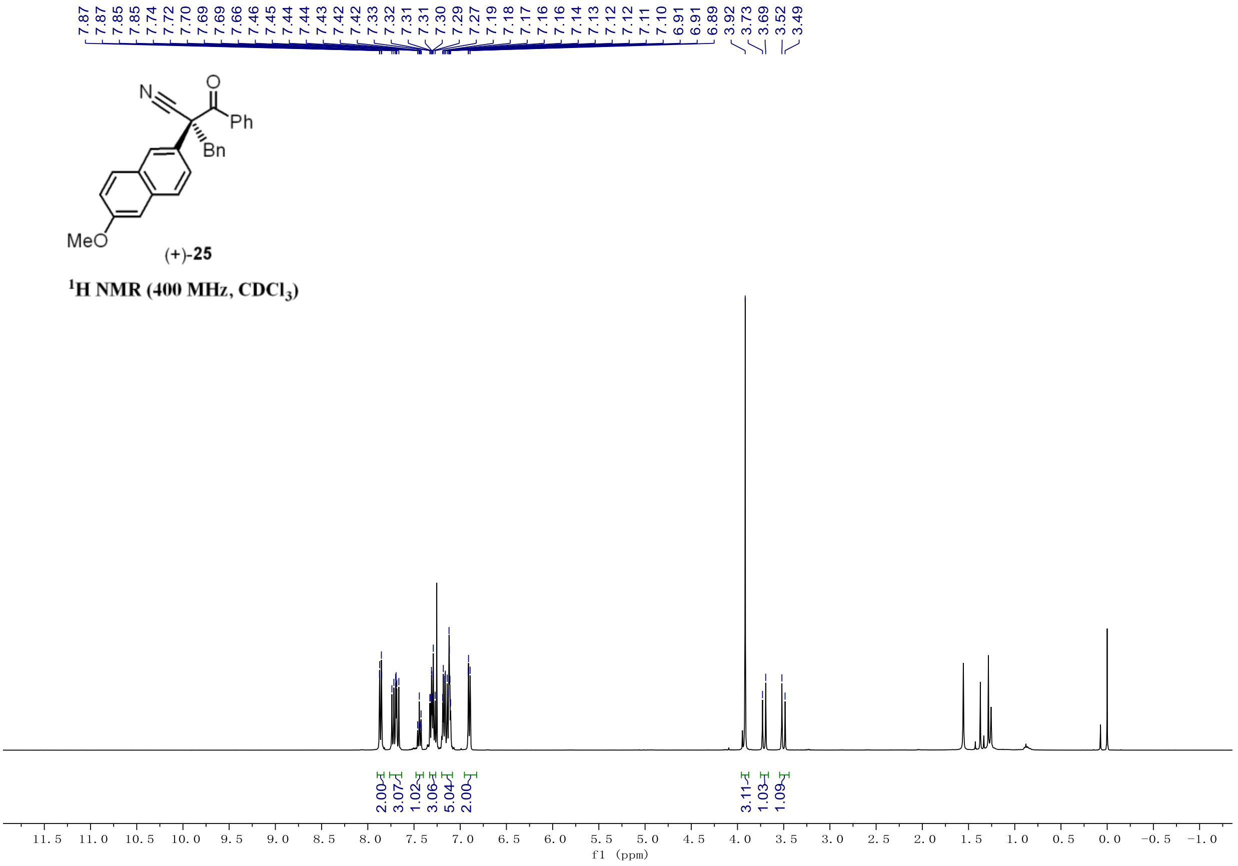

(+)-**25**

^{1}H NMR (400 MHz, $CDCl_3$)

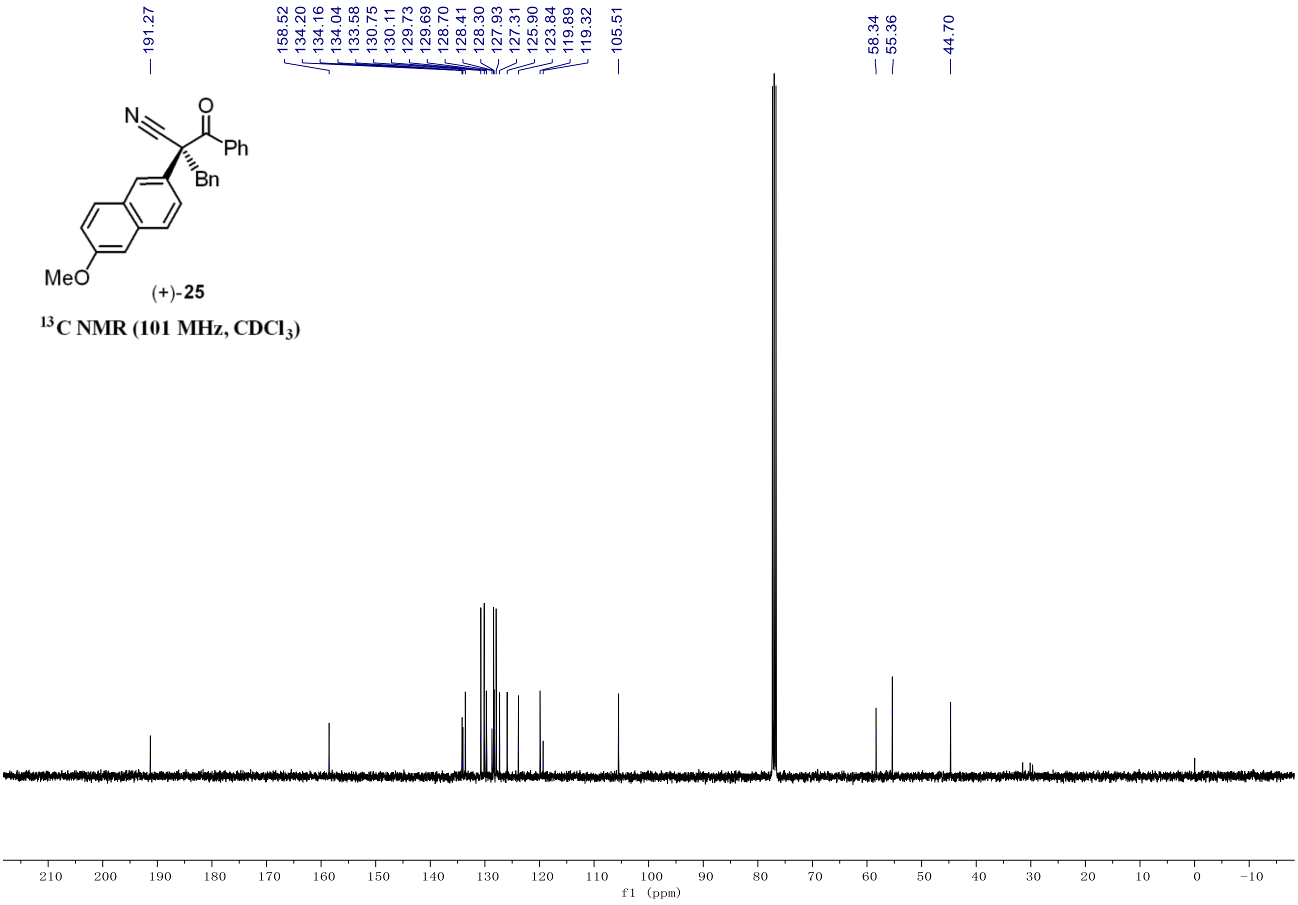

(+)-**25**

^{13}C NMR (101 MHz, $CDCl_3$)

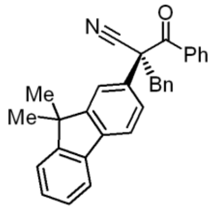

(+)-**26**

^{1}H NMR (400 MHz, $CDCl_3$)

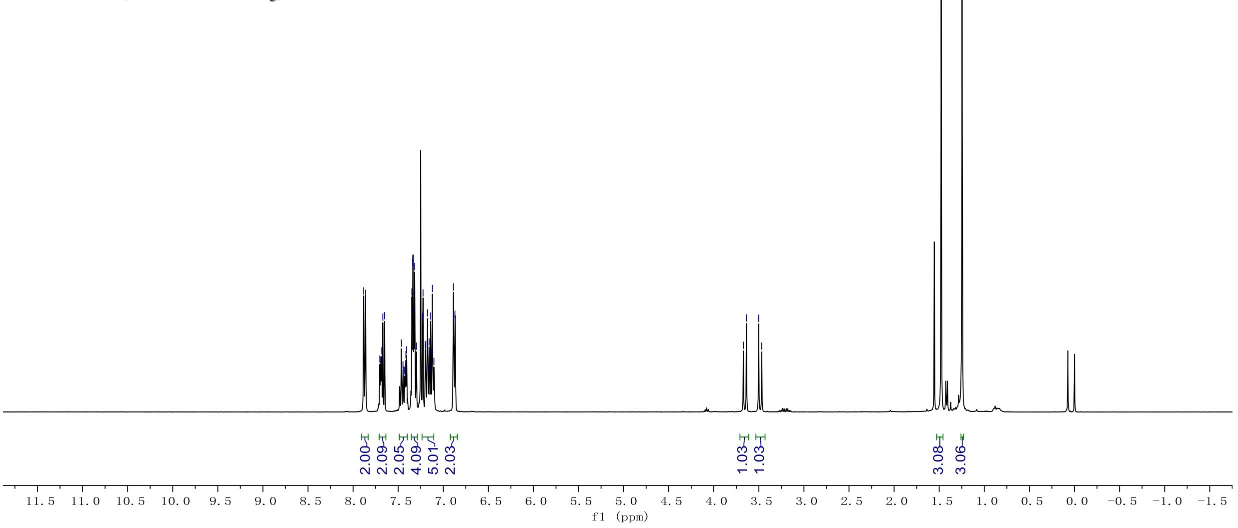

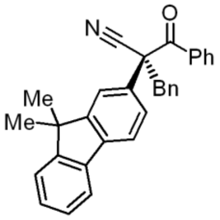

(+)-**26**

^{13}C NMR (101 MHz, $CDCl_3$)

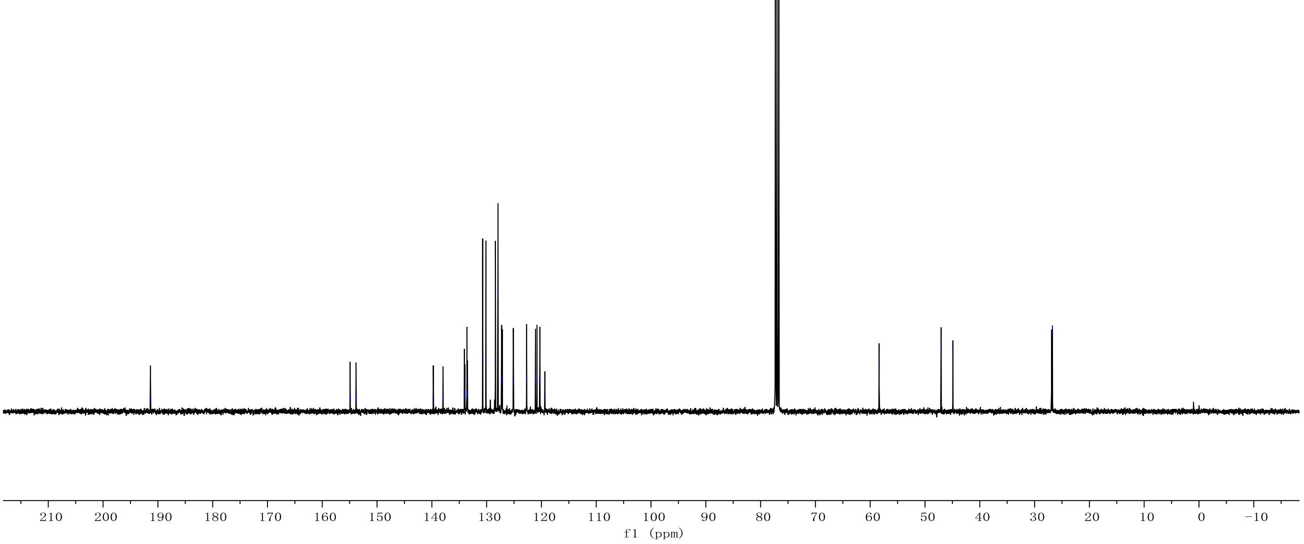

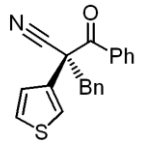

(+)-**27**

^{1}H NMR (400 MHz, $CDCl_3$)

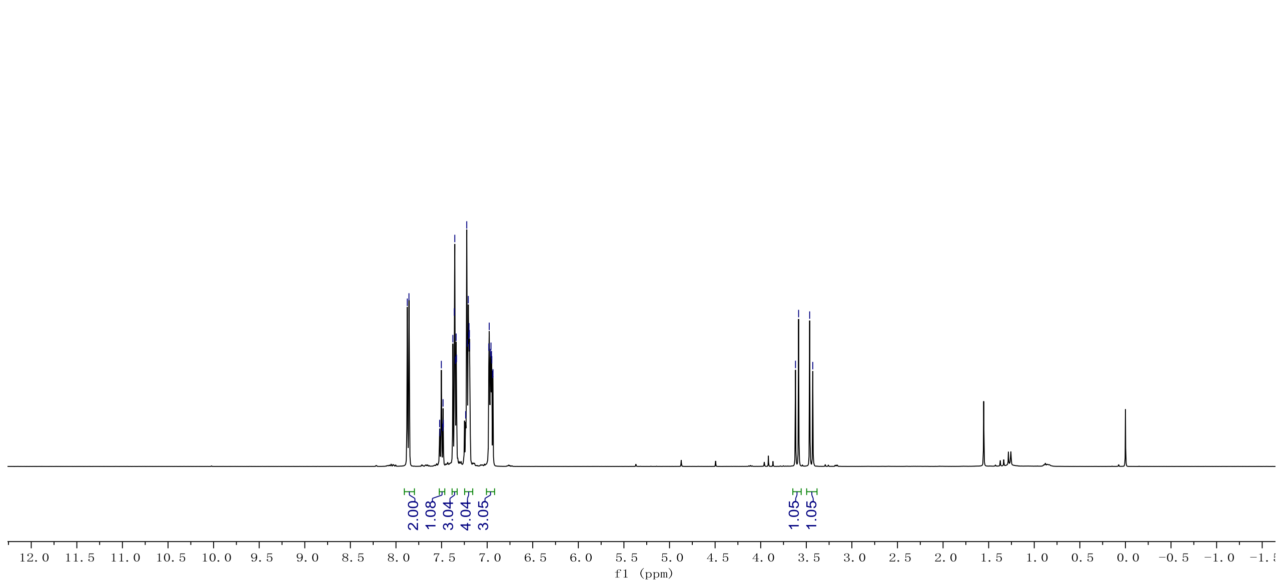

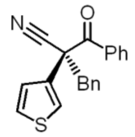

(+)-**27**

^{13}C NMR (101 MHz, $CDCl_3$)

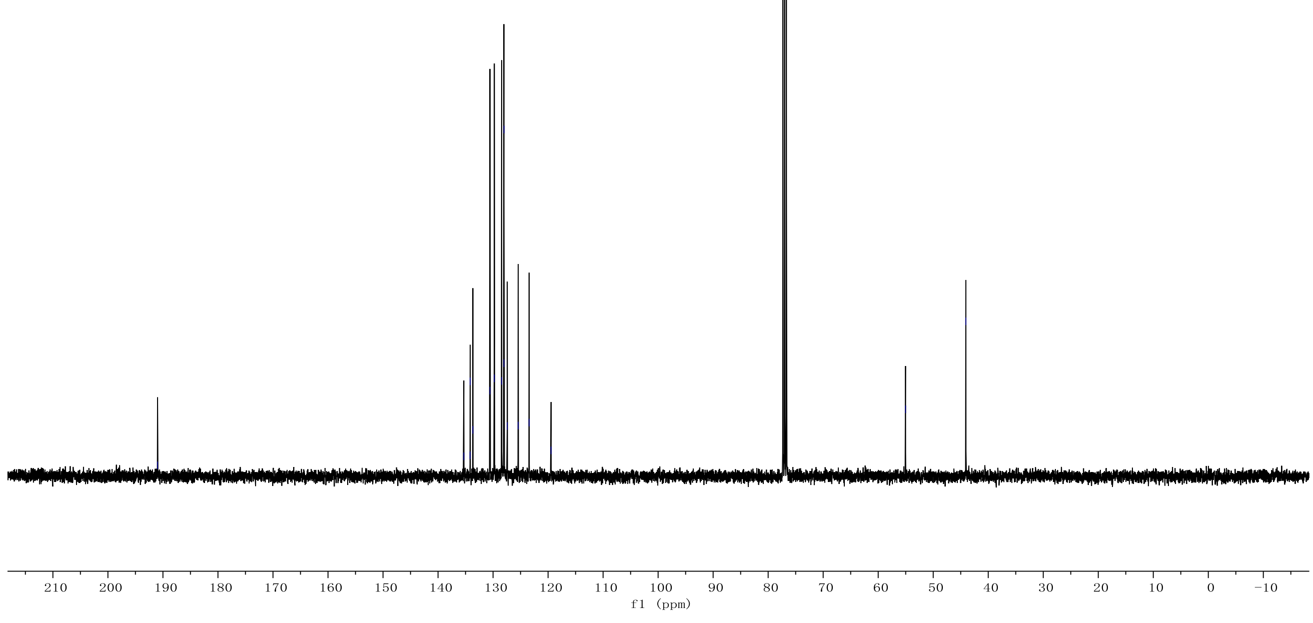

(+)-**28**

^{1}H NMR (400 MHz, CDCl$_3$)

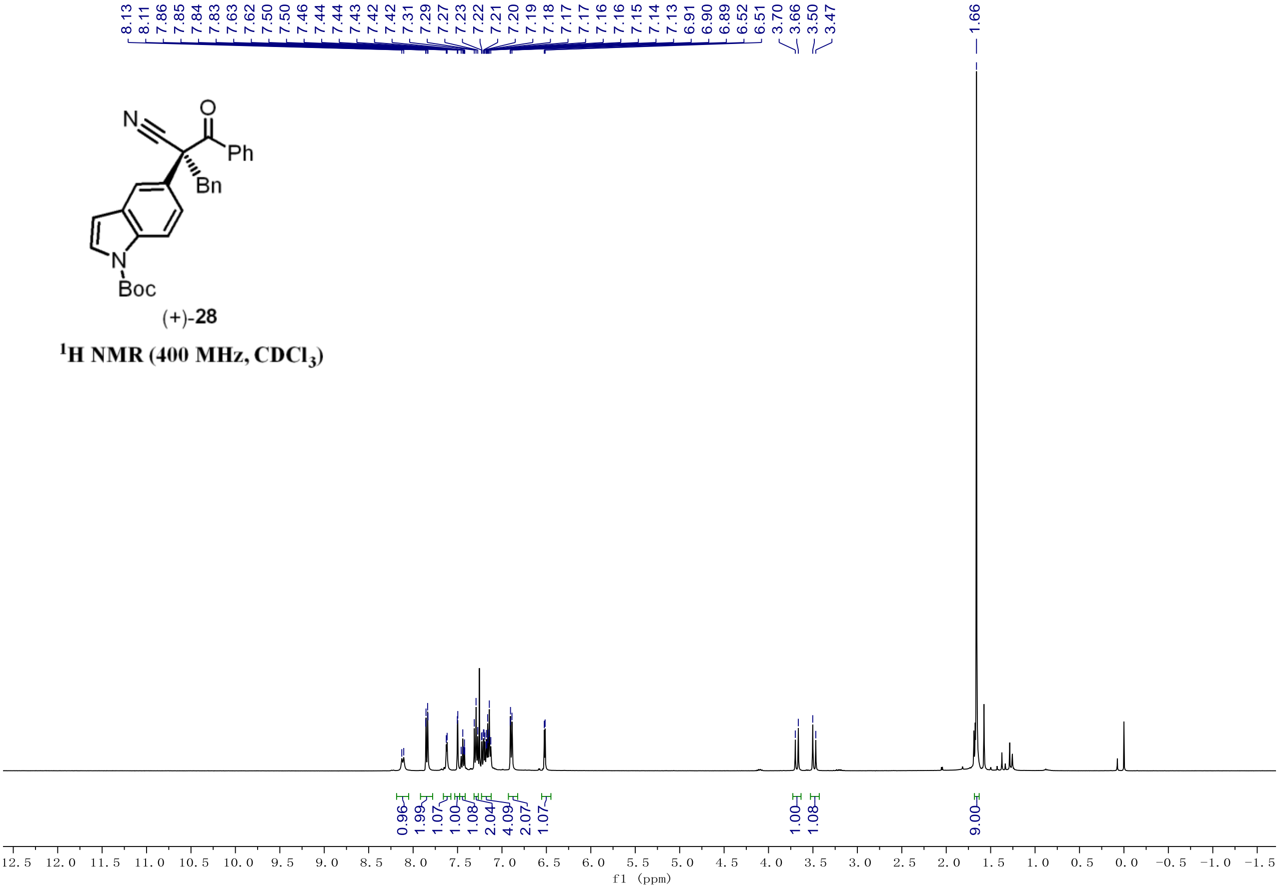

(+)-**28**

^{13}C NMR (101 MHz, CDCl$_3$)

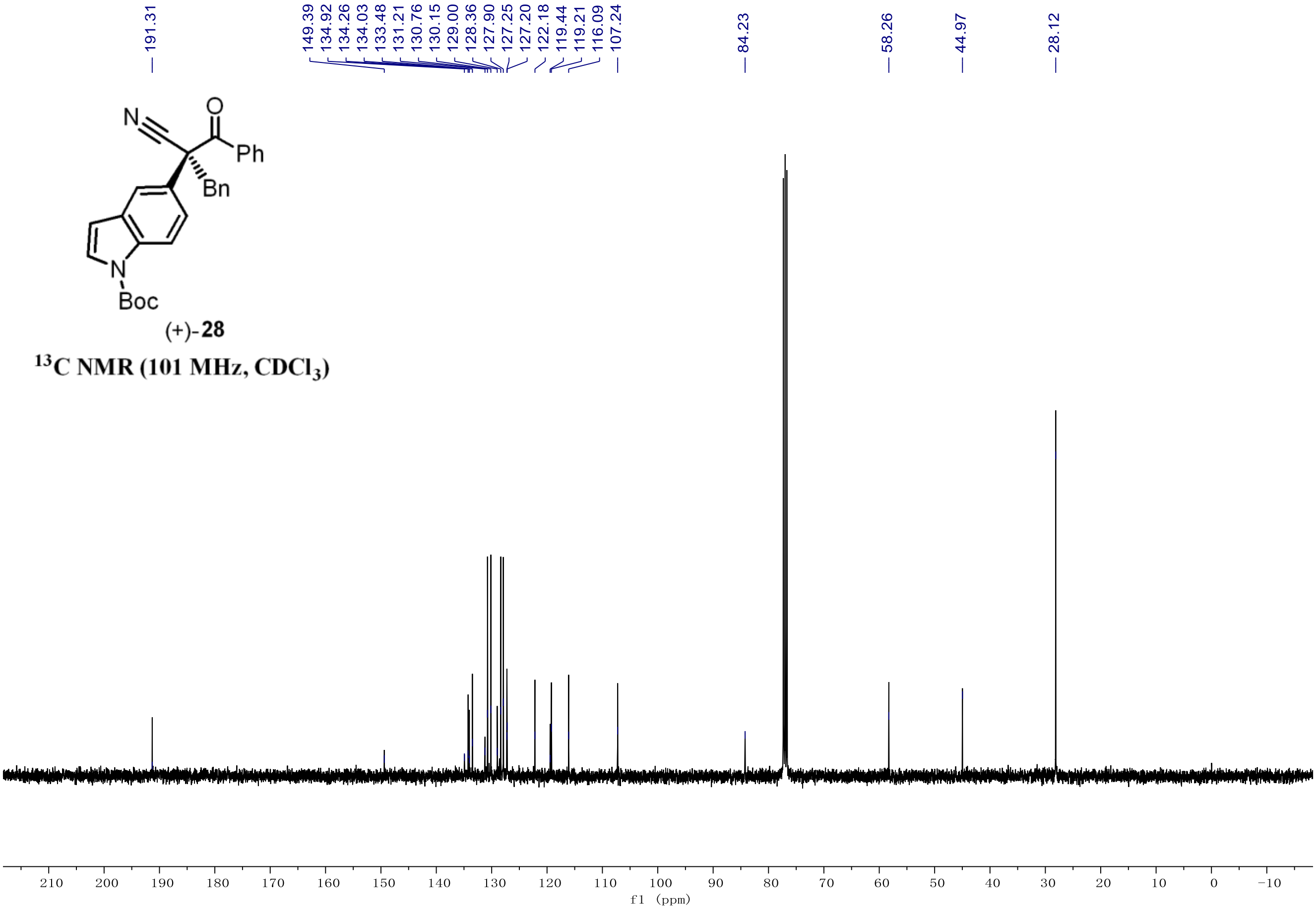

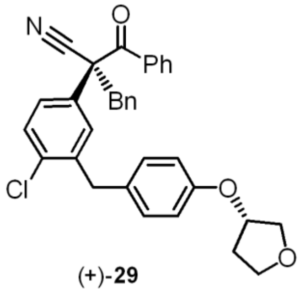

(+)-**29**

^{1}H NMR (500 MHz, CDCl$_3$)

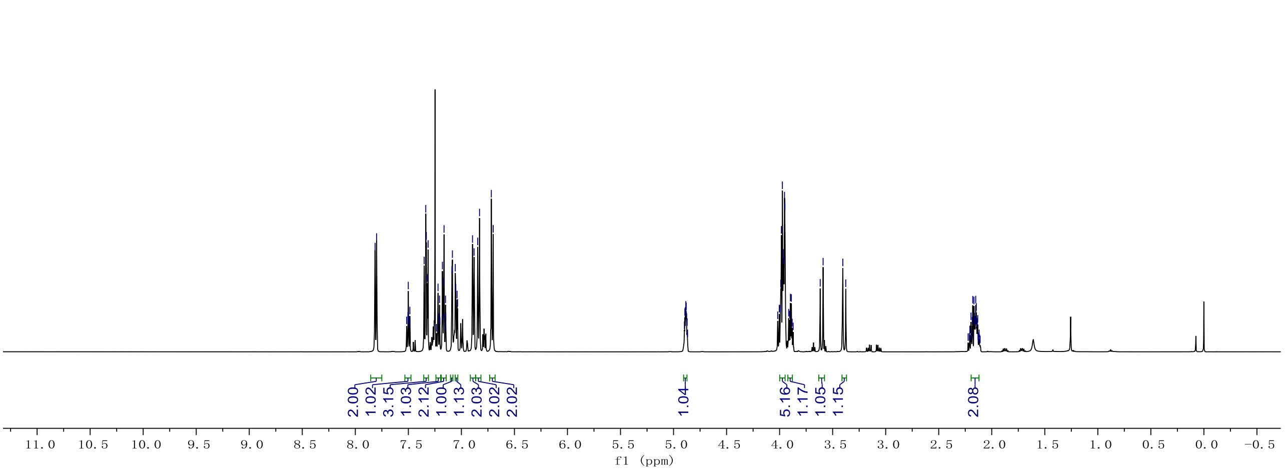

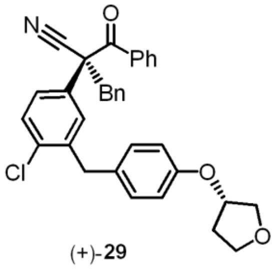

(+)-**29**

^{13}C NMR (126 MHz, CDCl$_3$)

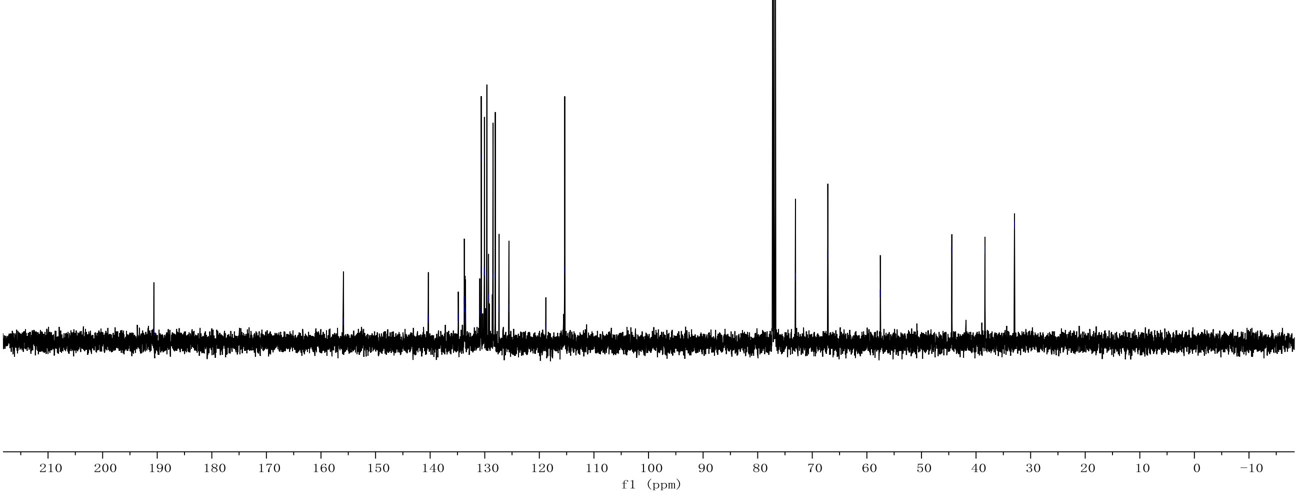

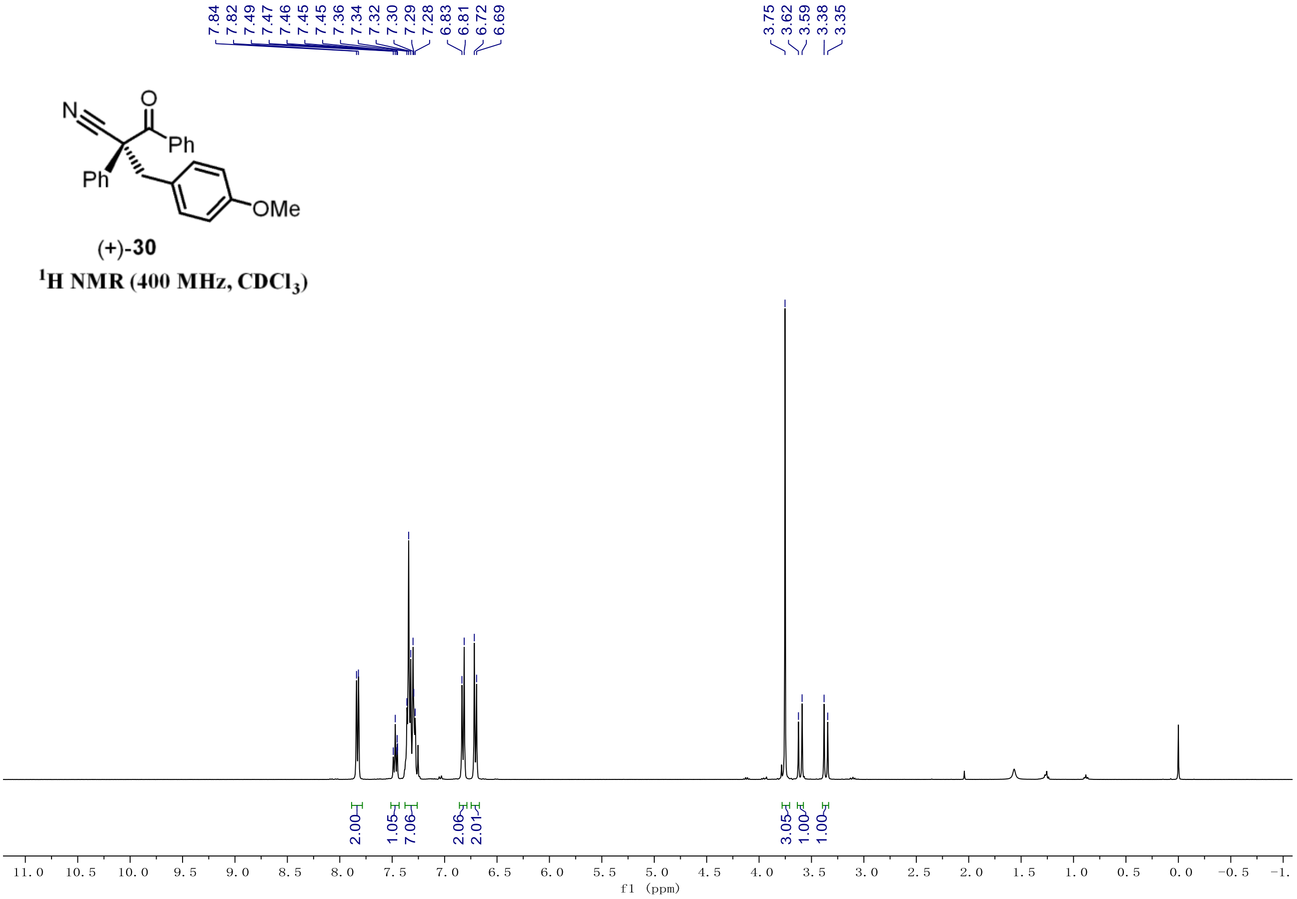
7.84 7.82 7.49 7.47 7.46 7.45 7.45 7.36 7.34 7.32 7.30 7.29 7.28 6.83 6.81 6.72 6.69
3.75 3.62 3.59 3.38 3.35
N O Ph Ph OMe
(+)-30
1H NMR (400 MHz, CDCl3)
2.00 1.05 7.06 2.06 2.01 3.05 1.00 1.00
11.0 10.5 10.0 9.5 9.0 8.5 8.0 7.5 7.0 6.5 6.0 5.5 5.0 4.5 4.0 3.5 3.0 2.5 2.0 1.5 1.0 0.5 0.0 -0.5 -1.
f1 (ppm)

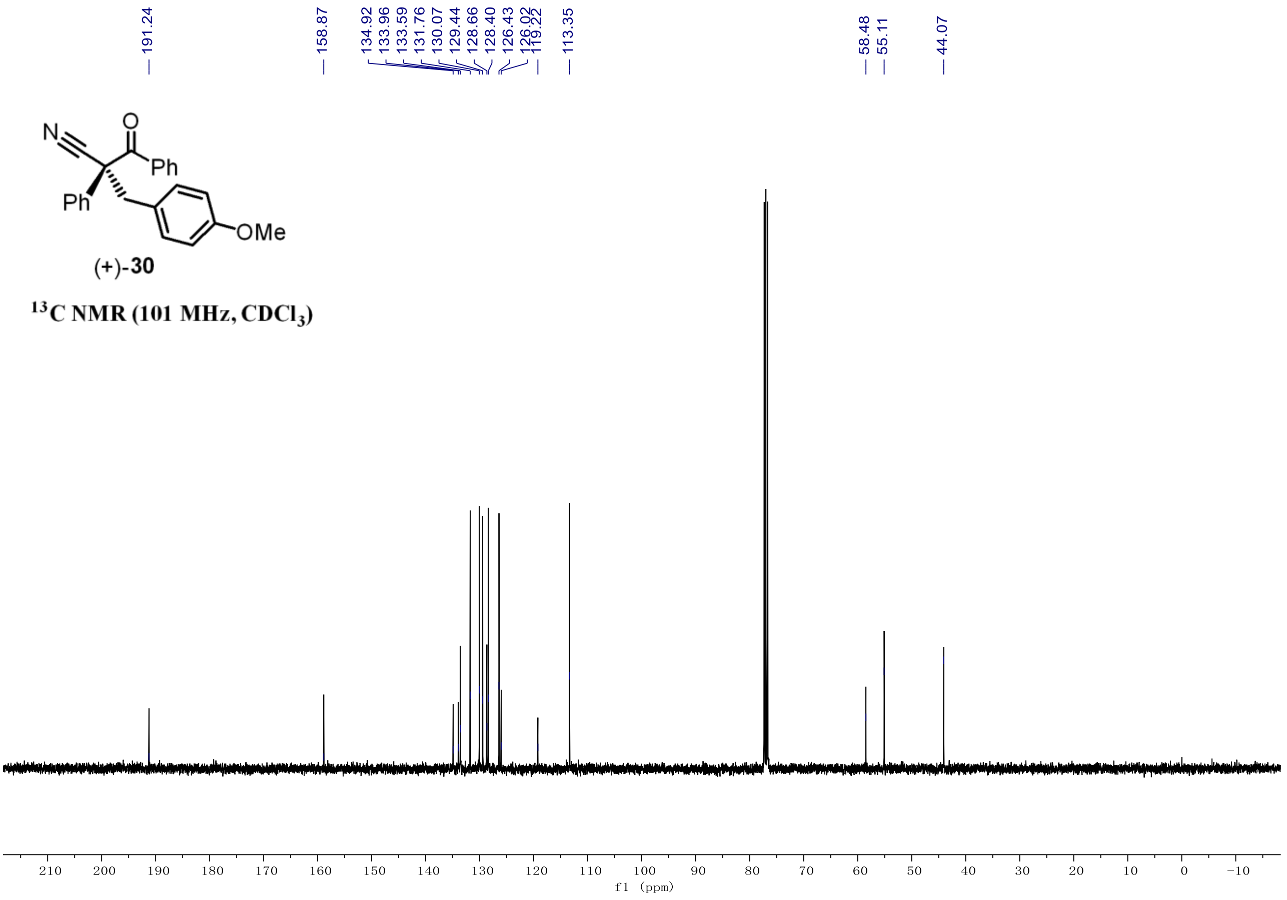
191.24
158.87
134.92 133.96 133.59 131.76 130.07 129.44 128.66 128.40 126.43 126.02 119.22
113.35
58.48 55.11
44.07
N O Ph Ph OMe
(+)-30
13C NMR (101 MHz, CDCl3)
210 200 190 180 170 160 150 140 130 120 110 100 90 80 70 60 50 40 30 20 10 0 -10
f1 (ppm)

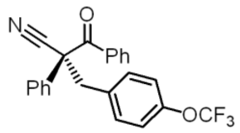

(+)-**31**

^{1}H NMR (500 MHz, CDCl$_3$)

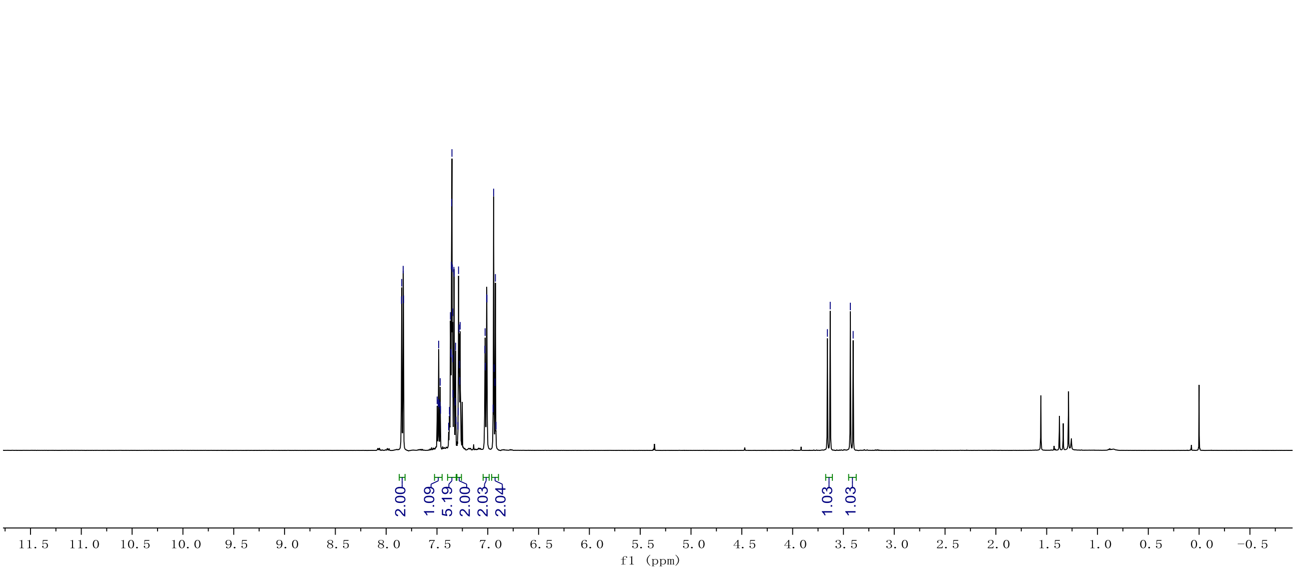

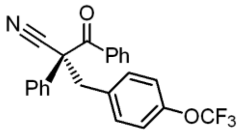

(+)-**31**

^{13}C NMR (126 MHz, CDCl$_3$)

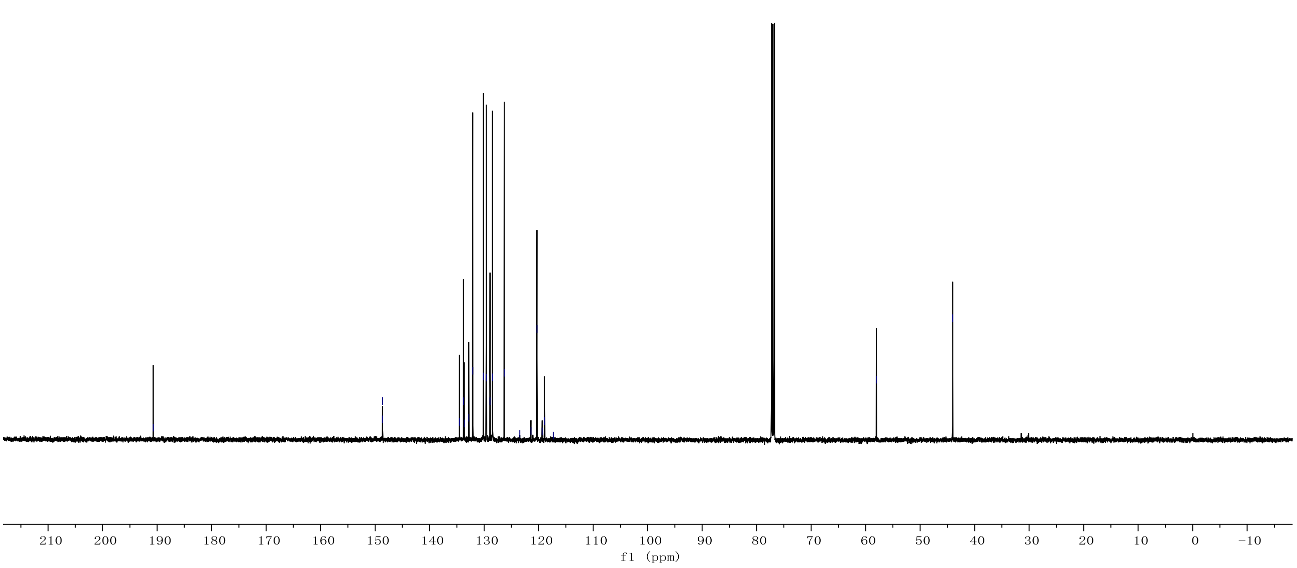

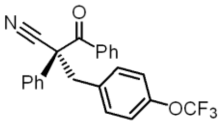

(+)-**31**

^{19}F NMR (471 MHz, $CDCl_3$)

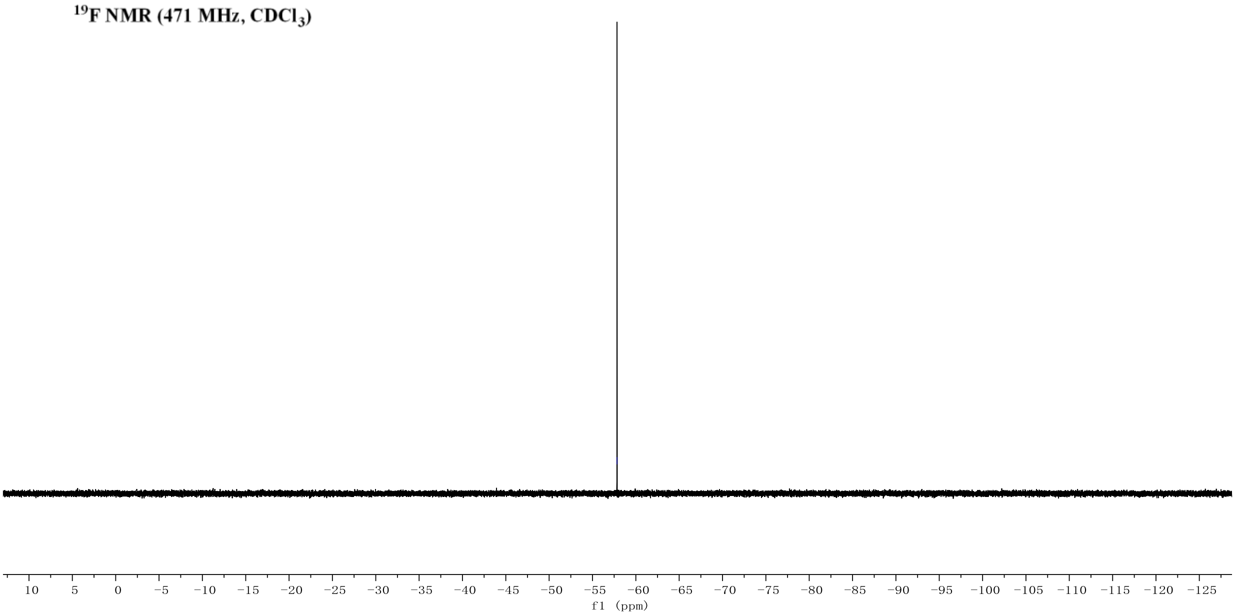

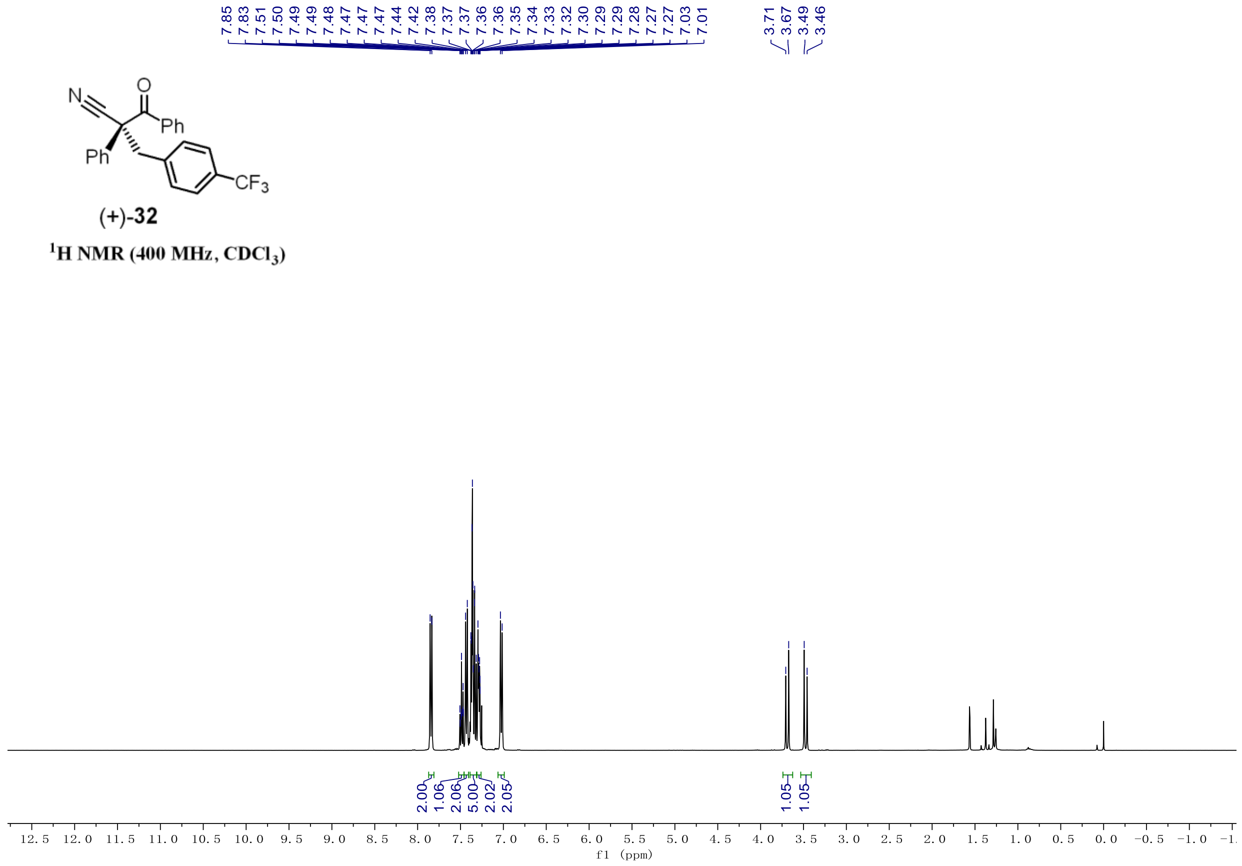
7.85 7.83 7.51 7.50 7.49 7.49 7.48 7.47 7.47 7.47 7.44 7.42 7.38 7.37 7.37 7.36 7.36 7.35 7.34 7.33 7.32 7.30 7.29 7.29 7.28 7.27 7.27 7.03 7.01
3.71 3.67 3.49 3.46
N
O
Ph
Ph
CF3
(+)-32
1H NMR (400 MHz, CDCl3)
2.00 1.06 2.06 5.00 2.02 2.05
1.05 1.05
f1 (ppm)

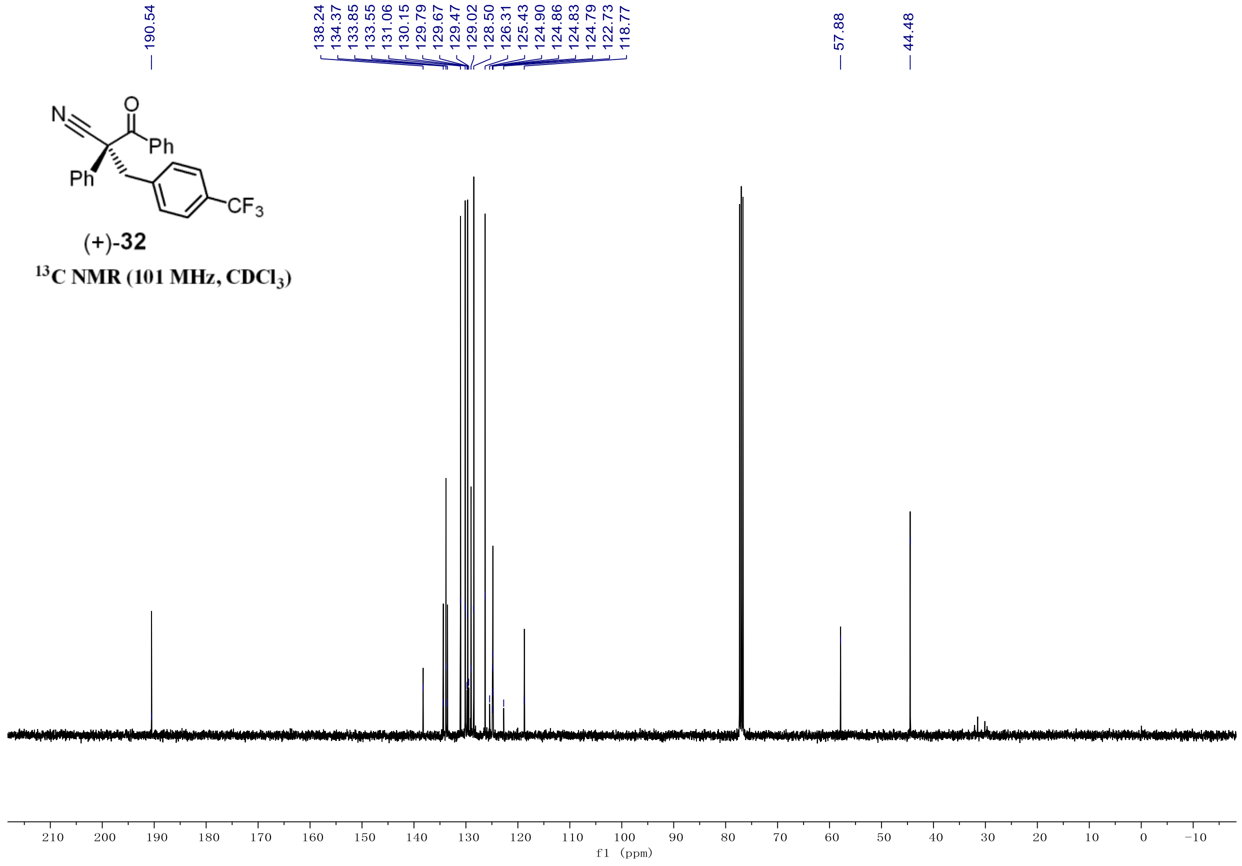
190.54
138.24 134.37 133.85 133.55 131.06 130.15 129.79 129.67 129.47 129.02 128.50 126.31 125.43 124.90 124.86 124.83 124.79 122.73 118.77
57.88
44.48
N
O
Ph
Ph
CF3
(+)-32
13C NMR (101 MHz, CDCl3)
f1 (ppm)

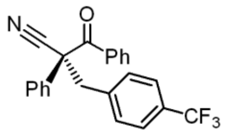

(+)-**32**

^{19}F NMR (376 MHz, $CDCl_3$)

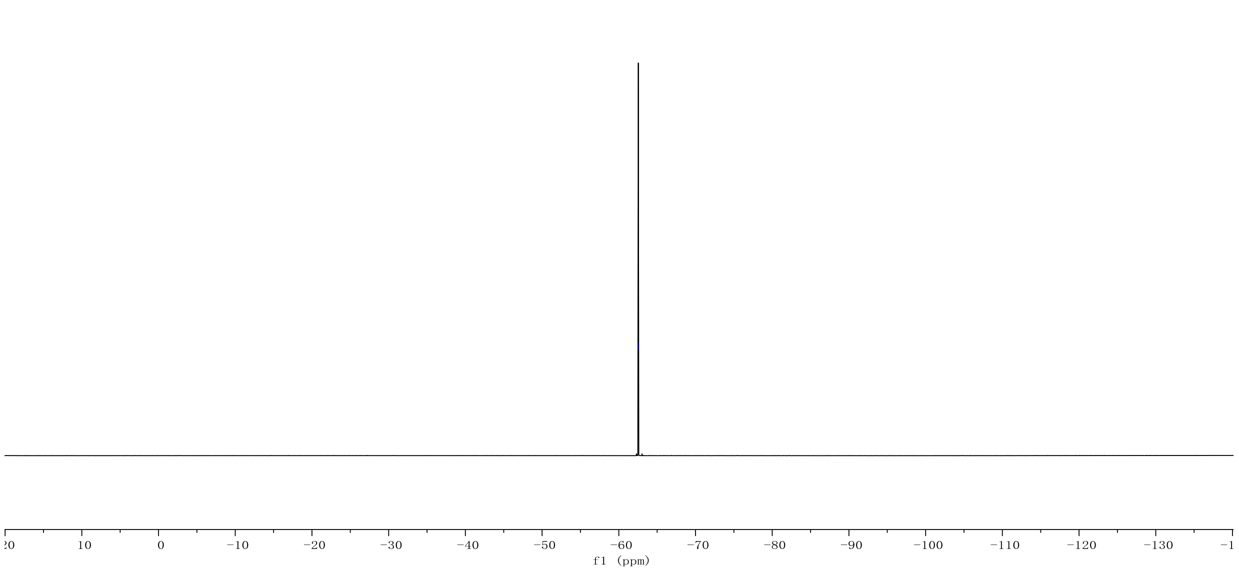

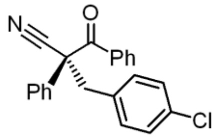

(+)-**33**

^{1}H NMR (400 MHz, $CDCl_3$)

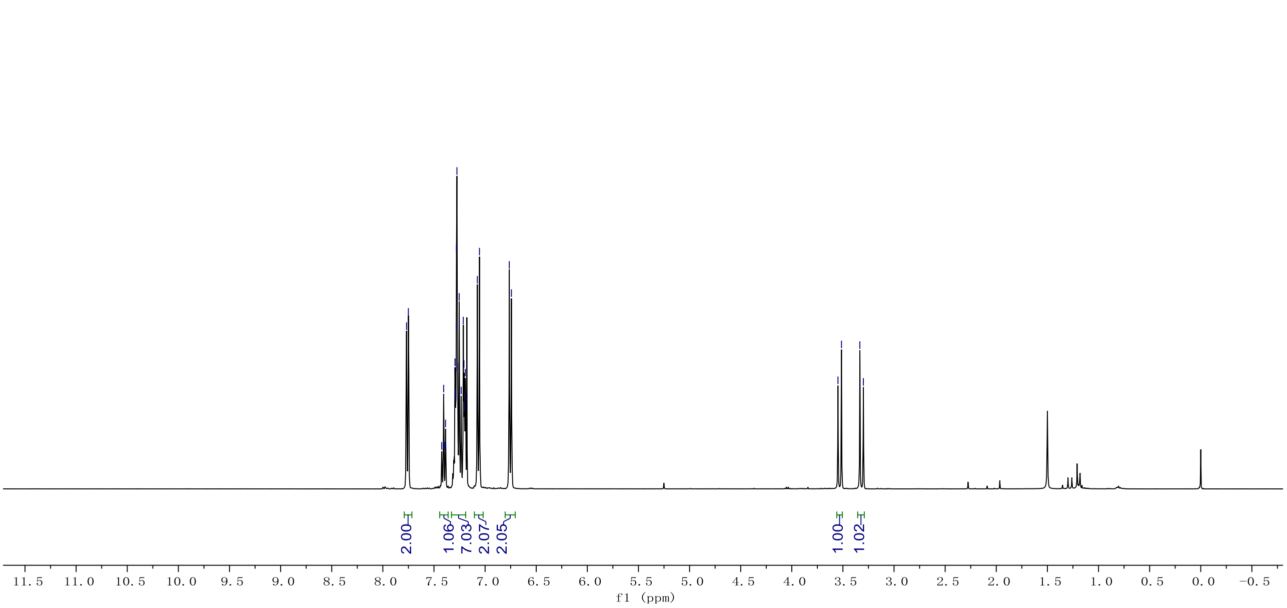

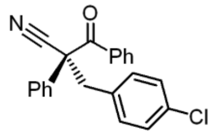

(+)-**33**

^{13}C NMR (101 MHz, $CDCl_3$)

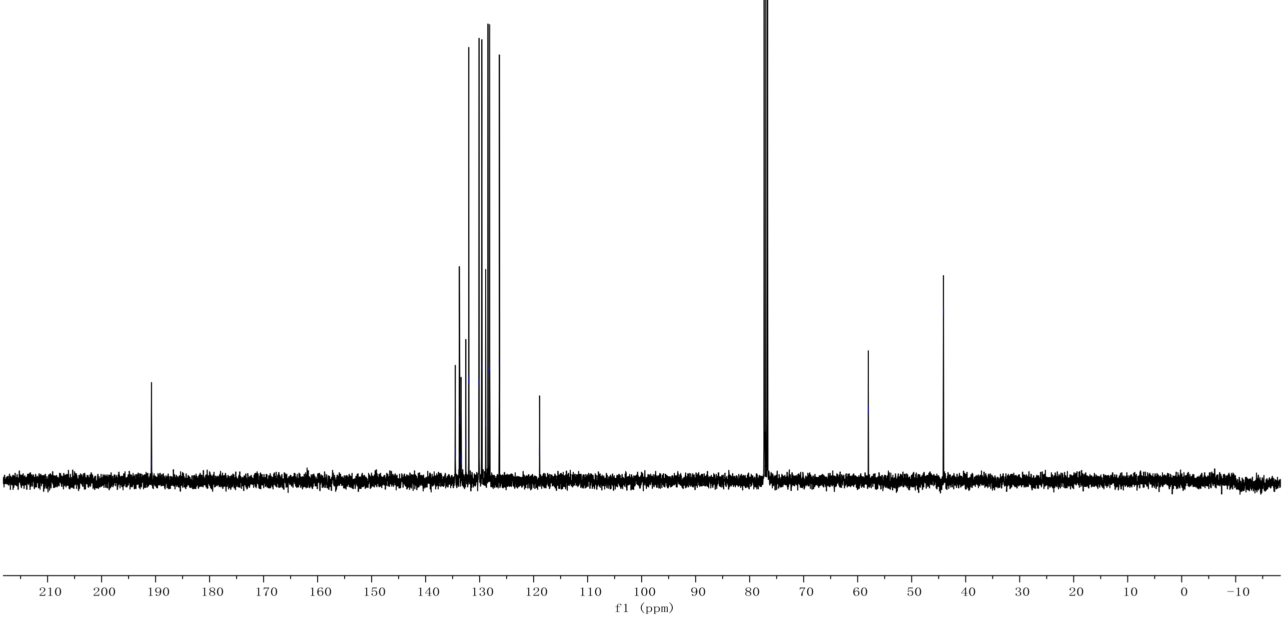

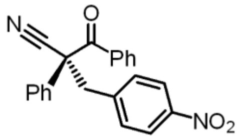

(+)-**34**

^{1}H NMR (500 MHz, $CDCl_3$)

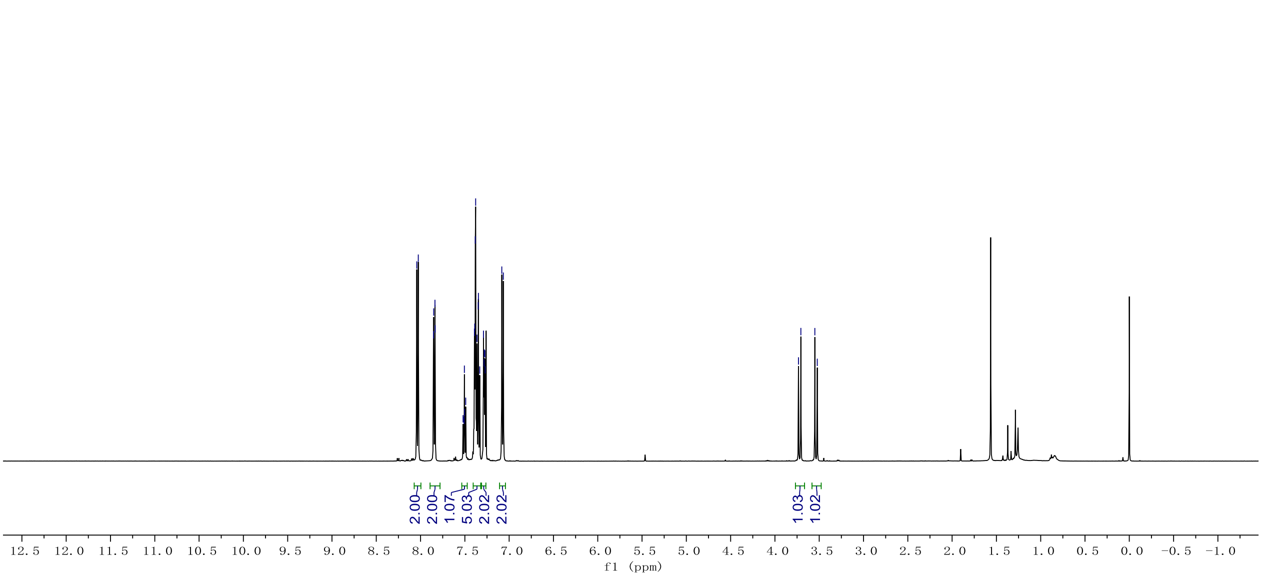

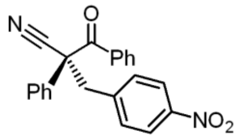

(+)-**34**

^{13}C NMR (126 MHz, $CDCl_3$)

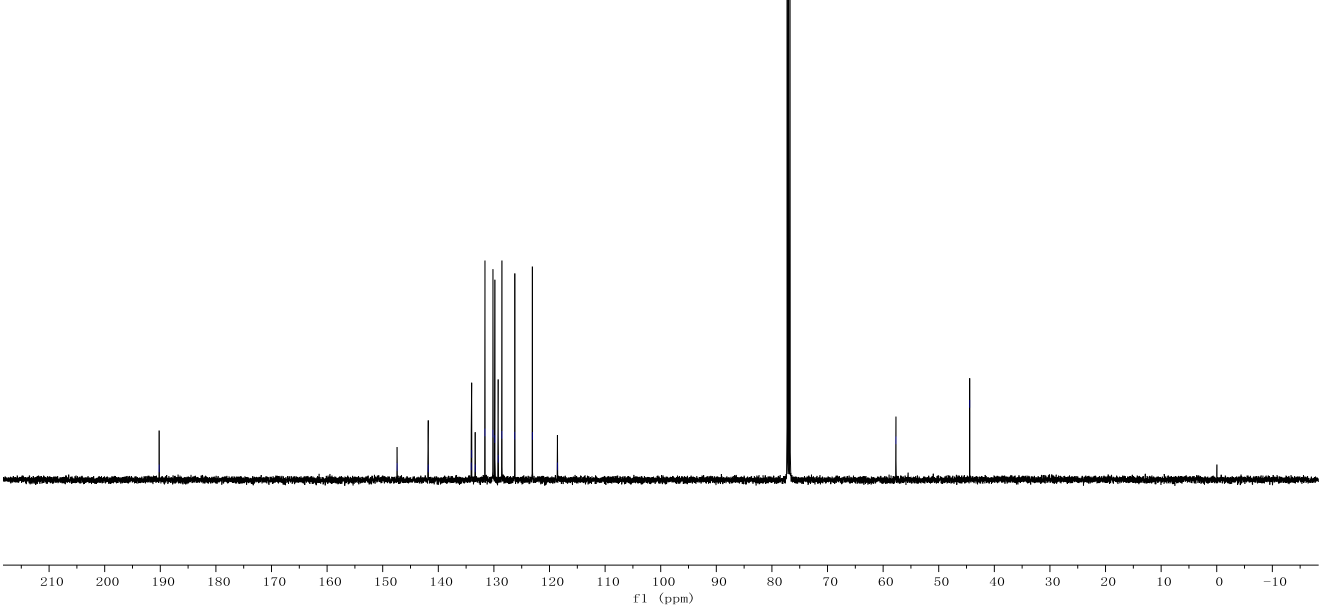

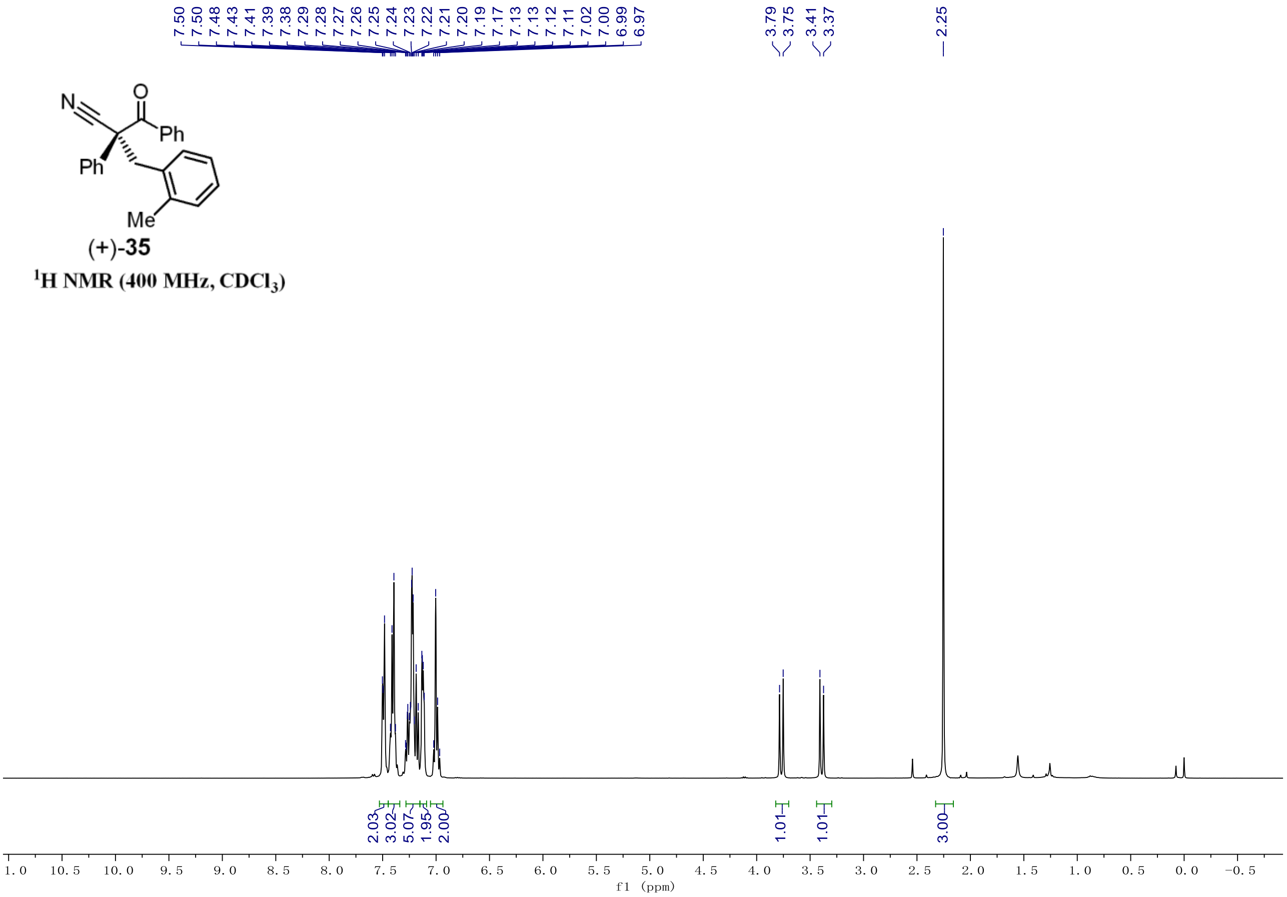

(+)-**35**

^{1}H NMR (400 MHz, $CDCl_3$)

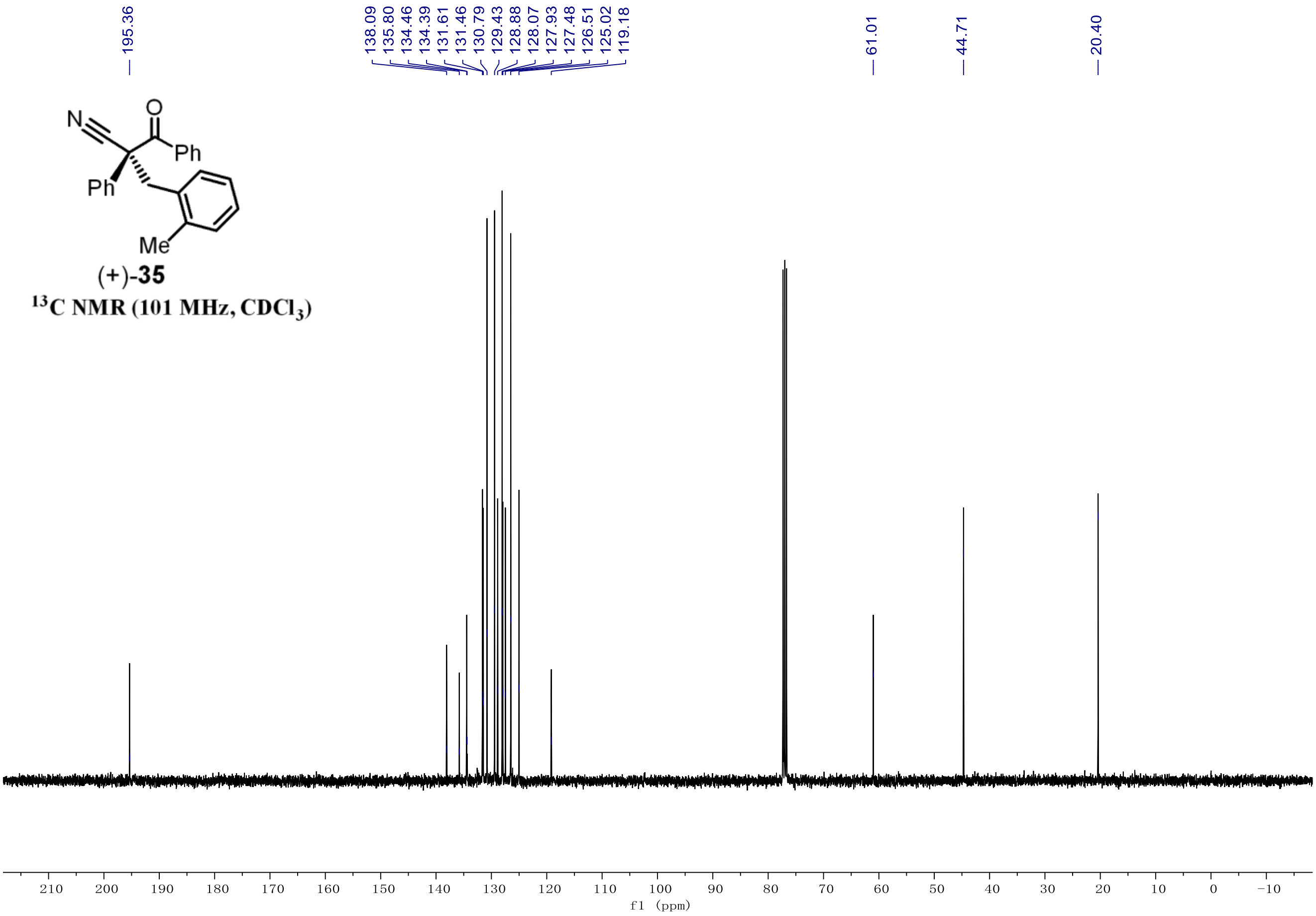

(+)-**35**

^{13}C NMR (101 MHz, $CDCl_3$)

(+)-**36**

^{1}H NMR (500 MHz, $CDCl_3$)

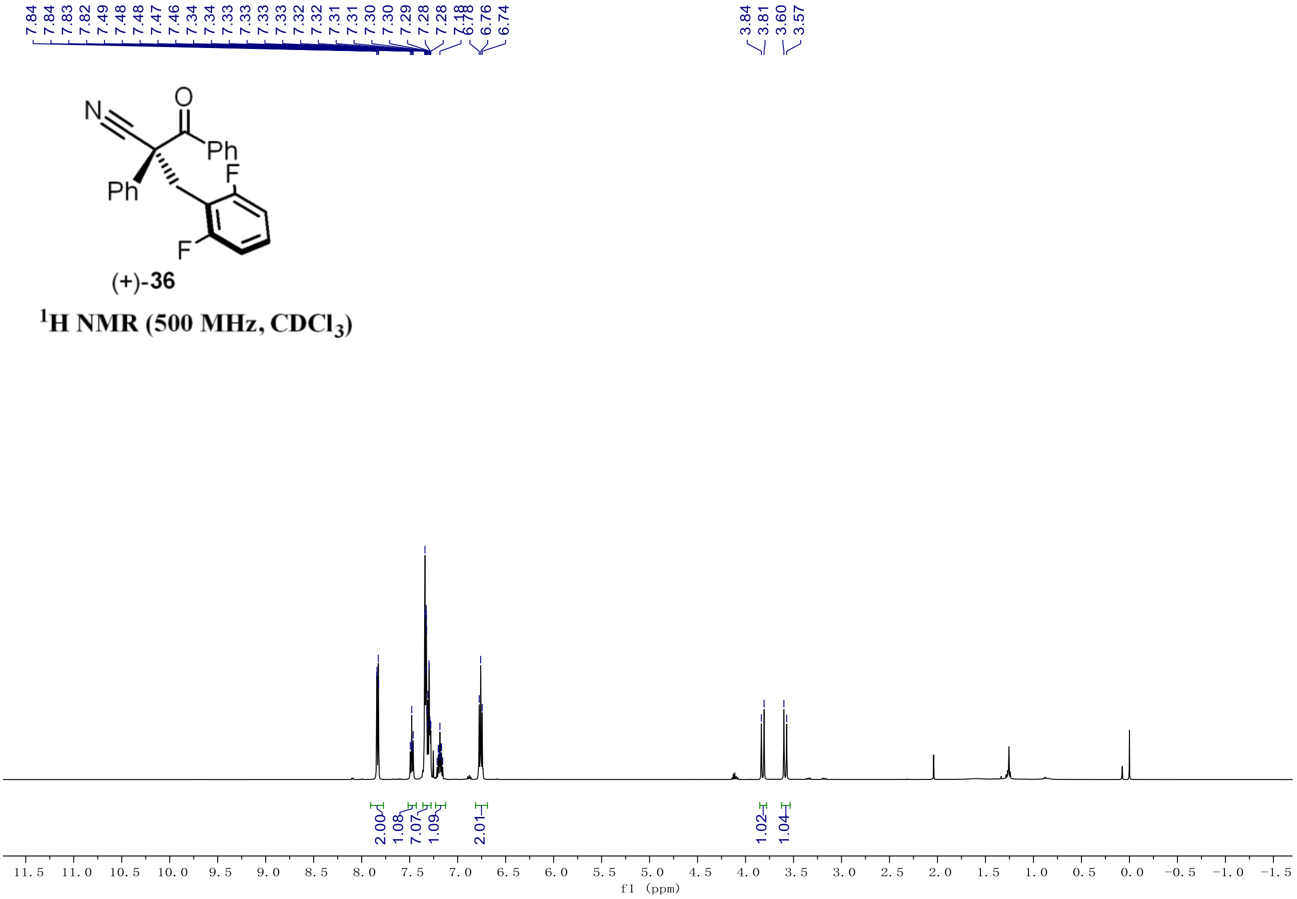

(+)-**36**

^{13}C NMR (126 MHz, $CDCl_3$)

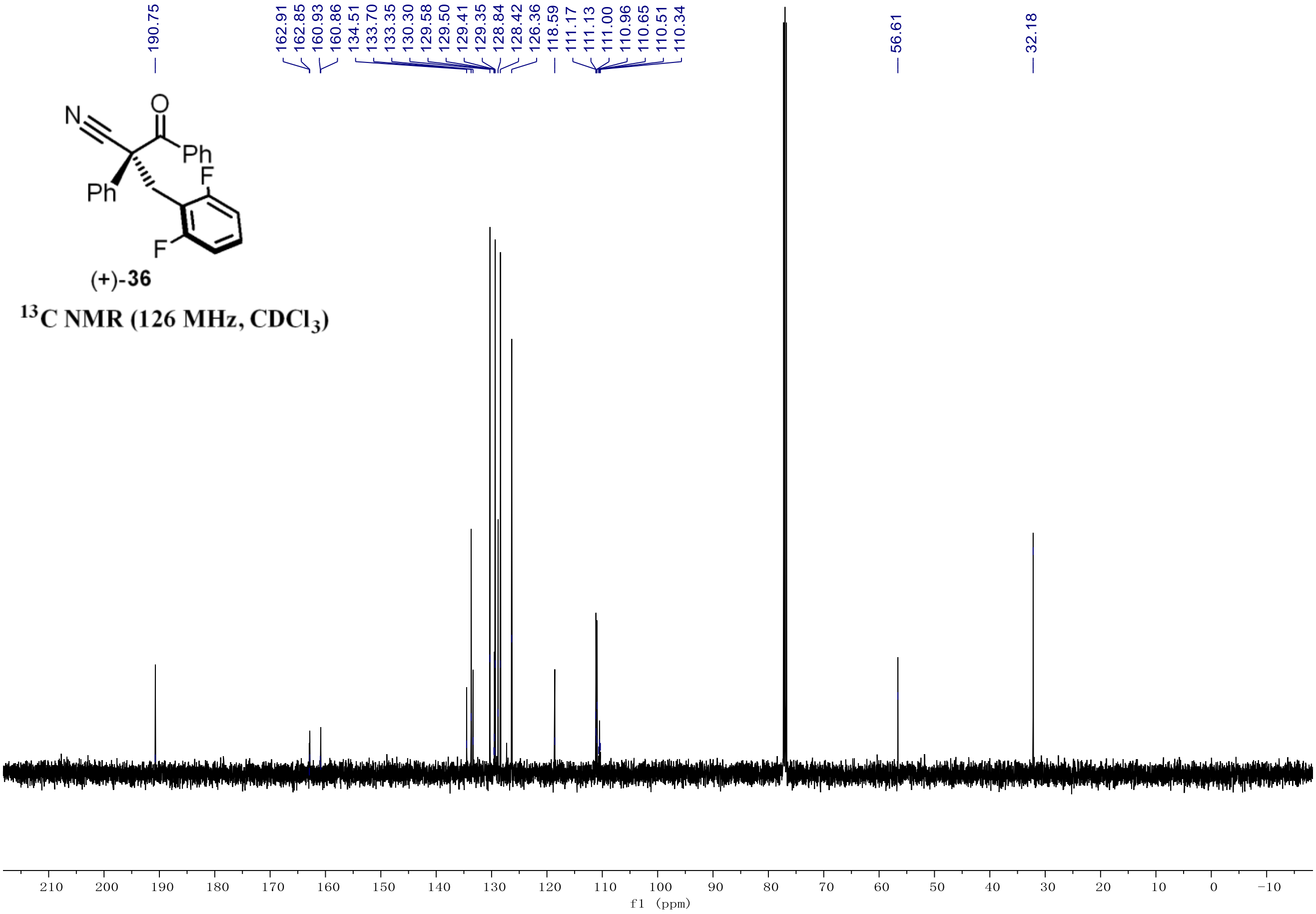

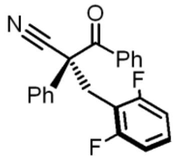
(+)-**36**

^{19}F NMR (471 MHz, CDCl$_3$)

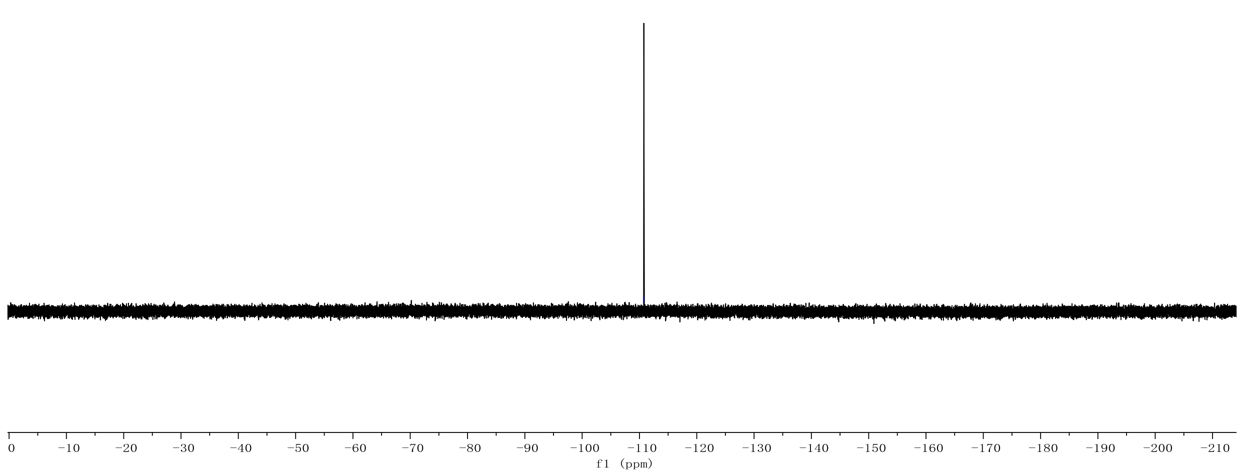

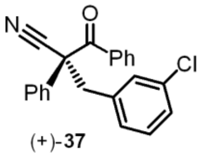

(+)-**37**

^{1}H NMR (400 MHz, $CDCl_3$)

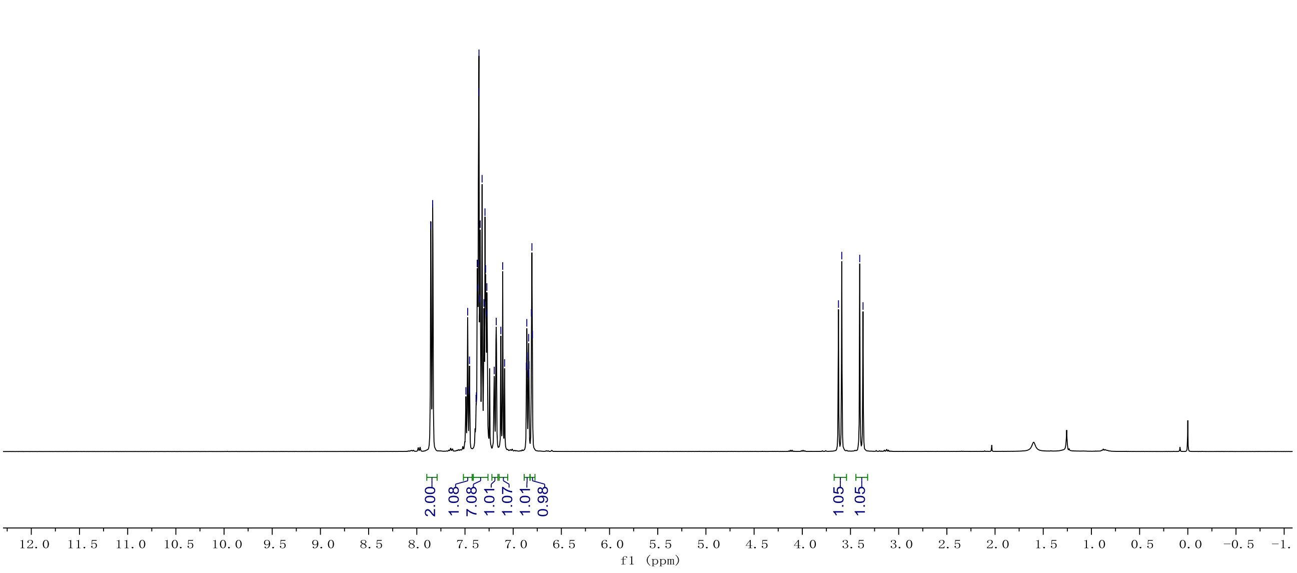

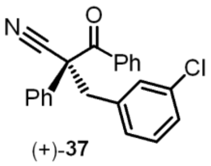

(+)-**37**

^{13}C NMR (101 MHz, $CDCl_3$)

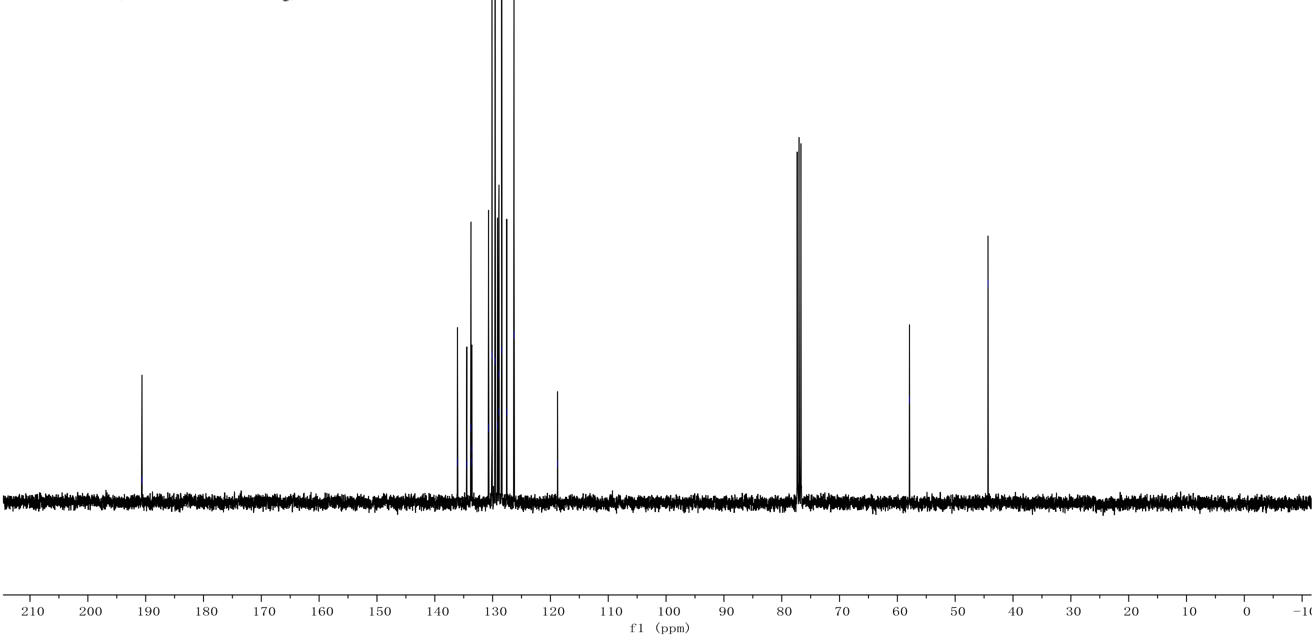

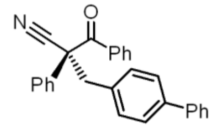

(+)-**38**

^{1}H NMR (400 MHz, CDCl$_3$)

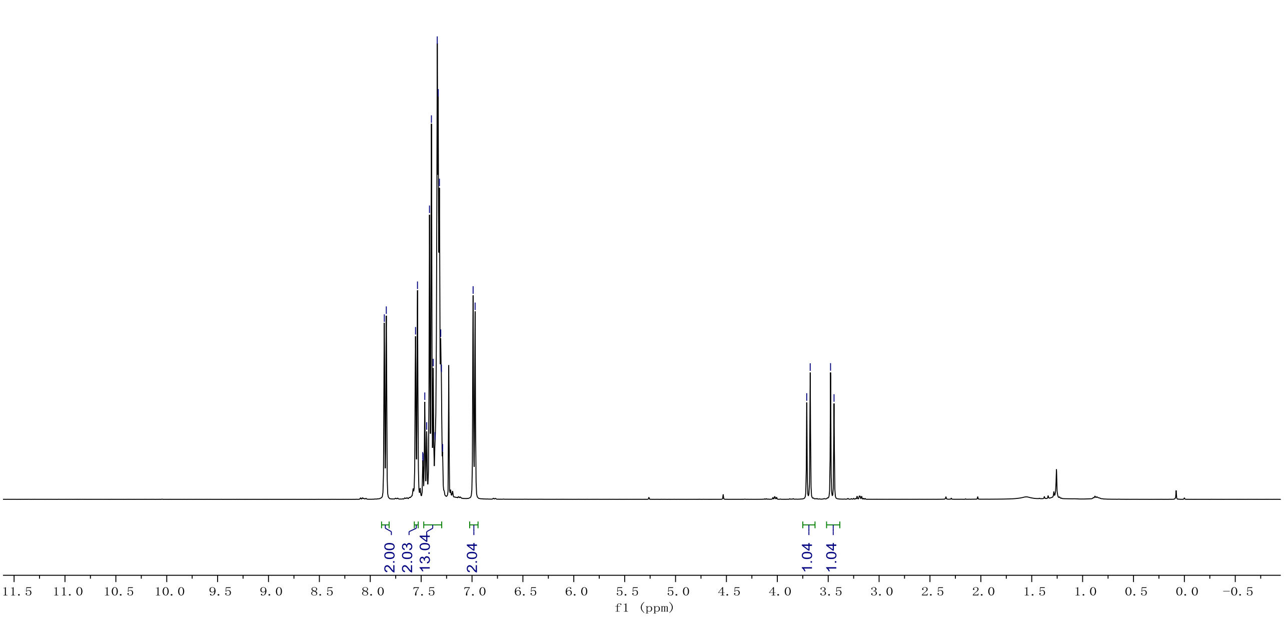

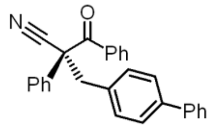

(+)-**38**

^{13}C NMR (101 MHz, CDCl$_3$)

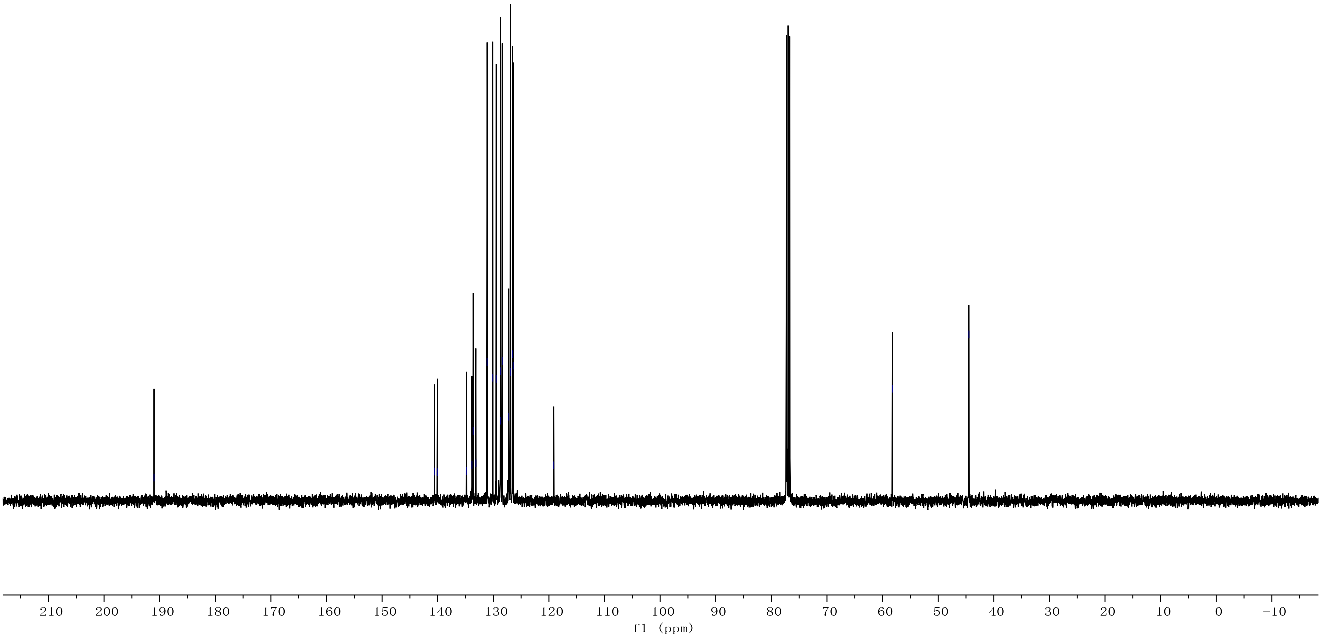

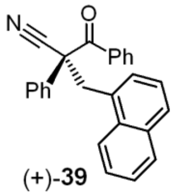

(+)-**39**

^{1}H NMR (400 MHz, CDCl$_3$)

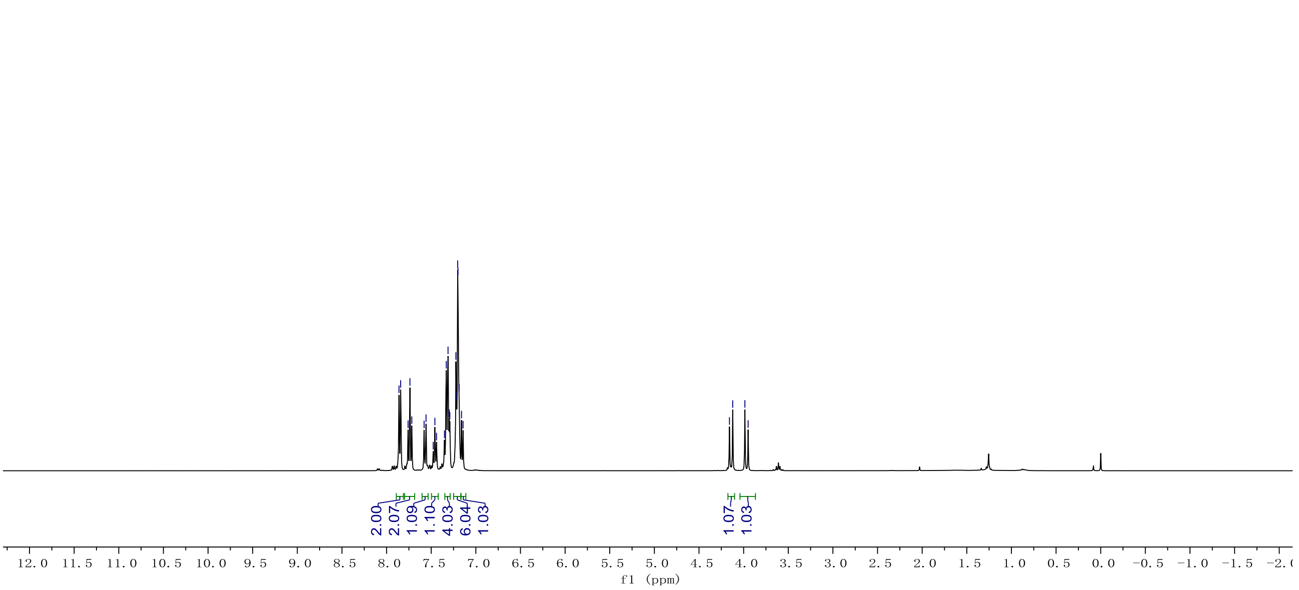

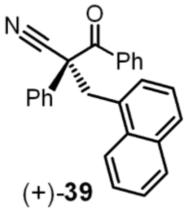

(+)-**39**

^{13}C NMR (101 MHz, CDCl$_3$)

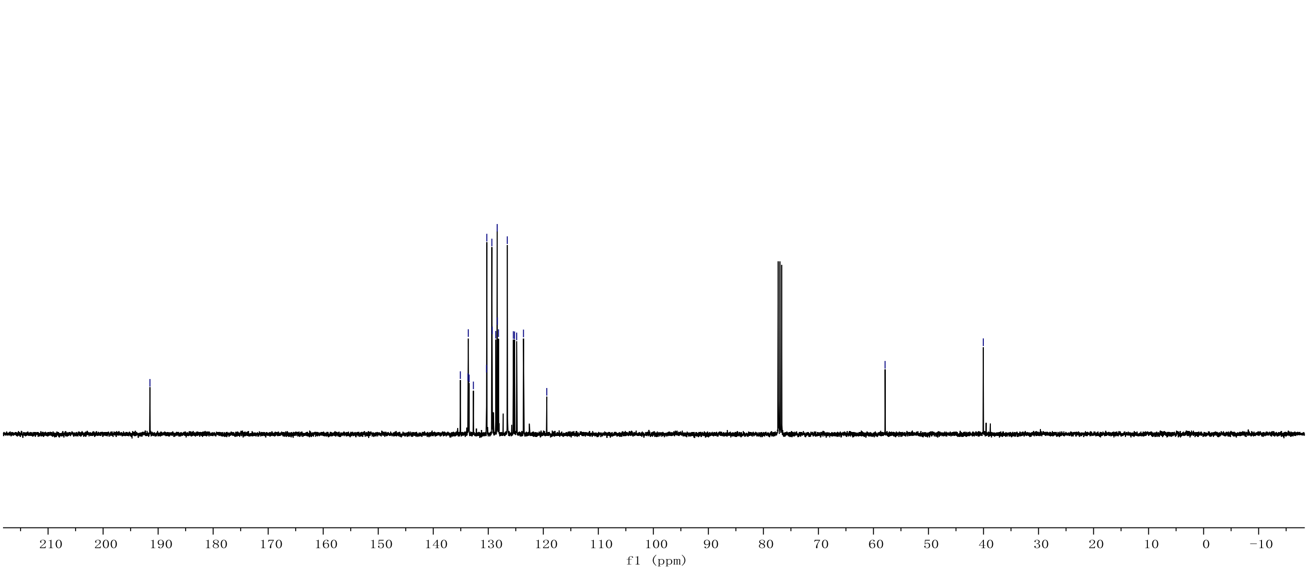

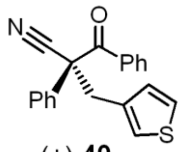

(+)-**40**

^{1}H NMR (400 MHz, $CDCl_3$)

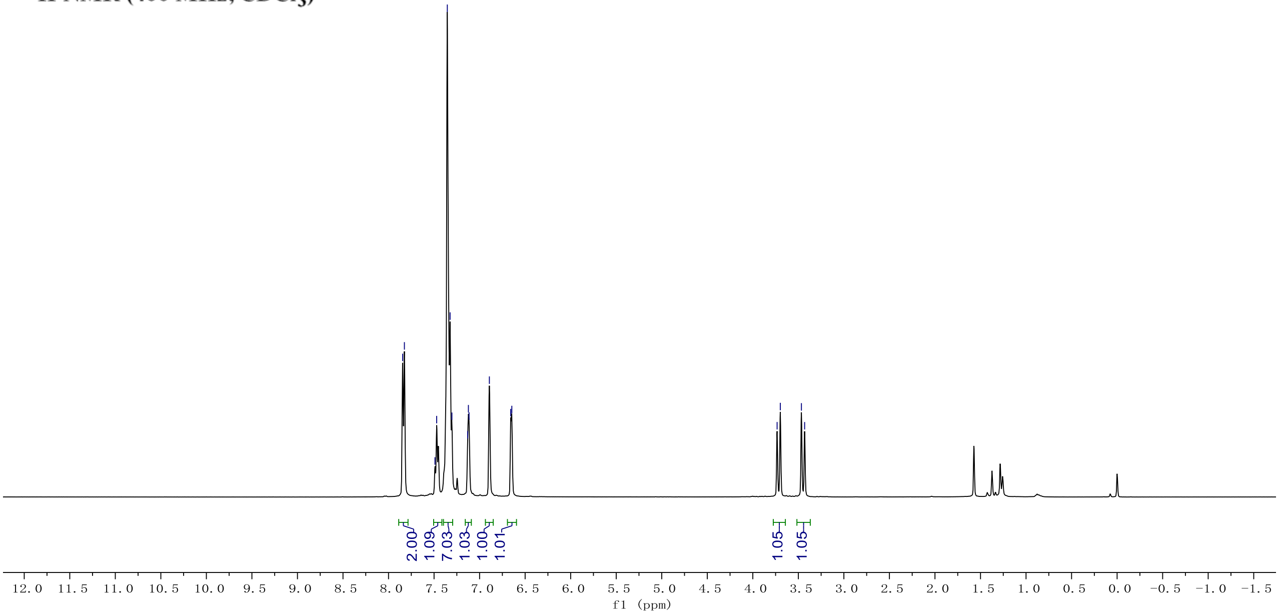

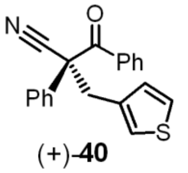

(+)-**40**

^{13}C NMR (101 MHz, $CDCl_3$)

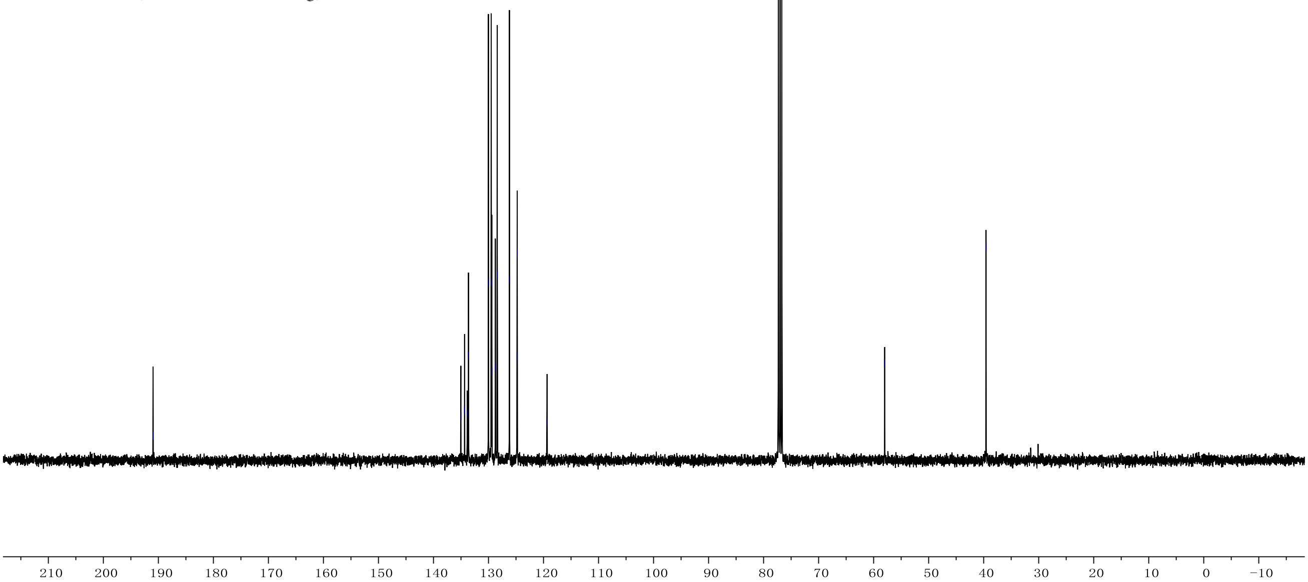

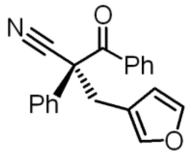

(+)-**41**

^{1}H NMR (500 MHz, CDCl$_3$)

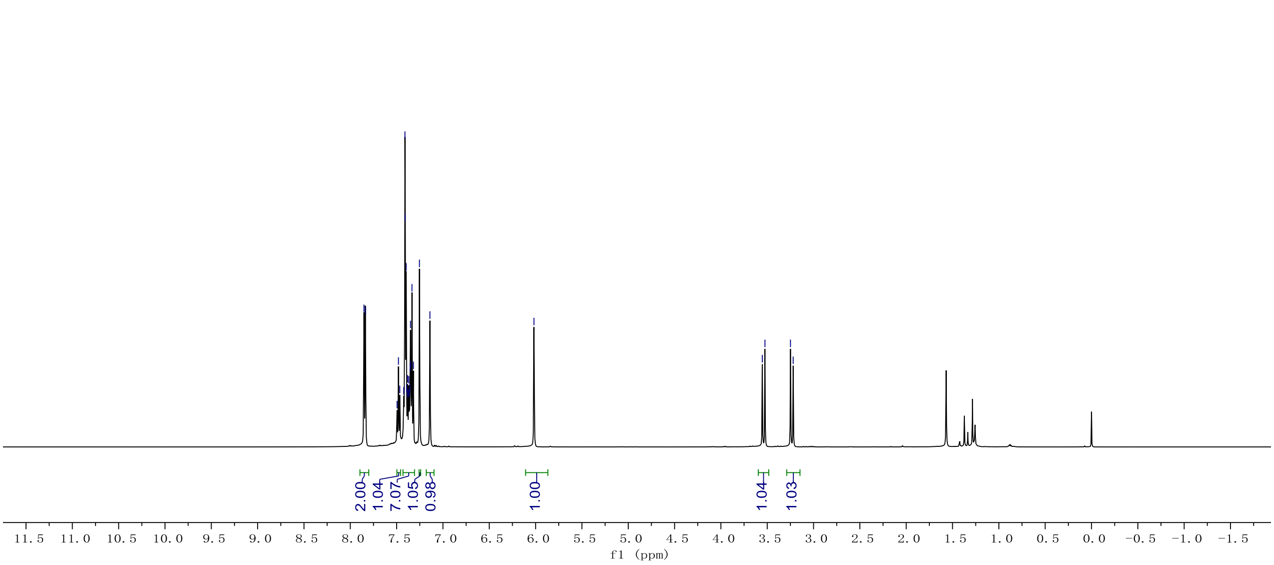

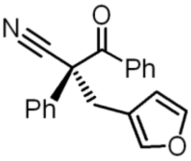

(+)-**41**

^{13}C NMR (126 MHz, CDCl$_3$)

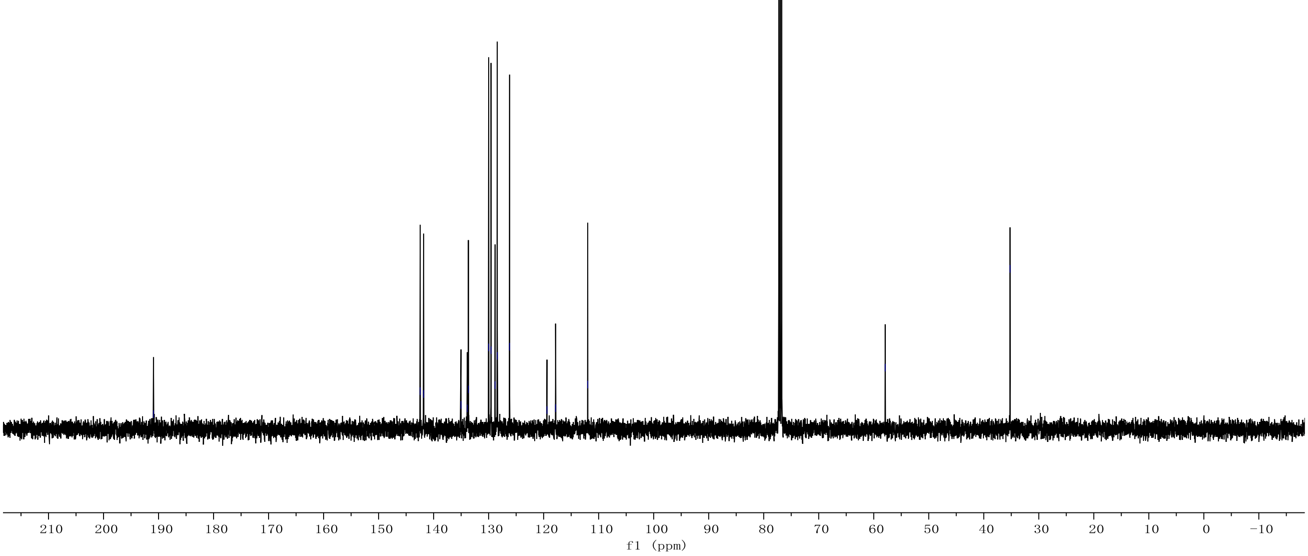

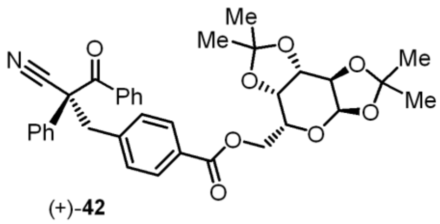

(+)-**42**

^{1}H NMR (400 MHz, $CDCl_3$)

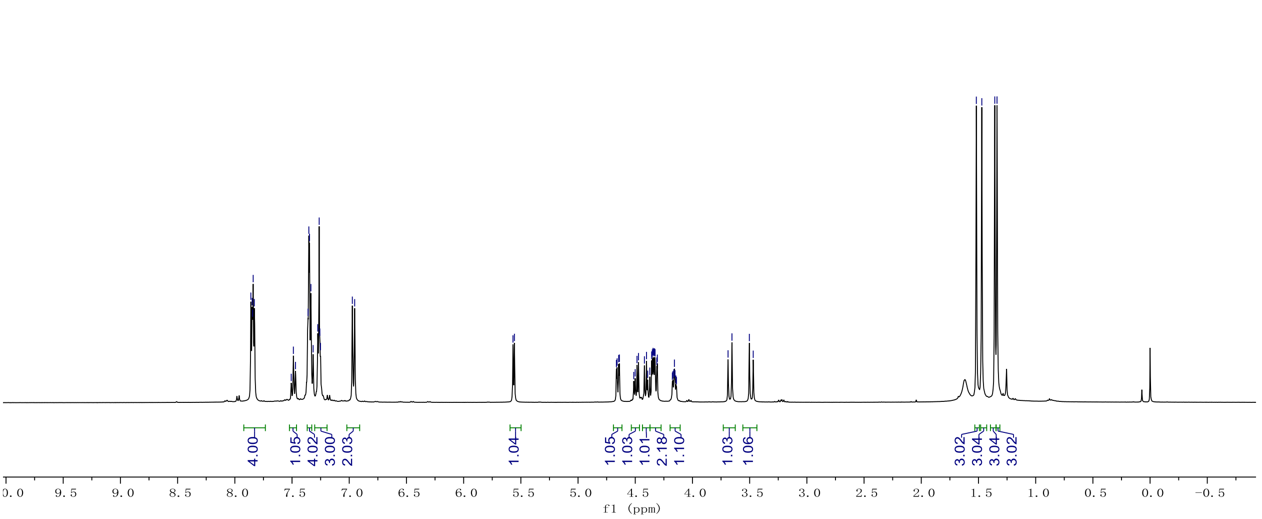

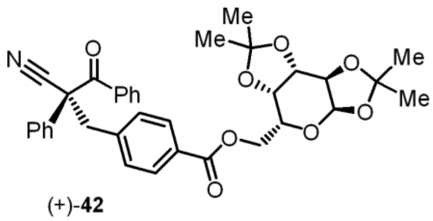

(+)-**42**

^{13}C NMR (101 MHz, $CDCl_3$)

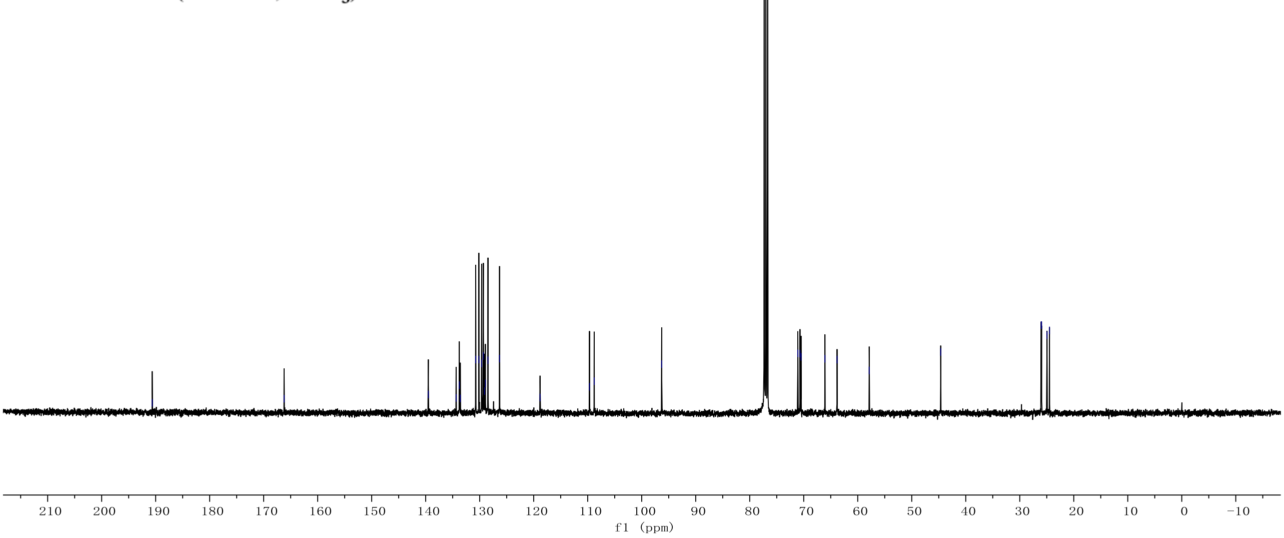

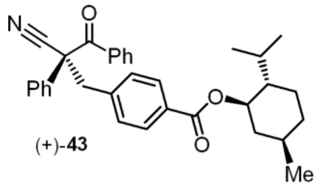

(+)-**43**

^{1}H NMR (400 MHz, CDCl$_3$)

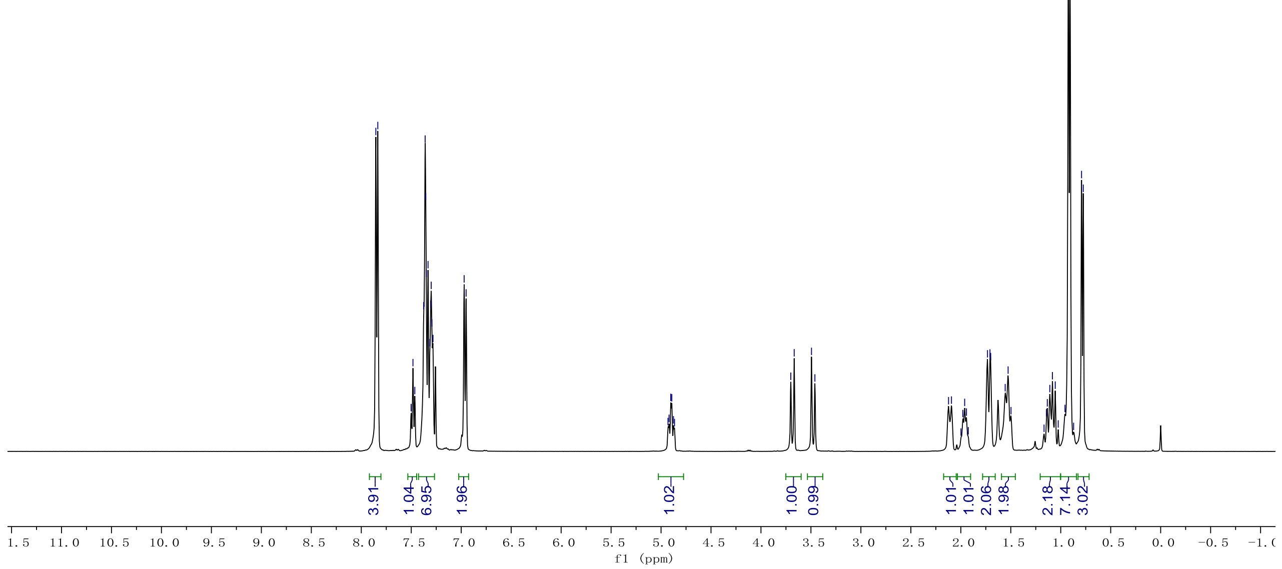

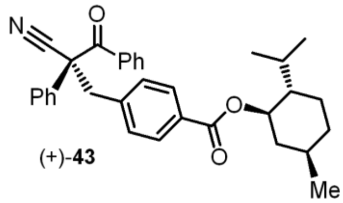

(+)-**43**

^{13}C NMR (101 MHz, CDCl$_3$)

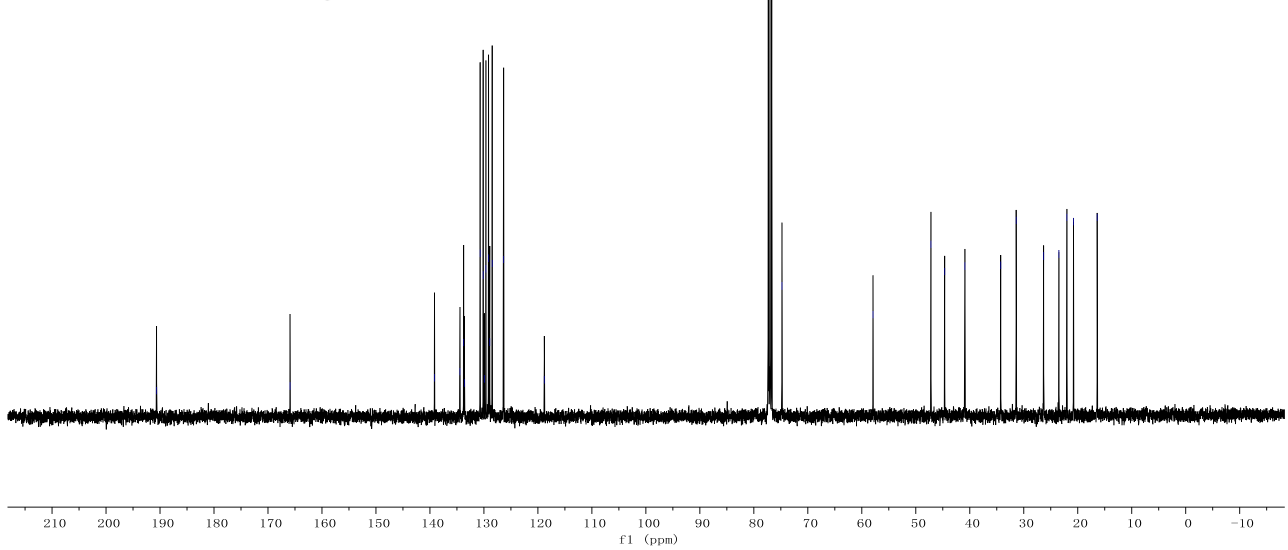

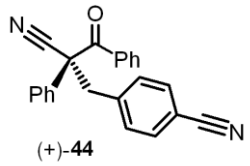

(+)-**44**

^{1}H NMR (400 MHz, $CDCl_3$)

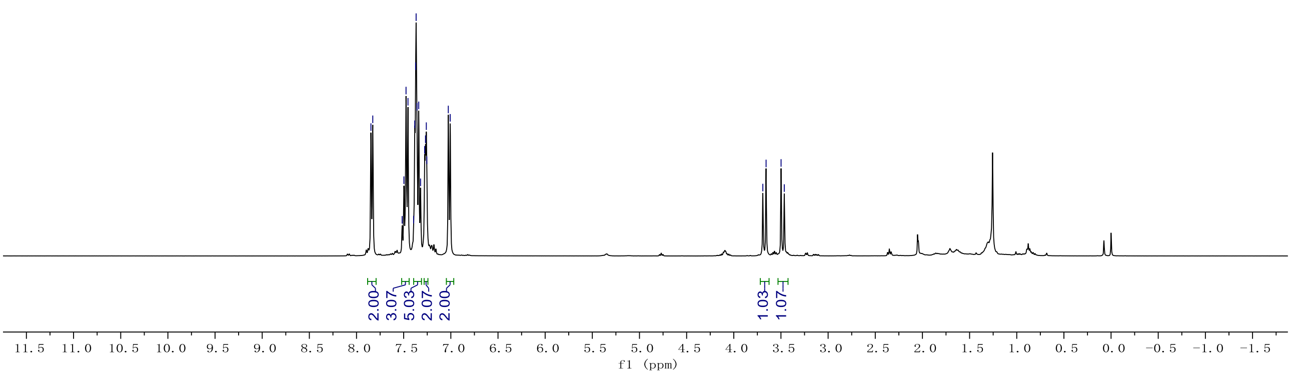

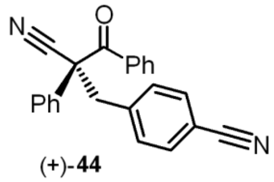

(+)-**44**

^{13}C NMR (101 MHz, $CDCl_3$)

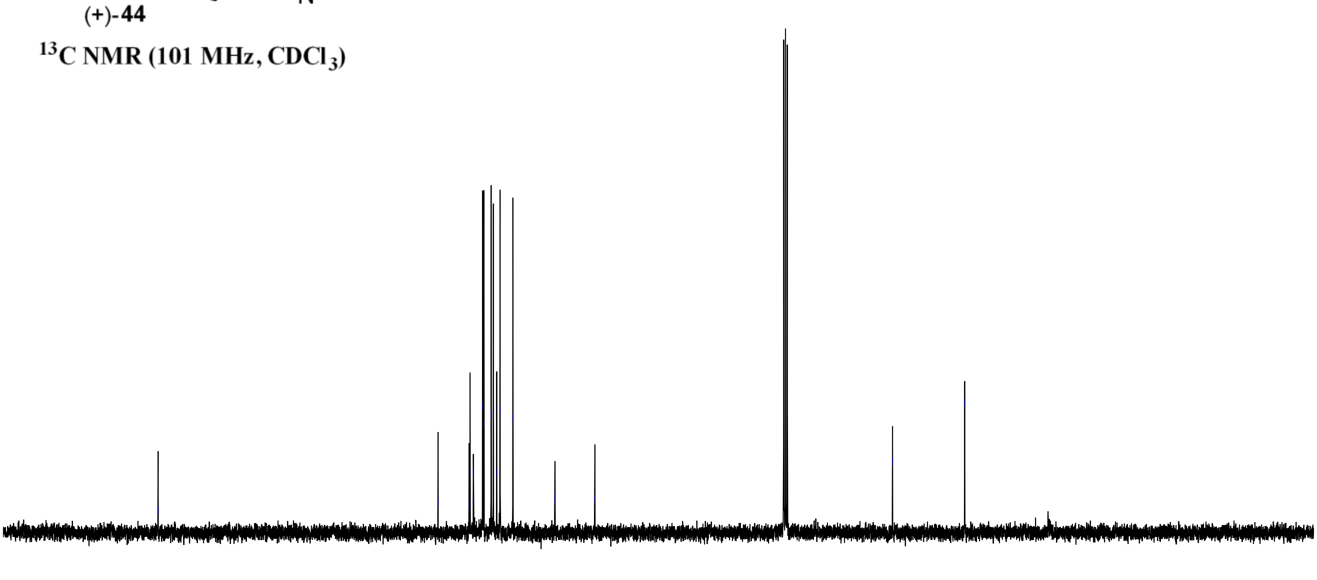

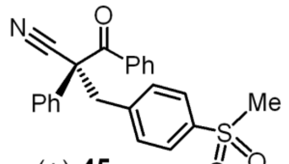

(+)-**45**

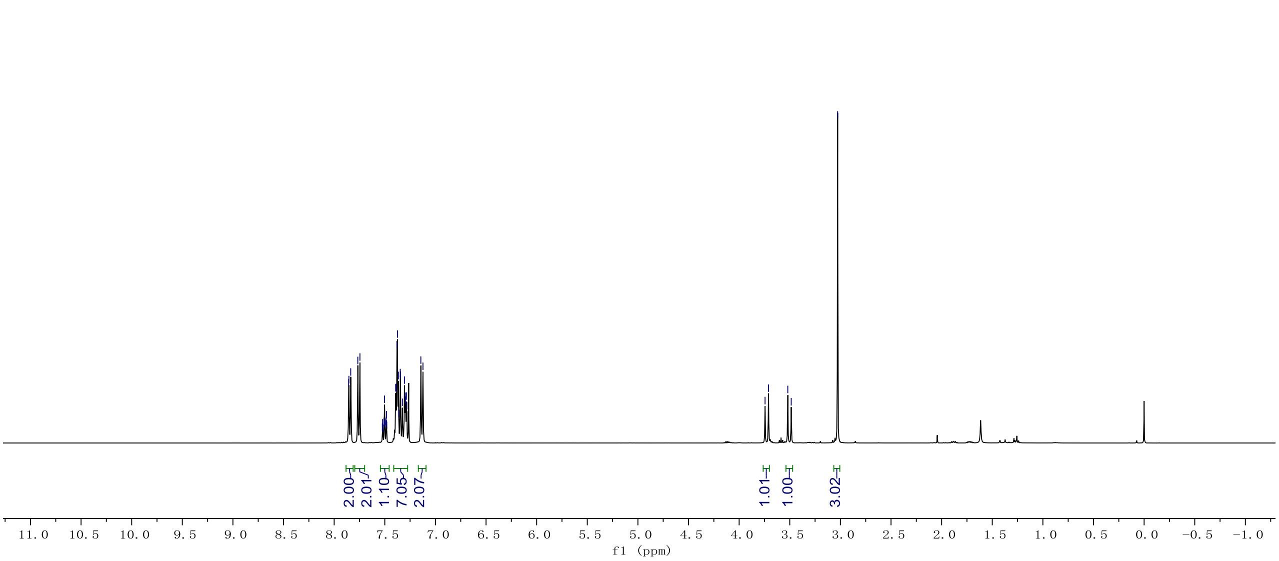

^{1}H NMR (400 MHz, $CDCl_3$)

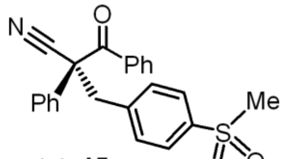

(+)-**45**

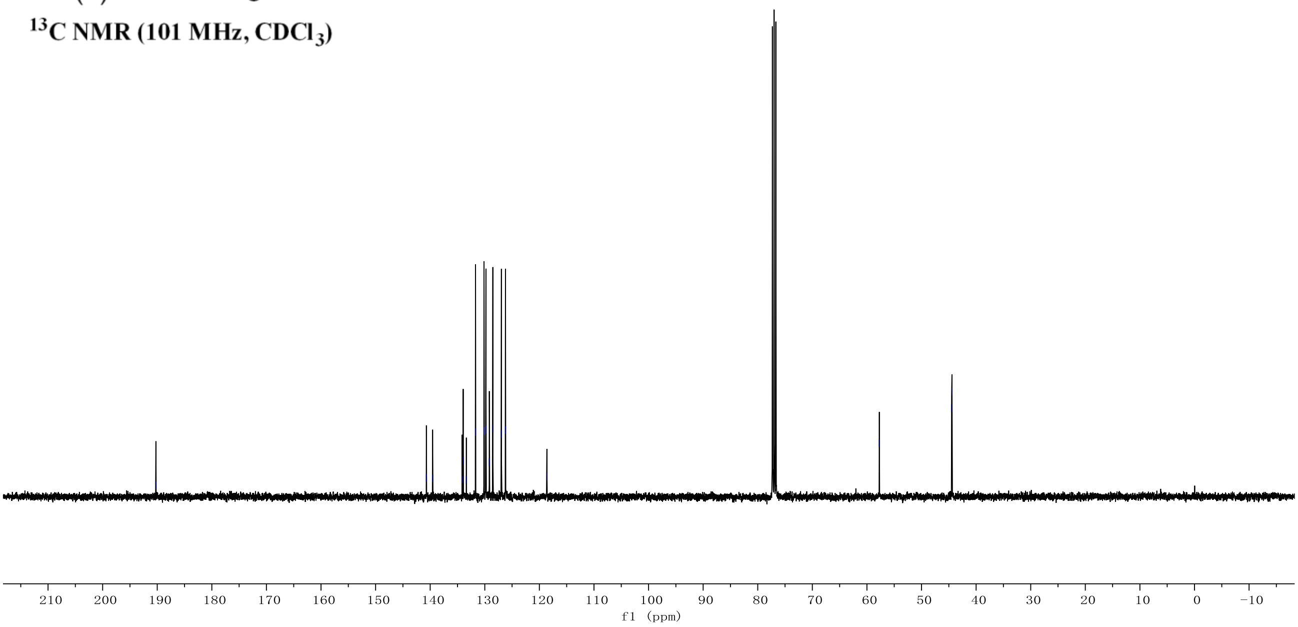

^{13}C NMR (101 MHz, $CDCl_3$)

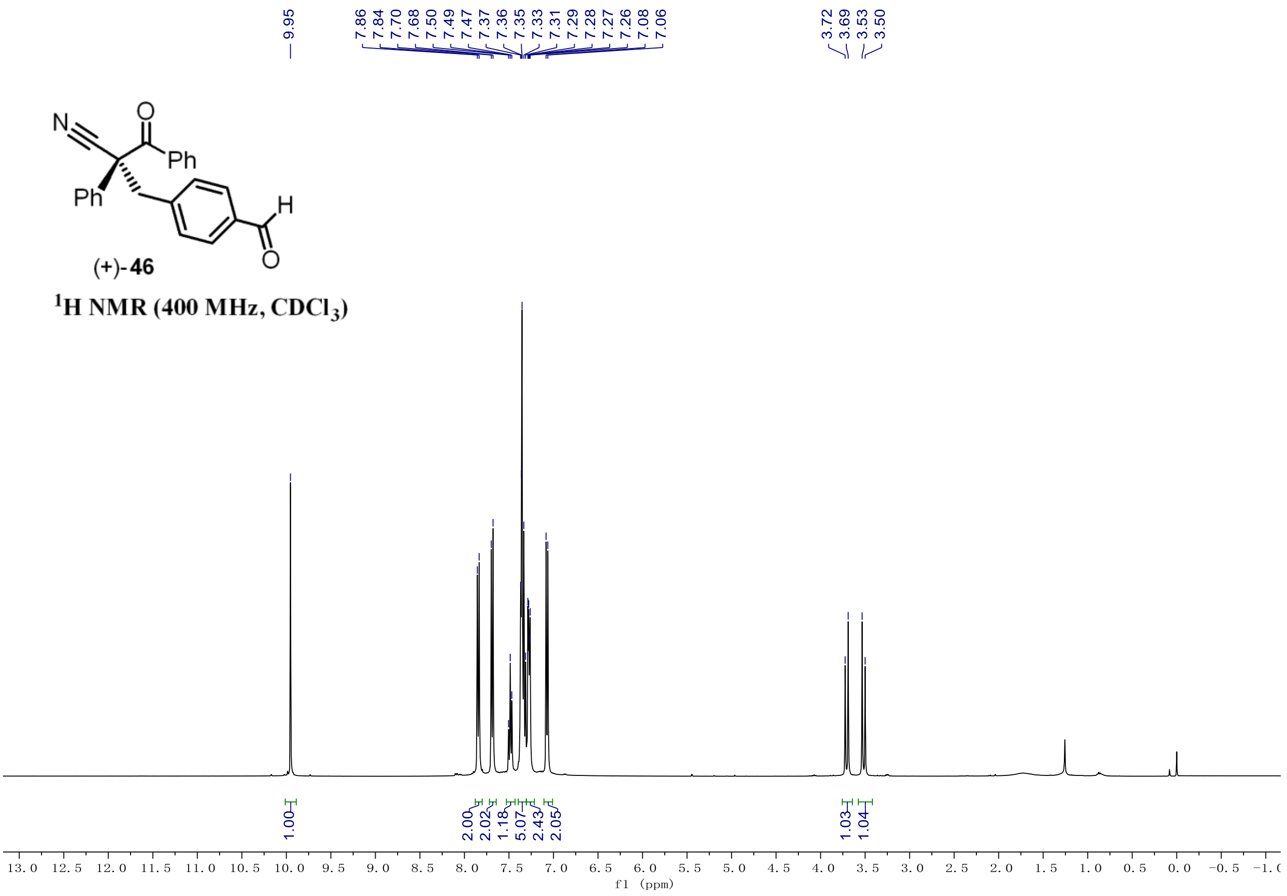

(+)-46
^{1}H NMR (400 MHz, CDCl$_3$)
9.95
7.86 7.84 7.70 7.68 7.50 7.49 7.47 7.37 7.36 7.35 7.33 7.31 7.29 7.28 7.27 7.26 7.08 7.06
3.72 3.69 3.53 3.50
1.00 2.00 2.02 1.18 5.07 2.43 2.05 1.03 1.04
f1 (ppm)

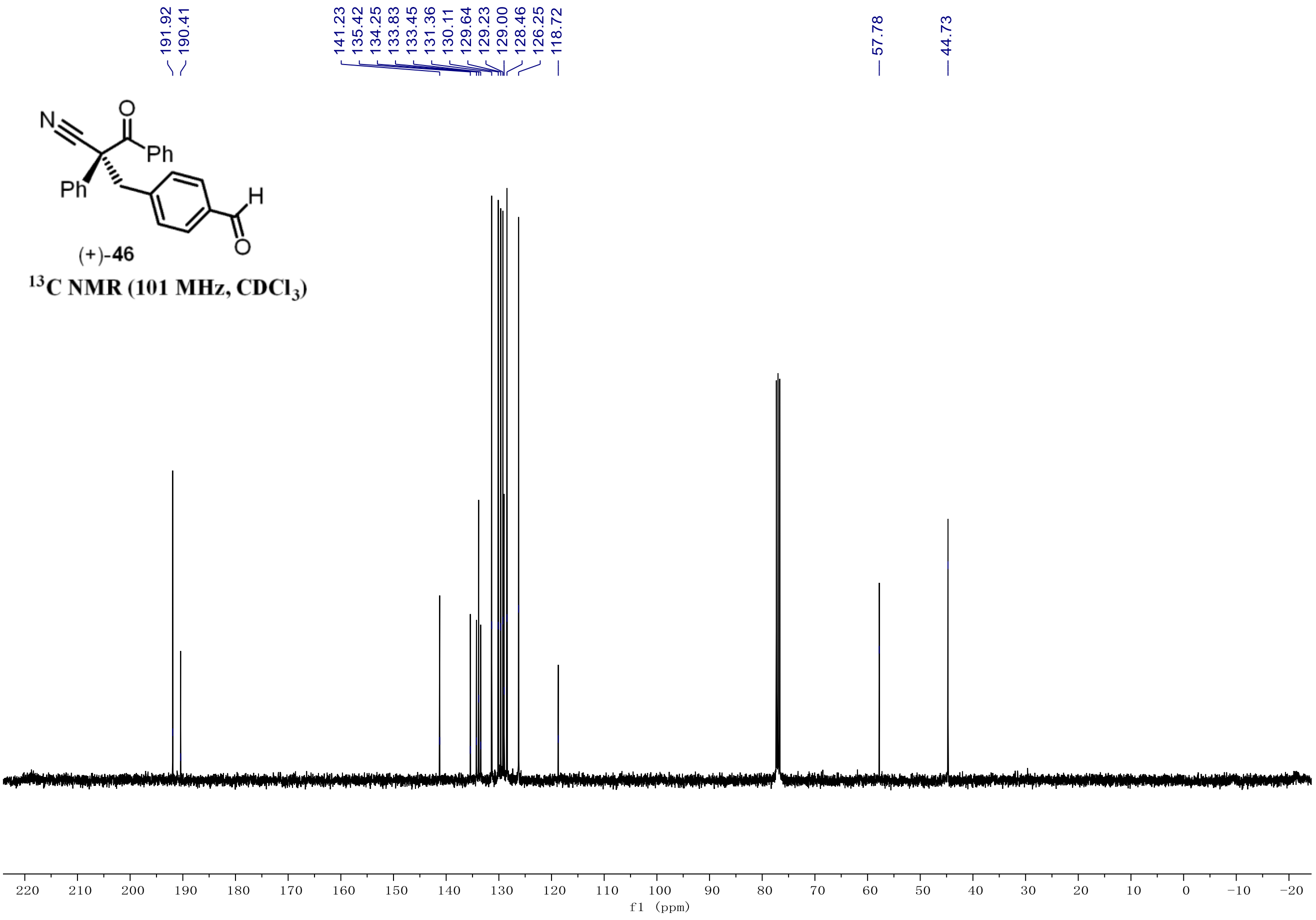

(+)-46
^{13}C NMR (101 MHz, CDCl$_3$)
191.92 190.41
141.23 135.42 134.25 133.83 133.45 131.36 130.11 129.64 129.23 129.00 128.46 126.25 118.72
57.78
44.73
f1 (ppm)

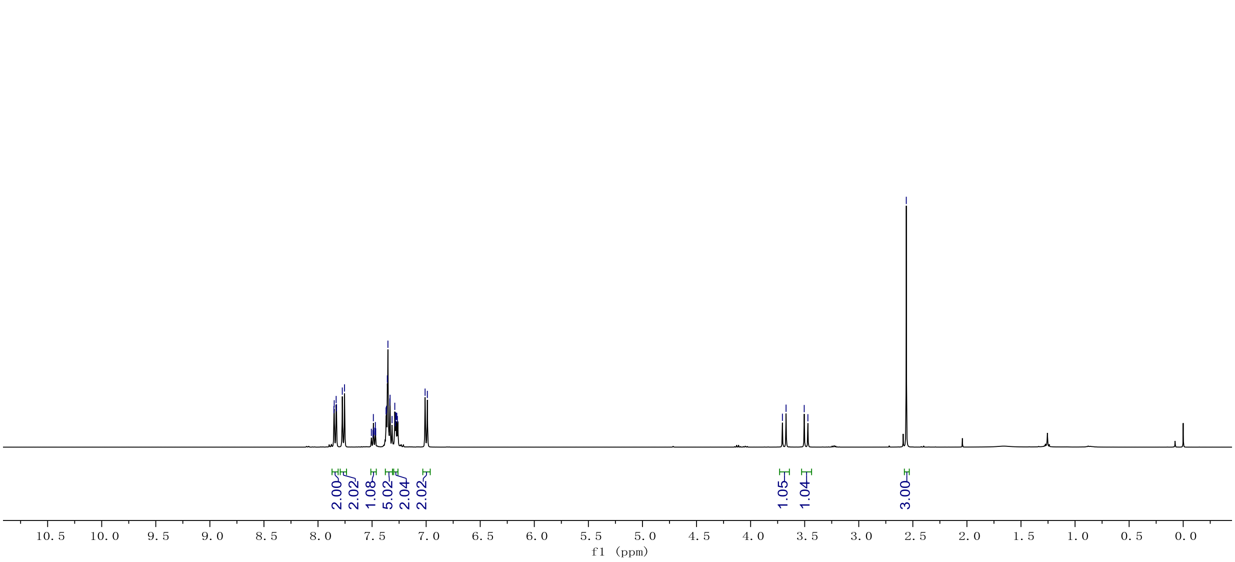

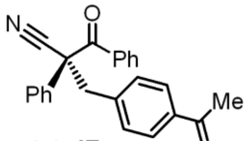

(+)-**47**

^{1}H NMR (400 MHz, CDCl$_3$)

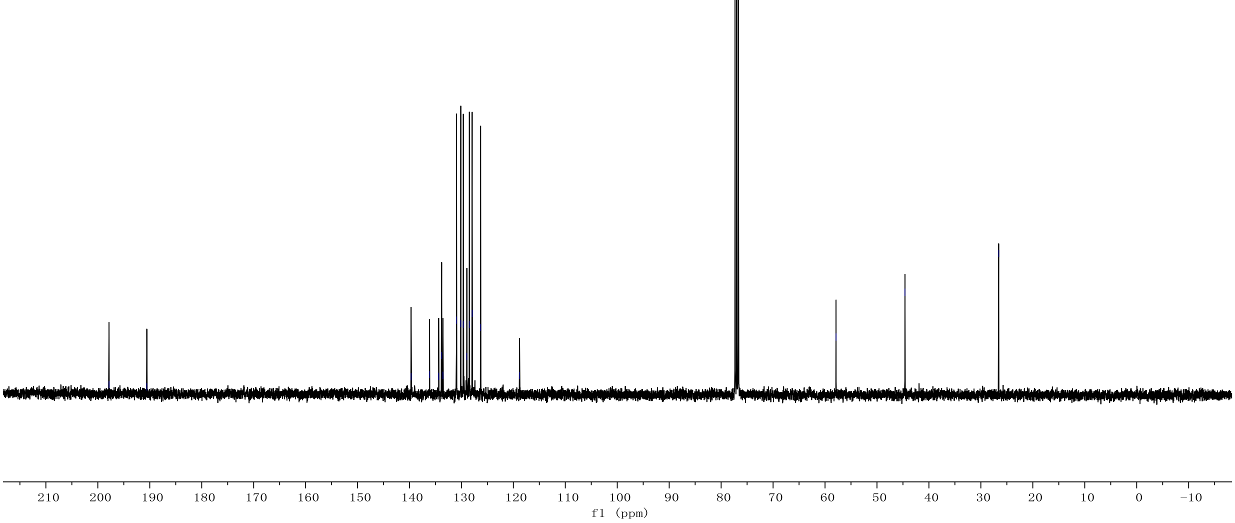

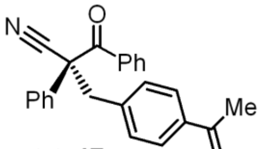

(+)-**47**

^{13}C NMR (101 MHz, CDCl$_3$)

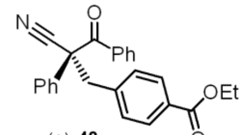

(+)-**48**

^{1}H NMR (400 MHz, CDCl$_3$)

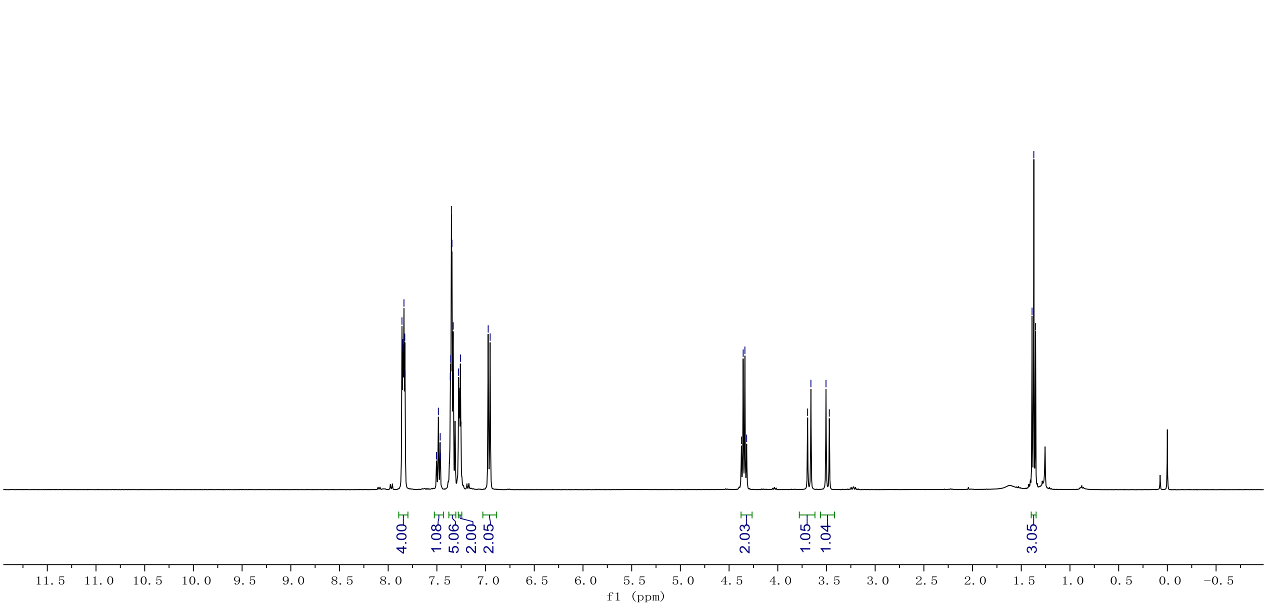

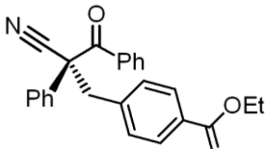

(+)-**48**

^{13}C NMR (101 MHz, CDCl$_3$)

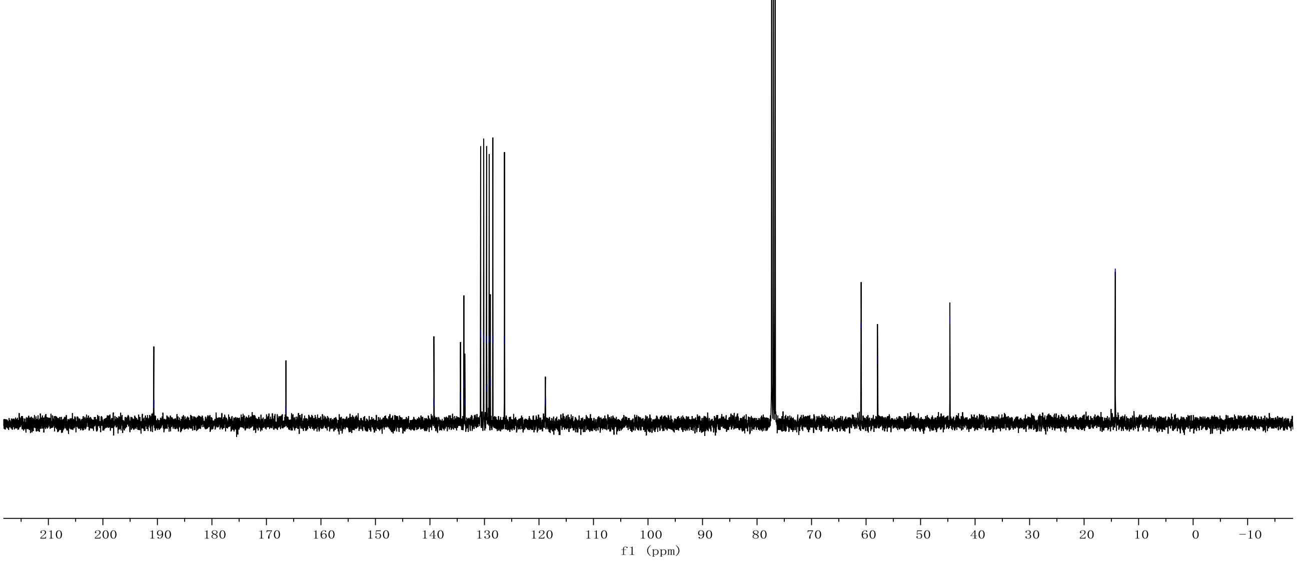

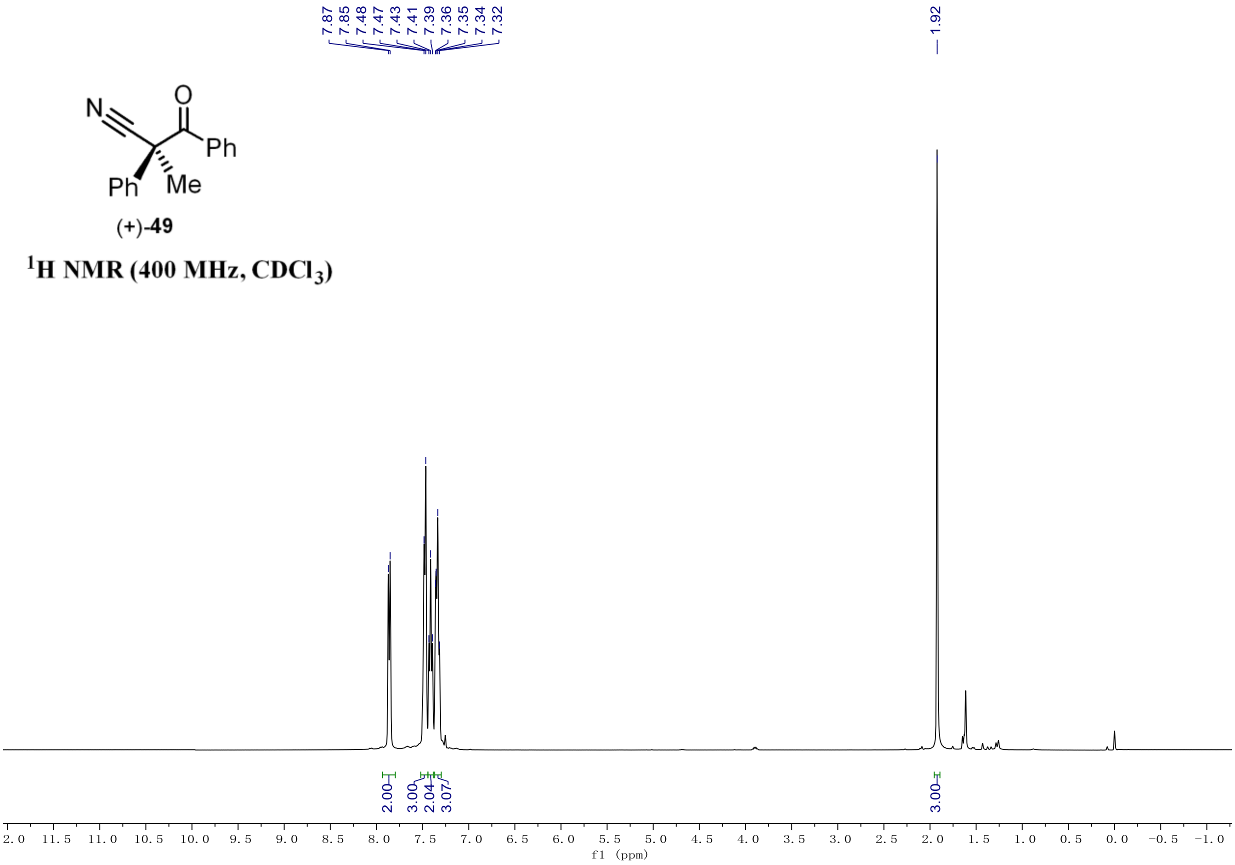

(+)-**49**

^{1}H NMR (400 MHz, $CDCl_3$)

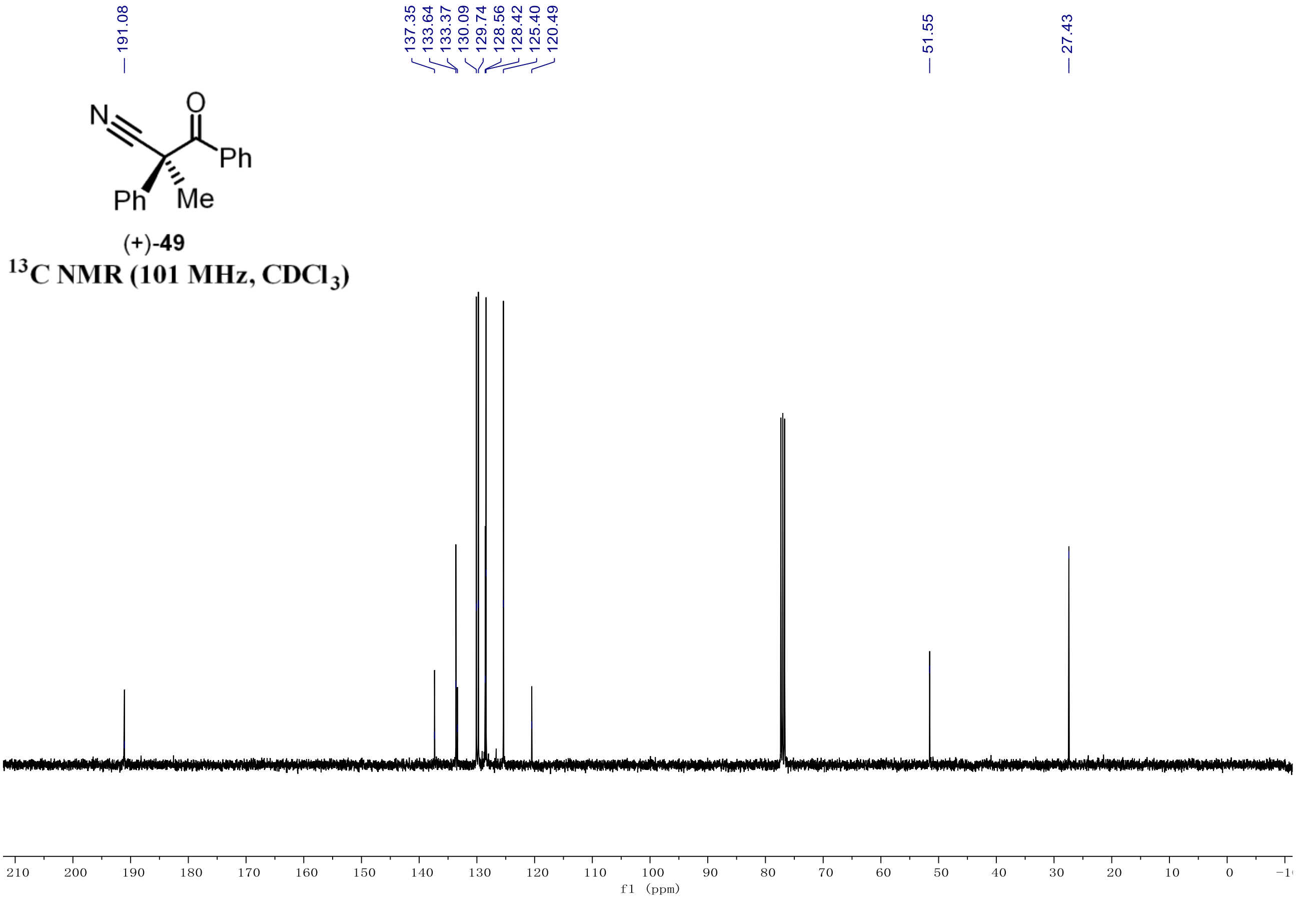

(+)-**49**

^{13}C NMR (101 MHz, $CDCl_3$)

(+)-**50**

^{1}H NMR (600 MHz, $CDCl_3$)

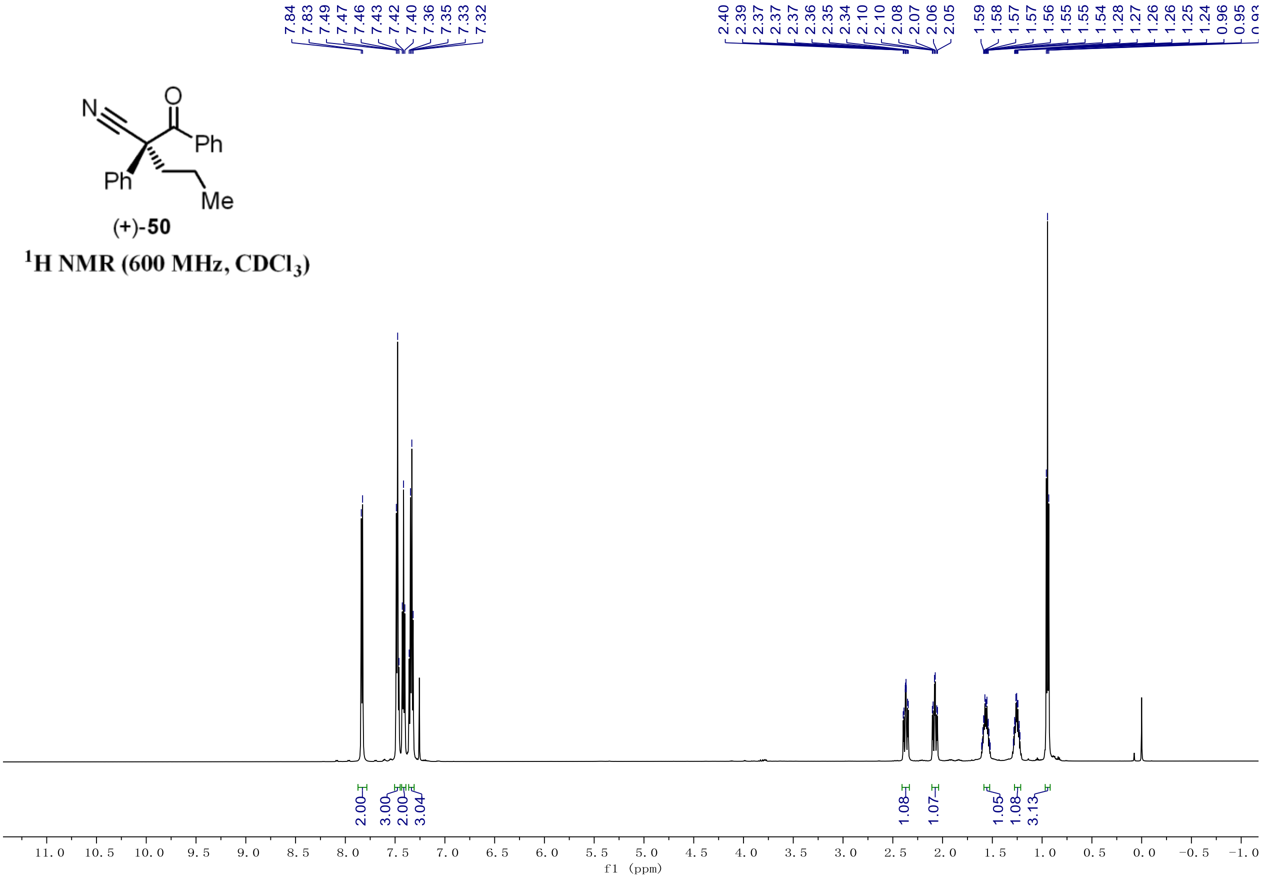

(+)-**50**

^{13}C NMR (151 MHz, $CDCl_3$)

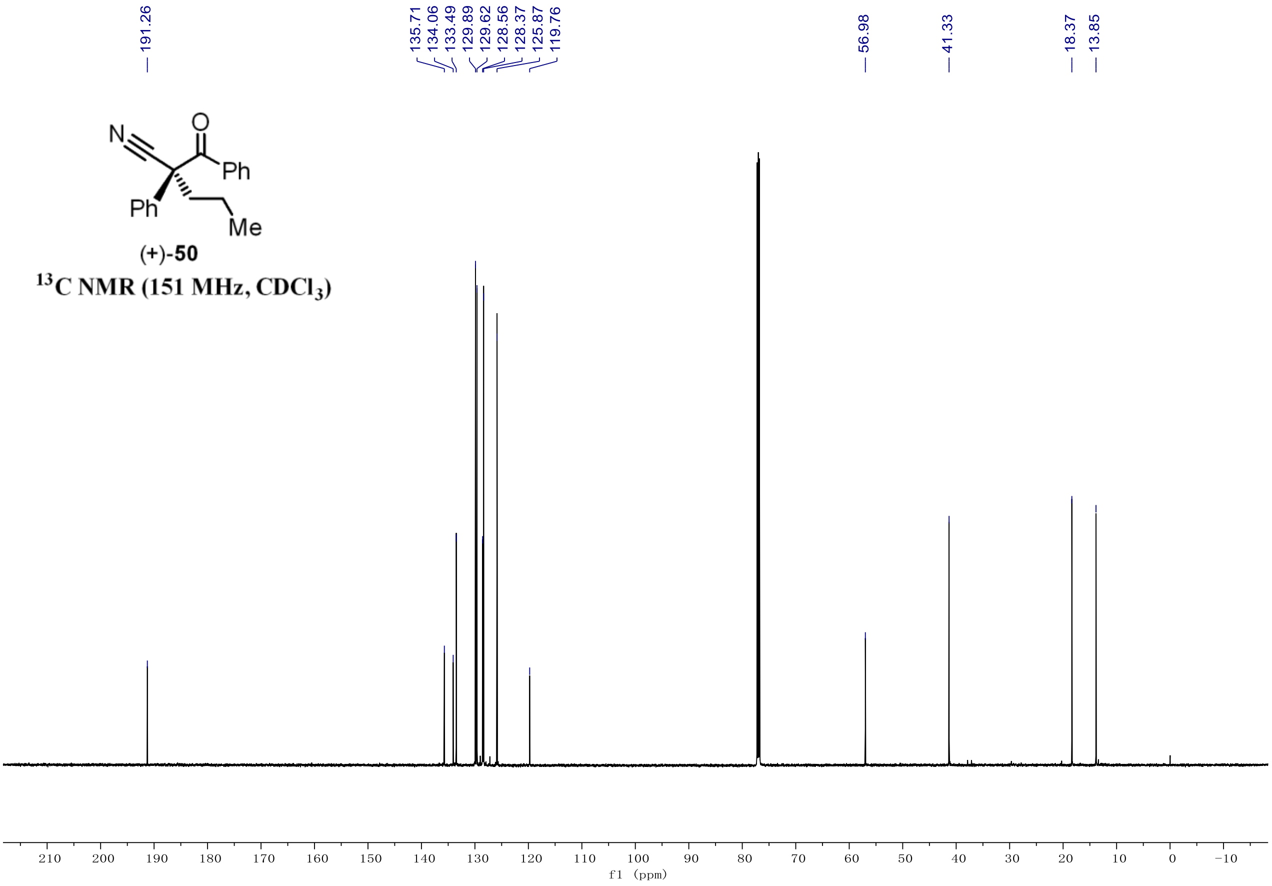

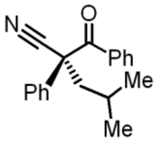

(+)-**51**

^{1}H NMR (400 MHz, $CDCl_3$)

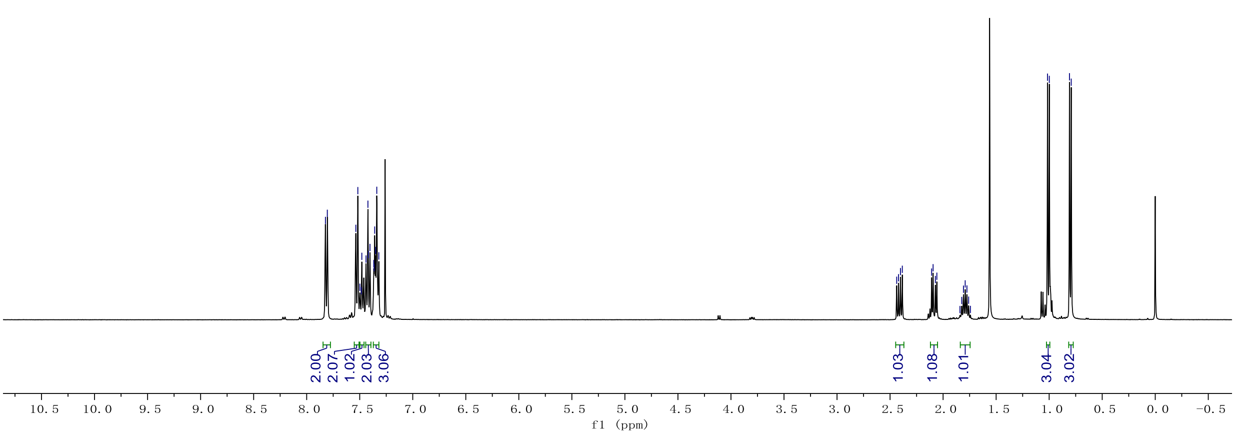

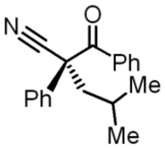

(+)-**51**

^{13}C NMR (101 MHz, $CDCl_3$)

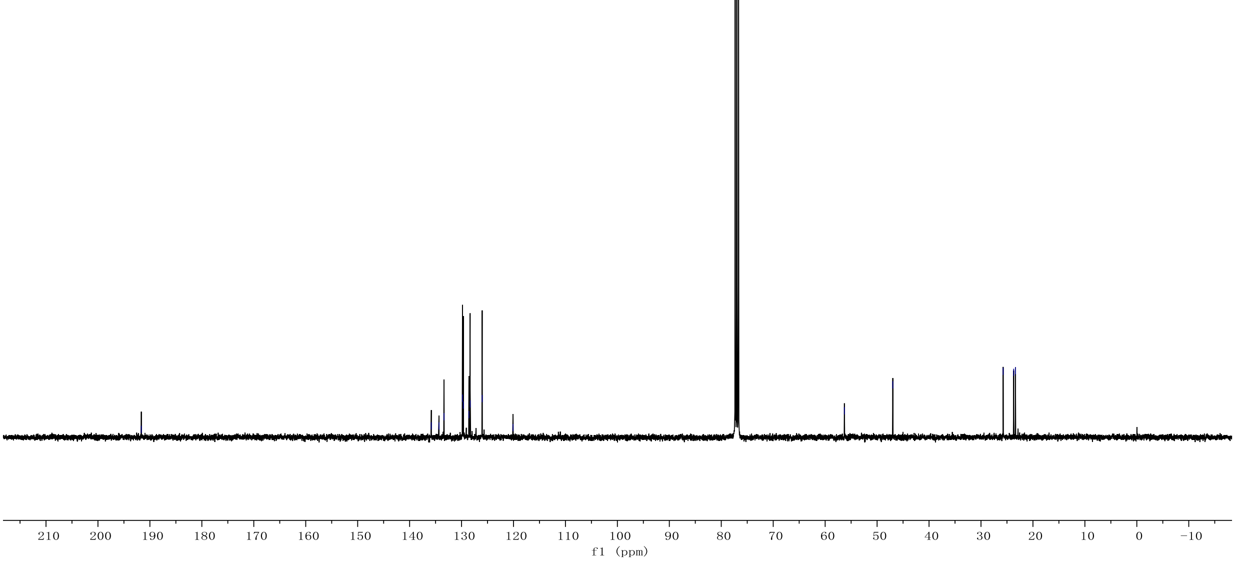

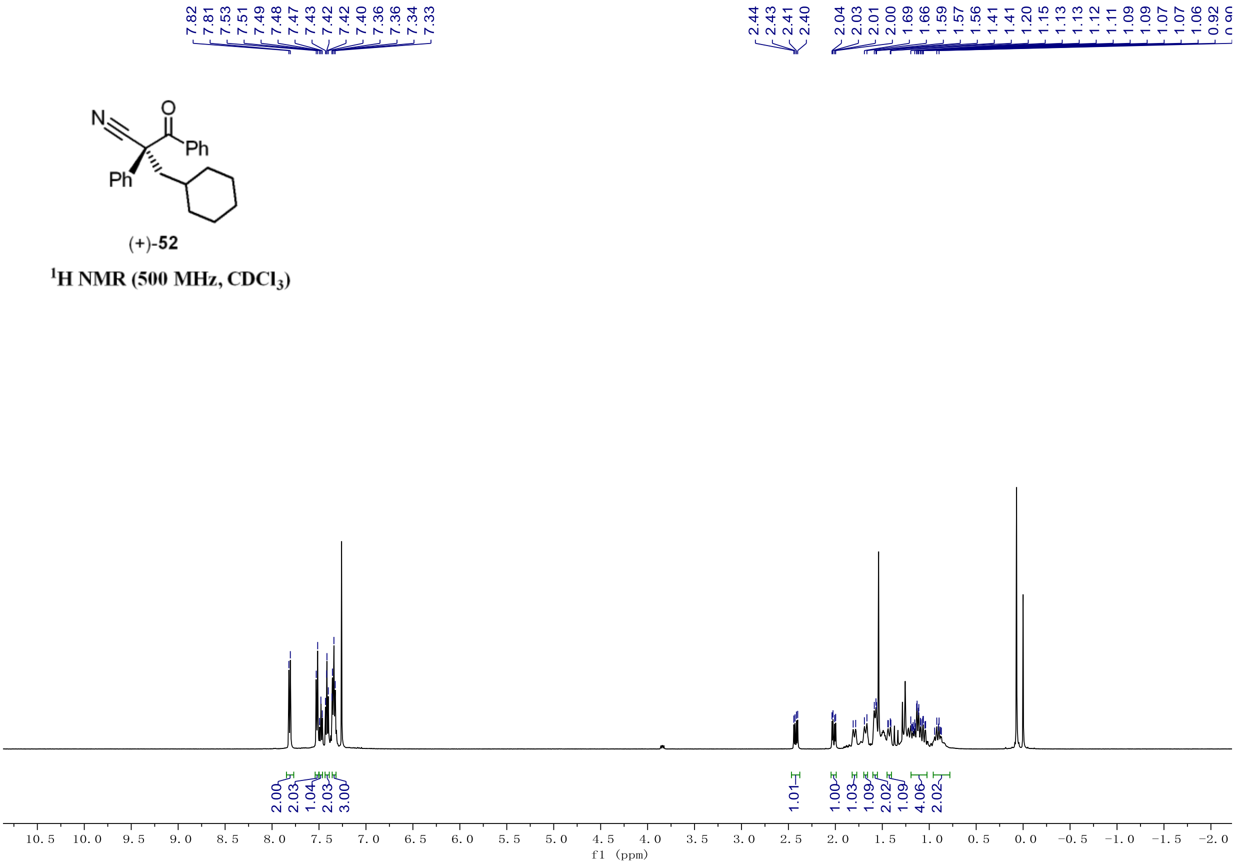

(+)-**52**

^{1}H NMR (500 MHz, $CDCl_3$)

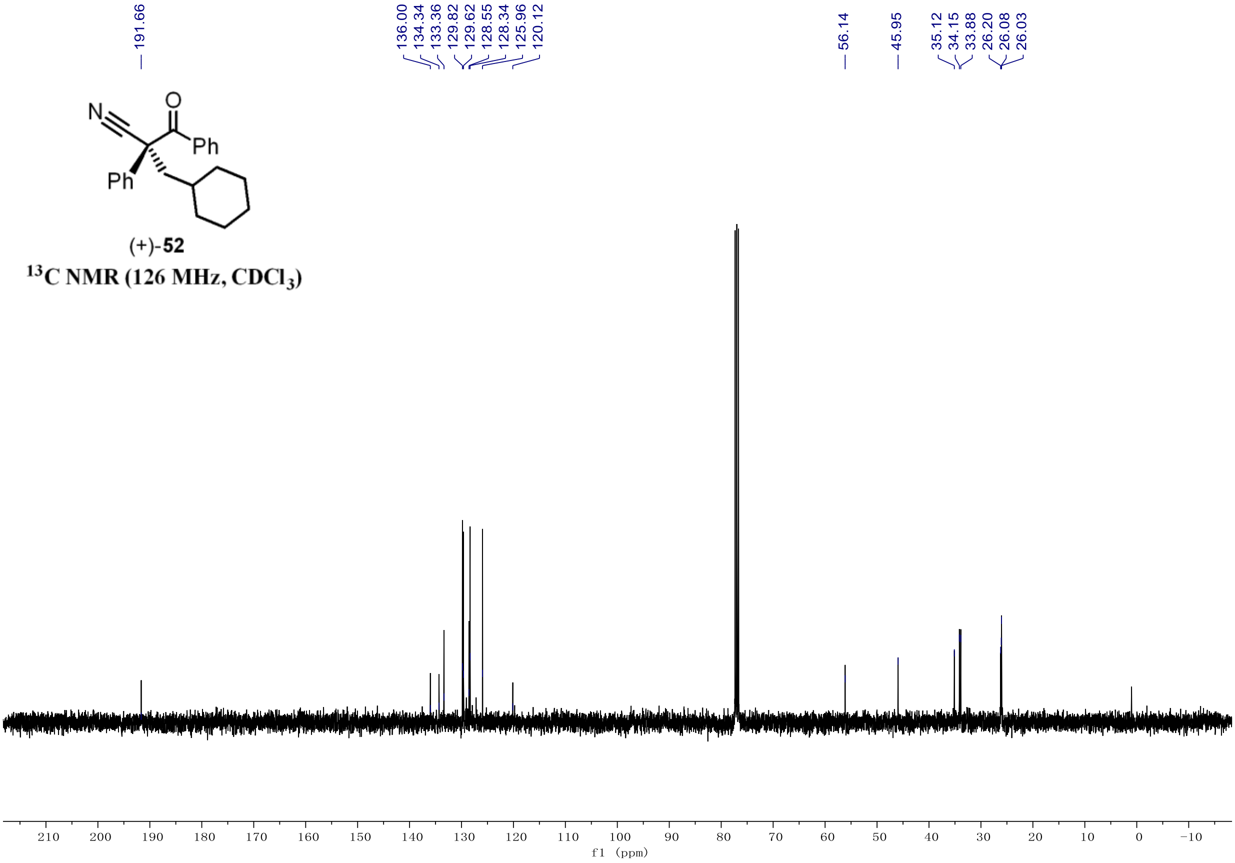

(+)-**52**

^{13}C NMR (126 MHz, $CDCl_3$)

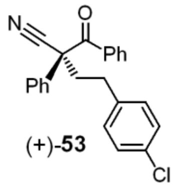

(+)-**53**

^{1}H NMR (400 MHz, $CDCl_3$)

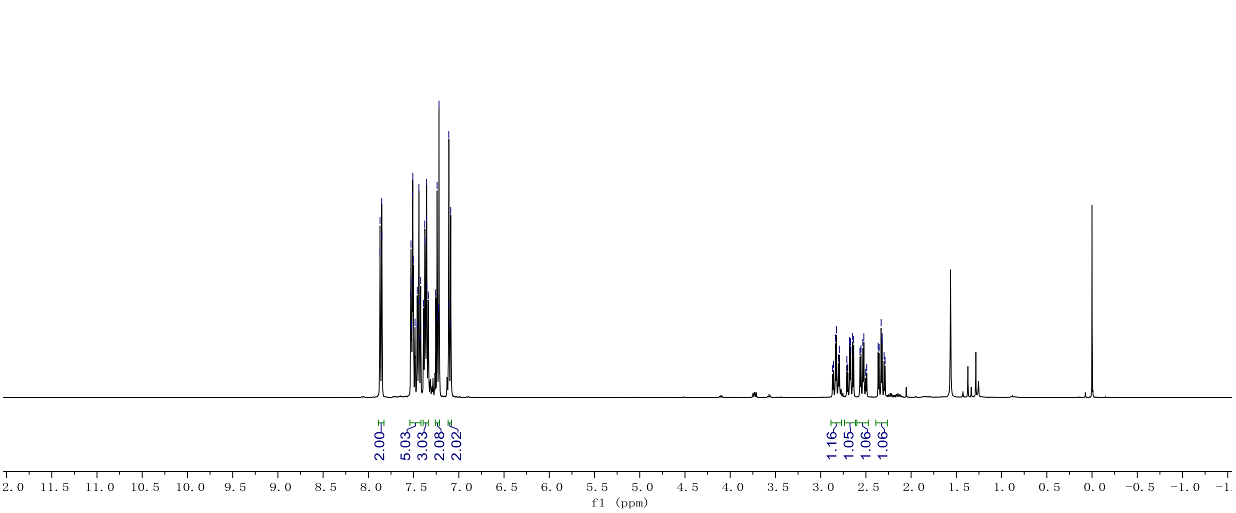

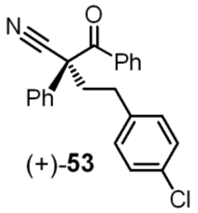

(+)-**53**

^{13}C NMR (101 MHz, $CDCl_3$)

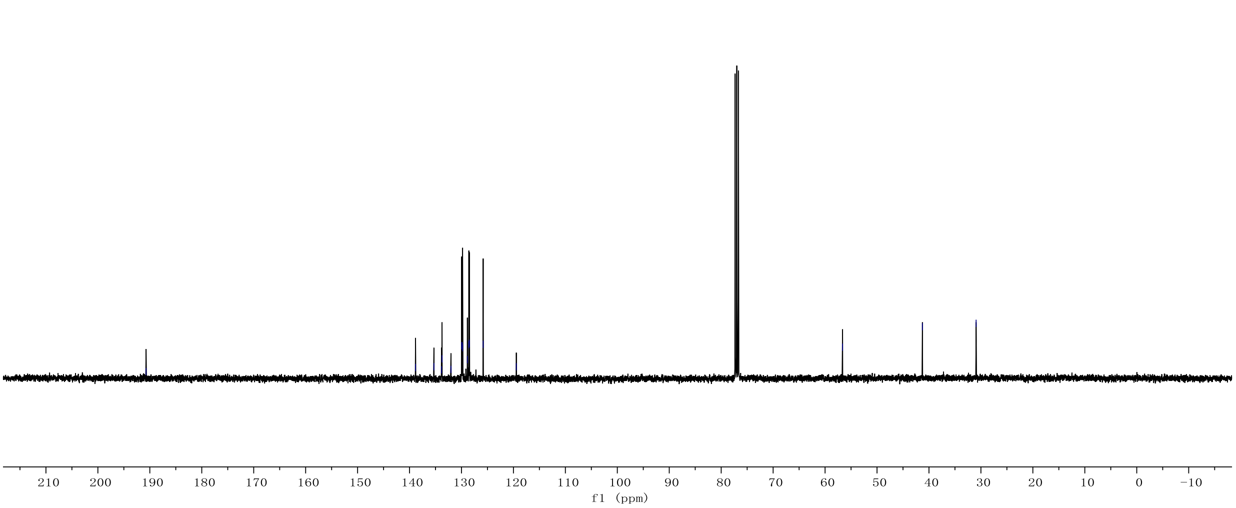

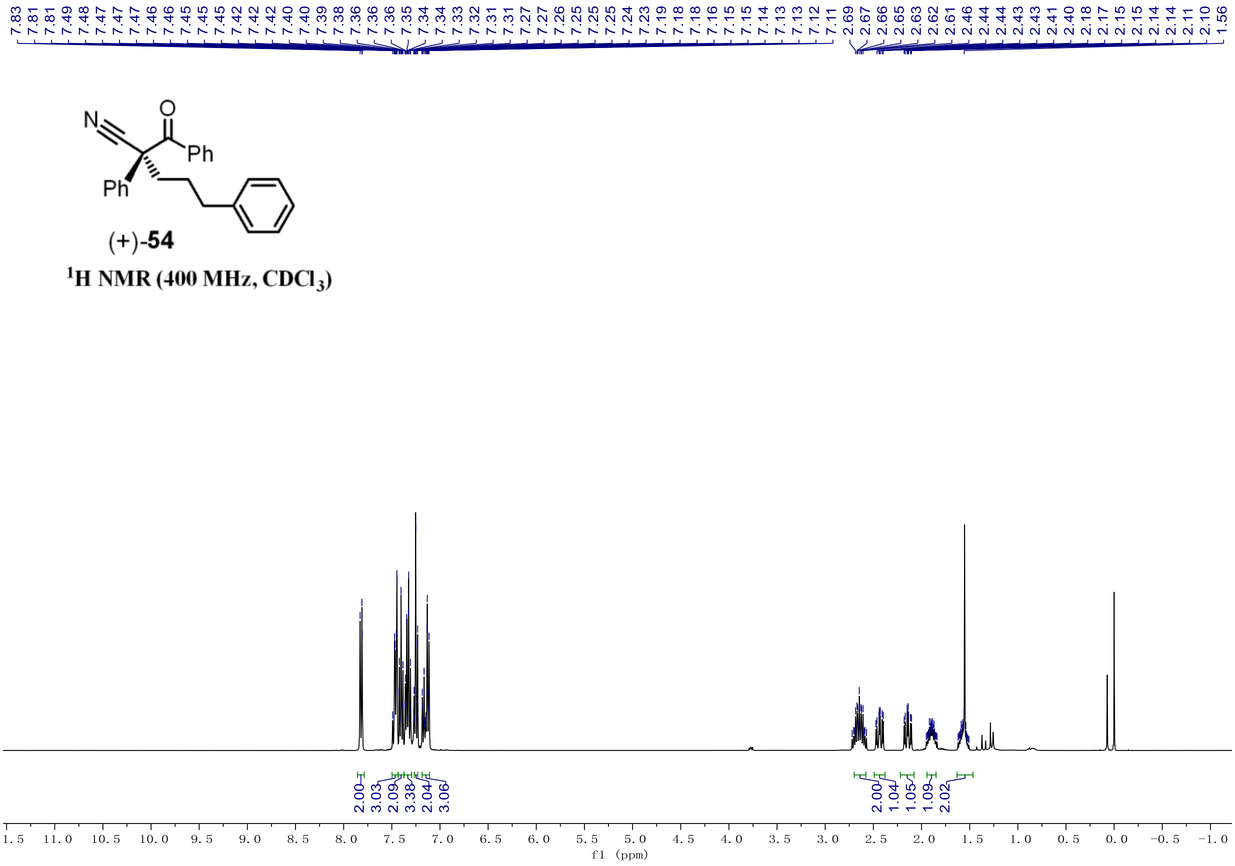
7.83 7.81 7.49 7.48 7.47 7.47 7.47 7.46 7.46 7.45 7.45 7.45 7.42 7.42 7.42 7.40 7.40 7.39 7.38 7.36 7.36 7.35 7.34 7.34 7.33 7.32 7.31 7.31 7.27 7.26 7.25 7.25 7.24 7.23 7.19 7.18 7.18 7.16 7.15 7.15 7.14 7.13 7.13 7.12 7.11 2.69 2.67 2.66 2.65 2.63 2.62 2.61 2.46 2.44 2.44 2.43 2.43 2.41 2.40 2.18 2.17 2.15 2.15 2.14 2.11 2.10 1.56
N
O
Ph
Ph
(+)-54
^{1}H NMR (400 MHz, CDCl$_3$)
2.00 3.03 2.09 3.38 2.04 3.06
2.00 1.04 1.05 1.09 2.02
1.5 11.0 10.5 10.0 9.5 9.0 8.5 8.0 7.5 7.0 6.5 6.0 5.5 5.0 4.5 4.0 3.5 3.0 2.5 2.0 1.5 1.0 0.5 0.0 −0.5 −1.0
f1 (ppm)

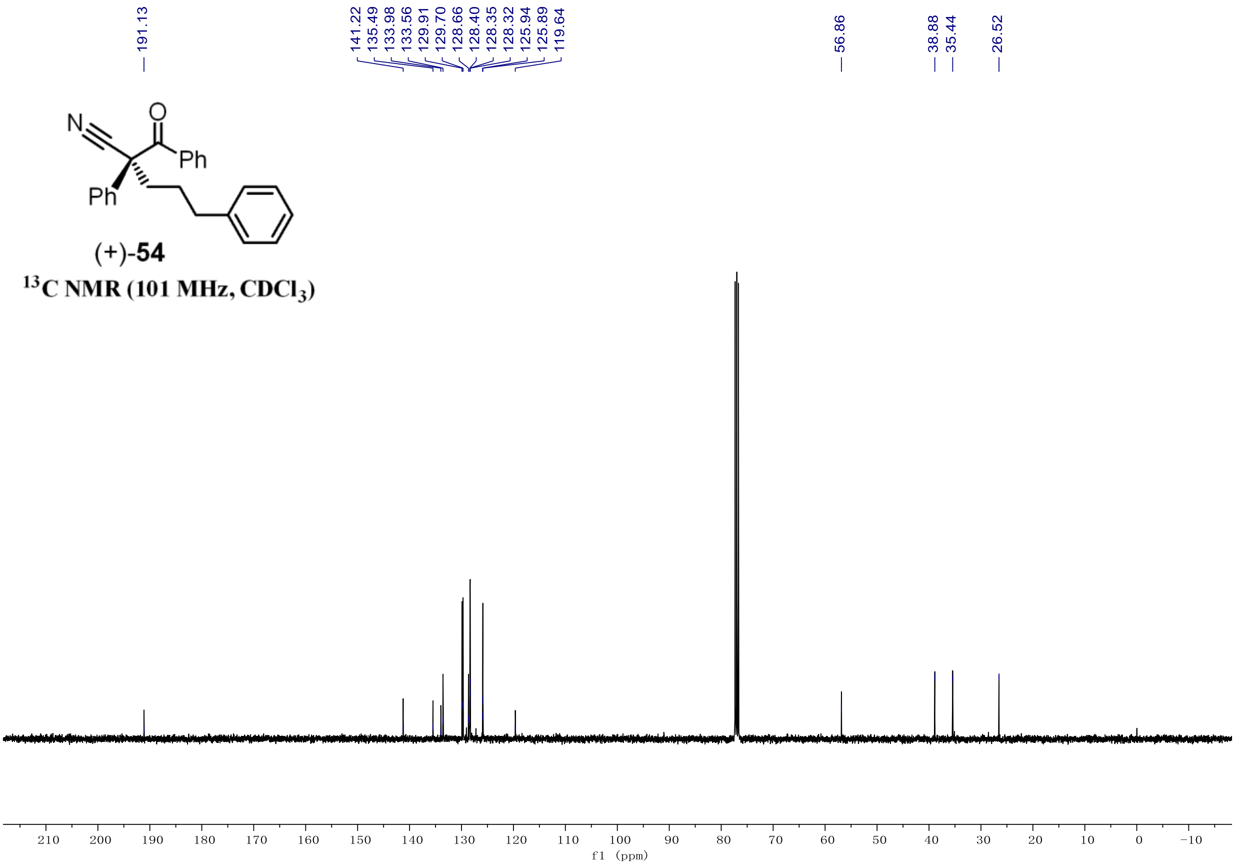
191.13
141.22 135.49 133.98 133.56 129.91 129.70 128.66 128.40 128.35 128.32 125.94 125.89 119.64
56.86
38.88 35.44
26.52
N
O
Ph
Ph
(+)-54
^{13}C NMR (101 MHz, CDCl$_3$)
210 200 190 180 170 160 150 140 130 120 110 100 90 80 70 60 50 40 30 20 10 0 −10
f1 (ppm)

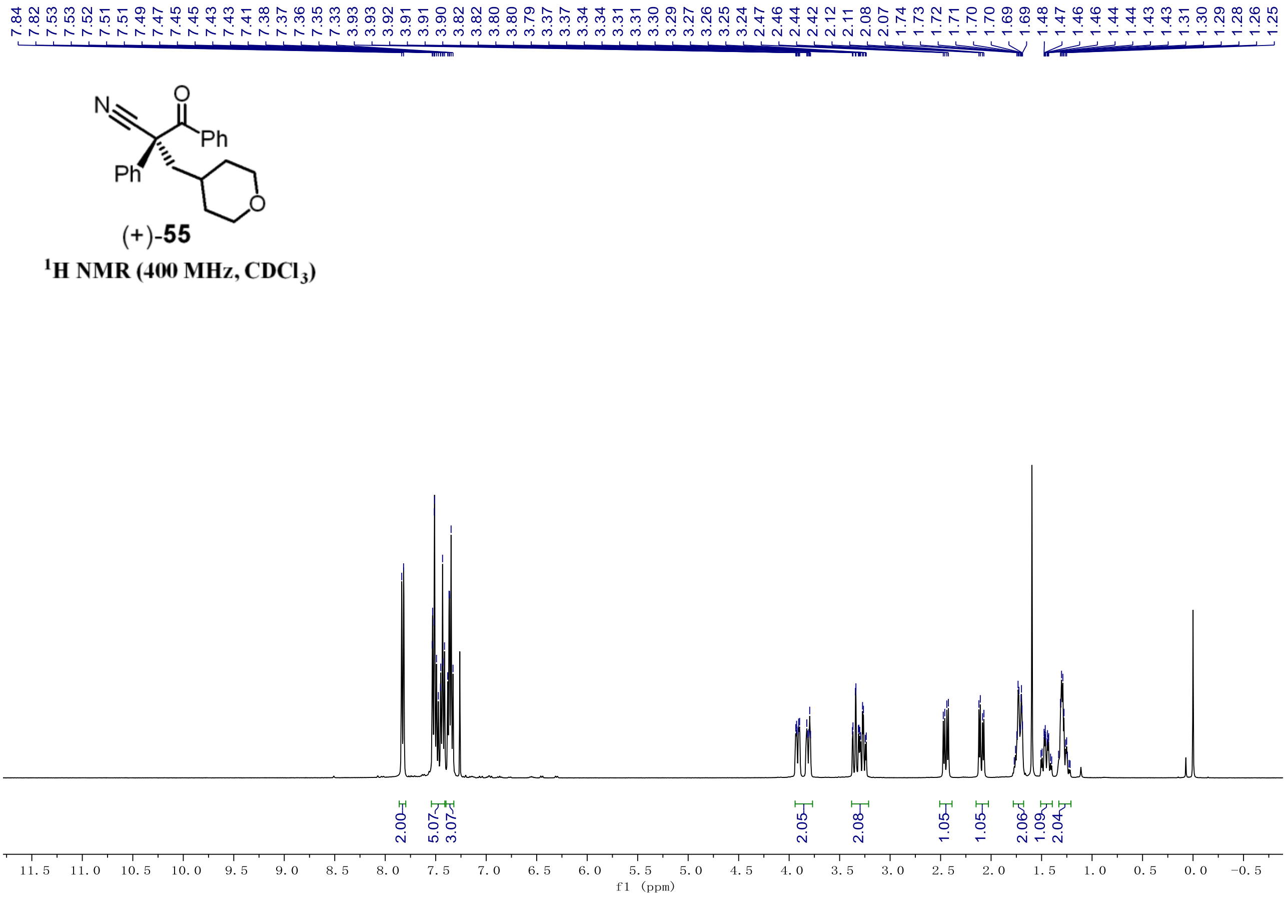

(+)-**55**

^{1}H NMR (400 MHz, CDCl$_3$)

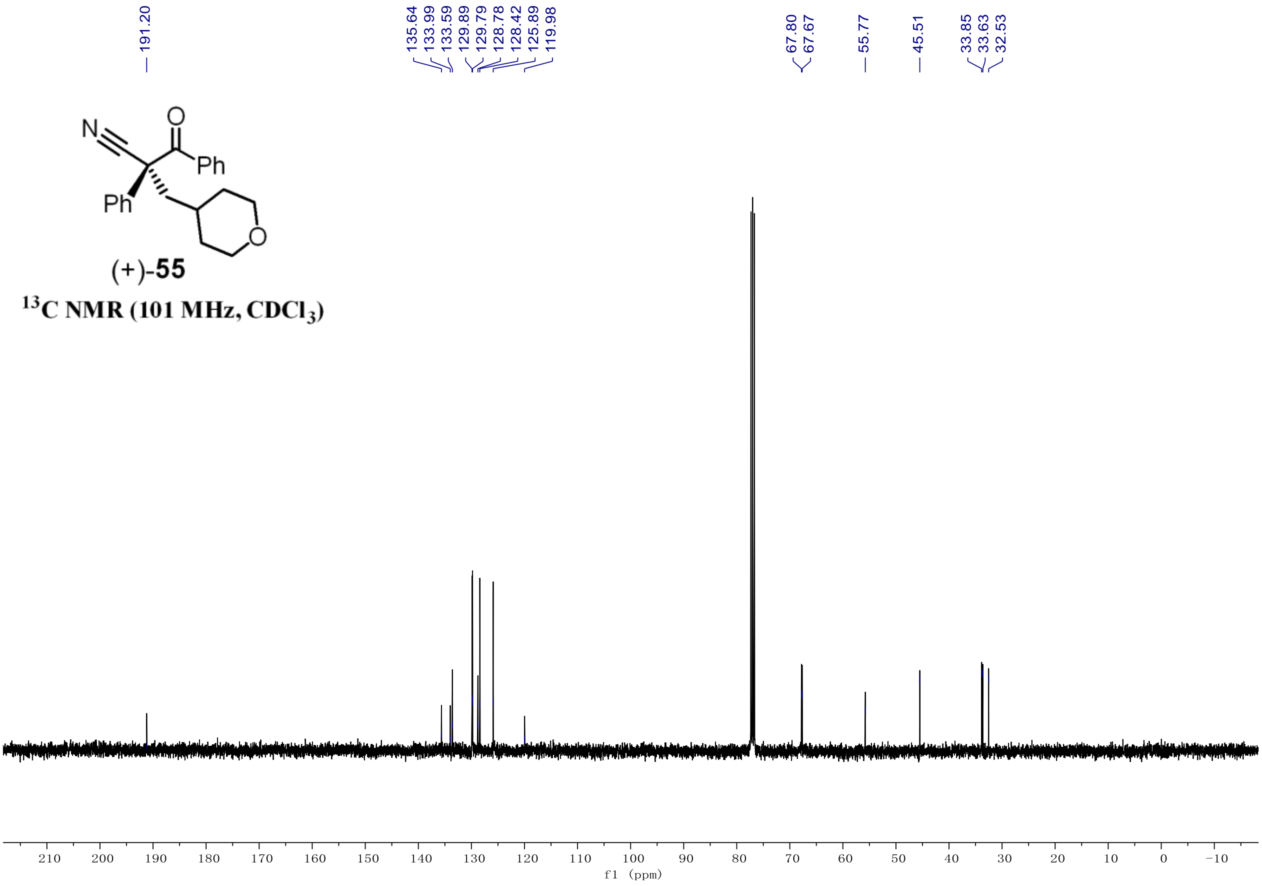

(+)-**55**

^{13}C NMR (101 MHz, CDCl$_3$)

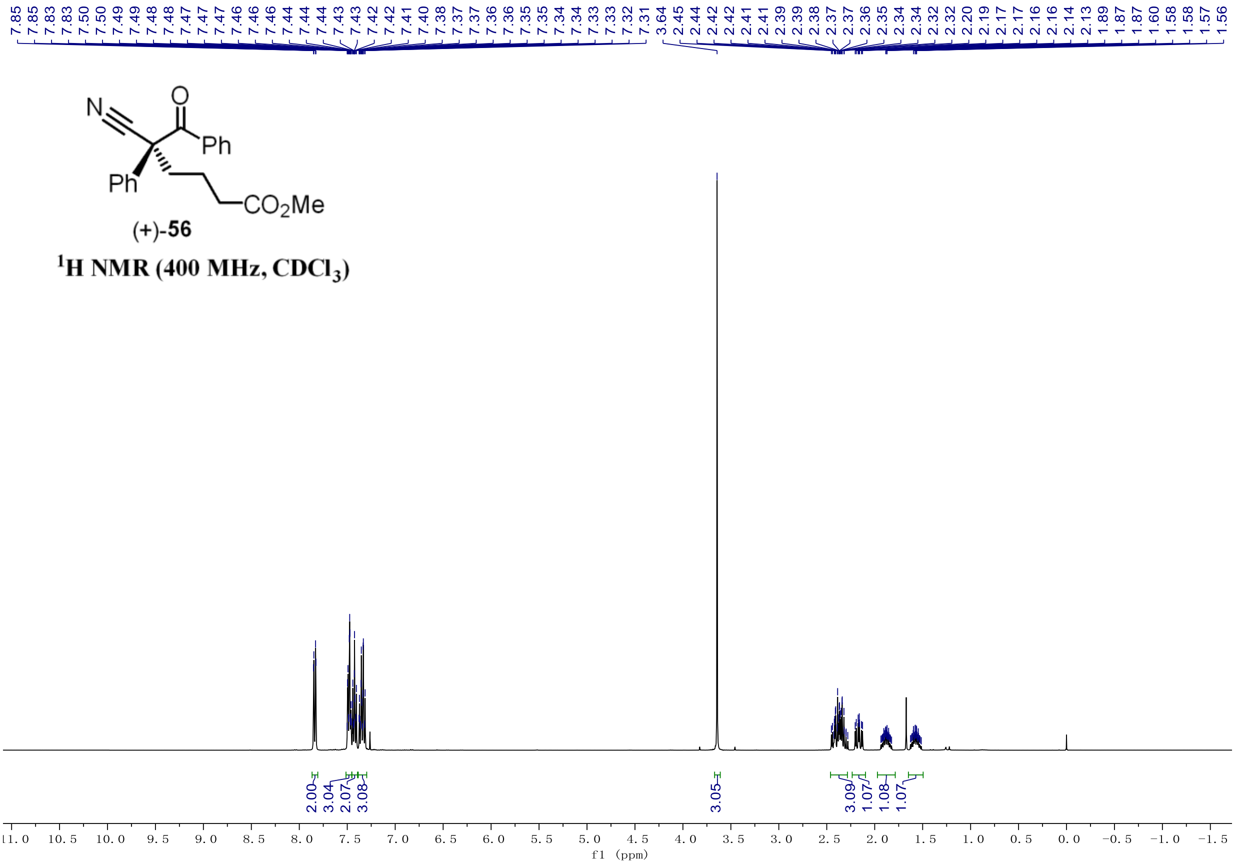

(+)-**56**

^{1}H NMR (400 MHz, $CDCl_3$)

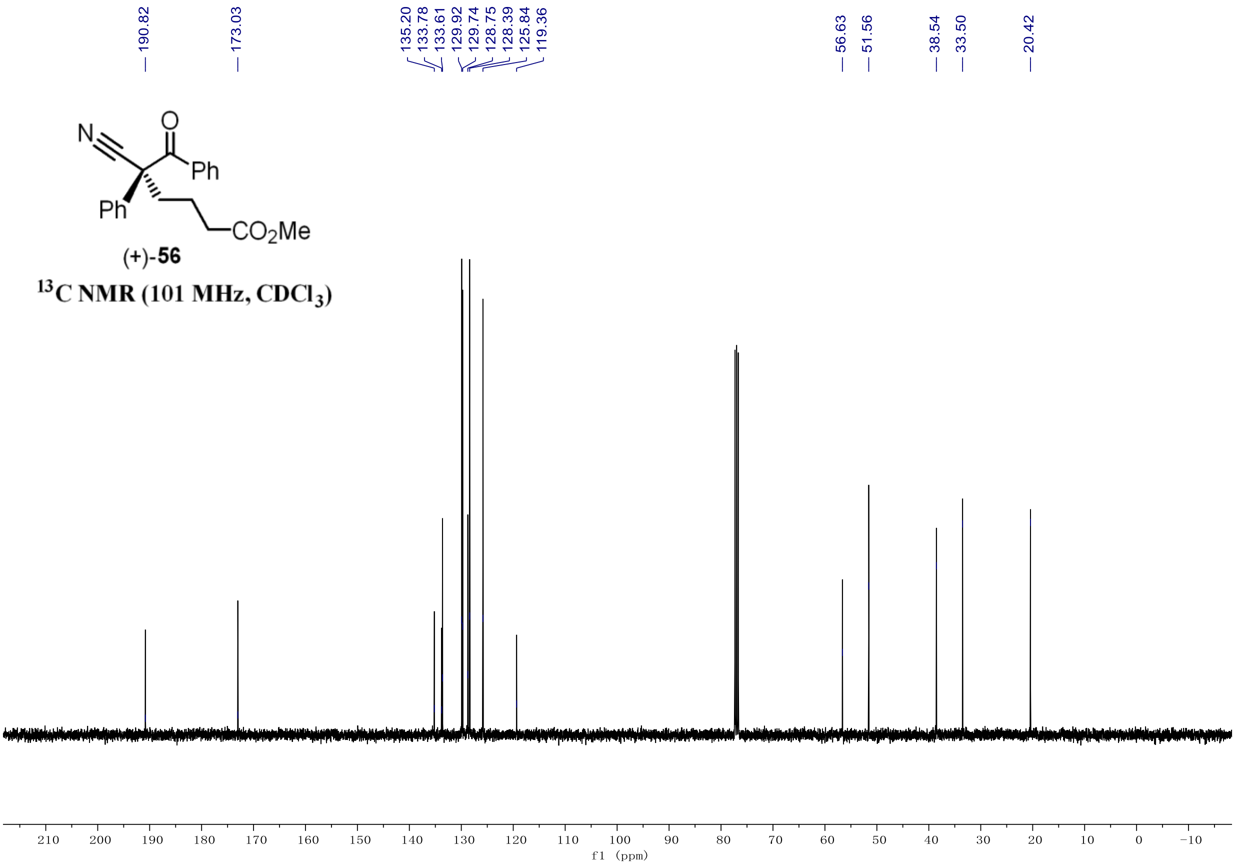

(+)-**56**

^{13}C NMR (101 MHz, $CDCl_3$)

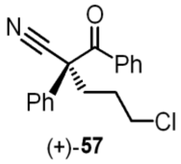

(+)-**57**

^{1}H NMR (400 MHz, CDCl$_3$)

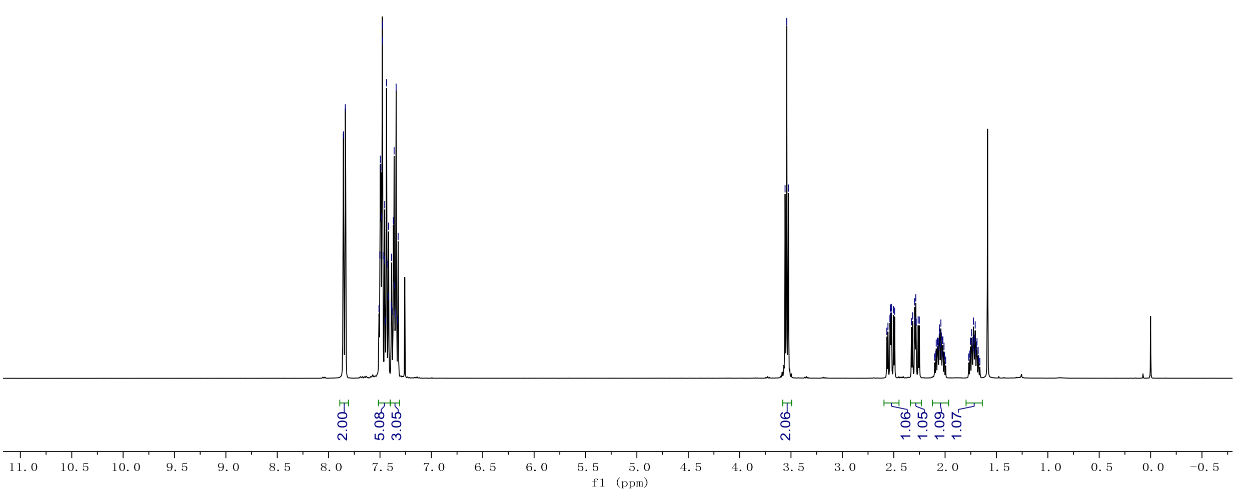

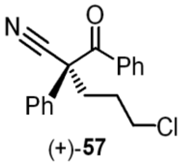

(+)-**57**

^{13}C NMR (101 MHz, CDCl$_3$)

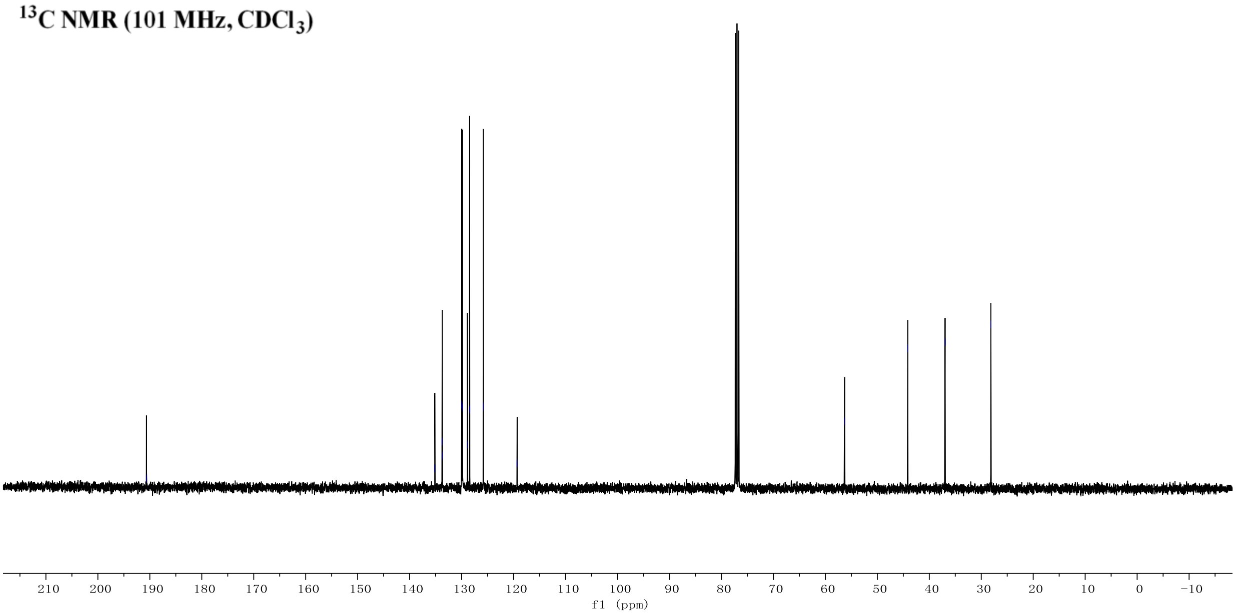

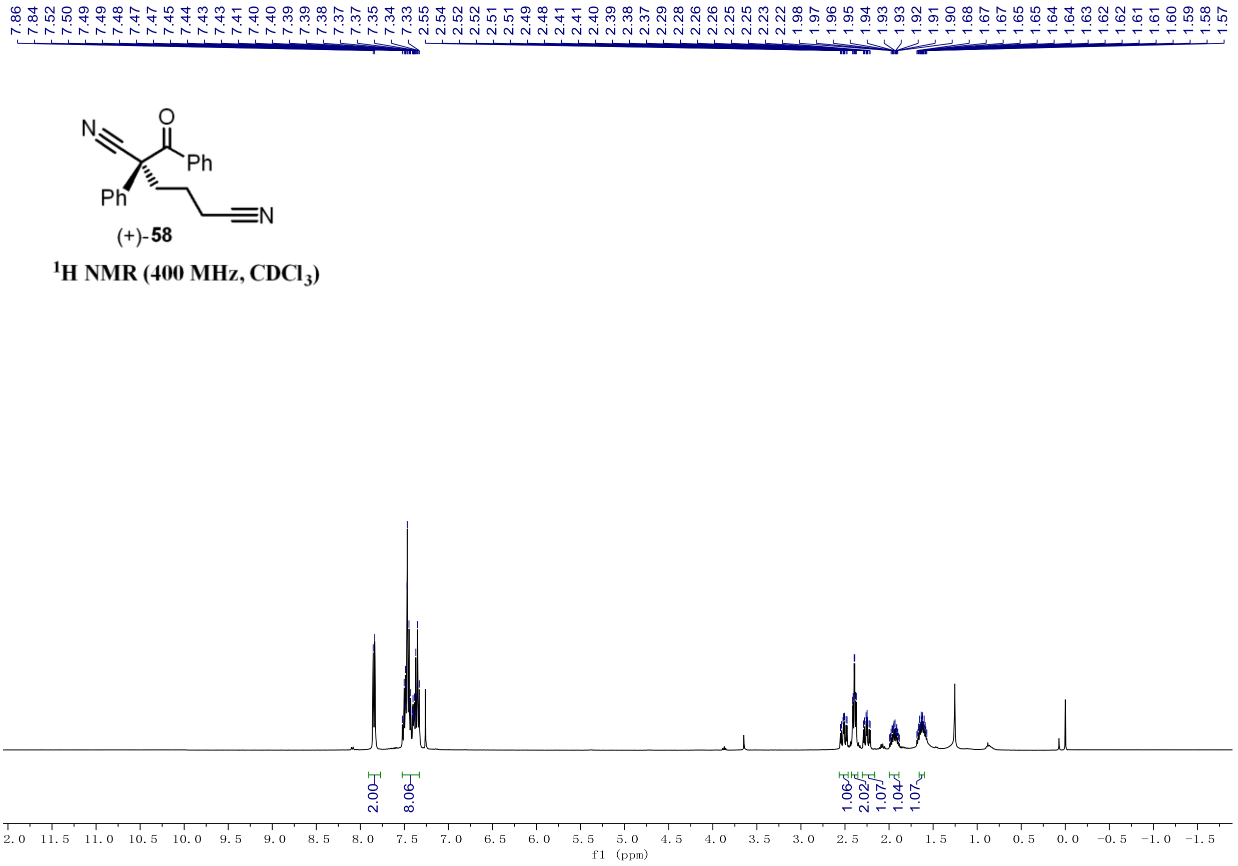
7.86 7.84 7.52 7.50 7.49 7.49 7.48 7.47 7.47 7.45 7.44 7.43 7.43 7.41 7.40 7.40 7.39 7.39 7.38 7.37 7.37 7.35 7.34 7.33 2.55 2.54 2.52 2.52 2.51 2.51 2.49 2.48 2.41 2.41 2.40 2.39 2.38 2.37 2.29 2.28 2.26 2.26 2.25 2.25 2.23 2.22 1.98 1.97 1.96 1.95 1.94 1.93 1.93 1.92 1.91 1.90 1.68 1.67 1.65 1.65 1.64 1.64 1.63 1.62 1.62 1.61 1.61 1.60 1.59 1.58 1.57
(+)-58
^{1}H NMR (400 MHz, CDCl$_3$)
2.00 8.06 1.06 2.02 1.07 1.04 1.07
f1 (ppm)

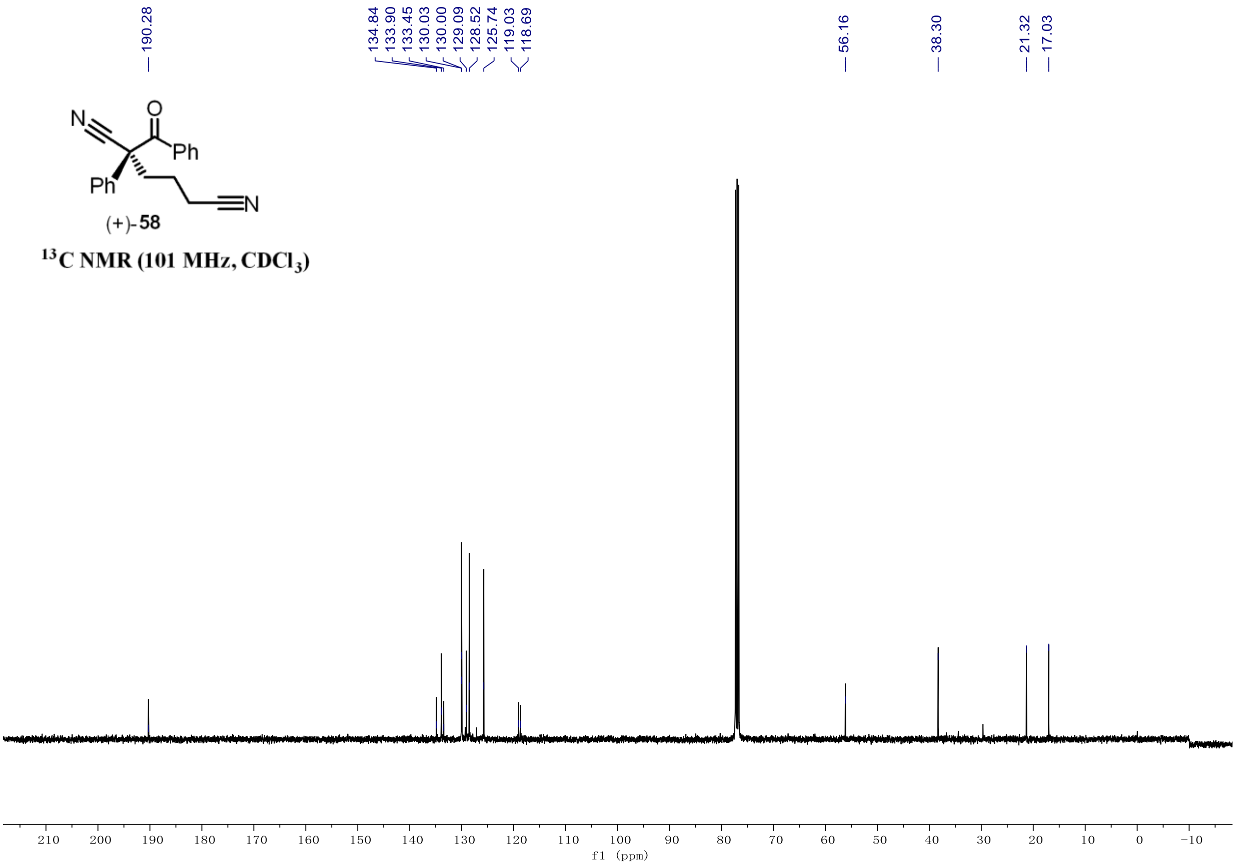
190.28
134.84 133.90 133.45 130.03 130.00 129.09 128.52 125.74 119.03 118.69
56.16
38.30
21.32 17.03
(+)-58
^{13}C NMR (101 MHz, CDCl$_3$)
f1 (ppm)

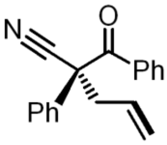

(+)-**59**

^{1}H NMR (400 MHz, $CDCl_3$)

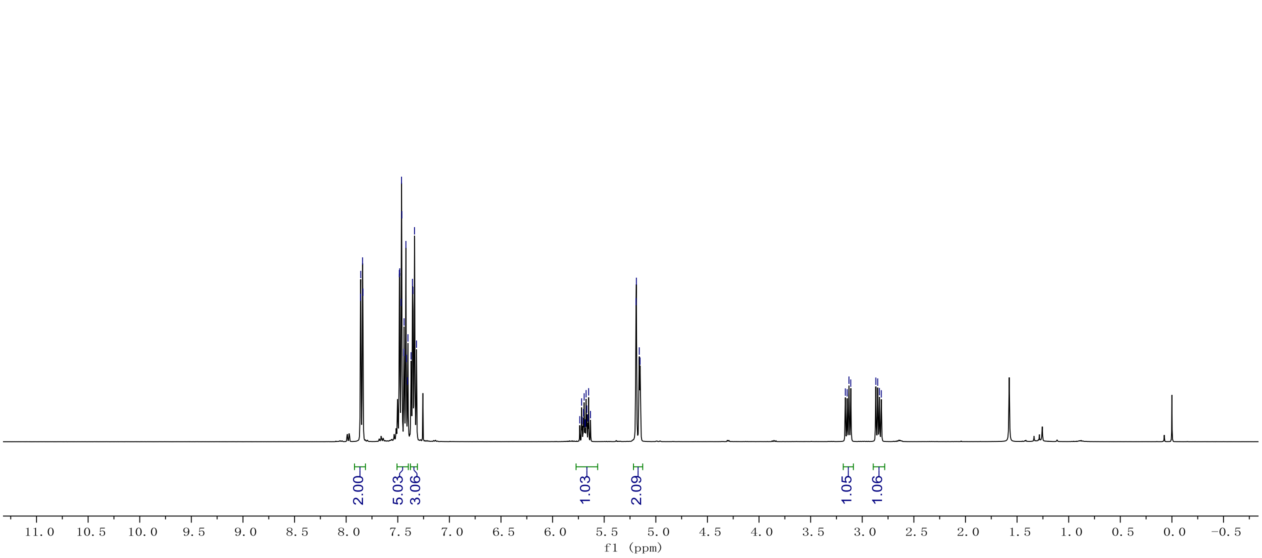

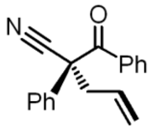

(+)-**59**

^{13}C NMR (101 MHz, $CDCl_3$)

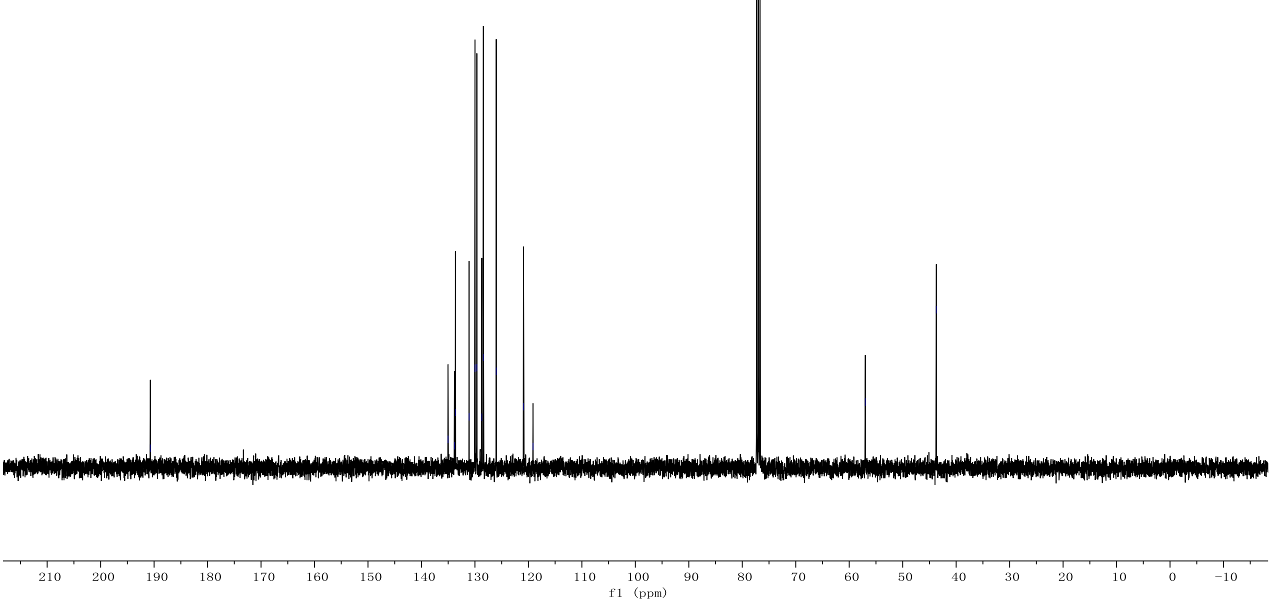

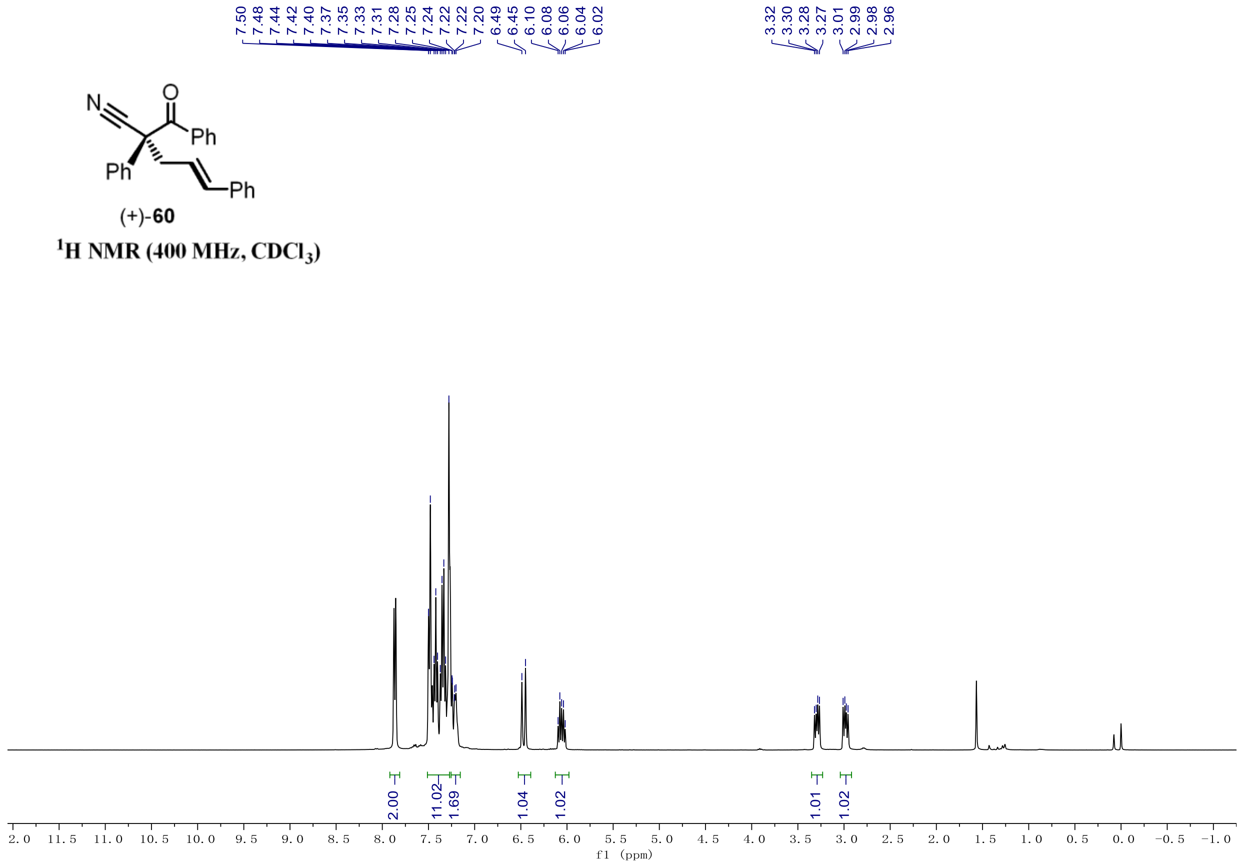

(+)-**60**

^{1}H NMR (400 MHz, CDCl$_3$)

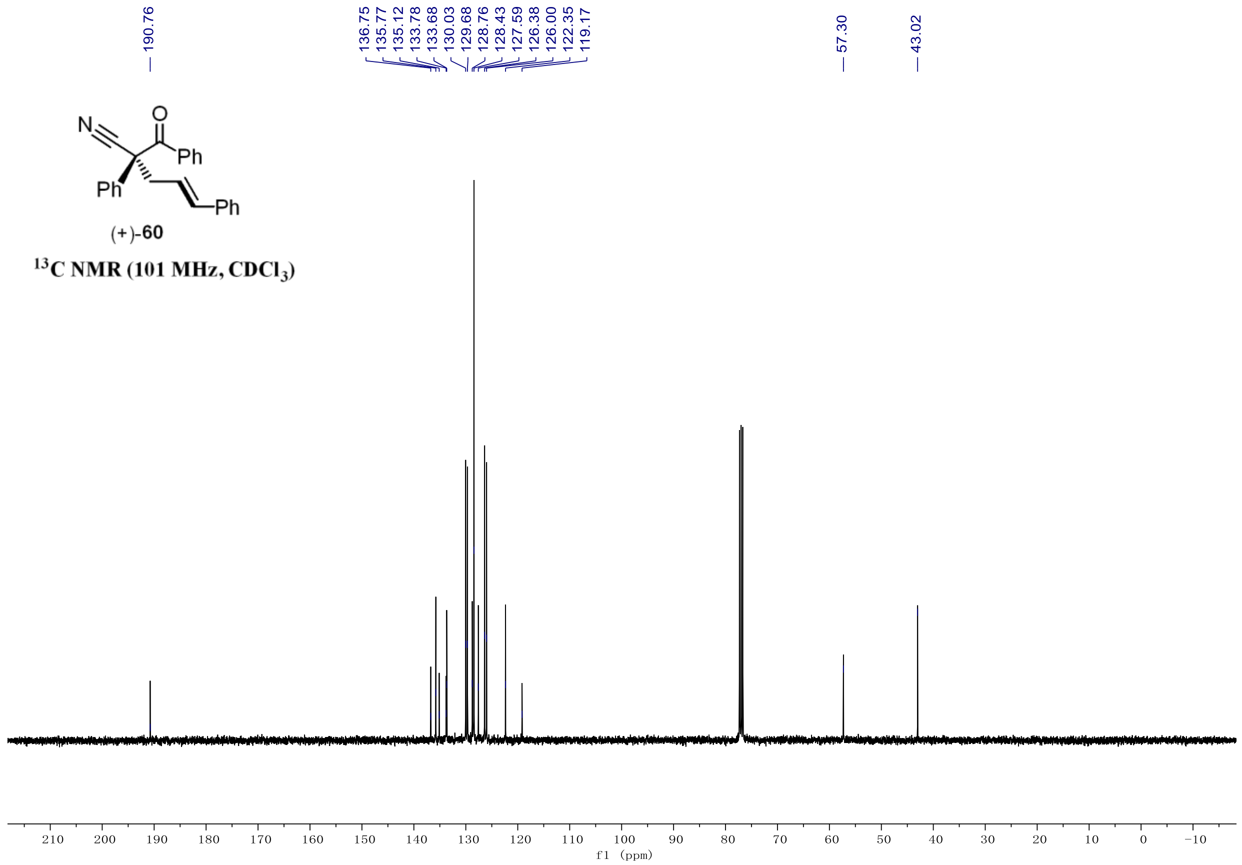

(+)-**60**

^{13}C NMR (101 MHz, CDCl$_3$)

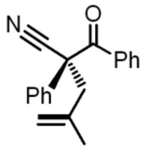

(+)-**61**

^{1}H NMR (400 MHz, $CDCl_3$)

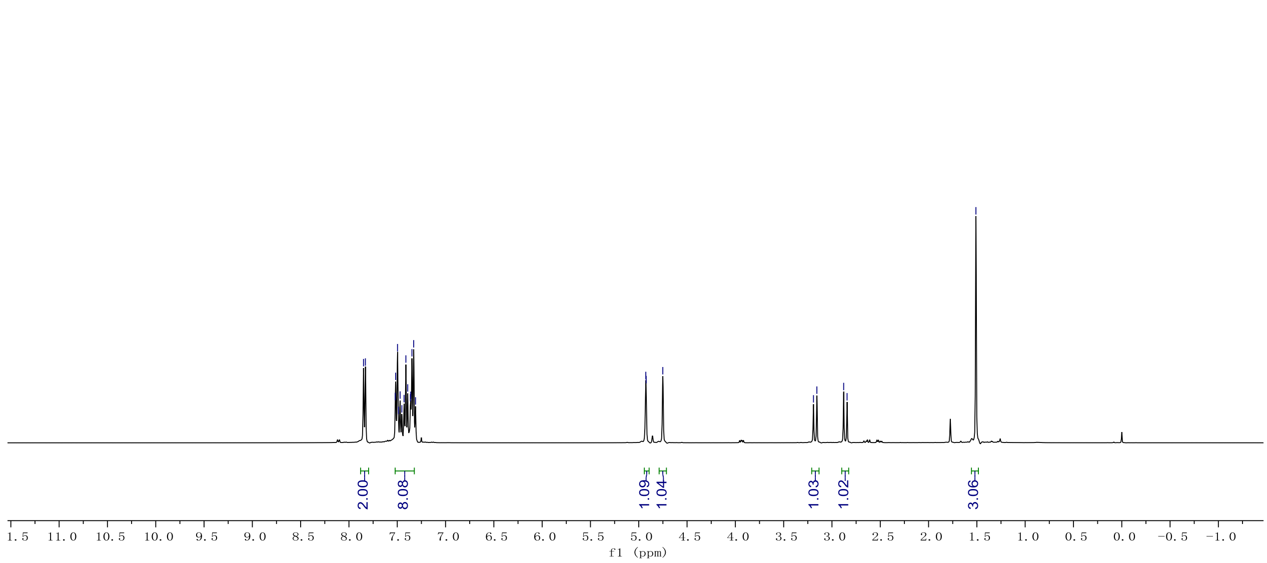

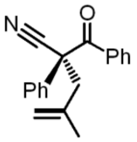

(+)-**61**

^{13}C NMR (101 MHz, $CDCl_3$)

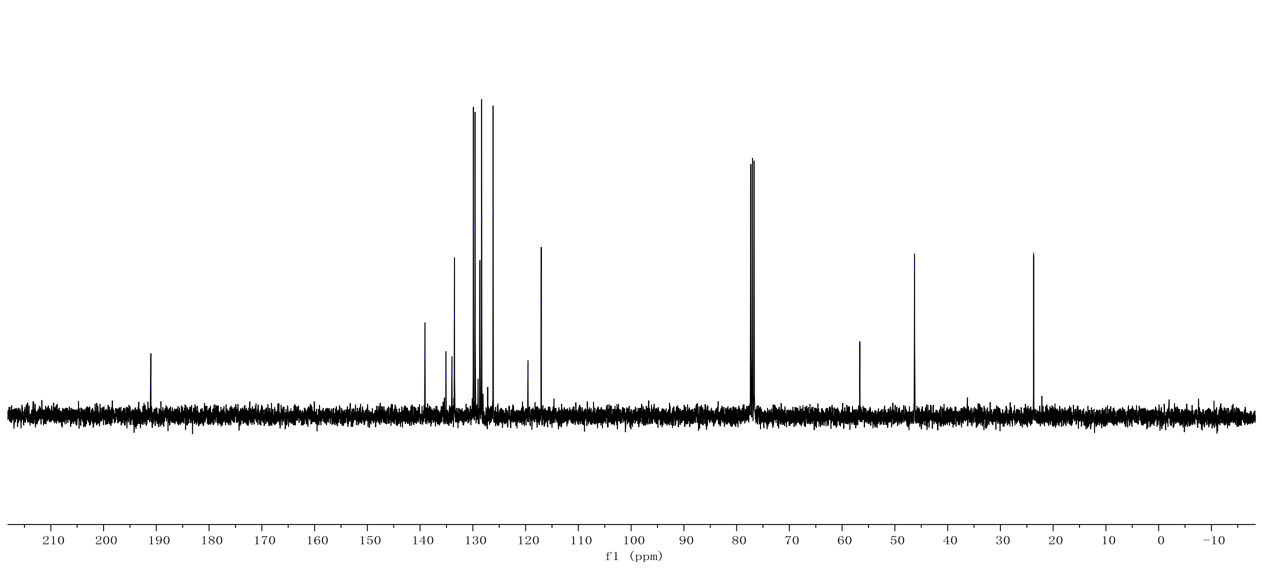

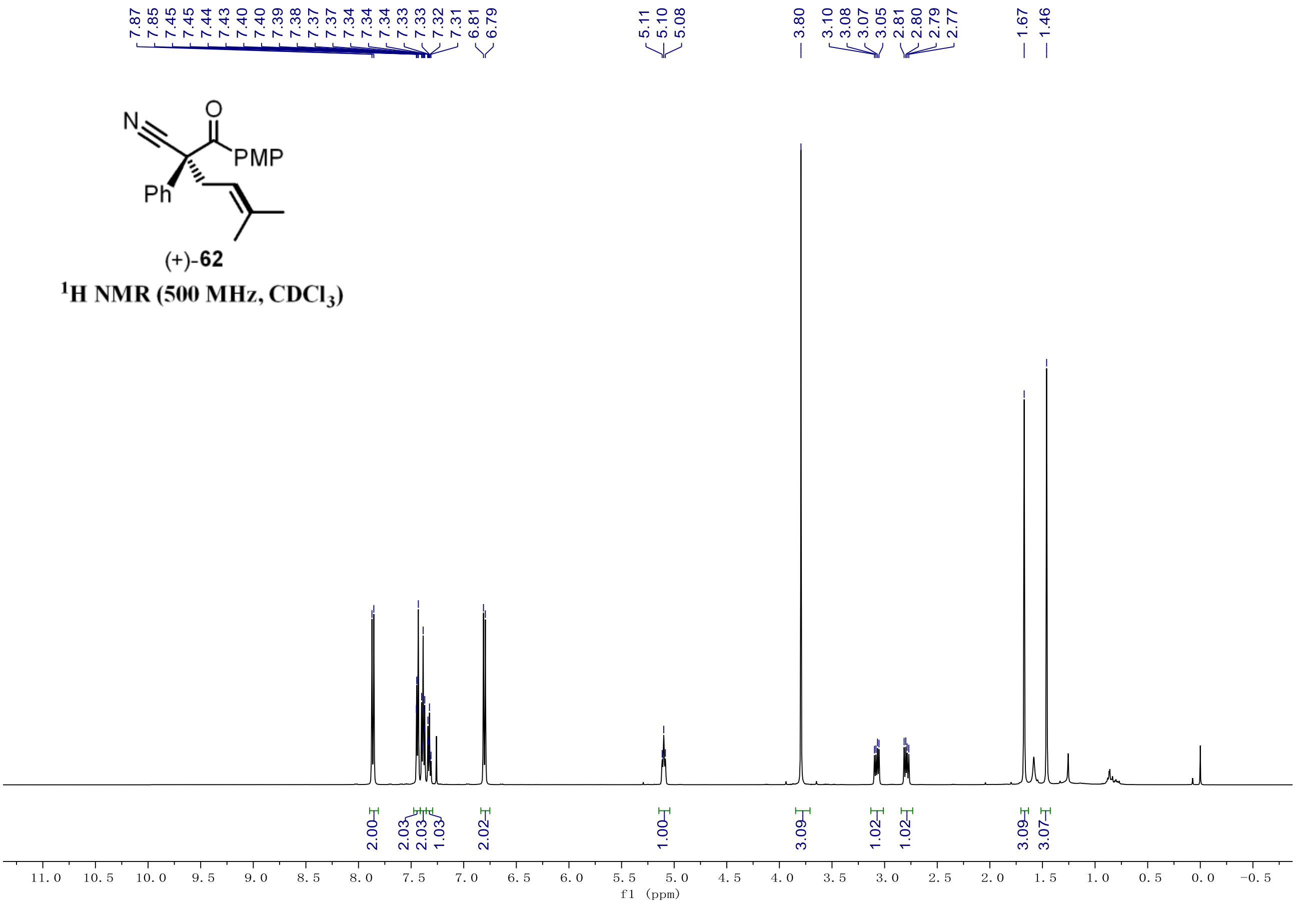
7.87 7.85 7.45 7.45 7.44 7.43 7.40 7.40 7.39 7.38 7.37 7.37 7.34 7.34 7.33 7.33 7.32 7.31 6.81 6.79
5.11 5.10 5.08
3.80
3.10 3.08 3.07 3.05 2.81 2.80 2.79 2.77
1.67
1.46
N
O
PMP
Ph
(+)-62
1H NMR (500 MHz, CDCl3)
2.00 2.03 2.03 1.03 2.02 1.00 3.09 1.02 1.02 3.09 3.07
11.0 10.5 10.0 9.5 9.0 8.5 8.0 7.5 7.0 6.5 6.0 5.5 5.0 4.5 4.0 3.5 3.0 2.5 2.0 1.5 1.0 0.5 0.0 -0.5
f1 (ppm)

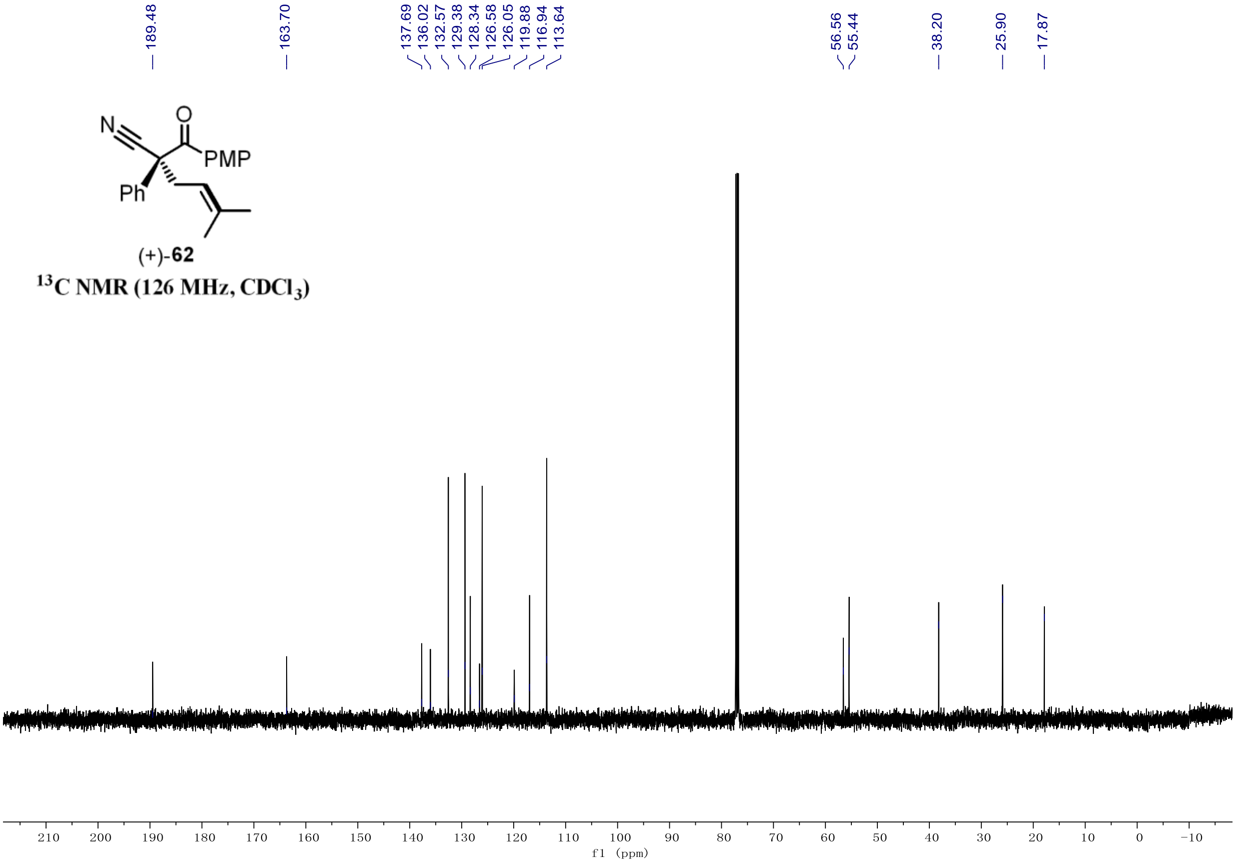
189.48
163.70
137.69 136.02 132.57 129.38 128.34 126.58 126.05 119.88 116.94 113.64
56.56 55.44
38.20
25.90
17.87
N
O
PMP
Ph
(+)-62
13C NMR (126 MHz, CDCl3)
210 200 190 180 170 160 150 140 130 120 110 100 90 80 70 60 50 40 30 20 10 0 -10
f1 (ppm)

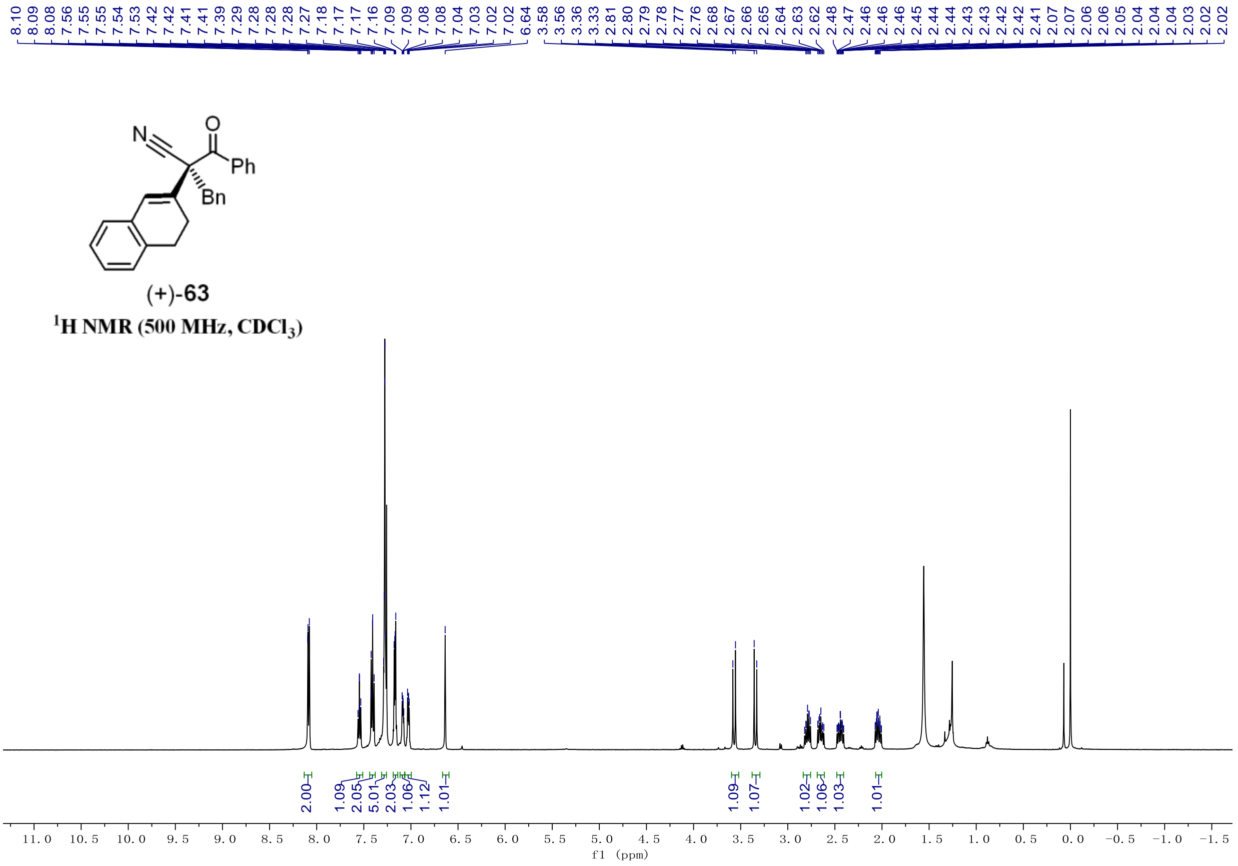

(+)-**63**

^{1}H NMR (500 MHz, $CDCl_3$)

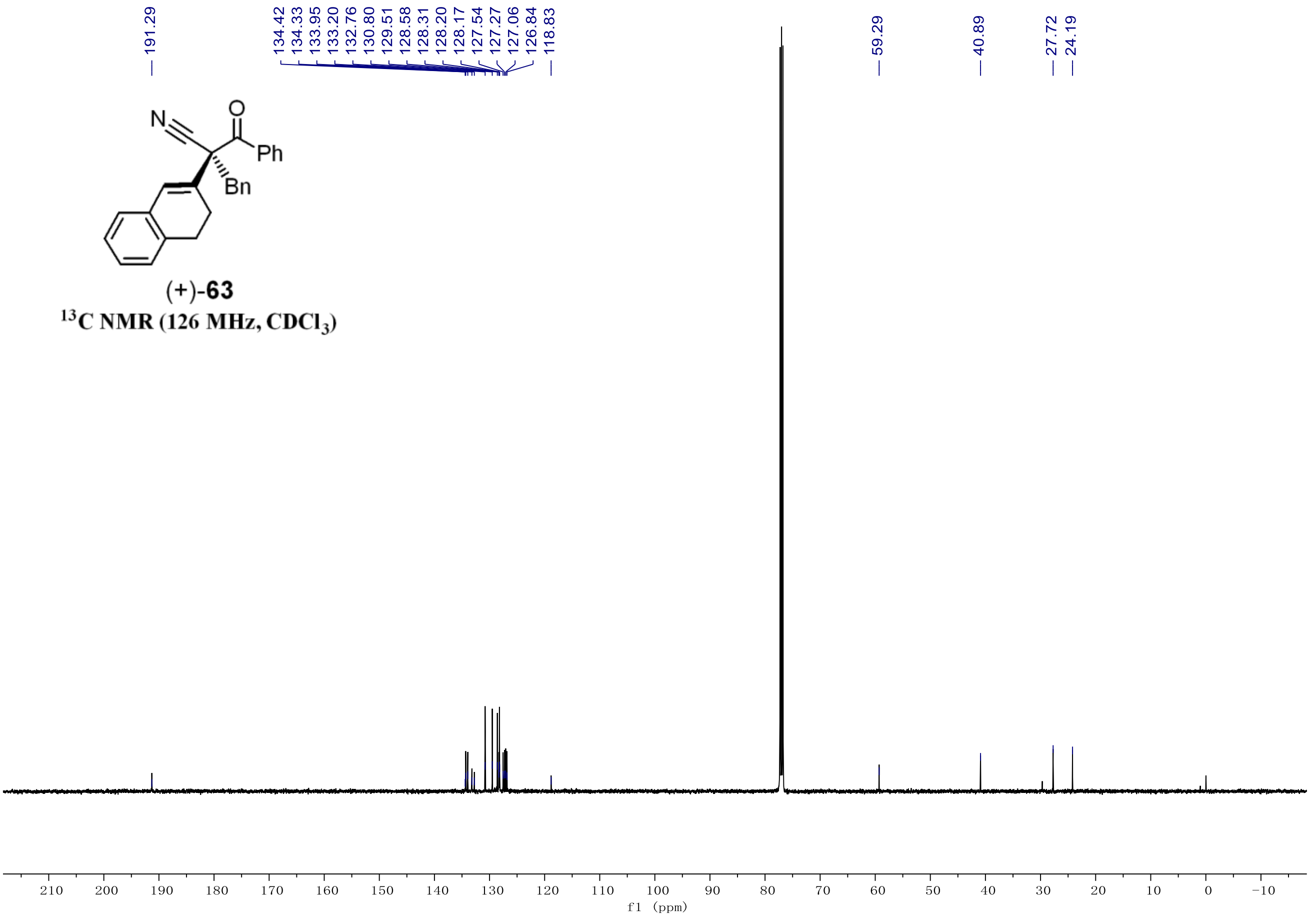

(+)-**63**

^{13}C NMR (126 MHz, $CDCl_3$)

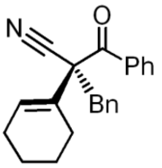

(+)-**64**

^{1}H NMR (400 MHz, CDCl$_3$)

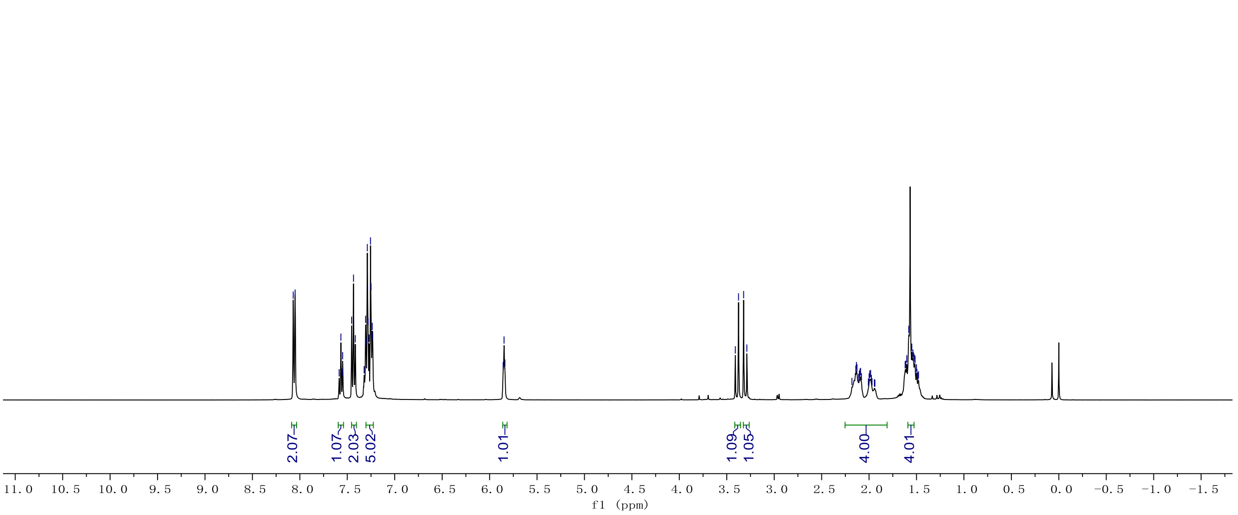

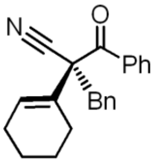

(+)-**64**

^{13}C NMR (101 MHz, CDCl$_3$)

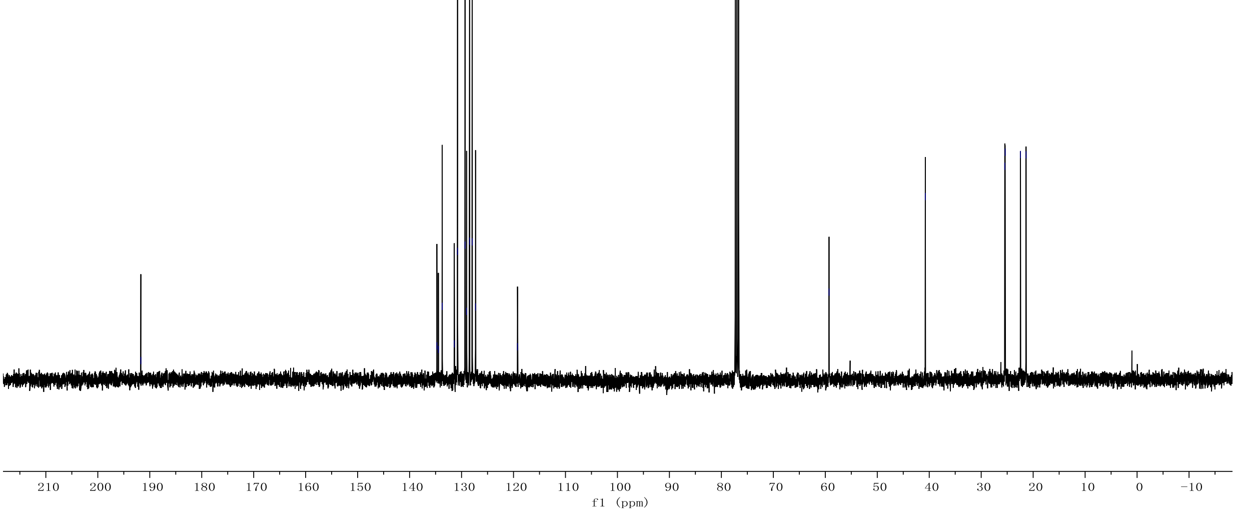

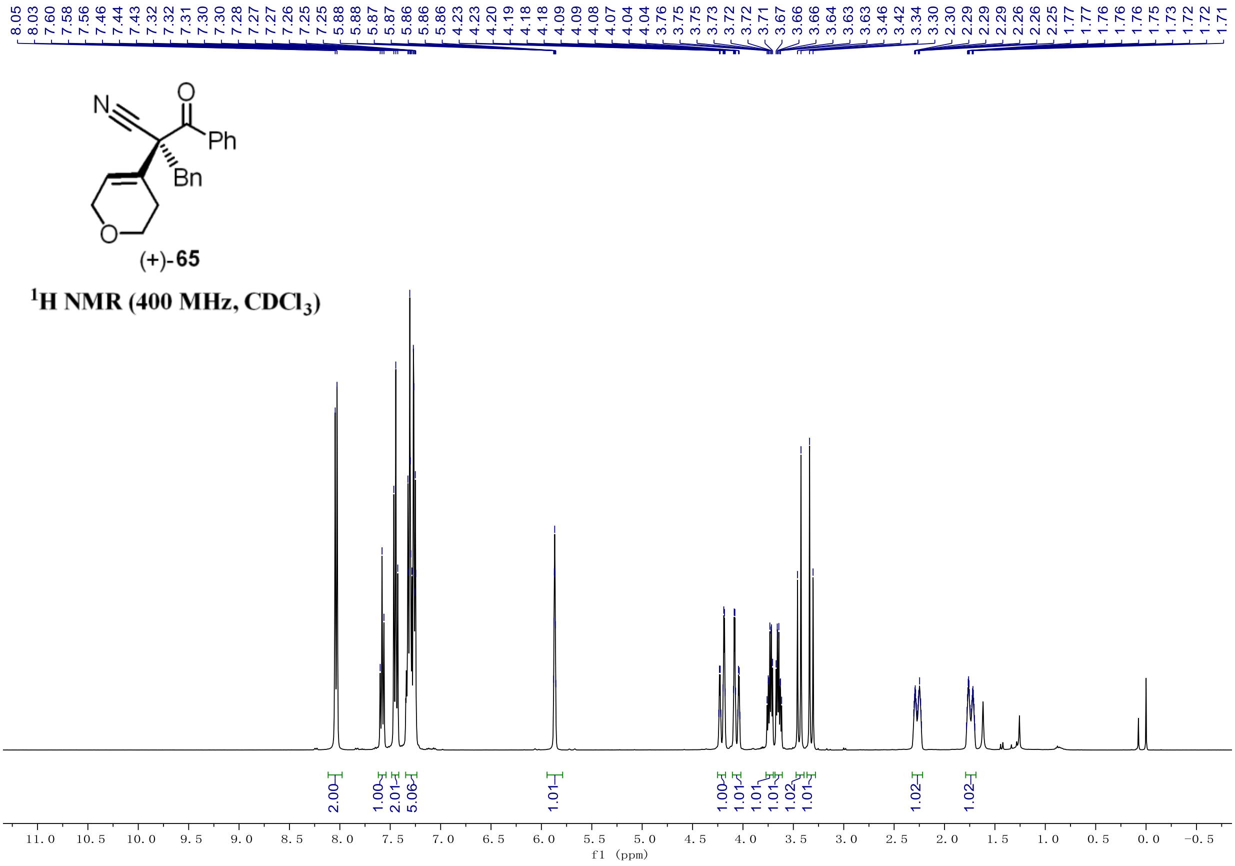
8.05 8.03 7.60 7.58 7.56 7.46 7.44 7.43 7.32 7.32 7.31 7.30 7.30 7.28 7.27 7.27 7.26 7.25 7.25 5.88 5.88 5.87 5.87 5.86 5.86 5.86 4.23 4.23 4.20 4.19 4.19 4.18 4.09 4.09 4.08 4.07 4.04 4.04 3.76 3.75 3.75 3.73 3.72 3.72 3.71 3.67 3.66 3.66 3.64 3.63 3.63 3.46 3.42 3.34 3.30 2.30 2.29 2.29 2.29 2.26 2.26 2.25 1.77 1.77 1.76 1.76 1.75 1.73 1.72 1.72 1.71
N
O
Ph
Bn
O
(+)-65
1H NMR (400 MHz, CDCl3)
2.00 1.00 2.01 5.06 1.01 1.00 1.01 1.01 1.01 1.02 1.01 1.02 1.02
f1 (ppm)

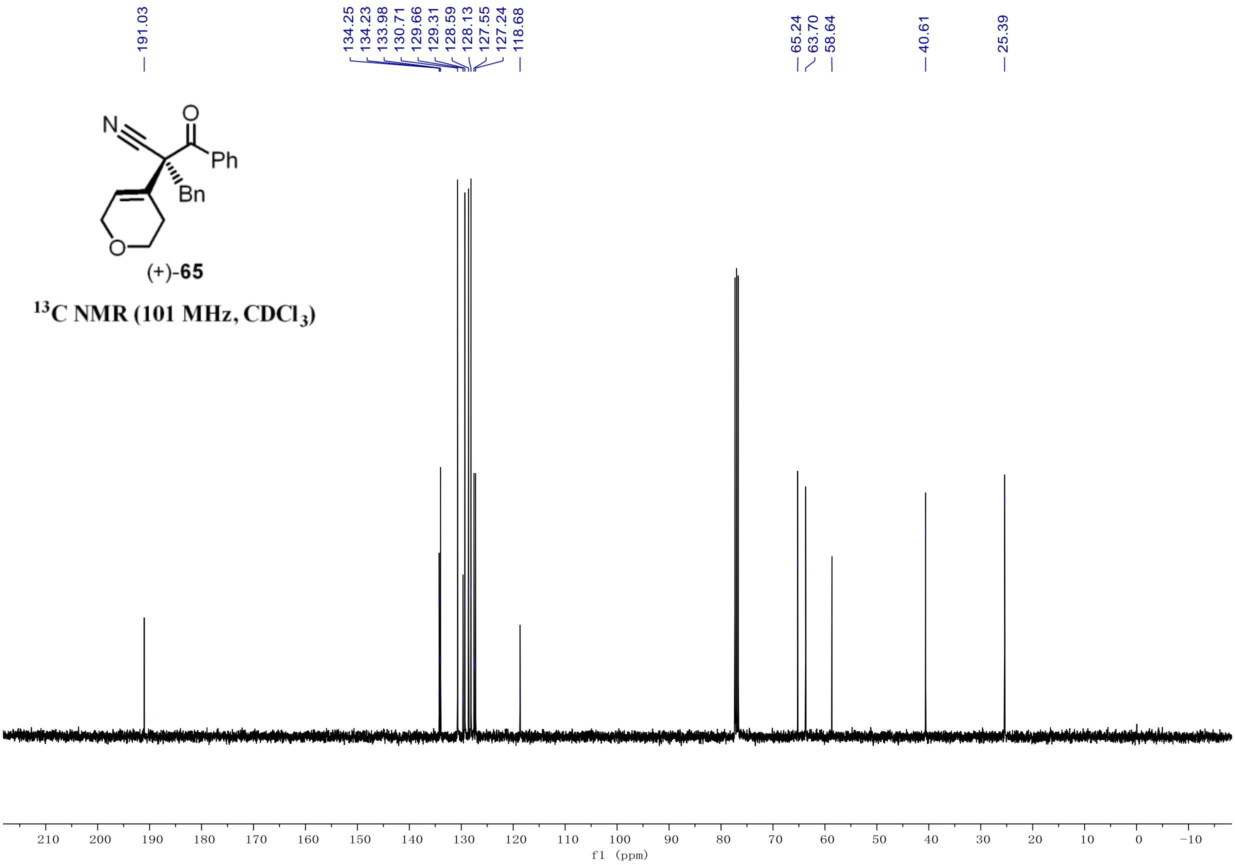
191.03
134.25 134.23 133.98 130.71 129.66 129.31 128.59 128.13 127.55 127.24 118.68
65.24 63.70 58.64
40.61
25.39
N
O
Ph
Bn
O
(+)-65
13C NMR (101 MHz, CDCl3)
f1 (ppm)

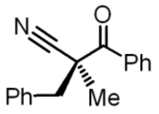

(+)-**66**

^{1}H NMR (400 MHz, $CDCl_3$)

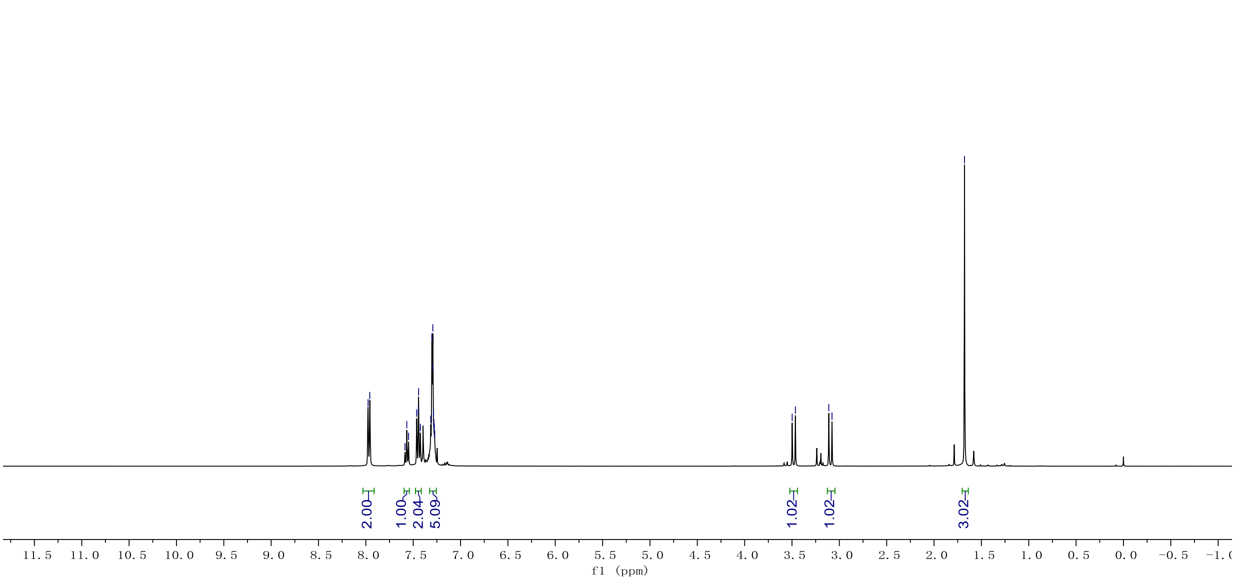

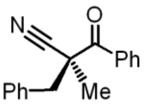

(+)-**66**

^{13}C NMR (101 MHz, $CDCl_3$)

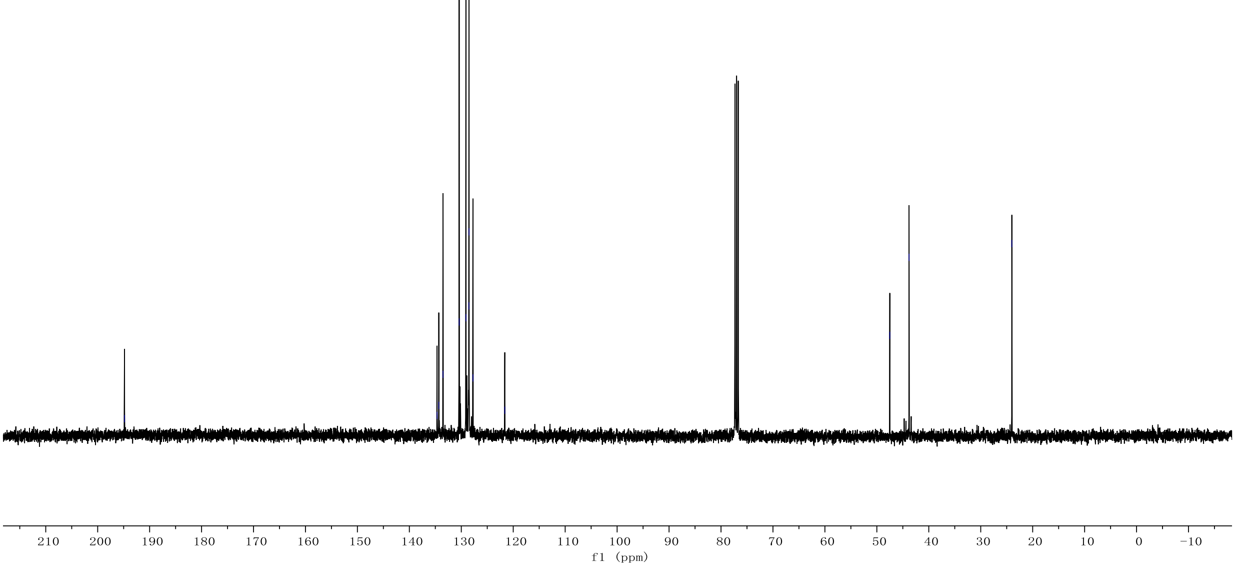

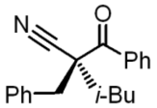

(+)-**67**

^{1}H NMR (400 MHz, $CDCl_3$)

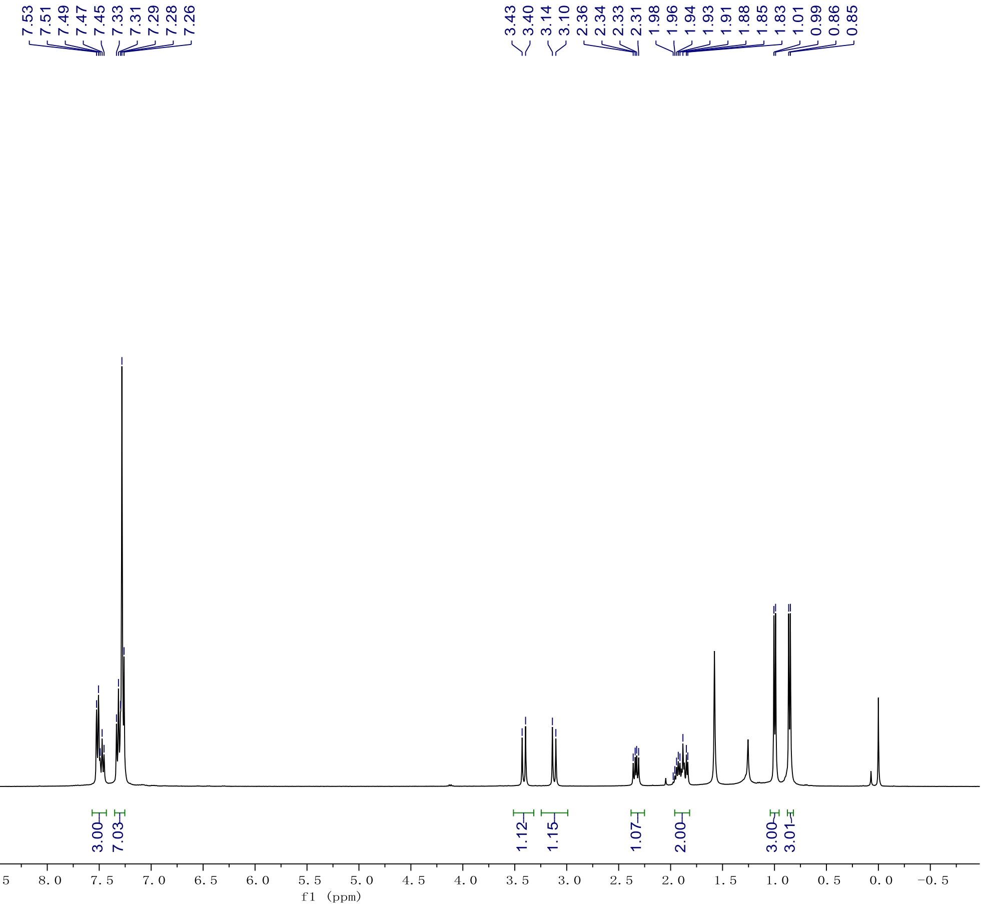

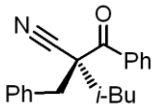

(+)-**67**

^{13}C NMR (101 MHz, $CDCl_3$)

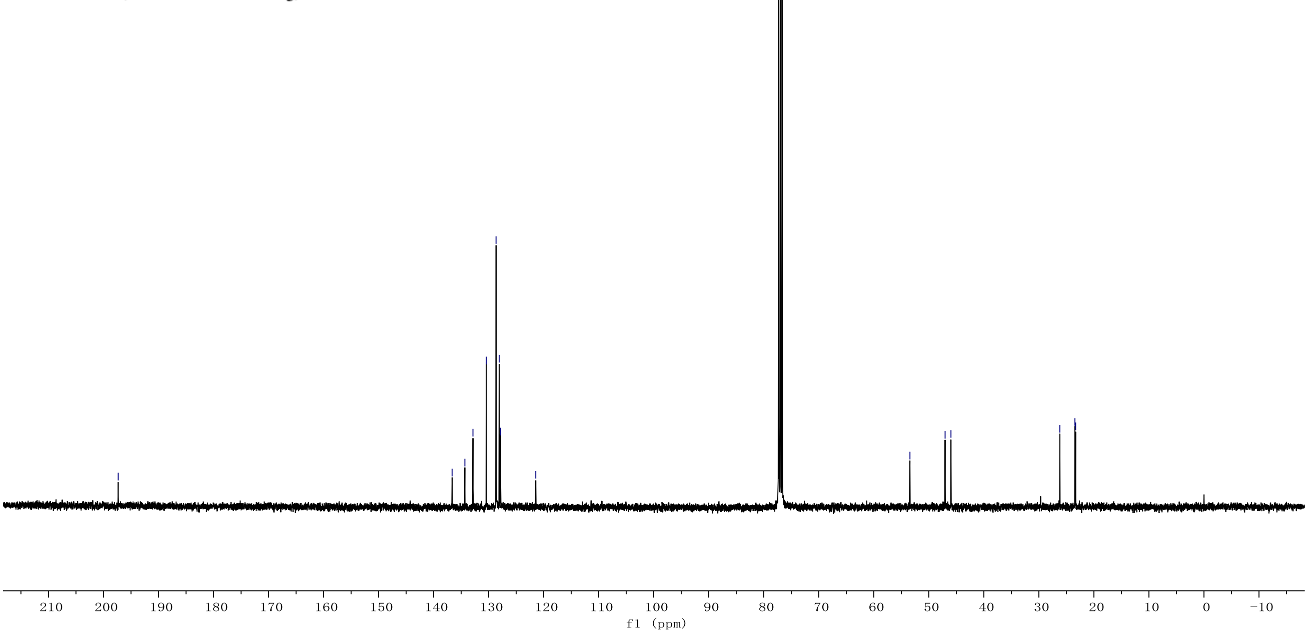

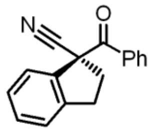

(+)-**68**

^{1}H NMR (400 MHz, $CDCl_3$)

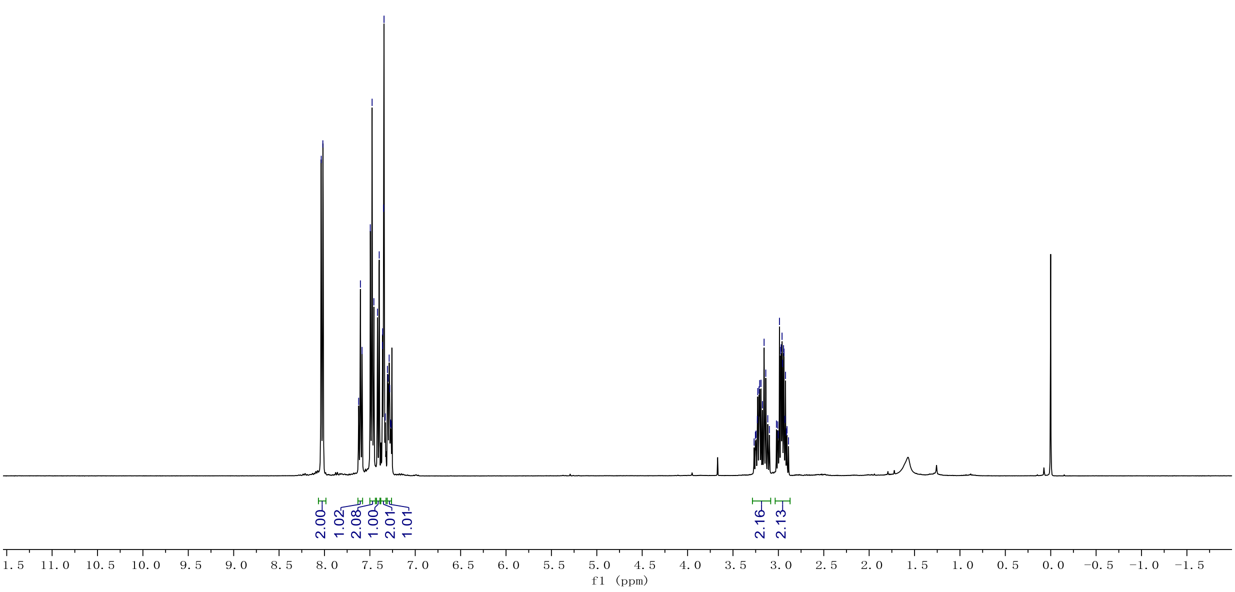

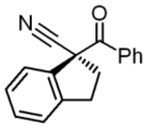

(+)-**68**

^{13}C NMR (101 MHz, $CDCl_3$)

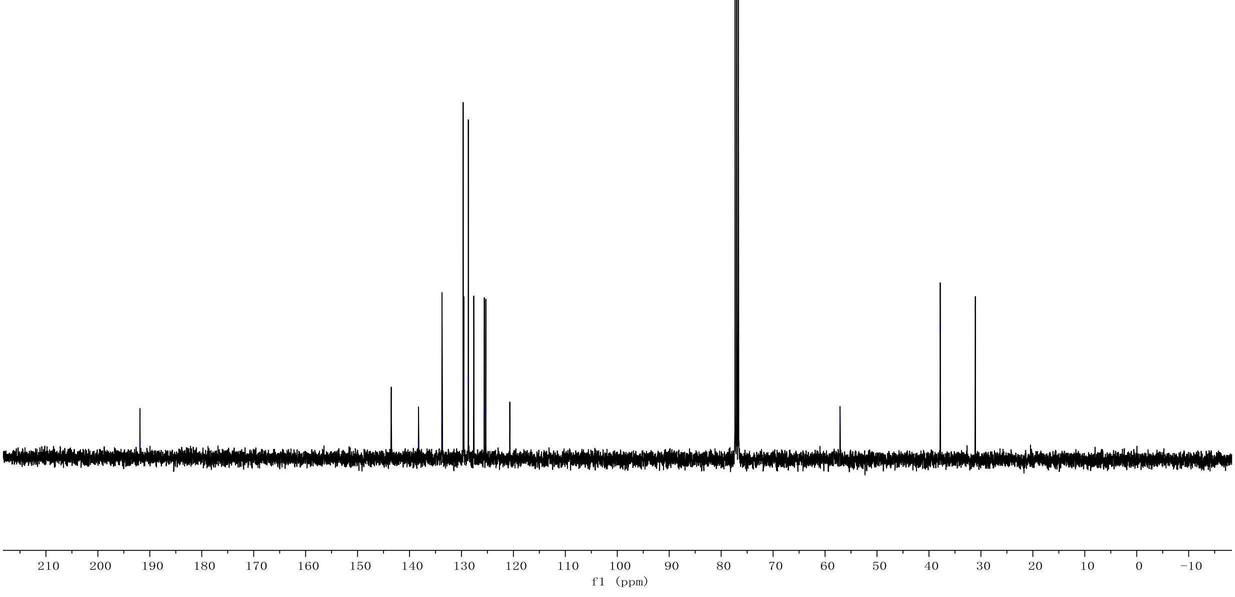

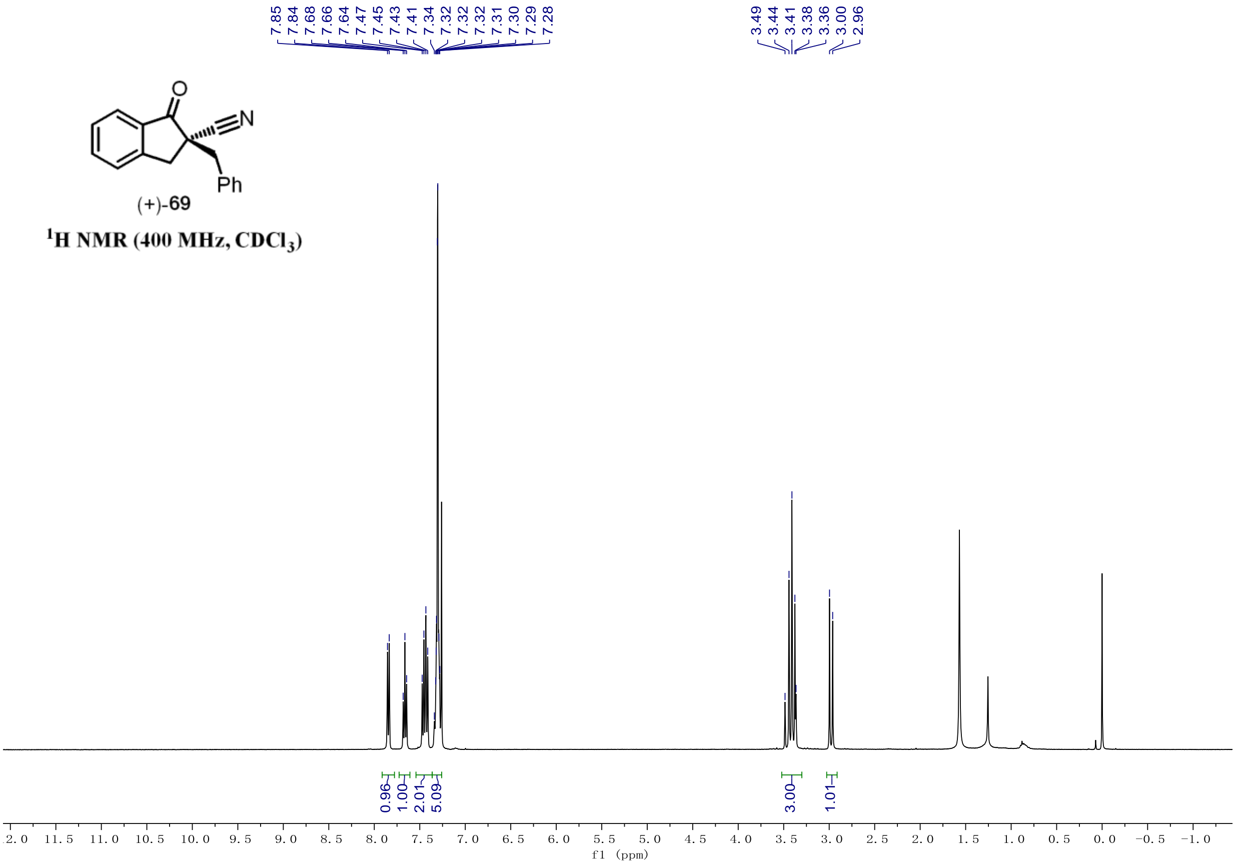
7.85 7.84 7.68 7.66 7.64 7.47 7.45 7.43 7.41 7.34 7.32 7.32 7.32 7.31 7.30 7.29 7.28
3.49 3.44 3.41 3.38 3.36 3.00 2.96
O
N
Ph
(+)-69
^{1}H NMR (400 MHz, CDCl$_3$)
0.96 1.00 2.01 5.09
3.00 1.01
f1 (ppm)

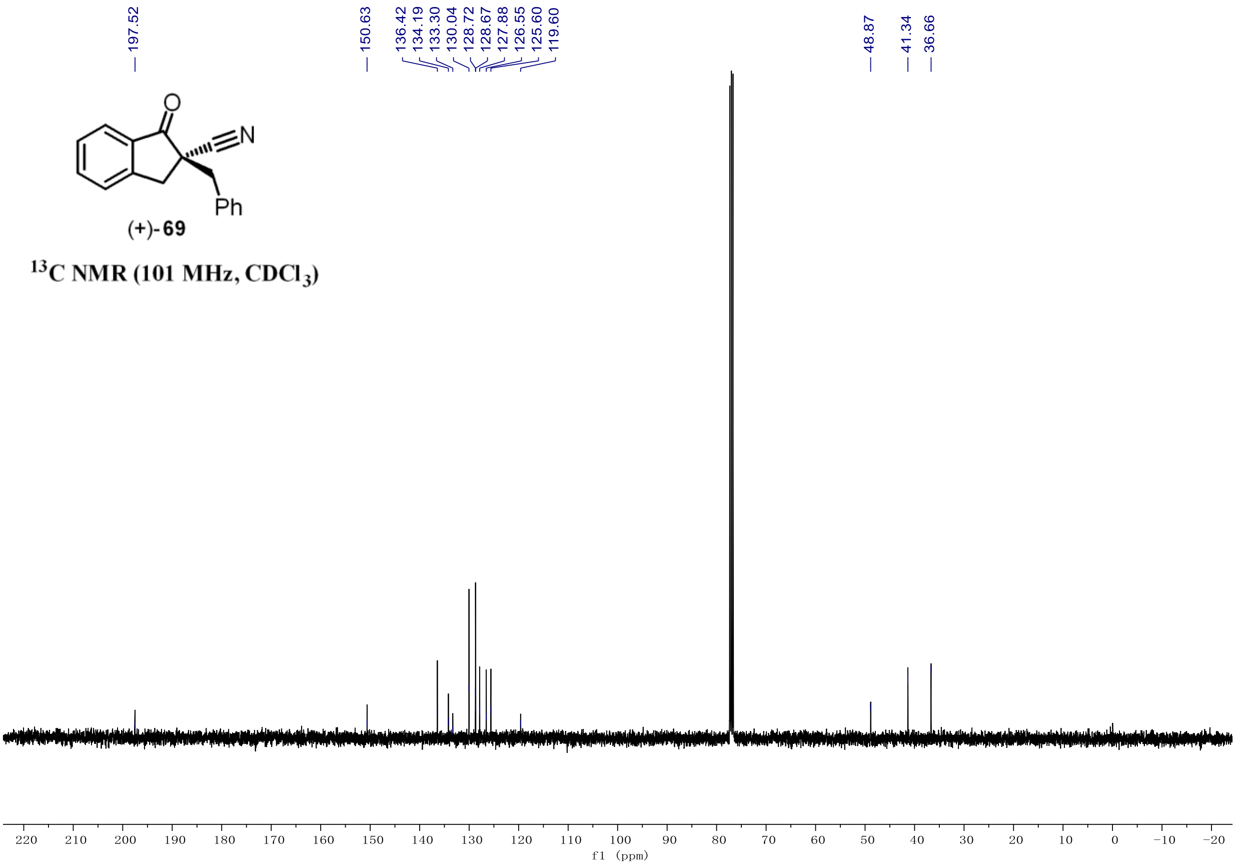
197.52
150.63
136.42 134.19 133.30 130.04 128.72 128.67 127.88 126.55 125.60 119.60
48.87 41.34 36.66
O
N
Ph
(+)-69
^{13}C NMR (101 MHz, CDCl$_3$)
f1 (ppm)

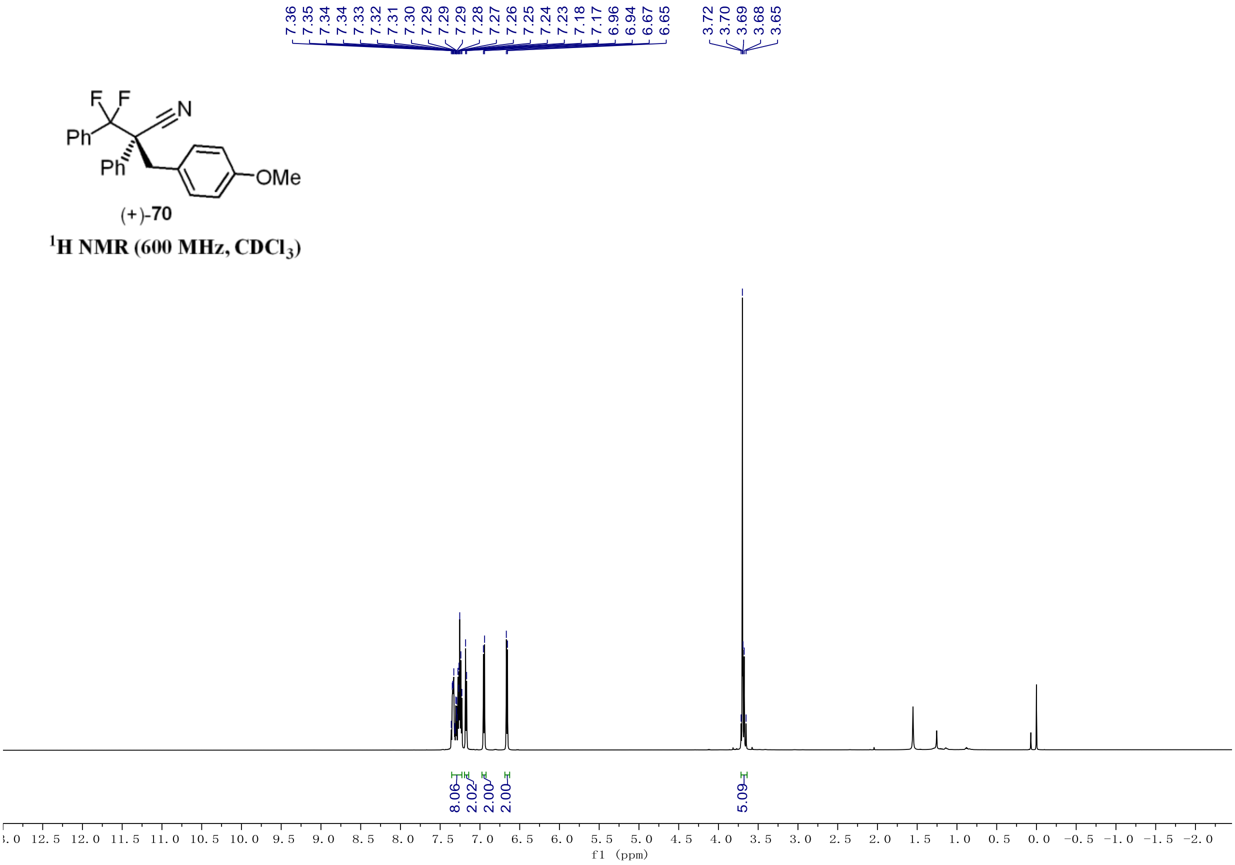

(+)-**70**

^{1}H NMR (600 MHz, $CDCl_3$)

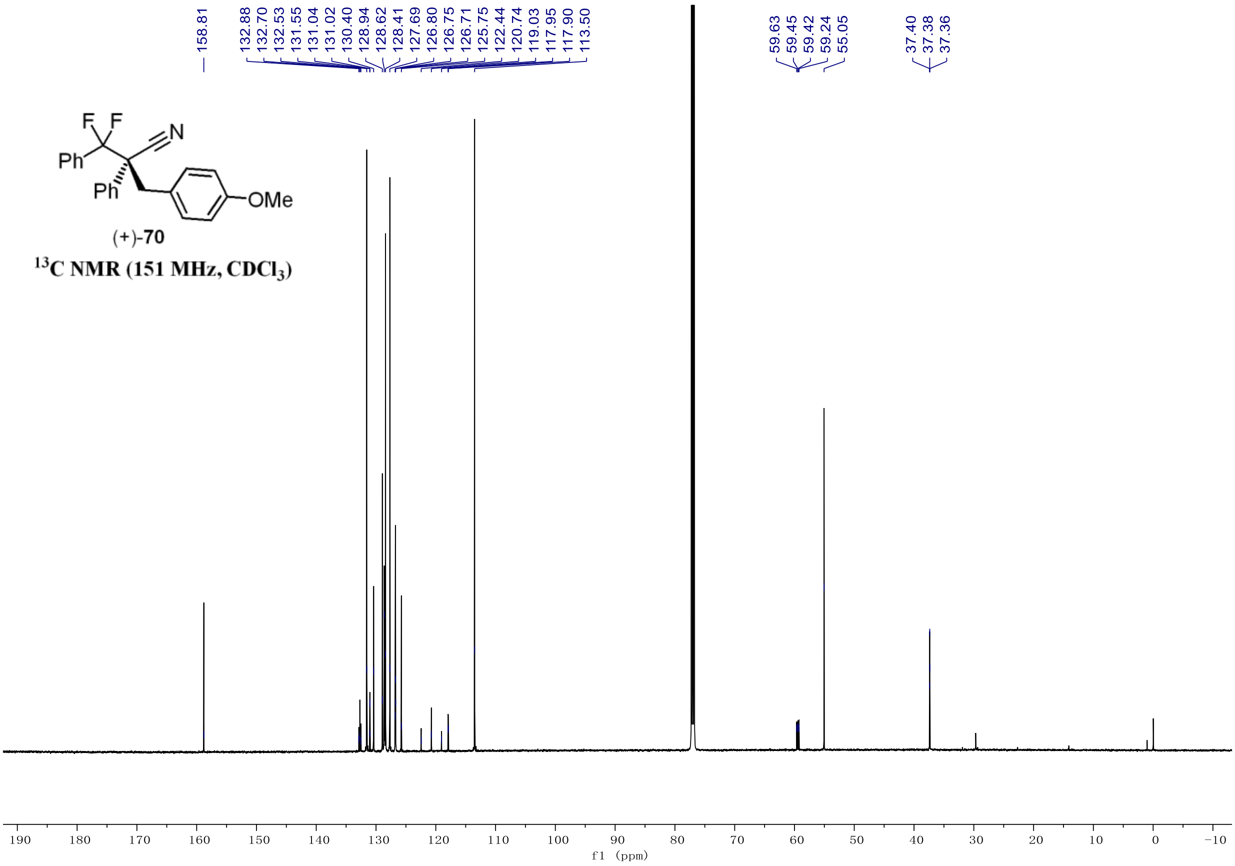

(+)-**70**

^{13}C NMR (151 MHz, $CDCl_3$)

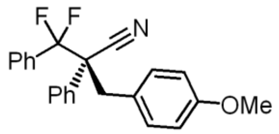

(+)-**70**

^{19}F NMR (565 MHz, CDCl$_3$)

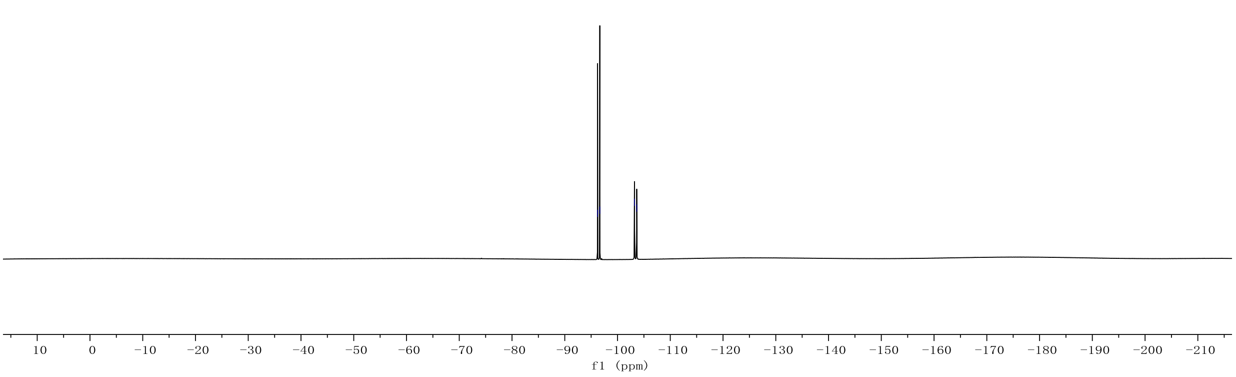

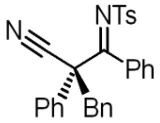

(+)-**71**

^{1}H NMR (400 MHz, CDCl$_3$)

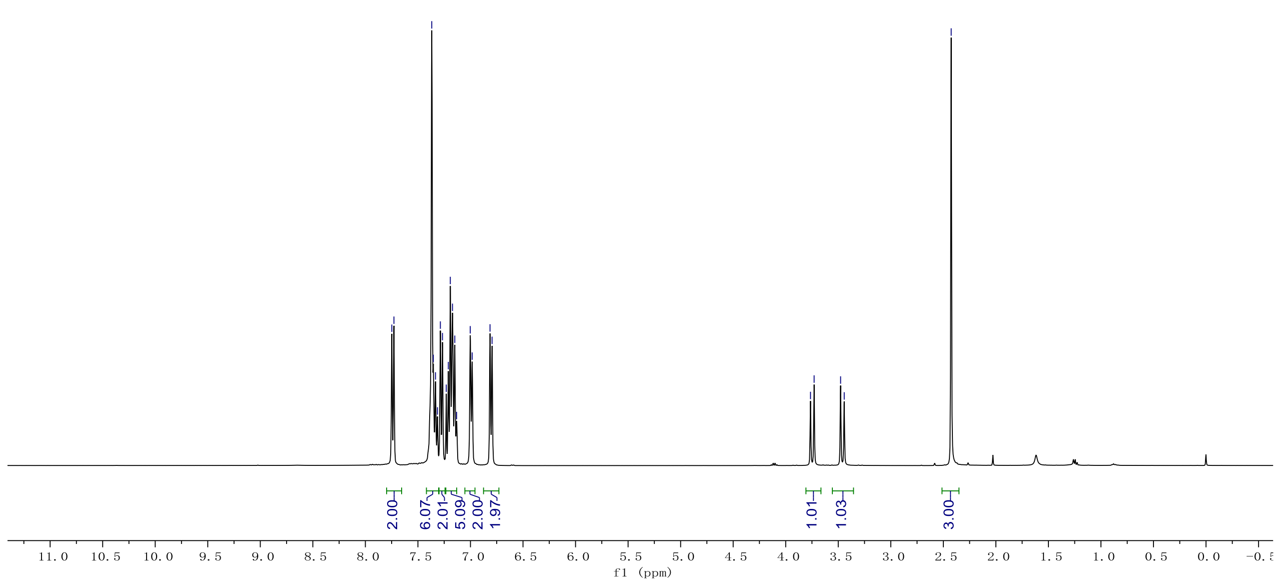

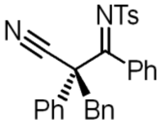

(+)-**71**

^{1}H NMR (400 MHz, CDCl$_3$)

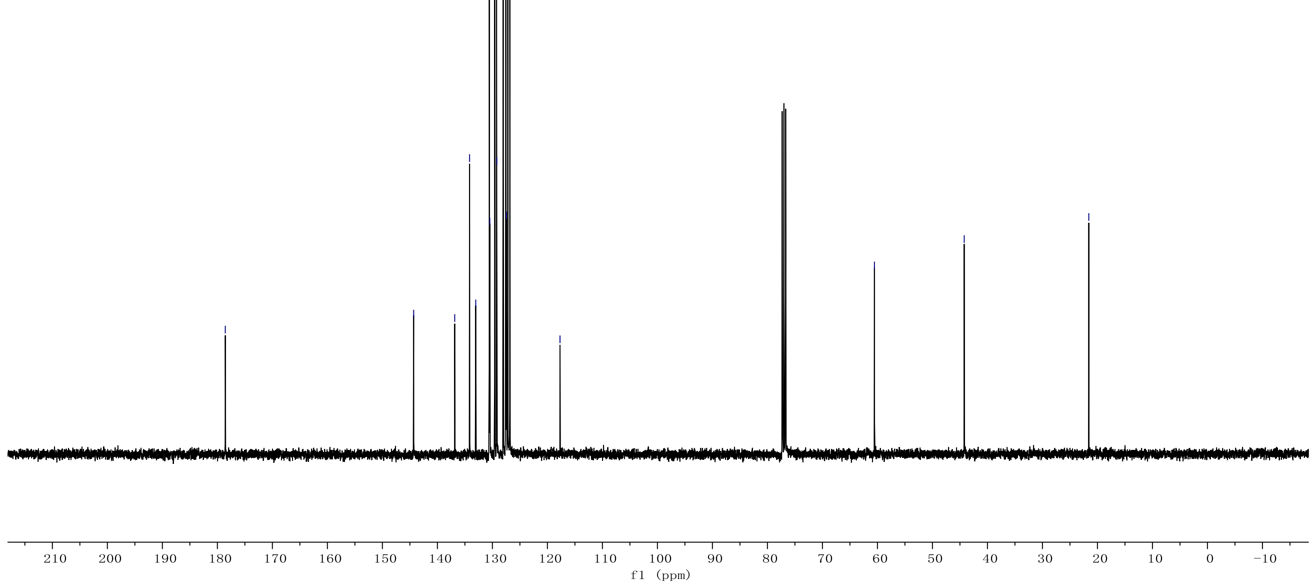

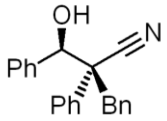

(+)-**72**

^{1}H NMR (400 MHz, $CDCl_3$)

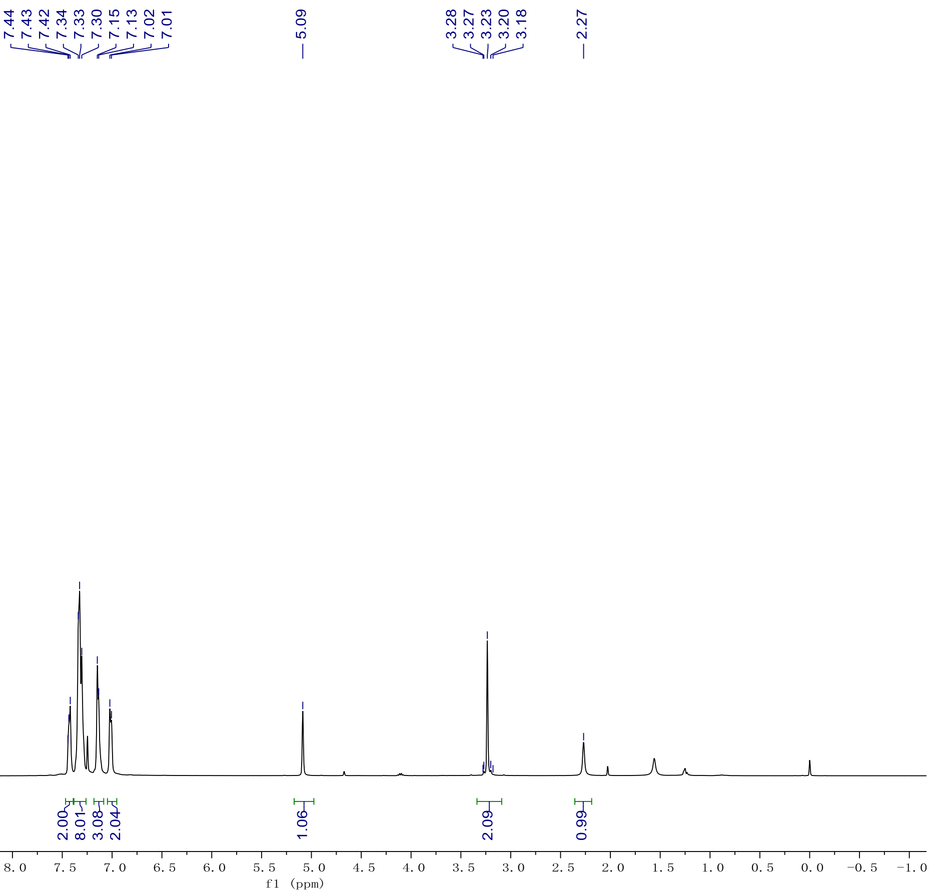

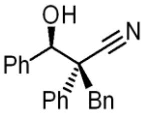

(+)-**72**

^{13}C NMR (101 MHz, CDCl$_3$)

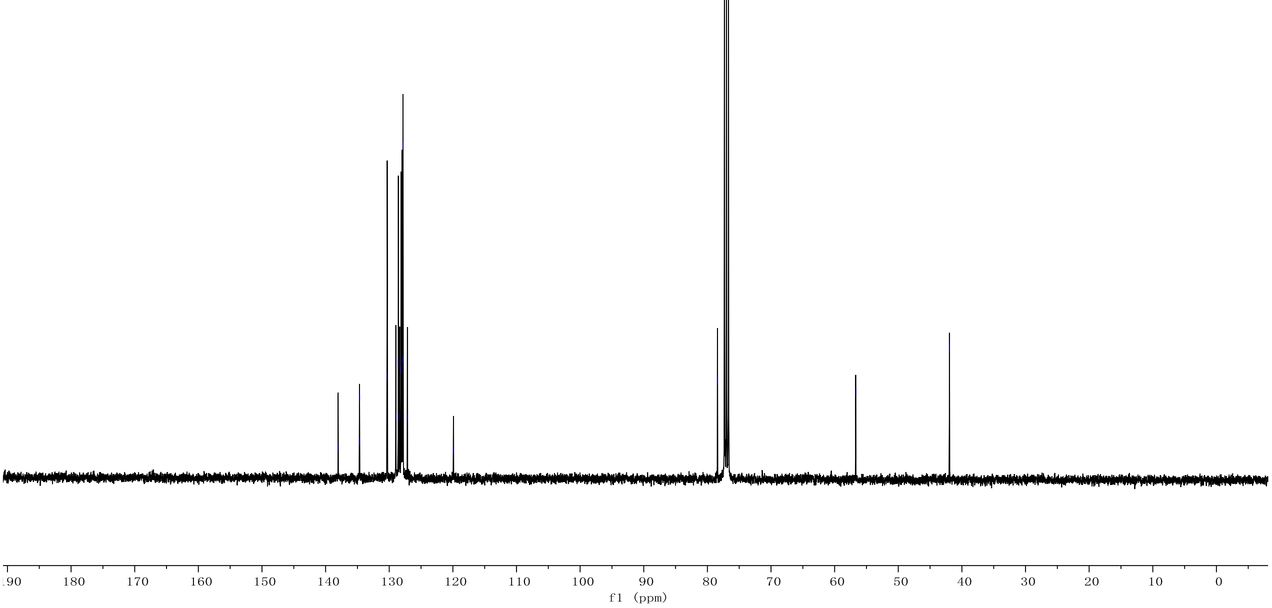

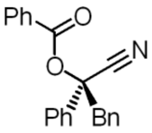

(+)-**73**

^{1}H NMR (400 MHz, CDCl$_3$)

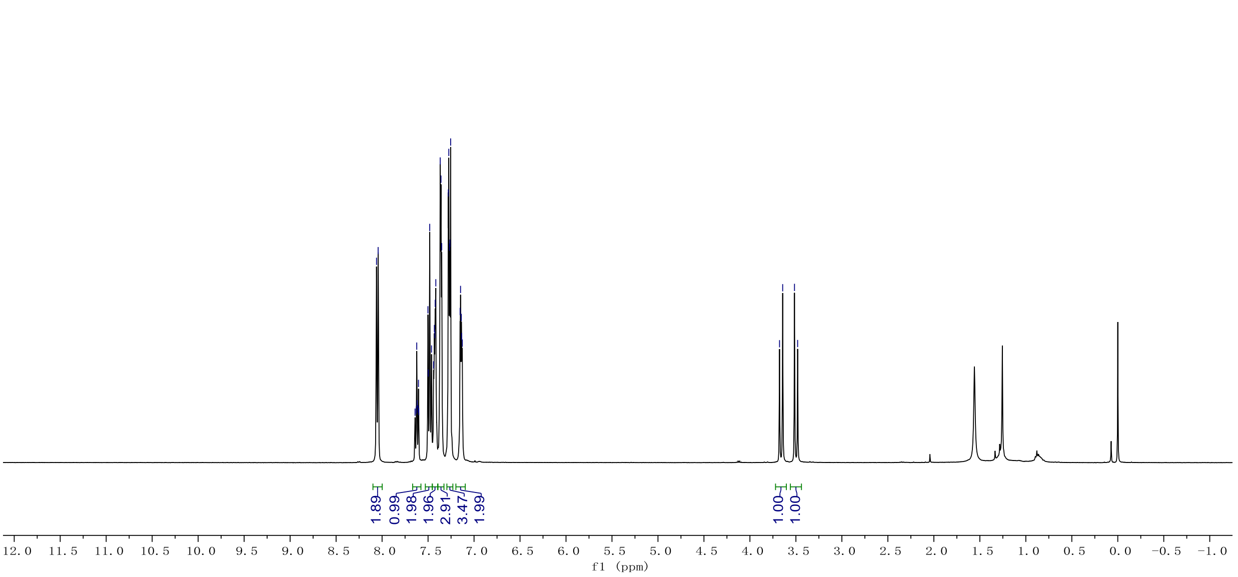

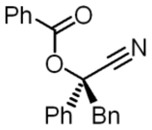

(+)-**73**

^{13}C NMR (101 MHz, CDCl$_3$)

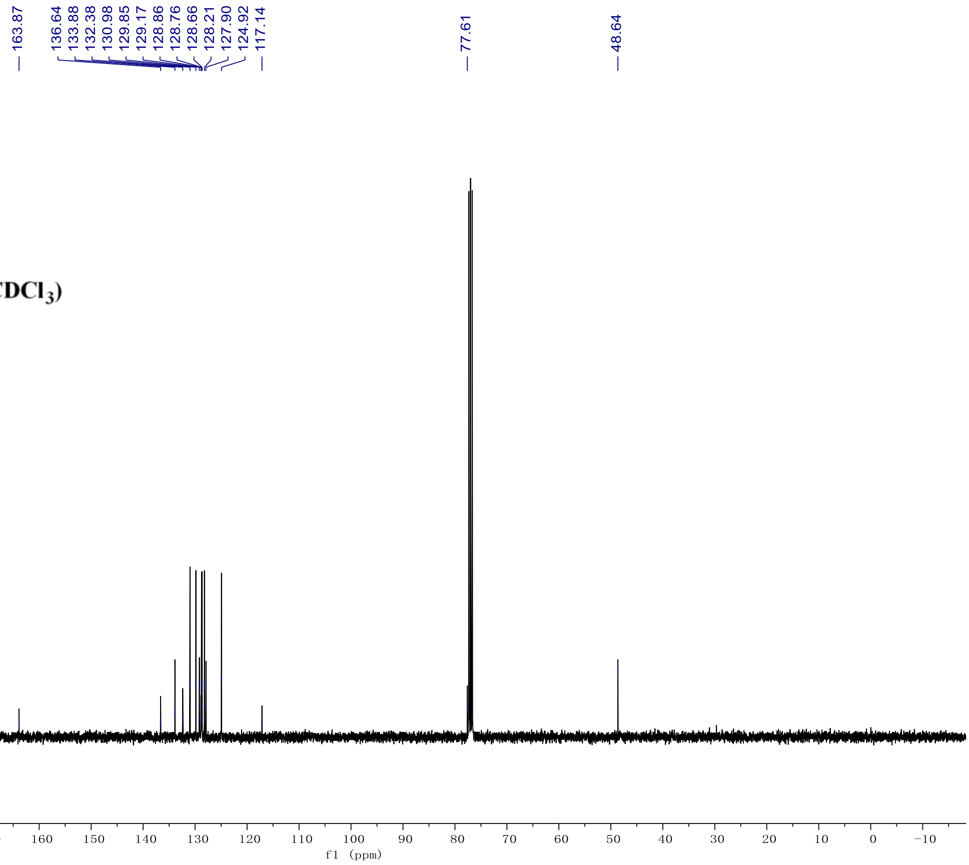

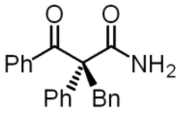

(+)-**74**

^{1}H NMR (400 MHz, CDCl$_3$)

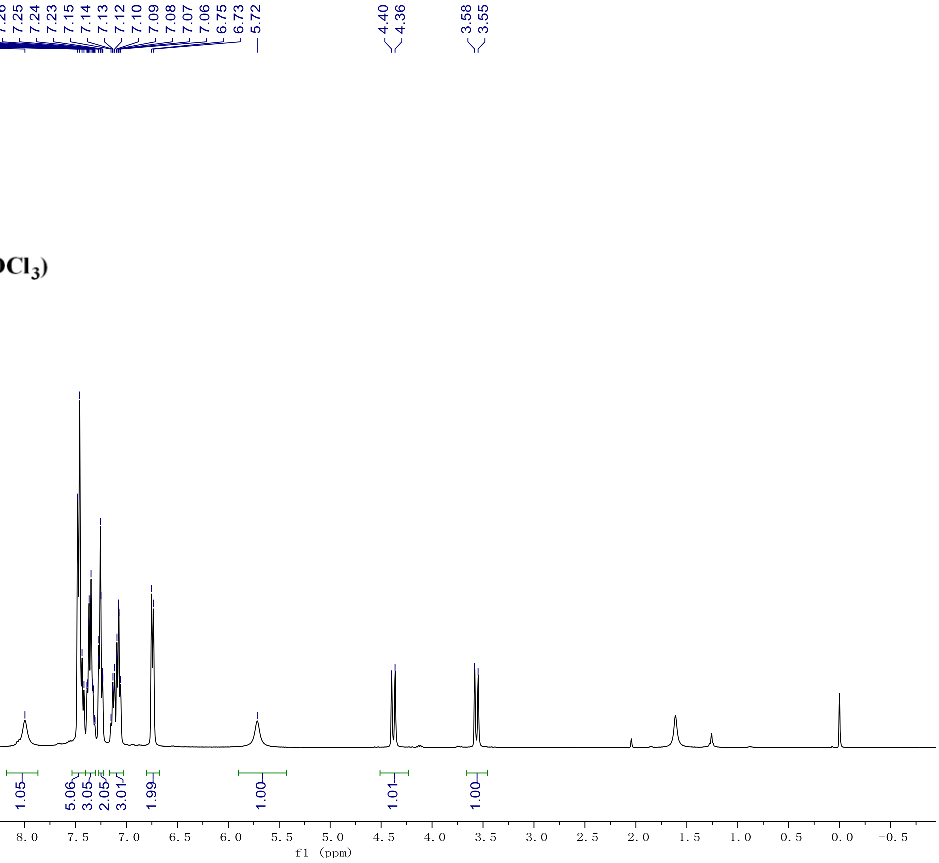

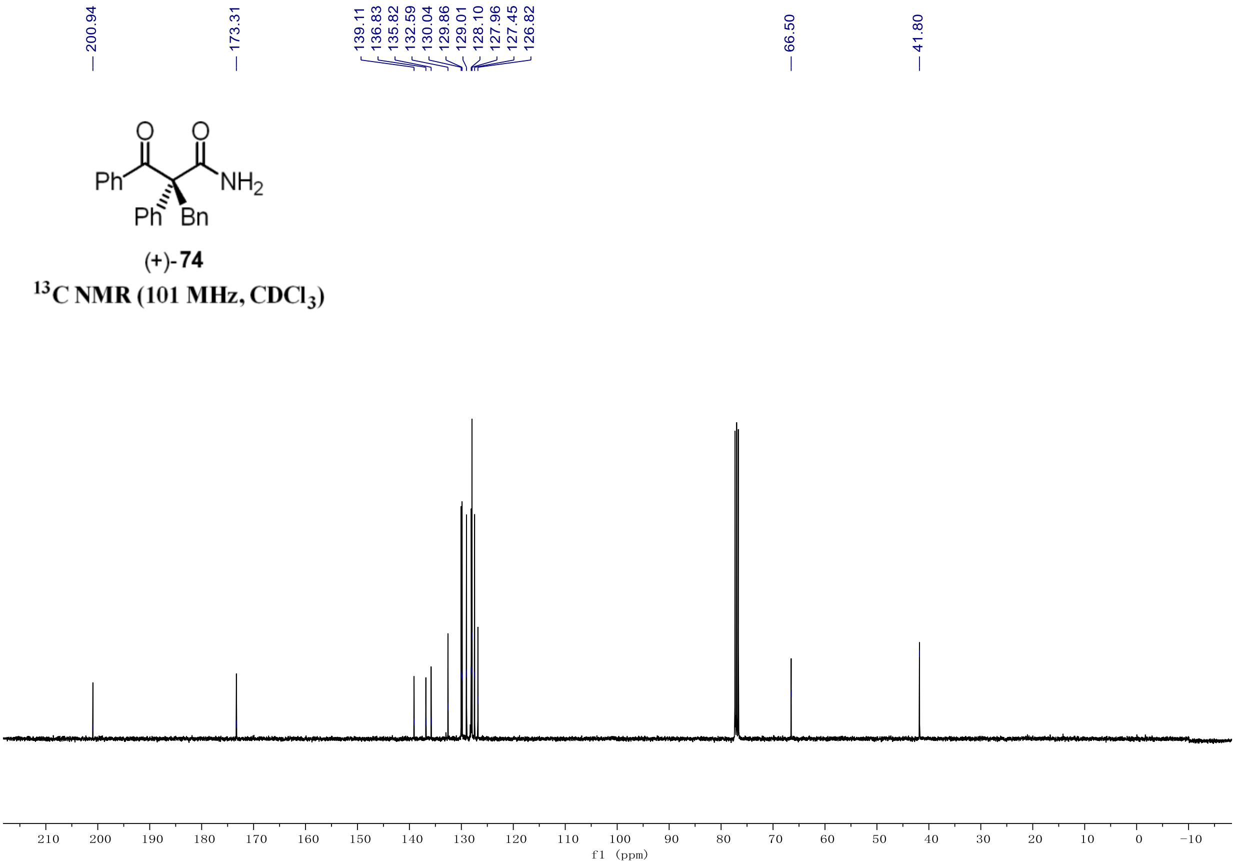
200.94
173.31
139.11
136.83
135.82
132.59
130.04
129.86
129.01
128.10
127.96
127.45
126.82
66.50
41.80
Ph
NH2
Ph
Bn
(+)-74
13C NMR (101 MHz, CDCl3)
f1 (ppm)

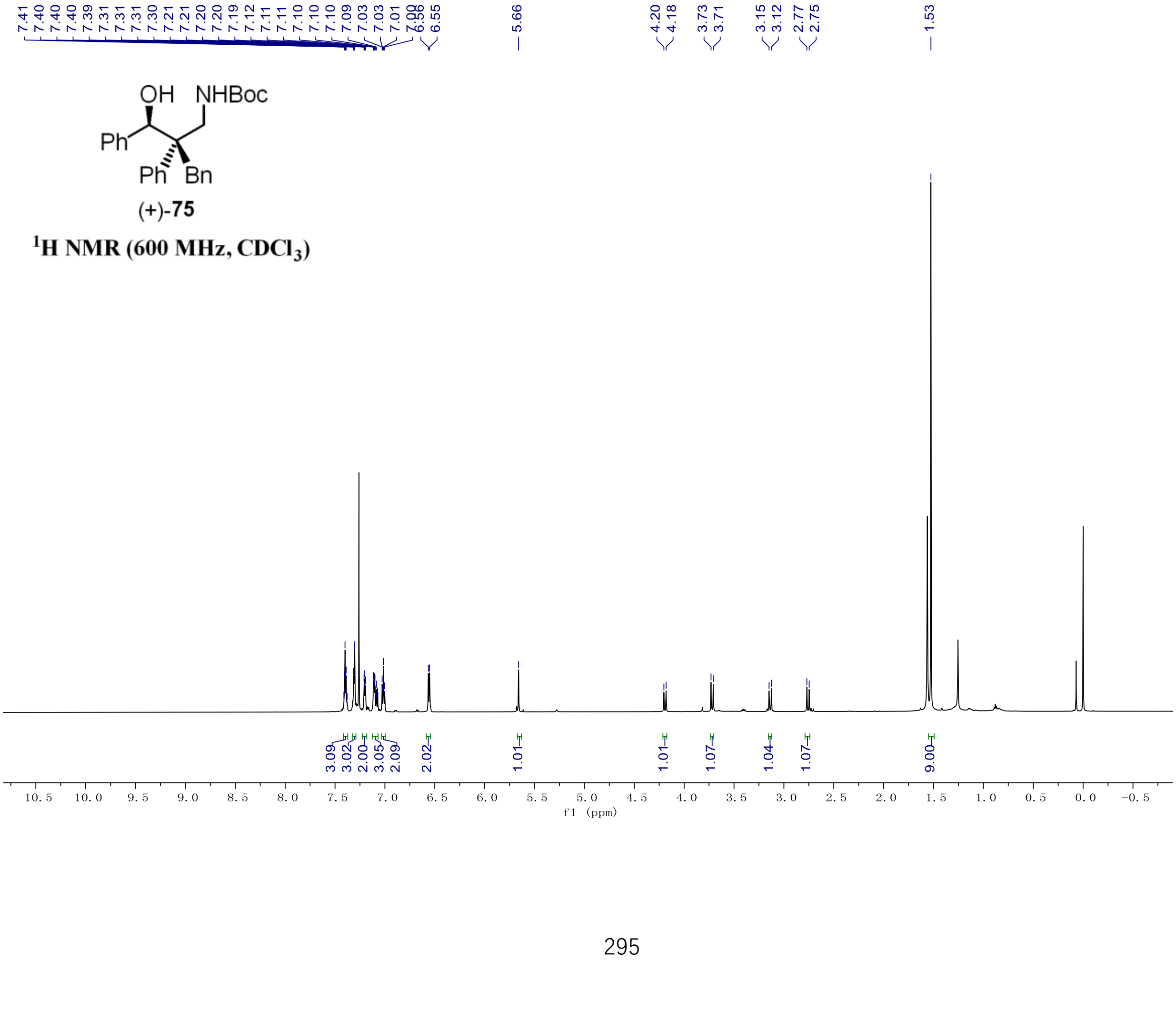
7.41
7.40
7.40
7.39
7.31
7.31
7.30
7.21
7.21
7.20
7.20
7.19
7.12
7.11
7.11
7.10
7.10
7.09
7.03
7.03
7.01
7.00
6.56
6.55
5.66
4.20
4.18
3.73
3.71
3.15
3.12
2.77
2.75
1.53
OH
NHBoc
Ph
Ph
Bn
(+)-75
1H NMR (600 MHz, CDCl3)
3.09
3.02
2.00
3.05
2.09
2.02
1.01
1.01
1.07
1.04
1.07
9.00
f1 (ppm)

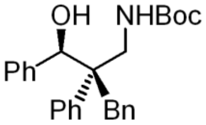
(+)-**75**

^{13}C NMR (151 MHz, $CDCl_3$)

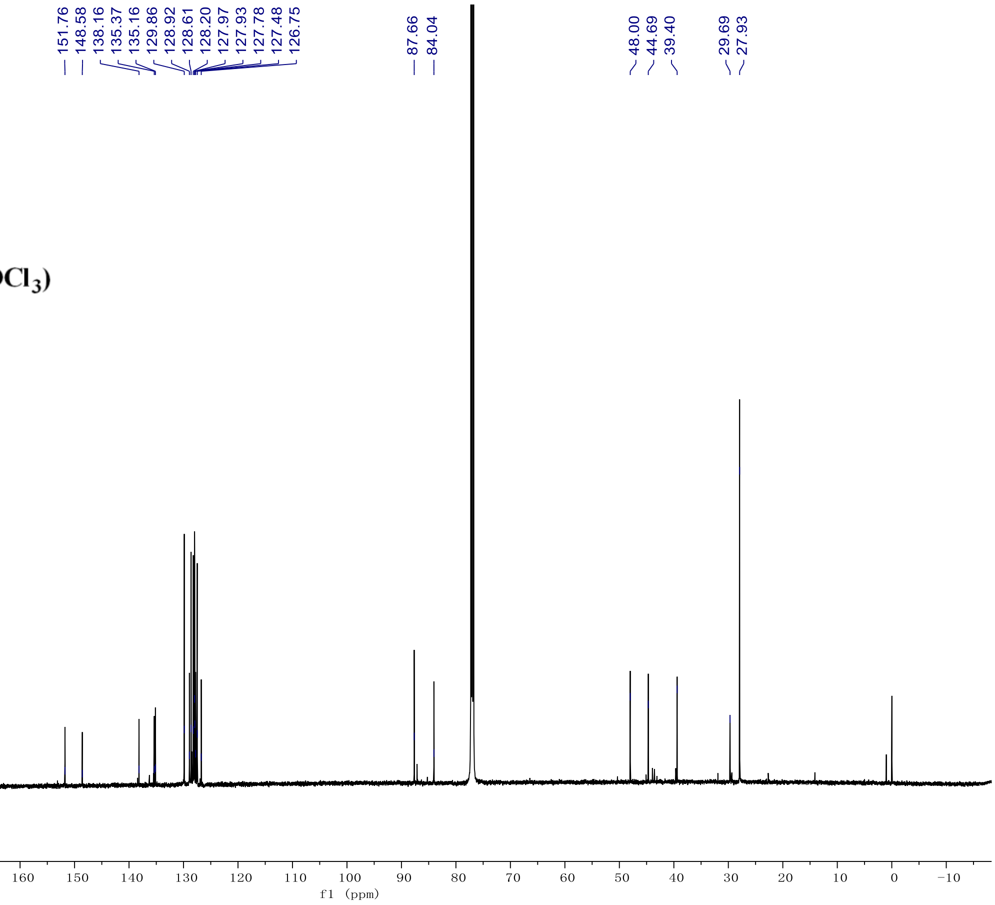

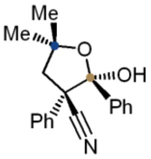
(–)-**76**

^{1}H NMR (400 MHz, $CDCl_3$)

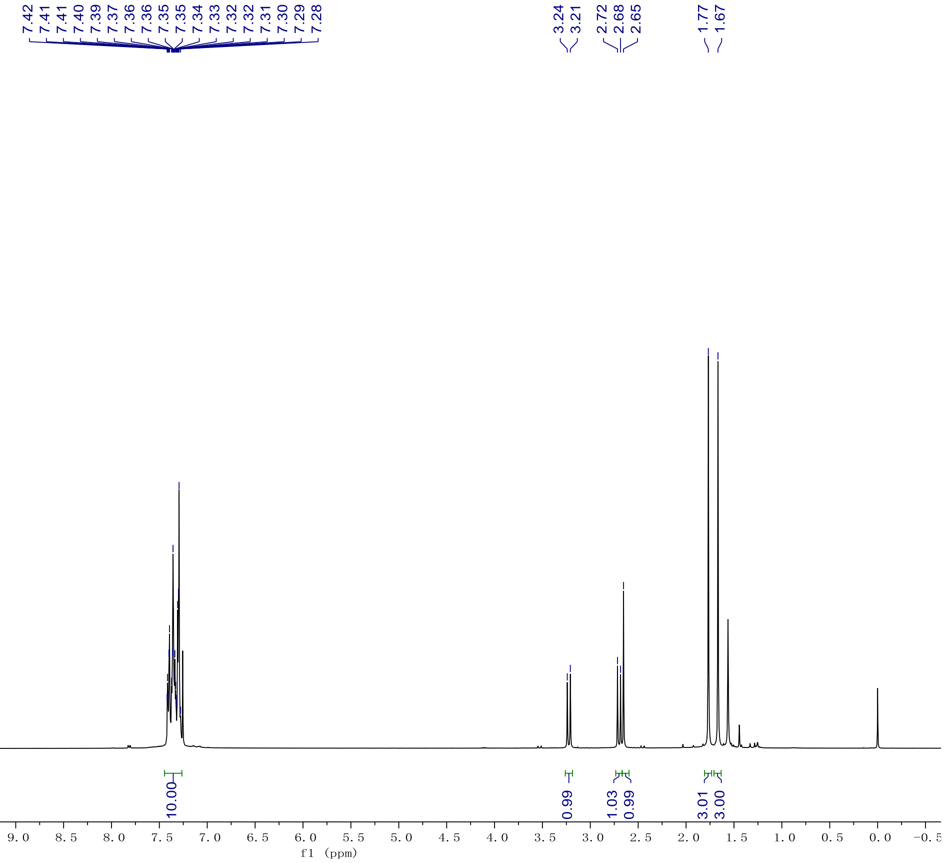

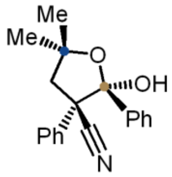

(–)-**76**

^{13}C NMR (101 MHz, CDCl$_3$)

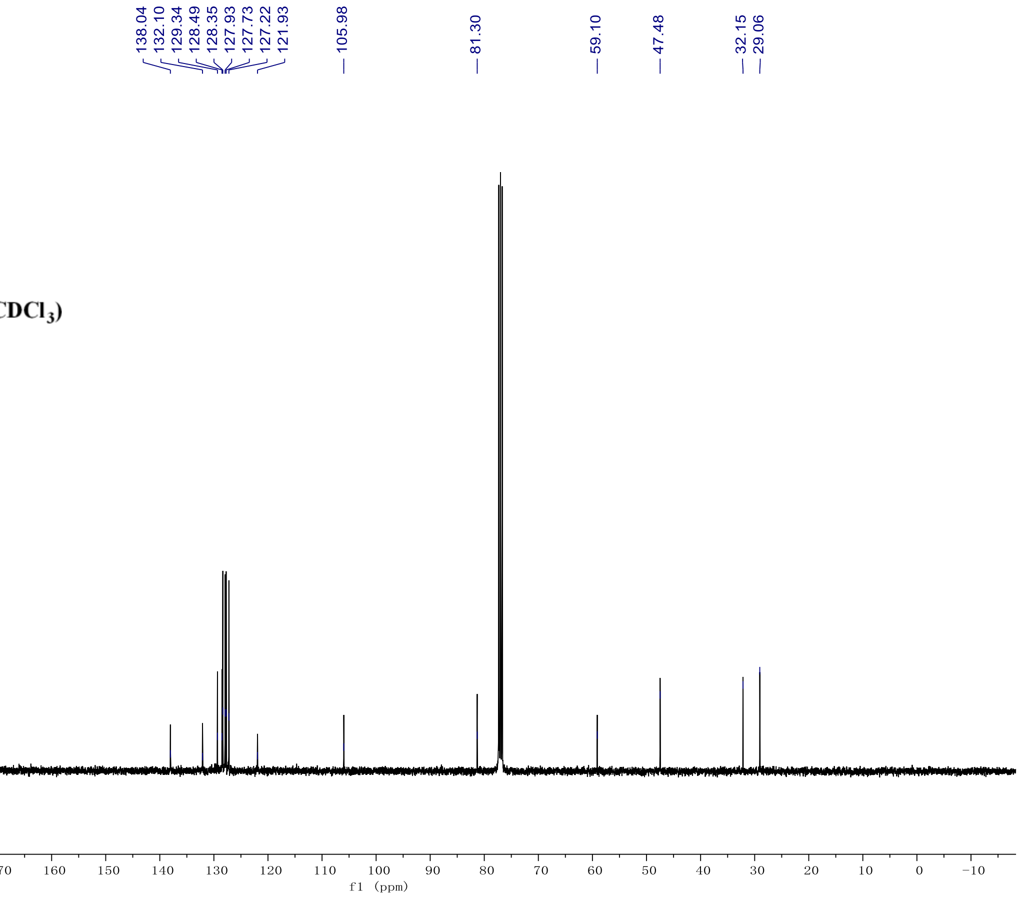

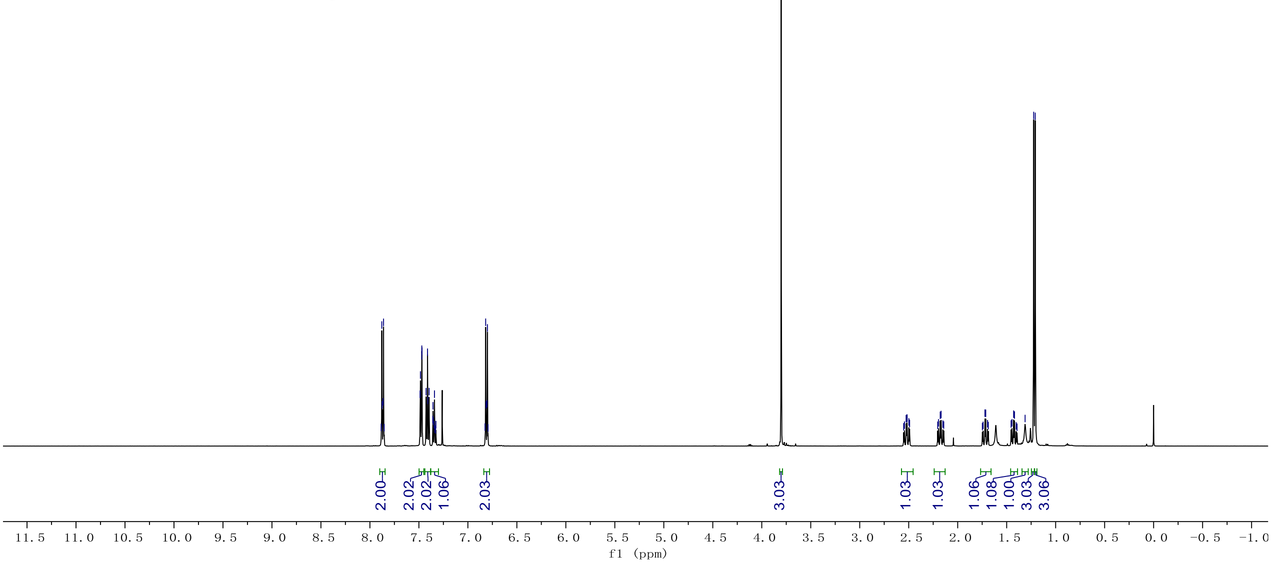

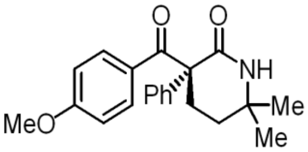

(+)-**77**

^{1}H NMR (500 MHz, $CDCl_3$)

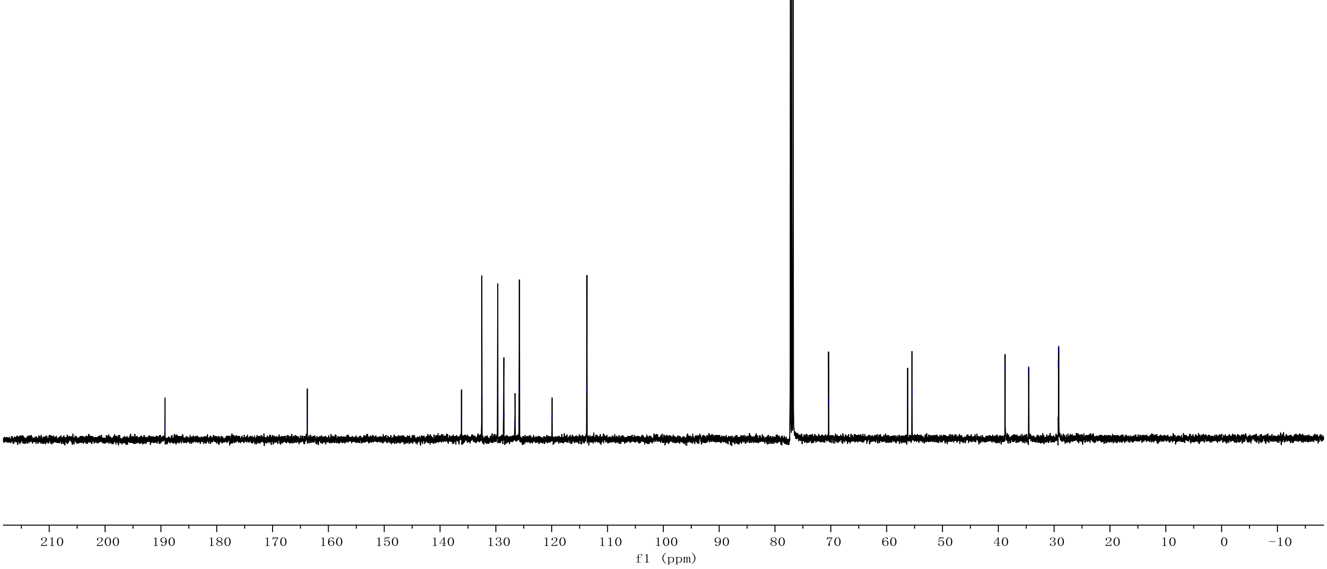

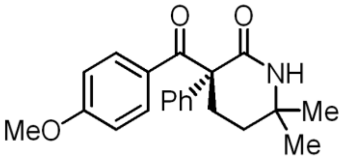

(+)-**77**

^{13}C NMR (126 MHz, $CDCl_3$)

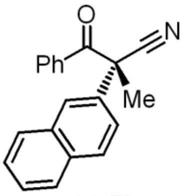

(+)-**78**

^{1}H NMR (400 MHz, $CDCl_3$)

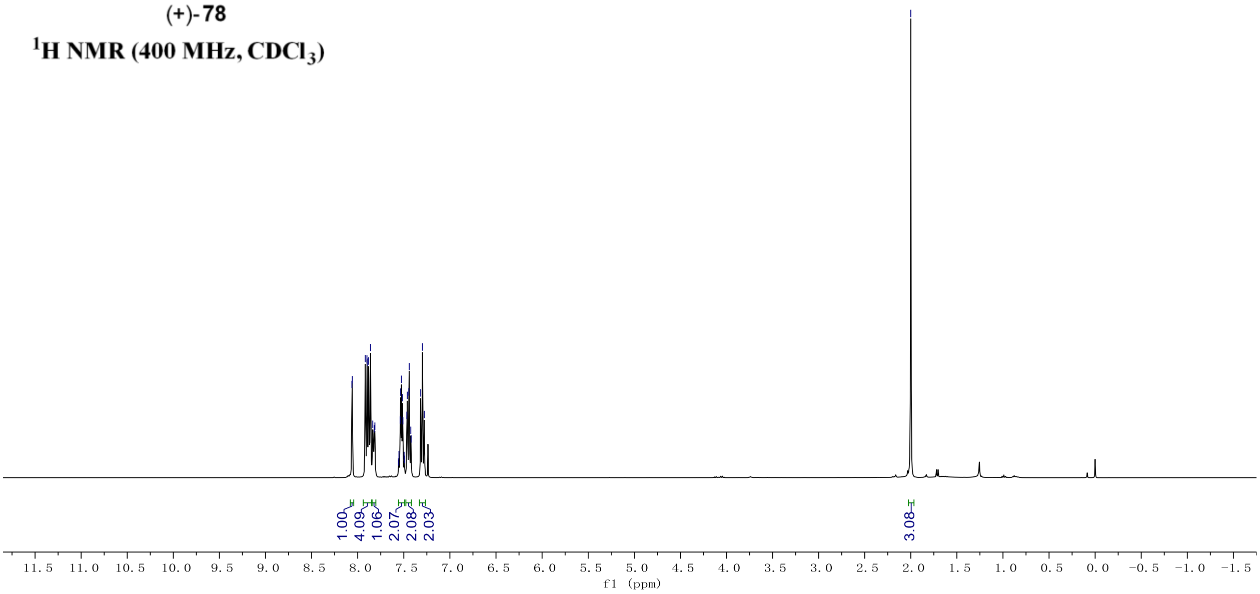

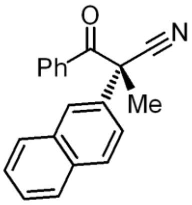

(+)-**78**

^{13}C NMR (101 MHz, $CDCl_3$)

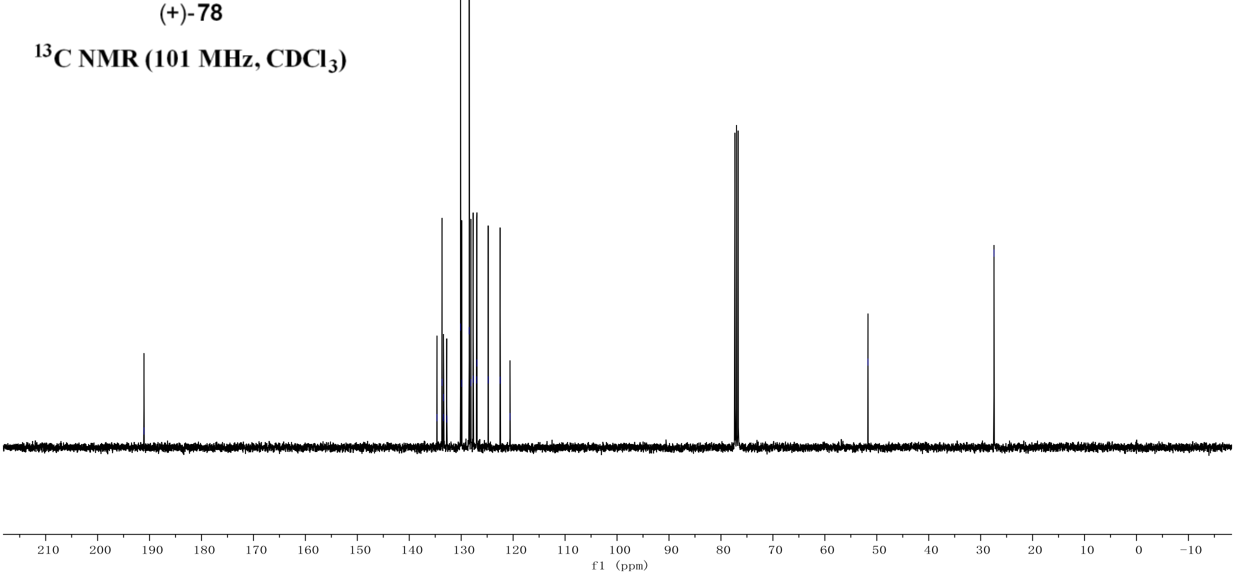

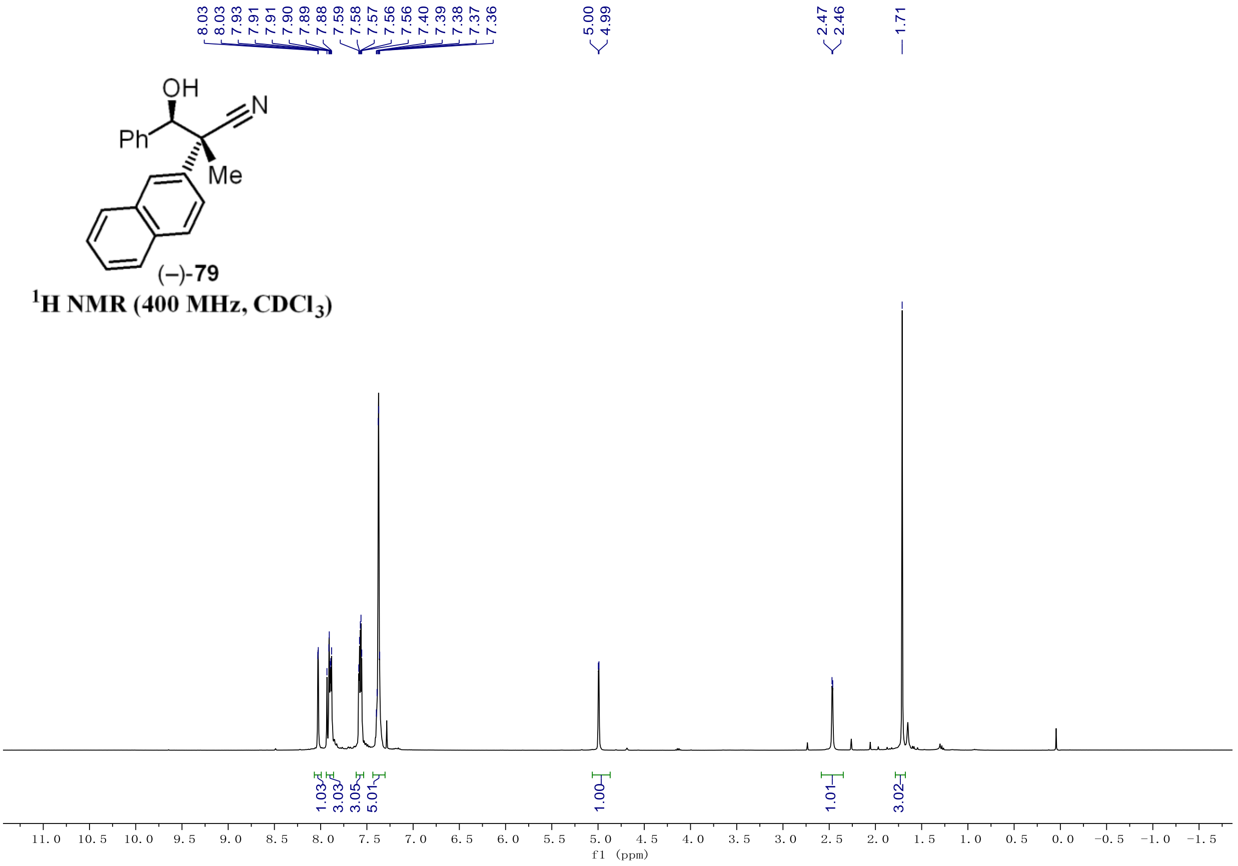
8.03 8.03 7.93 7.91 7.91 7.90 7.89 7.88 7.59 7.58 7.57 7.56 7.56 7.40 7.39 7.38 7.37 7.36
5.00 4.99
2.47 2.46
1.71
OH
Ph
N
Me
(–)-79
¹H NMR (400 MHz, CDCl₃)
1.03 3.03 3.05 5.01
1.00
1.01
3.02
11.0 10.5 10.0 9.5 9.0 8.5 8.0 7.5 7.0 6.5 6.0 5.5 5.0 4.5 4.0 3.5 3.0 2.5 2.0 1.5 1.0 0.5 0.0 −0.5 −1.0 −1.5
f1 (ppm)

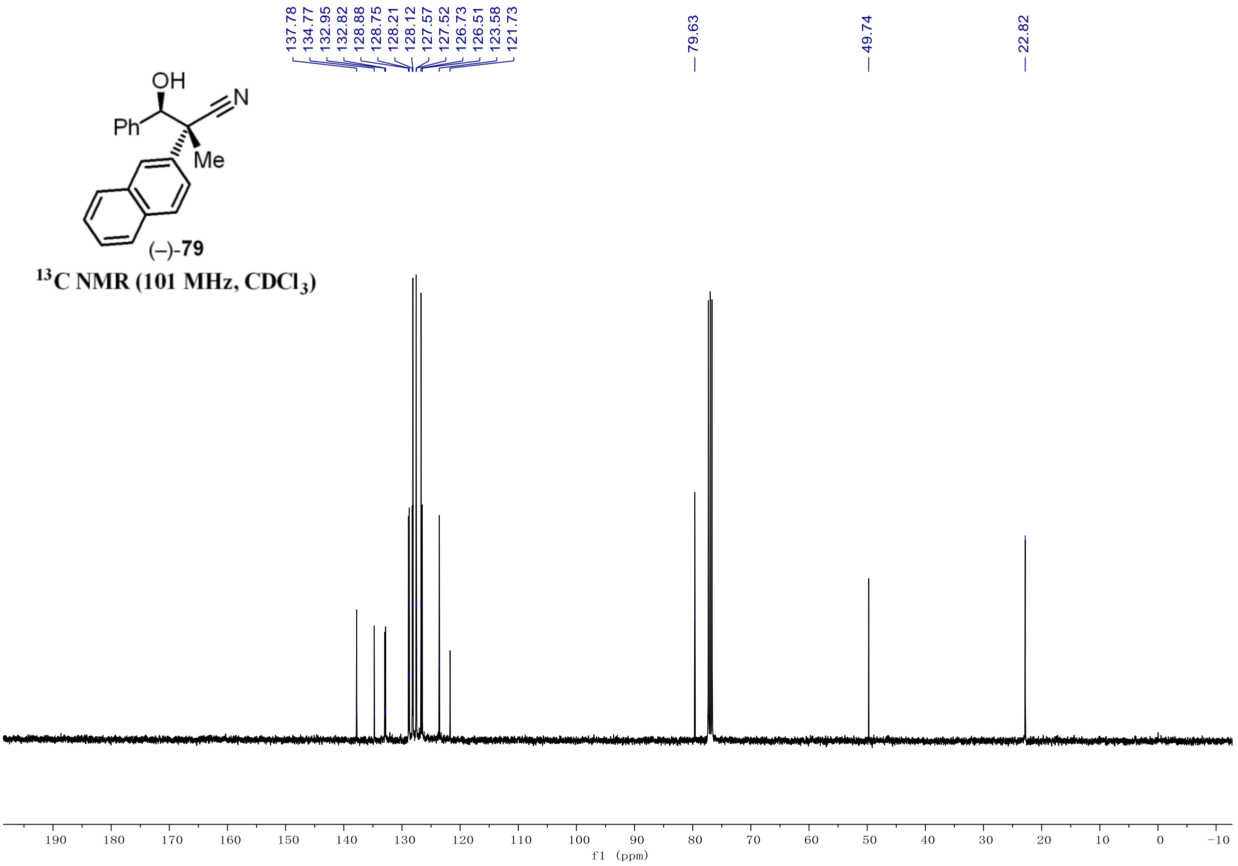
137.78 134.77 132.95 132.82 128.88 128.75 128.21 128.12 127.57 127.52 126.73 126.51 123.58 121.73
79.63
49.74
22.82
OH
Ph
N
Me
(–)-79
¹³C NMR (101 MHz, CDCl₃)
190 180 170 160 150 140 130 120 110 100 90 80 70 60 50 40 30 20 10 0 −10
f1 (ppm)

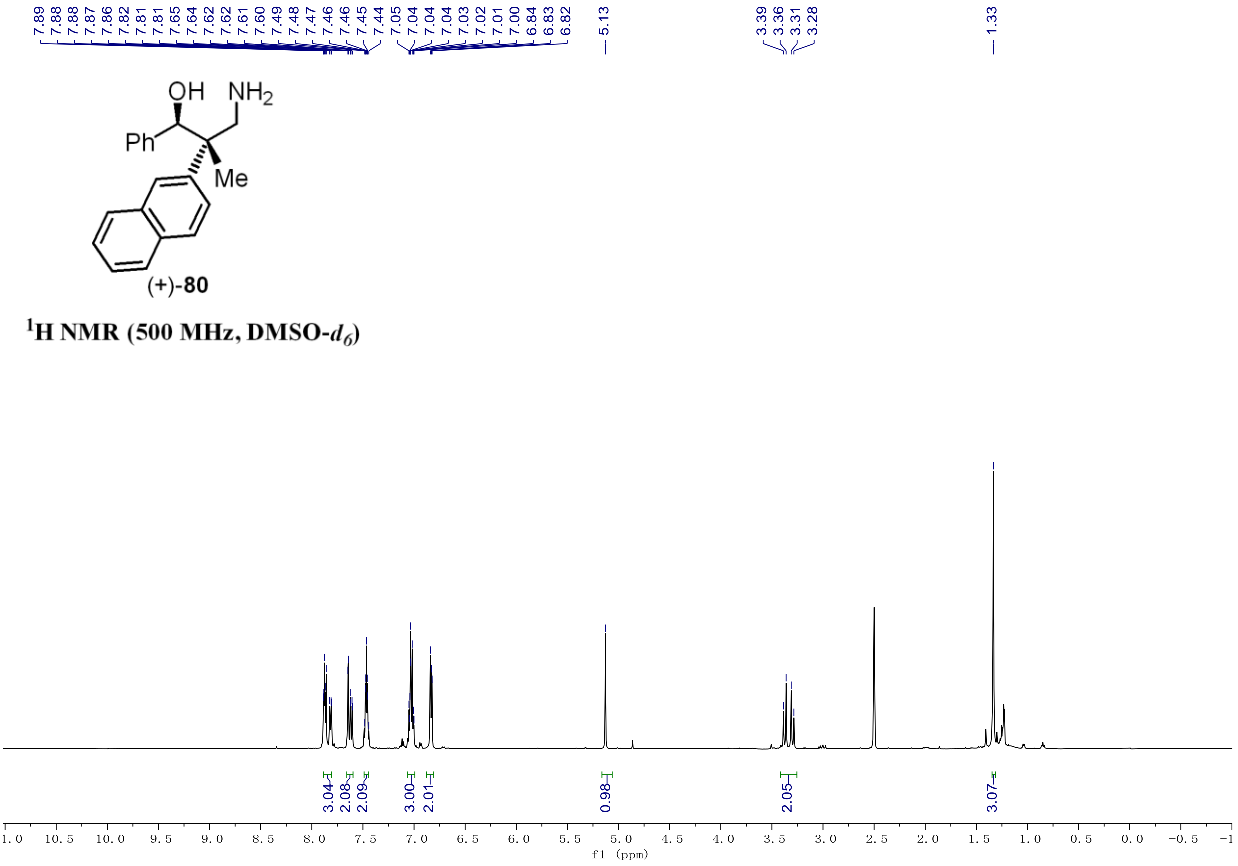

^{1}H NMR (500 MHz, DMSO-d_6)

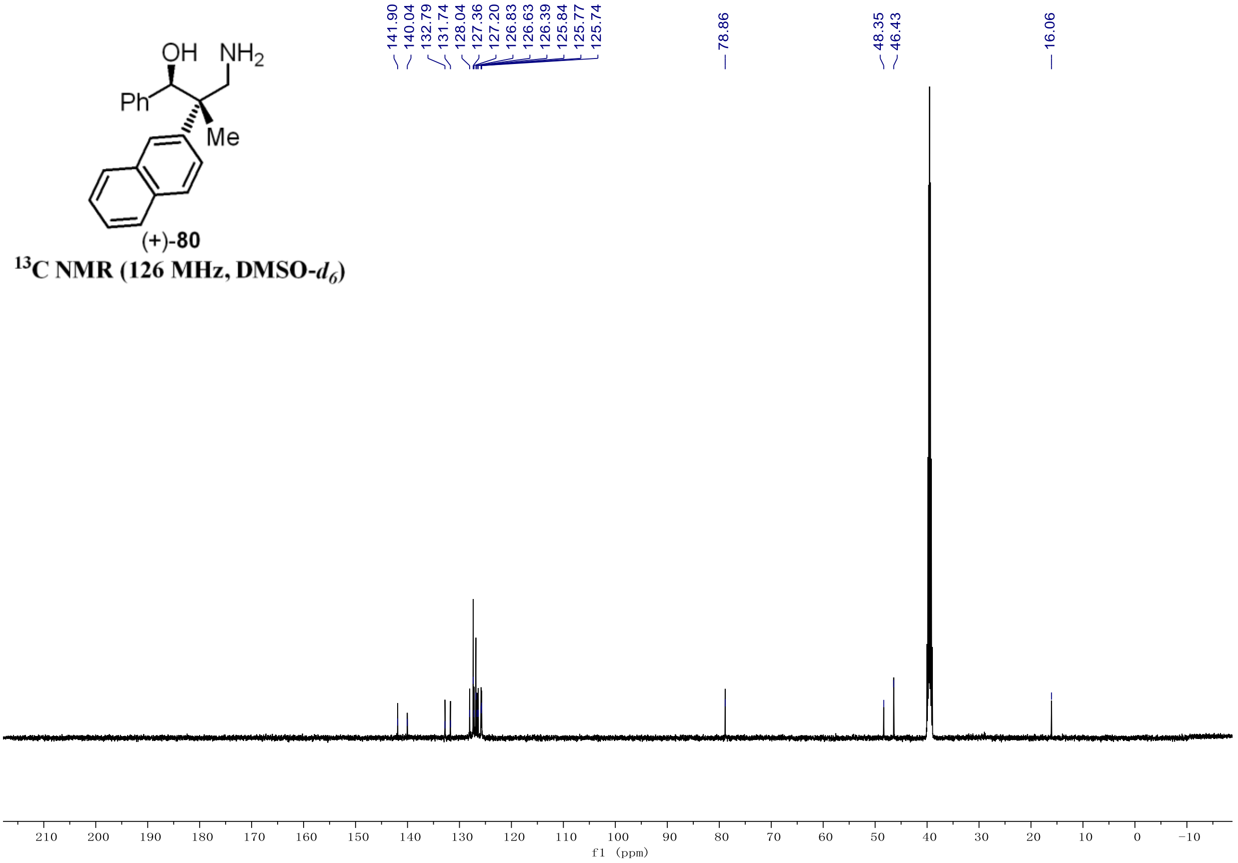

^{13}C NMR (126 MHz, DMSO-d_6)

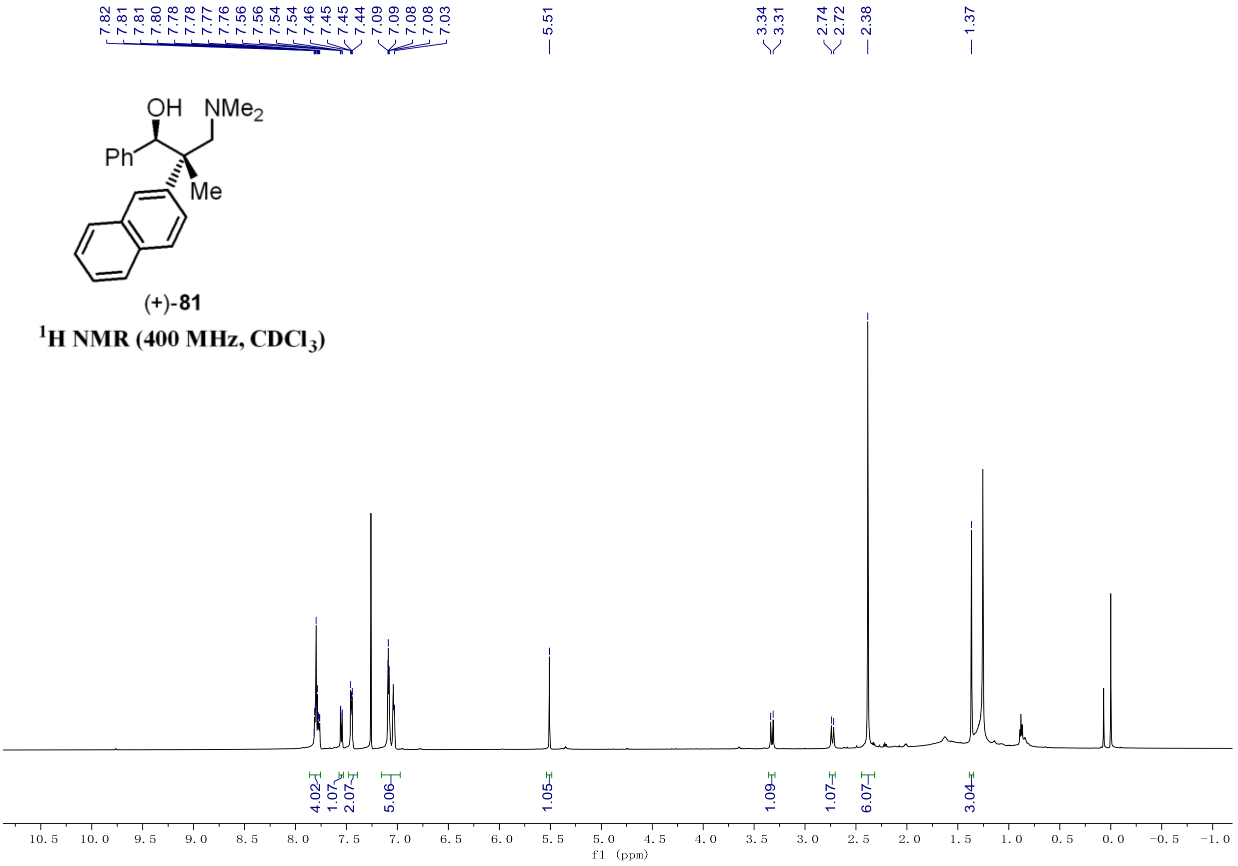

(+)-**81**

^{1}H NMR (400 MHz, CDCl$_3$)

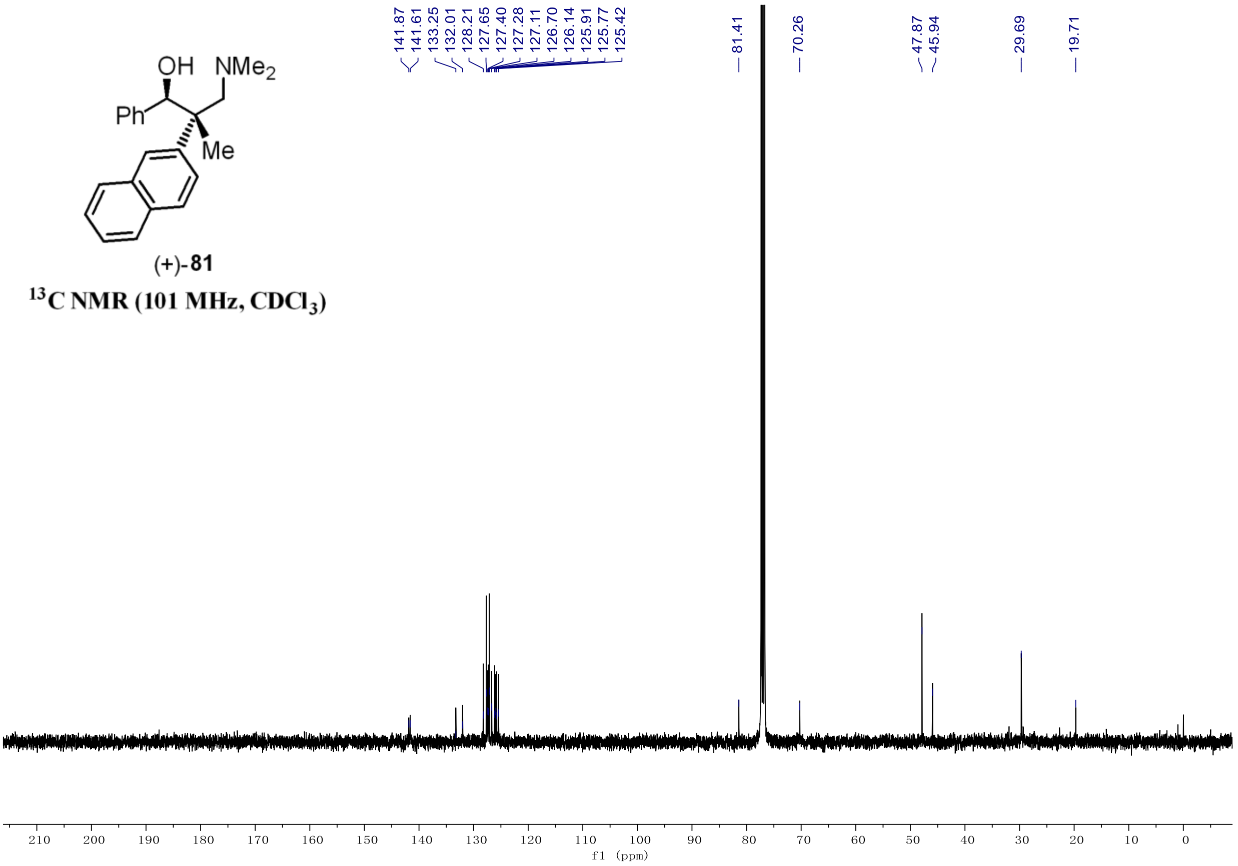

(+)-**81**

^{13}C NMR (101 MHz, CDCl$_3$)

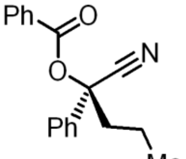

(+)-**82**

^{1}H NMR (600 MHz, $CDCl_3$)

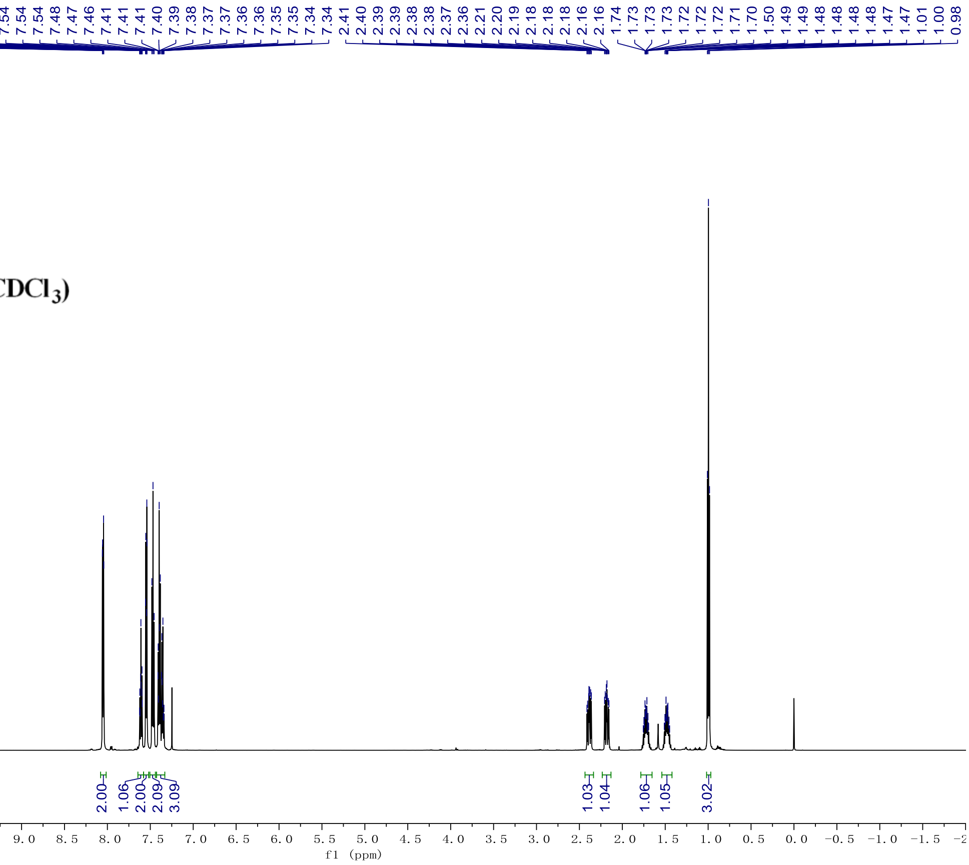

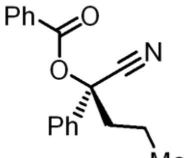

(+)-**82**

^{13}C NMR (151 MHz, $CDCl_3$)

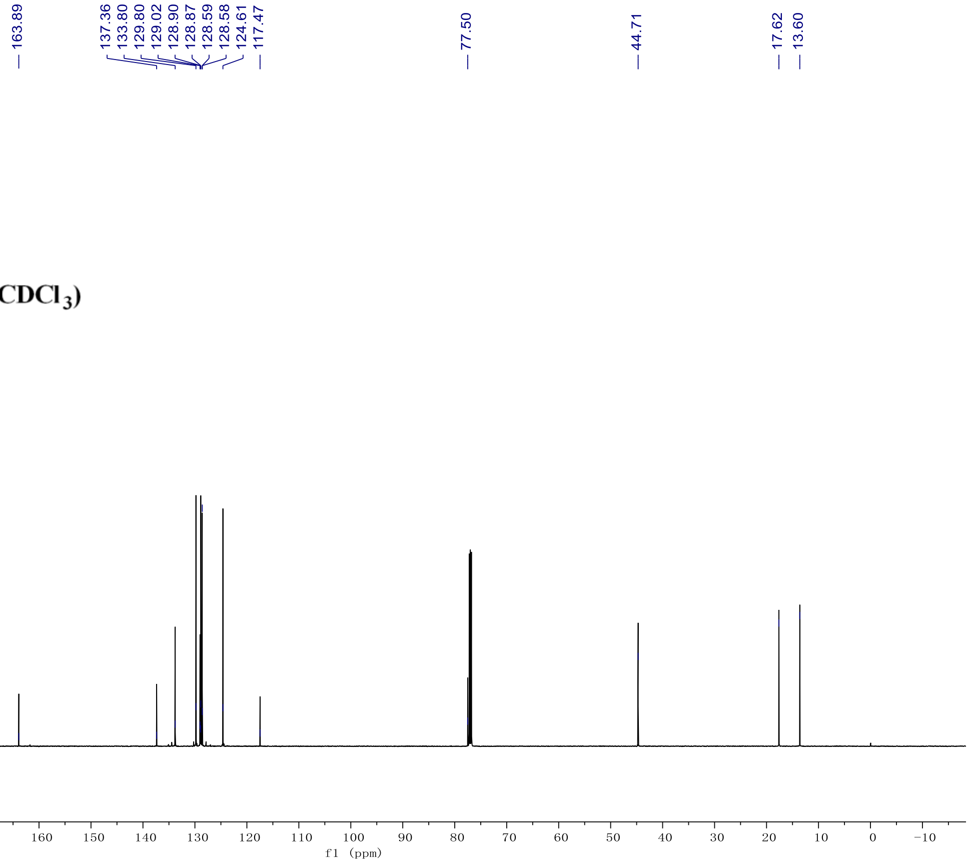

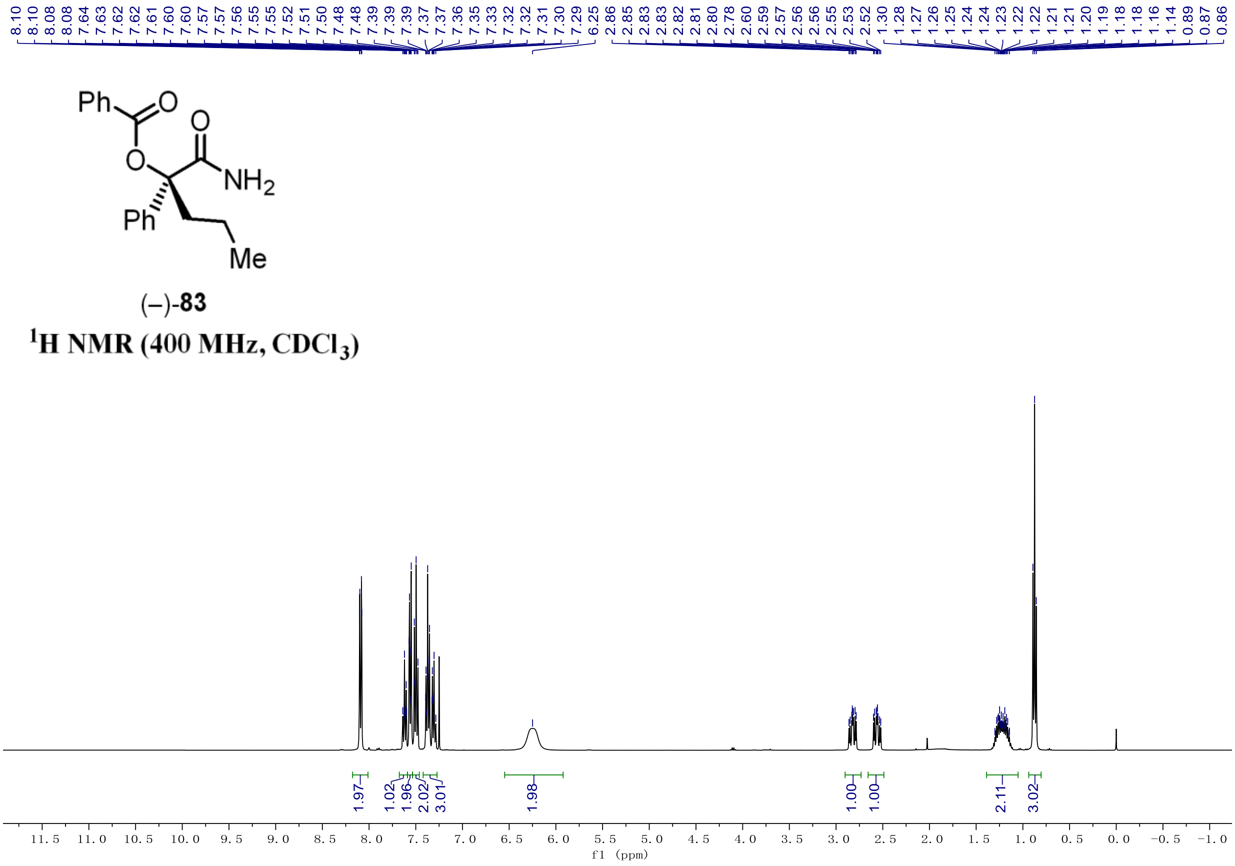
8.10 8.10 8.08 8.08 7.64 7.63 7.62 7.62 7.61 7.60 7.57 7.57 7.56 7.55 7.55 7.52 7.51 7.50 7.48 7.48 7.39 7.39 7.39 7.37 7.37 7.36 7.35 7.33 7.32 7.32 7.31 7.30 7.29 6.25 2.86 2.85 2.83 2.83 2.82 2.81 2.80 2.78 2.60 2.59 2.57 2.56 2.55 2.53 2.52 1.30 1.28 1.27 1.26 1.25 1.24 1.24 1.23 1.22 1.22 1.21 1.21 1.20 1.19 1.18 1.16 1.14 0.89 0.87 0.86
Ph
O
O
O
NH2
Ph
Me
(–)-83
1H NMR (400 MHz, CDCl3)
1.97 1.02 1.96 2.02 3.01 1.98 1.00 1.00 2.11 3.02
f1 (ppm)

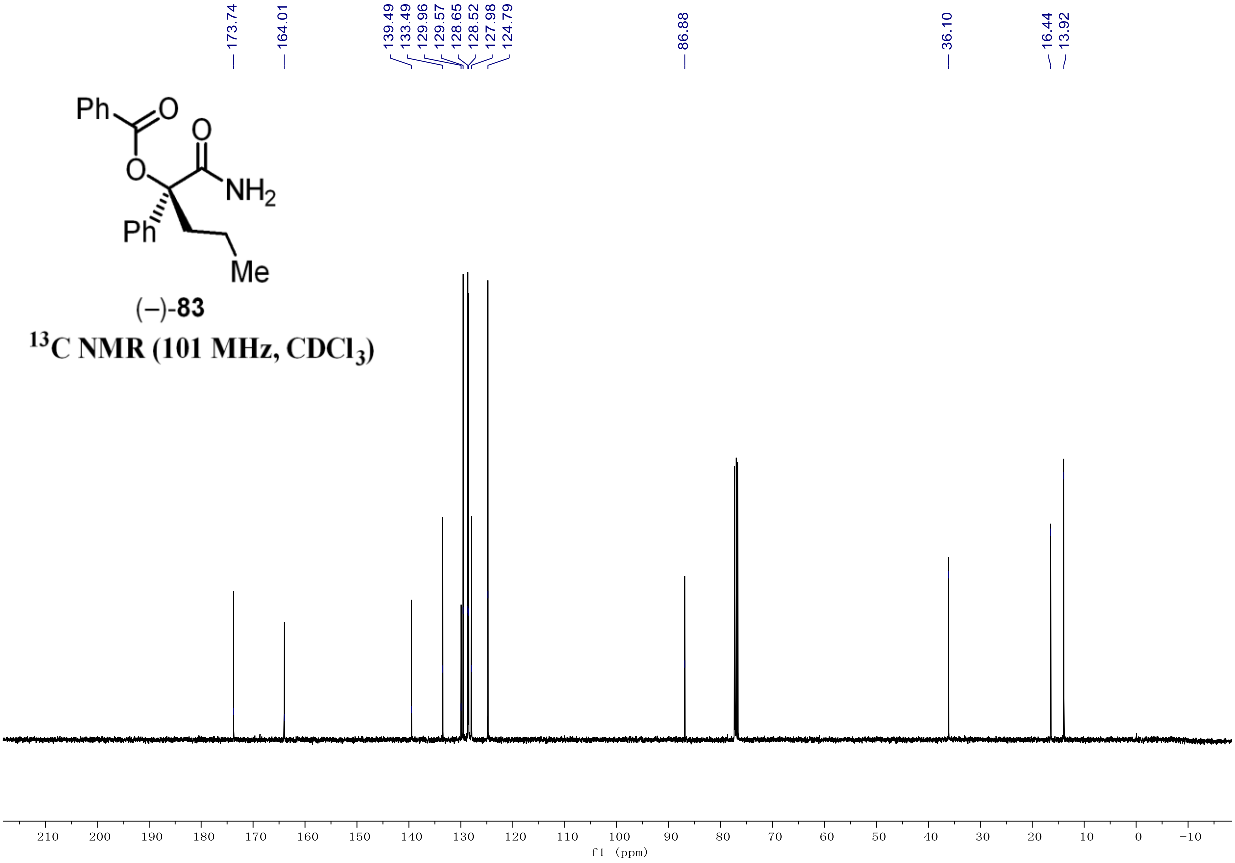
173.74 164.01 139.49 133.49 129.96 129.57 128.65 128.52 127.98 124.79 86.88 36.10 16.44 13.92
Ph
O
O
O
NH2
Ph
Me
(–)-83
13C NMR (101 MHz, CDCl3)
f1 (ppm)

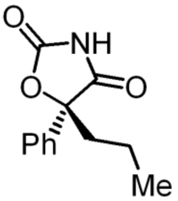

(+)-**84**

^{1}H NMR (400 MHz, $CDCl_3$)

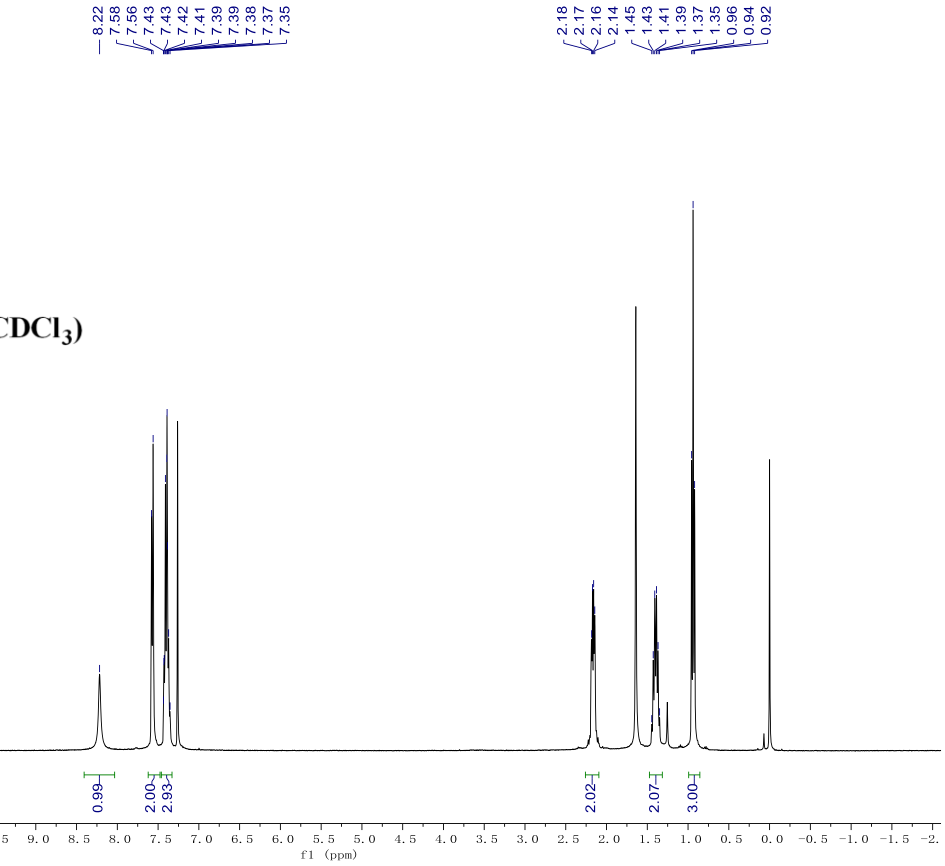

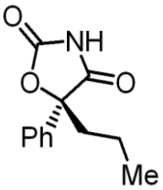

(+)-**84**

^{13}C NMR (101 MHz, $CDCl_3$)

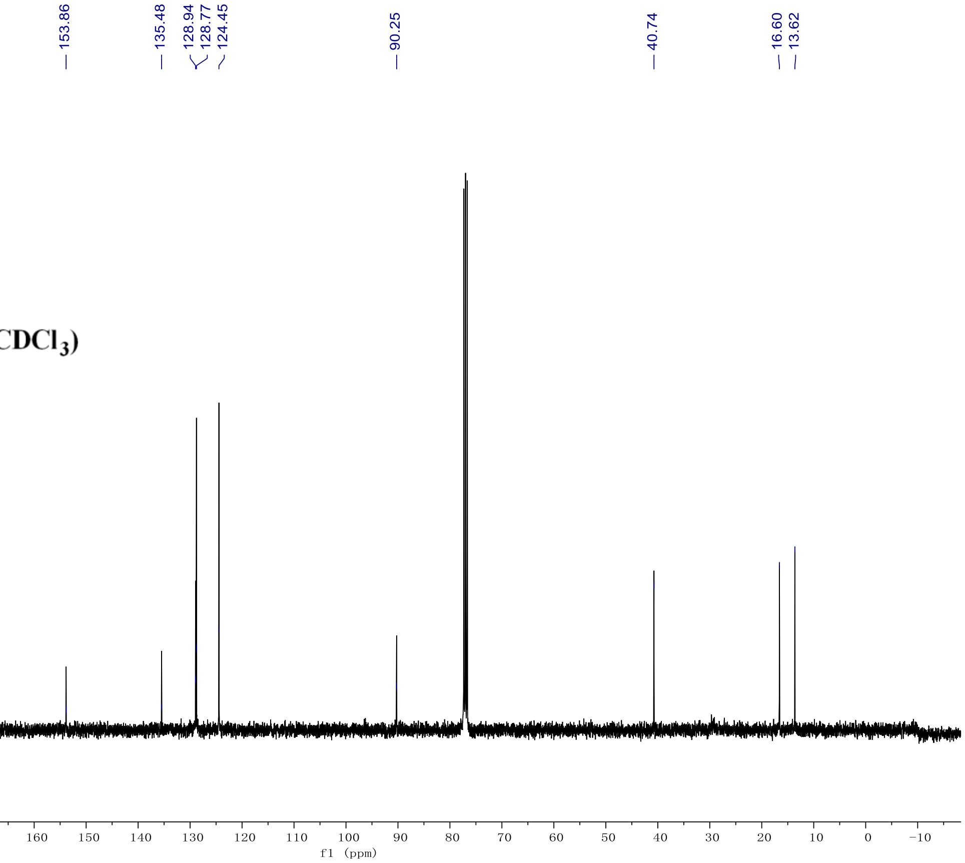

(+)-**85**

^{1}H NMR (400 MHz, CDCl$_3$)

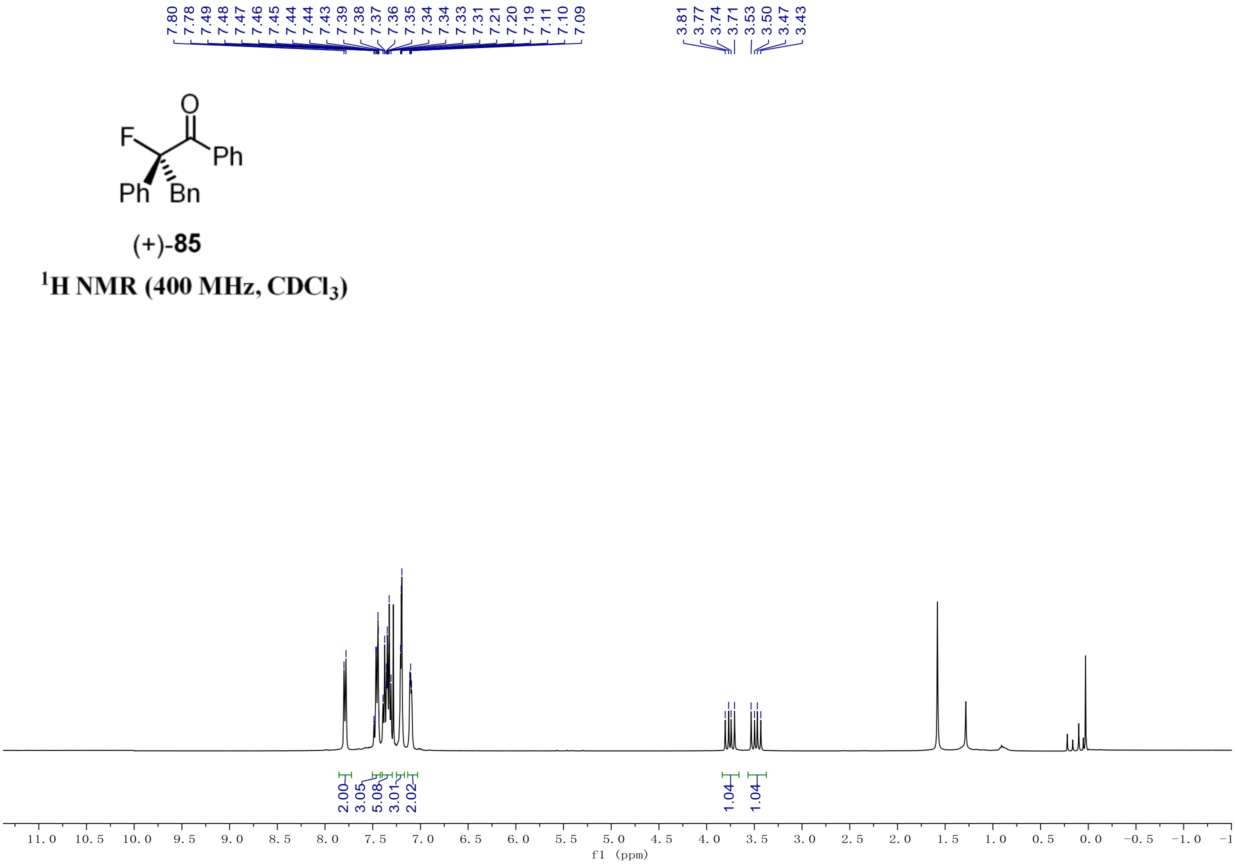

(+)-**85**

^{13}C NMR (101 MHz, CDCl$_3$)

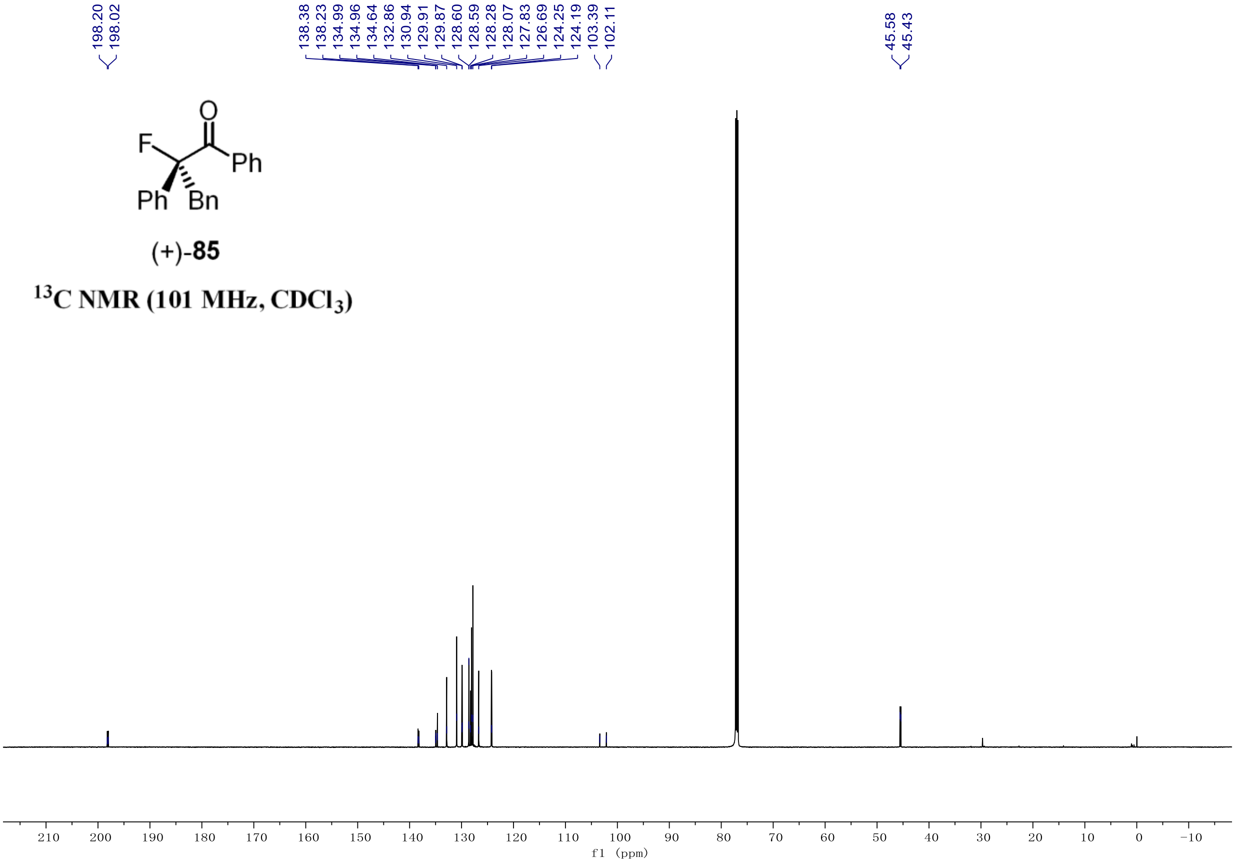

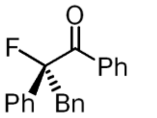

(+)-**85**

^{19}F NMR (376 MHz, CDCl$_3$)

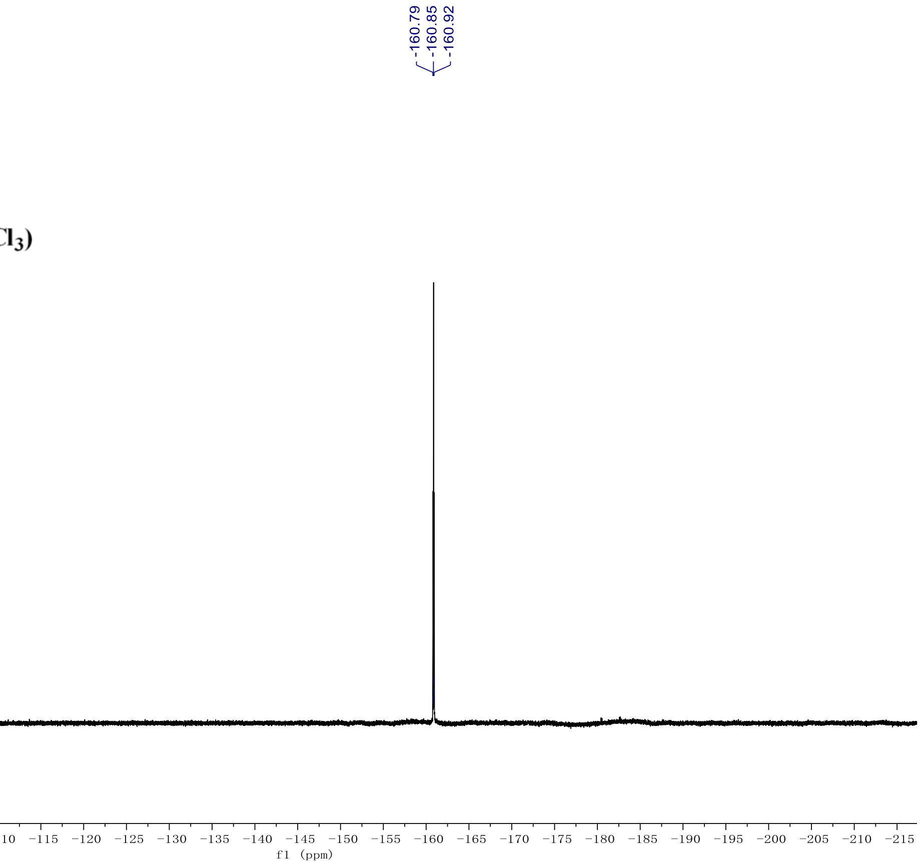

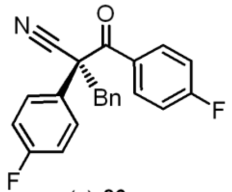

(+)-**86**

^{1}H NMR (400 MHz, CDCl$_3$)

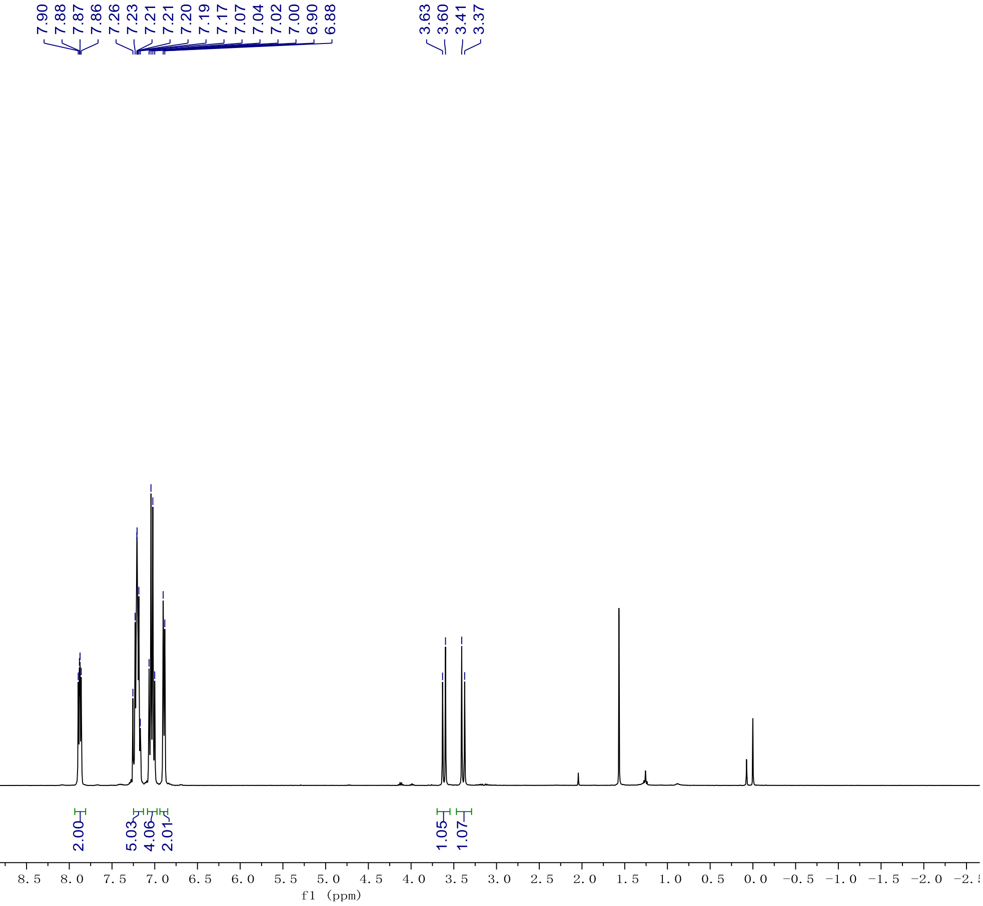

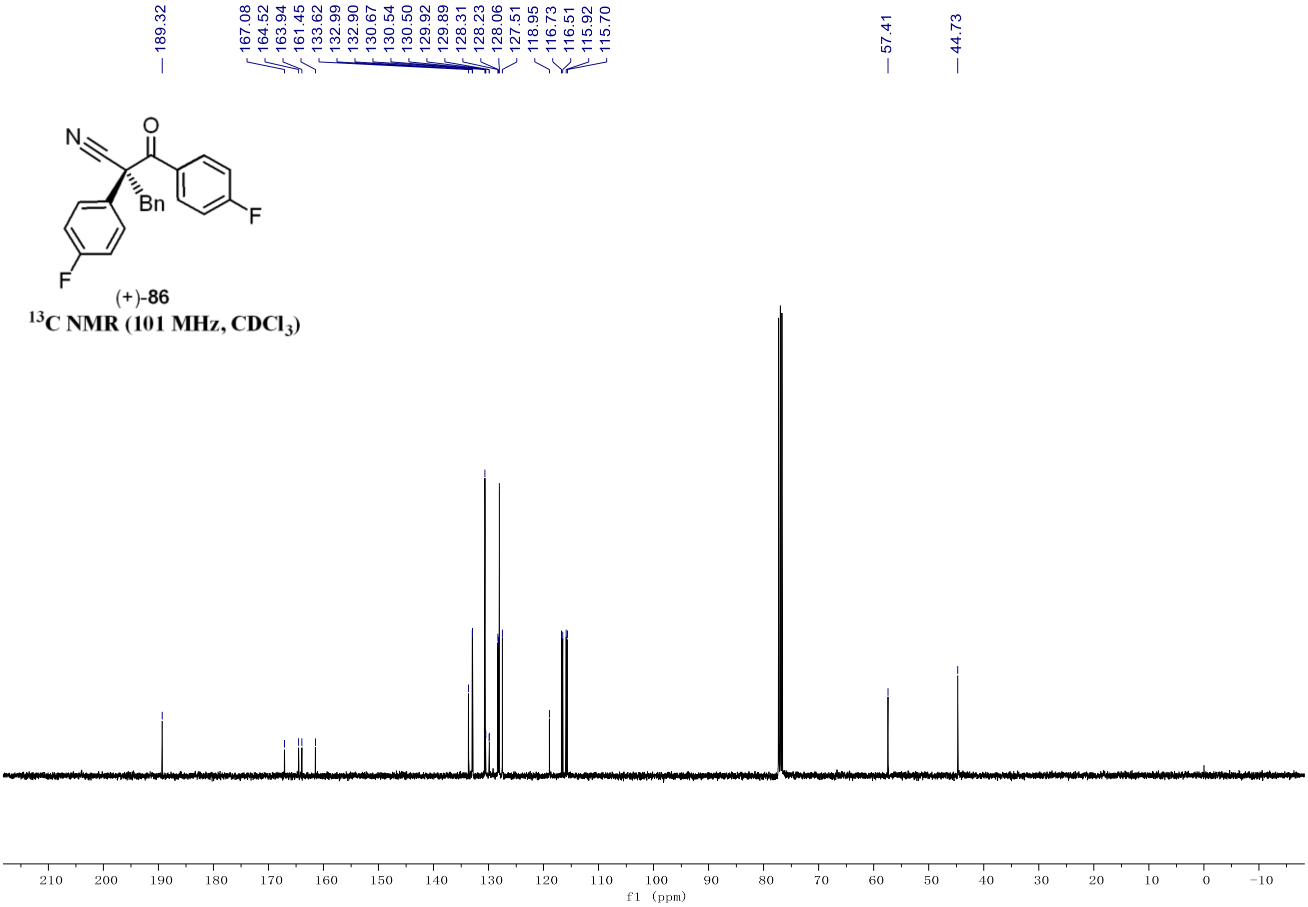

(+)-**86**

^{13}C NMR (101 MHz, $CDCl_3$)

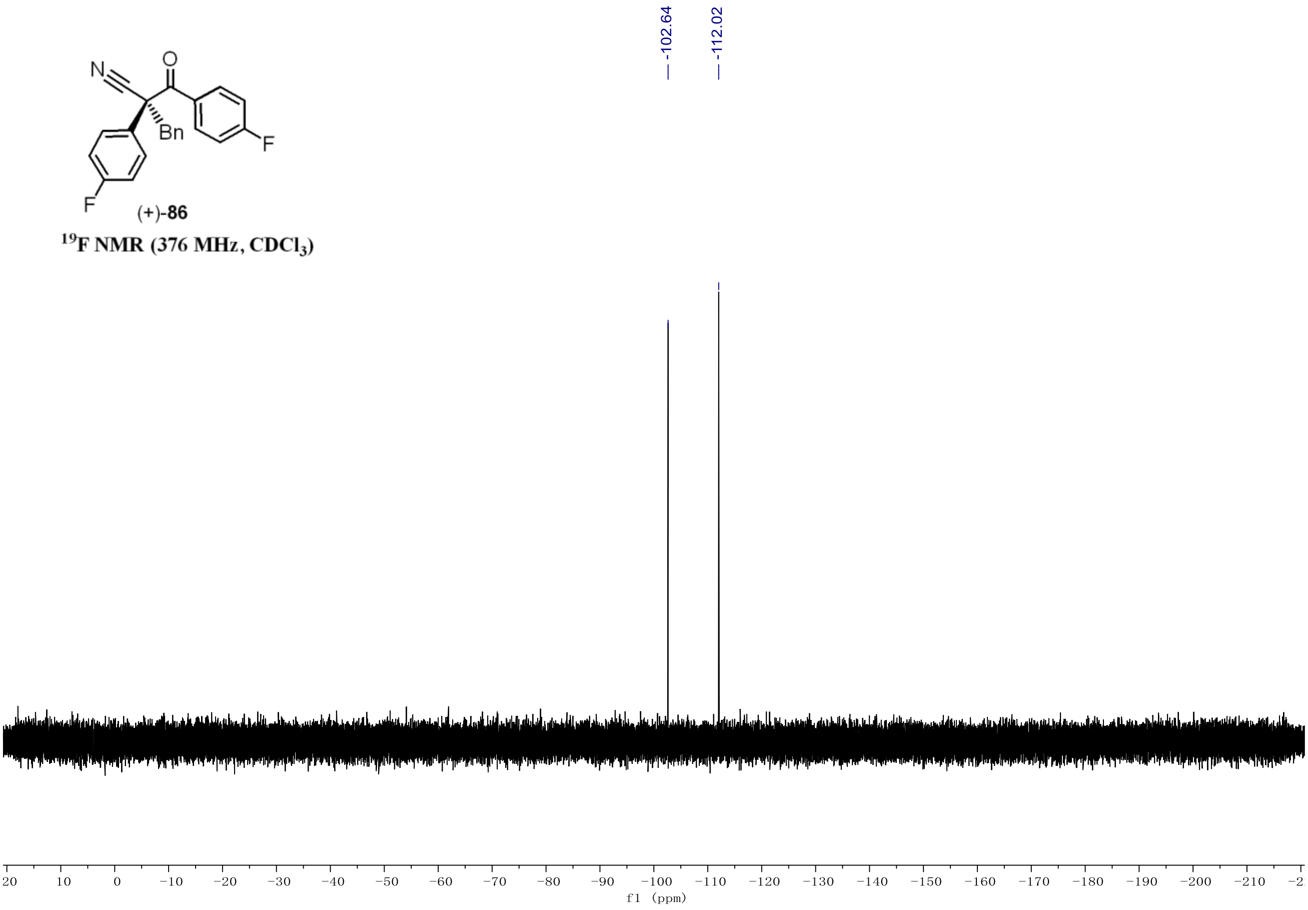

(+)-**86**

^{19}F NMR (376 MHz, $CDCl_3$)

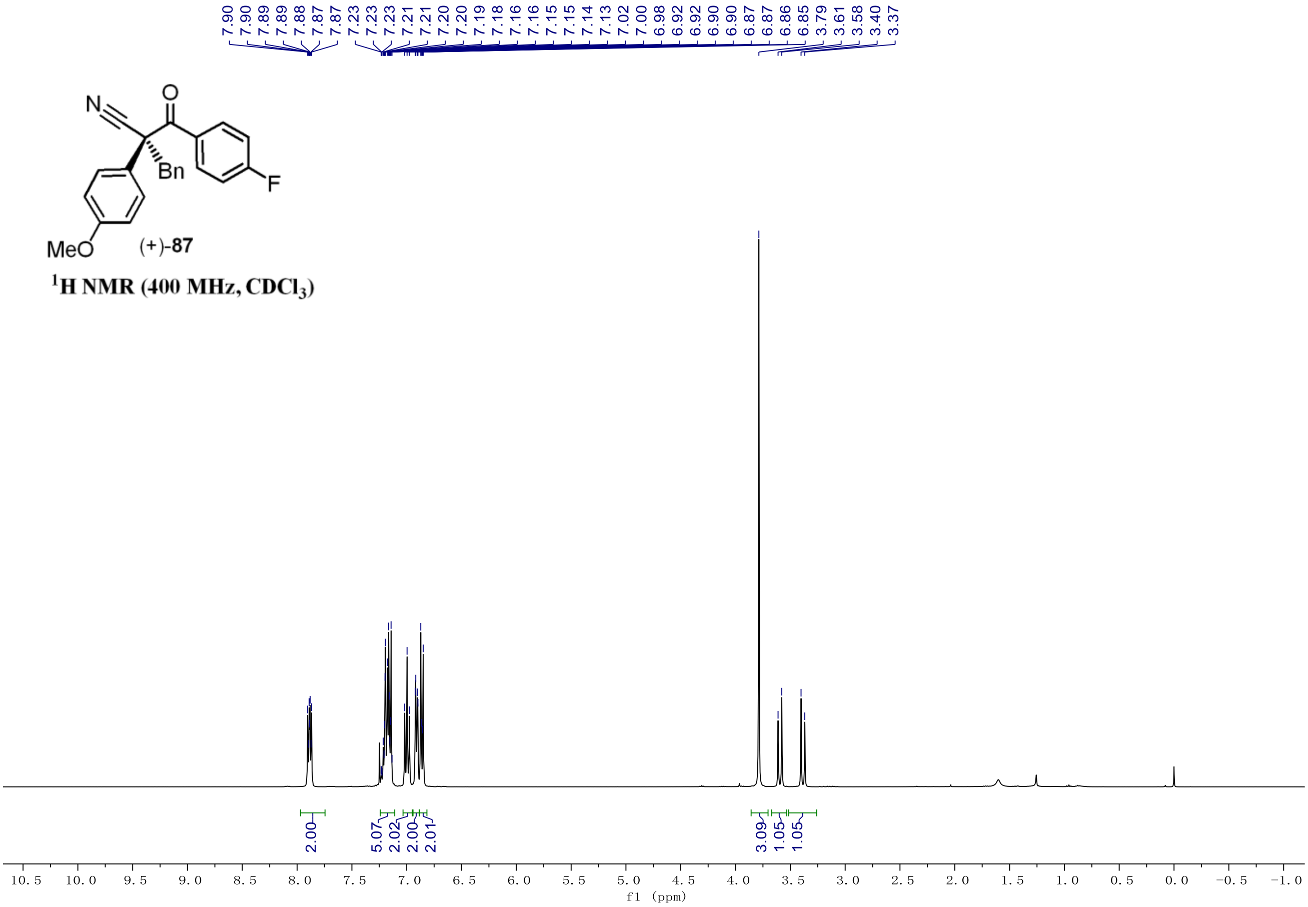

(+)-**87**

^{1}H NMR (400 MHz, $CDCl_3$)

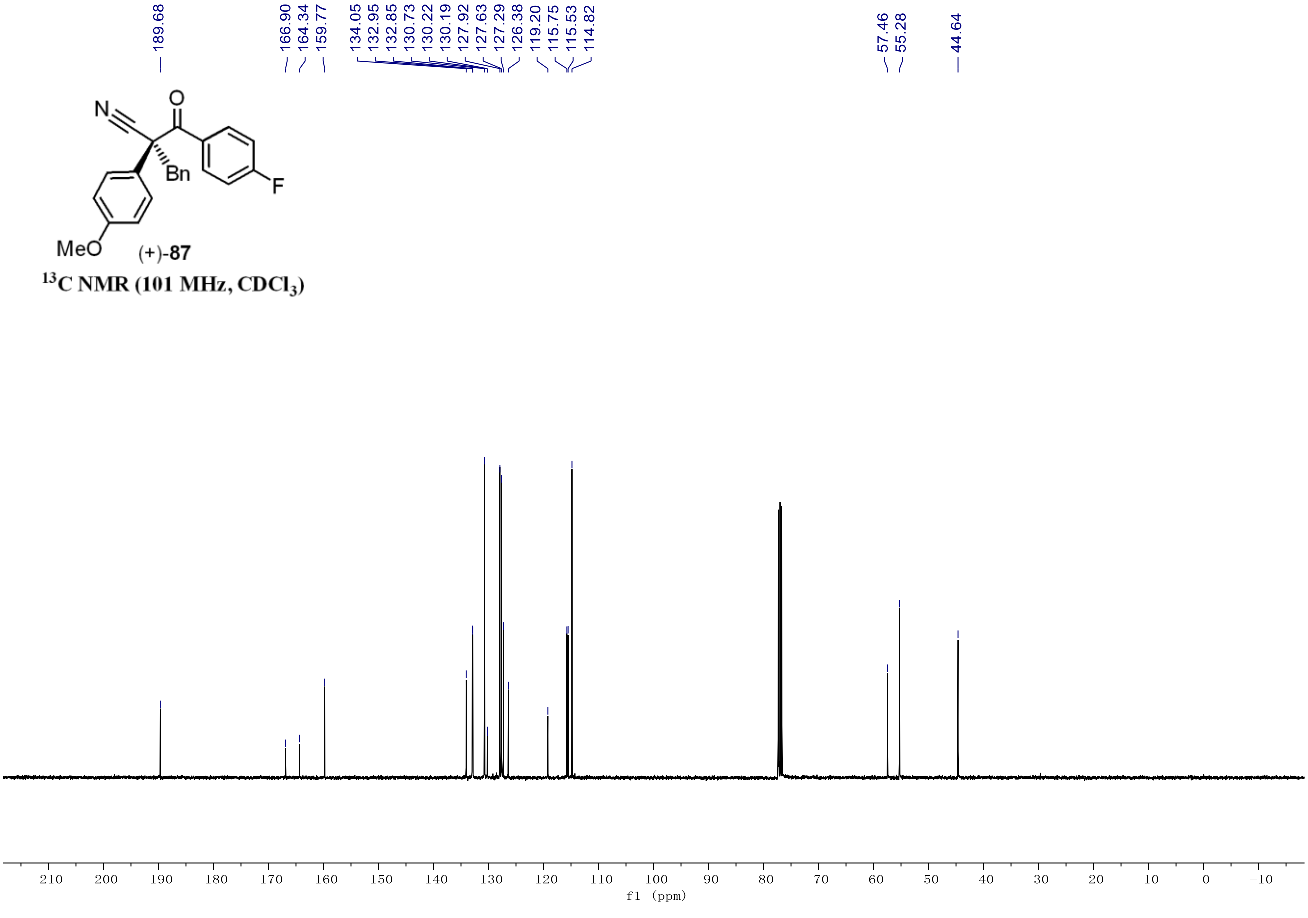

(+)-**87**

^{13}C NMR (101 MHz, $CDCl_3$)

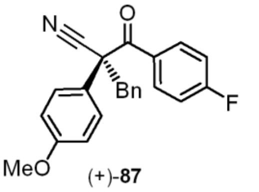

(+)-**87**

^{19}F NMR (376 MHz, CDCl$_3$)

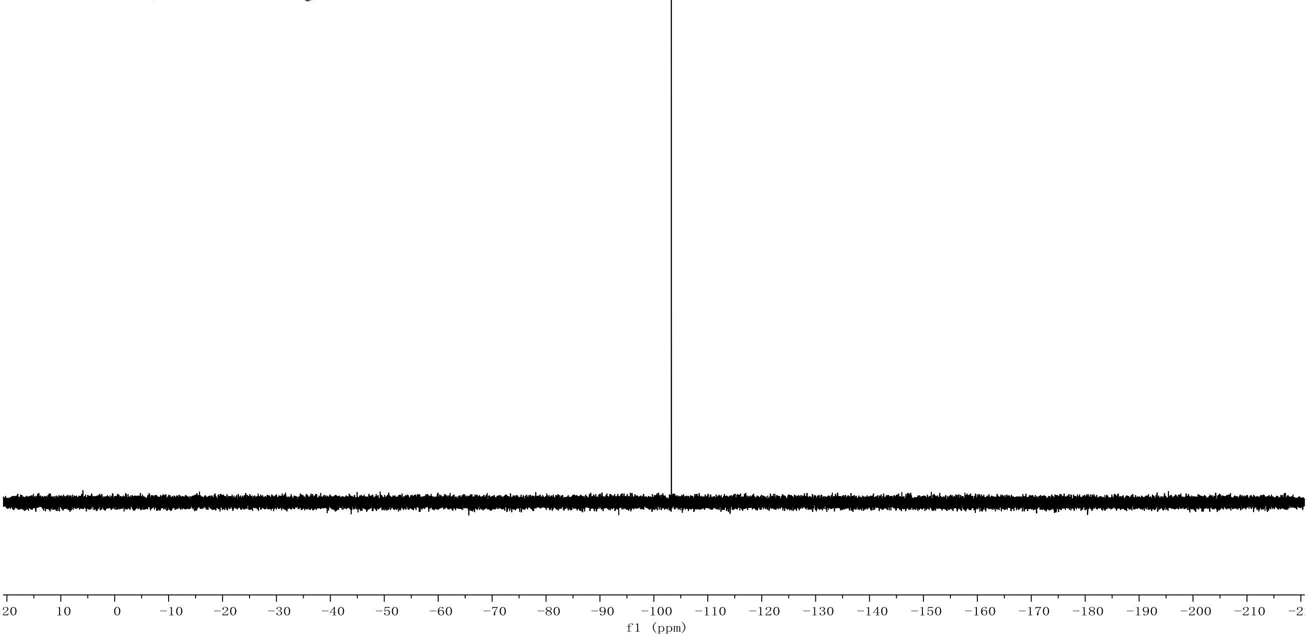

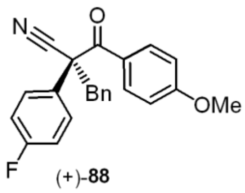

(+)-**88**

^{1}H NMR (400 MHz, CDCl$_3$)

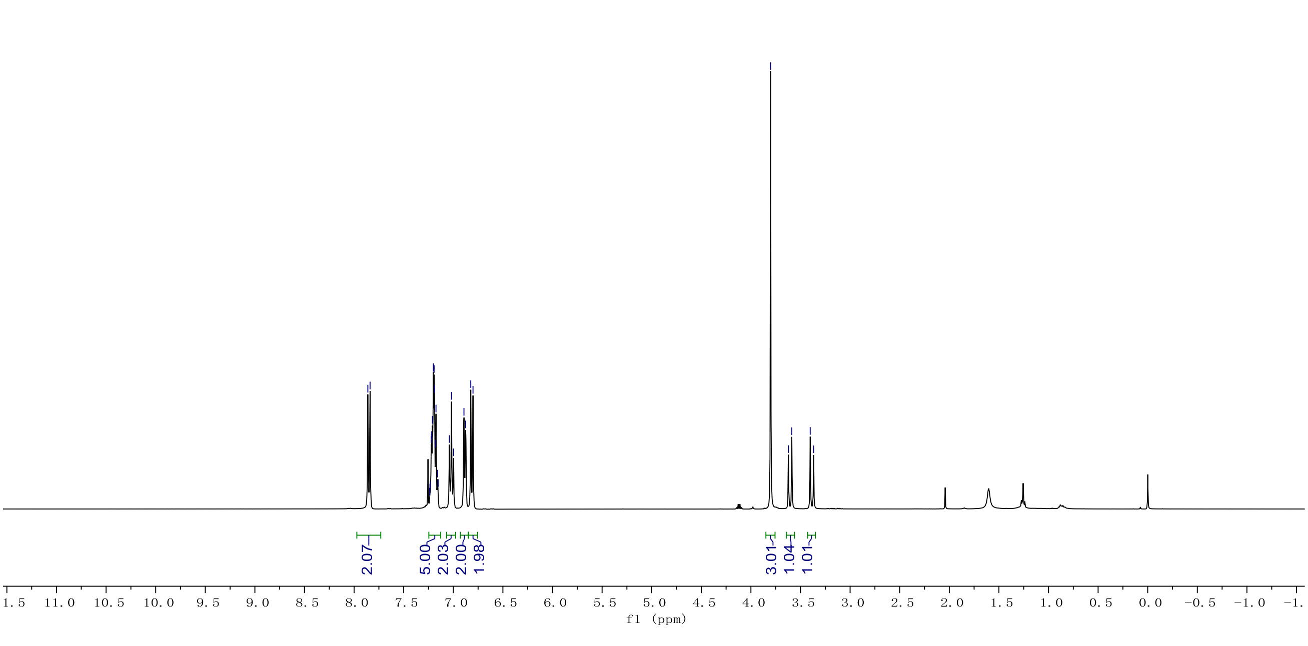

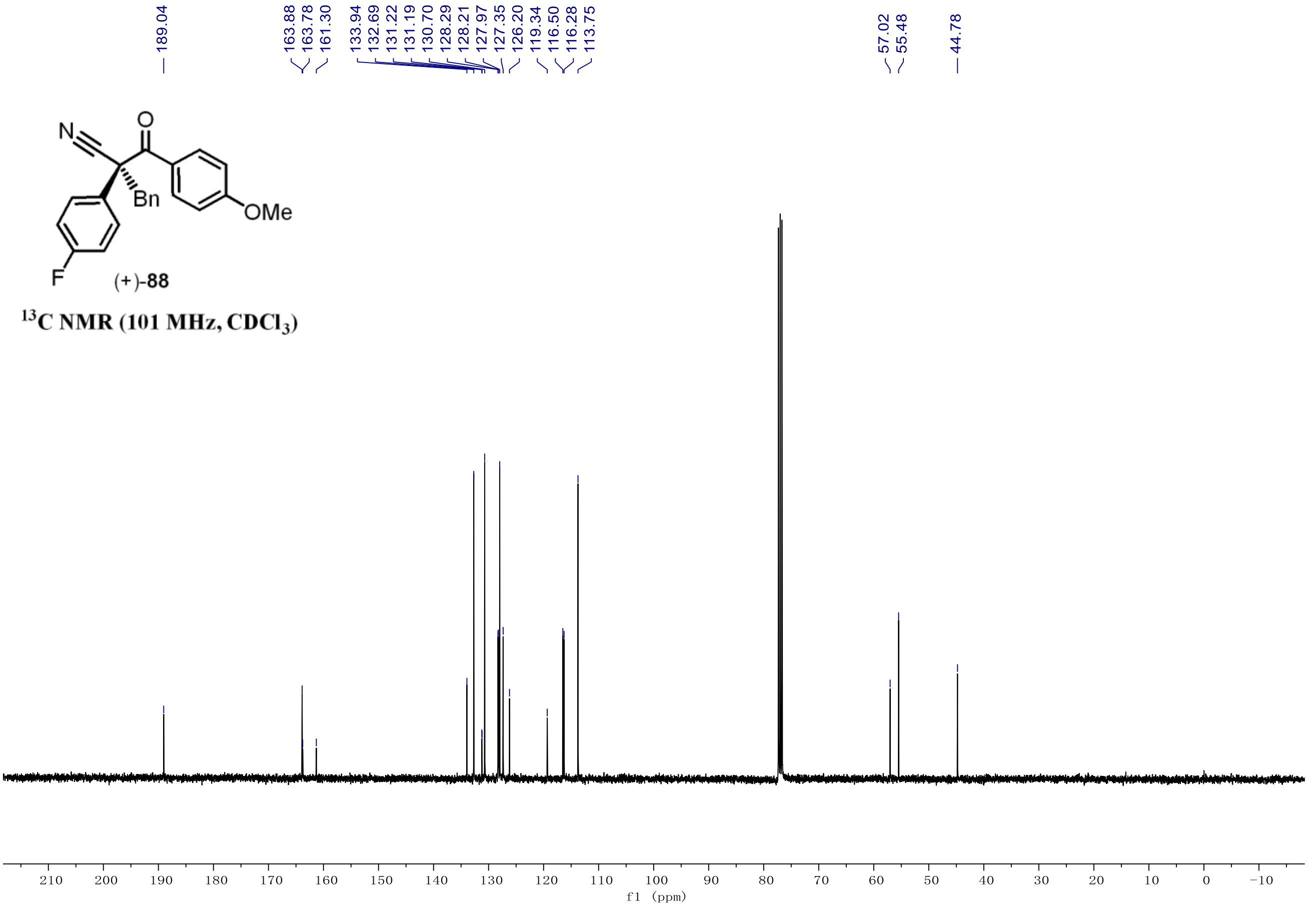

(+)-**88**

^{13}C NMR (101 MHz, CDCl$_3$)

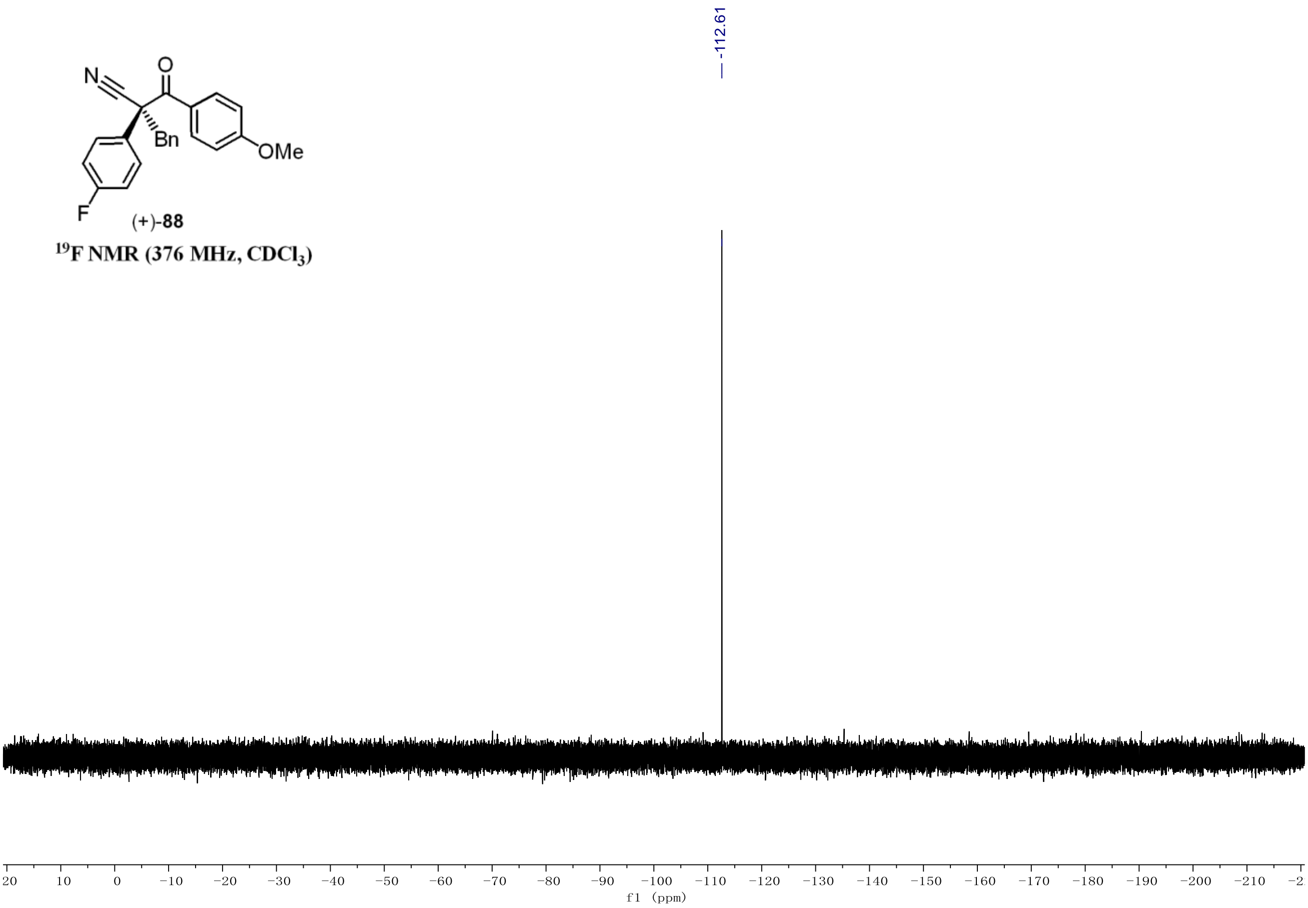

(+)-**88**

^{19}F NMR (376 MHz, CDCl$_3$)

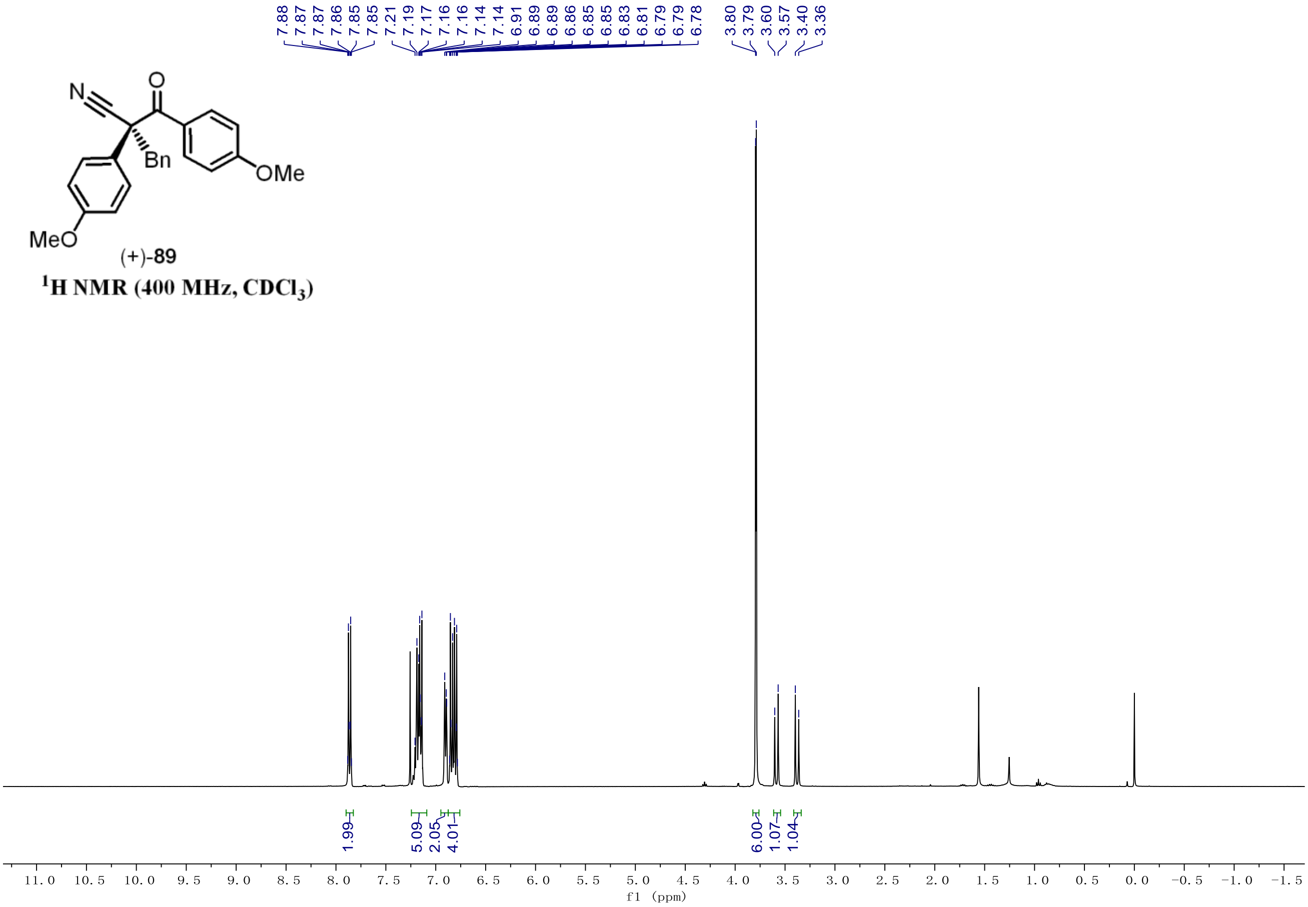
7.88 7.87 7.87 7.86 7.85 7.85 7.21 7.19 7.17 7.16 7.16 7.14 7.14 6.91 6.89 6.89 6.86 6.85 6.85 6.83 6.81 6.79 6.79 6.78
3.80 3.79 3.60 3.57 3.40 3.36
N
O
Bn
OMe
MeO
(+)-89
1H NMR (400 MHz, CDCl3)
1.99
5.09
2.05
4.01
6.00
1.07
1.04
11.0 10.5 10.0 9.5 9.0 8.5 8.0 7.5 7.0 6.5 6.0 5.5 5.0 4.5 4.0 3.5 3.0 2.5 2.0 1.5 1.0 0.5 0.0 −0.5 −1.0 −1.5
f1 (ppm)

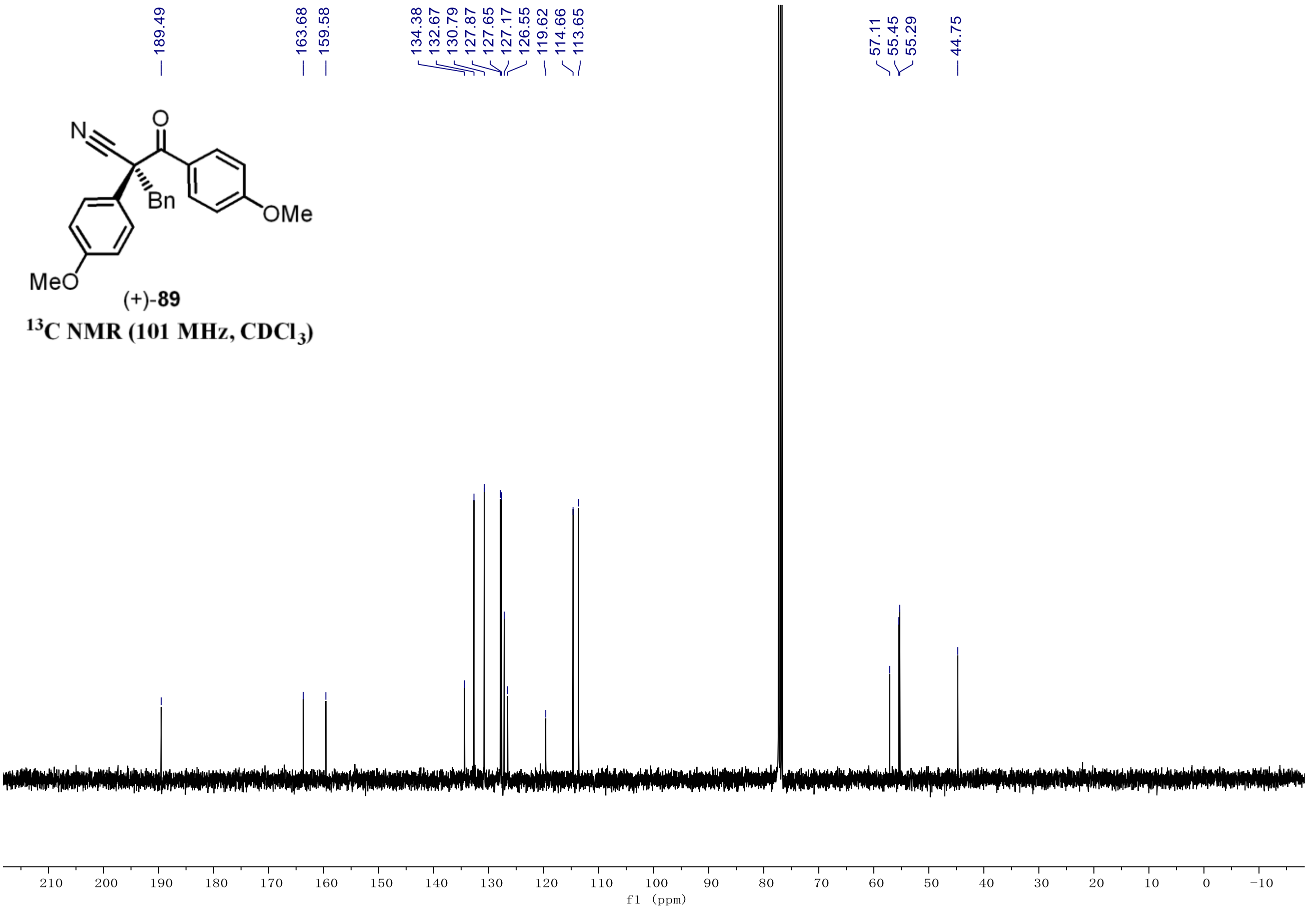
189.49
163.68 159.58
134.38 132.67 130.79 127.87 127.65 127.17 126.55 119.62 114.66 113.65
57.11 55.45 55.29
44.75
N
O
Bn
OMe
MeO
(+)-89
13C NMR (101 MHz, CDCl3)
210 200 190 180 170 160 150 140 130 120 110 100 90 80 70 60 50 40 30 20 10 0 −10
f1 (ppm)
